# Ultra-High Mass Resolution MALDI Imaging Mass Spectrometry of Proteins and Metabolites in a Mouse Model of Glioblastoma

**DOI:** 10.1038/s41598-017-00703-w

**Published:** 2017-04-04

**Authors:** M. Dilillo, R. Ait-Belkacem, C. Esteve, D. Pellegrini, S. Nicolardi, M. Costa, E. Vannini, E. L. de Graaf, M. Caleo, L. A. McDonnell

**Affiliations:** 1 Fondazione Pisana per la Scienza ONLUS - Via Panfilo Castaldi 2, 56121 Pisa, Italy; 2 Department of Chemistry and Industrial Chemistry - Università di Pisa - Via Giuseppe Moruzzi 13, 56124 Pisa, Italy; 3 Center for Proteomics and Metabolomics, Leiden University Medical Center, Leiden, The Netherlands; 4 NEST, Istituto Nanoscienze-National Research Council, 56127 Pisa, Italy; 5 CNR Neuroscience Institute, Via Moruzzi 1, 56124 Pisa, Italy; 6 Department of Pathology, Leiden University Medical Center, Leiden, The Netherlands

## Abstract

MALDI mass spectrometry imaging is able to simultaneously determine the spatial distribution of hundreds of molecules directly from tissue sections, without labeling and without prior knowledge. Ultra-high mass resolution measurements based on Fourier-transform mass spectrometry have been utilized to resolve isobaric lipids, metabolites and tryptic peptides. Here we demonstrate the potential of 15T MALDI-FTICR MSI for molecular pathology in a mouse model of high-grade glioma. The high mass accuracy and resolving power of high field FTICR MSI enabled tumor specific proteoforms, and tumor-specific proteins with overlapping and isobaric isotopic distributions to be clearly resolved. The protein ions detected by MALDI MSI were assigned to proteins identified by region-specific microproteomics (0.8 mm^2^ regions isolated using laser capture microdissection) on the basis of exact mass and isotopic distribution. These label free quantitative experiments also confirmed the protein expression changes observed by MALDI MSI and revealed changes in key metabolic proteins, which were supported by *in-situ* metabolite MALDI MSI.

## Introduction

Mass spectrometry imaging (MSI) is able to simultaneously record the distributions of hundreds of biomolecules directly from tissue, without labeling and without prior knowledge^1^. MSI based on matrix assisted laser desorption/ionization (MALDI) can be used to analyze proteins, peptides, glycans, lipids, metabolites and drugs, using essentially the same technology but different tissue preparation procedures^2–7^. MALDI MSI is particularly suited to biomedical research because the MSI datasets can be aligned with histological images of the MSI-analyzed tissue section. In this manner the molecular signatures from specific histopathological entities may be extracted from the often-heterogeneous tissues encountered in biomedical research. These capabilities have been used to determine molecular changes statistically associated with disease, metastatic status, patient prognosis and patient response to chemotherapy^2, 8, 9^.

The accuracy of the mass spectrometric signatures detected by MSI are intrinsic to its success; the large number of molecules detected within a single experiment often include isobaric species that have the same nominal mass but different exact mass. It has been demonstrated how high mass resolution instruments, such as Fourier Transform Ion Cyclotron Resonance (FTICR) and Orbitrap mass spectrometers, are able to resolve many of these isobaric species whereas the more commonly used time-of-flight systems amalgamate the signals. The benefits of high mass resolution have been repeatedly demonstrated for MALDI MSI of lipids, metabolites and proteolytic peptides^4–7^. Recently it was demonstrated how high mass resolution is also beneficial for MSI of intact proteins; the higher mass resolution resolved the isotopic distribution of individual protein ions, protein oxidation states, and thereby enabled more confident assignments^3, 10, 11^. However, the contribution of isobaric ions in protein MALDI MSI was not reported. The wide isotopic distributions of proteins mean that their isotopes can be isobaric (identical nominal mass) if the average masses of the proteins are similar; here we refer to this scenario as isobaric isotopomers. Overlapping, non-resolved mass spectral peaks undermine the ability to identify biomarkers because disease associated changes in expression are diluted by overlapping peaks; an effect similar to the ratio compression detected in LC-MS/MS with isobaric labeling strategies due to interfering co-eluting peptides^12, 13^.

Here we report an ultra-high mass resolution study, performed using a 15 T MALDI-FTICR mass spectrometer, of a murine model of glioblastoma multiforme (GBM). GBM is a highly malignant astrocytoma characterized by high infiltration, high heterogeneity, and poor prognosis^14–16^. The ultra-high mass resolution enabled GBM-associated proteoforms to be resolved, including proteins with interspersed and isobaric isotopomers. The presence of isobaric, isotopomer protein ions complicates the assignment of their identity, by comparison with LC-MS/MS analysis of protein extracts, because of the increased potential for false positives. To reduce the potential for false positives the 15 T MALDI-FTICR MSI results were aligned with the protein identifications obtained from LC-MS/MS analysis of extracts of small (0.8 mm^2^) defined histological areas isolated by laser capture microdissection (LCM).

## Results and Discussion

MALDI MSI provides the microscopy and molecular analysis capabilities necessary to resolve the spatio-molecular features of pathological tissue sections. The mass resolution defines the molecular specificity of the analysis. Here we assessed the importance of mass resolution for the analysis of intact proteins in pathological tissues. The ultra-high mass resolution of 15 T FTICR MSI not only allowed the detection of intact proteins between 3.5 and 16 kDa with full isotopic resolution, it also led to the detection of a much larger number of distinct protein ions, distinguished proteoforms and enabled the charge states and adduct types of many protein ions to be determined. We could assign identities to 94 isotope distributions from different protein ions. The full list of proteoforms and adducts can be found in the Supplementary Table ST1.

### Comparison of MALDI-FTICR MSI and MALDI-TOF MSI datasets

**Figure 1:**
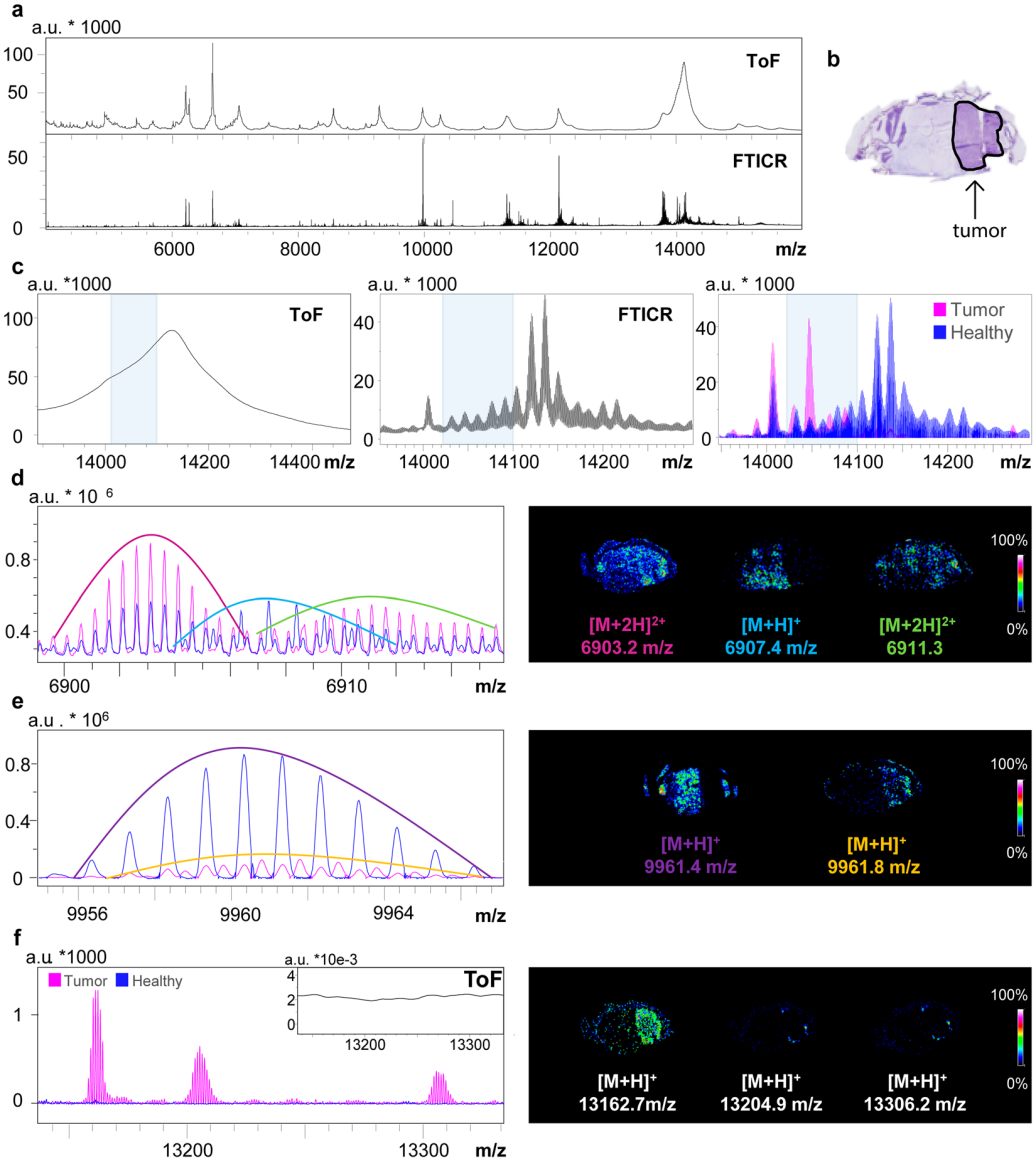
Insight into the complexity of the high mass resolution MSI data: (**a**) Comparison of the average mass spectra obtained from consecutive coronal mouse brain tissue sections with MALDI–ToF MSI and MALDI–FTICR MSI. (**b)** Scanned image of the Nissl-stained mouse brain tissue, the solid black line highlights the tumor. (**c)** From left to right: average MALDI TOF mass spectrum (left), average MALDI FTICR mass spectrum (center), and overlay of the average MALDI FTICR mass spectra from the tumor (pink) and healthy (blue) regions. The shaded region highlights tumor specific proteoforms that could only be revealed with the MALDI-FTICR. (**d)** The ultra-high resolution of the MALDI-FTICR instrument distinguished two doubly charged proteoforms of Histone H2B and a different singly charged protein. (**e)** MALDI-FTICR spectra showing the differentiation of two protein ions of similar mass with interspersed and isobaric isotopomers, and which have differential localization. (**f)** Overlaid average mass spectra of tumor and healthy regions showing the specific distribution of three ions that could not be detected with MALDI-TOF MSI (MALDI-TOF mass spectrum shown in insert).

The difference in the richness of the mass spectral information obtained by MALDI-TOF MSI and high field MALDI-FTICR MSI is shown in Fig. 1a, which shows the full-section average MSI mass spectra obtained with MALDI-ToF and MALDI FTICR from consecutive GBM mouse brain tissue sections. The difference in the information content is readily apparent on closer inspection, Fig. 1c. For example within the *m/z* range 14,000–14,250 the average mass spectrum of the MALDI-TOF MSI dataset contains only a broad peak with a non-resolved shoulder; the analogous region of the MALDI-FTICR spectrum displays a long series of isotopic distributions of distinct protein ions.

The average mass spectra of the MALDI-FTICR MSI datasets were extremely data rich; they contained proteins detected in multiple charge states, different proteoforms, as well as interspersed and isobaric isotopic distributions. To focus on disease-associated changes in expression the average mass spectral signatures were extracted from distinct regions of interest, defined on the basis of the aligned histological images. The right-hand panel in Fig. 1c shows a comparison of the average mass spectrum from a tumor region (pink) with that from a healthy region (blue). It can be seen that this histology-defined approach rapidly highlighted changes in proteoform expression that were not apparent in the average mass spectrum of the entire tissue section (Fig. 1c, middle panel).

Additional examples of protein ions with interspersed and isobaric isotopomers, including multiply charged proteins ions and proteins that exhibit higher expression in the tumor region, are included in Fig. 1. Figure 1d shows two doubly charged protein ions whose isotopic distributions are resolved but interspersed with the resolved isotopic distribution of a singly charged protein ion. With the MALDI-TOF system, these three protein ions could not be resolved.

Figure 1e shows an example in which ultra-high mass resolution MALDI MSI enabled the distinction of two protein ions with interspersed, isobaric isotopomers, one of which was exclusively present in the tumor area and the other in the healthy region. These two protein ions differed in average mass by approximately 0.4 Da (their corresponding images were obtained from single isotopes to ensure molecular specificity).

The FTICR and TOF based systems used here differ markedly in how the mass spectrometry analysis is performed. While previous MSI reports have focused on the higher mass resolution and mass accuracy of FTMS based mass analyzers, or the higher speed and mass range of TOF based systems, their chemical backgrounds also differ. In the linear TOF systems used for protein MALDI MSI metastable ions contribute to a significant chemical background, which is normally subtracted from each pixel’s individual mass spectrum prior to the calculation of the images and the MSI dataset’s average mass spectrum^17^. Nevertheless even after background subtraction it has been shown that the residual chemical background can overwhelm the contributions from localized proteins^18^. This chemical background is largely absent in MALDI-FTICR MSI datasets because metastable ions do not survive the longer time scale of the mass analysis process (10’s of microseconds for a linear TOF vs. 0.5–3 s for the FTICR). Accordingly, localized signals are much better represented in the average mass spectrum of the MALDI-FTICR MSI dataset. Figure 1f shows examples of protein ions detected by MALDI-FTICR MSI that were localized to the tumor border region, but which could not be detected in the analogous MALDI-TOF MSI dataset.

### Alignment of MALDI MSI with LC-MS/MS

The ultra-high mass resolution and accurate mass data provided by MALDI-FTICR MSI allows the MSI data to be aligned with public databases of proteins commonly detected by MALDI MSI and with the results of LC-MS/MS of protein extracts. The presence of protein ions of similar mass, with interspersed and isobaric isotopomers, in specific tissue locations (Fig. 1) increases the risk of false positive assignments. To reduce this risk laser capture microdissection was used to isolate small 0.8 mm^2^ regions of tumor and healthy tissue from each tissue section, from which protein extracts were analyzed by bottom-up proteomics. In this manner the protein assignments could be performed while maintaining regional specificity, *i.e*. the protein ions detected by MALDI MSI from the tumor region (pink spectrum in Fig. 1) were assigned using only proteins identified from the microdissected tumor region. All protein ions were assigned on the basis of high mass accuracy, <10 ppm, and a high correlation between the experimental and theoretical isotope distributions (performed in Matlab, Pearson correlation, r ≥ 0.95).

**Figure 2:**
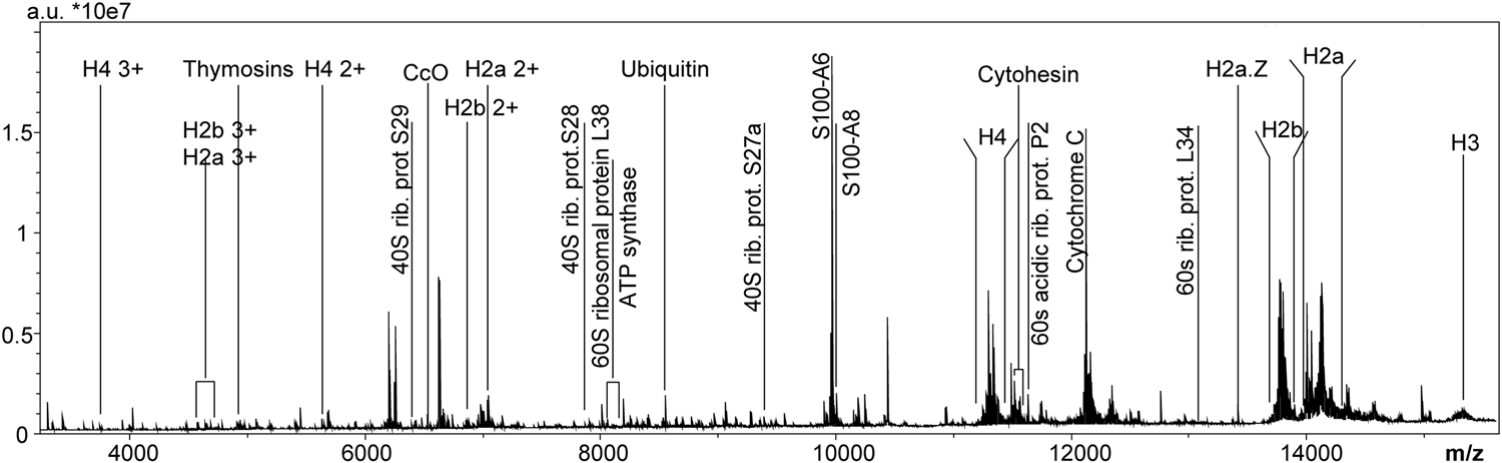
Average mass spectrum of a MALDI-FTICR MSI dataset of an entire mouse brain tissue section: annotation highlights most important and most abundant proteins identified with the support of the LCM-LC-MS/MS data.

The average mass spectrum of a protein MALDI-FTICR MSI dataset from the glioma mouse model is shown in Fig. 2, in which the most intense protein signals and specific proteins of interest are annotated. The list of proteins that could be assigned to the MALDI-FTICR MSI data is reported in Supporting Table ST1.

**Figure 3:**
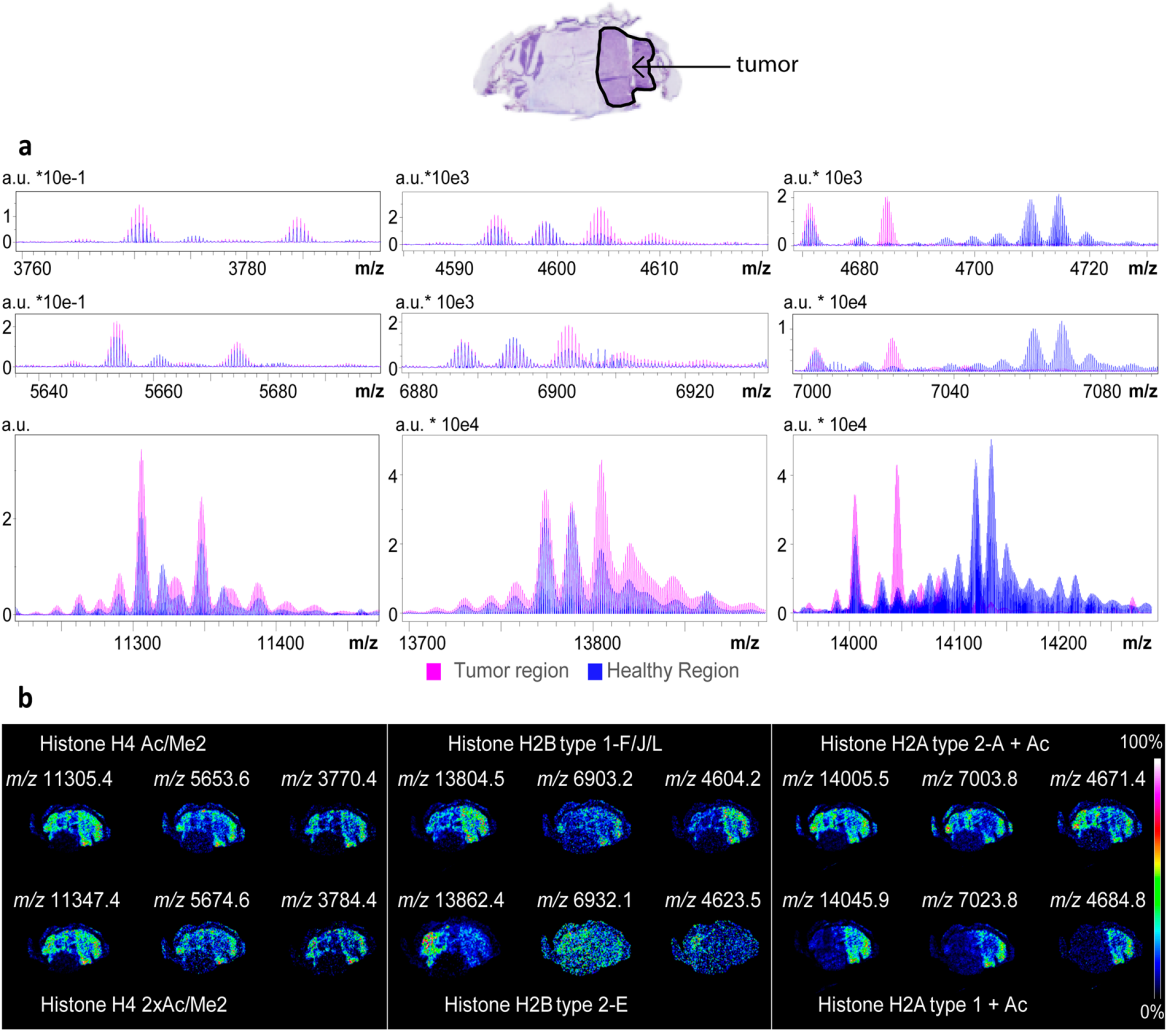
Differential regulation of histone proteoforms. (**a**) Overlay of the average mass spectra extracted for the tumor ROI (pink) and the healthy ROI (blue), showing the mass ranges of the triply (top), doubly (middle), singly (bottom) charged ions of Histone H4 (left), Histone H2B (middle), Histone H2A (right). (**b**) MS images for some of the detected proteoforms; protein assignments were made via alignment with the LC-MS/MS results for microdissected samples of tumor and healthy regions of tissue.

The ultra-high mass resolution protein MALDI-FTICR MSI data included histones H2, H3 and H4 and clearly resolved many of the post-translational modifications of these heavily modified proteins, and in which the acetylation and methylation status was dependent on whether tumor or healthy regions were analyzed. These basic proteins were detected as singly, doubly and triply charged ions (Fig. 3a). The same distributions were obtained for each charge state, thus providing internal verification of the distribution of each proteoform, Fig. 3b.

The protein ions detected in the mass range *m/z* 14000–14250 reported in Fig. 1c correspond to Histone H2A and its proteoforms, and the differential proteoform expression highlighted corresponds to an increase in acetylation in the tumor region. The two doubly charged ions reported in Fig. 1d could be identified as Histone H2B type 1.F/J/L and its methylated proteoform.

The different methylation and acetylation states of histones H2A, H2B, H3 and H4 detected by MALDI-FTICR MSI are well established in tumor biology^19–21^. Histone modifications are associated with gross structural chromatin changes. Acetylation modifies the charge state of the histone tails, influencing their interaction with DNA; methylation doesn’t alter the overall charge state but regulates the binding of effector molecules, like transcription factors, chromatin remodelers or chromatin structure proteins^22–24^. Previous MALDI-TOF MSI analyses of histones have resolved different histone variants and acetylation status, but not methylation status because of insufficient mass resolution^9, 25^.

The alignment of the MALDI-FTICR MSI data with the localized LC-MS/MS analysis (of microdissected regions of tissue) highlighted other proteins of interest that have previously been associated with cancer in MALDI MSI investigations. The pleiotropic actin-sequestering polypeptides, thymosin β4 (*m/z* 4964.3) and thymosin β10 (*m/z* 4937.3) were identified. These small proteins, known to be involved in wound healing and developmental processes, have been found by MALDI MSI to be associated with survival and recurrence for malignant melanoma patients^26^. Thymosin β4 expression has been proposed as a novel molecular target for anti-glioma therapy^27^, while thymosin β10 has been associated with invasion and metastasis of several kinds of tumors since it participates in the regulation of cancer cell motility^28^. Other proteins considered to play a role in tumor progression were also identified, including Calcyclin (S100A6, *m/z* 9961.5)^29^ and Cytochrome c oxidase (CcO Ac/2Ox, *m/z* 6648.02**)**. CcO is an apoptosis-related protein with an increased activity in a significant subset of high-grade glioma patients, and is considered an independent predictor of poor outcome and a useful marker for the categorization and targeted therapy of GBM^30^.

The alignment of the MALDI-FTICR MSI data with the results of localized LC-MS/MS enabled the identification of many ribosomal proteins. Ribosomal proteins of both small and large subunits (40 S and 60 S) have been found to be expressed at higher levels in a number of different cancers, including glioblastoma and other brain tumors^31, 32^. We could assign 40 S ribosomal protein S27a (*m/z* 9405.6), 40 S ribosomal protein 29 (*m/z* 6546.5), 40 S ribosomal protein S28 (*m/z* 7844.2), 60 S ribosomal protein L38 (*m/z* 8073.6), 60 S acidic ribosomal protein P2 (*m/z* 11710.1), 60 S Ribosomal protein L34 (*m/z* 13163.1) and 40 S ribosomal protein S25 (*m/z* 13758.4), all of which were detected at higher levels in the glioma region (Supporting Figure SF1).

### Semi-quantitative comparison of MALDI-FTICR MSI and LC-MS/MS datasets

**Figure 4:**
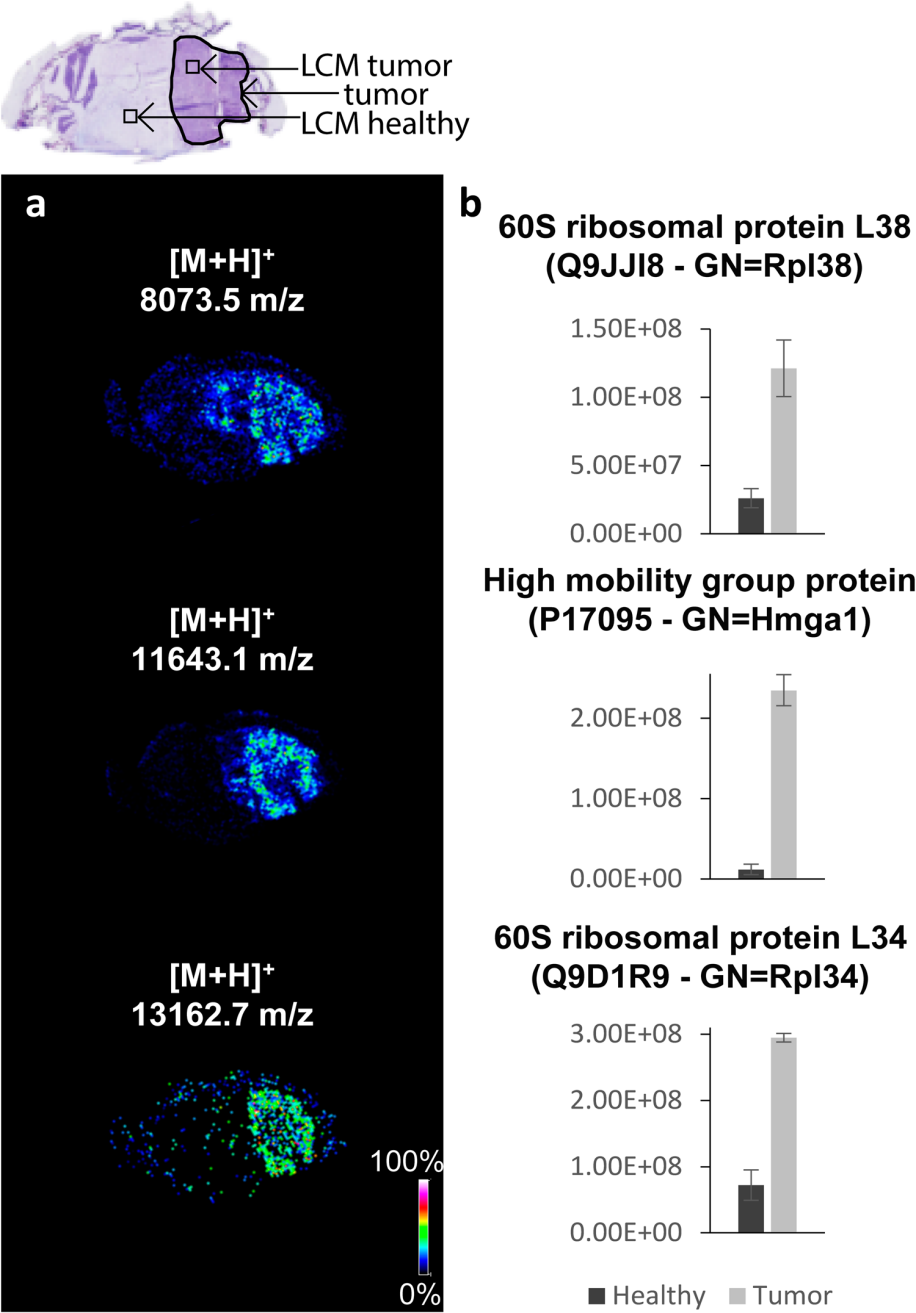
Comparison of LC-MS/MS and MALDI MSI data: the agreement between the fold-changes determined by LC-MS/MS of microdissected regions (microdissected regions are indicated in top histological image) and MSI datasets is demonstrated for three protein ions. In each case the LC-MS/MS relative quantification confirmed that observed with MALDI MSI. (**a**) Images extracted from the MSI dataset for 60 S ribosomal protein L38, High mobility group protein, 60 S ribosomal protein L34 (from the top). (**b**) LC-MS/MS relative quantification: the bar charts report the sum of the areas of each protein’s tryptic peptides (data are mean area ± standard deviation).

0.8 mm^2^ regions of healthy and tumor regions were isolated from three consecutive tissue sections for localized protein identification and relative quantitation. In addition to enabling the assignment of identities to the protein ions detected by MALDI MSI the label free quantification experiments confirmed the fold-changes detected by MALDI-FTICR MSI. Figure 4 shows three examples of the agreement between the MALDI-FTICR MSI and LCM-LC-MS/MS results. The distributions recorded by MSI of 60 S ribosomal protein L38 (UniProt Q9JJI8; *m/z* 8073.6), high mobility group protein (UniProt P17095; *m/z* 11643.3) and 60 S ribosomal protein L34 (UniProt Q9D1R9; *m/z* 13163.1) all showed an increased signal intensity in the tumor region (Fig. 4a). The label free quantitation obtained by LC-MS/MS showed an analogous fold-change between the healthy and tumor regions (Fig. 4b). The concordance between the expression changes detected by the LCM-LC-MS/MS microproteomics analysis and the MALDI-FTICR MSI data (Fig. 4), combined with the accurate mass measurements and isotopic distributions provided by high field MALDI-FTICR MSI, allows protein identities to be assigned to MALDI-FTICR MSI data with more confidence.

### MALDI MSI of metabolites: an insight in the tumor metabolism

**Figure 5:**
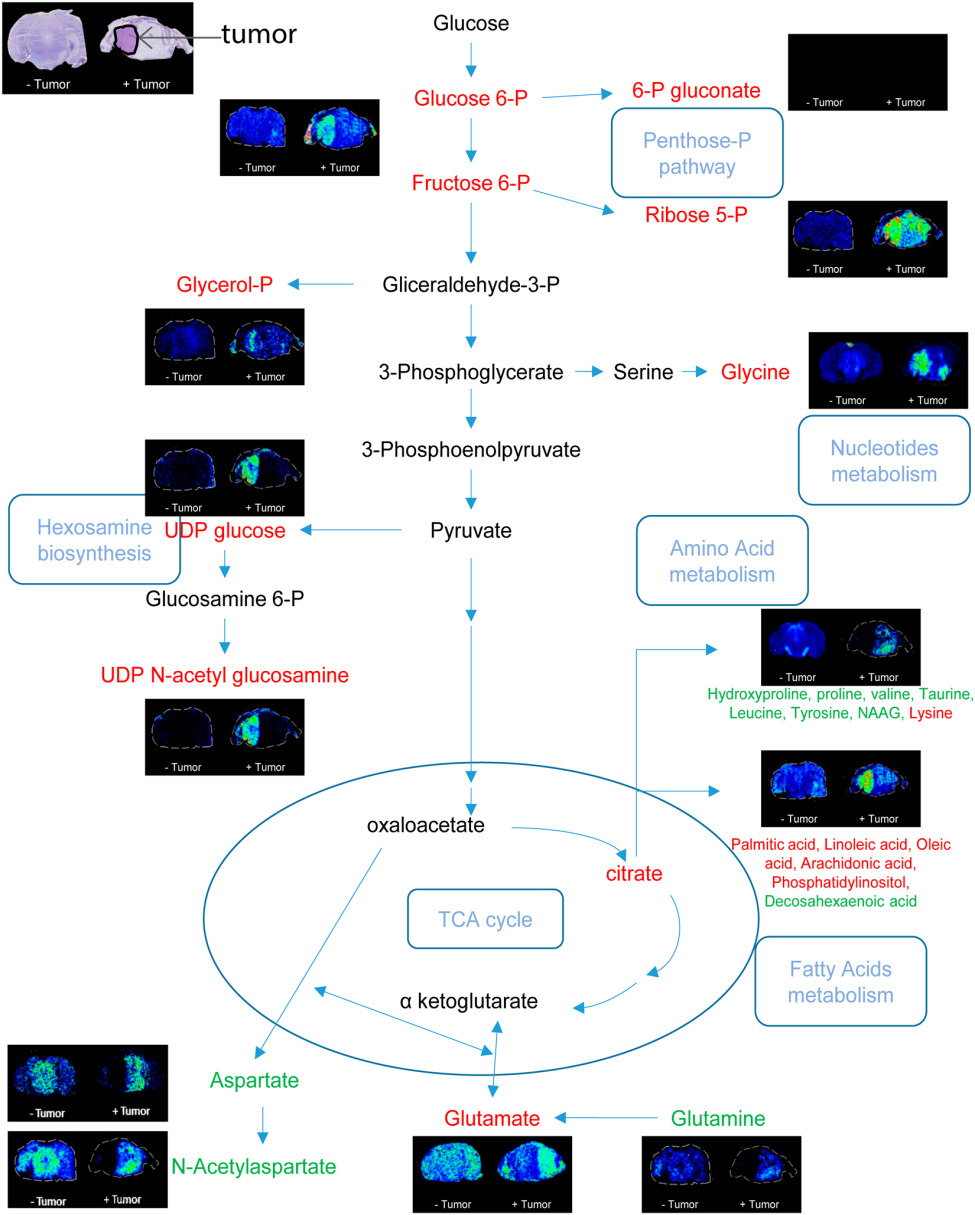
Qualitative metabolite analysis by MALDI-FTICR MSI. Nissl stained tissue sections are shown in the top left, with the tumor region of interest indicated by an arrow. Images acquired for brain metabolites are reported in a scheme that reproduces the glutaminolysis and some steps of the TCA cycle. Metabolite ions detected at lower levels in the tumor are indicated in green and at higher levels in red. Metabolites were assigned on the basis of previous MALDI MSI investigations utilizing 9-aminoacridine as matrix. Supporting Table ST2 lists all of the metabolites that could be assigned on the basis of accurate mass and isotopic distribution.

The microproteomics analysis also revealed a number of proteins involved in metabolic pathways that exhibited differences in expression levels. The role of the identified proteins was traced back to specific pathways using the PANTHER classification system^33^. The proteins are enzymes involved in glutamine anabolic processing and the TCA cycle (Protein groups are listed in Supplementary Protein List PL1 and PL2). Variations in tumor cellular metabolism are a hallmark of cancer^34^. MALDI MSI is also able to analyze metabolites, through applying different tissue preparation methods and adapting the settings of the mass spectrometer for the lower molecular mass of metabolites. Figure 5 summarizes the metabolites that were detected using 15 T MALDI-FTICR MSI of consecutive tissue sections, and in which the ultra-high resolution and mass accuracy enabled elemental formula to be assigned and isotope patterns to be matched (full list of assigned metabolites is reported in Supporting Table ST2). The comparison between tumor and healthy regions of the brain tissue confirmed the differential regulation of metabolites involved in a number of different metabolic pathways, and were consistent with increased biosynthetic substrate production for increased proliferation^15, 35–37^. We detected increased signals of glucose 6-phosphate/fructose 6-phosphate (structural isomers and so indistinguishable only on the basis of mass) consistent with increased glucose uptake to feed glycolysis^38, 39^. Glucose 6-phosphate can be converted into 3-phosphoglycerate, which can then be converted in two steps to glycine: both were found at higher levels in the tumor. Glycine is normally used as a substrate for nucleotide or protein metabolism and, together with the upregulation of mono-, di-, and tri-phosphate ribonucleotides (Supporting Figure SF2), indicates a possible increase in nucleotide metabolism^38^. Further support for increased biosynthetic substrate production is found with the observed increase in signals of ribose 5-phosphate in the tumor regions. Ribose 6-phosphate is also involved in the PPP pathway together with gluconate 6-phosphate (up-regulated in the tumor region). Higher concentrations of UDP glucose and UDP N-acetyl glucosamine in the tumor regions suggest upregulation of the hexosamine pathway; and the increased levels of small chain fatty acids are also consistent^15, 38, 40^.

It should be noted that one of the challenges to the analysis of metabolites in tissues is their very rapid post-mortem degradation^6, 7, 41^. The brains of the glioma model were obtained via funnel freezing, a technique recently demonstrated to be one of the more effective at maintaining metabolic integrity^10^.

### Concluding aspects

15 T MALDI-FTICR MSI was used for ultra-high mass resolution, accurate mass MSI analysis of proteins and metabolites in a murine model of glioblastoma. The ultra high mass resolution led to the detection of a much larger number of distinct protein ions compared to MALDI-TOF MSI, distinguished proteoforms and enabled the charge state and adduct type of many protein ions to be determined. Of particular note was the ability to detect proteins highly localized to tumor interface zones. MALDI-TOF MSI has previously been used to identify proteins located in the interface zone^42^, in which it was postulated that proteins localized at the interface might be more indicative of tumor growth. Here we demonstrate that the lower chemical background of MALDI-FTICR MSI is much more suited to the detection of such interface zone proteins. Note: the absence of chemical noise in Orbitrap based systems means a MALDI-Orbitrap would also be expected to be suitable for the detection of interface zone proteins by MSI.

The protein MSI datasets were complemented with microproteomics of microdissected tissue samples, obtained from consecutive tissue sections. This enabled an aspect of spatial specificity to be retained for protein assignment, thereby reducing potential false positive assignments and confirming the changes in protein expression detected by MALDI-FTICR MSI.

Different histone variants and proteoforms were detected as their singly, doubly and triply charged ions, thereby providing internal corroboration of the distributions detected by MALDI MSI.

## Methods

### Reagents

All the solvents and reagents were purchased from Sigma Aldrich (St. Louis, MO, USA). Complete mini EDTA-free Cocktail protease inhibitors were purchased from Roche. Indium tin oxide (ITO) coated glass slides were purchased from Bruker Daltonics (Bremen, Germany), polyethylene naphthalate (PEN) membrane slides (1.0 mm) were purchased from Carl Zeiss (Carl Zeiss Microsystems GmbH, Göttingen, Germany) and carboxylate modified beads, Sera-Mag 4515 and Sera-Mag 2415 SpeedBeads were purchased from Thermo Fisher Scientific.

### Sample Collection

The murine glioma GL261 cell line was grown in complete Dulbecco’s modified Eagle’s medium (DMEM) containing 10% newborn calf serum, 4.5 g/L glucose, 2 mM glutamine, 100 UI/mL penicillin and 100 mg/mL streptomycin at 37 °C in 5% CO_2_ with media changes three times per week^16^. To induce glioma formation, C57BL/6 mice (12–14 weeks old) received a stereotaxically guided injection of 40,000 GL261 cells into the visual cortex (2 mm lateral to the midline and in correspondence with lambda) using fine glass micropipettes (tip diameter 40 μm)^16, 43^.

GBM mouse brains were obtained using *in-situ* funnel-freezing^7^, 3 weeks after GL261 cell inoculation. Briefly, animals were anesthetized by intraperitoneal injection using avertin (2,2,2-tri-bromoethanol 20 µL/g of body weight). An incision was made to expose the skull and a funnel was placed onto the skull; the skin was then raised around the funnel and secured with four sutures. Liquid nitrogen was slowly poured for 3 minutes and the entire animal was then frozen in liquid nitrogen. This procedure was applied to preserve the molecular integrity of the tissues, as many biomolecules are known to undergo fast post-mortem degradation^7, 41, 44^. Extracted brains were stored at −80 °C until use.

All animal experiments conformed to the European Communities Council Directive n° 86/609/EEC and were approved by the Italian Ministry of Health.

All experiments were performed on two animals, and were performed in technical triplicate for each animal.

### Sample preparation

Coronal tissue sections of 12 µm thickness were sectioned at −17 °C using a Leica CM1950 cryostat. They were then thaw mounted onto ITO-coated slides for MSI, or onto PEN slides for LCM. Consecutive tissue sections were used for MALDI MSI and LCM.

For MALDI MSI of intact proteins the tissue sections were first washed according to the protocol of Enthaler *et al*.^45^ (96% EtOH, 70% EtOH, 10 dips H_2_O, 70% EtOH, 96% EtOH).

Matrix coating was performed with a SunCollect automated deposition system (SunChrom, Friedrichsdorf, Germany) and a solution of 5 mg/mL sinapinic acid in 50% ACN and 0.3% TFA (2 layers at 5 µL/min and 6 layers at 10 µL/min, 3 bar).

For metabolite MSI 9-aminoacridine (9-AA, 2 mg/mL 9-AA in 70% methanol) was sprayed onto the tissue section using the SunCollect (2 layers at 5 µL/min and 6 layers at 10 µL/min, 3 bar)^7^. For MALDI MSI of neurotransmitters and amino metabolites, the amine groups were first derivatized with 2,4-diphenyl-pyranylium tetrafluoroborate (DPP − TFB; 5 mg/mL in MeOH; 5 layers at 10 µL/min)^6^. After overnight incubation a uniform layer of 2,5-dihydroxy benzoic acid (DHB) matrix was deposited using the SunCollect and a solution 30 mg/mL DHB in 70% MeOH and 0.1% TFA (1 layer at 10 µL/min, 1 layer at 20 µL/min and 3 layers at 35 µL/min).

### MALDI MSI

MALDI-FTICR MSI of proteins was performed using a 15 T SolariX XR (Bruker Daltonics, Bremen, Germany). Protein MSI data was recorded in positive ion mode, using 300 laser shots per pixel and 125 µm pixel size. Ions were detected in *m/z* range 3,500–30,000, using a 512 k transient (3.4 s duration), which provided full isotopic resolution up to *m/z* 16 000.

MALDI-TOF-MSI of proteins was performed using an ultrafleXtreme MALDI-TOF/TOF (Bruker Daltonics, Bremen, Germany) in positive-ion, linear-detection mode using 125 µm pixel size and 500 laser shots per pixel. Ions were detected in *m/z* range 3,500–30,000.

MALDI-FTICR MSI of metabolites was performed using a 9.4 T SolariX XR (Bruker Daltonics, Bremen, Germany). Metabolite MSI data were recorded in positive ion mode (neurotransmitters and amino metabolites, DHB matrix, *m/z* 50–500) and negative ion mode (adenylates and TCA cycle intermediate, 9-AA matrix, *m/z* range 50–1000), using 500 laser shots per pixel, 125 µm pixel sizes and a 512 k transient (0.2 s duration).

Data acquisition, processing, and data visualization were performed using the Flex software suite (FlexControl 3.4, ftmsControl 2.0, FlexImaging 4.1 and DataAnalysis 4.2) from Bruker Daltonics. MSI data were acquired from each tissue section as well as matrix control areas adjacent to the tissue sections to check for analyte dispersion during sample preparation.

After MSI data acquisition, any residual matrix was removed with a 70% ethanol wash and the tissue samples then stained with cresyl violet solution (Nissl stain). High-resolution histological images were then recorded using a digital slide scanner (3D Histech MIDI).

### Laser Capture Microdissection and Protein Digestion

LCM was performed using a PALM Technologies system (Carl Zeiss MicroImaging GmbH, Munchen, Germany) consisting of a PALM MicroBeam, a RoboStage and PALM RoboMover (PALM Robo software, version 4.6 Pro). LCM was performed using an X40 ocular lens at UV laser energy of 44 and UV laser focus of 56. Small regions of tumor and healthy tissue, each of approximately 0.8 mm^2^, were isolated and collected into LoBind™ Eppendorf tubes (Eppendorf AG, Hamburg, Germany) and then stored at −80 °C until use.

Tissue lysis, protein extraction, proteolytic digestion and peptide purification was performed using the ultrasensitive SP3 method (single-pot solid-phase-enhanced sample preparation) based on paramagnetic beads^46, 47^. First, tissue/cells lysis was performed using a lysis buffer (LB) at pH 8.5, composed of 50 mM HEPES, 1% SDS and protease inhibitor (Complete mini EDTA-free Cocktail, 1 pill in 10 mL of buffer). An equal volume of trifluoroethanol (TFE) was then added, followed by 20 mg/mL of beads (50/50 Sera-Mag 4515/SeraMag 2415 SpeedBeads). The sample was then sonicated with a Bioruptor Pico (Diagenode, Seraing, Belgium), using 10 cycles each of 30 s duration. Reduction (DTT 200 mM), alkylation (IAA 400 mM) and overnight trypsin digestion (1:25 enzyme/protein) steps were applied on the same tube. The purified peptides were eluted from the beads with a 2% DMSO aqueous solution.

### LC-MS/MS Analysis and Data Processing

LC-MS/MS experiments were performed using an Easy-nLC 1000 coupled to an Orbitrap Fusion mass spectrometer (both Thermo Fisher Scientific, Bremen, Germany). Tryptic peptides were resuspended in 10% aqueous formic acid and injected into an EASY-spray C18 column (2 µm particle size, 75 µm × 50 cm) equipped with a trap column (2 µm particle size, 100 µm × 2 cm). Sample loading was perfomed at 800 bar with 100% buffer A (aqueous 0.1% formic acid) and eluted using a segmented gradient: 6% buffer B (acetonitrile 0.1% formic acid) for 1 minute, 6–23% B in 52 minutes, 23–33% B in 7 minutes, 33–90% B in 6 minutes, 90% B for 9 minutes.

Data dependent LC-MS/MS was performed in top speed mode using a 2 second maximum cycle time. MS scans were acquired in the Orbitrap, *m/z* 375 to 1500, at 120 k resolution with an AGC target of 5e5 and 100 ms maximum injection time. Monoisotopic precursor selection and a dynamic exclusion of 20 s were adopted. Ions with charge states 2+ to 8+ and intensity greater than 5e3 were selected for HCD fragmentation (32 NCE). MS^2^ spectra were recorded in the linear ion trap with a rapid scan rate, 2e3 AGC target and 300 ms maximum injection time. The acquisition was performed in profile mode for the MS scans and in centroid mode for MS/MS scans.

Proteome Discoverer 2.1 was used for protein identification using the SequestHT search engine with the following settings: Uniprot *Mus musculus* fasta database (2016-07); dynamic modifications: methionine oxidation; fixed modification: cysteine carbamidomethylation; maximum number of missed cleavages: 2; Precursor Mass Tolerance: 20 ppm; Fragment Mass Tolerance: 0.6 Da.

### Alignment of MALDI MSI and LC-MS/MS dataset and proteins identification

Average mass spectra were extracted from the tumor and healthy regions of the FTICR MSI datasets. The accurate mass measurements and isotopic distributions of the protein ions detected by FTICR MSI were compared with the theoretical mass and isotopic distributions of the proteins identified by LC-MS/MS. All protein ions were assigned on the basis of high mass accuracy, <10 ppm, and a high correlation between the experimental and theoretical isotope distributions (performed in Matlab, Pearson correlation, r ≥ 0.95). In order to retain spatial specificity the MSI data from the tumor (healthy) region were compared only with the proteins identified from the microdissected tumor (healthy) region. Database of proteins ions commonly detected by MALDI MSI were also considered^2, 48^. Possible mass shifts due to PTMs (mono-, di-, tri- methylation, acetylation of histones), chemical modifications (single and double oxidation, disulfide bonds and deamidation), as well as adducts (Na+, K+, matrix adducts) were also included.

## Electronic supplementary material

Supplementary Information

Supplementary Protein List PL1

Supplementary Protein List PL2
