# Supplementary material for: Ultra-High Mass Resolution MALDI Imaging Mass Spectrometry of Proteins and Metabolites in a Mouse Model of Glioblastoma
Source: Sci Rep. 2017 Apr 4;7:603. doi: 10.1038/s41598-017-00703-w (PMC5429601; doi:10.1038/s41598-017-00703-w)
Supplement: Supplementary file 1 — Supplementary Information [file 41598_2017_703_MOESM1_ESM.pdf]

Supplementary info

Ultra-High Mass Resolution MALDI Imaging Mass Spectrometry of Proteins and Metabolites in a Mouse Model of Glioblastoma

Dilillo M. [1, 2], Ait-Belkacem R. [1], Esteve C. [3], Pellegrini D. [4,1], Nicolardi S. [3], Costa M.[5], Vannini E.[5], de Graaf E. L.[1], Caleo M. [5], McDonnell L. A. [1,3,6] *

[1] Fondazione Pisana per la Scienza ONLUS – Via Panfilo Castaldi 2, 56121 – Pisa

[2] Department of Chemistry and Industrial Chemistry – Università di Pisa – Via Giuseppe Moruzzi 13, 56124 – Pisa

[3]Center for Proteomics and Metabolomics, Leiden University Medical Center, Leiden, The Netherlands

[4] NEST, Istituto Nanoscienze-National Research Council, 56127 Pisa, Italy

[5] CNR Neuroscience Institute, Via Moruzzi 1, 56124 Pisa, Italy

[6] Department of Pathology, Leiden University Medical Center, Leiden, The Netherlands

* Correspondence to l.a.mcdonnell@outlook.com

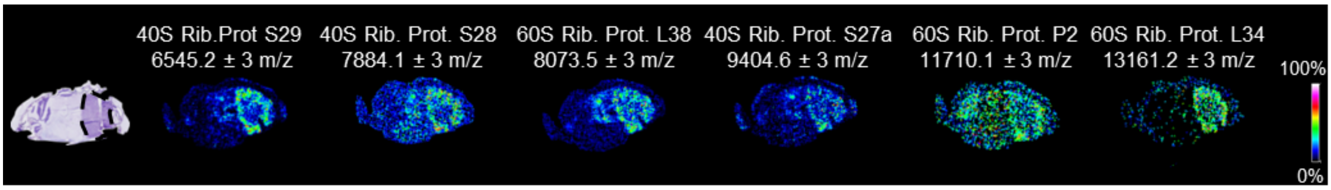

Supplementary Figure SF1 MALDI FTICR images of ribosomal proteins highlighted in the text

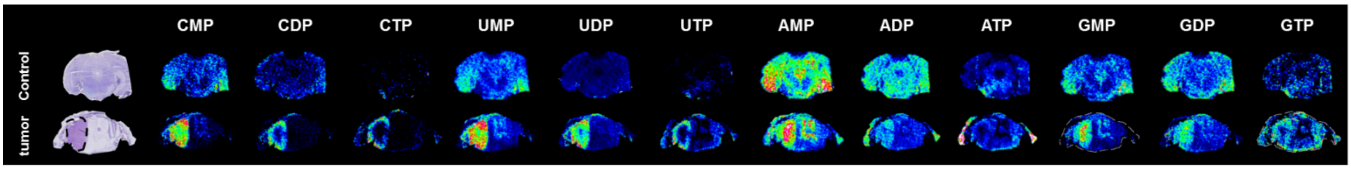

Supplementary Figure SF2 MALDI FTICR images of mono-, di-, and triphosphate ribonucleotides

Supplementary Table ST1: Protein identified in the MALDI - FTICR average spectra with alignment with the data obtained with the LCM-LC-MS/MS results and available mass lists [1, 2]. Me: methylation; Ac: acetylation; S-S: disulphide bond; deam: deamidation; heme: heme group; Ox: oxidation; SA: Sinapinic acid adduct;

Prot. Entry	Protein name	Sequence	PTMs and Chemical mod.	Average Mass detected
F8WIX8	Histone H2A	87-121		3805.18
Q7M088	Beta-amyloid protein	1-37		4075.30
Q7M088	Beta-amyloid protein	1-37	Me	4089.24
Q7M088	Beta-amyloid protein	1-38	2Me	4160.27
Q7M088	Beta-amyloid protein	1-39		4231.21
Q7M088	Beta-amyloid protein	1-39	Ac	4274.12
Q6ZWY8	Thymosin β 10	2-44	Ac	4937.30
P20065	Thymosin β 4	8-50	Ac	4964.25
P20065	Thymosin β 4	8-50	Ac / Ox	4980.24
P17665	Cytochrome C oxidase polypeptide VIIC	17-63		5444.60
P62274	40s ribosomal protein 29	2-56		6546.52
P48771	Cytocrome C oxidase polypeptide VIIa	24-83		6573.66
P48771	Cytocrome C oxidase polypeptide VIIa	24-83	2Ox	6605.62
P48771	Cytocrome C oxidase polypeptide VIIa	24-83	Ac / 2Ox	6648.02
P56394	Cytochrome C oxidase copper chaperone	2-63	Ox	6669.99
P48771	Cytocrome C oxidase polypeptide VIIa	24-83	Ac / 2Ox /SA	6872.06
P62858	40S ribosomal protein S28	1-69	2deam	7844.21
P62858	40s ribosomal protein S28	Full	Ac	7883.14
Q9JJI8	60s ribosomal protein L38	2-70		8073.60
Q06185	ATP synthase subunit e, mitochondrial	2-71		8106.11
P62983	Ubiquitin - 40S ribosomal protein S27a	1- 76	Ubiquitin	8565.41
D6RH49	40S ribosomal protein S27	Full	Ac S-S	8660.17
P62983	Ubiquitin - 40S ribosomal protein S27a	1-76	Ubiquitin / SA	8790.32
P97450	ATP synthase Coupling Factor 6	33-108		8946.23
Q9D8X0	Protein MANBAL	Full	2Me	9369.45
P62983	Ubiquitin - 40S ribosomal protein S27a	77-156	40S rib.p.S27a	9405.61
P14069	Calcyclin	2-89	Ac	9962.35
P14069	Calcyclin	2-89	Ac / Ox	9978.44
P56391	Cytochrome C oxydase subunit 6B	2-86	2Me / Ac	10011.35
P27005	S100-A8 (Calgranulin A)	2-93		10164.58
P14069	Calcyclin	2-89	Ac / Ox / SA	10202.55
P14069	Calcyclin	2-89	Ac / Ox / 2SA	10426.56
Q62426	Cystatin B	Full	Ac	11088.86
P62806	Histone H4	2-103	Ac / 2Me	11306.52
P62806	Histone H4	2-103	Ac / 3Me	11321.52
P62806	Histone H4	2-103	Ac / 4Me	11334.51
P62806	Histone H4	2-103	Ac / 5Me	11348.50
P62806	Histone H4	2-103	Ac / 6Me	11363.50
P62806	Histone H4	2-103	Ac / 7Me	11376.49

P62806	Histone H4	2-103	3Ac / 2Me	11390.98
P62806	Histone H4	2-103	2Ac / 5Me	11390.98
P62806	Histone H4	2-103	Ac / 2Me / SA	11530.42
P62806	Histone H4	2-103	Ac / 3Me / SA	11545.87
P62806	Histone H4	2-103	Ac / 5Me / SA	11573.96
P17095	High mobility group protein HMG-I/HMG-Y	1-107	2Me	11643.32
P99027	60S acidic ribosomal protein P2	1-115	Ac / OX	11710.11
P62806	Histone H4	2-103	Ac / 2Me / 2SA	11756.00
D3YWS3	Profilin	1-110		11778.46
P01837	Ig kappa chain C region	1-106		11778.37
P62897	Cytochromec, somatic	2-104	Heme / Ac	12132.63
P62897	Cytochromec, somatic	2-104	Heme / Ac / ox	12148.61
P62897	Cytochromec, somatic	2-104	Heme / Ac / SA	12357.47
P19788	Matrix Gla protein	Full	K+	12398.45
P61804	Dolichyl-diphosphooligosaccharide--protein glycosyltransferase subunit DAD-1	Full	Ox	12513.44
P62897	Cytochromec, somatic	2-104	Heme / Ac / 2SA	12581.39
Q9D1R9	60S ribosomal protein L34	2-117		13163.13
P0C0S6	Histone Ha.z	2-128		13421.33
Q9CQB4	Cytochrome b-c1 complex subunit 7	Full		13562.32
P62852	40S ribosomal protein S25	Full	Ox	13758.38
Q6ZWY9	Histone H2B type 1-C/E/g	2-126		13775.39
Q64478	Histone H2B type 1-H	2-126		13789.65
Q8CGP1	Histone H2B type 1-K	2-126		13789.65
Q6ZWY9	Histone H2B type 1-C/E/g	2-126	Me	13789.65
P10853	Histone H2B type 1-F/J/L	2-126		13805.63
P10853	Histone H2B type 1-F/J/L	2-126	Me	13819.72
Q64478	Histone H2B type 1-H	2-126	K+	13826.59
P10853	Histone H2B type 1-F/J/L	2-126	2Me	13833.59
P10853	Histone H2B type 1-F/J/L	2-126	3Me	13847.61
Q64524	Histone H2B type 2-E	2-126		13863.59
Q6GSS7	Histone H2A type 2-A	2-131	Ac	14007.98
Q6GSS7	Histone H2A type 2-A	2-131	Me / Ac	14021.36
A2AB79	Histone H2A	2-129	Ac	14033.21
P22752	Histone H2A type 1	2-130	Ac	14048.03
P22752	Histone H2A type 1	2-130	Ac / Me	14062.37
Q6GSS7	Histone H2A type 2-A	2-131	2Ac / 2Me	14078.38
Q6GSS7	Histone H2A type 2-A	2-131	2Ac / 3Me	14093.39
A2AB79	Histone H2A	2-129	Ac / 5Me	14106.39
Q6GSS7	Histone H2A type 2-A	Full	Ac	14137.40455
Q6GSS7	Histone H2A type 2-A	Full	Ac / Me	14152.41
Q6GSS7	Histone H2A type 2-A	Full	Ac / SA	14362.46
Q6GSS7	Histone H2A type 2-A	Full	Ac / Me / SA	14376.43
P01942	Hemoglobin subunit beta	2-142	2Me	14982.66
P84244	Histone H3.3	2-136	Ac	15240.21
P84244	Histone H3.3	2-136	Ac / Me	15253.24
P84244	Histone H3.3	2-136	Ac / 2Me	15267.42
P84244	Histone H3.3	2-136	Ac / 3Me	15281.45

P84228	Histone H3.2	2-136	Ac	15297.45
P68433	Histone H3.1	2-135	Ac / Me	15327.25
P84228	Histone H3.2	2-136	Ac / 3Me	15341.45
P84228	Histone H3.2	2-136	Ac / 4Me	15355.42
P84228	Histone H3.2	2-136	Ac / 5Me	15369.40
P84228	Histone H3.2	2-136	3AC	15838.42
P84228	Histone H3.2	2-136	Ac / 6Me	15383.42
P84228	Histone H3.2	2-136	Ac / 7Me	15397.38

1. McDonnell, L. a., Walch, A., Stoeckli, M. & Corthals, G. L. MSiMass list: A public database of identifications for protein MALDI MS imaging. *J. Proteome Res.* **13,** 1138–1142 (2014).
2. Maier, S. K. *et al.* Comprehensive identification of proteins from MALDI imaging. *Mol. Cell. Proteomics* **12,** 2901–10 (2013).

Supplementary Table ST2: List of metabolites detected with MALDI-FTICR-MSI with two experimental approaches. Second column: list of masses detected in negative ionization with 9-AA as matrix. Third column: list of detected m/z values identified as derivatized metabolites, derivatization performed with DPP-TFB and detection in positive mode .

Meatbolite	Mass detected with 9-AA	Mass detected with DPP-TFB derivatization [M+ 233.0961]+
Hydroxyproline	130.05	
Glutamihne	145.061	
Glutamate	146.045	
Uric Acid	167.02	
Glycerol monophosphate	171.005	
N - acetylaspartate	174.04	
Ascorbate	175.024	
Citric Acid	191.019	
Phosphocreatinine	210.027	
Ribose - phosphate	229.011	
Inositol cyclic phosphate	241.011	
Palmitic acid	255.232	
Glucose/Fructose - monophosphate	259.022	
6 – phosphogluconic acide	275.017	
Linoleic acid	279.232	
Oleic acid	281.248	
NAAG	303.083	
Arachidonic Acid	303.232	
Glutathione	306.076	
CMP	322.044	
UMP	323.028	
Docosahexaenoic Acid	327.232	
AMP	346.055	
IMP	347.039	
GMP	362.05	
CDP	402.01	
UDP	402.994	
ADP	426.022	
GDP	442.016	
CTP	481.977	
UTP	482.961	

ATP	505.988	
GTP	521.983	
Udp - glucose	565.047	
UDP-N acetylgucosamine	606.074	
Phosphadidylinositol (18:1)	883.534	
Phosphadidylinositol (18:0)	885.549	
Glycine		290.118
Alanine		304.133
GABA		318.149
Proline		330.149
Valine		332.165
Threonine		334.144
Taurine		340.1
Leucine		346.18
Aspartate		348.123
Tyramine		352.169
Glutamine		361.155
Lysine		361.191
Glutamate		362.139
Dopamine		368.164
Tryptamine		375.186
3-MT		382.18
Tyrosine		396.159
L-DOPA		412.154
