# Supplementary material for: Ultra-High Mass Resolution MALDI Imaging Mass Spectrometry of Proteins and Metabolites in a Mouse Model of Glioblastoma
Source: Sci Rep. 2017 Apr 4;7:603. doi: 10.1038/s41598-017-00703-w (PMC5429601; doi:10.1038/s41598-017-00703-w)
Supplement: Supplementary file 2 — Supplementary Protein List PL1 [file 41598_2017_703_MOESM2_ESM.pdf]

Accession	Description	Contaminant	Coverage	# Peptides	# PSMs	# Unique Peptides	MW [kDa]	calc. pI	Area: F3: Sample	Area: F2: Sample	Area: F1: Sample	Score Sequest HT
P56382	ATP synthase subunit epsilon, mitochondrial OS=Mus musculus GN=Atp5e PE=1 SV=2		15.384615	1	1	1	5.834	10.01		1700000		2.10
P62892	60S ribosomal protein L39 OS=Mus musculus GN=Rpl39 PE=1 SV=2		19.607843	1	1	1	6.403	12.56	970000			2.57
P0DN34	NADH dehydrogenase [ubiquinone] 1 beta subcomplex subunit 1 OS=Mus musculus GN=		19.298246	1	2	1	6.95	8.21	2100000		2900000	5.45
P17665	Cytochrome c oxidase subunit 7C, mitochondrial OS=Mus musculus GN=Cox7c PE=1 SV		14.285714	1	1	1	7.328	11	4500000			1.96
Q8R1I1	Cytochrome b-c1 complex subunit 9 OS=Mus musculus GN=Uqcr10 PE=1 SV=1		37.5	2	7	2	7.441	9.19	58000000	31000000	26000000	26.92
P60761	Neurogranin OS=Mus musculus GN=Nrgn PE=1 SV=1		17.948718	1	2	1	7.492	7.05	54000		3500000	5.29
P62858	40S ribosomal protein S28 OS=Mus musculus GN=Rps28 PE=1 SV=1		17.391304	1	2	1	7.836	10.7	1400000		1500000	5.48
P63213	Guanine nucleotide-binding protein G(I)/G(S)/G(O) subunit gamma-2 OS=Mus musculus (		25.352113	2	2	2	7.845	7.99			11000000	6.68
Q9JMF3	Guanine nucleotide-binding protein G(I)/G(S)/G(O) subunit gamma-13 OS=Mus musculus		11.940299	1	2	1	7.974	5.45	520000	540000		4.95
Q9DAS9	Guanine nucleotide-binding protein G(I)/G(S)/G(O) subunit gamma-12 OS=Mus musculus		15.277778	1	1	1	7.992	8.97			630000	2.47
Q9JJI8	60S ribosomal protein L38 OS=Mus musculus GN=Rpl38 PE=1 SV=3		14.285714	1	1	1	8.199	10.1				2.04
Q06185	ATP synthase subunit e, mitochondrial OS=Mus musculus GN=Atp5i PE=1 SV=2		16.901408	1	2	1	8.23	9.35	23000000		27000000	6.19
P63216	Guanine nucleotide-binding protein G(I)/G(S)/G(O) subunit gamma-3 OS=Mus musculus (		20	1	6	1	8.299	7.78	6100000	1300000	6100000	18.51
Q3UZP4	Small VCP/p97-interacting protein OS=Mus musculus GN=Svip PE=3 SV=1		32.467532	2	4	2	8.358	8.91	2100000	640000	990000	10.62
P50153	Guanine nucleotide-binding protein G(I)/G(S)/G(O) subunit gamma-4 OS=Mus musculus (		21.333333	1	2	1	8.399	7.08	2600000		1700000	6.49
Q9CPQ1	Cytochrome c oxidase subunit 6C OS=Mus musculus GN=Cox6c PE=1 SV=3		28.947368	3	9	3	8.464	10.14	30000000	13000000	26000000	19.62
P59648	FXYD domain-containing ion transport regulator 7 OS=Mus musculus GN=Fxyd7 PE=3 S\		16.25	1	2	1	8.481	7.84		24000000	30000000	5.12
P62309	Small nuclear ribonucleoprotein G OS=Mus musculus GN=Snrpg PE=1 SV=1		15.789474	1	1	1	8.49	8.88			720000	2.96
Q9CQZ1	Heat shock factor-binding protein 1 OS=Mus musculus GN=Hsbp1 PE=1 SV=1		39.473684	2	8	2	8.605	4.31	2500000	1400000	3800000	27.67
P29595	NEDD8 OS=Mus musculus GN=Nedd8 PE=1 SV=2		13.580247	1	3	1	8.967	7.25	5400000	1600000	4100000	8.71
P48771	Cytochrome c oxidase subunit 7A2, mitochondrial OS=Mus musculus GN=Cox7a2 PE=1 S		27.710843	2	3	2	9.285	10.27		1900000	4700000	7.59
Q62425	Cytochrome c oxidase subunit NDUFA4 OS=Mus musculus GN=Ndufa4 PE=1 SV=2		36.585366	3	8	3	9.321	9.52	11000000	14000000	17000000	23.27
Q6ZWU9	40S ribosomal protein S27 OS=Mus musculus GN=Rps27 PE=1 SV=3		13.095238	1	2	1	9.455	9.45	5600000			3.01
Q9CQ69	Cytochrome b-c1 complex subunit 8 OS=Mus musculus GN=Uqcrq PE=1 SV=3		19.512195	2	9	2	9.762	10.26	7100000	8500000	5400000	20.68
Q9CQS8	Protein transport protein Sec61 subunit beta OS=Mus musculus GN=Sec61b PE=1 SV=3		10.416667	1	3	1	9.952	11.56	6000000	1600000	2200000	6.92
P56391	Cytochrome c oxidase subunit 6B1 OS=Mus musculus GN=Cox6b1 PE=1 SV=2		12.790698	1	1	1	10.065	8.72		1100000		2.31
P56135	ATP synthase subunit f, mitochondrial OS=Mus musculus GN=Atp5j2 PE=1 SV=3		26.136364	2	6	2	10.337	9.95	17000000	9600000	16000000	12.72
Q9D0M5	Dynein light chain 2, cytoplasmic OS=Mus musculus GN=Dynll2 PE=1 SV=1		37.078652	2	6	2	10.343	7.37	2300000	2100000	4200000	12.53
Q9D164	FXYD domain-containing ion transport regulator 6 OS=Mus musculus GN=Fxyd6 PE=1 S\		35.106383	2	7	2	10.367	5.11	16000000	7500000	17000000	32.49
P99028	Cytochrome b-c1 complex subunit 6, mitochondrial OS=Mus musculus GN=Uqcrh PE=1 S		37.078652	2	5	2	10.428	4.87	5000000	5600000	6700000	15.39
P56565	Protein S100-A1 OS=Mus musculus GN=S100a1 PE=1 SV=2		13.829787	1	1	1	10.498	4.5		3700000		2.77
P50114	Protein S100-B OS=Mus musculus GN=S100b PE=1 SV=2		16.304348	1	1	1	10.721	4.55		2200000		3.88
O70554	Small proline-rich protein 2B OS=Mus musculus GN=Sprr2b PE=2 SV=1		9.1836735	1	2	1	10.727	7.46			30000000	4.52
P61957	Small ubiquitin-related modifier 2 OS=Mus musculus GN=Sumo2 PE=1 SV=1		23.157895	2	3	2	10.864	5.5	7600000		7400000	8.87
Q9CQ75	NADH dehydrogenase [ubiquinone] 1 alpha subcomplex subunit 2 OS=Mus musculus GN		14.141414	1	3	1	10.909	9.99	3900000	1500000	2500000	8.69
Q3LHH8	Exocrine gland-secreted peptide 1 OS=Mus musculus GN=Esp1 PE=1 SV=1		9.8039216	1	1	1	11.339	8.91		23000000		1.83
P62806	Histone H4 OS=Mus musculus GN=Hist1h4a PE=1 SV=2		56.31068	9	60	9	11.36	11.36	390000000	180000000	220000000	164.97
Q9CPQ8	ATP synthase subunit g, mitochondrial OS=Mus musculus GN=Atp5l PE=1 SV=1		19.417476	2	7	2	11.417	9.74	12000000	2900000	5400000	17.70
Q9D0J8	Parathymosin OS=Mus musculus GN=Ptms PE=1 SV=3		10.891089	1	2	1	11.423	4.22	5100000	3600000		4.88
P47955	60S acidic ribosomal protein P1 OS=Mus musculus GN=Rplp1 PE=1 SV=1		14.035088	1	1	1	11.468	4.32			670000	2.86
P63024	Vesicle-associated membrane protein 3 OS=Mus musculus GN=Vamp3 PE=1 SV=1		38.834951	4	18	1	11.473	8.5	480000			67.68
P62897	Cytochrome c, somatic OS=Mus musculus GN=Cycs PE=1 SV=2		20.952381	2	6	2	11.598	9.58	3500000	2400000	1500000	18.01
P17095	High mobility group protein HMG-I/HMG-Y OS=Mus musculus GN=Hmga1 PE=1 SV=4		8.411215	1	3	1	11.607	10.32				7.08
P99027	60S acidic ribosomal protein P2 OS=Mus musculus GN=Rplp2 PE=1 SV=3		60	5	17	5	11.644	4.54	36000000	17000000	36000000	59.14
P10639	Thioredoxin OS=Mus musculus GN=Txn PE=1 SV=3		20.952381	2	4	2	11.668	4.92		3700000	5500000	12.54

Q9CQZ6	NADH dehydrogenase [ubiquinone] 1 beta subcomplex subunit 3 OS=Mus musculus GN=	17.307692	2	6	2	11.685	9.04	42000000	14000000	26000000	15.05
P01837	Ig kappa chain C region OS=Mus musculus PE=1 SV=1	28.301887	2	4	2	11.771	5.41	1900000	840000	1200000	12.74
Q9CPS8	Small membrane A-kinase anchor protein OS=Mus musculus PE=1 SV=1	12.264151	1	2	1	11.919	4.25	2200000		2600000	5.17
Q91WS0	CDGSH iron-sulfur domain-containing protein 1 OS=Mus musculus GN=Cisd1 PE=1 SV=	35.185185	3	15	3	12.089	9.06	17000000	9300000	15000000	53.88
P26350	Prothymosin alpha OS=Mus musculus GN=Ptma PE=1 SV=2	12.612613	2	4	2	12.247	3.79	1100000	510000	1600000	13.21
P62878	E3 ubiquitin-protein ligase RBX1 OS=Mus musculus GN=Rbx1 PE=1 SV=1	7.4074074	1	2	1	12.266	6.96	3200000		2900000	4.75
P43024	Cytochrome c oxidase subunit 6A1, mitochondrial OS=Mus musculus GN=Cox6a1 PE=1 S	14.414414	1	2	1	12.344	9.98		2200000	1700000	2.96
P83882	60S ribosomal protein L36a OS=Mus musculus GN=Rpl36a PE=1 SV=2	8.490566	1	2	1	12.433	10.58		710000	1300000	4.32
P63030	Mitochondrial pyruvate carrier 1 OS=Mus musculus GN=Mpc1 PE=1 SV=1	7.3394495	1	2	1	12.446	9.61	12000000		10000000	4.82
P83940	Transcription elongation factor B polypeptide 1 OS=Mus musculus GN=Tceb1 PE=1 SV=1	12.5	1	1	1	12.465	4.78		510000		2.30
P97450	ATP synthase-coupling factor 6, mitochondrial OS=Mus musculus GN=Atp5j PE=1 SV=1	17.592593	1	1	1	12.489	9.36		2500000		3.48
O55142	60S ribosomal protein L35a OS=Mus musculus GN=Rpl35a PE=1 SV=2	14.545455	2	4	2	12.546	10.89	13000000	6800000	7300000	8.63
Q99LY9	NADH dehydrogenase [ubiquinone] iron-sulfur protein 5 OS=Mus musculus GN=Ndufs5 F	11.320755	1	3	1	12.639	8.92	4900000	2700000	6900000	10.24
P63044	Vesicle-associated membrane protein 2 OS=Mus musculus GN=Vamp2 PE=1 SV=2	35.344828	5	34	2	12.683	8.13	28000000	11000000	41000000	139.78
P62889	60S ribosomal protein L30 OS=Mus musculus GN=Rpl30 PE=1 SV=2	23.478261	2	5	2	12.776	9.63	4100000	4000000	4000000	14.99
Q62442	Vesicle-associated membrane protein 1 OS=Mus musculus GN=Vamp1 PE=1 SV=1	33.898305	3	11	2	12.882	6.65	3500000	1400000	3600000	39.22
P62855	40S ribosomal protein S26 OS=Mus musculus GN=Rps26 PE=1 SV=3	20.869565	2	6	2	13.007	11	9100000	4300000	7400000	18.50
Q9D882	Uncharacterized protein C10orf35 homolog OS=Mus musculus PE=1 SV=1	9.1666667	1	1	1	13.11	11.41			1100000	2.16
P62869	Transcription elongation factor B polypeptide 2 OS=Mus musculus GN=Tceb2 PE=1 SV=1	7.6271186	1	3	1	13.162	5.01		1700000	1700000	6.85
P03899	NADH-ubiquinone oxidoreductase chain 3 OS=Mus musculus GN=Mtnd3 PE=1 SV=3	13.043478	1	4	1	13.21	4.64	4100000	2700000	3400000	9.88
Q9D1R9	60S ribosomal protein L34 OS=Mus musculus GN=Rpl34 PE=1 SV=2	14.529915	2	4	2	13.284	11.47	12000000	6000000		8.33
Q9CPP6	NADH dehydrogenase [ubiquinone] 1 alpha subcomplex subunit 5 OS=Mus musculus GN	22.413793	3	7	3	13.351	8.1	13000000	7300000	11000000	22.40
Q9D1K2	V-type proton ATPase subunit F OS=Mus musculus GN=Atp6v1f PE=1 SV=2	17.647059	2	6	2	13.362	5.82	8400000	5700000	3100000	15.20
P60867	40S ribosomal protein S20 OS=Mus musculus GN=Rps20 PE=1 SV=1	19.327731	2	6	2	13.364	9.94	11000000	6900000	11000000	16.35
Q8R404	MICOS complex subunit MIC13 OS=Mus musculus GN=Mic13 PE=1 SV=1	5.8823529	1	3	1	13.365	8.63	9000000	6500000	7100000	6.56
P62317	Small nuclear ribonucleoprotein Sm D2 OS=Mus musculus GN=Snrpd2 PE=1 SV=1	39.830508	4	7	4	13.518	9.91	4300000	2200000	3700000	17.78
Q9D855	Cytochrome b-c1 complex subunit 7 OS=Mus musculus GN=Uqcrb PE=1 SV=3	24.324324	3	11	3	13.519	9.11	14000000	5300000	7700000	27.74
P0C0S6	Histone H2A.Z OS=Mus musculus GN=H2afz PE=1 SV=2	20.3125	3	10	1	13.545	10.58	46000000	21000000	30000000	29.28
O55186	CD59A glycoprotein OS=Mus musculus GN=Cd59a PE=2 SV=1	13.821138	1	1	1	13.639	7.47			2200000	2.52
Q9WTT4	V-type proton ATPase subunit G 2 OS=Mus musculus GN=Atp6v1g2 PE=1 SV=1	36.440678	4	16	4	13.643	10.26	2400000	3700000	2400000	54.50
Q9CR51	V-type proton ATPase subunit G 1 OS=Mus musculus GN=Atp6v1g1 PE=1 SV=3	23.728814	2	4	2	13.716	7.97	2600000	6000000	6700000	13.35
P62852	40S ribosomal protein S25 OS=Mus musculus GN=Rps25 PE=1 SV=1	22.4	3	9	3	13.734	10.11	3700000	2900000	2800000	20.94
P70349	Histidine triad nucleotide-binding protein 1 OS=Mus musculus GN=Hint1 PE=1 SV=3	11.111111	1	1	1	13.768	6.87	3200000			2.68
Q64253	Lymphocyte antigen 6E OS=Mus musculus GN=Ly6e PE=1 SV=2	10.769231	1	3	1	13.791	7.05	1200000	1500000	1700000	9.49
P19536	Cytochrome c oxidase subunit 5B, mitochondrial OS=Mus musculus GN=Cox5b PE=1 SV	15.625	2	5	2	13.804	8.38	5800000	9000000	11000000	13.30
P10853	Histone H2B type 1-F/J/L OS=Mus musculus GN=Hist1h2bf PE=1 SV=2	41.269841	7	40	4	13.928	10.32	8600000	83000000	100000000	137.85
Q64524	Histone H2B type 2-E OS=Mus musculus GN=Hist2h2be PE=1 SV=3	34.920635	4	31	1	13.985	10.32		17000000	20000000	105.55
Q91ZZ3	Beta-synuclein OS=Mus musculus GN=Sncb PE=1 SV=1	17.293233	2	6	1	14.043	4.37	12000000	14000000	9600000	18.89
P22752	Histone H2A type 1 OS=Mus musculus GN=Hist1h2ab PE=1 SV=3	54.615385	5	22	3	14.127	11.05	37000000	33000000	16000000	66.60
Q9CQ54	NADH dehydrogenase [ubiquinone] 1 subunit C2 OS=Mus musculus GN=Ndufc2 PE=1 S'	31.666667	4	5	4	14.154	9.2	6500000		770000	11.08
Q9D0T1	NHP2-like protein 1 OS=Mus musculus GN=Snu13 PE=1 SV=4	9.375	1	1	1	14.165	8.46			1600000	2.77
Q91VR7	Microtubule-associated proteins 1A/1B light chain 3A OS=Mus musculus GN=Map1lc3a P	23.140496	3	7	3	14.263	8.68	6800000	5000000	8700000	15.61
Q9D023	Mitochondrial pyruvate carrier 2 OS=Mus musculus GN=Mpc2 PE=1 SV=1	5.511811	1	3	1	14.277	10.61	4400000	1600000	3500000	6.35
P11031	Activated RNA polymerase II transcriptional coactivator p15 OS=Mus musculus GN=Sub1	20.472441	3	6	3	14.418	9.6	23000000	9300000	22000000	16.41
P62900	60S ribosomal protein L31 OS=Mus musculus GN=Rpl31 PE=1 SV=1	13.6	2	5	2	14.454	10.54	17000000	9300000	15000000	11.90
O55042	Alpha-synuclein OS=Mus musculus GN=Snca PE=1 SV=2	41.428571	4	13	3	14.476	4.77	2000000	2600000	2900000	42.91
Q6ZWV7	60S ribosomal protein L35 OS=Mus musculus GN=Rpl35 PE=1 SV=1	8.1300813	1	3	1	14.544	11.05	15000000	9800000	9600000	8.87
Q9WUC3	Lymphocyte antigen 6H OS=Mus musculus GN=Ly6h PE=1 SV=2	22.302158	3	12	3	14.659	7.53	50000000	40000000	49000000	34.50

P67984	60S ribosomal protein L22 OS=Mus musculus GN=Rpl22 PE=1 SV=2		18.75	2	4	2	14.75	9.19		7200000	11000000	11.11
P62245	40S ribosomal protein S15a OS=Mus musculus GN=Rps15a PE=1 SV=2		10.769231	1	3	1	14.83	10.13	18000000	6200000	9400000	12.42
P62830	60S ribosomal protein L23 OS=Mus musculus GN=Rpl23 PE=1 SV=1		12.857143	2	4	2	14.856	10.51	6900000	5400000	7100000	8.97
Q9D8B4	NADH dehydrogenase [ubiquinone] 1 alpha subcomplex subunit 11 OS=Mus musculus G		36.170213	4	8	4	14.972	8.35	11000000	3200000	6700000	23.53
Q9JKC6	Cell cycle exit and neuronal differentiation protein 1 OS=Mus musculus GN=Cend1 PE=1		19.463087	2	18	2	14.978	8.97	16000000	8100000	16000000	59.25
Q0VBF8	Protein stum homolog OS=Mus musculus PE=1 SV=1		17.730496	2	4	2	14.995	7.21	3100000	2300000	1800000	13.46
Q9JJV2	Profilin-2 OS=Mus musculus GN=Pfn2 PE=1 SV=3		30.714286	3	12	3	15.022	6.99	9700000	7600000	9800000	33.58
P02584	SWISS-PROT:P02584 (Bos taurus) Profilin-1	x	8.5714286	1	1	1	15.048	8.28	1900000			2.29
P01942	Hemoglobin subunit alpha OS=Mus musculus GN=Hba PE=1 SV=2		35.211268	4	12	4	15.076	8.22	28000000	3600000	24000000	37.95
P63040	Complexin-1 OS=Mus musculus GN=Cplx1 PE=1 SV=1		14.925373	1	4	1	15.112	4.97	3400000	1800000	3900000	17.22
P56395	Cytochrome b5 OS=Mus musculus GN=Cyb5a PE=1 SV=2		25.373134	2	4	2	15.232	5.07	4800000		6100000	13.17
Q9CQN7	39S ribosomal protein L41, mitochondrial OS=Mus musculus GN=Mrpl41 PE=1 SV=1		9.6296296	1	1	1	15.252	9.82			660000	1.96
Q9CQZ5	NADH dehydrogenase [ubiquinone] 1 alpha subcomplex subunit 6 OS=Mus musculus GN		25.19084	3	5	3	15.273	10.11	6900000	980000	390000	11.29
P02301	Histone H3.3C OS=Mus musculus GN=H3f3c PE=3 SV=3		19.117647	4	18	4	15.305	11.14	350000000	160000000	230000000	39.81
Q6RUT7	Protein CCSMST1 OS=Mus musculus GN=Ccsmst1 PE=1 SV=1		18.382353	1	1	1	15.339	4.93				2.92
Q8CFV4	Neuritin OS=Mus musculus GN=Nrn1 PE=1 SV=1		16.197183	2	8	2	15.343	6.99	12000000	6200000	14000000	24.54
P84086	Complexin-2 OS=Mus musculus GN=Cplx2 PE=1 SV=1		28.358209	2	11	2	15.385	5.08	8500000	6800000	12000000	39.06
P62849	40S ribosomal protein S24 OS=Mus musculus GN=Rps24 PE=1 SV=1		19.548872	2	6	2	15.413	10.78	3400000	2100000	2300000	16.49
P63276	40S ribosomal protein S17 OS=Mus musculus GN=Rps17 PE=1 SV=2		17.037037	2	4	2	15.514	9.85		910000	1100000	12.00
Q8VEA4	Mitochondrial intermembrane space import and assembly protein 40 OS=Mus musculus G		20.143885	1	1	1	15.515	4.32		3300000		2.59
Q9CPQ3	Mitochondrial import receptor subunit TOM22 homolog OS=Mus musculus GN=Tomm22 I		30.28169	3	8	3	15.527	4.34	2400000	1900000	3100000	26.51
P41105	60S ribosomal protein L28 OS=Mus musculus GN=Rpl28 PE=1 SV=2		16.058394	2	7	2	15.724	12.02	21000000	12000000	14000000	18.48
Q62266	Cornifin-A OS=Mus musculus GN=Sprr1a PE=1 SV=1		16.666667	1	3	1	15.755	7.85		1200000	7000000	6.29
P61358	60S ribosomal protein L27 OS=Mus musculus GN=Rpl27 PE=1 SV=2		27.941176	3	6	3	15.788	10.56	13000000	5800000	16000000	14.30
P62267	40S ribosomal protein S23 OS=Mus musculus GN=Rps23 PE=1 SV=3		7.6923077	1	2	1	15.798	10.49	2000000	570000		3.90
Q9D6K5	Synaptojanin-2-binding protein OS=Mus musculus GN=Synj2bp PE=1 SV=1		15.172414	2	5	2	15.805	6.3	1400000	1400000	1500000	15.01
Q7M750	Opalin OS=Mus musculus GN=Opalin PE=1 SV=1		6.993007	1	1	1	15.823	4.88			260000	2.05
P02088	Hemoglobin subunit beta-1 OS=Mus musculus GN=Hbb-b1 PE=1 SV=2		53.741497	6	17	6	15.83	7.65	23000000	7800000	22000000	53.16
P62911	60S ribosomal protein L32 OS=Mus musculus GN=Rpl32 PE=1 SV=2		27.407407	3	6	3	15.85	11.33	820000	680000	3000000	15.31
Q9CQI6	Coactosin-like protein OS=Mus musculus GN=Cotl1 PE=1 SV=3		5.6338028	1	3	1	15.934	5.4	470000	920000	1800000	7.06
Q9CZX8	40S ribosomal protein S19 OS=Mus musculus GN=Rps19 PE=1 SV=3		37.241379	5	12	5	16.076	10.4	13000000	15000000	17000000	30.82
P12787	Cytochrome c oxidase subunit 5A, mitochondrial OS=Mus musculus GN=Cox5a PE=1 SV		21.232877	3	8	3	16.091	6.54	8500000	9900000	11000000	23.95
P62264	40S ribosomal protein S14 OS=Mus musculus GN=Rps14 PE=1 SV=3		31.788079	5	15	5	16.263	10.05	18000000	11000000	14000000	53.50
Q9CPW4	Actin-related protein 2/3 complex subunit 5 OS=Mus musculus GN=Arpc5 PE=1 SV=3		22.516556	2	5	2	16.278	5.67	1600000	950000	3400000	16.61
Q9CQX2	Cytochrome b5 type B OS=Mus musculus GN=Cyb5b PE=1 SV=1		44.520548	4	7	4	16.308	4.89	4900000	6100000	3700000	28.93
Q9CR61	NADH dehydrogenase [ubiquinone] 1 beta subcomplex subunit 7 OS=Mus musculus GN=		58.394161	7	17	7	16.32	8.18	28000000	27000000	24000000	52.04
O70480	Vesicle-associated membrane protein 4 OS=Mus musculus GN=Vamp4 PE=1 SV=1		9.929078	1	2	1	16.343	7.36	2000000		1100000	6.69
Q9CZY3	Ubiquitin-conjugating enzyme E2 variant 1 OS=Mus musculus GN=Ube2v1 PE=1 SV=1		17.687075	2	4	2	16.344	7.96	6000000	760000	1700000	12.67
P14131	40S ribosomal protein S16 OS=Mus musculus GN=Rps16 PE=1 SV=4		12.328767	2	4	2	16.435	10.21	3100000	1600000	2800000	9.62
Q60872	Eukaryotic translation initiation factor 1A OS=Mus musculus GN=Eif1a PE=2 SV=3		7.6388889	1	3	1	16.492	5.24	8800000	3200000	6600000	10.03
Q9D6K8	FUN14 domain-containing protein 2 OS=Mus musculus GN=Fundc2 PE=1 SV=1		7.9470199	1	1	1	16.554	9.7	3700000			2.40
P14115	60S ribosomal protein L27a OS=Mus musculus GN=Rpl27a PE=1 SV=5		22.297297	3	8	3	16.595	11.12	11000000	5300000	6500000	21.44
Q9CQI3	Glia maturation factor beta OS=Mus musculus GN=Gmfb PE=1 SV=3		7.7464789	1	1	1	16.712	5.16		890000		2.65
P62204	Calmodulin OS=Mus musculus GN=Calm1 PE=1 SV=2		50.33557	8	36	8	16.827	4.22	120000000	130000000	120000000	89.27
Q9ERS2	NADH dehydrogenase [ubiquinone] 1 alpha subcomplex subunit 13 OS=Mus musculus G		38.888889	5	19	5	16.849	9.48	16000000	7700000	14000000	52.79
Q9D8W7	OCIA domain-containing protein 2 OS=Mus musculus GN=Ociad2 PE=1 SV=1		11.038961	2	3	2	16.915	9.41		870000	1500000	6.94
Q60605	Myosin light polypeptide 6 OS=Mus musculus GN=Myl6 PE=1 SV=3		30.463576	4	10	4	16.919	4.65	4700000	3800000	3600000	27.79
Q9CPU4	Microsomal glutathione S-transferase 3 OS=Mus musculus GN=Mgst3 PE=1 SV=1		23.529412	2	6	2	16.947	9.5	3500000	1800000	2400000	17.68

Q9D898	Actin-related protein 2/3 complex subunit 5-like protein OS=Mus musculus GN=Arpc5l PE	32.679739	3	7	3	16.97	6.8	5100000	3200000	5200000	21.54
Q9CQ92	Mitochondrial fission 1 protein OS=Mus musculus GN=Fis1 PE=1 SV=1	23.684211	3	7	3	16.998	8.53	8000000	3700000	8100000	20.54
P62743	AP-2 complex subunit sigma OS=Mus musculus GN=Ap2s1 PE=1 SV=1	28.169014	4	12	4	17.007	6.18	20000000	13000000	17000000	27.50
Q7TMF3	NADH dehydrogenase [ubiquinone] 1 alpha subcomplex subunit 12 OS=Mus musculus G	54.482759	7	24	7	17.076	9.36	10000000	6400000	10000000	73.33
P61327	Protein mago nashi homolog OS=Mus musculus GN=Magoh PE=2 SV=1	7.5342466	1	2	1	17.153	6.11	2800000		2800000	5.42
P15532	Nucleoside diphosphate kinase A OS=Mus musculus GN=Nme1 PE=1 SV=1	6.5789474	1	2	1	17.197	7.37	7700000	4900000		4.92
P62301	40S ribosomal protein S13 OS=Mus musculus GN=Rps13 PE=1 SV=2	16.556291	2	6	2	17.212	10.54	3300000	2400000	2400000	16.27
Q9CRB2	H/ACA ribonucleoprotein complex subunit 2 OS=Mus musculus GN=Nhp2 PE=1 SV=1	11.111111	1	3	1	17.236	8.41	620000	240000	760000	9.57
P61255	60S ribosomal protein L26 OS=Mus musculus GN=Rpl26 PE=1 SV=1	16.551724	3	5	3	17.248	10.55	13000000	6400000		10.91
Q9CQH7	Transcription factor BTF3 homolog 4 OS=Mus musculus GN=Btf3l4 PE=1 SV=1	24.050633	2	8	2	17.26	6.35	5400000	2600000	5700000	27.38
P54227	Stathmin OS=Mus musculus GN=Stmn1 PE=1 SV=2	20.805369	3	11	3	17.264	5.97	15000000	8900000	13000000	25.08
Q9CYR0	Single-stranded DNA-binding protein, mitochondrial OS=Mus musculus GN=Ssbp1 PE=1	9.8684211	1	1	1	17.308	9.92		620000		3.24
Q01768	Nucleoside diphosphate kinase B OS=Mus musculus GN=Nme2 PE=1 SV=1	9.2105263	1	3	1	17.352	7.5	4700000	2300000	3500000	9.07
Q9CR21	Acyl carrier protein, mitochondrial OS=Mus musculus GN=Ndufab1 PE=1 SV=1	8.974359	1	2	1	17.359	5.21		1200000	1000000	5.22
Q9D6G9	CKLF-like MARVEL transmembrane domain-containing protein 5 OS=Mus musculus GN=	12.179487	1	2	1	17.378	4.84	4300000		3400000	9.28
O09111	NADH dehydrogenase [ubiquinone] 1 beta subcomplex subunit 11, mitochondrial OS=Mus	46.357616	6	36	6	17.433	5.22	17000000	15000000	29000000	125.30
Q9QYK7	RING finger protein 11 OS=Mus musculus GN=Rnf11 PE=1 SV=1	7.7922078	1	1	1	17.446	4.78		860000		2.56
Q9D3D9	ATP synthase subunit delta, mitochondrial OS=Mus musculus GN=Atp5d PE=1 SV=1	13.690476	2	5	2	17.589	5.08	4000000	5300000	5200000	15.66
P62751	60S ribosomal protein L23a OS=Mus musculus GN=Rpl23a PE=1 SV=1	20.512821	3	12	3	17.684	10.45	34000000	18000000	22000000	33.56
P62270	40S ribosomal protein S18 OS=Mus musculus GN=Rps18 PE=1 SV=3	19.078947	3	8	3	17.708	10.99	9600000	6400000	10000000	21.87
Q8BP67	60S ribosomal protein L24 OS=Mus musculus GN=Rpl24 PE=1 SV=2	8.2802548	1	4	1	17.768	11.25	2400000	1700000	1700000	11.75
P35979	60S ribosomal protein L12 OS=Mus musculus GN=Rpl12 PE=1 SV=2	45.454545	5	13	5	17.794	9.42	9700000	4600000	6600000	45.79
Q9ERR7	15 kDa selenoprotein OS=Mus musculus GN=Sep15 PE=1 SV=3	17.283951	2	5	2	17.796	5.35	560000	550000	470000	13.25
P62983	Ubiquitin-40S ribosomal protein S27a OS=Mus musculus GN=Rps27a PE=1 SV=2	25.641026	4	19	4	17.939	9.64	7400000	140000000	170000000	61.02
P17742	Peptidyl-prolyl cis-trans isomerase A OS=Mus musculus GN=Ppia PE=1 SV=2	30.487805	5	21	5	17.96	7.9	44000000	20000000	38000000	55.72
P01831	Thy-1 membrane glycoprotein OS=Mus musculus GN=Thy1 PE=1 SV=1	37.654321	6	27	6	18.069	8.97	71000000	56000000	66000000	85.08
Q9D958	Signal peptidase complex subunit 1 OS=Mus musculus GN=Spcs1 PE=2 SV=3	7.4534161	1	1	1	18.174	10.01	3400000			2.15
Q9CRB8	Mitochondrial fission process protein 1 OS=Mus musculus GN=Mtfp1 PE=1 SV=1	6.626506	1	1	1	18.303	8.68		420000		2.09
Q9QUR7	Peptidyl-prolyl cis-trans isomerase NIMA-interacting 1 OS=Mus musculus GN=Pin1 PE=1	20	2	4	2	18.359	8.79	1300000	1400000	3200000	13.18
P62281	40S ribosomal protein S11 OS=Mus musculus GN=Rps11 PE=1 SV=3	25.949367	5	10	5	18.419	10.3	11000000	7000000	11000000	22.95
Q9D328	Transmembrane protein 35 OS=Mus musculus GN=Tmem35 PE=1 SV=1	8.9820359	1	4	1	18.493	9.99	6700000	2000000	4000000	10.88
Q9R0P5	Destrin OS=Mus musculus GN=Dstn PE=1 SV=3	19.393939	3	12	3	18.509	7.97	6000000	2300000	6700000	36.20
P18760	Cofilin-1 OS=Mus musculus GN=Cfl1 PE=1 SV=3	57.831325	9	25	8	18.548	8.09	22000000	15000000	47000000	89.82
Q64327	Male-enhanced antigen 1 OS=Mus musculus GN=Mea1 PE=1 SV=1	7.4712644	1	2	1	18.573	4.08	590000	830000		5.11
P60824	Cold-inducible RNA-binding protein OS=Mus musculus GN=Cirbp PE=1 SV=1	12.790698	2	3	2	18.596	9.61	3300000	1700000	2100000	6.21
Q5M8N0	CB1 cannabinoid receptor-interacting protein 1 OS=Mus musculus GN=Cnrip1 PE=1 SV=	9.7560976	1	1	1	18.601	7.96		1200000		3.72
Q9WTX5	S-phase kinase-associated protein 1 OS=Mus musculus GN=Skp1 PE=1 SV=3	21.472393	5	13	5	18.66	4.54	11000000	9900000	13000000	39.00
P45591	Cofilin-2 OS=Mus musculus GN=Cfl2 PE=1 SV=1	36.746988	5	8	4	18.698	7.88	1700000		2600000	23.29
Q9R0Q7	Prostaglandin E synthase 3 OS=Mus musculus GN=Ptges3 PE=1 SV=1	23.75	3	8	3	18.709	4.55	6800000	4000000	6700000	23.91
P61967	AP-1 complex subunit sigma-1A OS=Mus musculus GN=Ap1s1 PE=1 SV=1	10.126582	1	4	1	18.721	5.73	5800000	2500000	3800000	16.81
Q9DCX2	ATP synthase subunit d, mitochondrial OS=Mus musculus GN=Atp5h PE=1 SV=3	31.055901	5	11	5	18.738	5.69	27000000	15000000	22000000	29.18
O70492	Sorting nexin-3 OS=Mus musculus GN=Snx3 PE=1 SV=3	17.901235	3	7	3	18.746	8.66	2100000	1300000	2500000	18.56
P63325	40S ribosomal protein S10 OS=Mus musculus GN=Rps10 PE=1 SV=1	8.4848485	1	3	1	18.904	10.15	7300000	4200000	4400000	11.11
Q9DB50	AP-1 complex subunit sigma-2 OS=Mus musculus GN=Ap1s2 PE=1 SV=1	8.125	1	1	1	18.917	5.48		490000		2.80
Q62186	Translocon-associated protein subunit delta OS=Mus musculus GN=Ssr4 PE=1 SV=1	25	3	6	3	18.924	5.78	2900000	1800000	1700000	19.47
P97457	Myosin regulatory light chain 2, skeletal muscle isoform OS=Mus musculus GN=Mylpf PE=	7.6923077	1	1	1	18.943	4.92		450000		2.70
Q8C1M2	Zinc finger protein 428 OS=Mus musculus GN=Znf428 PE=1 SV=1	6.25	1	2	1	18.991	4.23	460000		470000	4.00
O70493	Sorting nexin-12 OS=Mus musculus GN=Snx12 PE=1 SV=1	15.151515	2	4	2	19.104	7.34	1600000	760000	1500000	9.04

Q9D2P8	Myelin-associated oligodendrocyte basic protein OS=Mus musculus GN=Mobp PE=1 SV=	6.4705882	1	2	1	19.185	11.14		540000	3000000	4.48
Q63810	Calcineurin subunit B type 1 OS=Mus musculus GN=Ppp3r1 PE=1 SV=3	22.352941	3	10	3	19.288	4.81	34000000	32000000	44000000	32.14
P84104	Serine/arginine-rich splicing factor 3 OS=Mus musculus GN=Srsf3 PE=1 SV=1	25	4	10	3	19.318	11.65	9100000		6500000	27.94
Q9CZH7	Matrix-remodeling-associated protein 7 OS=Mus musculus GN=Mxra7 PE=1 SV=2	31.460674	2	5	2	19.445	4.26	880000	500000	1200000	16.80
P63028	Translationally-controlled tumor protein OS=Mus musculus GN=Tpt1 PE=1 SV=1	15.697674	2	7	2	19.45	4.86	9000000	5400000	9100000	26.77
Q9JIG8	PRA1 family protein 2 OS=Mus musculus GN=Praf2 PE=1 SV=1	10.11236	2	3	2	19.466	9.6	5600000		3200000	7.58
P19783	Cytochrome c oxidase subunit 4 isoform 1, mitochondrial OS=Mus musculus GN=Cox4i1	36.686391	7	27	7	19.518	9.23	41000000	28000000	46000000	75.83
Q9D938	Transmembrane protein 160 OS=Mus musculus GN=Tmem160 PE=2 SV=1	6.9148936	1	2	1	19.575	7.3		2100000	2000000	5.20
Q59J78	Mimitin, mitochondrial OS=Mus musculus GN=Ndufaf2 PE=1 SV=1	9.5238095	1	3	1	19.616	8.25	2900000	2600000	3400000	9.78
Q99LW6	YY1-associated factor 2 OS=Mus musculus GN=Yaf2 PE=1 SV=1	13.407821	1	1	1	19.643	9.58	52000			2.13
P59999	Actin-related protein 2/3 complex subunit 4 OS=Mus musculus GN=Arpc4 PE=1 SV=3	16.071429	3	9	3	19.654	8.43	8900000	6600000	8600000	20.98
Q3THE2	Myosin regulatory light chain 12B OS=Mus musculus GN=Myl12b PE=1 SV=2	24.418605	4	13	4	19.767	4.84	11000000	7800000	12000000	37.77
Q9CXZ1	NADH dehydrogenase [ubiquinone] iron-sulfur protein 4, mitochondrial OS=Mus musculus	25.142857	4	10	4	19.772	9.99	30000000	17000000	23000000	29.08
Q63739	Protein tyrosine phosphatase type IVA 1 OS=Mus musculus GN=Ptp4a1 PE=1 SV=1	4.0462428	1	1	1	19.802	8.97		900000		2.43
Q9CWZ3	RNA-binding protein 8A OS=Mus musculus GN=Rbm8a PE=1 SV=3	21.83908	3	9	3	19.876	6.16	7300000	240000	6300000	29.89
Q9DCJ5	NADH dehydrogenase [ubiquinone] 1 alpha subcomplex subunit 8 OS=Mus musculus GN	19.767442	3	10	3	19.979	8.46	9000000	7000000	8200000	32.22
Q99LX0	Protein deglycase DJ-1 OS=Mus musculus GN=Park7 PE=1 SV=1	7.4074074	1	2	1	20.008	6.77		710000		4.84
Q9R0P4	Small acidic protein OS=Mus musculus GN=Smap PE=1 SV=1	8.839779	1	5	1	20.034	4.82			2000000	15.98
P62331	ADP-ribosylation factor 6 OS=Mus musculus GN=Arf6 PE=1 SV=2	6.2857143	1	2	1	20.069	8.95		1900000	3900000	4.38
Q9CZX9	ER membrane protein complex subunit 4 OS=Mus musculus GN=Emc4 PE=1 SV=1	8.7431694	1	1	1	20.103	8.62	1400000			2.95
Q9QZB9	Dynactin subunit 5 OS=Mus musculus GN=Dctn5 PE=1 SV=1	6.043956	1	4	1	20.127	8.02	1700000	1400000	2300000	8.78
Q8R2U6	Diphosphoinositol polyphosphate phosphohydrolase 2 OS=Mus musculus GN=Nudt4 PE=	5.027933	1	1	1	20.143	6.35	160000			2.15
P28667	MARCKS-related protein OS=Mus musculus GN=Marcksl1 PE=1 SV=2	65	5	11	5	20.153	4.61	3900000	1900000	3200000	38.51
P61924	Coatomer subunit zeta-1 OS=Mus musculus GN=Copz1 PE=1 SV=1	8.4745763	1	2	1	20.185	4.81		590000	350000	5.31
Q9CXW4	60S ribosomal protein L11 OS=Mus musculus GN=Rpl11 PE=1 SV=4	16.853933	3	5	3	20.24	9.6		5500000	6200000	11.36
O55013	Trafficking protein particle complex subunit 3 OS=Mus musculus GN=Trappc3 PE=1 SV=	10	2	2	2	20.289	4.96			850000	3.99
Q8VE22	28S ribosomal protein S23, mitochondrial OS=Mus musculus GN=Mrps23 PE=1 SV=1	7.9096045	1	2	1	20.335	8.59		800000	900000	3.87
Q9CXI5	Mesencephalic astrocyte-derived neurotrophic factor OS=Mus musculus GN=Manf PE=1	7.8212291	1	1	1	20.361	8.07		400000		2.20
P61750	ADP-ribosylation factor 4 OS=Mus musculus GN=Arf4 PE=1 SV=2	11.666667	2	4	1	20.384	7.14	2200000	950000	2800000	9.72
P61211	ADP-ribosylation factor-like protein 1 OS=Mus musculus GN=Arl1 PE=1 SV=1	7.7348066	1	2	1	20.398	5.72	1500000		1200000	7.04
Q9QZ88	Vacuolar protein sorting-associated protein 29 OS=Mus musculus GN=Vps29 PE=1 SV=1	10.989011	2	3	2	20.483	6.79	11000000		16000000	7.16
P61226	Ras-related protein Rap-2b OS=Mus musculus GN=Rap2b PE=1 SV=1	31.147541	6	17	3	20.491	4.81	2800000	2500000	2900000	47.70
Q9JM76	Actin-related protein 2/3 complex subunit 3 OS=Mus musculus GN=Arpc3 PE=1 SV=3	7.3033708	1	4	1	20.511	8.59	5800000	1700000	4800000	15.21
P84084	ADP-ribosylation factor 5 OS=Mus musculus GN=Arf5 PE=1 SV=2	25.555556	4	10	2	20.517	6.79	6500000	2100000	3400000	27.92
P97315	Cysteine and glycine-rich protein 1 OS=Mus musculus GN=Csrp1 PE=1 SV=3	38.341969	5	12	5	20.57	8.57	7400000	3700000	6600000	39.20
P61205	ADP-ribosylation factor 3 OS=Mus musculus GN=Arf3 PE=2 SV=2	32.044199	5	12	3	20.588	7.43	19000000	6500000	10000000	35.20
Q3UHX2	28 kDa heat- and acid-stable phosphoprotein OS=Mus musculus GN=Pdap1 PE=1 SV=1	32.044199	4	13	4	20.593	7.39	4100000	1500000	4800000	45.57
Q80ZJ1	Ras-related protein Rap-2a OS=Mus musculus GN=Rap2a PE=1 SV=2	26.229508	5	12	2	20.629	4.82		1400000	3400000	32.31
P62717	60S ribosomal protein L18a OS=Mus musculus GN=Rpl18a PE=1 SV=1	19.318182	3	5	3	20.719	10.71	3300000	1300000	2400000	12.95
Q9JKL4	NADH dehydrogenase [ubiquinone] 1 alpha subcomplex assembly factor 3 OS=Mus musc	5.9459459	1	2	1	20.721	8.05		1100000	1800000	4.21
Q9QXT0	Protein canopy homolog 2 OS=Mus musculus GN=Cnpy2 PE=1 SV=1	6.5934066	1	1	1	20.754	5.07			520000	2.80
Q99JI6	Ras-related protein Rap-1b OS=Mus musculus GN=Rap1b PE=1 SV=2	23.369565	5	9	1	20.812	5.78	1900000	1600000	1800000	23.07
P23198	Chromobox protein homolog 3 OS=Mus musculus GN=Cbx3 PE=1 SV=2	13.661202	3	10	3	20.842	5.22	15000000	2700000	12000000	39.77
P10922	Histone H1.0 OS=Mus musculus GN=H1f0 PE=2 SV=4	16.494845	3	11	3	20.848	10.9	12000000	7700000	13000000	32.83
Q8C8T8	Pre-rRNA-processing protein TSR2 homolog OS=Mus musculus GN=Tsr2 PE=1 SV=1	15.183246	2	5	2	20.878	4.23	2600000	1200000	2600000	16.69
P61082	NEDD8-conjugating enzyme Ubc12 OS=Mus musculus GN=Ube2m PE=1 SV=1	5.4644809	1	3	1	20.887	7.69	2200000	910000	1600000	8.34
Q4QQM4	Tumor protein p53-inducible protein 11 OS=Mus musculus GN=Trp53i11 PE=1 SV=1	13.227513	2	3	2	20.897	9.41	2900000		1500000	7.90
Q62092	Neuron-specific protein family member 1 OS=Mus musculus GN=Nsg1 PE=1 SV=3	5.9459459	1	1	1	20.916	6.21			390000	3.51

Q9Z0Y1	Dynactin subunit 3 OS=Mus musculus GN=Dctn3 PE=1 SV=2		4.3010753	1	1	1	20.965	6.06		790000		1.91
P62835	Ras-related protein Rap-1A OS=Mus musculus GN=Rap1a PE=1 SV=1		23.369565	5	12	1	20.974	6.67	20000000	5000000	16000000	30.49
Q8K4M5	COMM domain-containing protein 1 OS=Mus musculus GN=Commd1 PE=1 SV=2		7.4468085	1	1	1	20.983	7.59		240000		2.41
Q9DCS9	NADH dehydrogenase [ubiquinone] 1 beta subcomplex subunit 10 OS=Mus musculus GN		59.659091	9	30	9	21.01	8.03	12000000	14000000	15000000	89.22
Q60870	Receptor expression-enhancing protein 5 OS=Mus musculus GN=Reep5 PE=1 SV=1		10.27027	2	6	2	21.037	8.38	5400000	2800000	5800000	17.77
Q9DCF9	Translocon-associated protein subunit gamma OS=Mus musculus GN=Ssr3 PE=1 SV=1		7.5675676	1	1	1	21.051	9.61		1600000		2.74
Q9R257	Heme-binding protein 1 OS=Mus musculus GN=Hebp1 PE=1 SV=2		4.2105263	1	1	1	21.053	5.26		120000		2.07
O09114	Prostaglandin-H2 D-isomerase OS=Mus musculus GN=Ptgds PE=1 SV=1		8.994709	1	2	1	21.053	8.25		1600000	2700000	8.69
P09528	Ferritin heavy chain OS=Mus musculus GN=Fth1 PE=1 SV=2		15.384615	2	3	2	21.053	5.88	1900000		1900000	8.23
Q921L3	Calcium load-activated calcium channel OS=Mus musculus GN=Tmco1 PE=1 SV=1		7.4468085	1	3	1	21.161	9.74	1100000			9.66
P60766	Cell division control protein 42 homolog OS=Mus musculus GN=Cdc42 PE=1 SV=2		31.413613	5	16	4	21.245	6.55	3600000	3300000	5400000	45.77
Q61411	GTPase HRas OS=Mus musculus GN=Hras PE=1 SV=2		25.925926	4	9	4	21.285	5.31	5200000	4400000	6800000	22.11
P84096	Rho-related GTP-binding protein RhoG OS=Mus musculus GN=Rhog PE=1 SV=1		23.560209	3	9	2	21.295	8.12	2000000	810000	2100000	28.62
Q8BK08	Transmembrane protein 11, mitochondrial OS=Mus musculus GN=Tmem11 PE=1 SV=1		4.7368421	1	2	1	21.298	7.36	5300000		4900000	4.04
Q8VEH3	ADP-ribosylation factor-like protein 8A OS=Mus musculus GN=Arl8a PE=1 SV=1		18.27957	3	8	2	21.376	7.77	8900000	2700000	7300000	20.70
P83917	Chromobox protein homolog 1 OS=Mus musculus GN=Cbx1 PE=1 SV=1		15.135135	3	5	3	21.405	4.93	6200000		4800000	16.89
Q9CPR4	60S ribosomal protein L17 OS=Mus musculus GN=Rpl17 PE=1 SV=3		22.826087	4	7	4	21.409	10.18	11000000	4800000	3200000	16.56
P63001	Ras-related C3 botulinum toxin substrate 1 OS=Mus musculus GN=Rac1 PE=1 SV=1		29.6875	5	15	4	21.436	8.5	17000000	8000000	12000000	38.85
O35988	Syndecan-4 OS=Mus musculus GN=Sdc4 PE=1 SV=1		12.121212	2	4	2	21.469	4.41	6500000	4200000	4400000	13.21
Q9CQW2	ADP-ribosylation factor-like protein 8B OS=Mus musculus GN=Arl8b PE=1 SV=1		17.204301	3	9	2	21.525	8.43	9000000	5600000	12000000	22.24
Q9D8Y1	Transmembrane protein 126A OS=Mus musculus GN=Tmem126a PE=1 SV=1		6.122449	1	1	1	21.526	9.41	1500000			2.09
Q8R5J9	PRA1 family protein 3 OS=Mus musculus GN=Arl6ip5 PE=1 SV=2		5.8510638	1	3	1	21.543	9.61	11000000	3500000	8600000	9.04
Q6P069	Sorcin OS=Mus musculus GN=Sri PE=1 SV=1		5.5555556	1	2	1	21.613	5.59		1300000	640000	4.43
P35980	60S ribosomal protein L18 OS=Mus musculus GN=Rpl18 PE=1 SV=3		33.510638	6	15	6	21.631	11.78	22000000	11000000	10000000	44.84
P32883	GTPase KRas OS=Mus musculus GN=Kras PE=1 SV=1		17.989418	3	10	3	21.642	6.77	4000000	2300000	5500000	21.92
O55022	Membrane-associated progesterone receptor component 1 OS=Mus musculus GN=Pgrm		54.358974	8	26	7	21.681	4.7	9700000	13000000	13000000	97.64
Q9DB15	39S ribosomal protein L12, mitochondrial OS=Mus musculus GN=Mrpl12 PE=1 SV=2		6.9651741	1	2	1	21.695	9.29			3700000	6.16
Q9CQH3	NADH dehydrogenase [ubiquinone] 1 beta subcomplex subunit 5, mitochondrial OS=Mus		21.164021	4	19	4	21.696	9.41	15000000	19000000	23000000	51.25
Q9DCR2	AP-3 complex subunit sigma-1 OS=Mus musculus GN=Ap3s1 PE=1 SV=2		7.7720207	1	3	1	21.718	5.39		1400000	1200000	7.50
Q61171	Peroxiredoxin-2 OS=Mus musculus GN=Prdx2 PE=1 SV=3		31.313131	5	9	5	21.765	5.41	3700000	4100000	7900000	24.31
Q60829	Protein phosphatase 1 regulatory subunit 1B OS=Mus musculus GN=Ppp1r1b PE=1 SV=		8.2474227	1	3	1	21.767	4.65	270000		1500000	11.48
Q9QUI0	Transforming protein RhoA OS=Mus musculus GN=Rhoa PE=1 SV=1		31.606218	6	15	4	21.768	6.1	11000000	4400000	9200000	41.93
O88952	Protein lin-7 homolog C OS=Mus musculus GN=Lin7c PE=1 SV=2		32.48731	5	10	1	21.82	8.43			1700000	27.72
P12815	Programmed cell death protein 6 OS=Mus musculus GN=Pdcd6 PE=1 SV=2		10.471204	2	2	2	21.854	5.4		1200000		5.72
Q9D6J5	NADH dehydrogenase [ubiquinone] 1 beta subcomplex subunit 8, mitochondrial OS=Mus		30.107527	4	8	4	21.862	6.64	2400000	8700000	8100000	19.84
Q8BNY6	Neuronal calcium sensor 1 OS=Mus musculus GN=Ncs1 PE=1 SV=3		25.263158	4	9	4	21.865	4.83	3000000	3800000	4300000	24.09
P51410	60S ribosomal protein L9 OS=Mus musculus GN=Rpl9 PE=2 SV=2		5.2083333	1	3	1	21.868	9.95	14000000	6500000	8800000	7.86
Q9D892	Inosine triphosphate pyrophosphatase OS=Mus musculus GN=Itpa PE=1 SV=2		8.5858586	1	2	1	21.883	5.87			930000	5.89
P99029	Peroxiredoxin-5, mitochondrial OS=Mus musculus GN=Prdx5 PE=1 SV=2		18.571429	3	4	3	21.884	8.85	13000000	1300000	21000000	12.37
P43274	Histone H1.4 OS=Mus musculus GN=Hist1h1e PE=1 SV=2		14.611872	3	6	1	21.964	11.11	28000000	23000000	25000000	16.77
Q9CQJ8	NADH dehydrogenase [ubiquinone] 1 beta subcomplex subunit 9 OS=Mus musculus GN=		29.608939	5	29	5	21.97	7.8	8500000	4100000	7900000	92.47
Q8BSZ2	AP-3 complex subunit sigma-2 OS=Mus musculus GN=Ap3s2 PE=1 SV=1		5.1813472	1	1	1	22.003	5.22		760000		2.10
Q64152	Transcription factor BTF3 OS=Mus musculus GN=Btf3 PE=1 SV=3		12.254902	1	2	1	22.017	9.52	1400000		1400000	7.09
Q8CCT4	Transcription elongation factor A protein-like 5 OS=Mus musculus GN=Tceal5 PE=1 SV=		12	3	5	1	22.025	6.2	4400000	2000000	4500000	12.17
Q91XV3	Brain acid soluble protein 1 OS=Mus musculus GN=Basp1 PE=1 SV=3		71.681416	11	131	11	22.074	4.51	430000000	430000000	400000000	497.31
P43277	Histone H1.3 OS=Mus musculus GN=Hist1h1d PE=1 SV=2		14.479638	3	6	1	22.086	11.03	13000000	15000000	13000000	15.54
P60904	DnaJ homolog subfamily C member 5 OS=Mus musculus GN=Dnajc5 PE=1 SV=1		44.949495	5	16	5	22.086	5.07	16000000	7100000	14000000	53.76
Q60771	Claudin-11 OS=Mus musculus GN=Cldn11 PE=1 SV=1		6.763285	1	3	1	22.099	7.91	7300000	2600000	4900000	9.08

P62746	Rho-related GTP-binding protein RhoB OS=Mus musculus GN=Rhob PE=1 SV=1	38.265306	8	19	6	22.109	5.24	11000000	6600000	10000000	54.84
P62082	40S ribosomal protein S7 OS=Mus musculus GN=Rps7 PE=2 SV=1	4.6391753	1	3	1	22.113	10.1				6.15
P43407	Syndecan-2 OS=Mus musculus GN=Sdc2 PE=1 SV=1	4.950495	1	2	1	22.117	4.58		380000	450000	4.04
P62761	Visinin-like protein 1 OS=Mus musculus GN=Vsnl1 PE=1 SV=2	67.015707	13	59	11	22.128	5.15	61000000	48000000	59000000	170.58
Q9DBP5	UMP-CMP kinase OS=Mus musculus GN=Cmpk1 PE=1 SV=1	5.6122449	1	1	1	22.151	5.83			1600000	2.62
Q9CQJ6	Density-regulated protein OS=Mus musculus GN=Denr PE=1 SV=1	21.212121	3	4	3	22.152	5.3		3100000	3500000	14.43
P35700	Peroxiredoxin-1 OS=Mus musculus GN=Prdx1 PE=1 SV=1	48.241206	8	14	8	22.162	8.12	6400000	3300000	6800000	34.73
Q99MS3	Mpv17-like protein OS=Mus musculus GN=Mpv17l PE=1 SV=2	4.1237113	1	1	1	22.166	10.05	580000			2.00
Q61686	Chromobox protein homolog 5 OS=Mus musculus GN=Cbx5 PE=1 SV=1	29.319372	5	7	5	22.172	5.86	6600000		3800000	20.33
Q9D1G1	Ras-related protein Rab-1B OS=Mus musculus GN=Rab1b PE=1 SV=1	50.248756	9	26	3	22.173	5.73	9700000	4300000	7500000	67.24
Q8BGZ1	Hippocalcin-like protein 4 OS=Mus musculus GN=Hpcal4 PE=1 SV=3	48.691099	8	29	6	22.201	4.89	16000000	13000000	18000000	86.04
Q91X97	Neurocalcin-delta OS=Mus musculus GN=Ncald PE=1 SV=4	29.015544	5	20	3	22.231	5.35	6000000	4100000	4600000	56.17
Q91Z61	GTP-binding protein Di-Ras1 OS=Mus musculus GN=Diras1 PE=1 SV=1	5.0505051	1	2	1	22.25	8.9	970000		860000	4.40
Q9D6N5	Dr1-associated corepressor OS=Mus musculus GN=Drap1 PE=1 SV=3	10.731707	1	1	1	22.264	5.26	620000			2.14
Q9CQW1	Synaptobrevin homolog YKT6 OS=Mus musculus GN=Ykt6 PE=1 SV=1	8.5858586	1	1	1	22.3	6.35		190000		1.96
P11352	Glutathione peroxidase 1 OS=Mus musculus GN=Gpx1 PE=1 SV=2	13.432836	2	2	2	22.316	7.21		360000		4.60
P62748	Hippocalcin-like protein 1 OS=Mus musculus GN=Hpcal1 PE=1 SV=2	22.797927	4	12	1	22.324	5.5	1000000	1200000		31.44
P36536	GTP-binding protein SAR1a OS=Mus musculus GN=Sar1a PE=1 SV=1	9.5959596	2	3	2	22.357	6.93	1600000	1200000	3100000	5.97
P61087	Ubiquitin-conjugating enzyme E2 K OS=Mus musculus GN=Ube2k PE=1 SV=3	6	1	2	1	22.393	5.44		500000	860000	5.07
P84075	Neuron-specific calcium-binding protein hippocalcin OS=Mus musculus GN=Hpca PE=1 S	40.932642	7	20	3	22.413	4.97	6700000	4400000	6100000	52.49
Q9JMG7	Hepatoma-derived growth factor-related protein 3 OS=Mus musculus GN=Hdgfrp3 PE=1	25.742574	3	8	3	22.417	8.4	14000000		5000000	24.53
P61022	Calcineurin B homologous protein 1 OS=Mus musculus GN=Chp1 PE=1 SV=2	10.25641	2	4	2	22.418	5.1	1100000	1100000	1400000	8.03
P61759	Prefoldin subunit 3 OS=Mus musculus GN=Vbp1 PE=1 SV=2	10.204082	2	3	2	22.421	6.28		660000	690000	7.25
Q8R0A5	Transcription elongation factor A protein-like 3 OS=Mus musculus GN=Tceal3 PE=1 SV=	23.5	4	10	2	22.455	5.44	2400000	900000	1700000	24.18
Q9R1Q8	Transgelin-3 OS=Mus musculus GN=Tagln3 PE=1 SV=1	13.065327	3	5	3	22.456	7.33	1500000	2000000	1700000	11.11
Q5PR73	GTP-binding protein Di-Ras2 OS=Mus musculus GN=Diras2 PE=1 SV=1	17.58794	3	7	3	22.484	8.76	3800000	2000000	3400000	20.06
P61027	Ras-related protein Rab-10 OS=Mus musculus GN=Rab10 PE=1 SV=1	39	7	25	5	22.527	8.38	17000000	5800000	4500000	70.38
P43276	Histone H1.5 OS=Mus musculus GN=Hist1h1b PE=1 SV=2	10.313901	2	2	2	22.562	10.92	7400000	2600000		4.87
Q6ZWN5	40S ribosomal protein S9 OS=Mus musculus GN=Rps9 PE=1 SV=3	28.350515	7	14	7	22.578	10.65	19000000	4500000	9400000	31.17
Q9DCZ4	MICOS complex subunit Mic26 OS=Mus musculus GN=Apoo PE=1 SV=2	18.181818	3	7	3	22.59	9.25	9500000	6300000	14000000	21.09
Q9JJR8	Transmembrane protein 9B OS=Mus musculus GN=Tmem9b PE=1 SV=1	10.552764	2	2	2	22.593	8.18			620000	1.81
P62821	Ras-related protein Rab-1A OS=Mus musculus GN=Rab1A PE=1 SV=3	47.804878	9	37	3	22.663	6.21	8800000	5400000	8500000	97.59
Q9R0Q3	Transmembrane emp24 domain-containing protein 2 OS=Mus musculus GN=Tmed2 PE=	4.4776119	1	6	1	22.69	5.17	4400000	3100000	4400000	14.07
Q9CXE2	B-cell CLL/lymphoma 7 protein family member A OS=Mus musculus GN=Bcl7a PE=2 SV=	12.857143	1	1	1	22.767	5.06				4.38
P97461	40S ribosomal protein S5 OS=Mus musculus GN=Rps5 PE=1 SV=3	3.9215686	1	3	1	22.875	9.72	6200000	4400000	5700000	6.40
Q8BGR6	ADP-ribosylation factor-like protein 15 OS=Mus musculus GN=Arl15 PE=1 SV=1	4.4117647	1	1	1	22.891	5.29			1000000	2.18
Q9R1P3	Proteasome subunit beta type-2 OS=Mus musculus GN=Psmb2 PE=1 SV=1	9.9502488	2	4	2	22.892	7.02	1800000	1800000	530000	10.92
Q9R1P1	Proteasome subunit beta type-3 OS=Mus musculus GN=Psmb3 PE=1 SV=1	7.804878	1	1	1	22.949	6.55	2700000			3.58
Q9CQ37	Ubiquitin-conjugating enzyme E2 T OS=Mus musculus GN=Ube2t PE=1 SV=1	3.4313725	1	1	1	22.961	7.97		420000		1.85
Q9CQU3	Protein RER1 OS=Mus musculus GN=Rer1 PE=1 SV=1	9.1836735	1	1	1	22.973	9.51	1300000			3.12
O54879	High mobility group protein B3 OS=Mus musculus GN=Hmgb3 PE=1 SV=3	6.5	1	1	1	22.995	8.37	1500000			2.46
Q6PHN9	Ras-related protein Rab-35 OS=Mus musculus GN=Rab35 PE=1 SV=1	15.422886	3	13	1	23.011	8.29	18000000		21000000	32.73
P35293	Ras-related protein Rab-18 OS=Mus musculus GN=Rab18 PE=1 SV=2	31.553398	5	11	5	23.021	5.36	7000000	3900000	6400000	34.46
Q923S9	Ras-related protein Rab-30 OS=Mus musculus GN=Rab30 PE=1 SV=1	13.793103	2	12	1	23.044	4.97		360000	810000	31.72
Q9DCL8	Protein phosphatase inhibitor 2 OS=Mus musculus GN=Ppp1r2 PE=1 SV=3	12.135922	1	1	1	23.105	4.83			1500000	2.73
P35290	Ras-related protein Rab-24 OS=Mus musculus GN=Rab24 PE=1 SV=2	4.4334975	1	2	1	23.129	6.23	1400000		2000000	4.24
Q8VBV7	COP9 signalosome complex subunit 8 OS=Mus musculus GN=Cops8 PE=1 SV=1	7.1770335	1	2	1	23.241	5.2		760000	770000	6.16
P60879	Synaptosomal-associated protein 25 OS=Mus musculus GN=Snap25 PE=1 SV=1	71.359223	17	105	17	23.3	4.77	73000000	59000000	84000000	355.79

Q80UU9	Membrane-associated progesterone receptor component 2 OS=Mus musculus GN=Pgrm		34.562212	4	8	3	23.32	5.15	1400000	1300000	1400000	28.03
Q9JIW9	Ras-related protein Ral-B OS=Mus musculus GN=Ralb PE=1 SV=1		4.368932	1	1	1	23.335	6.62		240000		2.33
Q9DB20	ATP synthase subunit O, mitochondrial OS=Mus musculus GN=Atp5o PE=1 SV=1		32.394366	5	14	5	23.349	9.99	5900000	6100000	5500000	41.81
Q9DD18	D-tyrosyl-tRNA(Tyr) deacylase 1 OS=Mus musculus GN=Dtd1 PE=1 SV=2		6.6985646	1	2	1	23.37	7.87	1500000	1300000		5.58
P62071	Ras-related protein R-Ras2 OS=Mus musculus GN=Rras2 PE=1 SV=1		13.72549	2	5	2	23.385	6.01	1800000	1200000	1900000	13.25
Q99PT1	Rho GDP-dissociation inhibitor 1 OS=Mus musculus GN=Arhgdia PE=1 SV=3		12.745098	2	2	2	23.393	5.2		2300000	520000	4.57
P35278	Ras-related protein Rab-5C OS=Mus musculus GN=Rab5c PE=1 SV=2		27.777778	5	12	3	23.398	8.41	6600000	3400000	12000000	33.65
P0C0A3	Charged multivesicular body protein 6 OS=Mus musculus GN=Chmp6 PE=1 SV=2		13.5	2	6	2	23.401	5.44	3800000	2000000	2600000	21.95
P61294	Ras-related protein Rab-6B OS=Mus musculus GN=Rab6b PE=1 SV=1		25.480769	5	18	2	23.447	5.53	2200000	1500000	1800000	45.15
P19253	60S ribosomal protein L13a OS=Mus musculus GN=Rpl13a PE=1 SV=4		15.270936	3	8	3	23.449	11.02	12000000	9000000	7000000	19.63
P84099	60S ribosomal protein L19 OS=Mus musculus GN=Rpl19 PE=1 SV=1		13.265306	2	7	2	23.451	11.47	28000000	9600000	14000000	22.97
P51150	Ras-related protein Rab-7a OS=Mus musculus GN=Rab7a PE=1 SV=2		47.826087	8	27	8	23.475	6.7	20000000	9800000	18000000	90.51
P53994	Ras-related protein Rab-2A OS=Mus musculus GN=Rab2a PE=1 SV=1		47.641509	8	25	8	23.533	6.54	20000000	24000000	37000000	79.54
P63321	Ras-related protein Ral-A OS=Mus musculus GN=Rala PE=1 SV=1		14.07767	3	10	3	23.538	7.11	15000000	7900000	11000000	34.93
Q9CR57	60S ribosomal protein L14 OS=Mus musculus GN=Rpl14 PE=1 SV=3		10.138249	2	9	2	23.549	11.02	14000000	4000000	6600000	26.42
Q7TQD2	Tubulin polymerization-promoting protein OS=Mus musculus GN=Tppp PE=1 SV=1		20.642202	4	11	4	23.56	9.42	12000000	7600000	12000000	30.20
P35279	Ras-related protein Rab-6A OS=Mus musculus GN=Rab6a PE=1 SV=4		23.076923	4	17	1	23.575	5.54	2700000	2400000	3200000	43.11
P34022	Ran-specific GTPase-activating protein OS=Mus musculus GN=Ranbp1 PE=1 SV=2		16.748768	1	3	1	23.582	5.22	3500000		7300000	7.46
Q8VDP6	CDP-diacylglycerol--inositol 3-phosphatidyltransferase OS=Mus musculus GN=Cdipt PE=		4.6948357	1	1	1	23.583	8.27	4200000			2.61
Q9CQD1	Ras-related protein Rab-5A OS=Mus musculus GN=Rab5a PE=1 SV=1		27.906977	5	12	3	23.584	8.15	130000	4700000		30.60
P61028	Ras-related protein Rab-8B OS=Mus musculus GN=Rab8b PE=1 SV=1		30.434783	6	20	2	23.588	9.07	600000	270000	250000	50.05
P19157	Glutathione S-transferase P 1 OS=Mus musculus GN=Gstp1 PE=1 SV=2		7.6190476	1	4	1	23.594	7.87	720000	670000	1100000	8.47
Q91ZR1	Ras-related protein Rab-4B OS=Mus musculus GN=Rab4b PE=1 SV=2		9.8591549	2	12	1	23.614	6.06		1600000	2400000	30.40
P06837	Neuromodulin OS=Mus musculus GN=Gap43 PE=1 SV=1		50.220264	10	68	10	23.618	4.73	79000000	57000000	84000000	227.04
P0C7M9	C-type lectin domain family 2 member L OS=Mus musculus GN=Clec2l PE=1 SV=1		9.9526066	2	3	2	23.638	7.2	800000		630000	6.54
Q9D7V2	LysM and putative peptidoglycan-binding domain-containing protein 2 OS=Mus musculus		5.1162791	1	2	1	23.679	5.71	1000000	420000		4.17
P61021	Ras-related protein Rab-5B OS=Mus musculus GN=Rab5b PE=1 SV=1		22.325581	5	11	3	23.692	8.13	2700000	160000	400000	29.61
P24369	Peptidyl-prolyl cis-trans isomerase B OS=Mus musculus GN=Ppib PE=1 SV=2		25.462963	5	12	5	23.699	9.55	4200000	2700000	2800000	29.97
Q3UJP5	Protein C8orf37 homolog OS=Mus musculus PE=1 SV=1		5.7416268	1	1	1	23.833	6.86			530000	2.00
P12961	Neuroendocrine protein 7B2 OS=Mus musculus GN=Scg5 PE=1 SV=1		8.490566	1	1	1	23.851	5.81		420000		4.20
Q91V41	Ras-related protein Rab-14 OS=Mus musculus GN=Rab14 PE=1 SV=3		33.488372	5	20	4	23.882	6.21	10000000	6600000	11000000	54.03
O08989	Ras-related protein M-Ras OS=Mus musculus GN=Mras PE=1 SV=1		7.6923077	1	2	1	23.886	8.78	4900000	1300000		5.28
Q8BJF9	Charged multivesicular body protein 2b OS=Mus musculus GN=Chmp2b PE=1 SV=1		7.9812207	2	5	1	23.919	8.78	2000000	840000	1900000	13.27
Q8K3J1	NADH dehydrogenase [ubiquinone] iron-sulfur protein 8, mitochondrial OS=Mus musculus		17.924528	3	14	3	24.023	6.21	9800000	6300000	11000000	40.29
P97478	5-demethoxyubiquinone hydroxylase, mitochondrial OS=Mus musculus GN=Coq7 PE=1 S		4.1474654	1	1	1	24.026	7.17	750000			2.36
P35282	Ras-related protein Rab-21 OS=Mus musculus GN=Rab21 PE=1 SV=4		22.972973	4	7	4	24.091	7.94	2900000	860000	1800000	17.66
Q9CZM2	60S ribosomal protein L15 OS=Mus musculus GN=Rpl15 PE=2 SV=4		11.27451	3	8	3	24.131	11.62	11000000	8300000	7400000	16.88
Q922J6	Tetraspanin-2 OS=Mus musculus GN=Tspan2 PE=1 SV=1		5.4298643	1	4	1	24.165	7.96	17000000	5400000	11000000	14.28
Q8BWR2	PITH domain-containing protein 1 OS=Mus musculus GN=Pithd1 PE=1 SV=1		5.2132701	1	2	1	24.177	5.74	770000	360000		4.28
P62242	40S ribosomal protein S8 OS=Mus musculus GN=Rps8 PE=1 SV=2		39.903846	7	21	7	24.19	10.32	31000000	14000000	18000000	59.67
Q56A07	Sodium channel subunit beta-2 OS=Mus musculus GN=Scn2b PE=1 SV=1		18.604651	4	11	4	24.212	6.54	10000000	6400000	13000000	29.44
P47963	60S ribosomal protein L13 OS=Mus musculus GN=Rpl13 PE=1 SV=3		24.170616	5	11	5	24.291	11.55	21000000	11000000	13000000	27.94
Q99LP6	GrpE protein homolog 1, mitochondrial OS=Mus musculus GN=Grpel1 PE=1 SV=1		5.0691244	1	2	1	24.292	8.38	1200000		1300000	6.39
Q8K386	Ras-related protein Rab-15 OS=Mus musculus GN=Rab15 PE=1 SV=1		29.245283	4	17	2	24.303	5.71	140000000	9600000	160000000	51.32
Q9CYH2	Redox-regulatory protein FAM213A OS=Mus musculus GN=Fam213a PE=1 SV=2		19.724771	4	12	4	24.379	9.17	7400000	4100000	5300000	33.94
P00761	Trypsin - Sus scrofa (Pig).	x	25.108225	4	81	4	24.394	7.18	2.4E+09	2.3E+09	3.5E+09	280.25
P62827	GTP-binding nuclear protein Ran OS=Mus musculus GN=Ran PE=1 SV=3		19.444444	4	9	4	24.408	7.49	9300000	4100000	8500000	22.76
P46638	Ras-related protein Rab-11B OS=Mus musculus GN=Rab11b PE=1 SV=3		40.825688	9	23	9	24.474	5.94	18000000	13000000	20000000	57.54

Q8R191	Synaptogyrin-3 OS=Mus musculus GN=Syngr3 PE=1 SV=1	19.650655	4	11	4	24.545	8.18	25000000	20000000	22000000	34.04
Q9D1X0	Nucleolar protein 3 OS=Mus musculus GN=Nol3 PE=1 SV=1	17.272727	2	4	2	24.552	4.07	4000000	3100000	3000000	16.83
P00493	Hypoxanthine-guanine phosphoribosyltransferase OS=Mus musculus GN=Hprt1 PE=1 SV	17.431193	3	9	3	24.555	6.68	2700000	1500000	2500000	20.71
Q6ZWV3	60S ribosomal protein L10 OS=Mus musculus GN=Rpl10 PE=1 SV=3	11.682243	2	8	2	24.588	10.08	14000000	3700000	14000000	25.19
P09671	Superoxide dismutase [Mn], mitochondrial OS=Mus musculus GN=Sod2 PE=1 SV=3	19.81982	4	9	4	24.588	8.62	16000000	5200000	15000000	28.81
P23506	Protein-L-isoaspartate(D-aspartate) O-methyltransferase OS=Mus musculus GN=Pcmt1 P	13.215859	2	4	2	24.619	7.65	4500000	4300000	8900000	10.85
P97952	Sodium channel subunit beta-1 OS=Mus musculus GN=Scn1b PE=1 SV=1	11.46789	2	6	2	24.634	4.83	3500000	2500000	8000000	13.33
Q9DC70	NADH dehydrogenase [ubiquinone] iron-sulfur protein 7, mitochondrial OS=Mus musculus	17.410714	4	19	4	24.667	9.92	9200000	5200000	7500000	53.52
O70251	Elongation factor 1-beta OS=Mus musculus GN=Eef1b PE=1 SV=5	33.333333	5	18	4	24.678	4.69	9500000	5300000	7300000	63.77
O08547	Vesicle-trafficking protein SEC22b OS=Mus musculus GN=Sec22b PE=1 SV=3	24.186047	4	13	4	24.725	8.51	7100000	1500000	3800000	37.67
Q9CZT8	Ras-related protein Rab-3B OS=Mus musculus GN=Rab3b PE=1 SV=1	34.246575	7	46	2	24.741	5.11	21000000	16000000	26000000	140.01
Q8BHK2	Sodium channel subunit beta-3 OS=Mus musculus GN=Scn3b PE=1 SV=1	9.3023256	1	3	1	24.773	4.74	1500000	1500000	1700000	11.16
Q9R0P9	Ubiquitin carboxyl-terminal hydrolase isozyme L1 OS=Mus musculus GN=Uchl1 PE=1 SV	35.426009	5	14	5	24.822	5.24	10000000	6400000	11000000	48.93
O08709	Peroxiredoxin-6 OS=Mus musculus GN=Prdx6 PE=1 SV=3	22.321429	4	13	4	24.855	6.01	2600000	1900000	3300000	36.30
P63158	High mobility group protein B1 OS=Mus musculus GN=Hmgb1 PE=1 SV=2	26.976744	3	11	3	24.878	5.74	17000000	3100000	22000000	36.66
Q9D1D4	Transmembrane emp24 domain-containing protein 10 OS=Mus musculus GN=Tmed10 P	21.004566	4	8	4	24.895	6.7	4000000	2600000	3500000	23.37
P53026	60S ribosomal protein L10a OS=Mus musculus GN=Rpl10a PE=1 SV=3	19.354839	4	8	4	24.901	9.98	8400000	4400000	4500000	17.72
Q4VAE3	Transmembrane protein 65 OS=Mus musculus GN=Tmem65 PE=1 SV=1	23.076923	5	13	5	24.902	7.78	5900000	5100000	5200000	35.74
Q9D8B3	Charged multivesicular body protein 4b OS=Mus musculus GN=Chmp4b PE=1 SV=2	15.625	3	5	3	24.921	4.82	5200000	2900000	3700000	17.09
P67871	Casein kinase II subunit beta OS=Mus musculus GN=Csnk2b PE=1 SV=1	22.790698	4	10	4	24.926	5.55	6300000	1500000	7300000	28.61
Q9CQ06	39S ribosomal protein L24, mitochondrial OS=Mus musculus GN=Mrpl24 PE=1 SV=1	3.2407407	1	1	1	24.929	9.5		300000		1.90
P70280	Vesicle-associated membrane protein 7 OS=Mus musculus GN=Vamp7 PE=1 SV=1	8.6363636	2	3	2	24.951	8.6	860000		580000	6.56
P63011	Ras-related protein Rab-3A OS=Mus musculus GN=Rab3a PE=1 SV=1	61.818182	13	70	7	24.954	5.03	150000000	120000000	200000000	205.00
O89116	Vesicle transport through interaction with t-SNAREs homolog 1A OS=Mus musculus GN=V	6.9124424	1	1	1	24.971	6.4		160000		2.87
Q9DBZ5	Eukaryotic translation initiation factor 3 subunit K OS=Mus musculus GN=Eif3k PE=1 SV=	5.0458716	1	1	1	25.07	4.93		890000		2.21
Q9DB34	Charged multivesicular body protein 2a OS=Mus musculus GN=Chmp2a PE=1 SV=1	3.6036036	1	1	1	25.118	5.97	850000			2.03
Q62446	Peptidyl-prolyl cis-trans isomerase FKBP3 OS=Mus musculus GN=Fkbp3 PE=1 SV=2	4.9107143	1	3	1	25.132	9.28	5300000	3300000	4400000	7.91
Q6IRU5	Clathrin light chain B OS=Mus musculus GN=Cltb PE=1 SV=1	16.157205	4	13	4	25.156	4.63	12000000	9100000	14000000	33.53
Q60631	Growth factor receptor-bound protein 2 OS=Mus musculus GN=Grb2 PE=1 SV=1	23.963134	4	8	4	25.222	6.32	5800000	4900000	5400000	25.20
P40240	CD9 antigen OS=Mus musculus GN=Cd9 PE=1 SV=2	3.0973451	1	3	1	25.241	7.23	16000000	4600000	9100000	6.80
Q9JKV5	Secretory carrier-associated membrane protein 4 OS=Mus musculus GN=Scamp4 PE=1 S	4.7826087	1	3	1	25.326	8.66	4600000	1200000	2100000	8.21
Q60692	Proteasome subunit beta type-6 OS=Mus musculus GN=Psmb6 PE=1 SV=3	13.02521	3	8	3	25.362	5.11	9800000	5700000	11000000	21.03
Q9D1C8	Vacuolar protein sorting-associated protein 28 homolog OS=Mus musculus GN=Vps28 PE	6.7873303	1	1	1	25.436	5.54			620000	3.72
Q8BIF0	CD99 antigen-like protein 2 OS=Mus musculus GN=Cd99l2 PE=1 SV=1	26.160338	4	10	4	25.447	4.86	2800000	3100000	3000000	33.96
Q62093	Serine/arginine-rich splicing factor 2 OS=Mus musculus GN=Srsf2 PE=1 SV=4	14.932127	4	20	4	25.461	11.85	17000000	960000	13000000	64.53
Q9JM93	ADP-ribosylation factor-like protein 6-interacting protein 4 OS=Mus musculus GN=Arl6ip4	12.227074	2	2	2	25.51	11.19	1100000			6.28
Q99MB7	RING finger protein 141 OS=Mus musculus GN=Rnf141 PE=1 SV=2	4.3478261	1	1	1	25.512	5.2			1500000	2.77
P24472	Glutathione S-transferase A4 OS=Mus musculus GN=Gsta4 PE=1 SV=3	4.5045045	1	2	1	25.547	7.39	2300000	960000		4.39
Q8BVI4	Dihydropteridine reductase OS=Mus musculus GN=Qdpr PE=1 SV=2	7.4688797	1	5	1	25.554	7.81	750000	1000000	540000	15.63
O08585	Clathrin light chain A OS=Mus musculus GN=Clta PE=1 SV=2	17.87234	5	13	5	25.588	4.58	20000000	15000000	22000000	30.20
O55100	Synaptogyrin-1 OS=Mus musculus GN=Syngr1 PE=1 SV=2	10.25641	2	8	2	25.636	4.65	61000000	45000000	61000000	26.40
P35762	CD81 antigen OS=Mus musculus GN=Cd81 PE=1 SV=2	21.186441	3	22	3	25.797	5.83	18000000	5600000	12000000	86.45
Q9JIK9	28S ribosomal protein S34, mitochondrial OS=Mus musculus GN=Mrps34 PE=1 SV=1	6.8807339	1	1	1	25.811	10.43	1000000			2.47
P62823	Ras-related protein Rab-3C OS=Mus musculus GN=Rab3c PE=1 SV=1	45.374449	10	40	5	25.856	5.24	18000000	13000000	19000000	110.89
P49722	Proteasome subunit alpha type-2 OS=Mus musculus GN=Psma2 PE=1 SV=3	15.811966	3	4	3	25.91	7.43		880000	6900000	9.58
P10649	Glutathione S-transferase Mu 1 OS=Mus musculus GN=Gstm1 PE=1 SV=2	28.440367	6	14	5	25.953	7.94	11000000	4200000	12000000	40.02
P00405	Cytochrome c oxidase subunit 2 OS=Mus musculus GN=Mtco2 PE=1 SV=1	20.264317	3	14	3	25.959	4.73	5500000	2400000	9500000	35.30
Q02105	Complement C1q subcomponent subunit C OS=Mus musculus GN=C1qc PE=1 SV=2	5.2845528	1	1	1	25.975	8.54		1700000		2.91

Q8JZS0	Protein lin-7 homolog A OS=Mus musculus GN=Lin7a PE=1 SV=2		45.064378	9	25	5	25.977	8.72	7700000	5500000	8800000	66.66
Q61189	Methylosome subunit pICln OS=Mus musculus GN=Clns1a PE=1 SV=1		5.5084746	1	1	1	26.005	4.12	1400000			2.46
Q8R1V4	Transmembrane emp24 domain-containing protein 4 OS=Mus musculus GN=Tmed4 PE=		12.334802	2	3	1	26.005	8.18		2600000	3900000	10.16
Q6PEB6	MOB-like protein phocein OS=Mus musculus GN=Mob4 PE=1 SV=1		10.222222	2	4	2	26.016	5.78	2500000	2700000		8.45
Q9JKD3	Secretory carrier-associated membrane protein 5 OS=Mus musculus GN=Scamp5 PE=1 S		8.5106383	2	10	2	26.051	8.54	36000000	26000000	41000000	26.13
Q6PGH0	Ubiquitin domain-containing protein 2 OS=Mus musculus GN=Ubtd2 PE=2 SV=1		2.991453	1	1	1	26.128	5.83	380000			1.81
P50518	V-type proton ATPase subunit E 1 OS=Mus musculus GN=Atp6v1e1 PE=1 SV=2		38.495575	10	36	10	26.141	8.43	41000000	29000000	30000000	106.01
Q62348	Translin OS=Mus musculus GN=Tsn PE=1 SV=1		6.5789474	1	2	1	26.185	6.44	7200000	1500000		6.68
Q9CQ79	Thioredoxin domain-containing protein 9 OS=Mus musculus GN=Txndc9 PE=1 SV=1		5.7522124	1	1	1	26.243	5.95			2400000	2.53
P51859	Hepatoma-derived growth factor OS=Mus musculus GN=Hdgf PE=1 SV=2		22.362869	4	7	4	26.253	4.83	3200000	1100000	1600000	18.72
Q3UBX0	Transmembrane protein 109 OS=Mus musculus GN=Tmem109 PE=1 SV=2		4.9382716	1	2	1	26.289	9.89	4300000		3300000	6.14
Q9EP72	ER membrane protein complex subunit 7 OS=Mus musculus GN=Emc7 PE=1 SV=1		9.5435685	2	2	2	26.293	9.23	1500000	970000		4.51
Q80XU3	Nuclear ubiquitous casein and cyclin-dependent kinase substrate 1 OS=Mus musculus G		11.965812	2	4	2	26.298	5.14	4500000		2700000	12.61
Q9JMG3	Transmembrane and ubiquitin-like domain-containing protein 1 OS=Mus musculus GN=Tr		4.0816327	1	1	1	26.3	5.03			930000	2.08
Q9CRB9	MICOS complex subunit Mic19 OS=Mus musculus GN=Chchd3 PE=1 SV=1		31.277533	6	12	6	26.318	8.37	9900000	9100000	22000000	40.31
O09061	Proteasome subunit beta type-1 OS=Mus musculus GN=Psmb1 PE=1 SV=1		5.8333333	1	1	1	26.355	7.81			1300000	2.39
Q9Z2U1	Proteasome subunit alpha type-5 OS=Mus musculus GN=Psma5 PE=1 SV=1		7.8838174	1	4	1	26.394	4.79	2800000	3300000	4300000	15.40
Q8BH50	Uncharacterized protein C18orf25 homolog OS=Mus musculus PE=1 SV=1		5.7142857	1	2	1	26.43	5.19	1900000		1300000	6.94
Q9CXW3	Calcyclin-binding protein OS=Mus musculus GN=Cacybp PE=1 SV=1		4.8034934	1	2	1	26.494	7.87	2700000	1500000		4.30
Q9QY36	N-alpha-acetyltransferase 10 OS=Mus musculus GN=Naa10 PE=1 SV=1		6.3829787	1	2	1	26.503	5.64		670000	1300000	5.75
P97950	Ras-related protein Rab-33A OS=Mus musculus GN=Rab33a PE=2 SV=1		8.8607595	2	2	2	26.523	7.88	3300000	560000		5.44
P50171	Estradiol 17-beta-dehydrogenase 8 OS=Mus musculus GN=Hsd17b8 PE=1 SV=2		5.019305	1	2	1	26.572	6.54	1500000		1400000	5.27
P48774	Glutathione S-transferase Mu 5 OS=Mus musculus GN=Gstm5 PE=1 SV=1		22.767857	5	11	4	26.617	7.21	3300000	1200000	3100000	28.22
P62908	40S ribosomal protein S3 OS=Mus musculus GN=Rps3 PE=1 SV=1		36.213992	8	19	8	26.657	9.66	22000000	7800000	13000000	51.62
P35288	Ras-related protein Rab-23 OS=Mus musculus GN=Rab23 PE=1 SV=2		21.940928	4	7	4	26.662	6.79	1900000	610000	1700000	19.79
O88384	Vesicle transport through interaction with t-SNAREs homolog 1B OS=Mus musculus GN=		9.4827586	2	2	2	26.697	8.79	1300000		2300000	4.74
P14106	Complement C1q subcomponent subunit B OS=Mus musculus GN=C1qb PE=1 SV=2		4.743083	1	3	1	26.701	8.15	4400000	2500000	4000000	9.22
Q8BJU2	Tetraspanin-9 OS=Mus musculus GN=Tspan9 PE=1 SV=1		2.9288703	1	1	1	26.719	7.42	1100000			2.21
Q9D8Y0	EF-hand domain-containing protein D2 OS=Mus musculus GN=Efhd2 PE=1 SV=1		20.833333	4	18	4	26.775	5.06	4500000	4500000	11000000	57.14
O08583	THO complex subunit 4 OS=Mus musculus GN=Alyref PE=1 SV=3		7.0588235	1	1	1	26.924	11.15	1100000			2.62
Q9QY76	Vesicle-associated membrane protein-associated protein B OS=Mus musculus GN=Vapb		15.63786	3	10	2	26.929	7.78	19000000	9900000	17000000	31.10
Q9QZB1	Regulator of G-protein signaling 20 OS=Mus musculus GN=Rgs20 PE=1 SV=1		6.6945607	2	3	2	26.969	5.16	1500000		1200000	5.97
Q99KF1	Transmembrane emp24 domain-containing protein 9 OS=Mus musculus GN=Tmed9 PE=		11.914894	2	3	1	27.11	8.41	3800000		9800000	10.35
P04370	Myelin basic protein OS=Mus musculus GN=Mbp PE=1 SV=2		30.4	6	45	6	27.151	9.58	190000000	51000000	120000000	121.44
Q9QXV0	ProSAAS OS=Mus musculus GN=Pcsk1n PE=1 SV=2		18.604651	3	9	3	27.254	5.85	1400000	1700000	1800000	29.88
Q9D6J6	NADH dehydrogenase [ubiquinone] flavoprotein 2, mitochondrial OS=Mus musculus GN=		16.129032	3	10	3	27.268	7.4	6600000	5000000	5900000	31.72
P35283	Ras-related protein Rab-12 OS=Mus musculus GN=Rab12 PE=1 SV=3		17.695473	4	14	3	27.311	8.41	2600000		2300000	36.54
Q9WUK2	Eukaryotic translation initiation factor 4H OS=Mus musculus GN=Eif4h PE=1 SV=3		13.709677	3	4	3	27.324	7.23	23000000		7800000	10.67
Q9QUM9	Proteasome subunit alpha type-6 OS=Mus musculus GN=Psma6 PE=1 SV=1		14.227642	3	7	3	27.355	6.76	7100000	2900000	4000000	18.90
O08756	3-hydroxyacyl-CoA dehydrogenase type-2 OS=Mus musculus GN=Hsd17b10 PE=1 SV=4		12.643678	2	5	2	27.402	8.41	1000000	480000		16.65
Q9D1F4	Proline-rich AKT1 substrate 1 OS=Mus musculus GN=Akt1s1 PE=1 SV=1		7.3929961	1	2	1	27.466	4.72	510000		670000	8.75
Q9CXS4	Centromere protein V OS=Mus musculus GN=Cenpv PE=1 SV=2		38.492063	6	10	6	27.524	9.79	11000000		7400000	29.59
Q62283	Tetraspanin-7 OS=Mus musculus GN=Tspan7 PE=1 SV=2		6.0240964	1	11	1	27.526	7.2	39000000	20000000	31000000	40.59
Q9CRD0	OCIA domain-containing protein 1 OS=Mus musculus GN=Ociad1 PE=1 SV=1		8.9068826	1	4	1	27.593	7.81	2600000	2000000	3200000	17.35
Q9DCW4	Electron transfer flavoprotein subunit beta OS=Mus musculus GN=Etfb PE=1 SV=3		8.627451	2	4	2	27.606	8.1	2200000	1200000	2200000	8.95
Q3UY34	Uncharacterized protein C12orf43 homolog OS=Mus musculus PE=2 SV=1		11.71875	2	4	2	27.614	8.7	930000		600000	11.53
Q6PDM2	Serine/arginine-rich splicing factor 1 OS=Mus musculus GN=Srsf1 PE=1 SV=3		33.064516	9	18	9	27.728	10.36	34000000		18000000	45.69
P63101	14-3-3 protein zeta/delta OS=Mus musculus GN=Ywhaz PE=1 SV=1		64.897959	18	88	15	27.754	4.79	280000000	240000000	360000000	299.89

P68254	14-3-3 protein theta OS=Mus musculus GN=Ywhaq PE=1 SV=1		56.734694	15	54	11	27.761	4.78	46000000	31000000	54000000	169.85
Q9D883	Splicing factor U2AF 35 kDa subunit OS=Mus musculus GN=U2af1 PE=1 SV=4		12.552301	3	6	3	27.797	8.81	3200000		1300000	15.12
Q9WV55	Vesicle-associated membrane protein-associated protein A OS=Mus musculus GN=Vapa		28.11245	6	18	5	27.837	8.4	14000000	4600000	14000000	44.79
Q9Z2U0	Proteasome subunit alpha type-7 OS=Mus musculus GN=Psma7 PE=1 SV=1		22.580645	5	8	5	27.838	8.46	5800000	2400000	4300000	21.87
Q9EQQ2	Protein YIPF5 OS=Mus musculus GN=Yipf5 PE=1 SV=1		4.6692607	1	3	1	27.855	4.36	1200000	390000	570000	8.22
Q61335	B-cell receptor-associated protein 31 OS=Mus musculus GN=Bcap31 PE=1 SV=4		7.755102	2	6	2	27.939	8.7	1600000	1600000	3400000	14.42
Q61334	B-cell receptor-associated protein 29 OS=Mus musculus GN=Bcap29 PE=1 SV=1		5.8333333	1	1	1	27.946	9.72	1500000			0.00
P04925	Major prion protein OS=Mus musculus GN=Prnp PE=1 SV=2		16.929134	5	20	5	27.96	9.33	3100000	4300000	3400000	47.72
P40630	Transcription factor A, mitochondrial OS=Mus musculus GN=Tfam PE=1 SV=2		7.81893	2	4	2	27.97	9.69	2500000	1300000	1700000	9.93
Q9WVJ5	Beta-crystallin B1 OS=Mus musculus GN=Crybb1 PE=1 SV=3		5.6	1	1	1	27.985	7.3		360000		2.34
P62918	60S ribosomal protein L8 OS=Mus musculus GN=Rpl8 PE=1 SV=2		16.342412	4	12	4	28.007	11.03	13000000	5600000	6800000	34.53
P97765	WW domain-binding protein 2 OS=Mus musculus GN=Wbp2 PE=1 SV=1		5.3639847	1	3	1	28.013	6.33	420000		2300000	8.19
Q99KX1	Myeloid leukemia factor 2 OS=Mus musculus GN=Mlf2 PE=1 SV=1		12.145749	2	3	2	28.037	6.98	1700000	7700000		7.93
Q80X85	28S ribosomal protein S7, mitochondrial OS=Mus musculus GN=Mrps7 PE=1 SV=1		4.9586777	1	1	1	28.045	9.94	1600000			3.40
Q9CQV8	14-3-3 protein beta/alpha OS=Mus musculus GN=Ywhab PE=1 SV=3		62.601626	15	79	9	28.069	4.83	110000000	71000000	120000000	253.17
Q9D172	ES1 protein homolog, mitochondrial OS=Mus musculus GN=D10Jhu81e PE=1 SV=1		3.7593985	1	1	1	28.073	8.78			3300000	2.20
P20108	Thioredoxin-dependent peroxide reductase, mitochondrial OS=Mus musculus GN=Prdx3		3.1128405	1	1	1	28.109	7.58	5000000			2.52
Q9CQE8	UPF0568 protein C14orf166 homolog OS=Mus musculus PE=1 SV=1		14.344262	3	5	3	28.135	6.89	3300000	1700000	2500000	13.36
P68510	14-3-3 protein eta OS=Mus musculus GN=Ywhah PE=1 SV=2		64.634146	15	56	11	28.194	4.89	69000000	60000000	88000000	181.19
Q8BXV2	BRI3-binding protein OS=Mus musculus GN=Bri3bp PE=1 SV=1		9.0909091	2	6	2	28.245	9.52	4500000	1000000	2100000	15.77
Q61885	Myelin-oligodendrocyte glycoprotein OS=Mus musculus GN=Mog PE=1 SV=1		7.7235772	2	4	2	28.253	7.96	350000	11000000	25000000	9.90
P61982	14-3-3 protein gamma OS=Mus musculus GN=Ywhag PE=1 SV=2		56.680162	14	87	8	28.285	4.89	160000000	99000000	190000000	261.49
Q8CCM6	Mitochondrial import inner membrane translocase subunit Tim21 OS=Mus musculus GN=		4.0160643	1	1	1	28.286	10.3			1200000	2.35
P57746	V-type proton ATPase subunit D OS=Mus musculus GN=Atp6v1d PE=1 SV=1		28.744939	6	21	6	28.351	9.45	17000000	7800000	14000000	69.15
Q8QZT2	Centriole, cilia and spindle-associated protein OS=Mus musculus GN=Ccsap PE=1 SV=1		15.079365	3	7	3	28.361	9.22	2500000	1200000	4700000	16.91
O70435	Proteasome subunit alpha type-3 OS=Mus musculus GN=Psma3 PE=1 SV=3		12.156863	3	9	3	28.387	5.44	7700000	4200000	7000000	23.05
Q8VCD6	Receptor expression-enhancing protein 2 OS=Mus musculus GN=Reep2 PE=1 SV=2		13.385827	3	5	3	28.419	9.41	1700000		2900000	12.27
O88456	Calpain small subunit 1 OS=Mus musculus GN=Capns1 PE=1 SV=1		11.895911	3	7	3	28.445	5.63	2900000	4000000	4700000	21.42
Q6IRU2	Tropomyosin alpha-4 chain OS=Mus musculus GN=Tpm4 PE=1 SV=3		21.370968	5	13	3	28.45	4.68	2100000	1500000	3000000	37.25
O55234	Proteasome subunit beta type-5 OS=Mus musculus GN=Psmb5 PE=1 SV=3		12.878788	3	9	3	28.514	7.02	8700000	3000000	3000000	28.21
O35381	Acidic leucine-rich nuclear phosphoprotein 32 family member A OS=Mus musculus GN=A		19.838057	5	13	2	28.52	4.07	18000000	7600000	18000000	31.75
P62754	40S ribosomal protein S6 OS=Mus musculus GN=Rps6 PE=1 SV=1		14.457831	3	14	3	28.663	10.84	25000000	5400000	10000000	42.81
Q9QYB1	Chloride intracellular channel protein 4 OS=Mus musculus GN=Clic4 PE=1 SV=3		6.3241107	1	1	1	28.711	5.59			550000	3.71
Q6PHZ8	Kv channel-interacting protein 4 OS=Mus musculus GN=Kcnip4 PE=1 SV=1		11.2	2	2	2	28.737	5.21		400000	2400000	2.49
P57759	Endoplasmic reticulum resident protein 29 OS=Mus musculus GN=Erp29 PE=1 SV=2		6.870229	2	5	2	28.805	6.15	1400000	330000	1100000	11.03
Q9DBJ1	Phosphoglycerate mutase 1 OS=Mus musculus GN=Pgam1 PE=1 SV=3		35.433071	6	23	6	28.814	7.18	12000000	5500000	11000000	71.70
Q9ESJ7	Interleukin-1 receptor-associated kinase 1-binding protein 1 OS=Mus musculus GN=Irak1		4.6332046	1	2	1	28.843	8.32	2600000		1600000	4.25
P13717	SWISS-PROT:P13717 Nuclease - Serratia marcescens.	x	24.81203	5	14	5	28.927	7.42	3200000	2300000	4000000	41.20
Q9CQQ7	ATP synthase F(0) complex subunit B1, mitochondrial OS=Mus musculus GN=Atp5f1 PE=		19.921875	8	30	8	28.93	9.06	59000000	26000000	71000000	86.18
Q8BIG7	Catechol O-methyltransferase domain-containing protein 1 OS=Mus musculus GN=Comtd		8.0152672	2	4	2	28.943	8.32	2600000	1300000	2200000	10.93
Q9JKK1	Syntaxin-6 OS=Mus musculus GN=Stx6 PE=1 SV=1		7.4509804	1	2	1	28.979	4.92		450000	730000	8.83
Q8BQP9	Regulator of G-protein signaling 7-binding protein OS=Mus musculus GN=Rgs7bp PE=1 S		9.7276265	2	3	2	29.005	8.46	1300000		820000	8.37
P00920	Carbonic anhydrase 2 OS=Mus musculus GN=Ca2 PE=1 SV=4		17.307692	3	7	3	29.015	7.01	3400000	1500000	3300000	18.43
P99026	Proteasome subunit beta type-4 OS=Mus musculus GN=Psmb4 PE=1 SV=1		12.121212	2	4	2	29.097	5.64	8200000	6100000	6100000	10.10
P62259	14-3-3 protein epsilon OS=Mus musculus GN=Ywhae PE=1 SV=1		65.098039	17	117	15	29.155	4.74	110000000	96000000	140000000	353.63
O70152	Dolichol-phosphate mannosyltransferase subunit 1 OS=Mus musculus GN=Dpm1 PE=1 S		5.3846154	1	3	1	29.156	9.51	2000000	680000	830000	10.15
Q91XR9	Phospholipid hydroperoxide glutathione peroxidase, nuclear OS=Mus musculus GN=Gpx4		17.391304	4	10	4	29.234	10.2	3000000	720000	3100000	27.09
Q78IK4	MICOS complex subunit Mic27 OS=Mus musculus GN=Apool PE=1 SV=1		5.6603774	1	1	1	29.242	9.31		2400000		3.15

Q8K2J7	RELT-like protein 1 OS=Mus musculus GN=Rell1 PE=2 SV=2	3.3088235	1	1	1	29.333	7.85	830000			1.97
Q9CR68	Cytochrome b-c1 complex subunit Rieske, mitochondrial OS=Mus musculus GN=Uqcrfs1	15.328467	8	23	8	29.349	8.7	66000000	39000000	68000000	62.53
Q5M956	BTB/POZ domain-containing protein KCTD1 OS=Mus musculus GN=Kctd1 PE=1 SV=1	12.062257	1	1	1	29.386	7.08		710000		0.00
Q8BGX2	Uncharacterized protein C19orf52 homolog OS=Mus musculus PE=1 SV=1	11.278195	2	4	2	29.397	6.73	2100000	1600000	2200000	15.40
O08579	Emerin OS=Mus musculus GN=Emd PE=1 SV=1	19.305019	4	11	4	29.417	5.01	4100000	3800000	5200000	34.06
Q9DC71	28S ribosomal protein S15, mitochondrial OS=Mus musculus GN=Mrps15 PE=1 SV=2	5.8139535	1	1	1	29.445	10.13		1600000		3.28
Q9R1P0	Proteasome subunit alpha type-4 OS=Mus musculus GN=Psma4 PE=1 SV=1	6.8965517	2	6	2	29.452	7.72	4100000	2200000	3700000	14.99
O88587	Catechol O-methyltransferase OS=Mus musculus GN=Comt PE=1 SV=2	3.7735849	1	1	1	29.467	5.83	1500000			2.69
Q66JS6	Eukaryotic translation initiation factor 3 subunit J-B OS=Mus musculus GN=Eif3j2 PE=1 S	19.771863	4	10	4	29.468	4.81	5100000	4000000	7400000	30.75
Q9R1P4	Proteasome subunit alpha type-1 OS=Mus musculus GN=Psma1 PE=1 SV=1	25.475285	6	13	6	29.528	6.46	8300000	5500000	3700000	37.38
P62702	40S ribosomal protein S4, X isoform OS=Mus musculus GN=Rps4x PE=1 SV=2	31.558935	7	15	7	29.579	10.15	14000000	5800000	8800000	43.23
P97822	Acidic leucine-rich nuclear phosphoprotein 32 family member E OS=Mus musculus GN=A	11.923077	1	1	1	29.604	3.88				2.24
Q9CR95	Adaptin ear-binding coat-associated protein 1 OS=Mus musculus GN=Necap1 PE=1 SV=	6.5454545	2	5	2	29.621	6.38	5000000	4100000	7800000	10.61
P26645	Myristoylated alanine-rich C-kinase substrate OS=Mus musculus GN=Marcks PE=1 SV=2	67.313916	12	71	12	29.644	4.34	140000000	57000000	140000000	334.23
Q6P1B3	PILR alpha-associated neural protein OS=Mus musculus GN=Pianp PE=1 SV=1	3.2374101	1	1	1	29.725	8.72		480000		2.19
O88441	Metaxin-2 OS=Mus musculus GN=Mtx2 PE=1 SV=1	9.1254753	2	5	2	29.739	5.63	1500000	1300000	1800000	16.77
O88696	ATP-dependent Clp protease proteolytic subunit, mitochondrial OS=Mus musculus GN=C	6.6176471	1	3	1	29.781	7.47	1900000	2700000		8.30
Q6NZB0	DnaJ homolog subfamily C member 8 OS=Mus musculus GN=Dnajc8 PE=1 SV=2	3.1620553	1	1	1	29.794	9.06			2000000	1.93
O70439	Syntaxin-7 OS=Mus musculus GN=Stx7 PE=1 SV=3	11.111111	2	4	2	29.802	5.78	3700000	1900000	1400000	11.67
P67778	Prohibitin OS=Mus musculus GN=Phb PE=1 SV=1	15.073529	4	11	4	29.802	5.76	16000000	13000000	18000000	29.10
Q91VN4	MICOS complex subunit Mic25 OS=Mus musculus GN=Chchd6 PE=1 SV=2	13.919414	3	9	3	29.833	8.41	5300000	3600000	7100000	32.56
P97351	40S ribosomal protein S3a OS=Mus musculus GN=Rps3a PE=1 SV=3	22.727273	6	21	6	29.866	9.73	14000000	7300000	10000000	51.64
P70195	Proteasome subunit beta type-7 OS=Mus musculus GN=Psmb7 PE=1 SV=1	10.830325	3	6	3	29.872	7.99	3300000	2300000	4400000	15.25
Q9ERQ8	Carbonic anhydrase 7 OS=Mus musculus GN=Ca7 PE=1 SV=2	4.1666667	1	1	1	29.896	6.95			390000	2.80
P00416	Cytochrome c oxidase subunit 3 OS=Mus musculus GN=mt-Co3 PE=1 SV=2	5.3639847	1	7	1	29.903	7.3	17000000	12000000	19000000	24.35
P12970	60S ribosomal protein L7a OS=Mus musculus GN=Rpl7a PE=1 SV=2	30.451128	8	17	8	29.958	10.56	23000000	8700000	14000000	46.45
P12658	Calbindin OS=Mus musculus GN=Calb1 PE=1 SV=2	4.5977011	1	2	1	29.975	4.83	1000000	540000		5.97
Q61792	LIM and SH3 domain protein 1 OS=Mus musculus GN=Lasp1 PE=1 SV=1	25.475285	7	21	7	29.975	7.05	7500000	6200000	7800000	60.74
Q61166	Microtubule-associated protein RP/EB family member 1 OS=Mus musculus GN=Mapre1 P	13.432836	3	7	2	29.997	5.22	2400000	1300000	1900000	16.75
Q8BZJ7	DCN1-like protein 2 OS=Mus musculus GN=Dcun1d2 PE=1 SV=3	6.5637066	1	1	1	30.049	5.59			300000	2.22
P60202	Myelin proteolipid protein OS=Mus musculus GN=Plp1 PE=1 SV=2	18.772563	5	23	5	30.057	8.35	130000000	50000000	98000000	58.42
Q9QZ73	DCN1-like protein 1 OS=Mus musculus GN=Dcun1d1 PE=1 SV=1	6.5637066	1	1	1	30.078	5.34		180000		1.89
Q9DCT2	NADH dehydrogenase [ubiquinone] iron-sulfur protein 3, mitochondrial OS=Mus musculus	30.798479	8	22	8	30.131	7.17	21000000	17000000	21000000	72.18
Q6PAM0	5'-AMP-activated protein kinase subunit beta-2 OS=Mus musculus GN=Prkab2 PE=1 SV=	6.6420664	1	2	1	30.19	6.46		490000	1000000	7.05
Q9CZ04	COP9 signalosome complex subunit 7a OS=Mus musculus GN=Cops7a PE=1 SV=2	4.3636364	1	3	1	30.206	7.87	2100000	900000	1700000	8.03
O89051	Integral membrane protein 2B OS=Mus musculus GN=Itm2b PE=1 SV=1	3.3834586	1	1	1	30.24	5.3		1600000		2.40
P62080	Tetraspanin-5 OS=Mus musculus GN=Tspan5 PE=2 SV=1	5.9701493	1	2	1	30.317	4.78	450000			4.87
Q9QZ06	Toll-interacting protein OS=Mus musculus GN=Tollip PE=1 SV=1	5.1094891	1	3	1	30.325	5.17	2200000	1300000	2100000	11.64
O55023	Inositol monophosphatase 1 OS=Mus musculus GN=Impa1 PE=1 SV=1	3.9711191	1	3	1	30.416	5.19	1700000	1700000	2400000	6.06
Q9CX86	Heterogeneous nuclear ribonucleoprotein A0 OS=Mus musculus GN=Hnrnpa0 PE=1 SV=	6.557377	1	3	1	30.512	9.31	9800000	2000000	7200000	12.21
Q9DAU1	Protein canopy homolog 3 OS=Mus musculus GN=Cnpy3 PE=1 SV=1	6.884058	1	1	1	30.519	5.62			480000	4.19
Q91VJ5	Polyglutamine-binding protein 1 OS=Mus musculus GN=Pqbp1 PE=1 SV=1	9.8859316	1	1	1	30.579	6.23			1500000	5.56
Q9QYI5	DnaJ homolog subfamily B member 2 OS=Mus musculus GN=Dnajb2 PE=1 SV=2	12.274368	3	9	2	30.596	4.98	3800000	1800000	3800000	26.15
P48758	Carbonyl reductase [NADPH] 1 OS=Mus musculus GN=Cbr1 PE=1 SV=3	19.494585	3	6	3	30.622	8.31	3700000		4300000	19.63
Q60931	Voltage-dependent anion-selective channel protein 3 OS=Mus musculus GN=Vdac3 PE=	21.908127	6	19	5	30.733	8.79	23000000	19000000	21000000	57.15
Q8BL97	Serine/arginine-rich splicing factor 7 OS=Mus musculus GN=Srsf7 PE=1 SV=1	12.359551	3	8	2	30.799	11.9	6400000		5600000	22.54
Q99020	Heterogeneous nuclear ribonucleoprotein A/B OS=Mus musculus GN=Hnrnpab PE=1 SV	12.982456	3	11	2	30.812	7.91	12000000	4700000	9300000	33.43
Q9D061	Acyl-CoA-binding domain-containing protein 6 OS=Mus musculus GN=Acbd6 PE=1 SV=2	4.964539	1	1	1	30.868	5.11			750000	2.46

O35326	Serine/arginine-rich splicing factor 5 OS=Mus musculus GN=Srsf5 PE=1 SV=2	25.27881	6	11	5	30.873	11.56	7200000		4600000	32.85
Q9JJ69	Kv channel-interacting protein 2 OS=Mus musculus GN=Kcnip2 PE=1 SV=2	5.1851852	1	1	1	30.926	5.05			720000	2.24
O35658	Complement component 1 Q subcomponent-binding protein, mitochondrial OS=Mus musc	15.107914	4	14	4	30.994	4.92	10000000	8700000	17000000	48.70
Q9EST5	Acidic leucine-rich nuclear phosphoprotein 32 family member B OS=Mus musculus GN=A	13.602941	4	10	1	31.06	4.01	1900000	4800000	11000000	23.57
Q9DC07	LIM zinc-binding domain-containing Nebulette OS=Mus musculus GN=Nebl PE=1 SV=1	15.185185	3	4	3	31.093	8.31	3800000	2400000	220000	12.22
P35802	Neuronal membrane glycoprotein M6-a OS=Mus musculus GN=Gpm6a PE=1 SV=1	13.309353	5	26	5	31.128	5.27	140000000	90000000	170000000	89.32
P24668	Cation-dependent mannose-6-phosphate receptor OS=Mus musculus GN=M6pr PE=1 SV	18.345324	5	9	5	31.152	5.39	7800000	3500000	2800000	26.02
Q80X71	Transmembrane protein 106B OS=Mus musculus GN=Tmem106b PE=1 SV=1	4.7272727	1	1	1	31.153	6.68				2.08
Q8C996	Transmembrane protein 163 OS=Mus musculus GN=Tmem163 PE=1 SV=1	9.0277778	1	2	1	31.173	7.93	550000			6.40
Q9ER00	Syntaxin-12 OS=Mus musculus GN=Stx12 PE=1 SV=1	8.7591241	2	6	2	31.176	5.44	4800000	2200000	3200000	18.80
P25444	40S ribosomal protein S2 OS=Mus musculus GN=Rps2 PE=1 SV=3	32.423208	9	18	9	31.212	10.24	10000000	4700000	7000000	43.57
Q9D7A8	Armadillo repeat-containing protein 1 OS=Mus musculus GN=Armc1 PE=1 SV=1	9.2198582	2	4	2	31.227	5.57	1600000	1100000	1500000	12.24
O54901	OX-2 membrane glycoprotein OS=Mus musculus GN=Cd200 PE=1 SV=1	8.6330935	2	8	2	31.236	8.79	9000000	6400000	7600000	20.97
Q7TQI3	Ubiquitin thioesterase OTUB1 OS=Mus musculus GN=Otub1 PE=1 SV=2	16.605166	3	7	3	31.25	4.94	5100000	870000	3900000	23.10
P57776	Elongation factor 1-delta OS=Mus musculus GN=Eef1d PE=1 SV=3	30.604982	6	14	5	31.274	5.02	6000000	2500000	4500000	39.87
Q9R0P3	S-formylglutathione hydrolase OS=Mus musculus GN=Esd PE=1 SV=1	4.2553191	1	2	1	31.299	7.12	3000000		2200000	4.80
P47757	F-actin-capping protein subunit beta OS=Mus musculus GN=Capzb PE=1 SV=3	35.379061	8	22	8	31.326	5.74	11000000	5500000	9400000	59.32
Q08331	Calretinin OS=Mus musculus GN=Calb2 PE=1 SV=3	9.9630996	2	4	2	31.353	5.02	1300000	360000	910000	11.20
Q9CWS0	N(G),N(G)-dimethylarginine dimethylaminohydrolase 1 OS=Mus musculus GN=Ddah1 PE	29.824561	7	19	7	31.361	5.97	11000000	3800000	12000000	56.71
O35449	Proline-rich transmembrane protein 1 OS=Mus musculus GN=Prrt1 PE=1 SV=1	3.5947712	1	1	1	31.369	7.65		1000000		2.40
Q8VBT0	Thioredoxin-related transmembrane protein 1 OS=Mus musculus GN=Tmx1 PE=1 SV=1	6.8345324	2	2	2	31.376	5.29			1500000	4.93
P14148	60S ribosomal protein L7 OS=Mus musculus GN=Rpl7 PE=1 SV=2	24.074074	8	20	8	31.4	10.89	21000000	9100000	8800000	57.29
Q8BH95	Enoyl-CoA hydratase, mitochondrial OS=Mus musculus GN=Echs1 PE=1 SV=1	17.241379	4	6	4	31.454	8.48	2400000	1600000	2200000	17.52
O35083	1-acyl-sn-glycerol-3-phosphate acyltransferase alpha OS=Mus musculus GN=Agpat1 PE=	8.0701754	2	5	2	31.689	9.14	2400000	830000	1200000	12.99
Q9D387	Lysosome-associated membrane glycoprotein 5 OS=Mus musculus GN=Lamp5 PE=1 SV	3.5714286	1	3	1	31.701	6.14	4700000	2900000	4800000	9.52
Q9CWE0	Mitochondrial fission regulator 1-like OS=Mus musculus GN=Mtfr1l PE=1 SV=1	14.186851	3	8	3	31.706	6.1	2100000	1100000	2500000	21.76
Q60930	Voltage-dependent anion-selective channel protein 2 OS=Mus musculus GN=Vdac2 PE=	32.20339	7	27	7	31.713	7.49	85000000	64000000	85000000	89.99
Q9CQA3	Succinate dehydrogenase [ubiquinone] iron-sulfur subunit, mitochondrial OS=Mus muscul	24.113475	7	16	7	31.793	8.68	17000000	4800000	13000000	42.32
P97447	Four and a half LIM domains protein 1 OS=Mus musculus GN=Fhl1 PE=1 SV=3	4.2857143	1	3	1	31.867	8.37	1800000	460000	1300000	8.40
P53810	Phosphatidylinositol transfer protein alpha isoform OS=Mus musculus GN=Pitpna PE=1 S	18.081181	4	6	4	31.873	6.37	5700000	2200000	5900000	21.31
Q6PER3	Microtubule-associated protein RP/EB family member 3 OS=Mus musculus GN=Mapre3 F	15.658363	4	14	3	31.946	5.54	10000000	4800000	7000000	37.17
Q8BX10	Serine/threonine-protein phosphatase PGAM5, mitochondrial OS=Mus musculus GN=Pga	3.125	1	1	1	31.975	9.04		470000		2.42
Q9Z1R4	Uncharacterized protein C6orf47 homolog OS=Mus musculus GN=D17h6s53e PE=1 SV=	4.0955631	1	1	1	31.995	6.89	2200000			1.93
Q9CY50	Translocon-associated protein subunit alpha OS=Mus musculus GN=Ssr1 PE=1 SV=1	11.888112	2	8	2	32.045	4.45	7400000	2000000	4300000	24.10
O70433	Four and a half LIM domains protein 2 OS=Mus musculus GN=Fhl2 PE=1 SV=1	11.469534	2	4	2	32.051	7.3		720000	1300000	11.61
Q3TDK6	Protein rogdi homolog OS=Mus musculus GN=Rogdi PE=1 SV=2	5.2264808	1	3	1	32.08	8.18	3500000	2100000	3100000	12.34
Q5EBJ4	Ermin OS=Mus musculus GN=Ermn PE=1 SV=1	4.9822064	1	3	1	32.128	4.59	2500000	1800000	3700000	11.15
O08600	Endonuclease G, mitochondrial OS=Mus musculus GN=Endog PE=1 SV=1	5.7823129	1	1	1	32.171	9.54				2.85
P17751	Triosephosphate isomerase OS=Mus musculus GN=Tpi1 PE=1 SV=4	30.434783	7	27	7	32.171	5.74	8400000	5300000	10000000	84.96
Q8CDN6	Thioredoxin-like protein 1 OS=Mus musculus GN=Txnl1 PE=1 SV=3	11.072664	2	5	2	32.217	4.96	2100000	1800000	2500000	16.01
Q8CCS6	Polyadenylate-binding protein 2 OS=Mus musculus GN=Pabpn1 PE=1 SV=3	3.6423841	1	3	1	32.277	5.17	2000000	810000	1200000	9.15
Q924T2	28S ribosomal protein S2, mitochondrial OS=Mus musculus GN=Mrps2 PE=1 SV=1	7.2164948	1	1	1	32.293	9.14	600000			2.75
Q6ZQI3	Malectin OS=Mus musculus GN=Mlec PE=1 SV=2	24.742268	6	15	6	32.322	6.05	4200000	2100000	3300000	44.30
Q60932	Voltage-dependent anion-selective channel protein 1 OS=Mus musculus GN=Vdac1 PE=	52.702703	15	73	14	32.331	8.43	220000000	180000000	250000000	257.04
Q8BTF8	RNA-binding Raly-like protein OS=Mus musculus GN=Ralyl PE=2 SV=1	3.0716724	1	1	1	32.432	7.93	1200000			2.43
P10923	Osteopontin OS=Mus musculus GN=Spp1 PE=1 SV=1	3.0612245	1	1	1	32.44	4.53	2900000			2.80
B1AXV0	DOMON domain-containing protein FRRS1L OS=Mus musculus GN=Frrs1l PE=1 SV=1	12.627986	3	13	3	32.486	5.43	4500000	4100000	5500000	32.98
Q61937	Nucleophosmin OS=Mus musculus GN=Npm1 PE=1 SV=1	23.630137	5	11	5	32.54	4.77	18000000		20000000	42.54

Q9DC16	Endoplasmic reticulum-Golgi intermediate compartment protein 1 OS=Mus musculus GN=		3.1034483	1	1	1	32.541	7.06	3500000			1.94
P58771	Tropomyosin alpha-1 chain OS=Mus musculus GN=Tpm1 PE=1 SV=1		29.225352	9	31	4	32.661	4.74	29000000	17000000	37000000	92.16
P58774	Tropomyosin beta chain OS=Mus musculus GN=Tpm2 PE=1 SV=1	x	15.84507	6	14	1	32.817	4.7	1300000	450000	1100000	41.45
P14206	40S ribosomal protein SA OS=Mus musculus GN=Rpsa PE=1 SV=4		20.677966	4	12	4	32.817	4.87	4100000	4000000	5000000	38.30
Q8R164	Valacyclovir hydrolase OS=Mus musculus GN=Bphl PE=1 SV=1		4.467354	1	1	1	32.83	8.94		470000		2.49
Q9EQ06	Estradiol 17-beta-dehydrogenase 11 OS=Mus musculus GN=Hsd17b11 PE=1 SV=1		3.6912752	1	1	1	32.86	8.66	1800000			2.68
Q91VR2	ATP synthase subunit gamma, mitochondrial OS=Mus musculus GN=Atp5c1 PE=1 SV=1		29.530201	8	25	8	32.865	9.01	49000000	33000000	44000000	67.65
P48962	ADP/ATP translocase 1 OS=Mus musculus GN=Slc25a4 PE=1 SV=4		42.61745	13	44	8	32.883	9.72	220000000	76000000	150000000	109.52
Q8R088	Golgi phosphoprotein 3-like OS=Mus musculus GN=Golph3l PE=1 SV=1		5.2631579	1	2	1	32.885	5.99		230000	440000	5.18
Q9QZE7	Translin-associated protein X OS=Mus musculus GN=Tsnax PE=1 SV=1		5.862069	1	3	1	32.906	6.55	1200000	770000	1100000	10.13
P51881	ADP/ATP translocase 2 OS=Mus musculus GN=Slc25a5 PE=1 SV=3		34.563758	11	33	6	32.91	9.73	49000000	12000000	30000000	78.47
Q6PCP5	Mitochondrial fission factor OS=Mus musculus GN=Mff PE=1 SV=1		7.5601375	2	4	2	32.911	6.83	5900000	2600000	6200000	11.22
O55126	Protein NipSnap homolog 2 OS=Mus musculus GN=Gbas PE=1 SV=1		4.9822064	1	1	1	32.912	9.26			1300000	2.31
P20352	Tissue factor OS=Mus musculus GN=F3 PE=1 SV=2		8.1632653	2	3	2	32.914	9.32	910000	610000		9.15
P47753	F-actin-capping protein subunit alpha-1 OS=Mus musculus GN=Capza1 PE=1 SV=4		5.2447552	1	2	1	32.919	5.55		2000000	2600000	7.00
P47754	F-actin-capping protein subunit alpha-2 OS=Mus musculus GN=Capza2 PE=1 SV=3		33.216783	5	10	5	32.947	5.85	10000000	6300000	12000000	37.54
Q8BHE8	Uncharacterized protein C2orf47 homolog, mitochondrial OS=Mus musculus PE=1 SV=1		9.9656357	3	7	2	32.964	9.14	860000	2700000	840000	16.42
P21107	Tropomyosin alpha-3 chain OS=Mus musculus GN=Tpm3 PE=1 SV=3		31.929825	10	32	4	32.974	4.72	33000000	18000000	42000000	90.90
Q8CAK3	Repressor of yield of DENV protein homolog OS=Mus musculus GN=Ryden PE=2 SV=1		12.413793	2	2	2	32.994	7.28	430000			6.04
Q9Z2Z6	Mitochondrial carnitine/acylcarnitine carrier protein OS=Mus musculus GN=Slc25a20 PE=		2.6578073	1	3	1	33.005	9.11	1400000	720000	830000	6.46
Q6NVE9	Protein phosphatase PTC7 homolog OS=Mus musculus GN=Pptc7 PE=1 SV=1		4.1935484	1	1	1	33.027	5.27			830000	2.34
O35526	Syntaxin-1A OS=Mus musculus GN=Stx1a PE=1 SV=3		51.041667	14	72	13	33.034	5.24	110000000	43000000	91000000	227.22
Q9DCU6	39S ribosomal protein L4, mitochondrial OS=Mus musculus GN=Mrpl4 PE=1 SV=1		3.7414966	1	1	1	33.052	9.82	930000			2.14
Q61735	Leukocyte surface antigen CD47 OS=Mus musculus GN=Cd47 PE=1 SV=2		10.891089	3	13	3	33.076	8.63	25000000	11000000	22000000	36.02
Q64012	RNA-binding protein Raly OS=Mus musculus GN=Raly PE=1 SV=3		16.346154	4	5	4	33.168	8.84	2500000		1700000	13.39
Q9DB05	Alpha-soluble NSF attachment protein OS=Mus musculus GN=Napa PE=1 SV=1		43.050847	11	31	10	33.168	5.45	15000000	9800000	18000000	97.57
P61264	Syntaxin-1B OS=Mus musculus GN=Stx1b PE=1 SV=1		58.680556	18	87	17	33.224	5.38	190000000	64000000	140000000	296.83
Q91Z38	Tetratricopeptide repeat protein 1 OS=Mus musculus GN=Ttc1 PE=1 SV=1		9.2465753	2	4	2	33.242	5.01	1000000	530000	1300000	10.96
Q3V2J0	Protein FAM92B OS=Mus musculus GN=Fam92b PE=2 SV=2		2.3972603	1	1	1	33.254	6.11				1.81
P49615	Cyclin-dependent-like kinase 5 OS=Mus musculus GN=Cdk5 PE=1 SV=1		17.808219	4	7	4	33.267	7.66	3800000	1500000	2700000	21.09
O35129	Prohibitin-2 OS=Mus musculus GN=Phb2 PE=1 SV=1		27.424749	8	18	8	33.276	9.83	19000000	11000000	12000000	49.05
Q9D0L7	Armadillo repeat-containing protein 10 OS=Mus musculus GN=Armc10 PE=1 SV=1		5.2287582	1	2	1	33.29	7.99	3400000		2200000	7.67
Q9CPV4	Glyoxalase domain-containing protein 4 OS=Mus musculus GN=Glod4 PE=1 SV=1		5.033557	1	1	1	33.296	5.47		700000		3.10
P14231	Sodium/potassium-transporting ATPase subunit beta-2 OS=Mus musculus GN=Atp1b2 PI		33.103448	8	21	8	33.322	8.31	49000000	25000000	43000000	67.77
O55125	Protein NipSnap homolog 1 OS=Mus musculus GN=Nipsnap1 PE=1 SV=1		11.267606	3	8	3	33.342	9.44	7500000	7100000	6200000	22.17
Q9EQU5	Protein SET OS=Mus musculus GN=Set PE=1 SV=1		17.99308	5	12	5	33.358	4.32	16000000	5800000	15000000	41.98
O35678	Monoglyceride lipase OS=Mus musculus GN=Mgll PE=1 SV=1		10.891089	3	6	3	33.366	7.15	3900000	2300000	4000000	16.34
Q9JLZ3	Methylglutaconyl-CoA hydratase, mitochondrial OS=Mus musculus GN=Auh PE=1 SV=1		11.783439	3	6	3	33.374	9.51	2500000	1700000	970000	14.79
Q8WTY4	Anamorsin OS=Mus musculus GN=Ciapin1 PE=1 SV=1		3.8834951	1	2	1	33.408	5.2	940000		820000	5.04
Q8VD33	Small glutamine-rich tetratricopeptide repeat-containing protein beta OS=Mus musculus G		9.2105263	2	4	2	33.408	4.92	2900000	2000000	3600000	8.78
Q791V5	Mitochondrial carrier homolog 2 OS=Mus musculus GN=Mtch2 PE=1 SV=1		17.491749	5	14	5	33.477	8.25	9200000	4100000	5500000	35.74
P47911	60S ribosomal protein L6 OS=Mus musculus GN=Rpl6 PE=1 SV=3		17.567568	9	15	9	33.489	10.7	19000000	3400000	9700000	31.67
O54983	Ketimine reductase mu-crystallin OS=Mus musculus GN=Crym PE=1 SV=1		3.514377	1	1	1	33.502	5.67	1300000			2.26
Q9CY64	Biliverdin reductase A OS=Mus musculus GN=Blvra PE=1 SV=1		4.7457627	1	2	1	33.504	7.02	220000		890000	5.85
P28663	Beta-soluble NSF attachment protein OS=Mus musculus GN=Napb PE=1 SV=2		47.986577	13	53	12	33.535	5.47	47000000	26000000	47000000	181.92
Q9Z130	Heterogeneous nuclear ribonucleoprotein D-like OS=Mus musculus GN=Hnrnpdl PE=1 SV		15.946844	4	13	3	33.538	7.31	30000000	9400000	16000000	40.17
Q922Y1	UBX domain-containing protein 1 OS=Mus musculus GN=Ubxn1 PE=1 SV=1		9.7643098	1	1	1	33.552	5.26	1000000			3.40
Q922Q4	Pyrroline-5-carboxylate reductase 2 OS=Mus musculus GN=Pycr2 PE=1 SV=1		5	1	1	1	33.638	7.77		510000		2.74

P62996	Transformer-2 protein homolog beta OS=Mus musculus GN=Tra2b PE=1 SV=1	13.888889	4	7	4	33.646	11.25	2700000		3800000	16.53
Q80UW2	F-box only protein 2 OS=Mus musculus GN=Fbxo2 PE=1 SV=1	36.363636	7	19	7	33.655	4.28	7000000	5000000	11000000	65.92
Q5HZI9	Solute carrier family 25 member 51 OS=Mus musculus GN=Slc25a51 PE=1 SV=1	2.3489933	1	3	1	33.668	9.66	1800000	570000	1600000	6.60
Q8K2H2	OTU domain-containing protein 6B OS=Mus musculus GN=Otud6b PE=1 SV=1	4.4217687	1	1	1	33.737	5.53			1200000	3.51
Q9ERF3	WD repeat-containing protein 61 OS=Mus musculus GN=Wdr61 PE=1 SV=1	5.2459016	1	1	1	33.752	5.36		250000		3.04
P47941	Crk-like protein OS=Mus musculus GN=Crkl PE=1 SV=2	5.2805281	1	1	1	33.809	6.74			1600000	2.39
P10711	Transcription elongation factor A protein 1 OS=Mus musculus GN=Tcea1 PE=1 SV=2	4.3189369	1	1	1	33.859	8.38	950000			2.04
O35295	Transcriptional activator protein Pur-beta OS=Mus musculus GN=Purb PE=1 SV=3	12.037037	2	7	2	33.881	5.43	19000000	5000000	17000000	31.72
Q8JZU2	Tricarboxylate transport protein, mitochondrial OS=Mus musculus GN=Slc25a1 PE=1 SV=	3.5369775	1	3	1	33.91	9.89	120000		120000	6.37
Q9D710	Thioredoxin-related transmembrane protein 2 OS=Mus musculus GN=Tmx2 PE=1 SV=1	13.220339	4	8	4	33.921	8.75	3900000	1500000	2700000	18.75
Q8C0M9	Isoaspartyl peptidase/L-asparaginase OS=Mus musculus GN=Asrgl1 PE=1 SV=1	10.736196	3	3	3	33.928	7.65	2900000	1600000	230000	8.60
P31230	Aminoacyl tRNA synthase complex-interacting multifunctional protein 1 OS=Mus musculus	3.8709677	1	3	1	33.976	8.35	4300000	1900000	3400000	11.01
Q99L04	Dehydrogenase/reductase SDR family member 1 OS=Mus musculus GN=Dhrs1 PE=1 SV	10.543131	3	6	3	33.983	8.35	4000000	1400000	3400000	15.25
Q62277	Synaptophysin OS=Mus musculus GN=Syp PE=1 SV=2	12.738854	4	29	4	34.002	4.94	150000000	100000000	110000000	71.20
Q91YL2	E3 ubiquitin-protein ligase RNF126 OS=Mus musculus GN=Rnf126 PE=1 SV=1	5.4313099	1	2	1	34.059	5.17		280000	830000	6.49
Q99KB8	Hydroxyacylglutathione hydrolase, mitochondrial OS=Mus musculus GN=Hagh PE=1 SV=	12.621359	3	7	3	34.062	7.75	2700000	1800000	4700000	20.70
Q9DCN2	NADH-cytochrome b5 reductase 3 OS=Mus musculus GN=Cyb5r3 PE=1 SV=3	10.963455	3	9	3	34.106	8.38	10000000	3600000	8200000	21.83
Q9DB73	NADH-cytochrome b5 reductase 1 OS=Mus musculus GN=Cyb5r1 PE=1 SV=1	6.8852459	2	4	2	34.113	8.87	2900000	1700000		10.14
Q9CR62	Mitochondrial 2-oxoglutarate/malate carrier protein OS=Mus musculus GN=Slc25a11 PE=	25.477707	8	17	8	34.133	9.94	15000000	6000000	11000000	37.60
P70452	Syntaxin-4 OS=Mus musculus GN=Stx4 PE=1 SV=1	4.3624161	1	1	1	34.144	6.14			550000	2.43
Q9DB41	Mitochondrial glutamate carrier 2 OS=Mus musculus GN=Slc25a18 PE=1 SV=4	17.1875	5	11	3	34.144	9.19	4300000	2700000	3100000	31.66
P49312	Heterogeneous nuclear ribonucleoprotein A1 OS=Mus musculus GN=Hnrnpa1 PE=1 SV=	31.875	10	31	10	34.175	9.23	30000000	7900000	22000000	102.11
Q99M28	RNA-binding protein with serine-rich domain 1 OS=Mus musculus GN=Rnps1 PE=1 SV=1	8.852459	2	5	2	34.188	11.84	8100000		6800000	14.43
P14869	60S acidic ribosomal protein P0 OS=Mus musculus GN=Rplp0 PE=1 SV=3	19.242902	6	14	6	34.195	6.25	14000000	4000000	4800000	36.70
Q9CWU6	Ubiquinol-cytochrome-c reductase complex assembly factor 1 OS=Mus musculus GN=Uq	5.7627119	1	1	1	34.277	8.48			1300000	2.61
P35550	rRNA 2'-O-methyltransferase fibrillarin OS=Mus musculus GN=Fbl PE=1 SV=2	3.3639144	1	1	1	34.286	10.24	1700000			2.42
Q8BJU0	Small glutamine-rich tetratricopeptide repeat-containing protein alpha OS=Mus musculus	7.9365079	2	5	2	34.301	5.06	2500000	2600000		10.91
Q64444	Carbonic anhydrase 4 OS=Mus musculus GN=Ca4 PE=1 SV=1	7.8688525	2	7	2	34.33	8.21	3400000	1800000	3200000	19.30
Q9CVB6	Actin-related protein 2/3 complex subunit 2 OS=Mus musculus GN=Arpc2 PE=1 SV=3	31	10	34	10	34.336	7.36	20000000	14000000	16000000	87.86
Q9Z204	Heterogeneous nuclear ribonucleoproteins C1/C2 OS=Mus musculus GN=Hnrnpc PE=1 S	12.460064	3	9	3	34.364	5.05	20000000	3600000	12000000	28.10
P47962	60S ribosomal protein L5 OS=Mus musculus GN=Rpl5 PE=1 SV=3	13.131313	4	17	4	34.379	9.77	14000000	5500000	5800000	40.83
Q9DBX2	Phosducin-like protein OS=Mus musculus GN=Pdcl PE=1 SV=1	3.986711	1	1	1	34.385	4.87			700000	2.84
P55088	Aquaporin-4 OS=Mus musculus GN=Aqp4 PE=1 SV=2	3.0959752	1	2	1	34.414	7.42	4500000		3900000	4.43
Q8K274	Ketosamine-3-kinase OS=Mus musculus GN=Fn3krp PE=1 SV=2	6.4724919	1	2	1	34.446	7.87		1100000	2600000	6.58
O88531	Palmitoyl-protein thioesterase 1 OS=Mus musculus GN=Ppt1 PE=1 SV=2	10.784314	3	5	3	34.467	8	5200000	1900000	810000	13.56
O89079	Coatomer subunit epsilon OS=Mus musculus GN=Cope PE=1 SV=3	2.9220779	1	3	1	34.545	5.06		800000	870000	6.23
O35593	26S proteasome non-ATPase regulatory subunit 14 OS=Mus musculus GN=Psmd14 PE=	5.1612903	2	2	2	34.555	6.52		970000		3.68
Q9D6M3	Mitochondrial glutamate carrier 1 OS=Mus musculus GN=Slc25a22 PE=1 SV=1	21.671827	7	15	5	34.648	9.09	13000000	6700000	9100000	43.80
Q9CWZ7	Gamma-soluble NSF attachment protein OS=Mus musculus GN=Napg PE=1 SV=1	41.025641	12	34	12	34.71	5.41	15000000	12000000	27000000	99.49
O70503	Very-long-chain 3-oxoacyl-CoA reductase OS=Mus musculus GN=Hsd17b12 PE=1 SV=1	8.0128205	2	5	2	34.719	9.52	3700000	1300000	2100000	15.54
Q9JHQ5	Leucine zipper transcription factor-like protein 1 OS=Mus musculus GN=Lztfl1 PE=1 SV=	5.6856187	1	2	1	34.752	5.17	450000		530000	5.37
Q9D7G0	Ribose-phosphate pyrophosphokinase 1 OS=Mus musculus GN=Prps1 PE=1 SV=4	15.09434	4	9	4	34.812	6.98	4100000	2300000	3000000	25.36
Q9D8B7	Junctional adhesion molecule C OS=Mus musculus GN=Jam3 PE=1 SV=2	13.548387	3	5	3	34.816	7.03	940000	740000	1000000	14.44
Q922Q8	Leucine-rich repeat-containing protein 59 OS=Mus musculus GN=Lrrc59 PE=1 SV=1	12.703583	3	7	3	34.856	9.52	4700000	1600000	2800000	19.25
P42669	Transcriptional activator protein Pur-alpha OS=Mus musculus GN=Pura PE=1 SV=1	32.398754	7	14	7	34.862	6.44	15000000	3500000	9300000	51.31
Q8C4Q6	Axin interactor, dorsalization-associated protein OS=Mus musculus GN=Aida PE=1 SV=1	5.9016393	1	1	1	34.866	6.74			570000	2.78
Q9WUD1	STIP1 homology and U box-containing protein 1 OS=Mus musculus GN=Stub1 PE=1 SV=	8.2236842	2	3	2	34.887	6.01	2000000		1400000	7.25
Q9CRD2	ER membrane protein complex subunit 2 OS=Mus musculus GN=Emc2 PE=1 SV=1	3.3670034	1	3	1	34.912	6.81	3000000	1600000	2900000	8.46

Q99J47	Dehydrogenase/reductase SDR family member 7B OS=Mus musculus GN=Dhrs7b PE=1	4.6439628	1	1	1	34.964	9.63				3.02
Q9JJZ4	Ubiquitin-conjugating enzyme E2 J1 OS=Mus musculus GN=Ube2j1 PE=1 SV=2	4.7169811	1	3	1	34.968	6.99	2100000	810000	1800000	9.15
Q9DCV4	Regulator of microtubule dynamics protein 1 OS=Mus musculus GN=Rmdn1 PE=1 SV=2	3.6065574	1	1	1	34.978	8.7			1100000	2.83
Q99LC5	Electron transfer flavoprotein subunit alpha, mitochondrial OS=Mus musculus GN=Etfa PE	18.018018	4	6	4	34.988	8.38	1500000		1900000	19.27
Q9ER35	Fructosamine-3-kinase OS=Mus musculus GN=Fn3k PE=1 SV=1	5.8252427	1	3	1	35.01	8.4	820000	450000	1000000	12.09
P68040	Receptor of activated protein C kinase 1 OS=Mus musculus GN=Rack1 PE=1 SV=3	32.807571	8	19	8	35.055	7.69	20000000	11000000	18000000	53.95
Q8K1Z0	Ubiquinone biosynthesis protein COQ9, mitochondrial OS=Mus musculus GN=Coq9 PE=1	9.5846645	2	5	2	35.061	5.92	5100000	2800000	5900000	19.92
Q9CQR6	Serine/threonine-protein phosphatase 6 catalytic subunit OS=Mus musculus GN=Ppp6c P	8.852459	2	2	2	35.136	5.69		690000		5.53
Q8BP27	Swi5-dependent recombination DNA repair protein 1 homolog OS=Mus musculus GN=Sfr	9.0909091	2	3	2	35.161	5.15	370000		360000	7.23
P14094	Sodium/potassium-transporting ATPase subunit beta-1 OS=Mus musculus GN=Atp1b1 PI	32.894737	11	90	11	35.172	8.65	410000000	310000000	370000000	251.38
Q99JY8	Phospholipid phosphatase 3 OS=Mus musculus GN=Plpp3 PE=1 SV=1	10.576923	3	8	3	35.193	9.07	12000000	5000000	8800000	22.14
Q8R0Y8	Mitochondrial coenzyme A transporter SLC25A42 OS=Mus musculus GN=Slc25a42 PE=1	2.5157233	1	1	1	35.219	10.05	1300000			2.24
Q8K097	Protein lifeguard 2 OS=Mus musculus GN=Faim2 PE=2 SV=1	6.9400631	1	3	1	35.235	6.92	8500000	3000000	2300000	9.65
P47199	Quinone oxidoreductase OS=Mus musculus GN=Cryz PE=1 SV=1	4.2296073	1	1	1	35.246	8.07			340000	3.83
O35682	Myeloid-associated differentiation marker OS=Mus musculus GN=Myadm PE=1 SV=2	5.3125	1	3	1	35.261	8.31	2100000	930000	1500000	12.00
Q8C3W1	Uncharacterized protein C1orf198 homolog OS=Mus musculus PE=1 SV=1	5.2795031	1	1	1	35.295	5.24		430000		2.44
Q9D0M3	Cytochrome c1, heme protein, mitochondrial OS=Mus musculus GN=Cyc1 PE=1 SV=1	25.230769	6	32	6	35.305	9.16	20000000	13000000	18000000	100.14
Q9CZR8	Elongation factor Ts, mitochondrial OS=Mus musculus GN=Tsfm PE=1 SV=1	2.7777778	1	1	1	35.312	7.06			720000	1.82
Q8R010	Aminoacyl tRNA synthase complex-interacting multifunctional protein 2 OS=Mus musculus	7.8125	1	2	1	35.355	7.83			1300000	5.02
Q91V61	Sideroflexin-3 OS=Mus musculus GN=Sfxn3 PE=1 SV=1	31.152648	8	23	7	35.384	9.51	34000000	18000000	23000000	72.62
Q9D0Q7	39S ribosomal protein L45, mitochondrial OS=Mus musculus GN=Mrpl45 PE=1 SV=1	3.2679739	1	3	1	35.388	9.23	1100000	490000	800000	7.09
Q99L13	3-hydroxyisobutyrate dehydrogenase, mitochondrial OS=Mus musculus GN=Hibadh PE=1	4.7761194	1	3	1	35.417	8.13	1400000	1500000	1700000	8.65
Q7TN79	A-kinase anchor protein 7 isoform gamma OS=Mus musculus GN=Akap7 PE=1 SV=2	6.6878981	2	5	2	35.46	7.87	3600000	1700000	2300000	12.55
P28352	DNA-(apurinic or apyrimidinic site) lyase OS=Mus musculus GN=Apex1 PE=1 SV=2	4.1009464	1	1	1	35.468	7.91	1100000			2.58
Q9JJV5	Voltage-dependent calcium channel gamma-3 subunit OS=Mus musculus GN=Cacng3 PE	6.3492063	1	1	1	35.493	9.48				5.16
Q9D1M0	Protein SEC13 homolog OS=Mus musculus GN=Sec13 PE=1 SV=3	8.6956522	2	6	2	35.543	5.38	1800000	1300000	2300000	19.30
P62715	Serine/threonine-protein phosphatase 2A catalytic subunit beta isoform OS=Mus musculu	29.449838	7	25	2	35.552	5.43		2100000	6800000	88.90
Q922H9	Zinc finger protein 330 OS=Mus musculus GN=Znf330 PE=1 SV=1	5.3797468	1	1	1	35.584	6.16	910000			1.94
P63330	Serine/threonine-protein phosphatase 2A catalytic subunit alpha isoform OS=Mus muscul	26.860841	6	23	1	35.585	5.54		4600000		83.80
P08249	Malate dehydrogenase, mitochondrial OS=Mus musculus GN=Mdh2 PE=1 SV=3	47.633136	13	32	13	35.589	8.68	27000000	17000000	26000000	91.84
P47802	Metaxin-1 OS=Mus musculus GN=Mtx1 PE=1 SV=1	9.148265	2	5	2	35.601	6.18	1400000	4100000	3000000	14.86
Q9Z1D1	Eukaryotic translation initiation factor 3 subunit G OS=Mus musculus GN=Eif3g PE=1 SV=	8.4375	2	5	2	35.616	5.9	5800000	1900000	5000000	15.55
Q99JR1	Sideroflexin-1 OS=Mus musculus GN=Sfxn1 PE=1 SV=3	22.670807	6	14	5	35.626	9.23	11000000	5400000	7500000	38.03
P62960	Nuclease-sensitive element-binding protein 1 OS=Mus musculus GN=Ybx1 PE=1 SV=3	9.3167702	2	5	2	35.709	9.88	230000	210000	270000	16.43
O70252	Heme oxygenase 2 OS=Mus musculus GN=Hmox2 PE=1 SV=1	12.698413	4	7	4	35.716	5.87	1000000	1400000	1600000	17.48
P48036	Annexin A5 OS=Mus musculus GN=Anxa5 PE=1 SV=1	21.630094	7	14	7	35.73	4.96	4700000	3700000	3200000	33.99
Q9CPX6	Ubiquitin-like-conjugating enzyme ATG3 OS=Mus musculus GN=Atg3 PE=1 SV=1	4.1401274	1	1	1	35.773	4.72			3100000	2.59
P16858	Glyceraldehyde-3-phosphate dehydrogenase OS=Mus musculus GN=Gapdh PE=1 SV=2	55.555556	16	63	16	35.787	8.25	58000000	130000000	130000000	194.16
Q9DCJ1	Target of rapamycin complex subunit LST8 OS=Mus musculus GN=Mlst8 PE=1 SV=1	5.5214724	1	1	1	35.828	5.86			1800000	2.73
Q8R3Q0	Store-operated calcium entry-associated regulatory factor OS=Mus musculus GN=Saraf F	4.1916168	1	2	1	35.833	8.19		610000	620000	4.82
Q9CRY7	Glycerophosphodiester phosphodiesterase domain-containing protein 1 OS=Mus musculu	3.8216561	1	3	1	35.844	8.31	2400000	790000	1300000	9.06
P08226	Apolipoprotein E OS=Mus musculus GN=Apoe PE=1 SV=2	37.942122	10	22	10	35.844	5.68	8300000	5600000	8000000	60.10
O88545	COP9 signalosome complex subunit 6 OS=Mus musculus GN=Cops6 PE=1 SV=1	4.6296296	1	3	1	35.857	5.73	3200000	1800000	3100000	9.51
Q6WVG3	BTB/POZ domain-containing protein KCTD12 OS=Mus musculus GN=Kctd12 PE=1 SV=1	13.761468	4	9	4	35.87	5.81	3400000	1400000	2500000	25.61
O88602	Voltage-dependent calcium channel gamma-2 subunit OS=Mus musculus GN=Cacng2 PE	4.0247678	1	2	1	35.872	8.98	1600000		1600000	3.94
Q99JG3	Annexin A13 OS=Mus musculus GN=Anxa13 PE=1 SV=3	5.0473186	1	1	1	35.899	5.99				2.64
E9PUL5	Proline-rich transmembrane protein 2 OS=Mus musculus GN=Prrt2 PE=1 SV=1	38.728324	6	22	6	35.902	4.63	43000000	15000000	34000000	88.92
Q3TY60	Protein FAM131B OS=Mus musculus GN=Fam131b PE=1 SV=1	6.626506	2	4	2	35.935	4.45	1800000		2500000	6.98

Q8BJZ4	28S ribosomal protein S35, mitochondrial OS=Mus musculus GN=Mrps35 PE=1 SV=2	4.0625	1	1	1	35.953	8.59		430000		2.09
P10518	Delta-aminolevulinic acid dehydratase OS=Mus musculus GN=Alad PE=1 SV=1	5.7575758	1	1	1	36	6.79			430000	4.30
Q9CY27	Very-long-chain enoyl-CoA reductase OS=Mus musculus GN=Tecr PE=1 SV=1	5.8441558	2	2	2	36.067	9.55	8200000			4.37
Q6ZWX6	Eukaryotic translation initiation factor 2 subunit 1 OS=Mus musculus GN=Eif2s1 PE=1 SV	20.952381	5	15	5	36.085	5.08	3900000	1400000	3600000	50.34
Q9Z0G0	PDZ domain-containing protein GIPC1 OS=Mus musculus GN=Gipc1 PE=1 SV=1	4.2042042	1	3	1	36.107	5.91		1100000	950000	9.14
Q9WUM5	Succinate--CoA ligase [ADP/GDP-forming] subunit alpha, mitochondrial OS=Mus musculu	4.6242775	1	2	1	36.132	9.39		5000000	6800000	7.48
P70372	ELAV-like protein 1 OS=Mus musculus GN=Elavl1 PE=1 SV=2	6.4417178	2	2	2	36.146	9.04			3900000	5.29
P35803	Neuronal membrane glycoprotein M6-b OS=Mus musculus GN=Gpm6b PE=1 SV=2	15.853659	5	24	5	36.186	6.14	38000000	22000000	37000000	74.73
O55091	Protein IMPACT OS=Mus musculus GN=Impact PE=1 SV=2	9.1194969	2	5	2	36.253	5.05	4600000	1400000	2800000	14.01
Q9QZX7	Serine racemase OS=Mus musculus GN=Srr PE=1 SV=1	4.719764	1	2	1	36.336	6.02		650000	1300000	6.16
Q9ERI6	Retinol dehydrogenase 14 OS=Mus musculus GN=Rdh14 PE=1 SV=1	2.3952096	1	2	1	36.343	8.18	2100000	640000		4.28
Q9QZD9	Eukaryotic translation initiation factor 3 subunit I OS=Mus musculus GN=Eif3i PE=1 SV=1	19.692308	6	15	6	36.438	5.64	3900000	2500000	4600000	40.14
Q8CEE7	Retinol dehydrogenase 13 OS=Mus musculus GN=Rdh13 PE=1 SV=1	3.8922156	1	2	1	36.441	8.85	490000		620000	5.29
P06151	L-lactate dehydrogenase A chain OS=Mus musculus GN=Ldha PE=1 SV=3	23.795181	7	28	6	36.475	7.74	29000000	12000000	20000000	84.26
P14152	Malate dehydrogenase, cytoplasmic OS=Mus musculus GN=Mdh1 PE=1 SV=3	29.94012	9	25	9	36.488	6.58	50000000	20000000	48000000	71.98
Q9D1H7	Golgi to ER traffic protein 4 homolog OS=Mus musculus GN=Get4 PE=1 SV=2	8.2568807	2	4	2	36.502	5.41	2200000	810000	1900000	13.06
P26516	26S proteasome non-ATPase regulatory subunit 7 OS=Mus musculus GN=Psmd7 PE=1 S	3.1152648	1	3	1	36.517	6.77	4800000	2500000	3500000	7.27
P16125	L-lactate dehydrogenase B chain OS=Mus musculus GN=Ldhb PE=1 SV=2	35.928144	13	36	12	36.549	6.05	48000000	23000000	45000000	108.67
Q9JII6	Alcohol dehydrogenase [NADP(+)] OS=Mus musculus GN=Akr1a1 PE=1 SV=3	16.923077	4	8	4	36.564	7.39	1200000	1100000	1900000	22.66
P61965	WD repeat-containing protein 5 OS=Mus musculus GN=Wdr5 PE=1 SV=1	4.491018	1	1	1	36.565	8.27	250000			2.65
P58389	Serine/threonine-protein phosphatase 2A activator OS=Mus musculus GN=Ppp2r4 PE=1	4.0247678	1	3	1	36.687	6.39	3000000	1300000	2200000	7.88
Q8HW98	IgLON family member 5 OS=Mus musculus GN=Iglon5 PE=2 SV=2	9.2261905	2	4	2	36.744	7.69	1100000	1500000	2100000	10.90
Q921M7	Protein FAM49B OS=Mus musculus GN=Fam49b PE=1 SV=1	9.8765432	2	3	2	36.753	6.06	4600000		3100000	10.09
Q05117	Tartrate-resistant acid phosphatase type 5 OS=Mus musculus GN=Acp5 PE=1 SV=2	8.5626911	1	1	1	36.784	8.9		2900000		2.60
Q8VCY8	Phospholipid phosphatase-related protein type 2 OS=Mus musculus GN=Plppr2 PE=1 SV	5.5393586	1	1	1	36.912	9.5	5100000			3.06
Q8R001	Microtubule-associated protein RP/EB family member 2 OS=Mus musculus GN=Mapre2 P	24.846626	6	15	6	36.923	5.38	7500000	6200000	11000000	46.15
Q9WVA3	Mitotic checkpoint protein BUB3 OS=Mus musculus GN=Bub3 PE=1 SV=2	12.269939	3	5	3	36.931	6.84	9300000	2300000	8300000	13.57
P63087	Serine/threonine-protein phosphatase PP1-gamma catalytic subunit OS=Mus musculus G	13.312693	4	8	2	36.96	6.54	3200000	2600000	4100000	18.89
P21995	Embigin OS=Mus musculus GN=Emb PE=1 SV=2	3.9393939	1	1	1	37.041	6.02				2.83
O35887	Calumenin OS=Mus musculus GN=Calu PE=1 SV=1	23.174603	6	16	6	37.041	4.67	4000000	3200000	4700000	44.24
Q8BVI5	Syntaxin-16 OS=Mus musculus GN=Stx16 PE=1 SV=3	8.2822086	2	2	2	37.057	5.86	3100000	470000		6.04
Q8C0L0	Thioredoxin-related transmembrane protein 4 OS=Mus musculus GN=Tmx4 PE=1 SV=2	26.865672	5	14	5	37.108	4.37	11000000	3100000	6900000	46.66
P62141	Serine/threonine-protein phosphatase PP1-beta catalytic subunit OS=Mus musculus GN=	13.149847	4	10	1	37.163	6.19	3400000			23.66
Q8BP92	Reticulocalbin-2 OS=Mus musculus GN=Rcn2 PE=1 SV=1	24.0625	6	15	6	37.248	4.42	3700000	4000000	6200000	54.72
P10605	Cathepsin B OS=Mus musculus GN=Ctsb PE=1 SV=2	13.274336	4	9	4	37.256	5.91	1900000	1500000	1900000	23.31
P29699	Alpha-2-HS-glycoprotein OS=Mus musculus GN=Ahsg PE=1 SV=1	4.9275362	1	2	1	37.302	6.51	980000		340000	5.71
Q925N0	Sideroflexin-5 OS=Mus musculus GN=Sfxn5 PE=1 SV=2	14.912281	4	9	4	37.305	9.44	7600000	2500000	4700000	28.81
P62880	Guanine nucleotide-binding protein G(I)/G(S)/G(T) subunit beta-2 OS=Mus musculus GN=	27.352941	7	39	2	37.307	6	50000000	31000000	56000000	110.36
Q9CXI0	2-methoxy-6-polyprenyl-1,4-benzoquinol methylase, mitochondrial OS=Mus musculus GN	3.6697248	1	3	1	37.312	7.49	1100000	1000000	1300000	8.01
Q8BHZ0	Protein FAM49A OS=Mus musculus GN=Fam49a PE=1 SV=1	4.3343653	1	2	1	37.319	6.01	4800000		2800000	6.90
Q9R020	Zinc finger Ran-binding domain-containing protein 2 OS=Mus musculus GN=Zranb2 PE=	4.2424242	1	2	1	37.328	9.89	6500000		5200000	7.86
Q8BVA5	Lipid droplet-associated hydrolase OS=Mus musculus GN=Ldah PE=1 SV=1	3.9877301	1	1	1	37.349	8.28	1100000			2.76
P62874	Guanine nucleotide-binding protein G(I)/G(S)/G(T) subunit beta-1 OS=Mus musculus GN=	46.470588	12	47	7	37.353	6	76000000	52000000	100000000	139.42
P29387	Guanine nucleotide-binding protein subunit beta-4 OS=Mus musculus GN=Gnb4 PE=1 SV	15.294118	4	21	2	37.355	6.16	3700000	1700000		52.72
O88569	Heterogeneous nuclear ribonucleoproteins A2/B1 OS=Mus musculus GN=Hnrnpa2b1 PE	56.090652	19	110	19	37.38	8.95	180000000	80000000	150000000	336.91
P60335	Poly(rC)-binding protein 1 OS=Mus musculus GN=Pcbp1 PE=1 SV=1	14.044944	4	15	1	37.474	7.09	3800000	1300000	2200000	44.68
Q9CY73	39S ribosomal protein L44, mitochondrial OS=Mus musculus GN=Mrpl44 PE=1 SV=3	5.7057057	1	1	1	37.504	8.51	370000			2.54
P62137	Serine/threonine-protein phosphatase PP1-alpha catalytic subunit OS=Mus musculus GN	15.757576	5	9	2	37.516	6.33	1400000	3300000		21.56

P06797	Cathepsin L1 OS=Mus musculus GN=Ctsl PE=1 SV=2	4.7904192	1	2	1	37.523	6.83	480000	700000		6.95
O35864	COP9 signalosome complex subunit 5 OS=Mus musculus GN=Cops5 PE=1 SV=3	2.994012	1	3	1	37.525	6.54	1600000	1100000	1800000	7.82
Q8K0S0	Phytanoyl-CoA hydroxylase-interacting protein OS=Mus musculus GN=Phyhip PE=1 SV=	10	3	7	3	37.53	7.01	4500000	1900000	4700000	18.31
P13707	Glycerol-3-phosphate dehydrogenase [NAD(+)], cytoplasmic OS=Mus musculus GN=Gpd	5.1575931	2	4	2	37.548	7.17	2300000	830000	1500000	8.38
Q99N96	39S ribosomal protein L1, mitochondrial OS=Mus musculus GN=Mrpl1 PE=1 SV=2	4.7619048	1	2	1	37.573	8.72		540000		5.89
O08915	AH receptor-interacting protein OS=Mus musculus GN=Aip PE=1 SV=1	5.7575758	1	1	1	37.581	6.4			300000	3.00
Q9WUP7	Ubiquitin carboxyl-terminal hydrolase isozyme L5 OS=Mus musculus GN=Uchl5 PE=1 SV	4.8632219	1	1	1	37.593	5.33	940000			2.07
Q9JL56	Glycerophosphodiester phosphodiesterase 1 OS=Mus musculus GN=Gde1 PE=1 SV=1	3.021148	1	1	1	37.605	6.9	2700000			2.48
Q9QYS9	Protein quaking OS=Mus musculus GN=Qki PE=1 SV=1	2.3460411	1	1	1	37.647	8.56	720000			2.16
Q8K221	Arfaptin-2 OS=Mus musculus GN=Arfip2 PE=1 SV=2	9.6774194	2	5	2	37.749	5.87	340000	990000	850000	18.71
Q9CQM9	Glutaredoxin-3 OS=Mus musculus GN=Glrx3 PE=1 SV=1	7.1216617	2	4	2	37.754	5.59	2400000	1200000	2500000	9.93
Q9D832	DnaJ homolog subfamily B member 4 OS=Mus musculus GN=Dnajb4 PE=1 SV=1	15.133531	4	9	3	37.758	8.59	2100000	1200000	1900000	26.95
Q9CXD6	Mitochondrial calcium uniporter regulator 1 OS=Mus musculus GN=Mcur1 PE=1 SV=1	2.3529412	1	1	1	37.826	10.2			310000	2.07
Q62313	Trans-Golgi network integral membrane protein 1 OS=Mus musculus GN=Tgoln1 PE=1 S	25.779037	4	10	4	37.826	5.34	1300000	710000	740000	31.11
P52795	Ephrin-B1 OS=Mus musculus GN=Efnb1 PE=1 SV=1	11.014493	2	3	2	37.835	9.03	700000		1100000	8.60
Q9JM96	Cdc42 effector protein 4 OS=Mus musculus GN=Cdc42ep4 PE=1 SV=1	4.2979943	1	1	1	37.846	5.36	650000			1.92
Q8BFZ9	Erlin-2 OS=Mus musculus GN=Erlin2 PE=1 SV=1	16.470588	5	11	3	37.849	5.5	6300000	4800000	5900000	30.98
Q9QYA2	Mitochondrial import receptor subunit TOM40 homolog OS=Mus musculus GN=Tomm40 I	19.113573	4	7	4	37.871	7.74	2700000	1900000	2100000	22.64
Q80Z24	Neuronal growth regulator 1 OS=Mus musculus GN=Negr1 PE=1 SV=1	29.597701	7	25	7	37.876	6.52	14000000	9200000	15000000	79.15
Q80V91	Probable E3 ubiquitin-protein ligase DTX3 OS=Mus musculus GN=Dtx3 PE=1 SV=2	6.6282421	1	1	1	37.958	8.73				3.99
Q99PJ0	Neurotrimin OS=Mus musculus GN=Ntm PE=1 SV=2	35.755814	11	32	11	37.96	7.81	71000000	61000000	30000000	104.72
Q8K021	Secretory carrier-associated membrane protein 1 OS=Mus musculus GN=Scamp1 PE=1 S	17.455621	5	20	5	38.004	7.71	12000000	6500000	12000000	58.28
Q8BLK3	Limbic system-associated membrane protein OS=Mus musculus GN=Lsamp PE=1 SV=1	38.416422	13	60	13	38.063	6.65	32000000	30000000	40000000	184.32
Q99L45	Eukaryotic translation initiation factor 2 subunit 2 OS=Mus musculus GN=Eif2s2 PE=1 SV	20.543807	5	10	5	38.068	5.8	6200000	890000	3400000	31.58
Q05186	Reticulocalbin-1 OS=Mus musculus GN=Rcn1 PE=1 SV=1	4	1	2	1	38.09	4.84		500000	1000000	4.03
Q91VM9	Inorganic pyrophosphatase 2, mitochondrial OS=Mus musculus GN=Ppa2 PE=1 SV=1	13.636364	3	6	3	38.09	6.98	1600000	780000	1700000	18.12
P40336	Vacuolar protein sorting-associated protein 26A OS=Mus musculus GN=Vps26a PE=1 SV	15.29052	3	6	3	38.09	6.57	6900000	3800000	3800000	21.82
Q8BK64	Activator of 90 kDa heat shock protein ATPase homolog 1 OS=Mus musculus GN=Ahsa1	3.2544379	1	3	1	38.093	5.53	900000	400000	1800000	6.87
Q9CX34	Protein SGT1 homolog OS=Mus musculus GN=Sugt1 PE=1 SV=3	3.2738095	1	3	1	38.135	5.45	2000000	1100000	2800000	7.89
Q9CXR1	Dehydrogenase/reductase SDR family member 7 OS=Mus musculus GN=Dhrs7 PE=1 SV	5.9171598	1	1	1	38.143	8.32	1300000			2.17
Q9QYJ3	DnaJ homolog subfamily B member 1 OS=Mus musculus GN=Dnajb1 PE=1 SV=3	15	4	9	3	38.143	8.63	1900000	1500000	1300000	22.97
Q8JZW5	SH2 domain-containing protein 5 OS=Mus musculus GN=Sh2d5 PE=1 SV=2	4.3478261	1	3	1	38.149	8.32	980000		640000	8.41
Q922Q1	Mitochondrial amidoxime reducing component 2 OS=Mus musculus GN=Marc2 PE=1 SV=	13.905325	5	9	5	38.17	8.68	9200000	5000000	7200000	24.76
Q8R2Y0	Monoacylglycerol lipase ABHD6 OS=Mus musculus GN=Abhd6 PE=1 SV=1	11.309524	3	6	3	38.18	8.47	3900000	1500000	3100000	21.48
Q61990	Poly(rC)-binding protein 2 OS=Mus musculus GN=Pcbp2 PE=1 SV=1	13.812155	4	15	1	38.197	6.79	4100000	2900000	2500000	44.61
Q3ULJ0	Glycerol-3-phosphate dehydrogenase 1-like protein OS=Mus musculus GN=Gpd1l PE=1 S	3.7037037	1	2	1	38.201	6.77	5700000		3600000	5.85
Q80XN0	D-beta-hydroxybutyrate dehydrogenase, mitochondrial OS=Mus musculus GN=Bdh1 PE=	21.574344	6	17	6	38.274	9.01	9100000	3700000	7500000	52.81
Q60668	Heterogeneous nuclear ribonucleoprotein D0 OS=Mus musculus GN=Hnrnpd PE=1 SV=2	18.028169	5	12	4	38.33	7.81	41000000	14000000	24000000	37.16
O35685	Nuclear migration protein nudC OS=Mus musculus GN=Nudc PE=1 SV=1	17.168675	5	13	5	38.334	5.26	4000000	2200000	5000000	35.38
Q9ERR1	Nuclear distribution protein nudE-like 1 OS=Mus musculus GN=Ndel1 PE=1 SV=2	3.7681159	1	2	1	38.342	5.24	1800000		2000000	7.05
Q8K4R4	Cytoplasmic phosphatidylinositol transfer protein 1 OS=Mus musculus GN=Pitpnc1 PE=1	3.6144578	1	2	1	38.359	6.32	2200000		1800000	5.12
Q99JB2	Stomatin-like protein 2, mitochondrial OS=Mus musculus GN=Stoml2 PE=1 SV=1	18.413598	5	8	5	38.361	8.87	2700000	2000000	2000000	25.70
Q9QUN9	Dickkopf-related protein 3 OS=Mus musculus GN=Dkk3 PE=2 SV=1	2.2922636	1	3	1	38.363	4.54	1400000	1400000	1300000	7.45
Q8CAK1	Putative transferase CAF17 homolog, mitochondrial OS=Mus musculus GN=Iba57 PE=1 S	3.6312849	1	2	1	38.375	9.01	660000		690000	4.76
Q9Z1Z2	Serine-threonine kinase receptor-associated protein OS=Mus musculus GN=Strap PE=1 S	34.857143	7	14	7	38.418	5.12	4900000	2700000	4300000	45.36
O35609	Secretory carrier-associated membrane protein 3 OS=Mus musculus GN=Scamp3 PE=1 S	8.3094556	2	3	2	38.433	7.64	770000	530000	1400000	7.67
Q8BTG7	Protein NDRG4 OS=Mus musculus GN=Ndrg4 PE=1 SV=1	10.795455	2	2	2	38.484	6.32	1500000	600000		6.51
P70441	Na(+)/H(+) exchange regulatory cofactor NHE-RF1 OS=Mus musculus GN=Slc9a3r1 PE=	14.929577	5	7	5	38.577	5.9	4600000	2600000	3700000	16.14

P07356	Annexin A2 OS=Mus musculus GN=Anxa2 PE=1 SV=2	14.159292	5	6	5	38.652	7.69			4000000	18.18
Q9R226	KH domain-containing, RNA-binding, signal transduction-associated protein 3 OS=Mus m	8.0924855	3	6	2	38.784	8.1	8500000	2600000	4600000	12.84
O54984	ATPase Asna1 OS=Mus musculus GN=Asna1 PE=1 SV=2	2.8735632	1	2	1	38.797	4.91	870000		860000	4.08
Q9WU01	KH domain-containing, RNA-binding, signal transduction-associated protein 2 OS=Mus m	5.730659	2	2	1	38.843	6.48	510000			4.23
Q8BK63	Casein kinase I isoform alpha OS=Mus musculus GN=Csnk1a1 PE=1 SV=2	9.495549	2	4	2	38.89	9.57	630000		210000	13.37
P61963	DDB1- and CUL4-associated factor 7 OS=Mus musculus GN=Dcaf7 PE=1 SV=1	2.6315789	1	1	1	38.901	5.52		1300000		2.54
Q62421	Endophilin-A3 OS=Mus musculus GN=Sh3gl3 PE=1 SV=1	18.443804	5	8	5	38.91	5.12	1400000	1400000	1600000	22.08
Q91X78	Erlin-1 OS=Mus musculus GN=Erlin1 PE=1 SV=1	8.3815029	3	5	1	38.912	7.21		130000		11.90
Q9D051	Pyruvate dehydrogenase E1 component subunit beta, mitochondrial OS=Mus musculus G	32.311978	10	52	10	38.912	6.87	87000000	61000000	89000000	177.96
Q9DAR7	m7GpppX diphosphatase OS=Mus musculus GN=Dcps PE=1 SV=1	10.059172	2	2	2	38.964	6.48	860000			6.14
Q3TWW8	Serine/arginine-rich splicing factor 6 OS=Mus musculus GN=Srsf6 PE=1 SV=1	20.058997	7	13	4	39.002	11.46	5200000		2400000	34.27
Q9CYN9	Renin receptor OS=Mus musculus GN=Atp6ap2 PE=1 SV=2	6.2857143	2	4	2	39.067	5.54	4400000	3100000	6200000	12.05
Q8C0E2	Vacuolar protein sorting-associated protein 26B OS=Mus musculus GN=Vps26b PE=1 SV	17.559524	5	17	5	39.1	7.37	10000000	7800000	8500000	51.13
Q91WA3	Histone deacetylase 11 OS=Mus musculus GN=Hdac11 PE=1 SV=1	3.7463977	1	1	1	39.132	7.14			1000000	2.94
P05064	Fructose-bisphosphate aldolase A OS=Mus musculus GN=Aldoa PE=1 SV=2	52.747253	17	70	15	39.331	8.09	130000000	76000000	110000000	227.74
Q8BGU5	Cyclin-Y OS=Mus musculus GN=Ccny PE=1 SV=1	3.5190616	1	1	1	39.37	7.2	2900000			2.66
P05063	Fructose-bisphosphate aldolase C OS=Mus musculus GN=Aldoc PE=1 SV=4	52.61708	15	42	13	39.37	7.12	36000000	16000000	32000000	132.80
Q9Z0P5	Twinfilin-2 OS=Mus musculus GN=Twf2 PE=1 SV=1	18.624642	4	8	4	39.446	6.8	1800000	2000000	2300000	27.81
Q9JKK7	Tropomodulin-2 OS=Mus musculus GN=Tmod2 PE=1 SV=2	10.25641	3	6	3	39.487	5.35	4500000	3000000	2500000	17.31
Q60900	ELAV-like protein 3 OS=Mus musculus GN=Elavl3 PE=1 SV=1	5.7220708	1	2	1	39.508	9.28	1200000	780000		5.68
P28474	Alcohol dehydrogenase class-3 OS=Mus musculus GN=Adh5 PE=1 SV=3	4.0106952	1	2	1	39.522	7.25	1400000		2100000	5.22
Q8BTV1	Tumor suppressor candidate 3 OS=Mus musculus GN=Tusc3 PE=1 SV=1	4.0345821	1	1	1	39.523	10.11				2.65
Q60899	ELAV-like protein 2 OS=Mus musculus GN=Elavl2 PE=2 SV=1	12.222222	4	8	4	39.552	9.13	4100000	1600000	3400000	22.22
Q62086	Serum paraoxonase/arylesterase 2 OS=Mus musculus GN=Pon2 PE=1 SV=2	5.0847458	1	1	1	39.592	5.83			1100000	2.99
Q8VEM8	Phosphate carrier protein, mitochondrial OS=Mus musculus GN=Slc25a3 PE=1 SV=1	16.806723	6	20	6	39.606	9.26	130000000	50000000	78000000	52.17
Q9D6R2	Isocitrate dehydrogenase [NAD] subunit alpha, mitochondrial OS=Mus musculus GN=Idh3	29.234973	11	33	11	39.613	6.73	26000000	18000000	17000000	96.76
Q8BG05	Heterogeneous nuclear ribonucleoprotein A3 OS=Mus musculus GN=Hnrnpa3 PE=1 SV=	32.453826	12	48	12	39.628	9.01	100000000	23000000	64000000	150.06
Q3UMR5	Calcium uniporter protein, mitochondrial OS=Mus musculus GN=Mcu PE=1 SV=2	10	3	7	3	39.657	8.56	3600000	2300000	3600000	19.77
Q9QZQ8	Core histone macro-H2A.1 OS=Mus musculus GN=H2afy PE=1 SV=3	25.268817	8	24	6	39.71	9.8	17000000	2200000	5500000	74.55
Q9D880	Mitochondrial import inner membrane translocase subunit TIM50 OS=Mus musculus GN=	15.580737	4	6	4	39.752	8.13	4100000	2700000	4200000	17.14
O54946	DnaJ homolog subfamily B member 6 OS=Mus musculus GN=Dnajb6 PE=1 SV=4	6.3013699	2	6	1	39.783	9.36	16000000	11000000	14000000	20.07
Q91WK2	Eukaryotic translation initiation factor 3 subunit H OS=Mus musculus GN=Eif3h PE=1 SV=	5.3977273	1	5	1	39.807	6.67	1800000	1700000	3500000	22.62
Q8R4E6	Purine-rich element-binding protein gamma OS=Mus musculus GN=Purg PE=1 SV=1	9.7142857	2	4	2	39.913	9.51	2900000	210000	1700000	11.12
P97792	Coxsackievirus and adenovirus receptor homolog OS=Mus musculus GN=Cxadr PE=1 S	7.3972603	2	3	2	39.922	6.96	1600000	710000	1100000	7.38
Q62420	Endophilin-A1 OS=Mus musculus GN=Sh3gl2 PE=1 SV=2	36.647727	11	51	7	39.93	5.39	48000000	27000000	53000000	155.94
Q9Z1S5	Neuronal-specific septin-3 OS=Mus musculus GN=Sept3 PE=1 SV=2	35.428571	12	32	12	40.013	6.81	17000000	12000000	21000000	92.59
Q91YR1	Twinfilin-1 OS=Mus musculus GN=Twf1 PE=1 SV=2	18.285714	5	10	5	40.054	6.67	1600000	1600000	4600000	32.60
P18872	Guanine nucleotide-binding protein G(o) subunit alpha OS=Mus musculus GN=Gnao1 PE	46.327684	15	115	13	40.059	5.53	350000000	140000000	410000000	322.67
Q8CCK0	Core histone macro-H2A.2 OS=Mus musculus GN=H2afy2 PE=1 SV=3	14.784946	4	9	2	40.067	9.69	6300000	350000	3600000	25.46
P55264	Adenosine kinase OS=Mus musculus GN=Adk PE=1 SV=2	2.2160665	1	2	1	40.123	6.21	1800000		840000	4.50
P51863	V-type proton ATPase subunit d 1 OS=Mus musculus GN=Atp6v0d1 PE=1 SV=2	20.512821	6	20	6	40.275	5	51000000	42000000	58000000	51.58
B2RSH2	Guanine nucleotide-binding protein G(i) subunit alpha-1 OS=Mus musculus GN=Gnai1 PE	32.768362	10	41	5	40.335	5.97	17000000	9300000	26000000	122.62
Q9D8N2	Protein FAM45A OS=Mus musculus GN=Fam45a PE=1 SV=2	9.2436975	2	3	2	40.395	6.47	1900000	1600000		10.05
P49813	Tropomodulin-1 OS=Mus musculus GN=Tmod1 PE=1 SV=2	4.178273	1	3	1	40.441	5.1	2100000	1300000	2000000	11.39
Q99K85	Phosphoserine aminotransferase OS=Mus musculus GN=Psat1 PE=1 SV=1	9.4594595	3	5	3	40.447	8.03	1200000	1700000	2100000	13.18
Q9QUP5	Hyaluronan and proteoglycan link protein 1 OS=Mus musculus GN=Hapln1 PE=1 SV=1	30.898876	10	26	10	40.452	7.8	13000000	8800000	21000000	68.13
P08752	Guanine nucleotide-binding protein G(i) subunit alpha-2 OS=Mus musculus GN=Gnai2 PE	36.056338	11	44	6	40.463	5.45	18000000	9000000	17000000	137.58
Q8VHL1	Histone-lysine N-methyltransferase SETD7 OS=Mus musculus GN=Setd7 PE=1 SV=2	9.2896175	2	3	2	40.481	4.65	500000		2000000	6.94

Q9DC51	Guanine nucleotide-binding protein G(k) subunit alpha OS=Mus musculus GN=Gnai3 PE=		15.536723	5	20	1	40.512	5.69	1300000		1100000	52.84
Q99KV1	DnaJ homolog subfamily B member 11 OS=Mus musculus GN=Dnajb11 PE=1 SV=1		22.067039	5	16	5	40.53	6.32	4400000	3600000	3400000	46.22
Q9QWW1	Homer protein homolog 2 OS=Mus musculus GN=Homer2 PE=1 SV=1		5.9322034	2	6	1	40.545	6.01	830000	570000	250000	16.84
P05132	cAMP-dependent protein kinase catalytic subunit alpha OS=Mus musculus GN=Prkaca PI		17.378917	6	11	1	40.545	8.79	2300000	890000	1100000	31.36
Q99LC3	NADH dehydrogenase [ubiquinone] 1 alpha subcomplex subunit 10, mitochondrial OS=Mu		27.323944	10	30	10	40.578	7.78	29000000	25000000	41000000	79.89
Q8BIF2	RNA binding protein fox-1 homolog 3 OS=Mus musculus GN=Rbfox3 PE=1 SV=2		2.4064171	1	2	1	40.585	7.9	1700000		2100000	4.99
Q9D4C9	Clavesin-1 OS=Mus musculus GN=Clvs1 PE=1 SV=1		3.6723164	1	1	1	40.588	6.73	1100000			2.19
O35226	26S proteasome non-ATPase regulatory subunit 4 OS=Mus musculus GN=Psmd4 PE=1 S		10.37234	2	5	2	40.678	4.79	6100000	2500000	7000000	16.55
P68181	cAMP-dependent protein kinase catalytic subunit beta OS=Mus musculus GN=Prkacb PE		22.222222	7	11	2	40.682	8.56		1500000	2000000	26.20
Q9CZ44	NSFL1 cofactor p47 OS=Mus musculus GN=Nsfl1c PE=1 SV=1		39.189189	10	26	10	40.685	5.15	4900000	3600000	6700000	81.37
Q9CR16	Peptidyl-prolyl cis-trans isomerase D OS=Mus musculus GN=Ppid PE=1 SV=3		18.918919	6	13	6	40.717	7.43	4500000	2200000	2500000	34.40
Q9D5T0	ATPase family AAA domain-containing protein 1 OS=Mus musculus GN=Atad1 PE=1 SV=		12.742382	2	4	2	40.718	6.9		600000	2300000	10.94
Q9QYG0	Protein NDRG2 OS=Mus musculus GN=Ndrg2 PE=1 SV=1		16.442049	4	25	4	40.763	5.4	9800000	9000000	17000000	97.67
O88507	Ciliary neurotrophic factor receptor subunit alpha OS=Mus musculus GN=Cntfr PE=1 SV=		3.4946237	1	1	1	40.776	6.83		460000		0.00
O70443	Guanine nucleotide-binding protein G(z) subunit alpha OS=Mus musculus GN=Gnaz PE=		20.28169	6	14	6	40.824	7.61	8500000	2000000	6300000	39.02
Q8R1N4	NudC domain-containing protein 3 OS=Mus musculus GN=Nudcd3 PE=1 SV=3		4.1322314	1	1	1	40.865	5.26		730000		2.56
Q8BG18	N-terminal EF-hand calcium-binding protein 1 OS=Mus musculus GN=Necab1 PE=1 SV=		9.9431818	2	10	2	40.908	4.89	2100000	1000000	1900000	30.28
Q64314	Hematopoietic progenitor cell antigen CD34 OS=Mus musculus GN=Cd34 PE=1 SV=1		7.8534031	3	7	3	40.957	5.3	7500000	4300000	5900000	17.06
P62482	Voltage-gated potassium channel subunit beta-2 OS=Mus musculus GN=Kcnab2 PE=1 S		10.899183	4	6	4	40.995	9	5400000	1900000	2700000	15.00
Q8VEK0	Cell cycle control protein 50A OS=Mus musculus GN=Tmem30a PE=1 SV=1		16.483516	5	15	5	41.035	8.37	10000000	7300000	8400000	56.71
Q9CXW2	28S ribosomal protein S22, mitochondrial OS=Mus musculus GN=Mrps22 PE=1 SV=1		9.1922006	3	5	3	41.167	8.56	990000	490000	1300000	15.07
Q9JI19	Acidic fibroblast growth factor intracellular-binding protein OS=Mus musculus GN=Fibp PE		2.5210084	1	2	1	41.178	6.76	1000000	450000		3.92
Q9R0A0	Peroxisomal membrane protein PEX14 OS=Mus musculus GN=Pex14 PE=1 SV=1		3.1914894	1	2	1	41.183	5.11	2400000		1800000	5.51
O54833	Casein kinase II subunit alpha' OS=Mus musculus GN=Csnk2a2 PE=1 SV=1		5.7142857	1	2	1	41.189	8.56	1400000		1100000	6.64
P63085	Mitogen-activated protein kinase 1 OS=Mus musculus GN=Mapk1 PE=1 SV=3		25.418994	7	18	5	41.249	6.98	14000000	7400000	11000000	52.71
Q3UM45	Protein phosphatase 1 regulatory subunit 7 OS=Mus musculus GN=Ppp1r7 PE=1 SV=2		13.296399	3	4	3	41.266	4.92	1900000		1000000	11.36
O88741	Ganglioside-induced differentiation-associated protein 1 OS=Mus musculus GN=Gdap1 P		12.569832	4	6	4	41.285	8.37	11000000	7100000	14000000	21.65
Q8C163	Nuclease EXOG, mitochondrial OS=Mus musculus GN=Exog PE=1 SV=1		17.391304	4	10	4	41.358	8.12	3900000	2700000	2600000	30.90
Q9Z2Y3	Homer protein homolog 1 OS=Mus musculus GN=Homer1 PE=1 SV=2		42.349727	15	36	14	41.388	5.53	12000000	5200000	16000000	100.56
Q62419	Endophilin-A2 OS=Mus musculus GN=Sh3gl1 PE=1 SV=1		25.543478	8	22	4	41.492	5.72	3500000	830000	2700000	65.90
P42208	Septin-2 OS=Mus musculus GN=Sept2 PE=1 SV=2		24.65374	6	11	6	41.499	6.55	2300000	1500000	1800000	31.24
Q9QYF9	Protein NDRG3 OS=Mus musculus GN=Ndrg3 PE=1 SV=1		16.533333	4	12	4	41.529	5.25	2400000	1200000	1800000	36.39
Q791T5	Mitochondrial carrier homolog 1 OS=Mus musculus GN=Mtch1 PE=1 SV=1		6.1696658	2	2	2	41.538	9.32	1400000		840000	4.59
Q9Z0P4	Paralemmin-1 OS=Mus musculus GN=Palm PE=1 SV=1		42.29765	12	56	12	41.589	4.84	46000000	22000000	46000000	193.33
Q9R0Q6	Actin-related protein 2/3 complex subunit 1A OS=Mus musculus GN=Arpc1a PE=1 SV=1		22.702703	7	9	7	41.6	8.18	5200000	4600000	6500000	26.64
Q99L47	Hsc70-interacting protein OS=Mus musculus GN=St13 PE=1 SV=1		18.867925	6	17	6	41.629	5.26	15000000	7100000	12000000	52.45
Q924L1	LETM1 domain-containing protein 1 OS=Mus musculus GN=Letmd1 PE=1 SV=1		2.7777778	1	2	1	41.674	10.54	750000		610000	4.26
Q3UXZ6	Protein FAM81A OS=Mus musculus GN=Fam81a PE=1 SV=2		12.362637	4	7	4	41.684	8.75	2000000	940000	1700000	18.09
P60710	Actin, cytoplasmic 1 OS=Mus musculus GN=Actb PE=1 SV=1	x	71.466667	25	260	12	41.71	5.48	600000000	800000000	1.4E+09	766.25
Q80UY2	E3 ubiquitin-protein ligase KCMF1 OS=Mus musculus GN=Kcmf1 PE=1 SV=1		2.8871391	1	1	1	41.765	5.76			150000	2.02
P97441	Zinc transporter 3 OS=Mus musculus GN=Slc30a3 PE=1 SV=1		14.690722	5	11	5	41.797	6.38	16000000	5800000	9200000	30.00
Q8BWT1	3-ketoacyl-CoA thiolase, mitochondrial OS=Mus musculus GN=Acaa2 PE=1 SV=3		15.11335	4	12	4	41.803	8.09	4500000	1600000	2700000	36.65
Q91VA6	Polymerase delta-interacting protein 2 OS=Mus musculus GN=Poldip2 PE=1 SV=1		3.2608696	1	2	1	41.844	8.63		440000	750000	5.02
Q811Q9	Choline-phosphate cytidylyltransferase B OS=Mus musculus GN=Pcyt1b PE=1 SV=2		3.2520325	1	2	1	41.874	6.4	1200000		760000	4.91
Q9QYI4	DnaJ homolog subfamily B member 12 OS=Mus musculus GN=Dnajb12 PE=1 SV=2		7.712766	2	3	2	41.962	8.51	870000	880000	490000	7.76
P68033	Actin, alpha cardiac muscle 1 OS=Mus musculus GN=Actc1 PE=1 SV=1		40.318302	16	194	3	41.992	5.39	350000000	150000000	400000000	510.64
P21278	Guanine nucleotide-binding protein subunit alpha-11 OS=Mus musculus GN=Gna11 PE=		16.713092	5	14	2	41.997	5.97	4500000	2600000	7100000	34.97
Q9JKV1	Proteasomal ubiquitin receptor ADRM1 OS=Mus musculus GN=Adrm1 PE=1 SV=2		3.9312039	1	4	1	42.034	5.07	2900000	1900000	2300000	11.76

Q8BR92	Paralemmin-2 OS=Mus musculus GN=Palm2 PE=1 SV=1	6.9148936	2	4	2	42.069	5.15	8100000	3000000	4300000	11.50
O35188	Fractalkine OS=Mus musculus GN=Cx3cl1 PE=1 SV=3	4.556962	1	1	1	42.072	5.54				3.92
Q9DCE5	p21-activated protein kinase-interacting protein 1 OS=Mus musculus GN=Pak1ip1 PE=1 S	4.4502618	1	1	1	42.089	8.47				2.49
P15105	Glutamine synthetase OS=Mus musculus GN=Glul PE=1 SV=6	41.286863	14	55	14	42.092	7.08	62000000	40000000	54000000	177.06
P21279	Guanine nucleotide-binding protein G(q) subunit alpha OS=Mus musculus GN=Gnaq PE=	34.818942	10	33	7	42.131	5.68	17000000	11000000	17000000	90.01
Q8BHE3	Caytaxin OS=Mus musculus GN=Atcay PE=1 SV=1	21.505376	5	11	5	42.152	4.68	5500000	1700000	3900000	26.15
P55302	Alpha-2-macroglobulin receptor-associated protein OS=Mus musculus GN=Lrpap1 PE=1	14.166667	3	5	3	42.189	7.87	2300000	1900000	3300000	15.99
Q8BVQ5	Protein phosphatase methylesterase 1 OS=Mus musculus GN=Ppme1 PE=1 SV=5	11.917098	3	10	3	42.229	5.97	1900000	770000	2100000	31.74
Q8R5C5	Beta-centractin OS=Mus musculus GN=Actr1b PE=1 SV=1	20.744681	6	10	2	42.255	6.4	19000000	5200000	10000000	31.76
Q9WV02	RNA-binding motif protein, X chromosome OS=Mus musculus GN=Rbmx PE=1 SV=1	13.043478	4	8	4	42.275	10.05	11000000	3100000	7100000	24.03
Q8CGC4	Protein LSM14 homolog B OS=Mus musculus GN=Lsm14b PE=1 SV=3	2.8571429	1	1	1	42.284	9.63	1600000			2.42
Q8VE33	Ganglioside-induced differentiation-associated protein 1-like 1 OS=Mus musculus GN=Gc	7.2972973	2	4	2	42.291	6.6	7800000	5700000	9400000	14.56
Q8BGT8	Phytanoyl-CoA hydroxylase-interacting protein-like OS=Mus musculus GN=Phyhipl PE=1	5.0666667	1	1	1	42.314	6.35			780000	2.93
P49443	Protein phosphatase 1A OS=Mus musculus GN=Ppm1a PE=1 SV=1	15.445026	5	13	5	42.406	5.36	3700000	3400000	4600000	33.42
Q9JIF0	Protein arginine N-methyltransferase 1 OS=Mus musculus GN=Prmt1 PE=1 SV=1	10.781671	4	11	2	42.408	5.43	3300000	1500000	4900000	29.25
P62069	Ubiquitin carboxyl-terminal hydrolase 46 OS=Mus musculus GN=Usp46 PE=1 SV=1	3.5519126	1	1	1	42.415	6.83				1.84
P18572	Basigin OS=Mus musculus GN=Bsg PE=1 SV=2	18.766067	7	23	7	42.418	5.85	30000000	35000000	48000000	73.73
Q9DC69	NADH dehydrogenase [ubiquinone] 1 alpha subcomplex subunit 9, mitochondrial OS=Mus	15.915119	4	11	4	42.498	9.74	10000000	5500000	8800000	35.70
Q91V12	Cytosolic acyl coenzyme A thioester hydrolase OS=Mus musculus GN=Acot7 PE=1 SV=2	3.1496063	1	2	1	42.51	8.68		890000		5.39
Q99MQ4	Asporin OS=Mus musculus GN=Aspn PE=1 SV=1	2.6809651	1	1	1	42.545	8.57		1000000		2.59
P61164	Alpha-centractin OS=Mus musculus GN=Actr1a PE=1 SV=1	14.62766	5	8	1	42.587	6.64	2500000	1600000	2400000	21.44
Q8BHS6	Armadillo repeat-containing X-linked protein 3 OS=Mus musculus GN=Armcx3 PE=1 SV=	4.4854881	1	2	1	42.593	8.68	820000			6.25
Q78ZA7	Nucleosome assembly protein 1-like 4 OS=Mus musculus GN=Nap1l4 PE=1 SV=1	12.533333	3	5	2	42.653	4.67	2500000	2300000	2600000	18.16
Q04447	Creatine kinase B-type OS=Mus musculus GN=Ckb PE=1 SV=1	44.88189	14	74	14	42.686	5.67	130000000	53000000	120000000	263.58
Q8R464	Cell adhesion molecule 4 OS=Mus musculus GN=Cadm4 PE=1 SV=1	18.298969	4	14	4	42.697	6.3	5100000	4200000	6200000	58.65
Q9Z2Q6	Septin-5 OS=Mus musculus GN=Sept5 PE=1 SV=2	43.631436	15	53	14	42.721	6.67	43000000	19000000	41000000	164.61
Q99J95	Cyclin-dependent kinase 9 OS=Mus musculus GN=Cdk9 PE=1 SV=1	5.9139785	2	2	2	42.734	8.79	610000			4.40
P70404	Isocitrate dehydrogenase [NAD] subunit gamma 1, mitochondrial OS=Mus musculus GN=	23.664122	5	14	5	42.758	9.01	16000000	5200000	17000000	47.77
Q80WM4	Hyaluronan and proteoglycan link protein 4 OS=Mus musculus GN=Hapln4 PE=2 SV=2	4	1	3	1	42.782	8.85	910000	410000	490000	9.92
Q9WVJ2	26S proteasome non-ATPase regulatory subunit 13 OS=Mus musculus GN=Psmd13 PE=	8.2446809	3	7	3	42.782	5.71	2800000	2400000	3700000	16.62
Q99M51	Cytoplasmic protein NCK1 OS=Mus musculus GN=Nck1 PE=1 SV=1	6.8965517	2	2	2	42.863	6.47	2000000		2900000	4.77
Q8BW96	Calcium/calmodulin-dependent protein kinase type 1D OS=Mus musculus GN=Camk1d P	4.6753247	1	2	1	42.892	7.17	2400000		3100000	5.88
Q99N28	Cell adhesion molecule 3 OS=Mus musculus GN=Cadm3 PE=1 SV=1	26.262626	7	28	7	42.938	5.8	28000000	21000000	34000000	108.14
P23242	Gap junction alpha-1 protein OS=Mus musculus GN=Gja1 PE=1 SV=2	14.921466	3	12	3	42.977	8.76	7200000	980000	6500000	38.91
Q62433	Protein NDRG1 OS=Mus musculus GN=Ndrg1 PE=1 SV=1	3.5532995	1	1	1	42.981	6.1	1400000			2.75
P07310	Creatine kinase M-type OS=Mus musculus GN=Ckm PE=1 SV=1	2.6246719	1	1	1	43.018	7.06	1200000			2.15
Q9CXY6	Interleukin enhancer-binding factor 2 OS=Mus musculus GN=Ilf2 PE=1 SV=1	11.025641	3	8	3	43.035	5.26	1900000	1400000	1500000	22.74
Q63844	Mitogen-activated protein kinase 3 OS=Mus musculus GN=Mapk3 PE=1 SV=5	12.631579	5	9	3	43.039	6.61	1500000	980000	1200000	23.22
Q62465	Synaptic vesicle membrane protein VAT-1 homolog OS=Mus musculus GN=Vat1 PE=1 S	9.8522167	3	5	3	43.069	6.37	1700000	1000000		12.75
Q8K0G5	Protein TSSC1 OS=Mus musculus GN=Eipr1 PE=1 SV=2	4.9222798	1	1	1	43.1	5.14			3700000	5.12
Q9R0N5	Synaptotagmin-5 OS=Mus musculus GN=Syt5 PE=1 SV=1	8.2901554	4	14	1	43.103	9.6			3800000	43.95
Q8K2C9	Very-long-chain (3R)-3-hydroxyacyl-CoA dehydratase 3 OS=Mus musculus GN=Hacd3 P	8.5635359	3	5	3	43.103	9.13	4300000	1900000	3600000	13.54
Q7TNV0	Protein DEK OS=Mus musculus GN=Dek PE=1 SV=1	7.1052632	2	3	2	43.132	6.86	3400000		1700000	7.43
Q9DBC7	cAMP-dependent protein kinase type I-alpha regulatory subunit OS=Mus musculus GN=P	16.7979	5	15	4	43.158	5.35	7200000	4100000	6200000	41.54
Q6NTA4	Ras-related GTP-binding protein B OS=Mus musculus GN=Rragb PE=1 SV=1	2.6737968	1	1	1	43.164	6.38	2100000			2.48
P00158	Cytochrome b OS=Mus musculus GN=Mt-Cyb PE=1 SV=1	2.3622047	1	1	1	43.181	7.97		1600000		2.29
P12849	cAMP-dependent protein kinase type I-beta regulatory subunit OS=Mus musculus GN=Prl	14.173228	4	12	3	43.197	5.96	4700000	1800000	4500000	31.22
P35486	Pyruvate dehydrogenase E1 component subunit alpha, somatic form, mitochondrial OS=M	42.051282	17	56	17	43.204	8.19	60000000	32000000	54000000	152.20

Q8VDQ8	NAD-dependent protein deacetylase sirtuin-2 OS=Mus musculus GN=Sirt2 PE=1 SV=2	17.48072	6	11	6	43.228	5.35	7300000	3600000	5600000	39.68
Q9D517	1-acyl-sn-glycerol-3-phosphate acyltransferase gamma OS=Mus musculus GN=Agpat3 P	5.0531915	1	1	1	43.268	8.51	1000000			2.52
Q8BWM0	Prostaglandin E synthase 2 OS=Mus musculus GN=Ptges2 PE=1 SV=3	3.3854167	1	1	1	43.296	9			560000	2.34
Q80X73	Protein pelota homolog OS=Mus musculus GN=Pelo PE=1 SV=3	3.6363636	1	1	1	43.322	5.99			210000	2.81
Q91VL8	Telomeric repeat-binding factor 2-interacting protein 1 OS=Mus musculus GN=Terf2ip PE	4.0712468	1	1	1	43.326	4.81	280000			2.51
Q8VHW2	Voltage-dependent calcium channel gamma-8 subunit OS=Mus musculus GN=Cacng8 PE	8.9834515	2	4	2	43.426	9.2	4200000	1100000	3200000	14.57
P31938	Dual specificity mitogen-activated protein kinase kinase 1 OS=Mus musculus GN=Map2k	14.75827	5	9	5	43.446	6.7	3600000	1600000	2900000	31.72
P54728	UV excision repair protein RAD23 homolog B OS=Mus musculus GN=Rad23b PE=1 SV=2	2.1634615	1	2	1	43.486	4.83		1600000	3100000	3.77
O35465	Peptidyl-prolyl cis-trans isomerase FKBP8 OS=Mus musculus GN=Fkbp8 PE=1 SV=2	11.19403	3	12	3	43.501	5.16	4000000	1700000	4000000	41.04
P62881	Guanine nucleotide-binding protein subunit beta-5 OS=Mus musculus GN=Gnb5 PE=1 SV	26.582278	8	25	8	43.537	6.46	9300000	5500000	11000000	74.61
Q3THS6	S-adenosylmethionine synthase isoform type-2 OS=Mus musculus GN=Mat2a PE=1 SV=2	5.5696203	2	3	2	43.661	6.48	1800000	560000		9.13
P50580	Proliferation-associated protein 2G4 OS=Mus musculus GN=Pa2g4 PE=1 SV=3	14.974619	6	15	6	43.671	6.86	5800000	2500000	4700000	38.77
Q8K4X7	1-acyl-sn-glycerol-3-phosphate acyltransferase delta OS=Mus musculus GN=Agpat4 PE=	5.5555556	2	2	2	43.781	8.38	2200000			4.43
P11438	Lysosome-associated membrane glycoprotein 1 OS=Mus musculus GN=Lamp1 PE=1 SV	8.3743842	3	11	3	43.837	8.4	3800000	4200000	5900000	30.28
Q61733	28S ribosomal protein S31, mitochondrial OS=Mus musculus GN=Mrps31 PE=1 SV=1	3.3854167	1	3	1	43.854	8.51	1000000	960000	1100000	8.18
Q9Z1G3	V-type proton ATPase subunit C 1 OS=Mus musculus GN=Atp6v1c1 PE=1 SV=4	27.748691	12	36	12	43.86	7.46	19000000	9100000	19000000	93.27
Q9CYI4	Putative RNA-binding protein Luc7-like 1 OS=Mus musculus GN=Luc7l PE=1 SV=2	7.277628	2	2	1	43.907	9.88			260000	5.83
Q921H8	3-ketoacyl-CoA thiolase A, peroxisomal OS=Mus musculus GN=Acaa1a PE=1 SV=1	3.3018868	1	3	1	43.926	8.44	2700000	1200000	1700000	7.71
O54724	Polymerase I and transcript release factor OS=Mus musculus GN=Ptrf PE=1 SV=1	13.520408	4	9	4	43.927	5.52	1300000	350000	1500000	23.42
P48543	G protein-activated inward rectifier potassium channel 3 OS=Mus musculus GN=Kcnj9 PE	4.5801527	1	2	1	43.946	5	170000		390000	5.46
Q80YQ8	Protein RMD5 homolog A OS=Mus musculus GN=Rmnd5a PE=1 SV=2	2.8132992	1	1	1	43.964	6.06	670000			2.04
P27601	Guanine nucleotide-binding protein subunit alpha-13 OS=Mus musculus GN=Gna13 PE=	15.119363	5	13	4	44.027	8.21	4100000	250000	3400000	29.58
P47809	Dual specificity mitogen-activated protein kinase kinase 4 OS=Mus musculus GN=Map2k	7.3047859	2	3	2	44.085	8.07	1700000	450000		6.97
Q99KJ8	Dynactin subunit 2 OS=Mus musculus GN=Dctn2 PE=1 SV=3	30.348259	8	19	8	44.09	5.26	5000000	3100000	5900000	71.15
Q99K70	Ras-related GTP-binding protein C OS=Mus musculus GN=Rragc PE=1 SV=1	9.5477387	3	8	3	44.093	5.1	2500000	1300000	2100000	24.36
Q61187	Tumor susceptibility gene 101 protein OS=Mus musculus GN=Tsg101 PE=1 SV=2	11.764706	4	7	4	44.096	6.71	1900000	870000	1000000	18.37
Q9Z0H3	SWI/SNF-related matrix-associated actin-dependent regulator of chromatin subfamily B m	8.5714286	2	8	2	44.113	6.23	1400000	470000	1300000	24.53
P62334	26S protease regulatory subunit 10B OS=Mus musculus GN=Psmc6 PE=1 SV=1	12.85347	4	8	4	44.145	7.49	2400000	700000	1300000	20.59
Q91Y86	Mitogen-activated protein kinase 8 OS=Mus musculus GN=Mapk8 PE=1 SV=1	7.8125	3	4	3	44.201	7.69	1400000	1200000	2100000	8.76
P70429	Ena/VASP-like protein OS=Mus musculus GN=Evl PE=1 SV=2	12.318841	3	6	3	44.31	8.85	1800000	610000	1400000	24.50
Q8QZY9	Splicing factor 3B subunit 4 OS=Mus musculus GN=Sf3b4 PE=1 SV=1	3.3018868	1	2	1	44.327	8.56		1700000	2100000	6.45
P97300	Neuroplastin OS=Mus musculus GN=Nptn PE=1 SV=3	29.722922	13	67	13	44.345	7.74	110000000	96000000	130000000	191.79
Q8R3V5	Endophilin-B2 OS=Mus musculus GN=Sh3glb2 PE=1 SV=2	13.25	5	11	5	44.476	5.82	5900000	2900000	7800000	31.05
Q921F2	TAR DNA-binding protein 43 OS=Mus musculus GN=Tardbp PE=1 SV=1	23.188406	6	14	6	44.519	6.7	6100000	2000000	5300000	39.45
P09411	Phosphoglycerate kinase 1 OS=Mus musculus GN=Pgk1 PE=1 SV=4	38.609113	13	33	13	44.522	7.9	14000000	6500000	10000000	101.99
A2ADY9	Protein DDI1 homolog 2 OS=Mus musculus GN=Ddi2 PE=1 SV=1	2.2556391	1	1	1	44.562	5.05			910000	2.42
Q61081	Hsp90 co-chaperone Cdc37 OS=Mus musculus GN=Cdc37 PE=1 SV=1	13.192612	4	9	4	44.565	5.34	4800000	4100000	8600000	27.92
Q5HZI2	C2 calcium-dependent domain-containing protein 4C OS=Mus musculus GN=C2cd4cC2C	3.3412888	1	2	1	44.587	9.73	1400000		750000	4.73
Q9WV54	Acid ceramidase OS=Mus musculus GN=Asah1 PE=1 SV=1	7.6142132	3	5	3	44.641	8.46	3600000	2800000		13.33
Q9CY58	Plasminogen activator inhibitor 1 RNA-binding protein OS=Mus musculus GN=Serbp1 PE	20.14742	5	10	5	44.687	8.54	4200000	250000	1900000	30.55
P61161	Actin-related protein 2 OS=Mus musculus GN=Actr2 PE=1 SV=1	21.319797	7	28	7	44.732	6.74	13000000	10000000	16000000	83.70
Q8VE47	Ubiquitin-like modifier-activating enzyme 5 OS=Mus musculus GN=Uba5 PE=1 SV=2	1.9851117	1	1	1	44.761	4.96	580000			1.99
Q8VD63	Testis-specific Y-encoded-like protein 4 OS=Mus musculus GN=Tspyl4 PE=1 SV=1	3.9408867	1	2	1	44.783	6.99	1300000		930000	4.85
Q8QZT1	Acetyl-CoA acetyltransferase, mitochondrial OS=Mus musculus GN=Acat1 PE=1 SV=1	28.301887	9	37	9	44.787	8.51	20000000	15000000	18000000	124.68
P63037	DnaJ homolog subfamily A member 1 OS=Mus musculus GN=Dnaja1 PE=1 SV=1	10.327456	3	11	3	44.839	7.08	6900000	2800000	5100000	39.53
Q9JMC3	DnaJ homolog subfamily A member 4 OS=Mus musculus GN=Dnaja4 PE=1 SV=1	2.7707809	1	3	1	44.873	7.58	1200000	470000	1300000	7.00
Q9D7N3	28S ribosomal protein S9, mitochondrial OS=Mus musculus GN=Mrps9 PE=1 SV=3	2.8205128	1	1	1	44.901	8.81	900000			2.55
Q8VE37	Regulator of chromosome condensation OS=Mus musculus GN=Rcc1 PE=1 SV=1	1.9002375	1	1	1	44.903	8.1			410000	1.93

P18242	Cathepsin D OS=Mus musculus GN=Ctsd PE=1 SV=1		11.219512	3	11	3	44.925	7.15	5800000	2000000	5600000	32.04
Q9D7B6	Isobutyryl-CoA dehydrogenase, mitochondrial OS=Mus musculus GN=Acad8 PE=1 SV=2		2.1791768	1	1	1	44.99	8.13	720000			2.04
Q3URS9	Coiled-coil domain-containing protein 51 OS=Mus musculus GN=Ccdc51 PE=1 SV=1		7.3891626	2	2	2	45.104	8.09		360000	230000	6.16
Q60737	Casein kinase II subunit alpha OS=Mus musculus GN=Csnk2a1 PE=1 SV=2		30.179028	9	25	9	45.105	7.74	9000000	6600000	9300000	71.69
Q80TL4	PHD finger protein 24 OS=Mus musculus GN=Phf24 PE=1 SV=2		33.25	10	32	10	45.194	5.77	21000000	10000000	23000000	109.01
Q922E4	Ethanolamine-phosphate cytidylyltransferase OS=Mus musculus GN=Pcyt2 PE=1 SV=1		2.2277228	1	1	1	45.207	6.58			880000	2.78
Q99LR1	Monoacylglycerol lipase ABHD12 OS=Mus musculus GN=Abhd12 PE=1 SV=2		19.346734	5	12	5	45.241	8.72	3900000	1300000	2500000	38.07
Q6PAK3	Protein arginine N-methyltransferase 8 OS=Mus musculus GN=Prmt8 PE=1 SV=2		7.106599	3	8	1	45.247	6.93		960000	1900000	21.08
Q9EPJ9	ADP-ribosylation factor GTPase-activating protein 1 OS=Mus musculus GN=Arfgap1 PE=		34.299517	7	19	7	45.26	5.57	14000000	4900000	12000000	70.69
Q8JZX4	Splicing factor 45 OS=Mus musculus GN=Rbm17 PE=1 SV=1		2.962963	1	1	1	45.276	5.82			390000	2.47
Q9WV31	Activity-regulated cytoskeleton-associated protein OS=Mus musculus GN=Arc PE=1 SV=		4.040404	1	2	1	45.293	5.39	1500000		1500000	6.61
O89112	LanC-like protein 1 OS=Mus musculus GN=Lancl1 PE=1 SV=1		6.2656642	2	4	2	45.312	7.77	5700000	2900000	5500000	10.48
P28656	Nucleosome assembly protein 1-like 1 OS=Mus musculus GN=Nap1l1 PE=1 SV=2		7.4168798	2	4	1	45.317	4.46	2200000	1000000	2500000	10.68
Q80VD1	Protein FAM98B OS=Mus musculus GN=Fam98b PE=1 SV=1		2.7972028	1	3	1	45.321	8.5	2400000	650000	1700000	7.86
P12367	cAMP-dependent protein kinase type II-alpha regulatory subunit OS=Mus musculus GN=F		31.421446	10	27	8	45.361	4.89	16000000	5700000	17000000	79.45
Q9WV69	Dematin OS=Mus musculus GN=Dmtn PE=1 SV=1		14.567901	5	13	5	45.44	8.41	4500000	1200000	8500000	35.65
Q9R0N7	Synaptotagmin-7 OS=Mus musculus GN=Syt7 PE=1 SV=1		7.1960298	2	4	2	45.444	9.28	5100000	2300000	5500000	10.09
Q99JI4	26S proteasome non-ATPase regulatory subunit 6 OS=Mus musculus GN=Psmd6 PE=1 S		8.9974293	3	6	3	45.507	5.52	2600000	1200000	1400000	16.43
Q8BU14	Translocation protein SEC62 OS=Mus musculus GN=Sec62 PE=1 SV=1		2.2613065	1	1	1	45.552	7.31	1100000			2.53
P62196	26S protease regulatory subunit 8 OS=Mus musculus GN=Psmc5 PE=1 SV=1		15.763547	5	10	5	45.597	7.55	3000000	1700000	2100000	29.90
P17047	Lysosome-associated membrane glycoprotein 2 OS=Mus musculus GN=Lamp2 PE=1 SV		1.9277108	1	2	1	45.652	7.39	1900000		2800000	4.41
Q8VE62	Polyadenylate-binding protein-interacting protein 1 OS=Mus musculus GN=Paip1 PE=1 S		2.75	1	1	1	45.673	4.55	1100000			2.07
Q91XD7	Cysteine-rich with EGF-like domain protein 1 OS=Mus musculus GN=Creld1 PE=1 SV=1		14.761905	6	11	6	45.687	5.02	3500000	2700000	5200000	31.49
Q9Z2X1	Heterogeneous nuclear ribonucleoprotein F OS=Mus musculus GN=Hnrnpf PE=1 SV=3		15.903614	4	5	3	45.701	5.49		760000	4900000	17.69
Q8BLF1	Neutral cholesterol ester hydrolase 1 OS=Mus musculus GN=Nceh1 PE=1 SV=1		5.3921569	2	6	2	45.711	7.05	14000000	5900000	9200000	16.80
Q9QYJ0	DnaJ homolog subfamily A member 2 OS=Mus musculus GN=Dnaja2 PE=1 SV=1		27.669903	8	14	8	45.717	6.48	4900000	2400000	5600000	41.60
Q5DU31	Interactor protein for cytohesin exchange factors 1 OS=Mus musculus GN=Ipcef1 PE=1 S		3.6945813	1	1	1	45.784	6.73			660000	3.59
Q923Z0	G-protein coupled receptor family C group 5 member B OS=Mus musculus GN=Gprc5b P		2.9268293	1	1	1	45.869	8.38	1900000			3.24
Q99KU0	Vacuole membrane protein 1 OS=Mus musculus GN=Vmp1 PE=1 SV=2		3.9408867	1	2	1	45.931	6.95	2700000			3.09
Q64519	Syndecan-3 OS=Mus musculus GN=Sdc3 PE=1 SV=2		2.4886878	1	3	1	45.973	4.59	4200000	4500000	5200000	8.39
P07758	Alpha-1-antitrypsin 1-1 OS=Mus musculus GN=Serpina1a PE=1 SV=4		4.6004843	1	2	1	45.974	5.72	870000	820000		8.05
Q9JK42	[Pyruvate dehydrogenase (acetyl-transferring)] kinase isozyme 2, mitochondrial OS=Mus		13.022113	4	7	4	46.011	6.61	2200000	1700000	1500000	19.93
P27659	60S ribosomal protein L3 OS=Mus musculus GN=Rpl3 PE=1 SV=3		13.399504	5	9	5	46.081	10.21	15000000	2100000	4100000	25.04
O70172	Phosphatidylinositol 5-phosphate 4-kinase type-2 alpha OS=Mus musculus GN=Pip4k2a I		15.061728	5	15	2	46.122	6.99	7300000	5400000	4600000	41.71
P60843	Eukaryotic initiation factor 4A-I OS=Mus musculus GN=Eif4a1 PE=1 SV=1		18.719212	8	27	4	46.125	5.48	4300000	1500000	2600000	73.31
P31324	cAMP-dependent protein kinase type II-beta regulatory subunit OS=Mus musculus GN=Pr		38.461538	12	40	10	46.138	4.98	20000000	8000000	20000000	140.74
Q9QZB7	Actin-related protein 10 OS=Mus musculus GN=Actr10 PE=1 SV=2		9.352518	2	2	2	46.178	7.61	2400000		360000	5.83
E9Q4P1	WD repeat and FYVE domain-containing protein 1 OS=Mus musculus GN=Wdfy1 PE=1 S		3.4146341	1	1	1	46.187	7.34	2800000			3.36
Q9CQS4	Solute carrier family 25 member 46 OS=Mus musculus GN=Slc25a46 PE=1 SV=1		2.3923445	1	1	1	46.195	7.64	2800000			2.67
P05201	Aspartate aminotransferase, cytoplasmic OS=Mus musculus GN=Got1 PE=1 SV=3		29.539952	10	24	10	46.219	7.14	10000000	4400000	8300000	66.57
Q9QX11	Cytohesin-1 OS=Mus musculus GN=Cyth1 PE=1 SV=2		11.557789	3	4	2	46.244	5.63			520000	11.52
O08967	Cytohesin-3 OS=Mus musculus GN=Cyth3 PE=1 SV=1		8.0200501	2	3	1	46.25	5.54	2400000			10.18
O88544	COP9 signalosome complex subunit 4 OS=Mus musculus GN=Cops4 PE=1 SV=1		9.1133005	3	8	3	46.256	5.83	1600000	1100000	2100000	23.39
Q8R0A7	Uncharacterized protein KIAA0513 OS=Mus musculus GN=Kiaa0513 PE=1 SV=1		16.461916	6	13	6	46.289	5.02	15000000	4800000	14000000	37.48
Q9JHI5	Isovaleryl-CoA dehydrogenase, mitochondrial OS=Mus musculus GN=Ivd PE=1 SV=1		10.141509	4	10	4	46.296	8.29	3600000	2300000	3800000	28.59
Q9CZC8	Secernin-1 OS=Mus musculus GN=Scrn1 PE=1 SV=1		32.608696	11	29	11	46.297	4.79	7900000	5100000	12000000	83.64
Q640R3	Hepatocyte cell adhesion molecule OS=Mus musculus GN=Hepacam PE=1 SV=2		4.5454545	1	3	1	46.338	9.42	6100000	1800000	3600000	9.59
P10630	Eukaryotic initiation factor 4A-II OS=Mus musculus GN=Eif4a2 PE=1 SV=2		22.604423	8	27	4	46.373	5.48	7600000	2400000	4300000	73.87

Q91W90	Thioredoxin domain-containing protein 5 OS=Mus musculus GN=Txndc5 PE=1 SV=2	3.8369305	1	4	1	46.386	5.78	970000	1100000	1900000	14.83
Q9D7N9	Adipocyte plasma membrane-associated protein OS=Mus musculus GN=Apmap PE=1 SV	5.060241	2	2	2	46.405	6.32	3800000	4500000		5.24
P45952	Medium-chain specific acyl-CoA dehydrogenase, mitochondrial OS=Mus musculus GN=A	5.7007126	2	5	2	46.452	8.37	3600000	2300000	3400000	13.27
Q8R570	Synaptosomal-associated protein 47 OS=Mus musculus GN=Snap47 PE=1 SV=1	23.244552	7	19	7	46.495	5.76	6700000	4000000	7000000	64.56
Q3U1F9	Phosphoprotein associated with glycosphingolipid-enriched microdomains 1 OS=Mus mus	6.7599068	1	1	1	46.52	4.81			900000	4.90
P16460	Argininosuccinate synthase OS=Mus musculus GN=Ass1 PE=1 SV=1	7.038835	2	4	2	46.555	8.22	9500000	4900000	4500000	13.75
Q7TNC4	Putative RNA-binding protein Luc7-like 2 OS=Mus musculus GN=Luc7l2 PE=1 SV=1	10.714286	3	5	2	46.555	10.1	3800000		2700000	16.48
P63034	Cytohesin-2 OS=Mus musculus GN=Cyth2 PE=1 SV=2	9	3	6	2	46.556	5.63	1100000	640000	2100000	17.48
O55028	[3-methyl-2-oxobutanoate dehydrogenase [lipoamide]] kinase, mitochondrial OS=Mus mus	3.1553398	1	1	1	46.558	8.91		220000		2.10
Q6P8X1	Sorting nexin-6 OS=Mus musculus GN=Snx6 PE=1 SV=2	2.7093596	1	3	1	46.62	6.16	1700000	860000	2000000	7.74
P63005	Platelet-activating factor acetylhydrolase IB subunit alpha OS=Mus musculus GN=Pafah1	27.560976	9	26	8	46.64	7.37	15000000	6100000	11000000	74.04
O88844	Isocitrate dehydrogenase [NADP] cytoplasmic OS=Mus musculus GN=Idh1 PE=1 SV=2	2.173913	1	3	1	46.644	7.17	2600000	1300000	2200000	6.40
Q920N7	Synaptotagmin-12 OS=Mus musculus GN=Syt12 PE=1 SV=1	8.0760095	3	4	3	46.651	5.64	3900000			9.98
Q9WV60	Glycogen synthase kinase-3 beta OS=Mus musculus GN=Gsk3b PE=1 SV=2	13.333333	3	5	2	46.681	8.78	6300000	3200000	2800000	14.55
P59017	Bcl-2-like protein 13 OS=Mus musculus GN=Bcl2l13 PE=1 SV=2	5.2995392	2	5	2	46.691	4.59	2700000	1100000	2800000	12.10
Q9D8U8	Sorting nexin-5 OS=Mus musculus GN=Snx5 PE=1 SV=1	5.4455446	2	3	2	46.768	6.62		710000	1600000	7.92
Q91VC3	Eukaryotic initiation factor 4A-III OS=Mus musculus GN=Eif4a3 PE=1 SV=3	9.9756691	4	7	4	46.81	6.73	4000000	2400000	2800000	18.56
Q9Z2I8	Succinate--CoA ligase [GDP-forming] subunit beta, mitochondrial OS=Mus musculus GN=	7.1593533	2	3	2	46.811	7.02	1300000	520000	1500000	9.39
Q9CZN4	Protein shisa-9 OS=Mus musculus GN=Shisa9 PE=1 SV=2	3.5377358	1	1	1	46.812	8.62	1500000			2.76
Q9D1Q6	Endoplasmic reticulum resident protein 44 OS=Mus musculus GN=Erp44 PE=1 SV=1	18.472906	6	14	5	46.823	5.27	4100000	1900000	2200000	37.27
Q9ER58	Testican-2 OS=Mus musculus GN=Spock2 PE=1 SV=1	6.1465721	2	4	2	46.833	4.87	1100000	1500000	2100000	13.33
Q99MR0	Actin-like protein 6B OS=Mus musculus GN=Actl6b PE=1 SV=1	2.3474178	1	1	1	46.861	5.71	300000			1.87
Q8R2R9	AP-3 complex subunit mu-2 OS=Mus musculus GN=Ap3m2 PE=1 SV=1	5.9808612	2	3	2	46.886	7.56	2200000	770000		8.26
Q3TCJ1	BRISC complex subunit Abro1 OS=Mus musculus GN=Fam175b PE=1 SV=1	3.1325301	1	2	1	46.914	6.18	840000		820000	3.93
Q8BWG8	Beta-arrestin-1 OS=Mus musculus GN=Arrb1 PE=1 SV=1	10.526316	3	5	3	46.943	6.28	1100000	300000	720000	13.73
Q9ESW4	Acylglycerol kinase, mitochondrial OS=Mus musculus GN=Agk PE=1 SV=1	16.389549	6	9	6	46.946	8.4	6000000	2300000	2900000	23.19
P30275	Creatine kinase U-type, mitochondrial OS=Mus musculus GN=Ckmt1 PE=1 SV=1	29.665072	10	33	10	46.974	8.16	36000000	20000000	22000000	102.61
Q9DCL9	Multifunctional protein ADE2 OS=Mus musculus GN=Paics PE=1 SV=4	4.2352941	1	3	1	46.976	7.23	2000000	1300000	1800000	9.08
Q99JX3	Golgi reassembly-stacking protein 2 OS=Mus musculus GN=Gorasp2 PE=1 SV=3	6.2084257	2	4	2	47.009	4.79	1800000	1600000	1300000	10.87
Q60634	Flotillin-2 OS=Mus musculus GN=Flot2 PE=1 SV=2	24.766355	10	18	10	47.009	5.2	5900000	4400000	5000000	53.79
Q62443	Neuronal pentraxin-1 OS=Mus musculus GN=Nptx1 PE=1 SV=1	10.648148	4	8	4	47.088	6.44	4300000	2900000	4600000	22.85
P16330	2',3'-cyclic-nucleotide 3'-phosphodiesterase OS=Mus musculus GN=Cnp PE=1 SV=3	38.333333	18	55	18	47.094	8.97	77000000	25000000	66000000	173.71
Q8R127	Saccharopine dehydrogenase-like oxidoreductase OS=Mus musculus GN=Sccpdh PE=1	2.0979021	1	2	1	47.099	8.6	3200000		2100000	4.06
P17182	Alpha-enolase OS=Mus musculus GN=Eno1 PE=1 SV=3	39.861751	14	38	12	47.111	6.8	13000000	12000000	14000000	113.37
Q9D8E6	60S ribosomal protein L4 OS=Mus musculus GN=Rpl4 PE=1 SV=3	23.627685	9	22	9	47.124	11	28000000	11000000	14000000	62.68
Q64345	Interferon-induced protein with tetratricopeptide repeats 3 OS=Mus musculus GN=Ifit3 PE	2.2332506	1	3	1	47.192	5.64	4800000	2400000	3700000	8.88
P46097	Synaptotagmin-2 OS=Mus musculus GN=Syt2 PE=1 SV=1	23.933649	10	34	4	47.232	7.99	4600000	1300000	2100000	96.60
P17183	Gamma-enolase OS=Mus musculus GN=Eno2 PE=1 SV=2	31.105991	9	26	7	47.267	5.11	6100000	6600000	5000000	86.68
Q80XI4	Phosphatidylinositol 5-phosphate 4-kinase type-2 beta OS=Mus musculus GN=Pip4k2b P	18.269231	6	21	3	47.289	7.33	10000000	4000000	7400000	62.73
Q9JJC6	RILP-like protein 1 OS=Mus musculus GN=Rilpl1 PE=1 SV=1	7.635468	1	1	1	47.294	5.16			1600000	4.93
Q8BP71	RNA binding protein fox-1 homolog 2 OS=Mus musculus GN=Rbfox2 PE=1 SV=2	3.3407572	1	3	1	47.301	6.55	1300000	1600000	2700000	6.89
Q91XU3	Phosphatidylinositol 5-phosphate 4-kinase type-2 gamma OS=Mus musculus GN=Pip4k2	4.0380048	1	3	1	47.306	6.89		1400000		8.58
Q6P8K8	Carboxypeptidase A4 OS=Mus musculus GN=Cpa4 PE=2 SV=2	1.9047619	1	1	1	47.309	6.61			530000	2.02
Q99JY9	Actin-related protein 3 OS=Mus musculus GN=Actr3 PE=1 SV=3	23.205742	8	31	7	47.327	5.88	19000000	11000000	19000000	97.22
Q9DBE8	Alpha-1,3/1,6-mannosyltransferase ALG2 OS=Mus musculus GN=Alg2 PE=1 SV=2	6.0240964	2	4	2	47.374	7.97	1100000	1100000		10.17
Q9CZP5	Mitochondrial chaperone BCS1 OS=Mus musculus GN=Bcs1l PE=1 SV=1	4.5454545	2	3	2	47.376	7.93		1000000	1000000	6.20
P54775	26S protease regulatory subunit 6B OS=Mus musculus GN=Psmc4 PE=1 SV=2	11.722488	5	7	5	47.379	5.21	1500000	320000	1400000	21.74
P05202	Aspartate aminotransferase, mitochondrial OS=Mus musculus GN=Got2 PE=1 SV=1	26.511628	11	55	11	47.381	9	80000000	30000000	58000000	156.57

Q922R1	UPF0183 protein C16orf70 homolog OS=Mus musculus PE=1 SV=2		2.3696682	1	3	1	47.386	7.74	1100000	540000	1100000	8.49
P46096	Synaptotagmin-1 OS=Mus musculus GN=Syt1 PE=1 SV=1		41.330166	19	107	12	47.388	8.53	170000000	52000000	110000000	322.71
Q8BG32	26S proteasome non-ATPase regulatory subunit 11 OS=Mus musculus GN=Psmd11 PE=		6.1611374	2	4	2	47.407	6.48	3600000	940000	2600000	13.27
Q7TQ95	Protein lunapark OS=Mus musculus GN=Lnp PE=1 SV=1		10.352941	4	11	4	47.47	5.27	2400000	680000	1900000	31.60
O08917	Flotillin-1 OS=Mus musculus GN=Flot1 PE=1 SV=1		26.168224	9	19	9	47.484	7.15	5100000	2900000	4500000	58.01
Q8BLQ9	Cell adhesion molecule 2 OS=Mus musculus GN=Cadm2 PE=1 SV=2		21.609195	8	31	8	47.529	5.41	40000000	41000000	58000000	108.89
Q641P0	Actin-related protein 3B OS=Mus musculus GN=Actr3b PE=1 SV=1		19.37799	6	18	5	47.549	6.02	3400000	1900000	3300000	49.10
Q60972	Histone-binding protein RBBP4 OS=Mus musculus GN=Rbbp4 PE=1 SV=5		21.882353	8	16	4	47.626	4.89	4500000	3300000	5000000	43.31
Q91VZ6	Stromal membrane-associated protein 1 OS=Mus musculus GN=Smap1 PE=1 SV=1		2.5	1	3	1	47.63	8.51	3800000	1700000	5500000	8.47
O88712	C-terminal-binding protein 1 OS=Mus musculus GN=Ctbp1 PE=1 SV=2		21.995465	8	22	8	47.715	6.77	7400000	5000000	11000000	72.25
P32067	Lupus La protein homolog OS=Mus musculus GN=Ssb PE=1 SV=1		16.385542	6	11	6	47.727	9.77	7400000	1400000	3100000	30.81
Q8BK72	28S ribosomal protein S27, mitochondrial OS=Mus musculus GN=Mrps27 PE=1 SV=2		5.5421687	2	2	2	47.748	5.5			1500000	5.74
Q60973	Histone-binding protein RBBP7 OS=Mus musculus GN=Rbbp7 PE=1 SV=1		10.823529	5	8	1	47.76	5.05			1700000	19.32
O88543	COP9 signalosome complex subunit 3 OS=Mus musculus GN=Cops3 PE=1 SV=3		7.5650118	2	5	2	47.801	6.65	1500000	730000	780000	16.08
P51174	Long-chain specific acyl-CoA dehydrogenase, mitochondrial OS=Mus musculus GN=Acad		16.511628	5	14	5	47.877	8.31	6300000	2600000	5500000	48.51
Q922H2	[Pyruvate dehydrogenase (acetyl-transferring)] kinase isozyme 3, mitochondrial OS=Mus		5.5421687	2	5	2	47.893	8.82	1800000	1000000	710000	12.93
Q99KH8	Serine/threonine-protein kinase 24 OS=Mus musculus GN=Stk24 PE=1 SV=1		12.064965	4	5	4	47.924	5.43	2400000	980000	4100000	13.98
P14211	Calreticulin OS=Mus musculus GN=Calr PE=1 SV=1		52.644231	18	65	18	47.965	4.49	23000000	14000000	27000000	213.86
Q9CQC6	Basic leucine zipper and W2 domain-containing protein 1 OS=Mus musculus GN=Bzw1 P		1.6706444	1	1	1	48.013	5.92	1900000			1.84
Q99KK2	N-acylneuraminate cytidylyltransferase OS=Mus musculus GN=Cmas PE=1 SV=2		16.898148	6	9	6	48.028	8.1	8400000		3600000	23.31
Q922R8	Protein disulfide-isomerase A6 OS=Mus musculus GN=Pdia6 PE=1 SV=3		19.318182	6	13	6	48.07	5.14	7400000	4800000	5700000	39.05
Q64337	Sequestosome-1 OS=Mus musculus GN=Sqstm1 PE=1 SV=1		20.135747	4	7	4	48.132	5.21	2100000	1100000	2100000	20.21
Q9QWL7	SWISS-PROT:Q9QWL7 Tax_Id=10090 Gene_Symbol=Krt17 Keratin, type I	x	30.254042	15	47	3	48.132	5.06	1200000		4300000	126.95
Q60780	Growth arrest-specific protein 7 OS=Mus musculus GN=Gas7 PE=1 SV=1		23.515439	8	17	8	48.143	7.83	4600000	2700000	5500000	46.36
O09161	Calsequestrin-2 OS=Mus musculus GN=Casq2 PE=1 SV=3		3.1325301	1	1	1	48.146	4.27			1400000	2.79
Q99N87	28S ribosomal protein S5, mitochondrial OS=Mus musculus GN=Mrps5 PE=1 SV=1		2.7777778	1	1	1	48.176	10.14	1400000			1.82
Q9DB77	Cytochrome b-c1 complex subunit 2, mitochondrial OS=Mus musculus GN=Uqcrc2 PE=1		38.852097	14	41	14	48.205	9.25	46000000	34000000	38000000	142.76
Q3TIV5	Zinc finger CCCH domain-containing protein 15 OS=Mus musculus GN=Zc3h15 PE=1 SV		6.1032864	2	3	2	48.297	5.3	1400000		1100000	8.10
Q921S7	39S ribosomal protein L37, mitochondrial OS=Mus musculus GN=Mrpl37 PE=1 SV=1		4.2553191	1	2	1	48.31	8.84		890000	910000	5.10
P29758	Ornithine aminotransferase, mitochondrial OS=Mus musculus GN=Oat PE=1 SV=1		3.1890661	1	2	1	48.324	6.62	2300000		2300000	5.52
Q60749	KH domain-containing, RNA-binding, signal transduction-associated protein 1 OS=Mus m		5.1918736	3	8	2	48.34	8.72	7800000	1400000	4700000	27.56
P35585	AP-1 complex subunit mu-1 OS=Mus musculus GN=Ap1m1 PE=1 SV=3		18.91253	6	11	6	48.512	7.3	2800000	1200000	2500000	27.47
Q99JB8	Protein kinase C and casein kinase II substrate protein 3 OS=Mus musculus GN=Pacsin3		8.0188679	2	5	2	48.555	6.1	730000	310000	810000	15.19
Q60759	Glutaryl-CoA dehydrogenase, mitochondrial OS=Mus musculus GN=Gcdh PE=1 SV=2		3.196347	1	2	1	48.575	8.73	1900000		2000000	5.28
P46471	26S protease regulatory subunit 7 OS=Mus musculus GN=Psmc2 PE=1 SV=5		15.011547	5	11	5	48.617	5.95	4600000	1900000	4000000	35.46
Q62418	Drebrin-like protein OS=Mus musculus GN=Dbnl PE=1 SV=2		35.550459	11	41	11	48.67	4.92	7000000	5400000	9300000	134.25
Q3TPE9	Ankyrin repeat and MYND domain-containing protein 2 OS=Mus musculus GN=Ankmy2 P		2.5	1	1	1	48.742	6.67	870000			2.76
Q8C4X2	Casein kinase I isoform gamma-3 OS=Mus musculus GN=Csnk1g3 PE=1 SV=2		1.8867925	1	1	1	48.907	9.11				1.87
P59325	Eukaryotic translation initiation factor 5 OS=Mus musculus GN=Eif5 PE=1 SV=1		6.7599068	2	3	2	48.938	5.52	3000000		2300000	8.03
Q5DTY9	BTB/POZ domain-containing protein KCTD16 OS=Mus musculus GN=Kctd16 PE=1 SV=2		9.8360656	3	5	3	48.941	8.19	9000000		5400000	15.12
Q9D2G2	Dihydrolipoyllysine-residue succinyltransferase component of 2-oxoglutarate dehydrogena		22.687225	8	21	8	48.963	8.95	9200000	9800000	11000000	61.84
Q8BFP9	[Pyruvate dehydrogenase (acetyl-transferring)] kinase isozyme 1, mitochondrial OS=Mus		2.0737327	1	1	1	48.964	8.19	3100000			2.00
O54734	Dolichyl-diphosphooligosaccharide--protein glycosyltransferase 48 kDa subunit OS=Mus m		8.1632653	3	7	3	48.997	5.83	4300000	4200000	6000000	17.73
Q9D3A9	Protein tweety homolog 1 OS=Mus musculus GN=Ttyh1 PE=1 SV=1		2.8888889	1	1	1	49.001	5.03			1200000	2.34
Q9Z1N5	Spliceosome RNA helicase Ddx39b OS=Mus musculus GN=Ddx39b PE=1 SV=1		12.850467	5	16	5	49.004	5.67	9300000	1400000	3700000	43.08
Q8CI61	BAG family molecular chaperone regulator 4 OS=Mus musculus GN=Bag4 PE=1 SV=2		3.7199125	1	1	1	49.064	5.34			1100000	3.94
P62192	26S protease regulatory subunit 4 OS=Mus musculus GN=Psmc1 PE=1 SV=1		9.0909091	3	8	3	49.154	6.21	2300000	1200000	2000000	31.70
O35737	Heterogeneous nuclear ribonucleoprotein H OS=Mus musculus GN=Hnrnph1 PE=1 SV=3		22.717149	7	24	2	49.168	6.3	7900000	2100000	3300000	83.02

P70333	Heterogeneous nuclear ribonucleoprotein H2 OS=Mus musculus GN=Hnrnph2 PE=1 SV=		21.603563	6	23	2	49.248	6.3	8000000	5200000	7200000	83.22
Q63912	Oligodendrocyte-myelin glycoprotein OS=Mus musculus GN=Omg PE=1 SV=1		8.8636364	4	11	4	49.252	8.41	11000000	6300000	7400000	27.46
Q5NCE8	Magnesium transporter MRS2 homolog, mitochondrial OS=Mus musculus GN=Mrs2 PE=2		2.3041475	1	1	1	49.268	7.53	1300000			3.02
Q9JIY5	Serine protease HTRA2, mitochondrial OS=Mus musculus GN=Htra2 PE=1 SV=2		2.6200873	1	1	1	49.318	9.6	1900000			2.50
A2BDX3	Adenylyltransferase and sulfurtransferase MOCS3 OS=Mus musculus GN=Mocs3 PE=1 S		1.9565217	1	1	1	49.344	7.49		300000		2.11
P62484	Abl interactor 2 OS=Mus musculus GN=Abi2 PE=1 SV=1		7.8475336	3	9	2	49.356	6.01	7400000	4700000	6100000	28.32
Q6P2L7	Protein CASC4 OS=Mus musculus GN=Casc4 PE=1 SV=1		2.7586207	1	1	1	49.379	5.45	490000			0.00
P46467	Vacuolar protein sorting-associated protein 4B OS=Mus musculus GN=Vps4b PE=1 SV=2		1.8018018	1	1	1	49.389	7.11	450000			1.94
Q8BFR5	Elongation factor Tu, mitochondrial OS=Mus musculus GN=Tufm PE=1 SV=1		20.132743	8	21	8	49.477	7.56	9900000	4100000	7900000	61.93
Q8CE50	Sorting nexin-30 OS=Mus musculus GN=Snx30 PE=1 SV=1		1.8306636	1	1	1	49.489	5.35	860000			2.39
O88685	26S protease regulatory subunit 6A OS=Mus musculus GN=Psmc3 PE=1 SV=2		17.420814	5	12	5	49.518	5.19	4100000	1900000	1700000	36.94
Q9D6F9	Tubulin beta-4A chain OS=Mus musculus GN=Tubb4a PE=1 SV=3		73.423423	25	276	6	49.554	4.88	49000000	43000000	60000000	834.01
Q8CGA0	Protein phosphatase 1F OS=Mus musculus GN=Ppm1f PE=1 SV=1		5.3097345	2	2	2	49.58	5.3				4.85
Q9R1T4	Septin-6 OS=Mus musculus GN=Sept6 PE=1 SV=4		38.940092	13	46	6	49.588	6.43	16000000	12000000	24000000	146.46
P84091	AP-2 complex subunit mu OS=Mus musculus GN=Ap2m1 PE=1 SV=1		28.735632	12	31	12	49.623	9.54	30000000	12000000	17000000	87.37
P99024	Tubulin beta-5 chain OS=Mus musculus GN=Tubb5 PE=1 SV=1		73.423423	26	325	4	49.639	4.89	100000000	47000000	66000000	1000.62
Q8C1B7	Septin-11 OS=Mus musculus GN=Sept11 PE=1 SV=4		39.211137	16	51	7	49.663	6.68	31000000	22000000	37000000	144.62
P36552	Oxygen-dependent coproporphyrinogen-III oxidase, mitochondrial OS=Mus musculus GN=		3.3860045	1	1	1	49.683	8.53				2.72
Q8R5M8	Cell adhesion molecule 1 OS=Mus musculus GN=Cadm1 PE=1 SV=2		19.95614	7	30	7	49.757	5.03	37000000	39000000	44000000	98.62
Q99PL6	UBX domain-containing protein 6 OS=Mus musculus GN=Ubxn6 PE=1 SV=1		7.239819	2	3	2	49.765	8.54	720000	1100000		7.54
Q8CHH9	Septin-8 OS=Mus musculus GN=Sept8 PE=1 SV=4		33.566434	12	32	8	49.781	6	16000000	10000000	17000000	84.30
Q7Z3Y8	SWISS-PROT:Q7Z3Y8 Tax_Id=9606 Gene_Symbol=KRT27 Keratin, type I	x	12.636166	6	34	4	49.793	5.14			6200000	81.45
P68372	Tubulin beta-4B chain OS=Mus musculus GN=Tubb4b PE=1 SV=1		73.258427	27	345	2	49.799	4.89	130000000	59000000	87000000	1055.84
P03995	Glial fibrillary acidic protein OS=Mus musculus GN=Gfap PE=1 SV=4		26.27907	11	21	10	49.87	5.34	3100000	2500000	5000000	54.37
Q7TMM9	Tubulin beta-2A chain OS=Mus musculus GN=Tubb2a PE=1 SV=1		75.280899	28	346	1	49.875	4.89	430000000	190000000	260000000	1066.30
P68368	Tubulin alpha-4A chain OS=Mus musculus GN=Tuba4a PE=1 SV=1		58.928571	26	145	8	49.892	5.06	260000000	130000000	280000000	445.69
Q07076	Annexin A7 OS=Mus musculus GN=Anxa7 PE=1 SV=2		8.8552916	3	9	3	49.893	6.18	1600000	2400000	2000000	28.12
Q9D0G0	28S ribosomal protein S30, mitochondrial OS=Mus musculus GN=Mrps30 PE=1 SV=1		2.7149321	1	1	1	49.908	9.38	560000			2.96
Q9CWF2	Tubulin beta-2B chain OS=Mus musculus GN=Tubb2b PE=1 SV=1		75.280899	28	342	1	49.921	4.89	17000000	10000000	12000000	1055.47
P01872	Ig mu chain C region OS=Mus musculus GN=Ighm PE=1 SV=2		3.5242291	1	3	1	49.94	7.01	1800000	1000000	1500000	11.87
Q3UX10	Tubulin alpha chain-like 3 OS=Mus musculus GN=Tubal3 PE=2 SV=2		9.6412556	5	33	1	49.956	5.58	16000000	5400000	7200000	85.20
Q9JJZ2	Tubulin alpha-8 chain OS=Mus musculus GN=Tuba8 PE=1 SV=1		35.18931	14	86	3	50.02	5.1	2800000	1200000	2100000	261.70
Q9D8N0	Elongation factor 1-gamma OS=Mus musculus GN=Eef1g PE=1 SV=3		22.883295	10	38	10	50.029	6.74	32000000	9500000	18000000	115.57
P10126	Elongation factor 1-alpha 1 OS=Mus musculus GN=Eef1a1 PE=1 SV=3		16.450216	8	29	3	50.082	9.01	13000000	6400000	15000000	71.91
Q9Z2I9	Succinate--CoA ligase [ADP-forming] subunit beta, mitochondrial OS=Mus musculus GN=		28.941685	14	50	14	50.082	7.01	19000000	10000000	18000000	149.14
P68369	Tubulin alpha-1A chain OS=Mus musculus GN=Tuba1a PE=1 SV=1		59.645233	27	175	8	50.104	5.06	1.1E+09	500000000	1.2E+09	517.60
P60122	RuvB-like 1 OS=Mus musculus GN=Ruvbl1 PE=1 SV=1		9.4298246	3	6	3	50.182	6.42	1400000	1000000	1500000	17.10
P50136	2-oxoisovalerate dehydrogenase subunit alpha, mitochondrial OS=Mus musculus GN=Bcl		3.3936652	1	1	1	50.339	8.06		6500000		2.55
Q5FWK3	Rho GTPase-activating protein 1 OS=Mus musculus GN=Arhgap1 PE=1 SV=1		8.8838269	3	5	3	50.379	6.44	3300000		2200000	15.78
Q9ERD7	Tubulin beta-3 chain OS=Mus musculus GN=Tubb3 PE=1 SV=1		52.222222	22	219	7	50.386	4.93	78000000	42000000	73000000	709.11
Q8BVY0	Ribosomal L1 domain-containing protein 1 OS=Mus musculus GN=Rsl1d1 PE=1 SV=1		3.0973451	1	1	1	50.39	9.98			660000	2.37
P62631	Elongation factor 1-alpha 2 OS=Mus musculus GN=Eef1a2 PE=1 SV=1		16.414687	8	32	3	50.422	9.03	22000000	7400000	13000000	84.34
Q9QZI8	Serine incorporator 1 OS=Mus musculus GN=Serinc1 PE=1 SV=1		2.8697572	1	3	1	50.475	6.28	4800000	250000		7.66
P50396	Rab GDP dissociation inhibitor alpha OS=Mus musculus GN=Gdi1 PE=1 SV=3		46.756152	18	58	13	50.489	5.08	34000000	18000000	30000000	185.48
Q6KB66-1	SWISS-PROT:Q6KB66-1 Tax_Id=9606 Gene_Symbol=KRT80 Isoform 1 of	x	9.5132743	4	5	3	50.494	5.67		2000000	4100000	12.52
Q61598	Rab GDP dissociation inhibitor beta OS=Mus musculus GN=Gdi2 PE=1 SV=1		27.191011	11	32	6	50.505	6.25	6400000	3000000	5700000	95.27
Q8K2F8	Protein LSM14 homolog A OS=Mus musculus GN=Lsm14a PE=1 SV=1		4.3290043	2	2	2	50.515	9.52	1100000			3.87
O55131	Septin-7 OS=Mus musculus GN=Sept7 PE=1 SV=1		34.633028	14	71	13	50.518	8.57	83000000	47000000	66000000	181.79

Q9R0X4	Acyl-coenzyme A thioesterase 9, mitochondrial OS=Mus musculus GN=Acot9 PE=1 SV=1		4.7835991	2	3	2	50.528	8.59		740000	900000	8.17
Q9Z1J3	Cysteine desulfurase, mitochondrial OS=Mus musculus GN=Nfs1 PE=1 SV=3		5.2287582	2	4	2	50.538	8.16	130000	1100000	2600000	9.73
Q61644	Protein kinase C and casein kinase substrate in neurons protein 1 OS=Mus musculus GN		55.328798	21	91	21	50.544	5.24	91000000	68000000	120000000	274.17
Q924S8	Sprouty-related, EVH1 domain-containing protein 1 OS=Mus musculus GN=Spred1 PE=1		4.2792793	1	2	1	50.631	6.47	870000		890000	7.92
Q9JJK2	LanC-like protein 2 OS=Mus musculus GN=Lancl2 PE=1 SV=1		16.888889	6	19	6	50.745	7.28	5100000	1900000	3900000	57.92
Q91YT0	NADH dehydrogenase [ubiquinone] flavoprotein 1, mitochondrial OS=Mus musculus GN=		25.646552	10	28	10	50.802	8.21	23000000	11000000	18000000	79.45
Q8C5H8	NAD kinase 2, mitochondrial OS=Mus musculus GN=Nadk2 PE=1 SV=2		5.9734513	2	3	2	50.827	8.07	540000	430000		9.16
Q8C7D2	Protein cereblon OS=Mus musculus GN=Crbn PE=1 SV=1		3.1460674	1	1	1	50.847	5.33	1600000			2.27
Q91YP0	L-2-hydroxyglutarate dehydrogenase, mitochondrial OS=Mus musculus GN=L2hgdh PE=1		4.9568966	2	4	2	50.866	8.29	1100000	1000000	1900000	12.76
P54071	Isocitrate dehydrogenase [NADP], mitochondrial OS=Mus musculus GN=Idh2 PE=1 SV=3		17.699115	7	12	7	50.874	8.69	4600000	1600000	2400000	33.81
Q8R071	Inositol-trisphosphate 3-kinase A OS=Mus musculus GN=Itpka PE=1 SV=1		13.28976	5	13	5	50.903	7.72	6400000	3200000	4800000	39.47
Q8CI32	BAG family molecular chaperone regulator 5 OS=Mus musculus GN=Bag5 PE=1 SV=1		10.290828	3	4	3	50.911	6.05	990000		1700000	13.31
Q64378	Peptidyl-prolyl cis-trans isomerase FKBP5 OS=Mus musculus GN=Fkbp5 PE=1 SV=1		3.0701754	1	2	1	50.934	7.8	510000	220000		4.91
P61979	Heterogeneous nuclear ribonucleoprotein K OS=Mus musculus GN=Hnrnpk PE=1 SV=1		29.157667	13	46	13	50.944	5.54	60000000	23000000	40000000	138.42
O89053	Coronin-1A OS=Mus musculus GN=Coro1a PE=1 SV=5		34.056399	14	37	14	50.957	6.48	32000000	16000000	37000000	91.89
Q9R111	Guanine deaminase OS=Mus musculus GN=Gda PE=1 SV=1		18.281938	7	16	7	50.981	5.53	13000000	7300000	13000000	43.15
Q9Z0N1	Eukaryotic translation initiation factor 2 subunit 3, X-linked OS=Mus musculus GN=Eif2s3x		2.1186441	1	2	1	51.033	8.4		910000	1500000	4.81
O35857	Mitochondrial import inner membrane translocase subunit TIM44 OS=Mus musculus GN=		5.3097345	2	5	2	51.059	8.13	3000000	880000	1700000	15.35
P83887	Tubulin gamma-1 chain OS=Mus musculus GN=Tubg1 PE=1 SV=1		1.9955654	1	1	1	51.069	6.02	1700000			2.64
Q9WTM5	RuvB-like 2 OS=Mus musculus GN=Ruvbl2 PE=1 SV=3		12.311015	5	8	5	51.081	5.64	2200000	1800000	2000000	20.87
P21956	Lactadherin OS=Mus musculus GN=Mfge8 PE=1 SV=3		4.7516199	2	2	2	51.208	6.52	2500000		250000	4.89
P08779	SWISS-PROT:P08779 Tax_Id=9606 Gene_Symbol=KRT16 Keratin, type I c	x	43.340381	21	72	6	51.236	5.05	3400000	1300000	2900000	192.21
Q99L43	Phosphatidate cytidylyltransferase 2 OS=Mus musculus GN=Cds2 PE=1 SV=1		8.7837838	4	10	4	51.28	7.05	14000000	8400000	6600000	38.98
P12657	Muscarinic acetylcholine receptor M1 OS=Mus musculus GN=Chrm1 PE=1 SV=2		6.0869565	2	4	2	51.345	9.32	1600000	560000	1700000	11.13
Q99JY0	Trifunctional enzyme subunit beta, mitochondrial OS=Mus musculus GN=Hadhb PE=1 SV		8.6315789	4	7	4	51.353	9.38	3800000	1600000	2600000	16.27
Q8R1S0	Ubiquinone biosynthesis monooxygenase COQ6, mitochondrial OS=Mus musculus GN=C		1.8907563	1	1	1	51.36	7.17	420000			2.36
Q5SUF2	Luc7-like protein 3 OS=Mus musculus GN=Luc7l3 PE=1 SV=1		6.712963	2	2	2	51.419	9.77	1200000		950000	7.03
P40124	Adenylyl cyclase-associated protein 1 OS=Mus musculus GN=Cap1 PE=1 SV=4		37.974684	12	27	11	51.532	7.52	8600000	5600000	7800000	77.90
P30416	Peptidyl-prolyl cis-trans isomerase FKBP4 OS=Mus musculus GN=Fkbp4 PE=1 SV=5		10.262009	5	13	5	51.54	5.72	2900000	1800000	3000000	33.06
P61202	COP9 signalosome complex subunit 2 OS=Mus musculus GN=Cops2 PE=1 SV=1		10.835214	4	8	4	51.564	5.53	2000000	940000	2600000	22.13
P02533	SWISS-PROT:P02533 Tax_Id=9606 Gene_Symbol=KRT14 Keratin, type I c	x	55.29661	27	111	5	51.589	5.16	20000000	7100000	51000000	317.71
Q06890	Clusterin OS=Mus musculus GN=Clu PE=1 SV=1		18.75	7	19	7	51.623	5.67	4200000	4300000	5300000	55.97
Q2NL51	Glycogen synthase kinase-3 alpha OS=Mus musculus GN=Gsk3a PE=1 SV=2		4.8979592	2	2	1	51.628	8.81	1100000			5.46
Q6P1F6	Serine/threonine-protein phosphatase 2A 55 kDa regulatory subunit B alpha isoform OS=l		13.199105	5	8	5	51.659	6.2	6300000	3100000	3700000	21.88
O08842	GDNF family receptor alpha-2 OS=Mus musculus GN=Gfra2 PE=1 SV=2		4.9568966	2	4	2	51.693	7.75	4900000	4400000	5300000	8.12
Q9CZU6	Citrate synthase, mitochondrial OS=Mus musculus GN=Cs PE=1 SV=1		22.198276	11	32	11	51.703	8.57	47000000	29000000	44000000	78.60
P62812	Gamma-aminobutyric acid receptor subunit alpha-1 OS=Mus musculus GN=Gabra1 PE=1		14.945055	6	16	6	51.721	9.31	8000000	3800000	7100000	40.08
Q91YJ2	Sorting nexin-4 OS=Mus musculus GN=Snx4 PE=1 SV=1		12.666667	5	9	5	51.745	5.8	1600000	1200000	1900000	26.83
P26339	Chromogranin-A OS=Mus musculus GN=Chga PE=1 SV=1		2.3758099	1	3	1	51.758	4.72	520000	540000	840000	9.91
P97855	Ras GTPase-activating protein-binding protein 1 OS=Mus musculus GN=G3bp1 PE=1 SV		6.0215054	2	2	2	51.797	5.59		360000	610000	5.59
Q8CI78	Required for meiotic nuclear division protein 1 homolog OS=Mus musculus GN=Rmnd1 P		5.5555556	2	4	2	51.81	7.99	1400000	1300000	1900000	13.64
Q8BXZ1	Protein disulfide-isomerase TMX3 OS=Mus musculus GN=Tmx3 PE=1 SV=2		8.7719298	3	8	3	51.815	5.16	4200000	2500000	4200000	28.34
Q8BGH2	Sorting and assembly machinery component 50 homolog OS=Mus musculus GN=Samm5		18.123667	8	14	8	51.831	6.8	4600000	2900000	2600000	28.25
A2RT62	F-box/LRR-repeat protein 16 OS=Mus musculus GN=Fbxl16 PE=1 SV=1		5.2192067	2	2	2	51.845	6.6	2400000		2500000	6.32
Q7TNR6	Immunoglobulin superfamily member 21 OS=Mus musculus GN=Igsf21 PE=2 SV=1		4.7008547	2	2	2	51.905	6.95		2000000		4.94
Q9CQU1	Microfibrillar-associated protein 1 OS=Mus musculus GN=Mfap1 PE=1 SV=1		4.1002278	1	2	1	51.923	4.98	1400000		1500000	5.60
Q62376	U1 small nuclear ribonucleoprotein 70 kDa OS=Mus musculus GN=Snrnp70 PE=1 SV=2		13.392857	6	10	6	51.961	9.94	8600000		5400000	29.60
Q8BTV2	Cleavage and polyadenylation specificity factor subunit 7 OS=Mus musculus GN=Cpsf7 P		4.0339703	1	2	1	51.979	8	2600000		2600000	7.17

Q5Y4Y6	Gasdermin-A3 OS=Mus musculus GN=Gsdma3 PE=1 SV=1	2.8017241	1	1	1	51.987	5.71			1000000	2.37
Q3UJU9	Regulator of microtubule dynamics protein 3 OS=Mus musculus GN=Rmdn3 PE=1 SV=2	15.744681	5	16	5	51.997	5.21	4400000	1200000	2900000	47.13
Q3UYG8	O-acetyl-ADP-ribose deacetylase MACROD2 OS=Mus musculus GN=Macrod2 PE=1 SV=	3.1578947	1	3	1	52.112	4.75	1200000	450000	1200000	11.09
Q9Z2W0	Aspartyl aminopeptidase OS=Mus musculus GN=Dnpep PE=1 SV=2	2.5369979	1	1	1	52.174	7.25			1300000	2.66
P60229	Eukaryotic translation initiation factor 3 subunit E OS=Mus musculus GN=Eif3e PE=1 SV=	4.7191011	2	5	2	52.187	6.04	2100000	2700000	3900000	12.73
P70290	55 kDa erythrocyte membrane protein OS=Mus musculus GN=Mpp1 PE=1 SV=1	2.360515	1	1	1	52.194	7.2			700000	2.37
Q99J85	Neuronal pentraxin receptor OS=Mus musculus GN=Nptxr PE=1 SV=1	7.7079108	3	7	3	52.252	6.13	2300000	1600000	2700000	16.68
Q8CBW3	Abl interactor 1 OS=Mus musculus GN=Abi1 PE=1 SV=3	9.3555094	4	9	3	52.255	7.64	3900000	2000000	3600000	28.36
Q9Z2D6	Methyl-CpG-binding protein 2 OS=Mus musculus GN=Mecp2 PE=1 SV=1	24.380165	8	21	8	52.275	9.96	9600000	450000	6100000	72.37
Q99M87	DnaJ homolog subfamily A member 3, mitochondrial OS=Mus musculus GN=Dnaja3 PE=	11.666667	4	7	4	52.41	9.22	4600000	1200000	2200000	17.48
Q6GQS1	Calcium-binding mitochondrial carrier protein SCaMC-3 OS=Mus musculus GN=Slc25a23	5.9957173	3	8	2	52.464	7.59	3600000	1900000	1400000	17.71
Q8R016	Bleomycin hydrolase OS=Mus musculus GN=Blmh PE=1 SV=1	2.8571429	1	1	1	52.477	6.48			5000000	3.11
Q9CT10	Ran-binding protein 3 OS=Mus musculus GN=Ranbp3 PE=1 SV=2	6.3136456	2	5	2	52.541	5.12	500000		620000	19.89
Q9DB72	BTB/POZ domain-containing protein 17 OS=Mus musculus GN=Btbd17 PE=1 SV=1	3.9748954	1	2	1	52.569	9.42		860000	850000	7.51
A2ASZ8	Calcium-binding mitochondrial carrier protein SCaMC-2 OS=Mus musculus GN=Slc25a25	5.54371	3	6	2	52.587	8.54	2300000	1000000	1800000	11.93
Q91WD5	NADH dehydrogenase [ubiquinone] iron-sulfur protein 2, mitochondrial OS=Mus musculus	16.846652	7	21	7	52.592	6.99	14000000	8800000	9900000	58.48
P08414	Calcium/calmodulin-dependent protein kinase type IV OS=Mus musculus GN=Camk4 PE=	3.4115139	1	5	1	52.594	4.93	520000	200000	680000	16.87
Q99JR5	Tubulointerstitial nephritis antigen-like OS=Mus musculus GN=Tinagl1 PE=1 SV=1	6.6523605	2	2	2	52.63	6.77	6600000		1400000	4.55
P56959	RNA-binding protein FUS OS=Mus musculus GN=Fus PE=1 SV=1	4.4401544	2	4	2	52.642	9.36	22000000	4600000	13000000	11.88
Q99JG2	Prosaposin receptor GPR37L1 OS=Mus musculus GN=Gpr37l1 PE=1 SV=2	1.6632017	1	1	1	52.694	7.36	5900000			2.06
Q9D1A2	Cytosolic non-specific dipeptidase OS=Mus musculus GN=Cndp2 PE=1 SV=1	2.5263158	1	2	1	52.734	5.66		550000	710000	4.39
P47746	Cannabinoid receptor 1 OS=Mus musculus GN=Cnr1 PE=1 SV=1	1.9027484	1	1	1	52.796	8.27	1300000			1.96
Q9CZ13	Cytochrome b-c1 complex subunit 1, mitochondrial OS=Mus musculus GN=Uqcrc1 PE=1	49.166667	21	64	21	52.819	6.21	75000000	50000000	88000000	206.48
Q9CYT6	Adenylyl cyclase-associated protein 2 OS=Mus musculus GN=Cap2 PE=1 SV=1	27.10084	11	33	10	52.829	6.43	7900000	5100000	9800000	100.68
Q9D8W5	26S proteasome non-ATPase regulatory subunit 12 OS=Mus musculus GN=Psmd12 PE=	12.280702	4	10	4	52.861	7.06	5000000	2500000	4000000	31.70
O08663	Methionine aminopeptidase 2 OS=Mus musculus GN=Metap2 PE=1 SV=1	6.9037657	3	3	3	52.888	5.82	1700000		2200000	9.68
D3YZP9	Coiled-coil domain-containing protein 6 OS=Mus musculus GN=Ccdc6 PE=1 SV=1	2.3454158	1	1	1	52.906	7.34			1900000	2.76
O35495	Cyclin-dependent kinase 14 OS=Mus musculus GN=Cdk14 PE=1 SV=2	2.771855	1	1	1	52.962	8.92	1300000			2.59
Q9D394	Protein RUFY3 OS=Mus musculus GN=Rufy3 PE=1 SV=1	17.697228	6	10	6	52.973	5.49	4000000	1900000	2200000	29.50
Q8CBY8	Dynactin subunit 4 OS=Mus musculus GN=Dctn4 PE=1 SV=1	2.1413276	1	1	1	53.023	7.72	730000			1.90
Q8C5Q4	G-rich sequence factor 1 OS=Mus musculus GN=Grsf1 PE=1 SV=2	2.5052192	1	1	1	53.043	6.67			790000	2.20
Q9WUM4	Coronin-1C OS=Mus musculus GN=Coro1c PE=1 SV=2	13.50211	6	16	6	53.087	7.08	7500000	2600000	6800000	46.52
Q9JI44	DNA methyltransferase 1-associated protein 1 OS=Mus musculus GN=Dmap1 PE=1 SV=	4.0598291	1	2	1	53.097	9.5	1100000		280000	5.60
Q9DCD0	6-phosphogluconate dehydrogenase, decarboxylating OS=Mus musculus GN=Pgd PE=1	3.5196687	1	2	1	53.213	7.23		580000	1900000	7.06
P53395	Lipoamide acyltransferase component of branched-chain alpha-keto acid dehydrogenase	3.7344398	2	4	2	53.213	8.6	2300000	10000000	1900000	10.80
Q63850	Nuclear pore glycoprotein p62 OS=Mus musculus GN=Nup62 PE=1 SV=2	2.661597	1	2	1	53.222	5.31	780000		620000	5.71
Q00493	Carboxypeptidase E OS=Mus musculus GN=Cpe PE=1 SV=2	9.0336134	4	6	4	53.222	5.19	2100000	3000000	1600000	16.70
P53986	Monocarboxylate transporter 1 OS=Mus musculus GN=Slc16a1 PE=1 SV=1	6.693712	2	4	2	53.232	7.47	5700000	1600000	4400000	12.93
Q9D0L8	mRNA cap guanine-N7 methyltransferase OS=Mus musculus GN=Rnmt PE=1 SV=1	4.0860215	2	4	2	53.258	6.48	1600000	280000	1400000	8.57
Q920M7	Synaptotagmin-17 OS=Mus musculus GN=Syt17 PE=1 SV=1	6.8085106	2	3	2	53.26	7.15	890000	270000		8.55
Q9R0M4	Podocalyxin OS=Mus musculus GN=Podxl PE=1 SV=2	2.9821074	1	2	1	53.356	4.97	950000		800000	5.15
Q02819	Nucleobindin-1 OS=Mus musculus GN=Nucb1 PE=1 SV=2	10.239651	4	7	4	53.376	5.07	1600000	1200000	2400000	17.48
Q99LD4	COP9 signalosome complex subunit 1 OS=Mus musculus GN=Gps1 PE=1 SV=1	9.7664544	4	7	4	53.408	6.84	2600000	990000	2700000	19.80
P32037	Solute carrier family 2, facilitated glucose transporter member 3 OS=Mus musculus GN=S	10.141988	5	18	5	53.444	4.98	13000000	2700000	7000000	61.81
Q9JLJ2	4-trimethylaminobutyraldehyde dehydrogenase OS=Mus musculus GN=Aldh9a1 PE=1 S\	3.8461538	1	1	1	53.48	6.98	1400000			3.10
P26369	Splicing factor U2AF 65 kDa subunit OS=Mus musculus GN=U2af2 PE=1 SV=3	7.7894737	3	4	3	53.483	9.09	2300000		1400000	7.80
Q91V09	WD repeat-containing protein 13 OS=Mus musculus GN=Wdr13 PE=1 SV=1	4.3298969	1	1	1	53.63	9.14				3.69
Q61234	Alpha-1-syntrophin OS=Mus musculus GN=Snta1 PE=1 SV=1	1.3916501	1	1	1	53.632	6.86	1200000			2.03

P20152	Vimentin OS=Mus musculus GN=Vim PE=1 SV=3	31.759657	14	31	13	53.655	5.12	4400000	7000000	16000000	87.49
P28658	Ataxin-10 OS=Mus musculus GN=Atxn10 PE=1 SV=2	7.1578947	3	5	3	53.673	5.25	1500000	890000	2100000	16.79
Q80U23	Syntaphilin OS=Mus musculus GN=Snph PE=1 SV=3	9.8989899	3	6	3	53.72	5.9	1300000		2200000	18.35
Q64735	Complement component receptor 1-like protein OS=Mus musculus GN=Cr1l PE=1 SV=1	2.2774327	1	2	1	53.728	6.65	1000000	570000		4.95
Q9WUM3	Coronin-1B OS=Mus musculus GN=Coro1b PE=1 SV=1	14.46281	6	16	6	53.878	5.78	3700000	2800000	5800000	53.16
P47740	Fatty aldehyde dehydrogenase OS=Mus musculus GN=Aldh3a2 PE=1 SV=2	5.5785124	2	5	2	53.936	8.35	3600000	1200000	2100000	16.23
P17809	Solute carrier family 2, facilitated glucose transporter member 1 OS=Mus musculus GN=S	5.0813008	2	5	2	53.949	8.87	21000000	6100000	3800000	16.33
Q8BKZ9	Pyruvate dehydrogenase protein X component, mitochondrial OS=Mus musculus GN=Pdl	11.576846	4	15	4	53.965	7.75	5600000	4100000	6300000	46.19
O35114	Lysosome membrane protein 2 OS=Mus musculus GN=Scarb2 PE=1 SV=3	5.0209205	2	4	2	54.009	5.1	2000000	1700000	1700000	10.30
Q8BH43	Wiskott-Aldrich syndrome protein family member 2 OS=Mus musculus GN=Wasf2 PE=1 S	2.6156942	1	1	1	54.04	5.53				2.70
P97384	Annexin A11 OS=Mus musculus GN=Anxa11 PE=1 SV=2	2.1868787	1	1	1	54.045	7.66		780000		2.55
P97379	Ras GTPase-activating protein-binding protein 2 OS=Mus musculus GN=G3bp2 PE=1 SV	12.655602	4	5	4	54.055	5.62	2800000		2000000	14.64
P11798	Calcium/calmodulin-dependent protein kinase type II subunit alpha OS=Mus musculus GN	43.305439	18	80	14	54.081	7.08	250000000	73000000	270000000	252.20
P63080	Gamma-aminobutyric acid receptor subunit beta-3 OS=Mus musculus GN=Gabrb3 PE=2	8.4566596	3	7	2	54.131	9.1	3600000	1200000	3500000	22.79
P54823	Probable ATP-dependent RNA helicase DDX6 OS=Mus musculus GN=Ddx6 PE=1 SV=1	4.3478261	2	2	2	54.157	8.66	520000		880000	4.58
Q6PDL0	Cytoplasmic dynein 1 light intermediate chain 2 OS=Mus musculus GN=Dync1li2 PE=1 SV	16.869919	6	15	6	54.185	6.28	3600000	2100000	4000000	43.62
Q9Z0H4	CUGBP Elav-like family member 2 OS=Mus musculus GN=Celf2 PE=1 SV=1	6.1023622	2	2	2	54.236	8.76		380000	1900000	5.22
O08749	Dihydrolipoyl dehydrogenase, mitochondrial OS=Mus musculus GN=Dld PE=1 SV=2	17.288802	7	20	7	54.238	7.9	18000000	16000000	22000000	61.55
Q91YD9	Neural Wiskott-Aldrich syndrome protein OS=Mus musculus GN=Wasl PE=1 SV=1	8.9820359	4	6	4	54.24	7.93	2800000	660000	1700000	14.11
O55026	Ectonucleoside triphosphate diphosphohydrolase 2 OS=Mus musculus GN=Entpd2 PE=1	4.8484848	2	4	2	54.285	8.37	2000000	830000	1300000	9.09
O88746	Target of Myb protein 1 OS=Mus musculus GN=Tom1 PE=1 SV=1	2.6422764	1	3	1	54.291	4.94	980000	670000	940000	8.33
Q8VCX5	Calcium uptake protein 1, mitochondrial OS=Mus musculus GN=Micu1 PE=1 SV=1	2.7253669	1	3	1	54.318	8.59	3800000	1600000	2500000	10.61
P97807	Fumarate hydratase, mitochondrial OS=Mus musculus GN=Fh PE=1 SV=3	12.426036	6	15	6	54.322	9.04	13000000	2800000	9700000	47.70
P32921	Tryptophan--tRNA ligase, cytoplasmic OS=Mus musculus GN=Wars PE=1 SV=2	8.5239085	3	6	3	54.323	6.89	2700000	1100000	2900000	18.19
O35405	Phospholipase D3 OS=Mus musculus GN=Pld3 PE=1 SV=1	4.3032787	2	4	2	54.354	6.52	4600000	3400000	2700000	10.28
Q61553	Fascin OS=Mus musculus GN=Fscn1 PE=1 SV=4	6.4908722	3	4	3	54.474	6.89	1000000	680000	450000	9.19
Q9Z2H2	Regulator of G-protein signaling 6 OS=Mus musculus GN=Rgs6 PE=1 SV=2	18.432203	6	13	4	54.497	7.42	3000000	1100000	2600000	37.31
Q99K48	Non-POU domain-containing octamer-binding protein OS=Mus musculus GN=Nono PE=1	14.376321	7	26	7	54.506	8.95	8800000	1700000	3900000	71.16
P54285	Voltage-dependent L-type calcium channel subunit beta-3 OS=Mus musculus GN=Cacnb	14.46281	6	10	3	54.538	6.27	4300000	980000	4800000	27.31
Q8CHY7	Tetratricopeptide repeat protein 23 OS=Mus musculus GN=Ttc23 PE=2 SV=1	1.6393443	1	3	1	54.574	7.75		430000	980000	7.08
Q61151	Serine/threonine-protein phosphatase 2A 56 kDa regulatory subunit epsilon isoform OS=M	5.5674518	2	4	2	54.679	6.95	6400000	1800000	3100000	10.51
Q60738	Zinc transporter 1 OS=Mus musculus GN=Slc30a1 PE=1 SV=1	2.1868787	1	1	1	54.681	6.62	800000			2.66
Q80W54	CAAX prenyl protease 1 homolog OS=Mus musculus GN=Zmpste24 PE=1 SV=2	3.3684211	1	1	1	54.699	6.95	1300000			3.13
Q8K0E8	Fibrinogen beta chain OS=Mus musculus GN=Fgb PE=1 SV=1	6.029106	2	4	2	54.718	7.08	1900000	850000	1600000	12.23
O54829	Regulator of G-protein signaling 7 OS=Mus musculus GN=Rgs7 PE=1 SV=2	16.84435	8	20	6	54.758	8.13	16000000	4800000	6300000	50.67
Q3UHL1	CaM kinase-like vesicle-associated protein OS=Mus musculus GN=Camkv PE=1 SV=2	49.414063	17	55	17	54.785	5.54	31000000	11000000	24000000	189.21
Q8BN59	La-related protein 6 OS=Mus musculus GN=Larp6 PE=1 SV=1	2.8455285	1	1	1	54.839	7.9	410000			2.01
P63056	Noelin-3 OS=Mus musculus GN=Olfm3 PE=1 SV=1	3.1380753	1	1	1	54.851	8.15			360000	2.91
Q8VEH8	Endoplasmic reticulum lectin 1 OS=Mus musculus GN=Erlec1 PE=1 SV=1	6.8322981	3	7	3	54.871	6.25	1600000	1200000	1700000	18.63
Q8BH44	Coronin-2B OS=Mus musculus GN=Coro2b PE=1 SV=2	3.5416667	2	5	2	54.901	8.27	5500000	2000000	5300000	13.20
P28661	Septin-4 OS=Mus musculus GN=Sept4 PE=1 SV=1	14.435146	5	20	4	54.901	5.87	2700000	3500000	7500000	56.88
Q8CHX7	Raftlin-2 OS=Mus musculus GN=Rftn2 PE=1 SV=3	10.4	3	4	3	54.937	5.5	950000		1100000	11.66
Q8CBE3	WD repeat-containing protein 37 OS=Mus musculus GN=Wdr37 PE=1 SV=1	15.120968	6	11	6	55.012	7.23	7100000	950000	4400000	33.11
Q63943	Myocyte-specific enhancer factor 2D OS=Mus musculus GN=Mef2d PE=1 SV=2	2.1400778	1	4	1	55.031	7.88	3300000		2200000	10.01
P22723	Gamma-aminobutyric acid receptor subunit gamma-2 OS=Mus musculus GN=Gabrg2 PE	1.6877637	1	2	1	55.063	8.66	4900000	1700000		4.39
Q8K0T4	Katanin p60 ATPase-containing subunit A-like 1 OS=Mus musculus GN=Katnal1 PE=1 SV	4.3032787	2	3	2	55.13	7.09	1500000		1300000	6.34
Q8VHI6	Wiskott-Aldrich syndrome protein family member 3 OS=Mus musculus GN=Wasf3 PE=1 S	4.99002	2	4	2	55.17	6.37	9500000	2600000	5800000	10.12
Q9DC63	F-box only protein 3 OS=Mus musculus GN=Fbxo3 PE=1 SV=1	2.7083333	1	1	1	55.192	5.02			110000	2.06

Q99KP6	Pre-mRNA-processing factor 19 OS=Mus musculus GN=Prpf19 PE=1 SV=1		13.492063	5	10	5	55.204	6.61	3600000	1600000	4700000	27.80
Q99LF4	tRNA-splicing ligase RtcB homolog OS=Mus musculus GN=Rtcb PE=1 SV=1		14.455446	6	16	6	55.214	7.23	9000000	2700000	6500000	45.21
Q8QZV4	Serine/threonine-protein kinase 32C OS=Mus musculus GN=Stk32c PE=1 SV=1		2.4590164	1	1	1	55.227	6.16	1400000			3.30
Q8C522	Endonuclease domain-containing 1 protein OS=Mus musculus GN=Endod1 PE=1 SV=2		2.5948104	1	3	1	55.227	6.16	5600000	1600000	3700000	10.38
P70288	Histone deacetylase 2 OS=Mus musculus GN=Hdac2 PE=1 SV=1		2.4590164	1	2	1	55.267	5.91	4300000		3000000	6.12
Q6P6M7	O-phosphoseryl-tRNA(Sec) selenium transferase OS=Mus musculus GN=Sepsecs PE=1		1.3888889	1	1	1	55.29	8.06	27000000			2.21
P46660	Alpha-internexin OS=Mus musculus GN=Ina PE=1 SV=3		46.307385	26	77	22	55.349	5.4	21000000	20000000	47000000	219.84
O88998	Noelin OS=Mus musculus GN=Olfm1 PE=1 SV=1		10.103093	4	8	4	55.363	6.95	5100000	2100000	5700000	25.23
Q9WTR6	Cystine/glutamate transporter OS=Mus musculus GN=Slc7a11 PE=1 SV=1		2.7888446	1	1	1	55.42	9.23	69000			1.83
Q8C1Y8	Vacuolar fusion protein CCZ1 homolog OS=Mus musculus GN=Ccz1 PE=1 SV=1		2.7083333	1	1	1	55.469	6.02		970000		2.28
Q7TPM6	Fibronectin type III and SPRY domain-containing protein 1 OS=Mus musculus GN=Fsd1 F		6.6532258	3	5	3	55.49	6.67	450000		550000	9.16
Q9DCP2	Sodium-coupled neutral amino acid transporter 3 OS=Mus musculus GN=Slc38a3 PE=1 S		1.5841584	1	1	1	55.555	7.09			3400000	2.31
Q5SRX1	TOM1-like protein 2 OS=Mus musculus GN=Tom1l2 PE=1 SV=1		12.820513	5	11	5	55.628	4.82	4500000	3400000	5800000	29.62
P14576	Signal recognition particle 54 kDa protein OS=Mus musculus GN=Srp54 PE=1 SV=2		4.1666667	2	4	2	55.684	8.75	1400000		500000	9.51
P24547	Inosine-5'-monophosphate dehydrogenase 2 OS=Mus musculus GN=Impdh2 PE=1 SV=2		2.3346304	1	1	1	55.78	7.28	1200000			3.12
Q9WVE8	Protein kinase C and casein kinase substrate in neurons protein 2 OS=Mus musculus GN		15.020576	8	15	8	55.798	5.2	5400000	4400000	7200000	32.55
Q8VBY2	Calcium/calmodulin-dependent protein kinase kinase 1 OS=Mus musculus GN=Camkk1 F		7.9207921	3	5	3	55.802	5.99	1100000		1800000	15.04
Q9D024	Coiled-coil domain-containing protein 47 OS=Mus musculus GN=Ccdc47 PE=1 SV=2		5.5900621	2	5	2	55.808	4.84	1800000	1200000	2100000	14.25
Q8BVE3	V-type proton ATPase subunit H OS=Mus musculus GN=Atp6v1h PE=1 SV=1		28.15735	11	35	11	55.819	6.61	16000000	9500000	18000000	111.15
Q9Z127	Large neutral amino acids transporter small subunit 1 OS=Mus musculus GN=Slc7a5 PE=		8.3984375	4	8	4	55.836	7.9	2900000	1200000	1700000	26.61
Q14CN4-1	SWISS-PROT:Q14CN4-1 Tax_Id=9606 Gene_Symbol=KRT72 Isoform 1 of	x	9.1976517	5	20	1	55.842	6.89	11000000	6800000	13000000	50.29
Q8BWF0	Succinate-semialdehyde dehydrogenase, mitochondrial OS=Mus musculus GN=Aldh5a1		15.678776	7	17	7	55.933	8.25	13000000	5800000	10000000	53.58
Q8BJY1	26S proteasome non-ATPase regulatory subunit 5 OS=Mus musculus GN=Psmd5 PE=1 S		1.984127	1	2	1	55.937	5.21	910000			5.87
Q8VE97	Serine/arginine-rich splicing factor 4 OS=Mus musculus GN=Srsf4 PE=2 SV=1		16.973415	8	14	5	55.947	11.4	7600000		5500000	38.90
Q9D0K2	Succinyl-CoA:3-ketoacid coenzyme A transferase 1, mitochondrial OS=Mus musculus GN		14.615385	7	27	7	55.953	8.53	18000000	7300000	13000000	71.09
Q9JMA1	Ubiquitin carboxyl-terminal hydrolase 14 OS=Mus musculus GN=Usp14 PE=1 SV=3		7.0993915	3	5	3	55.966	5.24	3300000	1300000	2400000	16.93
P41242	Megakaryocyte-associated tyrosine-protein kinase OS=Mus musculus GN=Matk PE=1 SV		3.960396	1	1	1	56.021	8.81	1100000			4.11
O35874	Neutral amino acid transporter A OS=Mus musculus GN=Slc1a4 PE=1 SV=1		16.353383	6	17	5	56.026	5.87	18000000	9600000	12000000	47.14
Q9CPY7	Cytosol aminopeptidase OS=Mus musculus GN=Lap3 PE=1 SV=3		2.8901734	1	1	1	56.106	7.72		380000		3.05
P56480	ATP synthase subunit beta, mitochondrial OS=Mus musculus GN=Atp5b PE=1 SV=2		60.302457	24	203	24	56.265	5.34	210000000	180000000	260000000	700.89
Q6PD03	Serine/threonine-protein phosphatase 2A 56 kDa regulatory subunit alpha isoform OS=Mu		2.0576132	1	3	1	56.311	7.03	1500000	490000	1000000	6.47
Q6PHZ2	Calcium/calmodulin-dependent protein kinase type II subunit delta OS=Mus musculus GN		23.647295	10	33	5	56.334	7.25	14000000	3200000	12000000	109.15
Q3UYC0	Protein phosphatase 1H OS=Mus musculus GN=Ppm1h PE=1 SV=1		15.789474	6	11	6	56.345	6.73	2600000	2300000	4700000	29.56
P16388	Potassium voltage-gated channel subfamily A member 1 OS=Mus musculus GN=Kcna1 F		5.0505051	2	3	2	56.373	5.14	260000		940000	7.59
P97797	Tyrosine-protein phosphatase non-receptor type substrate 1 OS=Mus musculus GN=Sirpa		13.255361	6	21	6	56.39	8.15	20000000	15000000	15000000	63.22
P61922	4-aminobutyrate aminotransferase, mitochondrial OS=Mus musculus GN=Abat PE=1 SV=		16.6	6	20	6	56.416	8.09	14000000	2700000	7800000	71.95
Q9QYI3	DnaJ homolog subfamily C member 7 OS=Mus musculus GN=Dnajc7 PE=1 SV=2		8.9068826	4	9	4	56.44	6.49	2000000	690000	2200000	26.06
Q9CQF9	Prenylcysteine oxidase OS=Mus musculus GN=Pcyox1 PE=1 SV=1		13.267327	5	12	5	56.459	6.92	2800000	960000	2200000	41.30
P47738	Aldehyde dehydrogenase, mitochondrial OS=Mus musculus GN=Aldh2 PE=1 SV=1		21.001927	9	21	9	56.502	7.62	3400000	3000000	5700000	55.61
P62814	V-type proton ATPase subunit B, brain isoform OS=Mus musculus GN=Atp6v1b2 PE=1 S'		45.988258	20	74	20	56.515	5.81	76000000	51000000	72000000	214.47
Q61753	D-3-phosphoglycerate dehydrogenase OS=Mus musculus GN=Phgdh PE=1 SV=3		6.1913696	3	5	3	56.549	6.54	4600000	2200000	2500000	11.77
Q99K28	ADP-ribosylation factor GTPase-activating protein 2 OS=Mus musculus GN=Arfgap2 PE=		4.0384615	2	3	2	56.563	8.18		380000		7.11
Q9JLT4	Thioredoxin reductase 2, mitochondrial OS=Mus musculus GN=Txnrd2 PE=1 SV=4		2.2900763	1	2	1	56.568	8.46		2300000	7900000	6.05
Q8R1Q8	Cytoplasmic dynein 1 light intermediate chain 1 OS=Mus musculus GN=Dync1li1 PE=1 S'		16.443595	6	13	6	56.579	6.42	4400000	1600000	3400000	34.96
P27773	Protein disulfide-isomerase A3 OS=Mus musculus GN=Pdia3 PE=1 SV=2		36.435644	14	40	14	56.643	6.21	25000000	15000000	23000000	120.48
P63141	Potassium voltage-gated channel subfamily A member 2 OS=Mus musculus GN=Kcna2 F		3.6072144	1	1	1	56.664	4.86	790000			4.10
O35841	Apoptosis inhibitor 5 OS=Mus musculus GN=Api5 PE=1 SV=2		5.7539683	2	2	2	56.75	5.92	1100000			6.48
Q9WVK8	Cholesterol 24-hydroxylase OS=Mus musculus GN=Cyp46a1 PE=1 SV=1		14.2	6	13	6	56.777	8.82	7700000	1500000	4300000	39.92

Q60676	Serine/threonine-protein phosphatase 5 OS=Mus musculus GN=Ppp5c PE=1 SV=3		4.2084168	2	5	2	56.84	6.2	2700000	1800000	2700000	11.96
Q8BYN5	FSD1-like protein OS=Mus musculus GN=Fsd1l PE=1 SV=2		2.9585799	1	1	1	56.85	6.7		170000		3.27
O70310	Glycylpeptide N-tetradecanoyltransferase 1 OS=Mus musculus GN=Nmt1 PE=1 SV=1		2.6209677	1	4	1	56.852	8	190000	310000	390000	10.78
Q8N1N4-2	SWISS-PROT:Q8N1N4-2 Tax_Id=9606 Gene_Symbol=KRT78 Isoform 2 of	x	14.779271	7	16	5	56.93	6.02		2000000	5400000	40.15
Q8CIM7	Cytochrome P450 2D26 OS=Mus musculus GN=Cyp2d26 PE=1 SV=1		2.4	1	3	1	56.939	6.64	1200000	280000	740000	6.18
Q91ZJ5	UTP--glucose-1-phosphate uridylyltransferase OS=Mus musculus GN=Ugp2 PE=1 SV=3		4.7244094	2	3	2	56.944	7.61		3000000	3500000	6.65
Q99KK9	Probable histidine--tRNA ligase, mitochondrial OS=Mus musculus GN=Hars2 PE=1 SV=1		3.3663366	2	3	1	56.949	8.32	1500000			6.45
Q9ERA0	Alpha-globin transcription factor CP2 OS=Mus musculus GN=Tfcp2 PE=1 SV=1		2.3904382	1	3	1	56.996	5.54	370000	180000	480000	8.89
Q9JKF6	Nectin-1 OS=Mus musculus GN=Nectin1 PE=1 SV=3		6.2135922	2	6	2	56.999	6.35	2700000	1300000	2500000	20.42
P09103	Protein disulfide-isomerase OS=Mus musculus GN=P4hb PE=1 SV=2		22.789784	9	19	9	57.023	4.88	3300000	2900000	4800000	52.47
Q922B2	Aspartate--tRNA ligase, cytoplasmic OS=Mus musculus GN=Dars PE=1 SV=2		17.365269	7	12	7	57.111	6.49	6200000	1900000	4000000	37.50
Q5XJY5	Coatomer subunit delta OS=Mus musculus GN=Arcn1 PE=1 SV=2		10.763209	5	11	5	57.193	6.21	2600000	1300000	1700000	29.03
Q9CXG3	Peptidyl-prolyl cis-trans isomerase-like 4 OS=Mus musculus GN=Ppil4 PE=1 SV=2		2.2357724	1	1	1	57.195	6.11			1100000	2.24
Q3THK3	General transcription factor IIF subunit 1 OS=Mus musculus GN=Gtf2f1 PE=1 SV=2		3.1496063	1	1	1	57.206	7.01			250000	2.58
O35633	Vesicular inhibitory amino acid transporter OS=Mus musculus GN=Slc32a1 PE=1 SV=3		13.714286	5	12	5	57.344	6.64	15000000	3900000	8000000	46.24
Q9R0H5	Keratin, type II cytoskeletal 71 OS=Mus musculus GN=Krt71 PE=1 SV=1	x	16.603053	9	31	3	57.347	6.99			3400000	89.40
Q9ESX5	H/ACA ribonucleoprotein complex subunit 4 OS=Mus musculus GN=Dkc1 PE=1 SV=4		5.697446	2	3	2	57.365	9.28	2300000		1500000	8.64
Q61035	Histidine--tRNA ligase, cytoplasmic OS=Mus musculus GN=Hars PE=1 SV=2		5.5009823	3	6	2	57.396	6	2100000	840000	1800000	14.23
Q91YW3	DnaJ homolog subfamily C member 3 OS=Mus musculus GN=Dnajc3 PE=1 SV=1		3.968254	2	2	2	57.428	5.85		670000	460000	4.24
P80314	T-complex protein 1 subunit beta OS=Mus musculus GN=Cct2 PE=1 SV=4		15.88785	7	18	7	57.441	6.4	4900000	2400000	3100000	51.35
Q923M0	Protein phosphatase 1 regulatory subunit 16A OS=Mus musculus GN=Ppp1r16a PE=1 SV		3.2442748	1	1	1	57.494	5.81			1000000	1.85
Q6NS82	Protein FAM134A OS=Mus musculus GN=Fam134a PE=1 SV=2		2.4029575	1	3	1	57.506	4.46	4700000	1700000	3700000	12.69
Q8JZK9	Hydroxymethylglutaryl-CoA synthase, cytoplasmic OS=Mus musculus GN=Hmgcs1 PE=1		3.4615385	1	2	1	57.532	5.99	510000	190000		6.88
Q8C0C7	Phenylalanine--tRNA ligase alpha subunit OS=Mus musculus GN=Farsa PE=1 SV=1		4.7244094	2	2	2	57.563	8.28	5200000	820000		6.21
Q6P5F7	Protein tweety homolog 3 OS=Mus musculus GN=Ttyh3 PE=1 SV=1		3.0534351	2	4	2	57.677	5.85	2900000	710000	1500000	15.38
Q80XA6	RalBP1-associated Eps domain-containing protein 2 OS=Mus musculus GN=Reps2 PE=1		5.3742802	2	3	1	57.714	7.53	1900000		3600000	9.59
O35344	Importin subunit alpha-4 OS=Mus musculus GN=Kpna3 PE=1 SV=1		9.2130518	4	7	3	57.737	4.94	1100000	450000	900000	15.93
Q9D0F3	Protein ERGIC-53 OS=Mus musculus GN=Lman1 PE=1 SV=1		5.9961315	2	2	2	57.753	6.34	1700000			5.77
P52480	Pyruvate kinase PKM OS=Mus musculus GN=Pkm PE=1 SV=4		46.13936	22	70	22	57.808	7.47	130000000	63000000	120000000	247.94
Q9QXW9	Large neutral amino acids transporter small subunit 2 OS=Mus musculus GN=Slc7a8 PE=		7.3446328	3	3	3	57.836	6.67	2200000			8.73
Q9EQ20	Methylmalonate-semialdehyde dehydrogenase [acylating], mitochondrial OS=Mus muscul		11.775701	4	8	4	57.879	8.07	2200000	690000	1200000	24.27
O35343	Importin subunit alpha-3 OS=Mus musculus GN=Kpna4 PE=1 SV=1		3.2629559	2	5	1	57.887	4.94	1800000	710000	1500000	10.34
Q8CIN4	Serine/threonine-protein kinase PAK 2 OS=Mus musculus GN=Pak2 PE=1 SV=1		8.9694656	3	9	1	57.894	5.77	1100000	610000		25.28
P80317	T-complex protein 1 subunit zeta OS=Mus musculus GN=Cct6a PE=1 SV=3		19.397363	10	24	10	57.968	7.08	8100000	4100000	6000000	61.86
P80315	T-complex protein 1 subunit delta OS=Mus musculus GN=Cct4 PE=1 SV=3		9.6474954	4	9	4	58.03	8.02	5800000	2000000	3300000	23.95
G3XA57	Rab11 family-interacting protein 2 OS=Mus musculus GN=Rab11fip2 PE=1 SV=1		2.9296875	1	2	1	58.171	9.45	620000		500000	5.11
Q61466	SWI/SNF-related matrix-associated actin-dependent regulator of chromatin subfamily D m		5.0485437	2	2	2	58.208	9.25			810000	3.95
Q9DC61	Mitochondrial-processing peptidase subunit alpha OS=Mus musculus GN=Pmpca PE=1 S		3.6259542	1	1	1	58.242	6.83			600000	3.11
Q8C3Q5	Protein shisa-7 OS=Mus musculus GN=Shisa7 PE=1 SV=3		3.046595	1	2	1	58.297	9.82	850000	400000		5.16
P10852	4F2 cell-surface antigen heavy chain OS=Mus musculus GN=Slc3a2 PE=1 SV=1		32.129278	15	48	15	58.3	5.91	63000000	27000000	45000000	130.66
Q9DAW6	U4/U6 small nuclear ribonucleoprotein Prp4 OS=Mus musculus GN=Prpf4 PE=1 SV=1		3.0710173	1	1	1	58.333	7.28	1100000			3.12
P26638	Serine--tRNA ligase, cytoplasmic OS=Mus musculus GN=Sars PE=1 SV=3		8.7890625	3	5	3	58.352	6.3	5900000	1900000	4200000	15.98
Q99MN9	Propionyl-CoA carboxylase beta chain, mitochondrial OS=Mus musculus GN=Pccb PE=1		10.351201	4	8	4	58.372	7.66	3900000	1900000	4300000	25.69
Q9CWK8	Sorting nexin-2 OS=Mus musculus GN=Snx2 PE=1 SV=2		12.716763	6	14	5	58.435	5.12	2400000	1200000	2000000	41.79
Q8BW75	Amine oxidase [flavin-containing] B OS=Mus musculus GN=Maob PE=1 SV=4		17.307692	7	18	6	58.52	8.29	4500000	2900000	5000000	52.14
Q8C729	Protein FAM126B OS=Mus musculus GN=Fam126b PE=1 SV=1		4.7169811	2	5	2	58.55	7.72	9700000	3000000	5700000	15.88
P63328	Serine/threonine-protein phosphatase 2B catalytic subunit alpha isoform OS=Mus muscul		35.892514	16	60	12	58.606	5.86	45000000	17000000	50000000	187.51
Q61074	Protein phosphatase 1G OS=Mus musculus GN=Ppm1g PE=1 SV=3		23.062731	6	12	6	58.691	4.39	2100000	400000	810000	37.10

Q8R326	Paraspeckle component 1 OS=Mus musculus GN=Pspc1 PE=1 SV=1		7.456979	3	7	3	58.721	6.67	4800000	2100000	7900000	19.84
O08795	Glucosidase 2 subunit beta OS=Mus musculus GN=Prkcsh PE=1 SV=1		15.163148	6	18	6	58.756	4.46	4700000	4100000	6300000	53.59
Q9D554	Splicing factor 3A subunit 3 OS=Mus musculus GN=Sf3a3 PE=1 SV=2		11.576846	3	6	3	58.805	5.34	3200000		2000000	19.52
Q8C166	Copine-1 OS=Mus musculus GN=Cpne1 PE=1 SV=1		1.6791045	1	3	1	58.849	5.66	540000	220000	430000	6.20
Q32MB2	TREMBL:Q32MB2;Q86Y46 Tax_Id=9606 Gene_Symbol=KRT73 Keratin-73	x	8.5185185	5	23	0	58.887	7.23				55.70
Q80SW1	Putative adenosylhomocysteinase 2 OS=Mus musculus GN=Ahcyl1 PE=1 SV=1		23.584906	12	39	3	58.913	6.89	14000000	3300000	5500000	100.87
Q9WV80	Sorting nexin-1 OS=Mus musculus GN=Snx1 PE=1 SV=1		10.91954	5	13	4	58.916	5.22	2800000	1700000	2600000	36.35
Q91WQ3	Tyrosine--tRNA ligase, cytoplasmic OS=Mus musculus GN=Yars PE=1 SV=3		9.6590909	5	5	5	59.068	7.01	1300000	1100000	1200000	12.02
Q9Z2A5	Arginyl-tRNA--protein transferase 1 OS=Mus musculus GN=Ate1 PE=1 SV=2		2.3255814	1	1	1	59.108	8.19	800000			2.64
Q6NVF9	Cleavage and polyadenylation specificity factor subunit 6 OS=Mus musculus GN=Cpsf6 P		2.5408348	1	2	1	59.116	7.15	3200000		720000	5.90
P48453	Serine/threonine-protein phosphatase 2B catalytic subunit beta isoform OS=Mus musculu		29.52381	11	41	7	59.136	5.91	4000000	3400000	6200000	117.65
P63137	Gamma-aminobutyric acid receptor subunit beta-2 OS=Mus musculus GN=Gabrb2 PE=1		11.132813	4	9	3	59.158	9.31	2900000	1400000	2600000	26.43
Q8BKX1	Brain-specific angiogenesis inhibitor 1-associated protein 2 OS=Mus musculus GN=Baiap		32.71028	14	31	14	59.2	9.04	13000000	7300000	8400000	87.38
Q00612	Glucose-6-phosphate 1-dehydrogenase X OS=Mus musculus GN=G6pdx PE=1 SV=3		1.3592233	1	1	1	59.225	6.49	950000			1.84
Q8VH51	RNA-binding protein 39 OS=Mus musculus GN=Rbm39 PE=1 SV=2		7.1698113	3	6	3	59.37	10.1	4500000		2800000	19.13
P13645	SWISS-PROT:P13645 Tax_Id=9606 Gene_Symbol=KRT10 Keratin, type I c	x	47.048904	26	164	21	59.475	5.21	730000000	700000000	1E+09	510.74
P42932	T-complex protein 1 subunit theta OS=Mus musculus GN=Cct8 PE=1 SV=3		19.708029	9	26	9	59.518	5.62	5400000	2600000	3600000	72.28
Q8C0P5	Coronin-2A OS=Mus musculus GN=Coro2a PE=2 SV=1		4.5801527	2	3	1	59.535	7.71		760000		7.61
Q64133	Amine oxidase [flavin-containing] A OS=Mus musculus GN=Maoa PE=1 SV=3		17.870722	8	17	7	59.564	7.81	8400000	3000000	3500000	45.16
Q923T9	Calcium/calmodulin-dependent protein kinase type II subunit gamma OS=Mus musculus C		22.495274	10	29	6	59.569	7.58	22000000	2100000	20000000	92.10
P56564	Excitatory amino acid transporter 1 OS=Mus musculus GN=Slc1a3 PE=1 SV=2		17.679558	7	49	6	59.584	8.4	46000000	18000000	35000000	188.41
P80316	T-complex protein 1 subunit epsilon OS=Mus musculus GN=Cct5 PE=1 SV=1		17.744917	9	21	9	59.586	6.02	7700000	4500000	6200000	58.37
P80313	T-complex protein 1 subunit eta OS=Mus musculus GN=Cct7 PE=1 SV=1		12.5	7	17	7	59.614	7.84	7900000	2600000	3800000	40.74
Q99JF8	PC4 and SFRS1-interacting protein OS=Mus musculus GN=Psip1 PE=1 SV=1		15.909091	6	20	6	59.66	9.13	15000000	350000	8900000	70.20
Q922P9	Putative oxidoreductase GLYR1 OS=Mus musculus GN=Glyr1 PE=1 SV=1		2.5641026	1	2	1	59.678	9.22	700000		530000	5.10
Q9D0L4	Uncharacterized aarF domain-containing protein kinase 1 OS=Mus musculus GN=Adck1		3.2380952	1	3	1	59.698	8.05	1700000	590000	630000	11.31
Q8BI72	CDKN2A-interacting protein OS=Mus musculus GN=Cdkn2aip PE=1 SV=1		5.8614565	2	4	2	59.708	9.16	840000		370000	11.51
Q03265	ATP synthase subunit alpha, mitochondrial OS=Mus musculus GN=Atp5a1 PE=1 SV=1		44.303797	23	111	23	59.716	9.19	310000000	200000000	230000000	359.10
P70297	Signal transducing adapter molecule 1 OS=Mus musculus GN=Stam PE=1 SV=3		5.2919708	2	4	2	59.733	4.84	1700000	990000	1600000	11.78
P24270	Catalase OS=Mus musculus GN=Cat PE=1 SV=4		5.1233397	2	2	2	59.758	7.88		260000	3300000	5.31
Q9CTY5	Calcium uptake protein 3, mitochondrial OS=Mus musculus GN=Micu3 PE=2 SV=2		6.500956	3	6	3	59.772	7.15	1400000	330000	1400000	15.84
P97492	Regulator of G-protein signaling 14 OS=Mus musculus GN=Rgs14 PE=1 SV=2		2.0109689	1	1	1	59.81	7.43	400000			2.21
Q99J45	Nuclear receptor-binding protein OS=Mus musculus GN=Nrbp1 PE=1 SV=1		2.2429907	1	1	1	59.828	5.08	330000			2.87
O35345	Importin subunit alpha-7 OS=Mus musculus GN=Kpna6 PE=1 SV=2		6.1567164	2	4	1	59.926	4.94	1400000	510000		12.04
O70324	Monocarboxylate transporter 8 OS=Mus musculus GN=Slc16a2 PE=1 SV=2		4.2201835	1	1	1	59.986	5.59	630000			2.61
P48668	SWISS-PROT:P48668 Tax_Id=9606 Gene_Symbol=KRT6C Keratin, type II	x	38.297872	24	65	8	59.988	8	3200000	1200000	16000000	167.49
Q8BMP6	Golgi resident protein GCP60 OS=Mus musculus GN=Acbd3 PE=1 SV=3		2.8571429	1	2	1	60.144	5.11			1400000	5.73
Q60960	Importin subunit alpha-5 OS=Mus musculus GN=Kpna1 PE=1 SV=2		6.133829	2	4	1	60.144	5.01	240000		1000000	12.25
Q80VP1	Epsin-1 OS=Mus musculus GN=Epn1 PE=1 SV=3		5.3913043	3	9	3	60.175	4.81	4800000	4100000	6200000	23.90
Q3UEB3	Poly(U)-binding-splicing factor PUF60 OS=Mus musculus GN=Puf60 PE=1 SV=2		6.7375887	3	8	3	60.211	5.29	3200000	1800000	2600000	25.33
Q8VBW6	NEDD8-activating enzyme E1 regulatory subunit OS=Mus musculus GN=Nae1 PE=1 SV=		5.6179775	2	4	2	60.236	5.52	2100000	820000	1500000	10.89
O88939	Zinc finger and BTB domain-containing protein 7A OS=Mus musculus GN=Zbtb7a PE=1 S		6.8541301	2	2	2	60.243	5.12				6.47
Q6DFW4	Nucleolar protein 58 OS=Mus musculus GN=Nop58 PE=1 SV=1		2.238806	1	2	1	60.305	8.34	21000000			5.57
Q71M36	Chondroitin sulfate proteoglycan 5 OS=Mus musculus GN=Cspg5 PE=1 SV=2		12.367491	6	22	6	60.368	4.54	25000000	15000000	25000000	65.19
P11983	T-complex protein 1 subunit alpha OS=Mus musculus GN=Tcp1 PE=1 SV=3		14.388489	7	16	7	60.411	6.16	7400000	2500000	2600000	43.83
P28652	Calcium/calmodulin-dependent protein kinase type II subunit beta OS=Mus musculus GN=		39.483395	14	65	9	60.423	7.28	51000000	14000000	52000000	225.55
Q8R4G0	Netrin-G1 OS=Mus musculus GN=Ntng1 PE=1 SV=2		5.5658627	2	4	2	60.526	6.24	740000	590000	1400000	10.41
Q91YH5	Atlastin-3 OS=Mus musculus GN=Atl3 PE=1 SV=1		3.8817006	2	3	2	60.537	6.1	810000	230000		4.46

Q9JLB9	Nectin-3 OS=Mus musculus GN=Nectin3 PE=1 SV=1		3.6429872	1	3	1	60.545	6.54	1600000	1200000	1700000	10.25
Q9WVK4	EH domain-containing protein 1 OS=Mus musculus GN=Ehd1 PE=1 SV=1		8.988764	4	9	3	60.565	6.83	1900000	1600000	2000000	25.21
P80318	T-complex protein 1 subunit gamma OS=Mus musculus GN=Cct3 PE=1 SV=1		21.651376	10	21	10	60.591	6.7	6500000	3000000	2300000	55.64
P05480	Neuronal proto-oncogene tyrosine-protein kinase Src OS=Mus musculus GN=Src PE=1 S		6.284658	3	6	3	60.606	7.84	3100000	3000000	2200000	18.34
Q9EP89	Serine beta-lactamase-like protein LACTB, mitochondrial OS=Mus musculus GN=Lactb P		3.9927405	2	5	2	60.667	8.9	3100000	2500000	2700000	13.10
P14685	26S proteasome non-ATPase regulatory subunit 3 OS=Mus musculus GN=Psmd3 PE=1 S		18.679245	8	23	8	60.68	8.44	7100000	1700000	4200000	68.35
O88643	Serine/threonine-protein kinase PAK 1 OS=Mus musculus GN=Pak1 PE=1 SV=1		19.449541	8	23	6	60.699	5.74	5600000	2700000	6100000	67.79
P54830	Tyrosine-protein phosphatase non-receptor type 5 OS=Mus musculus GN=Ptpn5 PE=1 S		1.4787431	1	2	1	60.776	5.2	1500000		1000000	4.75
Q9QXY6	EH domain-containing protein 3 OS=Mus musculus GN=Ehd3 PE=1 SV=2		24.299065	10	28	9	60.783	6.46	9800000	6000000	8100000	90.72
Q60996	Serine/threonine-protein phosphatase 2A 56 kDa regulatory subunit gamma isoform OS=I		2.480916	1	2	1	60.785	6.64	2300000		1500000	5.59
Q3TEA8	Heterochromatin protein 1-binding protein 3 OS=Mus musculus GN=Hp1bp3 PE=1 SV=1		23.104693	10	15	10	60.829	9.7	14000000		8900000	37.36
Q3UTH8	Rho guanine nucleotide exchange factor 9 OS=Mus musculus GN=Arhgef9 PE=1 SV=1		2.1317829	1	2	1	60.888	5.64	1000000		1500000	5.16
P63038	60 kDa heat shock protein, mitochondrial OS=Mus musculus GN=Hspd1 PE=1 SV=1		32.111693	15	48	15	60.917	6.18	24000000	23000000	30000000	159.03
Q3UHD6	Sorting nexin-27 OS=Mus musculus GN=Snx27 PE=1 SV=2		10.575139	5	10	5	60.95	6.4	1600000	930000	1600000	25.97
P20060	Beta-hexosaminidase subunit beta OS=Mus musculus GN=Hexb PE=1 SV=2		1.4925373	1	1	1	61.077	8.12			520000	2.04
Q3UV70	[Pyruvate dehydrogenase [acetyl-transferring]]-phosphatase 1, mitochondrial OS=Mus mu		4.4609665	2	3	2	61.142	6.67		1600000	2900000	11.14
Q01065	Calcium/calmodulin-dependent 3',5'-cyclic nucleotide phosphodiesterase 1B OS=Mus mu		4.1121495	2	4	1	61.187	5.72	2100000			11.76
Q64516	Glycerol kinase OS=Mus musculus GN=Gk PE=1 SV=2		8.9445438	5	11	5	61.188	5.87	2500000	1500000	2800000	25.85
Q60598	Src substrate cortactin OS=Mus musculus GN=Cttn PE=1 SV=2		22.893773	9	19	9	61.212	5.4	12000000	5500000	12000000	56.71
P26443	Glutamate dehydrogenase 1, mitochondrial OS=Mus musculus GN=Glud1 PE=1 SV=1		42.114695	20	73	20	61.298	8	100000000	55000000	93000000	216.84
Q3ULD5	Methylcrotonoyl-CoA carboxylase beta chain, mitochondrial OS=Mus musculus GN=Mccc		9.4138544	3	7	3	61.34	8	2600000	780000	1300000	24.96
Q9D0F9	Phosphoglucomutase-1 OS=Mus musculus GN=Pgm1 PE=1 SV=4		2.8469751	1	2	1	61.38	6.57	1600000	340000		5.40
Q61207	Prosaposin OS=Mus musculus GN=Psap PE=1 SV=2		3.5906643	2	4	2	61.381	5.19	18000000	6400000	15000000	10.32
O88447	Kinesin light chain 1 OS=Mus musculus GN=Klc1 PE=1 SV=3		16.63586	8	17	7	61.412	5.68	3800000	1200000	2100000	46.31
Q9CSN1	SNW domain-containing protein 1 OS=Mus musculus GN=Snw1 PE=1 SV=3		6.3432836	2	3	2	61.438	9.48	980000			6.63
Q8R5H6	Wiskott-Aldrich syndrome protein family member 1 OS=Mus musculus GN=Wasf1 PE=1 S		10.733453	5	11	5	61.471	6.37	12000000	3400000	16000000	35.56
P08551	Neurofilament light polypeptide OS=Mus musculus GN=Nefl PE=1 SV=5		54.327808	28	82	25	61.471	4.64	25000000	8600000	49000000	245.75
Q9EQF6	Dihydropyrimidinase-related protein 5 OS=Mus musculus GN=Dpysl5 PE=1 SV=1		10.638298	5	12	5	61.477	7.09	6200000	1400000	2000000	29.83
Q9WV34	MAGUK p55 subfamily member 2 OS=Mus musculus GN=Mpp2 PE=1 SV=1		26.992754	14	33	14	61.517	6.44	11000000	4800000	9400000	94.60
Q3TXX4	Vesicular glutamate transporter 1 OS=Mus musculus GN=Slc17a7 PE=1 SV=2		14.821429	8	28	7	61.597	7.34	67000000	17000000	39000000	76.01
Q9Z140	Copine-6 OS=Mus musculus GN=Cpne6 PE=1 SV=1		19.928187	10	20	9	61.742	5.59	7000000	3400000	5800000	48.33
Q7Z794	SWISS-PROT:Q7Z794 Tax_Id=9606 Gene_Symbol=KRT77 Keratin 77	x	18.512111	12	36	7	61.764	5.85		980000	4800000	98.92
Q8K406	Leucine-rich repeat LGI family member 3 OS=Mus musculus GN=Lgi3 PE=1 SV=1		6.5693431	3	6	3	61.779	8.16	650000	520000	1200000	19.92
Q5SU73	Coilin OS=Mus musculus GN=Coil PE=1 SV=1		2.4561404	1	1	1	61.879	9	410000			1.90
Q62188	Dihydropyrimidinase-related protein 3 OS=Mus musculus GN=Dpysl3 PE=1 SV=1		20	8	30	4	61.897	6.49	3600000	2400000	3200000	97.39
O35098	Dihydropyrimidinase-related protein 4 OS=Mus musculus GN=Dpysl4 PE=1 SV=1		14.685315	6	14	5	61.922	6.98	5100000	1800000	3000000	47.00
Q8R317	Ubiquilin-1 OS=Mus musculus GN=Ubqln1 PE=1 SV=1		3.7800687	2	4	1	61.937	4.94	1500000	920000	1700000	9.88
P43006	Excitatory amino acid transporter 2 OS=Mus musculus GN=Slc1a2 PE=1 SV=1		20.27972	12	114	12	61.99	6.68	370000000	130000000	310000000	326.88
P35527	SWISS-PROT:P35527 Tax_Id=9606 Gene_Symbol=KRT9 Keratin, type I cy	x	53.932584	28	170	27	62.092	5.3	140000000	44000000	670000000	603.41
Q80VL1	Tudor and KH domain-containing protein OS=Mus musculus GN=Tdrkh PE=1 SV=1		7.1428571	3	4	3	62.095	4.94	1600000		860000	10.34
P97427	Dihydropyrimidinase-related protein 1 OS=Mus musculus GN=Crmp1 PE=1 SV=1		24.825175	12	43	8	62.129	7.12	16000000	8000000	13000000	140.25
O08553	Dihydropyrimidinase-related protein 2 OS=Mus musculus GN=Dpysl2 PE=1 SV=2		67.482517	27	162	22	62.239	6.38	190000000	89000000	170000000	547.31
Q6PAM1	Alpha-taxilin OS=Mus musculus GN=Txlna PE=1 SV=1		3.2490975	1	1	1	62.33	6.74	570000			2.30
P13647	SWISS-PROT:P13647 Tax_Id=9606 Gene_Symbol=KRT5 Keratin, type II c	x	42.20339	29	111	16	62.34	7.74	54000000	32000000	100000000	301.38
Q8BLR2	Copine-4 OS=Mus musculus GN=Cpne4 PE=1 SV=1		3.2315978	2	5	1	62.368	6.33	730000	190000	360000	11.47
P34914	Bifunctional epoxide hydrolase 2 OS=Mus musculus GN=Ephx2 PE=1 SV=2		3.4296029	1	3	1	62.475	6.19	800000	220000	600000	8.83
Q8CJ67	Double-stranded RNA-binding protein Staufen homolog 2 OS=Mus musculus GN=Stau2 F		2.2807018	1	1	1	62.496	9.6	450000			3.82
Q60864	Stress-induced-phosphoprotein 1 OS=Mus musculus GN=Stip1 PE=1 SV=1		17.495396	8	14	8	62.542	6.8	4600000	2700000	5000000	40.32

Q9JLB0	MAGUK p55 subfamily member 6 OS=Mus musculus GN=Mpp6 PE=1 SV=1		11.934901	6	12	6	62.592	6.4	5000000	2400000	3400000	33.07
Q3U0K8	Prolyl 3-hydroxylase OGFOD1 OS=Mus musculus GN=Ogfod1 PE=1 SV=1		9.3577982	2	5	2	62.695	4.91	960000	490000	710000	22.55
P06745	Glucose-6-phosphate isomerase OS=Mus musculus GN=Gpi PE=1 SV=4		23.297491	10	30	10	62.727	8.13	14000000	5600000	12000000	86.82
Q3UV17	Keratin, type II cytoskeletal 2 oral OS=Mus musculus GN=Krt76 PE=1 SV=1		9.2592593	6	22	1	62.806	8.43		310000000	1.6E+09	50.97
Q9Z2D0	Myotubularin-related protein 9 OS=Mus musculus GN=Mtmr9 PE=1 SV=2		1.4678899	1	2	1	62.868	6.62	740000		560000	4.28
Q8VCM8	Nicalin OS=Mus musculus GN=Ncln PE=1 SV=2		4.0852575	2	2	2	62.868	6.49		860000		5.56
Q91WG5	5'-AMP-activated protein kinase subunit gamma-2 OS=Mus musculus GN=Prkag2 PE=1 S		1.7667845	1	1	1	62.91	9.36	1600000			3.07
Q8K4Z0	Leucine-rich repeat LGI family member 2 OS=Mus musculus GN=Lgi2 PE=2 SV=1		2.1818182	1	1	1	62.966	6.54			1800000	2.04
Q8C6B2	Rhotekin OS=Mus musculus GN=Rtkn PE=1 SV=3		2.6595745	1	1	1	62.973	6.67			590000	3.13
Q9Z1Q2	Protein ABHD16A OS=Mus musculus GN=Abhd16a PE=1 SV=3		2.8673835	1	3	1	63.046	8.25	2300000		1300000	9.09
Q8C437	PEX5-related protein OS=Mus musculus GN=Pex5l PE=1 SV=2		12.169312	5	14	5	63.096	5.33	4600000	1600000	4300000	45.31
Q3UHB1	5'-nucleotidase domain-containing protein 3 OS=Mus musculus GN=Nt5dc3 PE=1 SV=1		5.4945055	3	9	3	63.13	8.56	2700000	1300000	1900000	23.21
O08914	Fatty-acid amide hydrolase 1 OS=Mus musculus GN=Faah PE=1 SV=1		1.5544041	1	2	1	63.18	7.87	1000000		470000	4.50
Q5U458	DnaJ homolog subfamily C member 11 OS=Mus musculus GN=Dnajc11 PE=1 SV=2		6.2611807	3	6	3	63.194	8.32	2600000	710000	2000000	15.79
Q3UUG6	TBC1 domain family member 24 OS=Mus musculus GN=Tbc1d24 PE=1 SV=2		7.8431373	4	9	4	63.195	7.24	4800000	1400000	2800000	23.00
Q8C854	Myelin expression factor 2 OS=Mus musculus GN=Myef2 PE=1 SV=1		13.367174	8	17	8	63.254	8.87	12000000	3400000	6000000	50.34
Q8CI51	PDZ and LIM domain protein 5 OS=Mus musculus GN=Pdlim5 PE=1 SV=4		3.7225042	1	1	1	63.259	8.25	360000			2.48
Q920P5	Adenylate kinase isoenzyme 5 OS=Mus musculus GN=Ak5 PE=1 SV=2		3.202847	1	2	1	63.283	5.29		1900000	3300000	6.03
Q64332	Synapsin-2 OS=Mus musculus GN=Syn2 PE=1 SV=2		37.883959	16	67	14	63.333	8.43	31000000	23000000	30000000	239.63
Q8BH66	Atlastin-1 OS=Mus musculus GN=Atl1 PE=1 SV=1		13.978495	8	17	8	63.337	6.49	14000000	3000000	7600000	45.99
Q8BXN9	Transmembrane protein 87A OS=Mus musculus GN=Tmem87a PE=1 SV=1		5.045045	2	4	2	63.339	6.67	1600000	520000	570000	10.91
Q8C4G9	Adhesion G protein-coupled receptor A1 OS=Mus musculus GN=Adgra1 PE=2 SV=1		2.4221453	1	2	1	63.455	7.43	2700000		1300000	5.90
P46061	Ran GTPase-activating protein 1 OS=Mus musculus GN=Rangap1 PE=1 SV=2		5.942275	3	5	3	63.491	4.68	2400000		1100000	13.09
Q505F5	Leucine-rich repeat-containing protein 47 OS=Mus musculus GN=Lrrc47 PE=1 SV=1		6.0240964	3	5	3	63.551	8.1	2100000	720000	1700000	14.66
Q9R1C6	Diacylglycerol kinase epsilon OS=Mus musculus GN=Dgke PE=1 SV=1		6.7375887	3	3	3	63.593	7.44	1300000		1600000	7.37
Q9JIA1	Leucine-rich glioma-inactivated protein 1 OS=Mus musculus GN=Lgi1 PE=1 SV=1		17.235189	9	22	9	63.603	8.02	8000000	5100000	6100000	60.98
Q91YN0	Protein C12orf4 homolog OS=Mus musculus GN=D6Wsu163e PE=1 SV=1		4.3478261	1	1	1	63.604	6.18	1200000			2.90
Q8BMK4	Cytoskeleton-associated protein 4 OS=Mus musculus GN=Ckap4 PE=1 SV=2		11.478261	5	6	5	63.654	5.64	1300000		1300000	15.80
Q9Z2A0	3-phosphoinositide-dependent protein kinase 1 OS=Mus musculus GN=Pdpk1 PE=1 SV=		5.3667263	2	4	2	63.718	7.37	2400000		720000	11.33
Q80WJ7	Protein LYRIC OS=Mus musculus GN=Mtdh PE=1 SV=1		23.143351	10	20	10	63.808	9.33	7000000	1100000	3300000	54.79
Q4VBD2	Transmembrane anterior posterior transformation protein 1 OS=Mus musculus GN=Tapt1		1.7730496	1	1	1	63.853	8.22	990000			2.36
O55176	E3 ubiquitin-protein ligase Praja-1 OS=Mus musculus GN=Pja1 PE=1 SV=3		2.4221453	1	2	1	63.866	4.97	360000		470000	5.51
Q69Z99	Zinc finger protein 512 OS=Mus musculus GN=Znf512 PE=2 SV=2		15.480427	6	9	6	63.867	9.51	1400000		1900000	26.74
P06801	NADP-dependent malic enzyme OS=Mus musculus GN=Me1 PE=1 SV=2		4.8951049	3	5	3	63.913	7.44	1200000		1100000	11.63
Q8BYK6	YTH domain-containing family protein 3 OS=Mus musculus GN=Ythdf3 PE=1 SV=2		2.7350427	1	1	1	63.922	9.04	700000			3.77
Q8R081	Heterogeneous nuclear ribonucleoprotein L OS=Mus musculus GN=Hnrnpl PE=1 SV=2		29.692833	10	37	10	63.923	8.1	46000000	9000000	27000000	140.90
O70194	Eukaryotic translation initiation factor 3 subunit D OS=Mus musculus GN=Eif3d PE=1 SV=		19.890511	8	15	8	63.948	6.05	4200000	610000	2400000	37.99
Q9Z1K5	E3 ubiquitin-protein ligase ARIH1 OS=Mus musculus GN=Arih1 PE=1 SV=3		2.5225225	1	1	1	63.975	5.08			960000	2.29
Q921F4	Heterogeneous nuclear ribonucleoprotein L-like OS=Mus musculus GN=Hnrnpll PE=1 SV		10.829103	4	7	4	64.084	5.85	6800000		4200000	27.60
O08919	Numb-like protein OS=Mus musculus GN=Numbl PE=1 SV=3		4.6357616	2	5	2	64.09	8.82	2000000	270000	960000	14.66
Q9CWJ9	Bifunctional purine biosynthesis protein PURH OS=Mus musculus GN=Atic PE=1 SV=2		2.8716216	1	1	1	64.177	6.76			1800000	2.88
Q62073	Mitogen-activated protein kinase kinase kinase 7 OS=Mus musculus GN=Map3k7 PE=1 S		2.0725389	1	1	1	64.187	6.67			8400000	2.28
Q8BP47	Asparagine--tRNA ligase, cytoplasmic OS=Mus musculus GN=Nars PE=1 SV=2		7.5134168	3	8	3	64.238	5.86	7800000	2000000	5600000	26.65
Q60803	TNF receptor-associated factor 3 OS=Mus musculus GN=Traf3 PE=1 SV=2		4.4091711	2	2	2	64.252	8.05	910000		1100000	5.22
O88910	MAGUK p55 subfamily member 3 OS=Mus musculus GN=Mpp3 PE=1 SV=2		13.556338	6	9	6	64.361	6.34	2000000	380000	1700000	28.44
Q9D6Z1	Nucleolar protein 56 OS=Mus musculus GN=Nop56 PE=1 SV=2		9.137931	4	8	4	64.424	9.14	5000000		3200000	21.90
O08539	Myc box-dependent-interacting protein 1 OS=Mus musculus GN=Bin1 PE=1 SV=1		27.55102	12	39	11	64.43	5.03	26000000	15000000	32000000	132.38
P12035	SWISS-PROT:P12035 Tax_Id=9606 Gene_Symbol=KRT3 Keratin, type II c	x	11.287758	11	35	1	64.465	6.48			670000	85.58

Q61481	Calcium/calmodulin-dependent 3',5'-cyclic nucleotide phosphodiesterase 1A OS=Mus mus		19.115044	8	20	7	64.487	5.97	4400000	2100000	4700000	61.64
Q8BLE7	Vesicular glutamate transporter 2 OS=Mus musculus GN=Slc17a6 PE=1 SV=1		5.4982818	4	14	3	64.519	6.68	6400000	1500000	4700000	44.84
Q8C078	Calcium/calmodulin-dependent protein kinase kinase 2 OS=Mus musculus GN=Camkk2 F		4.0816327	2	2	2	64.576	5.9			1000000	5.11
Q05CL8	La-related protein 7 OS=Mus musculus GN=Larp7 PE=1 SV=2		2.1052632	1	1	1	64.762	9.54			1200000	2.95
Q9D824	Pre-mRNA 3'-end-processing factor FIP1 OS=Mus musculus GN=Fip1l1 PE=1 SV=1		2.2375215	1	1	1	64.92	5.77	1600000			3.28
Q9CWL8	Beta-catenin-like protein 1 OS=Mus musculus GN=Ctnnbl1 PE=1 SV=1		2.8419183	1	1	1	64.939	5.07	2200000			3.52
Q8R366	Immunoglobulin superfamily member 8 OS=Mus musculus GN=Igsf8 PE=1 SV=2		14.238953	6	18	6	64.97	7.99	17000000	9800000	9200000	61.00
P97390	Vacuolar protein sorting-associated protein 45 OS=Mus musculus GN=Vps45 PE=1 SV=1		7.5438596	4	6	4	65.012	8.25	1500000	810000	1300000	15.47
Q61490	CD166 antigen OS=Mus musculus GN=Alcam PE=1 SV=3		19.210978	8	20	8	65.051	6.15	11000000	13000000	23000000	57.05
P48320	Glutamate decarboxylase 2 OS=Mus musculus GN=Gad2 PE=1 SV=1		3.5897436	2	3	1	65.181	6.9		2900000	7200000	10.04
O88291	DBIRD complex subunit ZNF326 OS=Mus musculus GN=Znf326 PE=1 SV=1		7.9310345	3	3	3	65.185	5.19	2300000		4700000	8.34
Q68EF6	Brain-enriched guanylate kinase-associated protein OS=Mus musculus GN=Begain PE=1		2.8333333	1	2	1	65.269	5.87	1000000		720000	7.06
Q91W39	Nuclear receptor coactivator 5 OS=Mus musculus GN=Ncoa5 PE=1 SV=1		2.0725389	1	2	1	65.28	9.82	1800000		1300000	4.02
Q76MZ3	Serine/threonine-protein phosphatase 2A 65 kDa regulatory subunit A alpha isoform OS=I		27.164686	12	31	12	65.281	5.11	24000000	9000000	17000000	93.05
Q8VHR5	Transcriptional repressor p66-beta OS=Mus musculus GN=Gatad2b PE=1 SV=1		2.1885522	1	2	1	65.37	9.7	1100000			5.59
Q8R3Z5	Voltage-dependent L-type calcium channel subunit beta-1 OS=Mus musculus GN=Cacnb		6.5326633	4	7	1	65.446	6.62			810000	18.22
Q8BK03	Mitoguardin-2 OS=Mus musculus GN=Fam73b PE=1 SV=1		2.529511	1	1	1	65.506	5.41	730000			3.12
Q80UG5	Septin-9 OS=Mus musculus GN=Sept9 PE=1 SV=1		17.667238	9	16	9	65.534	8.9	5200000	3500000	4200000	44.29
Q8JZW4	Copine-5 OS=Mus musculus GN=Cpne5 PE=1 SV=1		3.5413153	2	3	1	65.551	5.77			510000	6.81
Q9Z1M8	Protein Red OS=Mus musculus GN=Ik PE=1 SV=2		3.4111311	2	3	2	65.576	6.64	1300000		1000000	6.90
Q6PDI6	Ubiquitin carboxyl-terminal hydrolase MINDY-2 OS=Mus musculus GN=Fam63b PE=1 SV		2.4958403	1	2	1	65.596	4.63	940000		940000	6.75
Q9WUA2	Phenylalanine--tRNA ligase beta subunit OS=Mus musculus GN=Farsb PE=1 SV=2		7.3005093	4	6	4	65.655	7.12	2700000		990000	15.04
P35908	SWISS-PROT:P35908 Tax_Id=9606 Gene_Symbol=KRT2 Keratin, type II c	x	62.015504	45	216	30	65.825	8	300000000	420000000	690000000	680.78
Q80WT0	Junctophilin-4 OS=Mus musculus GN=Jph4 PE=2 SV=1		2.5477707	1	2	1	65.96	6.71	750000			6.15
P04264	SWISS-PROT:P04264 Tax_Id=9606 Gene_Symbol=KRT1 Keratin, type II c	x	63.198758	46	250	37	65.978	8.12	650000000	430000000	1.6E+09	757.71
Q8R4F1	Netrin-G2 OS=Mus musculus GN=Ntng2 PE=1 SV=2		2.37691	1	1	1	66.122	6.13		1000000		2.03
Q2M3X8	Phosphatase and actin regulator 1 OS=Mus musculus GN=Phactr1 PE=1 SV=1		11.896552	5	12	5	66.245	6.96	3900000	1100000	3300000	36.02
O88342	WD repeat-containing protein 1 OS=Mus musculus GN=Wdr1 PE=1 SV=3		32.178218	14	30	14	66.365	6.6	13000000	6000000	12000000	75.82
P50427	Steryl-sulfatase OS=Mus musculus GN=Sts PE=1 SV=1		1.7628205	1	1	1	66.549	8.5		150000000		2.14
Q8QZY1	Eukaryotic translation initiation factor 3 subunit L OS=Mus musculus GN=Eif3l PE=1 SV=1		11.347518	5	10	5	66.57	6.44	2700000	1100000	1900000	29.67
P48318	Glutamate decarboxylase 1 OS=Mus musculus GN=Gad1 PE=1 SV=2		9.9494098	5	8	4	66.605	7.17	4800000		4200000	23.62
O88448	Kinesin light chain 2 OS=Mus musculus GN=Klc2 PE=1 SV=1		11.352254	6	10	5	66.621	7.21	1400000		1400000	27.42
Q925I1	ATPase family AAA domain-containing protein 3 OS=Mus musculus GN=Atad3 PE=1 SV=		9.6446701	6	9	6	66.701	9.29	1900000	1300000	1200000	20.60
Q9Z0X1	Apoptosis-inducing factor 1, mitochondrial OS=Mus musculus GN=Aifm1 PE=1 SV=1		18.137255	8	17	8	66.724	9.17	7300000	3800000	3700000	58.21
P14733	Lamin-B1 OS=Mus musculus GN=Lmnb1 PE=1 SV=3		49.14966	28	66	24	66.745	5.16	11000000	3000000	8000000	183.42
Q3TY86	Apoptosis-inducing factor 3 OS=Mus musculus GN=Aifm3 PE=1 SV=1		1.4876033	1	1	1	66.75	9.04			670000	2.65
Q68FL4	Putative adenosylhomocysteinase 3 OS=Mus musculus GN=Ahcyl2 PE=1 SV=1		18.433931	10	37	1	66.857	7.36	6100000	2000000	5100000	98.46
Q9EP69	Phosphatidylinositide phosphatase SAC1 OS=Mus musculus GN=Sacm1l PE=1 SV=1		4.4293015	3	8	3	66.901	7.3	3600000	920000	2000000	17.98
Q68ED7	CREB-regulated transcription coactivator 1 OS=Mus musculus GN=Crtc1 PE=1 SV=1		4.7619048	1	1	1	66.904	6.05		330000		2.62
P31648	Sodium- and chloride-dependent GABA transporter 1 OS=Mus musculus GN=Slc6a1 PE=		11.853088	4	16	4	66.957	7.96	21000000	9700000	15000000	58.17
Q8BYI8	Protein FAM234B OS=Mus musculus GN=Fam234b PE=1 SV=1		7.6923077	3	6	3	66.989	5.03	2200000	450000	820000	21.34
Q924K8	Metastasis-associated protein MTA3 OS=Mus musculus GN=Mta3 PE=1 SV=1		2.8764805	1	1	1	67.035	8.88	2000000			4.78
Q8BMF3	NADP-dependent malic enzyme, mitochondrial OS=Mus musculus GN=Me3 PE=1 SV=2		4.8013245	2	3	2	67.056	7.83	1800000		1400000	6.90
P17141	Zinc finger protein 37 OS=Mus musculus GN=Zfp37 PE=2 SV=4		2.6936027	1	1	1	67.184	8.82				2.76
P35564	Calnexin OS=Mus musculus GN=Canx PE=1 SV=1		27.411168	15	49	15	67.236	4.64	53000000	20000000	39000000	149.83
P61222	ATP-binding cassette sub-family E member 1 OS=Mus musculus GN=Abce1 PE=1 SV=1		2.0033389	1	1	1	67.271	8.34	4000000			2.82
P21619	Lamin-B2 OS=Mus musculus GN=Lmnb2 PE=1 SV=2		34.731544	20	49	16	67.277	5.5	11000000	3900000	11000000	141.65
Q9QZM0	Ubiquilin-2 OS=Mus musculus GN=Ubqln2 PE=1 SV=2		7.9937304	4	7	3	67.308	5.22	1700000	1300000	1300000	19.27

Q80YN3	Breast carcinoma-amplified sequence 1 homolog OS=Mus musculus GN=Bcas1 PE=1 SV		7.2669826	3	7	3	67.336	6.21	690000	110000	910000	19.42
O88559	Menin OS=Mus musculus GN=Men1 PE=1 SV=2		2.6186579	1	1	1	67.458	6.39	280000			2.84
Q9ET30	Transmembrane 9 superfamily member 3 OS=Mus musculus GN=Tm9sf3 PE=1 SV=1		2.5553663	1	1	1	67.5	7.21	2600000			2.65
O08599	Syntaxin-binding protein 1 OS=Mus musculus GN=Stxbp1 PE=1 SV=2		53.198653	31	184	31	67.526	6.96	200000000	94000000	180000000	584.11
P04627	Serine/threonine-protein kinase A-Raf OS=Mus musculus GN=Araf PE=1 SV=2		3.6423841	2	3	2	67.538	9.11	1500000	780000		6.64
Q9CZW5	Mitochondrial import receptor subunit TOM70 OS=Mus musculus GN=Tomm70 PE=1 SV=		22.094926	11	36	11	67.547	7.53	20000000	8200000	17000000	100.52
P40142	Transketolase OS=Mus musculus GN=Tkt PE=1 SV=1		27.126806	14	32	14	67.588	7.5	9600000	2800000	6700000	95.36
Q9R069	Basal cell adhesion molecule OS=Mus musculus GN=Bcam PE=1 SV=1		4.5016077	2	3	2	67.628	6.25	2100000		1000000	9.21
Q02956	Protein kinase C zeta type OS=Mus musculus GN=Prkcz PE=1 SV=2		2.3648649	1	1	1	67.639	5.76			250000	2.71
P26041	Moesin OS=Mus musculus GN=Msn PE=1 SV=3		11.785095	7	12	3	67.725	6.6	3900000	630000	1100000	30.66
Q99MN1	Lysine--tRNA ligase OS=Mus musculus GN=Kars PE=1 SV=1		7.5630252	2	2	2	67.796	5.94		930000		6.85
Q8BMF4	Dihydrolipoyllysine-residue acetyltransferase component of pyruvate dehydrogenase com		19.937695	10	31	10	67.899	8.57	35000000	30000000	40000000	87.46
P60670	Nuclear protein localization protein 4 homolog OS=Mus musculus GN=Nploc4 PE=1 SV=3		1.4802632	1	1	1	67.974	6.46			970000	2.15
Q9WU79	Proline dehydrogenase 1, mitochondrial OS=Mus musculus GN=Prodh PE=1 SV=2		2.0033389	1	2	1	67.993	8.24		530000	1100000	4.61
Q921G7	Electron transfer flavoprotein-ubiquinone oxidoreductase, mitochondrial OS=Mus musculu		13.798701	8	19	8	68.048	7.58	5400000	3200000	5300000	50.60
Q8BL65	Actin-binding LIM protein 2 OS=Mus musculus GN=Ablim2 PE=1 SV=1		16.666667	8	16	8	68.063	8.02	5300000	930000	4400000	49.26
P15636	SWISS-PROT:P15636 Protease I precursor Lysyl endopeptidase Achromob	x	21.592649	11	278	11	68.083	7.24	4.3E+09	2E+09	4.5E+09	1080.03
Q62074	Protein kinase C iota type OS=Mus musculus GN=Prkci PE=1 SV=3		2.6890756	1	1	1	68.159	5.85	1200000			2.29
P50516	V-type proton ATPase catalytic subunit A OS=Mus musculus GN=Atp6v1a PE=1 SV=2		40.356564	22	86	22	68.283	5.58	49000000	28000000	50000000	242.98
O88487	Cytoplasmic dynein 1 intermediate chain 2 OS=Mus musculus GN=Dync1i2 PE=1 SV=1		11.111111	4	14	4	68.352	5.29	9200000	4800000	7400000	53.40
Q52KF3	Protein spire homolog 1 OS=Mus musculus GN=Spire1 PE=1 SV=1		5.0167224	2	3	2	68.407	9.01	1200000		1300000	9.72
P35235	Tyrosine-protein phosphatase non-receptor type 11 OS=Mus musculus GN=Ptpn11 PE=1		8.3752094	4	6	4	68.417	7.3	710000	300000	1100000	14.54
Q99KN9	Clathrin interactor 1 OS=Mus musculus GN=Clint1 PE=1 SV=2		4.9128368	2	4	2	68.469	6.25	3900000		2600000	12.70
Q91YQ5	Dolichyl-diphosphooligosaccharide--protein glycosyltransferase subunit 1 OS=Mus muscu		19.407895	10	23	10	68.486	6.46	9400000	4100000	6300000	60.73
Q91WJ8	Far upstream element-binding protein 1 OS=Mus musculus GN=Fubp1 PE=1 SV=1		3.0721966	1	2	1	68.497	7.93		250000		5.97
P07724	Serum albumin OS=Mus musculus GN=Alb PE=1 SV=3		26.973684	12	37	12	68.648	6.07	7500000	7100000	8400000	99.87
Q8JZN5	Acyl-CoA dehydrogenase family member 9, mitochondrial OS=Mus musculus GN=Acad9		11.68	7	14	7	68.679	7.46	5500000	2300000	2500000	33.01
P36916	Guanine nucleotide-binding protein-like 1 OS=Mus musculus GN=Gnl1 PE=1 SV=4		4.9423394	3	4	3	68.729	5.68	2000000		1500000	8.75
Q8BGD9	Eukaryotic translation initiation factor 4B OS=Mus musculus GN=Eif4b PE=1 SV=1		8.1833061	5	9	5	68.799	5.67	4500000	820000	5500000	27.42
P35922	Synaptic functional regulator FMR1 OS=Mus musculus GN=Fmr1 PE=1 SV=1		7.8175896	4	8	3	68.947	7.62	2300000	540000	1600000	22.51
P24527	Leukotriene A-4 hydrolase OS=Mus musculus GN=Lta4h PE=1 SV=4		2.1276596	1	1	1	69.007	6.42	950000			1.81
Q3UHK1	Proton myo-inositol cotransporter OS=Mus musculus GN=Slc2a13 PE=1 SV=2		6.9073783	3	6	3	69.018	6.86	3900000	1700000	600000	17.84
Q9DBG6	Dolichyl-diphosphooligosaccharide--protein glycosyltransferase subunit 2 OS=Mus muscu		7.1315372	3	5	3	69.02	5.81	4400000	2900000	3600000	17.05
Q8JZN7	Mitochondrial Rho GTPase 2 OS=Mus musculus GN=Rhot2 PE=1 SV=1		4.0322581	2	3	1	69.027	6	1500000			6.59
Q9D1T0	Leucine-rich repeat and immunoglobulin-like domain-containing nogo receptor-interacting		14.820847	7	17	7	69.057	8.31	4500000	2000000	3000000	53.28
Q9JHS4	ATP-dependent Clp protease ATP-binding subunit clpX-like, mitochondrial OS=Mus musc		2.0504732	1	1	1	69.186	7.71			760000	2.11
P20917	Myelin-associated glycoprotein OS=Mus musculus GN=Mag PE=1 SV=2		7.827476	4	10	4	69.216	5.1	3400000	4000000	6100000	27.14
Q61656	Probable ATP-dependent RNA helicase DDX5 OS=Mus musculus GN=Ddx5 PE=1 SV=2		30.29316	20	40	14	69.247	8.92	17000000	1600000	10000000	100.98
P02769	SWISS-PROT:P02769 (Bos taurus) Bovine serum albumin precursor GN=A	x	7.7429984	4	4	4	69.248	6.18		410000	2900000	8.97
P02768-1	SWISS-PROT:P02768-1 Tax_Id=9606 Gene_Symbol=ALB Isoform 1 of Ser	x	9.3596059	5	9	5	69.321	6.28	2500000	1200000	1000000	20.55
P26040	Ezrin OS=Mus musculus GN=Ezr PE=1 SV=3		10.068259	6	17	1	69.364	6.1	4400000	2000000	4400000	45.63
Q8C2Q3	RNA-binding protein 14 OS=Mus musculus GN=Rbm14 PE=1 SV=1		4.3348281	3	5	3	69.406	9.67	1800000		1000000	11.14
Q8R361	Rab11 family-interacting protein 5 OS=Mus musculus GN=Rab11fip5 PE=1 SV=2		6.3565891	3	9	3	69.51	9.07	1500000	52000	890000	26.27
Q7TMK9	Heterogeneous nuclear ribonucleoprotein Q OS=Mus musculus GN=Syncrip PE=1 SV=2		13.001605	7	16	7	69.59	8.59	10000000	2100000	5500000	34.65
P17156	Heat shock-related 70 kDa protein 2 OS=Mus musculus GN=Hspa2 PE=1 SV=2		33.333333	20	75	8	69.599	5.67	6900000	4400000	4900000	228.59
Q923D5	WW domain-binding protein 11 OS=Mus musculus GN=Wbp11 PE=1 SV=2		2.6521061	1	2	1	69.831	8.4	1600000		410000	4.92
Q8K012	Formin-binding protein 1-like OS=Mus musculus GN=Fnbp1l PE=1 SV=2		6.2809917	3	4	3	69.842	6.64	1800000		4100000	10.45
Q8CCJ4	APC membrane recruitment protein 2 OS=Mus musculus GN=Amer2 PE=1 SV=2		8.1845238	2	2	2	69.89	6.64	1200000		510000	6.72

P31650	Sodium- and chloride-dependent GABA transporter 3 OS=Mus musculus GN=Slc6a11 PE	8.6124402	5	16	5	69.914	6.98	20000000	8700000	16000000	50.21
Q8BZR9	Nuclear cap-binding protein subunit 3 OS=Mus musculus GN=Ncbp3 PE=1 SV=1	2.1138211	1	2	1	70	5.8	460000			6.58
Q9EPR4	Solute carrier family 23 member 2 OS=Mus musculus GN=Slc23a2 PE=1 SV=2	2.6234568	1	3	1	70.003	7.5	1600000	390000	830000	10.22
P17879	Heat shock 70 kDa protein 1B OS=Mus musculus GN=Hspa1b PE=1 SV=3	19.626168	11	33	6	70.133	5.72	5400000	3300000	5200000	100.98
Q99JP7	Gamma-glutamyltransferase 7 OS=Mus musculus GN=Ggt7 PE=1 SV=2	7.7039275	3	6	3	70.208	5.06	3800000	1800000	4200000	19.78
Q8C8N2	Protein SCAI OS=Mus musculus GN=Scai PE=1 SV=2	4.950495	2	3	2	70.231	8.6	6000000	1500000	2400000	8.93
Q8CAA7	Glucose 1,6-bisphosphate synthase OS=Mus musculus GN=Pgm2l1 PE=1 SV=1	5.6360709	3	5	3	70.234	6.49	1100000		1100000	9.68
Q8R555	Cartilage acidic protein 1 OS=Mus musculus GN=Crtac1 PE=2 SV=1	3.250774	1	2	1	70.28	5.14	2900000		1100000	8.21
Q14B80	Potassium voltage-gated channel subfamily C member 2 OS=Mus musculus GN=Kcnc2 F	2.3364486	1	2	1	70.458	7.69	1100000		930000	5.15
P59016	Vacuolar protein sorting-associated protein 33B OS=Mus musculus GN=Vps33b PE=1 SV	3.0794165	1	2	1	70.481	6.86	1700000		980000	6.77
Q8BH04	Phosphoenolpyruvate carboxykinase [GTP], mitochondrial OS=Mus musculus GN=Pck2 F	1.875	1	2	1	70.482	7.28	1200000		920000	6.15
Q9Z1F9	SUMO-activating enzyme subunit 2 OS=Mus musculus GN=Uba2 PE=1 SV=1	4.8589342	2	2	2	70.525	5.24	1700000			7.51
Q8BMA6	Signal recognition particle subunit SRP68 OS=Mus musculus GN=Srp68 PE=1 SV=2	7.36	3	4	3	70.53	8.57	1700000		890000	9.36
Q9Z0V2	Potassium voltage-gated channel subfamily D member 2 OS=Mus musculus GN=Kcnd2 F	8.2539683	3	6	3	70.531	8.07	1800000		1100000	18.87
O54865	Guanylate cyclase soluble subunit beta-1 OS=Mus musculus GN=Gucy1b3 PE=1 SV=1	15.967742	8	14	8	70.553	5.31	5300000	940000	3500000	36.77
P29341	Polyadenylate-binding protein 1 OS=Mus musculus GN=Pabpc1 PE=1 SV=2	11.006289	6	12	6	70.626	9.5	5900000	1200000	4000000	31.55
O88485	Cytoplasmic dynein 1 intermediate chain 1 OS=Mus musculus GN=Dync1i1 PE=1 SV=2	13.375796	7	18	7	70.681	5.12	4000000	3800000	5100000	57.70
Q7TNG5	Echinoderm microtubule-associated protein-like 2 OS=Mus musculus GN=Eml2 PE=1 SV	1.540832	1	1	1	70.689	6.28	2100000			1.99
Q99K51	Plastin-3 OS=Mus musculus GN=Pls3 PE=1 SV=3	4.1269841	1	2	1	70.697	5.62	950000	350000		5.08
P47934	Carnitine O-acetyltransferase OS=Mus musculus GN=Crat PE=1 SV=3	2.3961661	1	3	1	70.794	8.44	2500000	1200000	2100000	11.36
P63017	Heat shock cognate 71 kDa protein OS=Mus musculus GN=Hspa8 PE=1 SV=1	47.678019	32	168	21	70.827	5.52	140000000	86000000	150000000	540.48
P50544	Very long-chain specific acyl-CoA dehydrogenase, mitochondrial OS=Mus musculus GN=	8.0792683	4	6	4	70.831	8.75	1100000	720000	1300000	16.65
P69566	Ran-binding protein 9 OS=Mus musculus GN=Ranbp9 PE=1 SV=1	3.9816233	2	2	2	70.967	6.84	790000		1600000	4.91
Q6ZPU9	KIF1-binding protein OS=Mus musculus GN=Kif1bp PE=1 SV=2	4.8622366	1	1	1	71.007	5.48				3.90
Q6PGE7	Sodium-dependent proline transporter OS=Mus musculus GN=Slc6a7 PE=1 SV=1	3.7676609	2	3	2	71.019	6.61	2600000		2200000	6.40
Q91XI1	tRNA-dihydrouridine(47) synthase [NAD(P)(+)]-like OS=Mus musculus GN=Dus3l PE=1 S	2.9827316	1	1	1	71.033	7.97			200000	2.78
Q9JJY3	Sphingomyelin phosphodiesterase 3 OS=Mus musculus GN=Smpd3 PE=1 SV=1	3.3587786	1	3	1	71.152	5.88	3800000	1500000	3600000	13.71
Q60714	Long-chain fatty acid transport protein 1 OS=Mus musculus GN=Slc27a1 PE=1 SV=1	1.8575851	1	1	1	71.231	8.38	1100000			2.97
Q9R0L7	A-kinase anchor protein 8-like OS=Mus musculus GN=Akap8l PE=1 SV=1	4.0498442	2	4	2	71.408	5.05	3100000		820000	11.53
Q8R349	Cell division cycle protein 16 homolog OS=Mus musculus GN=Cdc16 PE=1 SV=1	2.5806452	1	1	1	71.414	5.76			1000000	2.96
Q91YM4	Protein TBRG4 OS=Mus musculus GN=Tbrg4 PE=1 SV=1	1.2698413	1	1	1	71.468	8.34		260000		1.95
Q7M6Y3	Phosphatidylinositol-binding clathrin assembly protein OS=Mus musculus GN=Picalm PE=	3.3333333	2	5	1	71.498	7.91	1400000	1000000		16.73
Q60722	Transcription factor 4 OS=Mus musculus GN=Tcf4 PE=1 SV=1	2.3880597	1	2	1	71.58	7.17	350000		390000	5.44
Q99LE6	ATP-binding cassette sub-family F member 2 OS=Mus musculus GN=Abcf2 PE=1 SV=1	1.5923567	1	2	1	71.736	7.05	2800000		1400000	4.42
Q99PD7	Sodium/potassium/calcium exchanger 3 OS=Mus musculus GN=Slc24a3 PE=1 SV=3	4.1860465	2	3	2	71.895	5.07	210000		160000	7.93
P08003	Protein disulfide-isomerase A4 OS=Mus musculus GN=Pdia4 PE=1 SV=3	9.4043887	4	10	4	71.938	5.31	2600000	860000	2500000	27.32
Q8CH72	E3 ubiquitin-protein ligase TRIM32 OS=Mus musculus GN=Trim32 PE=1 SV=2	1.6793893	1	1	1	72.011	6.9			2300000	3.22
Q61550	Double-strand-break repair protein rad21 homolog OS=Mus musculus GN=Rad21 PE=1 S	8.1889764	3	4	3	72.038	4.64	1100000		1000000	12.68
Q8BG51	Mitochondrial Rho GTPase 1 OS=Mus musculus GN=Rhot1 PE=1 SV=1	9.5087163	4	7	3	72.196	6.49	4500000	1400000	1600000	19.75
P70663	SPARC-like protein 1 OS=Mus musculus GN=Sparcl1 PE=1 SV=3	22.153846	11	32	11	72.243	4.6	3100000	2000000	4400000	106.45
O08582	GTP-binding protein 1 OS=Mus musculus GN=Gtpbp1 PE=1 SV=2	2.0958084	1	1	1	72.255	8.29	950000			2.64
Q91VE0	Long-chain fatty acid transport protein 4 OS=Mus musculus GN=Slc27a4 PE=1 SV=1	6.8429238	4	4	4	72.272	8.59	2300000	410000	2600000	10.47
Q8BRF7	Sec1 family domain-containing protein 1 OS=Mus musculus GN=Scfd1 PE=1 SV=1	2.6604069	1	3	1	72.277	6.38	2400000	1100000	1700000	10.86
Q501J6	Probable ATP-dependent RNA helicase DDX17 OS=Mus musculus GN=Ddx17 PE=1 SV=	20.615385	13	25	7	72.354	8.59	8600000		5000000	71.54
O70161	Phosphatidylinositol 4-phosphate 5-kinase type-1 gamma OS=Mus musculus GN=Pip5k1	12.556732	6	12	6	72.363	5.59	3900000	1000000	3600000	32.22
P20029	78 kDa glucose-regulated protein OS=Mus musculus GN=Hspa5 PE=1 SV=3	38.931298	21	71	19	72.377	5.16	32000000	24000000	30000000	231.21
P97494	Glutamate--cysteine ligase catalytic subunit OS=Mus musculus GN=Gclc PE=1 SV=4	4.5525903	2	4	2	72.525	5.9	970000	420000	650000	10.55
Q8K2B3	Succinate dehydrogenase [ubiquinone] flavoprotein subunit, mitochondrial OS=Mus musc	17.46988	10	33	10	72.539	7.37	27000000	15000000	32000000	104.09

Q8BG40	Katanin p80 WD40 repeat-containing subunit B1 OS=Mus musculus GN=Katnb1 PE=1 SV	4.8632219	2	3	2	72.593	7.27	960000		940000	9.92
Q8R1X6	Spartin OS=Mus musculus GN=Spg20 PE=1 SV=1	1.9374069	1	1	1	72.61	5.86				2.93
Q03157	Amyloid-like protein 1 OS=Mus musculus GN=Aplp1 PE=1 SV=1	1.8376723	1	2	1	72.705	5.67	1500000	880000		7.98
O70551	SRSF protein kinase 1 OS=Mus musculus GN=Srpk1 PE=1 SV=2	4.3209877	2	3	1	73.043	6.19			410000	7.59
Q62167	ATP-dependent RNA helicase DDX3X OS=Mus musculus GN=Ddx3x PE=1 SV=3	13.444109	8	16	7	73.056	7.18	3100000		3300000	43.56
Q9R049	E3 ubiquitin-protein ligase AMFR OS=Mus musculus GN=Amfr PE=1 SV=2	2.6438569	1	1	1	73.058	6.46		350000		1.89
Q8BVL9	Janus kinase and microtubule-interacting protein 1 OS=Mus musculus GN=Jakmip1 PE=1	2.5559105	2	3	2	73.094	6.09	1700000		1700000	7.38
Q5XG69	Soluble lamin-associated protein of 75 kDa OS=Mus musculus GN=Fam169a PE=1 SV=3	14.736842	6	18	6	73.195	4.68	7400000	850000	6300000	63.22
A2ALS5	Rap1 GTPase-activating protein 1 OS=Mus musculus GN=Rap1gap PE=1 SV=2	5.8823529	3	7	3	73.387	5.87	3500000	950000	3600000	22.07
Q9Z0V1	Potassium voltage-gated channel subfamily D member 3 OS=Mus musculus GN=Kcnd3 F	1.3740458	1	1	1	73.415	8.25			1400000	2.10
P38647	Stress-70 protein, mitochondrial OS=Mus musculus GN=Hspa9 PE=1 SV=3	34.756996	21	56	21	73.416	6.07	15000000	11000000	15000000	185.97
Q3TRR0	Microtubule-associated protein 9 OS=Mus musculus GN=Map9 PE=1 SV=2	9.1331269	3	4	3	73.466	7.62	600000		96000	9.96
O88845	A-kinase anchor protein 10, mitochondrial OS=Mus musculus GN=Akap10 PE=1 SV=3	5.7401813	2	2	2	73.586	6.79	450000		290000	5.33
Q9WVR4	Fragile X mental retardation syndrome-related protein 2 OS=Mus musculus GN=Fxr2 PE=	8.4695394	4	9	3	73.698	6.23	1400000		1400000	25.98
Q8K3G9	DCC-interacting protein 13-beta OS=Mus musculus GN=Appl2 PE=1 SV=1	2.1148036	1	1	1	73.807	5.03			730000	3.04
Q64336	T-box brain protein 1 OS=Mus musculus GN=Tbr1 PE=1 SV=2	4.9926579	2	2	2	73.894	7.33	810000			5.51
D3Z7P3	Glutaminase kidney isoform, mitochondrial OS=Mus musculus GN=Gls PE=1 SV=1	26.261128	12	43	12	73.916	7.99	22000000	12000000	23000000	129.70
O88935	Synapsin-1 OS=Mus musculus GN=Syn1 PE=1 SV=2	52.691218	25	117	23	74.052	9.8	56000000	32000000	43000000	411.40
Q66L44	Voltage-dependent calcium channel beta subunit-associated regulatory protein OS=Mus r	1.8624642	1	2	1	74.086	6.33		450000	390000	5.66
Q9DBC0	Selenoprotein O OS=Mus musculus GN=Selo PE=1 SV=4	1.9490255	1	1	1	74.175	5.83				2.36
P48678	Prelamin-A/C OS=Mus musculus GN=Lmna PE=1 SV=2	42.105263	27	68	26	74.193	6.98	23000000	5500000	19000000	201.56
Q3UMU9	Hepatoma-derived growth factor-related protein 2 OS=Mus musculus GN=Hdgfrp2 PE=1	4.9327354	3	3	3	74.246	8.66	71000		2300000	6.88
Q3UHH2	Solute carrier family 22 member 23 OS=Mus musculus GN=Slc22a23 PE=2 SV=1	1.7416546	1	2	1	74.276	7.83	1000000	350000		4.59
Q7TS72	Inositol-trisphosphate 3-kinase C OS=Mus musculus GN=Itpkc PE=1 SV=1	1.0324484	1	1	1	74.446	5.36	900000			1.89
Q8BH59	Calcium-binding mitochondrial carrier protein Aralar1 OS=Mus musculus GN=Slc25a12 P	32.644018	20	61	20	74.523	8.25	35000000	19000000	24000000	174.83
Q8BH24	Transmembrane 9 superfamily member 4 OS=Mus musculus GN=Tm9sf4 PE=1 SV=1	6.6874028	3	4	3	74.644	7.23	880000	360000		10.01
Q8R480	Nuclear pore complex protein Nup85 OS=Mus musculus GN=Nup85 PE=1 SV=1	1.5243902	1	1	1	74.728	5.57				1.80
Q8K0U4	Heat shock 70 kDa protein 12A OS=Mus musculus GN=Hspa12a PE=1 SV=1	31.259259	18	53	18	74.825	6.77	21000000	8500000	17000000	160.80
Q7TQF7	Amphiphysin OS=Mus musculus GN=Amph PE=1 SV=1	34.402332	18	68	17	74.967	4.63	50000000	31000000	57000000	235.11
Q62172	RalA-binding protein 1 OS=Mus musculus GN=Ralbp1 PE=1 SV=4	5.2469136	3	5	3	74.997	5.92	710000		620000	13.34
Q61033	Lamina-associated polypeptide 2, isoforms alpha/zeta OS=Mus musculus GN=Tmpo PE=	4.6176046	2	4	2	75.122	8.05	9000000	1000000	3300000	13.50
Q8VBZ3	Cleft lip and palate transmembrane protein 1 homolog OS=Mus musculus GN=Clptm1 PE	14.608434	5	11	5	75.243	6.3	1300000	700000	900000	32.65
Q9Z2C4	Myotubularin-related protein 1 OS=Mus musculus GN=Mtmr1 PE=1 SV=1	4.9327354	3	4	3	75.266	6.8	1800000		960000	10.16
Q8VIJ6	Splicing factor, proline- and glutamine-rich OS=Mus musculus GN=Sfpq PE=1 SV=1	14.735336	9	35	9	75.394	9.44	21000000	6000000	14000000	99.39
P55096	ATP-binding cassette sub-family D member 3 OS=Mus musculus GN=Abcd3 PE=1 SV=2	2.124431	1	1	1	75.426	9.26	2800000			2.61
P47708	Rabphilin-3A OS=Mus musculus GN=Rph3a PE=1 SV=2	25.403818	12	35	12	75.442	8.27	7800000	3300000	5500000	112.89
Q9Z2C9	Myotubularin-related protein 7 OS=Mus musculus GN=Mtmr7 PE=1 SV=2	2.4242424	1	1	1	75.56	6.43	590000			4.51
Q9D0I9	Arginine--tRNA ligase, cytoplasmic OS=Mus musculus GN=Rars PE=1 SV=2	2.4242424	1	1	1	75.625	7.55	1900000			2.76
P14824	Annexin A6 OS=Mus musculus GN=Anxa6 PE=1 SV=3	29.123328	15	35	15	75.837	5.5	5300000	2500000	4400000	98.37
Q8BH57	WD repeat-containing protein 48 OS=Mus musculus GN=Wdr48 PE=1 SV=1	9.9112426	5	11	5	75.959	7.17	2300000	810000	2400000	30.95
Q8K2C7	Protein OS-9 OS=Mus musculus GN=Os9 PE=1 SV=2	3.7202381	2	3	2	76.061	4.84	650000	910000	510000	9.51
Q61584	Fragile X mental retardation syndrome-related protein 1 OS=Mus musculus GN=Fxr1 PE=	6.7946824	3	5	2	76.175	6.98	1700000		2100000	13.40
P10637	Microtubule-associated protein tau OS=Mus musculus GN=Mapt PE=1 SV=3	19.099591	9	25	9	76.197	6.79	14000000	5400000	10000000	68.75
Q80X80	C2 domain-containing protein 2-like OS=Mus musculus GN=C2cd2l PE=1 SV=3	3.9660057	2	5	2	76.282	7.21	8700000		9600000	17.52
P28571	Sodium- and chloride-dependent glycine transporter 1 OS=Mus musculus GN=Slc6a9 PE	3.1791908	1	3	1	76.494	7.81	2800000	1100000	1500000	6.38
Q8C0L9	Glycerophosphocholine phosphodiesterase GPCPD1 OS=Mus musculus GN=Gpcpd1 PE	2.3703704	1	1	1	76.531	5.58			480000	2.31
Q921I1	Serotransferrin OS=Mus musculus GN=Tf PE=1 SV=1	16.212339	9	16	9	76.674	7.18	960000	1600000	1300000	49.85
Q3THK7	GMP synthase [glutamine-hydrolyzing] OS=Mus musculus GN=Gmps PE=1 SV=2	3.1746032	2	4	2	76.675	6.73	2500000	980000	2100000	10.92

P09405	Nucleolin OS=Mus musculus GN=Ncl PE=1 SV=2	22.065064	14	30	14	76.677	4.75	23000000	1100000	19000000	85.78
P68404	Protein kinase C beta type OS=Mus musculus GN=Prkcb PE=1 SV=4	18.628912	10	31	6	76.701	7.01	22000000	6900000	18000000	91.28
O54781	SRSF protein kinase 2 OS=Mus musculus GN=Srpk2 PE=1 SV=2	6.4610866	4	10	3	76.709	4.91	1300000	500000	2600000	28.43
Q3U0V1	Far upstream element-binding protein 2 OS=Mus musculus GN=Khsrp PE=1 SV=2	4.144385	2	2	2	76.728	7.33	4400000	200000		5.22
Q6P9S0	MTSS1-like protein OS=Mus musculus GN=Mtss1l PE=1 SV=1	2.9370629	1	1	1	76.797	6.99	920000			3.56
P20444	Protein kinase C alpha type OS=Mus musculus GN=Prkca PE=1 SV=3	12.202381	7	21	2	76.802	7.14	3800000		860000	61.48
P37040	NADPH--cytochrome P450 reductase OS=Mus musculus GN=Por PE=1 SV=2	11.20944	6	12	6	76.995	5.53	2600000	1400000	2500000	31.65
Q8CGU1	Calcium-binding and coiled-coil domain-containing protein 1 OS=Mus musculus GN=Calc	3.617945	2	4	2	77.232	4.82	1000000		1200000	11.75
Q9QXS6	Drebrin OS=Mus musculus GN=Dbn1 PE=1 SV=4	35.127479	16	65	16	77.239	4.49	45000000	19000000	51000000	236.91
Q8BG39	Synaptic vesicle glycoprotein 2B OS=Mus musculus GN=Sv2b PE=1 SV=1	15.22694	9	39	9	77.406	5.57	58000000	17000000	33000000	119.68
Q922U1	U4/U6 small nuclear ribonucleoprotein Prp3 OS=Mus musculus GN=Prpf3 PE=1 SV=1	1.6105417	1	1	1	77.407	9.5	1600000			2.20
Q9QXK7	Cleavage and polyadenylation specificity factor subunit 3 OS=Mus musculus GN=Cpsf3 P	2.1929825	1	1	1	77.455	5.6	190000			2.76
P70302	Stromal interaction molecule 1 OS=Mus musculus GN=Stim1 PE=1 SV=2	2.919708	1	1	1	77.518	6.54			1800000	3.96
Q9D0E1	Heterogeneous nuclear ribonucleoprotein M OS=Mus musculus GN=Hnrnpm PE=1 SV=3	15.775034	9	21	9	77.597	8.63	8800000	2100000	8200000	62.36
Q6PDJ6	F-box only protein 42 OS=Mus musculus GN=Fbxo42 PE=1 SV=1	1.9525802	1	1	1	77.728	7.58			88000	2.90
Q8CIB5	Fermitin family homolog 2 OS=Mus musculus GN=Fermt2 PE=1 SV=1	4.7058824	3	6	3	77.751	6.7	2700000	530000	1500000	16.38
P41216	Long-chain-fatty-acid--CoA ligase 1 OS=Mus musculus GN=Acsl1 PE=1 SV=2	3.8626609	3	4	3	77.901	7.15	2300000		2300000	9.10
Q80U04	E3 ubiquitin-protein ligase Praja-2 OS=Mus musculus GN=Pja2 PE=1 SV=2	3.2531825	1	2	1	77.91	4.44	1000000		630000	8.58
P16014	Secretogranin-1 OS=Mus musculus GN=Chgb PE=1 SV=2	2.2156573	1	3	1	77.922	5.07	1300000	1600000	2200000	9.30
Q9CXJ4	ATP-binding cassette sub-family B member 8, mitochondrial OS=Mus musculus GN=Abcb	3.0683403	2	2	2	77.95	9.07	1300000		2500000	4.80
Q91WC3	Long-chain-fatty-acid--CoA ligase 6 OS=Mus musculus GN=Acsl6 PE=1 SV=1	15.064562	9	22	9	77.967	7.34	9900000	2400000	6700000	67.37
Q08879	Fibulin-1 OS=Mus musculus GN=Fbln1 PE=1 SV=2	2.4113475	1	1	1	77.981	5.16		170000		2.85
Q60865	Caprin-1 OS=Mus musculus GN=Caprin1 PE=1 SV=2	10.749646	4	13	4	78.121	5.25	8000000	3200000	6300000	40.53
Q8R550	SH3 domain-containing kinase-binding protein 1 OS=Mus musculus GN=Sh3kbp1 PE=1 S	6.629055	3	5	3	78.122	7.55	20000000		930000	15.65
Q9WTR5	Cadherin-13 OS=Mus musculus GN=Cdh13 PE=1 SV=2	7.9831933	4	12	4	78.137	5.12	4300000	4100000	4500000	38.68
Q5SVL6	Rap1 GTPase-activating protein 2 OS=Mus musculus GN=Rap1gap2 PE=1 SV=1	3.9325843	2	3	2	78.205	6.43	4300000	810000	3300000	8.93
Q5K6N0	Transmembrane protein 232 OS=Mus musculus GN=Tmem232 PE=2 SV=2	2.5185185	1	1	1	78.225	7.28	5600000			0.00
P63318	Protein kinase C gamma type OS=Mus musculus GN=Prkcg PE=1 SV=1	27.259684	14	36	13	78.307	7.46	29000000	9000000	19000000	109.03
P47856	Glutamine--fructose-6-phosphate aminotransferase [isomerizing] 1 OS=Mus musculus GN	2.1520803	1	1	1	78.489	6.84			430000	2.20
Q9ESJ4	NCK-interacting protein with SH3 domain OS=Mus musculus GN=Nckipsd PE=1 SV=2	12.044818	6	13	6	78.523	6.05	2400000	530000	2200000	37.24
Q9QYB5	Gamma-adducin OS=Mus musculus GN=Add3 PE=1 SV=2	17.280453	10	24	10	78.728	5.95	6700000	2300000	6500000	69.40
Q9Z0E0	Neurochondrin OS=Mus musculus GN=Ncdn PE=1 SV=1	16.59808	11	22	11	78.845	5.54	16000000	3300000	11000000	57.94
Q9QUJ7	Long-chain-fatty-acid--CoA ligase 4 OS=Mus musculus GN=Acsl4 PE=1 SV=2	2.1097046	1	1	1	79.026	8.28	520000			2.17
Q8K3H0	DCC-interacting protein 13-alpha OS=Mus musculus GN=Appl1 PE=1 SV=1	4.950495	3	7	3	79.278	5.41	3100000	660000	3300000	17.42
Q99MR8	Methylcrotonoyl-CoA carboxylase subunit alpha, mitochondrial OS=Mus musculus GN=Mc	10.739191	6	17	6	79.293	7.83	6500000	3700000	1800000	38.50
D3YVF0	A-kinase anchor protein 5 OS=Mus musculus GN=Akap5 PE=1 SV=2	47.516779	16	46	16	79.35	4.75	32000000	14000000	40000000	182.49
P51660	Peroxisomal multifunctional enzyme type 2 OS=Mus musculus GN=Hsd17b4 PE=1 SV=3	7.2108844	4	5	4	79.432	8.57	2200000		1100000	14.29
Q80Y98	Phospholipase DDHD2 OS=Mus musculus GN=Ddhd2 PE=1 SV=3	2.0028612	1	4	1	79.527	5.31	1500000	660000	1200000	11.39
P59823	Interleukin-1 receptor accessory protein-like 1 OS=Mus musculus GN=Il1rapl1 PE=1 SV=	4.6043165	2	5	2	79.579	6.32	4200000	710000	2700000	14.20
Q99MK8	Beta-adrenergic receptor kinase 1 OS=Mus musculus GN=Adrbk1 PE=1 SV=2	5.8055152	3	5	3	79.588	7.28	3200000	1500000	3300000	14.94
P28740	Kinesin-like protein KIF2A OS=Mus musculus GN=Kif2a PE=1 SV=2	9.5035461	6	17	6	79.707	6.73	6900000	1700000	4300000	44.32
Q91VD9	NADH-ubiquinone oxidoreductase 75 kDa subunit, mitochondrial OS=Mus musculus GN=	38.376891	24	78	24	79.726	5.72	32000000	15000000	28000000	231.57
Q5U3K5	Rab-like protein 6 OS=Mus musculus GN=Rabl6 PE=1 SV=2	8.6896552	5	10	5	79.782	5.53	4300000	640000	2300000	29.81
Q3UHB8	Coiled-coil domain-containing protein 177 OS=Mus musculus GN=Ccdc177 PE=1 SV=1	3.5410765	2	4	2	79.809	10.8	3800000	610000	2300000	13.75
O08529	Calpain-2 catalytic subunit OS=Mus musculus GN=Capn2 PE=1 SV=4	12.857143	8	19	8	79.822	4.96	3800000	1200000	4100000	55.08
Q91ZA3	Propionyl-CoA carboxylase alpha chain, mitochondrial OS=Mus musculus GN=Pcca PE=	12.154696	7	17	7	79.871	7.25	4600000	2600000	5100000	49.68
O35250	Exocyst complex component 7 OS=Mus musculus GN=Exoc7 PE=1 SV=2	6.025825	3	6	3	79.911	6.98	1800000	1200000	1400000	16.28
Q7TN98	Cytoplasmic polyadenylation element-binding protein 4 OS=Mus musculus GN=Cpeb4 PE	3.9780521	2	4	2	80.072	7.18	860000		550000	11.08

P28741	Kinesin-like protein KIF3A OS=Mus musculus GN=Kif3a PE=1 SV=2		6.2767475	4	8	4	80.12	6.54	1800000		2000000	18.28
Q8BLV3	Sodium/hydrogen exchanger 7 OS=Mus musculus GN=Slc9a7 PE=1 SV=1		1.5151515	1	1	1	80.238	6.4			230000	2.33
Q99PU5	Long-chain-fatty-acid--CoA ligase ACSBG1 OS=Mus musculus GN=Acsbg1 PE=1 SV=1		15.950069	8	16	8	80.374	5.94	6200000	1800000	4600000	42.54
Q06335	Amyloid-like protein 2 OS=Mus musculus GN=Aplp2 PE=1 SV=4		5.9405941	3	6	3	80.416	4.7	1400000	1100000	1800000	18.37
Q62108	Disks large homolog 4 OS=Mus musculus GN=Dlg4 PE=1 SV=1		26.79558	18	51	17	80.423	5.87	47000000	6300000	38000000	155.30
Q8BWS5	G protein-regulated inducer of neurite outgrowth 3 OS=Mus musculus GN=Gprin3 PE=1 S		6.9462647	4	5	4	80.435	7.02	800000			12.44
Q9CZW4	Long-chain-fatty-acid--CoA ligase 3 OS=Mus musculus GN=Acsl3 PE=1 SV=2		3.4722222	2	4	2	80.441	8.54	3200000		1500000	10.47
P46978	Dolichyl-diphosphooligosaccharide--protein glycosyltransferase subunit STT3A OS=Mus r		1.7021277	1	3	1	80.545	8.1	3500000	890000	1600000	7.32
Q9QYB8	Beta-adducin OS=Mus musculus GN=Add2 PE=1 SV=4		32.689655	20	58	19	80.591	6.21	27000000	8200000	26000000	176.41
Q9QYC0	Alpha-adducin OS=Mus musculus GN=Add1 PE=1 SV=2		35.510204	20	70	19	80.596	5.9	30000000	9300000	27000000	209.08
Q9R1R2	Tripartite motif-containing protein 3 OS=Mus musculus GN=Trim3 PE=1 SV=1		3.6290323	2	3	2	80.724	7.81	5500000	1300000	1700000	8.35
A2AQ19	RNA polymerase-associated protein RTF1 homolog OS=Mus musculus GN=Rtf1 PE=1 SV		1.5384615	1	1	1	80.748	8.16	1100000			2.24
Q64521	Glycerol-3-phosphate dehydrogenase, mitochondrial OS=Mus musculus GN=Gpd2 PE=1		33.837689	22	62	22	80.902	6.61	19000000	13000000	16000000	183.33
Q6PGF7	Exocyst complex component 8 OS=Mus musculus GN=Exoc8 PE=1 SV=1		4.4692737	2	4	2	80.984	5.4	1400000	590000	1000000	11.86
Q8BJI1	Sodium-dependent neutral amino acid transporter SLC6A17 OS=Mus musculus GN=Slc6		11.141678	5	14	5	81.018	6.23	12000000	1900000	6100000	48.54
Q8C561	LMBR1 domain-containing protein 2 OS=Mus musculus GN=Lmbrd2 PE=1 SV=1		4.3227666	3	3	3	81.049	7.34	1600000			10.33
Q9ET77	Junctophilin-3 OS=Mus musculus GN=Jph3 PE=1 SV=1		1.7473118	1	2	1	81.18	9.22	2100000		1700000	5.50
P29533	Vascular cell adhesion protein 1 OS=Mus musculus GN=Vcam1 PE=1 SV=1		10.14885	6	10	6	81.265	5.3	1400000	1100000	1900000	29.46
Q9ESN6	Tripartite motif-containing protein 2 OS=Mus musculus GN=Trim2 PE=1 SV=1		11.155914	6	12	6	81.394	6.96	5000000	1400000	3000000	34.93
Q02111	Protein kinase C theta type OS=Mus musculus GN=Prkcq PE=1 SV=1		2.5459689	2	4	1	81.519	7.71			2900000	8.99
P58404	Striatin-4 OS=Mus musculus GN=Strn4 PE=1 SV=2		8.5526316	6	16	6	81.595	5.38	3000000	1200000	3800000	44.94
Q69Z98	Serine/threonine-protein kinase BRSK2 OS=Mus musculus GN=Brsk2 PE=1 SV=2		1.9047619	1	3	1	81.682	8.79	4700000	840000	2600000	11.28
Q3TPX4	Exocyst complex component 5 OS=Mus musculus GN=Exoc5 PE=1 SV=2		2.8248588	2	2	2	81.686	6.71		420000	1300000	4.31
Q02257	Junction plakoglobin OS=Mus musculus GN=Jup PE=1 SV=3		6.5771812	4	10	3	81.749	6.14	2100000	500000	2900000	28.48
Q9CZD3	Glycine--tRNA ligase OS=Mus musculus GN=Gars PE=1 SV=1		2.0576132	1	1	1	81.826	6.65			620000	3.35
Q8VDU5	SNF-related serine/threonine-protein kinase OS=Mus musculus GN=Snrk PE=1 SV=1		3.7433155	1	1	1	81.861	7.49	330000			4.34
Q8K0V4	CCR4-NOT transcription complex subunit 3 OS=Mus musculus GN=Cnot3 PE=1 SV=1		1.8641811	1	1	1	81.896	6.2			870000	2.91
Q8C754	Vacuolar protein sorting-associated protein 52 homolog OS=Mus musculus GN=Vps52 PE		4.4260028	3	5	3	81.993	5.9	1400000		740000	12.59
O35350	Calpain-1 catalytic subunit OS=Mus musculus GN=Capn1 PE=1 SV=1		1.6830295	1	2	1	82.054	5.87		1100000	2200000	5.82
Q63959	Potassium voltage-gated channel subfamily C member 3 OS=Mus musculus GN=Kcnc3 F		3.3810143	2	2	2	82.09	7.09			740000	4.63
Q8CHT1	Ephexin-1 OS=Mus musculus GN=Ngef PE=1 SV=1		4.2253521	3	8	3	82.148	5.99	7100000	1800000	5200000	20.17
Q9QYE6	Golgin subfamily A member 5 OS=Mus musculus GN=Golga5 PE=1 SV=2		3.5665295	1	1	1	82.318	6.23	330000			2.80
O35691	Pinin OS=Mus musculus GN=Pnn PE=1 SV=4		9.6551724	4	8	4	82.386	7.01	2500000		2100000	29.17
Q91VR5	ATP-dependent RNA helicase DDX1 OS=Mus musculus GN=Ddx1 PE=1 SV=1		14.054054	8	14	8	82.448	7.21	8700000	1400000	4200000	39.17
Q61102	ATP-binding cassette sub-family B member 7, mitochondrial OS=Mus musculus GN=Abcb		1.5957447	1	2	1	82.529	9.32	3300000		1900000	4.74
P46460	Vesicle-fusing ATPase OS=Mus musculus GN=Nsf PE=1 SV=2		47.177419	34	130	34	82.561	6.95	110000000	61000000	88000000	367.41
Q9JIS5	Synaptic vesicle glycoprotein 2A OS=Mus musculus GN=Sv2a PE=1 SV=1		12.398922	9	47	9	82.594	5.57	63000000	12000000	27000000	143.47
Q8K1M6	Dynamin-1-like protein OS=Mus musculus GN=Dnm1l PE=1 SV=2		21.563342	13	30	13	82.606	7.05	6200000	2300000	4700000	90.64
Q8BMS1	Trifunctional enzyme subunit alpha, mitochondrial OS=Mus musculus GN=Hadha PE=1 S		7.2083879	5	10	5	82.617	9.14	15000000	5400000	4100000	29.68
P98084	Amyloid beta A4 precursor protein-binding family A member 2 OS=Mus musculus GN=Apl		3.4666667	2	3	1	82.706	4.88	2400000		1700000	6.42
P16332	Methylmalonyl-CoA mutase, mitochondrial OS=Mus musculus GN=Mut PE=1 SV=2		5.0802139	3	7	3	82.792	6.89	1900000	820000	1500000	20.43
Q99LI7	Cleavage stimulation factor subunit 3 OS=Mus musculus GN=Cstf3 PE=1 SV=1		1.8131102	1	1	1	82.824	8.12				3.01
Q6PGN3	Serine/threonine-protein kinase DCLK2 OS=Mus musculus GN=Dclk2 PE=1 SV=1		1.984127	1	1	1	82.927	8.44			500000	3.95
Q9Z2I0	LETM1 and EF-hand domain-containing protein 1, mitochondrial OS=Mus musculus GN=l		21.409214	14	42	14	82.937	6.52	15000000	7800000	12000000	124.19
Q8BSY0	Aspartyl/asparaginyl beta-hydroxylase OS=Mus musculus GN=Asph PE=1 SV=1		7.0175439	2	10	2	82.991	5.08	3900000	2100000	3800000	30.22
Q8BH55	Threonine synthase-like 1 OS=Mus musculus GN=Thnsl1 PE=1 SV=1		1.7402945	1	1	1	83.047	7.2			530000	1.87
Q8C167	Prolyl endopeptidase-like OS=Mus musculus GN=Prepl PE=1 SV=1		11.172414	7	15	7	83.141	6.65	4900000	1500000	4400000	46.67
Q8BUK6	Protein Hook homolog 3 OS=Mus musculus GN=Hook3 PE=1 SV=2		7.1030641	4	11	4	83.166	5.19	2200000	1300000	6400000	34.16

P11499	Heat shock protein HSP 90-beta OS=Mus musculus GN=Hsp90ab1 PE=1 SV=3	41.850829	29	117	16	83.229	5.03	77000000	29000000	69000000	351.31
Q8BUV3	Gephyrin OS=Mus musculus GN=Gphn PE=1 SV=2	18.335501	9	19	9	83.23	5.6	7200000	1500000	7800000	58.67
Q7TME0	Phospholipid phosphatase-related protein type 4 OS=Mus musculus GN=Plppr4 PE=1 SV	8.0939948	4	7	4	83.238	8.84	2400000	750000	3500000	17.90
Q9D0R2	Threonine--tRNA ligase, cytoplasmic OS=Mus musculus GN=Tars PE=1 SV=2	6.3711911	3	3	3	83.303	7.36	900000			9.02
P63154	Crooked neck-like protein 1 OS=Mus musculus GN=Crnkl1 PE=1 SV=1	1.7391304	1	1	1	83.363	6.93	450000			2.62
Q80TL0	Protein phosphatase 1E OS=Mus musculus GN=Ppm1e PE=1 SV=2	2.0026702	1	5	1	83.367	4.97	18000000	5200000	18000000	17.17
Q7TMQ7	WD repeat-containing protein 91 OS=Mus musculus GN=Wdr91 PE=1 SV=1	2.1390374	1	1	1	83.368	6.64		1500000		2.55
Q7TNM2	Tripartite motif-containing protein 46 OS=Mus musculus GN=Trim46 PE=1 SV=1	1.9762846	1	2	1	83.378	7.65	1200000		560000	6.84
P16054	Protein kinase C epsilon type OS=Mus musculus GN=Prkce PE=1 SV=1	19.674355	12	37	11	83.507	7.03	8000000	1900000	4700000	104.70
Q8K0T0	Reticulon-1 OS=Mus musculus GN=Rtn1 PE=1 SV=1	20.128205	12	33	11	83.521	4.58	25000000	6000000	19000000	93.23
Q8BHL5	Engulfment and cell motility protein 2 OS=Mus musculus GN=Elmo2 PE=1 SV=1	11.065574	5	7	5	83.833	5.95	1500000	1200000	1500000	21.83
Q8CAQ8	MICOS complex subunit Mic60 OS=Mus musculus GN=Immt PE=1 SV=1	38.441215	25	82	25	83.848	6.61	32000000	22000000	38000000	236.65
Q6Y685	Transforming acidic coiled-coil-containing protein 1 OS=Mus musculus GN=Tacc1 PE=1 S	3.4883721	1	1	1	83.9	5.03				4.15
Q99MD9	Nuclear autoantigenic sperm protein OS=Mus musculus GN=Nasp PE=1 SV=2	1.4230272	1	1	1	83.903	4.37			640000	3.00
Q8BXR1	Probable cationic amino acid transporter OS=Mus musculus GN=Slc7a14 PE=1 SV=1	3.2425422	2	6	2	83.929	5.35	2800000	880000	1700000	13.95
Q3UFS0	Protein zyg-11 homolog B OS=Mus musculus GN=Zyg11b PE=1 SV=2	2.016129	1	1	1	83.937	6.87			310000	2.02
Q9D2N4	Dystrobrevin alpha OS=Mus musculus GN=Dtna PE=1 SV=2	4.155496	3	5	3	84.014	6.76	2600000		2000000	15.06
Q9JIG4	Protein phosphatase 1 regulatory subunit 3F OS=Mus musculus GN=Ppp1r3f PE=1 SV=3	2.252816	1	1	1	84.068	4.7			380000	3.45
Q9R1V4	Disintegrin and metalloproteinase domain-containing protein 11 OS=Mus musculus GN=A	12.936611	7	14	7	84.08	7.5	4800000	3700000	4400000	44.18
Q9JLM8	Serine/threonine-protein kinase DCLK1 OS=Mus musculus GN=Dclk1 PE=1 SV=1	19.57672	10	21	10	84.101	8.87	7600000	1700000	3500000	67.34
Q01063	cAMP-specific 3',5'-cyclic phosphodiesterase 4D OS=Mus musculus GN=Pde4d PE=1 SV	5.4886212	3	3	3	84.509	4.91	1700000		1200000	8.80
Q3UMT1	Protein phosphatase 1 regulatory subunit 12C OS=Mus musculus GN=Ppp1r12c PE=1 SV	1.6624041	1	1	1	84.634	6			530000	2.17
P07901	Heat shock protein HSP 90-alpha OS=Mus musculus GN=Hsp90aa1 PE=1 SV=4	43.24693	33	121	22	84.735	5.01	96000000	35000000	80000000	353.01
Q00PI9	Heterogeneous nuclear ribonucleoprotein U-like protein 2 OS=Mus musculus GN=Hnrnpu	27.785235	17	43	17	84.888	4.89	15000000	1100000	9600000	122.75
Q5RJI5	Serine/threonine-protein kinase BRSK1 OS=Mus musculus GN=Brsk1 PE=1 SV=1	1.7994859	1	2	1	85.102	9.32	1600000		850000	5.89
P47857	ATP-dependent 6-phosphofructokinase, muscle type OS=Mus musculus GN=Pfkm PE=1	12.564103	8	23	7	85.215	8	8700000	2600000	4000000	61.49
Q61771	Kinesin-like protein KIF3B OS=Mus musculus GN=Kif3b PE=1 SV=1	8.2998661	4	8	4	85.236	7.69	2000000	760000	1200000	18.97
Q68FF6	ARF GTPase-activating protein GIT1 OS=Mus musculus GN=Git1 PE=1 SV=1	10.649351	6	11	6	85.247	6.93	3800000	360000	2000000	32.00
Q80TI0	GRAM domain-containing protein 1B OS=Mus musculus GN=Gramd1b PE=1 SV=2	1.4905149	1	1	1	85.301	6.14	2300000			2.34
P12382	ATP-dependent 6-phosphofructokinase, liver type OS=Mus musculus GN=Pfkl PE=1 SV=	5.6410256	4	13	2	85.305	7.17	3400000	4300000	4800000	42.01
Q9WUA3	ATP-dependent 6-phosphofructokinase, platelet type OS=Mus musculus GN=Pfkp PE=1 S	13.010204	9	24	7	85.4	7.11	14000000	3700000	5100000	70.70
Q99KI0	Aconitate hydratase, mitochondrial OS=Mus musculus GN=Aco2 PE=1 SV=1	36.794872	29	95	29	85.41	7.93	87000000	34000000	67000000	300.47
Q02248	Catenin beta-1 OS=Mus musculus GN=Ctnnb1 PE=1 SV=1	15.364917	8	23	7	85.416	5.86	11000000	3500000	6800000	76.86
Q9QYH6	Melanoma-associated antigen D1 OS=Mus musculus GN=Maged1 PE=1 SV=1	1.9354839	1	3	1	85.617	7.5	1400000	690000	760000	10.97
Q62351	Transferrin receptor protein 1 OS=Mus musculus GN=Tfrc PE=1 SV=1	5.7667104	4	8	4	85.677	6.57	1700000	560000	990000	21.56
Q03173	Protein enabled homolog OS=Mus musculus GN=Enah PE=1 SV=2	1.3715711	1	1	1	85.792	7.72			1100000	2.33
Q8R3B1	1-phosphatidylinositol 4,5-bisphosphate phosphodiesterase delta-1 OS=Mus musculus GI	2.6455026	1	1	1	85.819	6.21			1200000	3.41
Q60611	DNA-binding protein SATB1 OS=Mus musculus GN=Satb1 PE=1 SV=2	1.3089005	1	1	1	85.826	6.54	780000			2.70
P13020	Gelsolin OS=Mus musculus GN=Gsn PE=1 SV=3	5	2	2	2	85.888	6.18		2000000	2900000	7.33
Q9WTR1	Transient receptor potential cation channel subfamily V member 2 OS=Mus musculus GN	3.5714286	2	4	2	85.91	6.99	970000		710000	13.26
O55106	Striatin OS=Mus musculus GN=Strn PE=1 SV=2	14.871795	7	15	7	85.913	5.27	2900000	1000000	2700000	47.85
Q99LI8	Hepatocyte growth factor-regulated tyrosine kinase substrate OS=Mus musculus GN=Hgs	5.6774194	4	9	4	85.961	6.16	2400000	1200000	2600000	22.14
A2AG50	MAP7 domain-containing protein 2 OS=Mus musculus GN=Map7d2 PE=1 SV=1	5.7618438	3	4	3	85.997	8.81	780000		650000	13.40
P06537	Glucocorticoid receptor OS=Mus musculus GN=Nr3c1 PE=1 SV=1	1.7879949	1	1	1	85.998	6.35	290000			3.04
Q8VD37	SH3-containing GRB2-like protein 3-interacting protein 1 OS=Mus musculus GN=Sgip1 PI	20.595533	10	26	10	86.01	7.87	11000000	2100000	8200000	73.86
Q01405	Protein transport protein Sec23A OS=Mus musculus GN=Sec23a PE=1 SV=2	1.6993464	1	1	1	86.106	7.08	2200000			1.93
Q80U63	Mitofusin-2 OS=Mus musculus GN=Mfn2 PE=1 SV=3	18.361955	11	18	11	86.133	6.77	5200000	1600000	3000000	52.27
Q8BGC0	HIV Tat-specific factor 1 homolog OS=Mus musculus GN=Htatsf1 PE=1 SV=1	10.964333	4	5	4	86.188	4.4	1300000		350000	15.24

Q8C145	Zinc transporter ZIP6 OS=Mus musculus GN=Slc39a6 PE=1 SV=1	8.2352941	3	3	3	86.326	6.84	410000	680000		4.13
Q6KAR6	Exocyst complex component 3 OS=Mus musculus GN=Exoc3 PE=1 SV=2	1.4569536	1	1	1	86.4	6.2		580000		2.12
Q6NZF1	Zinc finger CCCH domain-containing protein 11A OS=Mus musculus GN=Zc3h11a PE=1	2.020202	1	1	1	86.438	8.13	210000			3.19
O54916	RalBP1-associated Eps domain-containing protein 1 OS=Mus musculus GN=Reps1 PE=1	2.6415094	2	4	1	86.466	5.58	1500000		1100000	9.37
P12023	Amyloid beta A4 protein OS=Mus musculus GN=App PE=1 SV=3	17.012987	9	29	9	86.667	4.79	6200000	5200000	9400000	91.06
Q9D4H8	Cullin-2 OS=Mus musculus GN=Cul2 PE=1 SV=2	2.8187919	2	3	2	86.821	7.01	1400000		1500000	6.93
Q810J8	Zinc finger FYVE domain-containing protein 1 OS=Mus musculus GN=Zfyve1 PE=1 SV=2	3.8610039	2	5	2	86.884	7.34	1200000	230000	970000	13.88
P52479	Ubiquitin carboxyl-terminal hydrolase 10 OS=Mus musculus GN=Usp10 PE=1 SV=3	1.5151515	1	1	1	86.968	5.17	1500000			2.31
Q9ERG2	Striatin-3 OS=Mus musculus GN=Strn3 PE=1 SV=1	15.075377	8	13	8	87.096	5.29	2800000	2800000	4900000	43.51
Q6ZQK5	Arf-GAP with coiled-coil, ANK repeat and PH domain-containing protein 2 OS=Mus muscu	3.7662338	2	3	2	87.156	6.68	210000		570000	8.54
P27612	Phospholipase A-2-activating protein OS=Mus musculus GN=Plaa PE=1 SV=4	5.5415617	3	5	3	87.166	6.14	1500000	420000	790000	16.75
Q8BHL3	TBC1 domain family member 10B OS=Mus musculus GN=Tbc1d10b PE=1 SV=2	0.877193	1	3	1	87.221	8.9	2200000	670000	1900000	6.25
Q8K1N1	Calcium-independent phospholipase A2-gamma OS=Mus musculus GN=Pnpla8 PE=1 SV	1.9329897	1	1	1	87.326	9.23				2.13
A2A699	Protein FAM171A2 OS=Mus musculus GN=Fam171a2 PE=1 SV=1	1.8248175	1	3	1	87.433	8.05	2100000	600000	1500000	8.66
Q8K2L8	Trafficking protein particle complex subunit 12 OS=Mus musculus GN=Trappc12 PE=1 SV	1.5056462	1	1	1	87.639	4.81			680000	2.75
Q8VEK3	Heterogeneous nuclear ribonucleoprotein U OS=Mus musculus GN=Hnrnpu PE=1 SV=1	18.375	11	36	11	87.863	6.24	34000000	2000000	17000000	96.41
P55288	Cadherin-11 OS=Mus musculus GN=Cdh11 PE=1 SV=1	2.0100503	1	1	1	88.058	4.89			830000	2.88
P09055	Integrin beta-1 OS=Mus musculus GN=Itgb1 PE=1 SV=1	5.2631579	4	5	4	88.173	5.94	1800000	1400000	840000	11.15
P70408	Cadherin-10 OS=Mus musculus GN=Cdh10 PE=1 SV=3	3.0456853	2	4	2	88.257	4.94	1600000		1700000	11.04
Q8VHJ5	Serine/threonine-protein kinase MARK1 OS=Mus musculus GN=Mark1 PE=1 SV=2	2.7672956	2	5	2	88.28	9.39	3100000	1100000	2000000	13.64
Q3TDD9	Protein phosphatase 1 regulatory subunit 21 OS=Mus musculus GN=Ppp1r21 PE=1 SV=2	5.1282051	3	5	3	88.282	6.9	1700000	140000	630000	15.28
Q9Z2G6	Protein sel-1 homolog 1 OS=Mus musculus GN=Sel1l PE=1 SV=2	4.9367089	3	9	3	88.285	5.57	2800000	2500000	4100000	28.13
O35218	Cleavage and polyadenylation specificity factor subunit 2 OS=Mus musculus GN=Cpsf2 P	1.6624041	1	1	1	88.327	5.11			740000	2.72
Q499E0	BMP/retinoic acid-inducible neural-specific protein 3 OS=Mus musculus GN=Brinp3 PE=2	1.6971279	1	1	1	88.426	7.9			520000	2.32
Q91WG7	Diacylglycerol kinase gamma OS=Mus musculus GN=Dgkg PE=1 SV=1	5.7106599	3	4	3	88.466	6.76	1400000		330000	11.98
Q8K4Z5	Splicing factor 3A subunit 1 OS=Mus musculus GN=Sf3a1 PE=1 SV=1	2.9077118	2	5	2	88.489	5.22	2600000	560000	1000000	12.86
Q920P3	BMP/retinoic acid-inducible neural-specific protein 1 OS=Mus musculus GN=Brinp1 PE=1	3.6842105	3	4	3	88.584	8.97	2500000	750000	1800000	8.54
P28028	Serine/threonine-protein kinase B-raf OS=Mus musculus GN=Braf PE=1 SV=3	1.4925373	1	1	1	88.724	7.65			380000	2.69
Q91W50	Cold shock domain-containing protein E1 OS=Mus musculus GN=Csde1 PE=1 SV=1	14.786967	10	15	10	88.735	6.37	2600000	590000	2700000	42.00
Q3B7Z2	Oxysterol-binding protein 1 OS=Mus musculus GN=Osbp PE=1 SV=3	1.3664596	1	1	1	88.741	7.2			700000	2.51
Q62318	Transcription intermediary factor 1-beta OS=Mus musculus GN=Trim28 PE=1 SV=3	10.911271	5	12	5	88.791	5.77	8700000	770000	5200000	46.31
Q9JLV5	Cullin-3 OS=Mus musculus GN=Cul3 PE=1 SV=1	13.802083	8	15	8	88.891	8.46	4300000	1300000	2800000	46.14
Q5SSM3	Rho GTPase-activating protein 44 OS=Mus musculus GN=Arhgap44 PE=1 SV=1	4.4226044	3	6	3	88.937	6.6	1300000	990000	2500000	16.28
Q9EQC5	N-terminal kinase-like protein OS=Mus musculus GN=Scyl1 PE=1 SV=1	4.3424318	2	4	2	89.104	6.44	2200000	870000	2000000	12.21
Q01853	Transitional endoplasmic reticulum ATPase OS=Mus musculus GN=Vcp PE=1 SV=4	38.709677	26	90	26	89.266	5.26	27000000	11000000	20000000	291.26
Q9Z268	RasGAP-activating-like protein 1 OS=Mus musculus GN=Rasal1 PE=1 SV=2	15.769712	11	22	11	89.339	6.37	9200000	2500000	5200000	68.60
Q8JZQ2	AFG3-like protein 2 OS=Mus musculus GN=Afg3l2 PE=1 SV=1	15.087282	13	35	13	89.463	8.6	4400000	1900000	2600000	103.67
Q8CCJ3	E3 UFM1-protein ligase 1 OS=Mus musculus GN=Ufl1 PE=1 SV=2	1.5132409	1	1	1	89.464	6.67	1300000			2.34
Q6R891	Neurabin-2 OS=Mus musculus GN=Ppp1r9b PE=1 SV=1	21.052632	11	26	11	89.466	4.92	12000000	3700000	12000000	80.10
Q05BC3	Echinoderm microtubule-associated protein-like 1 OS=Mus musculus GN=Eml1 PE=1 SV	1.3513514	1	1	1	89.624	7.02	1300000			2.12
Q9WTX6	Cullin-1 OS=Mus musculus GN=Cul1 PE=1 SV=1	3.9948454	2	3	2	89.635	8	1100000			7.90
Q9JLF6	Protein-glutamine gamma-glutamyltransferase K OS=Mus musculus GN=Tgm1 PE=1 SV=	2.5766871	2	4	2	89.769	6.51		440000	2200000	8.72
Q8BGD5	Carnitine O-palmitoyltransferase 1, brain isoform OS=Mus musculus GN=Cpt1c PE=1 SV	1.3784461	1	1	1	89.972	7.49	1500000			2.44
Q6NS52	Diacylglycerol kinase beta OS=Mus musculus GN=Dgkb PE=1 SV=2	1.7456359	1	2	1	90.213	7.93	1400000		1300000	5.66
P20918	Plasminogen OS=Mus musculus GN=Plg PE=1 SV=3	1.2315271	1	1	1	90.749	6.6		500000		0.00
Q6NXK7	Inactive dipeptidyl peptidase 10 OS=Mus musculus GN=Dpp10 PE=1 SV=1	11.668758	9	21	9	90.769	6.48	6900000	2900000	9400000	53.38
Q8C7M3	E3 ubiquitin-protein ligase TRIM9 OS=Mus musculus GN=Trim9 PE=1 SV=2	6.2423501	4	8	4	90.812	7.24	3000000	490000	2200000	22.39
Q04750	DNA topoisomerase 1 OS=Mus musculus GN=Top1 PE=1 SV=2	7.6923077	5	8	5	90.819	9.33	2800000		1900000	20.21

Q9Z315	U4/U6.U5 tri-snRNP-associated protein 1 OS=Mus musculus GN=Sart1 PE=1 SV=1	4.3424318	2	3	2	90.83	5.82	1900000		1200000	10.17
Q9D5V5	Cullin-5 OS=Mus musculus GN=Cul5 PE=1 SV=3	6.4102564	5	6	5	90.916	7.81	2400000		860000	15.63
Q69ZK9	Neuroligin-2 OS=Mus musculus GN=Nlgn2 PE=1 SV=2	9.9282297	6	17	5	90.932	6.18	8100000	5400000	10000000	48.81
O35286	Pre-mRNA-splicing factor ATP-dependent RNA helicase DHX15 OS=Mus musculus GN=I	5.0314465	3	5	3	90.949	7.46	3800000	1000000	1900000	15.30
Q8BYM5	Neuroligin-3 OS=Mus musculus GN=Nlgn3 PE=1 SV=2	7.5151515	5	10	4	91.104	5.8	5900000	1300000	4900000	32.01
Q6PHU5	Sortilin OS=Mus musculus GN=Sort1 PE=1 SV=1	2.9090909	2	3	2	91.142	5.88	990000	390000		7.86
Q9Z218	Dipeptidyl aminopeptidase-like protein 6 OS=Mus musculus GN=Dpp6 PE=1 SV=1	21.641791	14	33	14	91.203	6.68	11000000	8000000	11000000	98.63
P70188	Kinesin-associated protein 3 OS=Mus musculus GN=Kifap3 PE=1 SV=1	3.5308953	2	3	2	91.233	5.11		440000	680000	9.79
Q8BLY2	Probable threonine--tRNA ligase 2, cytoplasmic OS=Mus musculus GN=Tarsl2 PE=1 SV=	2.1518987	1	1	1	91.26	7.53	870000			3.34
P22892	AP-1 complex subunit gamma-1 OS=Mus musculus GN=Ap1g1 PE=1 SV=3	4.0145985	2	2	2	91.292	6.8	1100000		950000	5.66
Q8JZQ9	Eukaryotic translation initiation factor 3 subunit B OS=Mus musculus GN=Eif3b PE=1 SV=	18.181818	8	14	8	91.313	5.02	5100000	570000	3100000	43.63
Q61220	Protein kinase C-binding protein NELL2 OS=Mus musculus GN=Nell2 PE=1 SV=3	2.3199023	2	2	2	91.369	5.82			3700000	4.61
Q61165	Sodium/hydrogen exchanger 1 OS=Mus musculus GN=Slc9a1 PE=1 SV=1	2.0731707	1	1	1	91.41	7.12	1000000			3.81
Q9R1V7	Disintegrin and metalloproteinase domain-containing protein 23 OS=Mus musculus GN=A	7.1170084	4	13	4	91.488	7.68	2500000	2300000	1900000	36.49
Q9EQH3	Vacuolar protein sorting-associated protein 35 OS=Mus musculus GN=Vps35 PE=1 SV=1	11.432161	8	25	8	91.655	5.44	22000000	7600000	17000000	83.76
Q61548	Clathrin coat assembly protein AP180 OS=Mus musculus GN=Snap91 PE=1 SV=1	12.319645	11	38	10	91.794	4.88	52000000	21000000	37000000	113.06
Q3UYV9	Nuclear cap-binding protein subunit 1 OS=Mus musculus GN=Ncbp1 PE=1 SV=2	3.4177215	2	4	2	91.868	6.64	2100000		1100000	11.33
Q9DBT5	AMP deaminase 2 OS=Mus musculus GN=Ampd2 PE=1 SV=1	2.5062657	2	3	2	91.965	6.23	1500000		980000	7.55
Q14CH0	Protein FAM171B OS=Mus musculus GN=Fam171b PE=1 SV=2	2.5454545	1	1	1	91.988	8.37	850000			2.74
Q6ZQ82	Rho GTPase-activating protein 26 OS=Mus musculus GN=Arhgap26 PE=1 SV=3	3.6855037	2	5	2	92.012	6.68	2000000	620000	1400000	16.04
P15209	BDNF/NT-3 growth factors receptor OS=Mus musculus GN=Ntrk2 PE=1 SV=1	10.231425	6	13	6	92.074	6.55	6400000	5400000	7500000	39.48
Q6A068	Cell division cycle 5-like protein OS=Mus musculus GN=Cdc5l PE=1 SV=2	5.9850374	3	7	3	92.133	8.02	2300000		1200000	20.06
Q6ZQ18	Protein EFR3 homolog B OS=Mus musculus GN=Efr3b PE=1 SV=2	7.5887393	5	11	5	92.348	6.54	5900000	950000	3000000	30.98
P08113	Endoplasmin OS=Mus musculus GN=Hsp90b1 PE=1 SV=2	24.064838	17	48	15	92.418	4.82	22000000	10000000	21000000	158.61
Q8VD04	GRIP1-associated protein 1 OS=Mus musculus GN=Gripap1 PE=1 SV=1	11.042184	6	11	6	92.658	5.25	1900000	810000	2000000	38.10
Q8BUY8	G-protein coupled receptor-associated sorting protein 2 OS=Mus musculus GN=Gprasp2	2.0581114	1	2	1	92.744	5.11	940000			7.30
B2RUJ5	Amyloid beta A4 precursor protein-binding family A member 1 OS=Mus musculus GN=Apl	7.4821853	5	9	4	92.851	4.88	2300000	610000	1800000	22.80
P97366	Ecotropic viral integration site 5 protein OS=Mus musculus GN=Evi5 PE=1 SV=2	1.3597033	1	1	1	92.884	6.16				2.48
O35136	Neural cell adhesion molecule 2 OS=Mus musculus GN=Ncam2 PE=1 SV=1	21.983274	13	32	13	93.145	6.07	11000000	4000000	6300000	99.82
Q3TDQ1	Dolichyl-diphosphooligosaccharide--protein glycosyltransferase subunit STT3B OS=Mus r	2.308627	2	4	2	93.185	8.95	3100000	1300000	2600000	11.46
Q8BJ71	Nuclear pore complex protein Nup93 OS=Mus musculus GN=Nup93 PE=1 SV=1	1.3431013	1	2	1	93.222	5.72	150000		610000	5.25
P70175	Disks large homolog 3 OS=Mus musculus GN=Dlg3 PE=1 SV=1	10.365135	7	18	6	93.424	6.79	3800000	940000	3400000	52.58
Q9JIK5	Nucleolar RNA helicase 2 OS=Mus musculus GN=Ddx21 PE=1 SV=3	0.9400705	1	1	1	93.493	9.11		19000000		1.96
P18052	Receptor-type tyrosine-protein phosphatase alpha OS=Mus musculus GN=Ptpra PE=1 SV	5.066345	4	7	4	93.638	7.05	1600000		1700000	17.55
Q80WG5	Volume-regulated anion channel subunit LRRC8A OS=Mus musculus GN=Lrrc8a PE=1 S	1.1111111	1	1	1	94.06	7.94	480000			2.18
A6H5Z3	Exocyst complex component 6B OS=Mus musculus GN=Exoc6b PE=1 SV=1	4.1975309	2	2	2	94.07	6.39	1300000			5.55
Q61316	Heat shock 70 kDa protein 4 OS=Mus musculus GN=Hspa4 PE=1 SV=1	33.174792	21	58	21	94.073	5.24	14000000	5800000	12000000	176.74
E9Q6P5	Tetratricopeptide repeat protein 7B OS=Mus musculus GN=Ttc7b PE=1 SV=1	4.5077106	3	6	3	94.144	6.9	1500000		1300000	16.27
Q6NS60	F-box only protein 41 OS=Mus musculus GN=Fbxo41 PE=1 SV=3	6.4146621	3	4	3	94.272	8.24			1700000	9.83
Q80T74	Kelch-like protein 29 OS=Mus musculus GN=Klhl29 PE=2 SV=3	1.0285714	1	1	1	94.3	7.17		6400000		1.88
P48722	Heat shock 70 kDa protein 4L OS=Mus musculus GN=Hspa4l PE=1 SV=2	26.849642	17	39	17	94.322	5.74	6800000	2300000	6400000	131.55
Q6P5F6	Zinc transporter ZIP10 OS=Mus musculus GN=Slc39a10 PE=1 SV=1	4.3217287	3	7	3	94.335	6.71	2600000	930000	1700000	23.16
Q8CCB4	Vacuolar protein sorting-associated protein 53 homolog OS=Mus musculus GN=Vps53 PE	1.2019231	1	2	1	94.363	6.61	1500000		610000	4.95
Q9DBY8	Nuclear valosin-containing protein-like OS=Mus musculus GN=Nvl PE=1 SV=1	1.754386	1	2	1	94.417	6.35				5.01
Q8K310	Matrin-3 OS=Mus musculus GN=Matr3 PE=1 SV=1	19.267139	15	38	15	94.572	6.25	42000000	2100000	28000000	112.51
O88703	Potassium/sodium hyperpolarization-activated cyclic nucleotide-gated channel 2 OS=Mus	2.0857474	1	3	1	94.661	8.73	1500000	430000	1100000	8.92
Q9ER72	Cysteine--tRNA ligase, cytoplasmic OS=Mus musculus GN=Cars PE=1 SV=2	1.8050542	1	2	1	94.8	6.76	2700000	650000		7.21
Q91XM9	Disks large homolog 2 OS=Mus musculus GN=Dlg2 PE=1 SV=2	23.004695	15	40	14	94.821	6.24	16000000	1100000	16000000	122.12

Q6P542	ATP-binding cassette sub-family F member 1 OS=Mus musculus GN=Abcf1 PE=1 SV=1	4.0621266	2	4	2	94.887	6.51	1400000		1200000	13.92
P58252	Elongation factor 2 OS=Mus musculus GN=Eef2 PE=1 SV=2	21.212121	16	43	15	95.253	6.83	21000000	3500000	11000000	118.59
Q3UNH4	G protein-regulated inducer of neurite outgrowth 1 OS=Mus musculus GN=Gprin1 PE=1 S	26.39485	20	48	20	95.437	7.93	12000000	4600000	10000000	143.35
Q8C079	Striatin-interacting protein 1 OS=Mus musculus GN=Strip1 PE=1 SV=2	3.1063321	1	2	1	95.524	6.25	2200000		540000	7.61
Q03717	Potassium voltage-gated channel subfamily B member 1 OS=Mus musculus GN=Kcnb1 F	1.4002334	1	2	1	95.53	8.16	3500000		1900000	5.93
Q61361	Brevican core protein OS=Mus musculus GN=Bcan PE=1 SV=2	25.934315	20	84	20	95.755	4.93	45000000	34000000	57000000	263.43
P56399	Ubiquitin carboxyl-terminal hydrolase 5 OS=Mus musculus GN=Usp5 PE=1 SV=1	15.850816	10	23	10	95.772	5.01	8000000	2200000	7500000	65.68
Q14BI2	Metabotropic glutamate receptor 2 OS=Mus musculus GN=Grm2 PE=1 SV=2	7.1100917	5	8	4	95.826	8.06	11000000	1400000	3700000	22.16
Q4KMM3	Oxidation resistance protein 1 OS=Mus musculus GN=Oxr1 PE=1 SV=3	25.635104	16	37	16	95.852	5.33	9600000	3100000	10000000	131.24
P08553	Neurofilament medium polypeptide OS=Mus musculus GN=Nefm PE=1 SV=4	40.330189	31	85	27	95.859	4.77	22000000	5000000	31000000	249.53
Q9Z1X4	Interleukin enhancer-binding factor 3 OS=Mus musculus GN=Ilf3 PE=1 SV=2	11.469933	7	12	7	95.961	8.76	3800000		2200000	36.34
Q9WU78	Programmed cell death 6-interacting protein OS=Mus musculus GN=Pdcd6ip PE=1 SV=3	2.9919448	2	3	2	95.964	6.52	1100000		550000	6.28
P58069	Ras GTPase-activating protein 2 OS=Mus musculus GN=Rasa2 PE=1 SV=2	1.2987013	1	1	1	96.34	7.59				2.08
Q61699	Heat shock protein 105 kDa OS=Mus musculus GN=Hsph1 PE=1 SV=2	23.659674	13	36	13	96.346	5.53	7100000	1800000	5300000	107.60
Q7TSJ2	Microtubule-associated protein 6 OS=Mus musculus GN=Map6 PE=1 SV=2	43.156733	30	100	30	96.391	9.5	26000000	8500000	17000000	343.21
Q9Z1G4	V-type proton ATPase 116 kDa subunit a isoform 1 OS=Mus musculus GN=Atp6v0a1 PE=	22.169249	17	69	17	96.404	6.76	110000000	29000000	65000000	226.14
Q8CI94	Glycogen phosphorylase, brain form OS=Mus musculus GN=Pygb PE=1 SV=3	28.113879	20	50	15	96.668	6.73	32000000	10000000	16000000	138.44
Q8K4G5	Actin-binding LIM protein 1 OS=Mus musculus GN=Ablim1 PE=1 SV=1	2.0905923	2	3	2	96.743	8.63	6800000		1100000	7.25
Q62165	Dystroglycan OS=Mus musculus GN=Dag1 PE=1 SV=4	4.5912654	3	11	3	96.844	8.44	2100000	2200000	2400000	26.60
Q3URD3	Sarcolemmal membrane-associated protein OS=Mus musculus GN=Slmap PE=1 SV=2	2.2485207	2	2	2	96.873	5.33	1700000		200000	4.51
Q60625	Intercellular adhesion molecule 5 OS=Mus musculus GN=Icam5 PE=1 SV=2	13.522356	9	26	9	96.885	6.32	9400000	7000000	13000000	79.51
Q9ES28	Rho guanine nucleotide exchange factor 7 OS=Mus musculus GN=Arhgef7 PE=1 SV=2	4.4083527	3	6	3	96.995	6.8	2600000	1600000	4500000	17.96
P70168	Importin subunit beta-1 OS=Mus musculus GN=Kpnb1 PE=1 SV=2	11.415525	8	18	8	97.122	4.78	4600000	1100000	2900000	48.32
Q8BZ98	Dynamin-3 OS=Mus musculus GN=Dnm3 PE=1 SV=1	18.308227	16	45	9	97.13	8.35	11000000	3800000	6300000	129.36
Q9WUB3	Glycogen phosphorylase, muscle form OS=Mus musculus GN=Pygm PE=1 SV=3	14.845606	11	24	6	97.225	7.11	6700000	1600000	2800000	63.81
B0F2B4	Neuroligin 4-like OS=Mus musculus GN=Nlgn4l PE=1 SV=1	3.5978836	3	5	2	97.287	6.32	2100000	1700000	1800000	13.01
Q4V9Z5	Seizure 6-like protein 2 OS=Mus musculus GN=Sez6l2 PE=1 SV=1	7.4725275	4	12	4	97.442	4.87	3200000	2500000	2900000	38.10
Q9QZE5	Coatomer subunit gamma-1 OS=Mus musculus GN=Copg1 PE=1 SV=1	2.0594966	1	2	1	97.45	5.35	1500000		1000000	6.51
Q5SSL4	Active breakpoint cluster region-related protein OS=Mus musculus GN=Abr PE=1 SV=1	6.9848661	4	8	4	97.605	6.58	3800000	810000	3300000	25.68
Q9QXK3	Coatomer subunit gamma-2 OS=Mus musculus GN=Copg2 PE=1 SV=1	1.9517796	1	2	1	97.617	5.8	2000000		1100000	6.29
P39053	Dynamin-1 OS=Mus musculus GN=Dnm1 PE=1 SV=2	49.365629	41	163	34	97.741	7.74	140000000	64000000	93000000	508.06
Q8VHH5	Arf-GAP with GTPase, ANK repeat and PH domain-containing protein 3 OS=Mus musculu	2.967033	2	6	2	97.905	7.75	3400000	1600000	2900000	20.50
P28271	Cytoplasmic aconitate hydratase OS=Mus musculus GN=Aco1 PE=1 SV=3	2.4746907	1	1	1	98.063	7.5				2.89
Q9Z2H5	Band 4.1-like protein 1 OS=Mus musculus GN=Epb41l1 PE=1 SV=2	22.639363	13	29	13	98.254	5.62	11000000	1100000	6300000	87.30
O54931	A-kinase anchor protein 2 OS=Mus musculus GN=Akap2 PE=1 SV=3	1.1198208	1	2	1	98.519	5.21	720000		710000	4.19
P23819	Glutamate receptor 2 OS=Mus musculus GN=Gria2 PE=1 SV=3	22.083805	17	49	15	98.599	7.39	20000000	6800000	15000000	144.81
Q8R0Y6	Cytosolic 10-formyltetrahydrofolate dehydrogenase OS=Mus musculus GN=Aldh1l1 PE=1	13.192905	9	18	9	98.647	5.91	8700000	1200000	3600000	55.68
Q8K1S4	Netrin receptor UNC5A OS=Mus musculus GN=Unc5a PE=1 SV=1	1.8930958	1	1	1	98.794	6.92	990000			3.50
Q9QYS2	Metabotropic glutamate receptor 3 OS=Mus musculus GN=Grm3 PE=1 SV=1	9.556314	7	20	6	99.05	7.75	6400000	1900000	4000000	57.25
Q80YR4	Zinc finger protein 598 OS=Mus musculus GN=Znf598 PE=1 SV=1	3.6343612	2	2	2	99.13	8.34	190000			5.28
Q7TSQ8	Pyruvate dehydrogenase phosphatase regulatory subunit, mitochondrial OS=Mus muscul	2.7334852	2	3	2	99.167	6.35	1400000	520000	1700000	8.19
Q60902	Epidermal growth factor receptor substrate 15-like 1 OS=Mus musculus GN=Eps15l1 PE=	19.294377	14	32	14	99.248	5.02	2900000	2200000	3300000	94.45
O35551	Rab GTPase-binding effector protein 1 OS=Mus musculus GN=Rabep1 PE=1 SV=2	2.4361949	2	2	2	99.462	5.01	380000		1200000	5.38
Q8CC35	Synaptopodin OS=Mus musculus GN=Synpo PE=1 SV=2	7.8579117	4	9	4	99.49	9.42	8900000	2800000	5700000	32.89
B2RY56	RNA-binding protein 25 OS=Mus musculus GN=Rbm25 PE=1 SV=2	10.023866	5	7	5	99.491	6.32	2000000		1200000	21.60
Q9R1V6	Disintegrin and metalloproteinase domain-containing protein 22 OS=Mus musculus GN=A	15.044248	11	30	11	99.651	7.83	11000000	3200000	10000000	92.72
P15116	Cadherin-2 OS=Mus musculus GN=Cdh2 PE=1 SV=2	8.388521	5	10	5	99.734	4.78	9100000	1600000	9200000	37.47
O08759	Ubiquitin-protein ligase E3A OS=Mus musculus GN=Ube3a PE=1 SV=2	2.6436782	2	4	2	99.756	5.08	2700000	1000000	2100000	10.65

P26231	Catenin alpha-1 OS=Mus musculus GN=Ctnna1 PE=1 SV=1	6.5121413	5	11	1	100.044	6.23				32.55
Q811D0	Disks large homolog 1 OS=Mus musculus GN=Dlg1 PE=1 SV=1	15.690608	10	23	8	100.058	5.8	10000000	3800000	5500000	69.05
Q3UVK0	Endoplasmic reticulum metallopeptidase 1 OS=Mus musculus GN=Ermp1 PE=1 SV=2	1.2249443	1	1	1	100.084	7.49	1400000			2.13
Q8VDM4	26S proteasome non-ATPase regulatory subunit 2 OS=Mus musculus GN=Psmd2 PE=1 S	7.9295154	6	17	6	100.139	5.17	3400000	1500000	3700000	56.07
Q80XR2	Calcium-transporting ATPase type 2C member 1 OS=Mus musculus GN=Atp2c1 PE=1 SV	1.1982571	1	1	1	100.234	6.83	850000			2.99
Q9WU40	Inner nuclear membrane protein Man1 OS=Mus musculus GN=Lemd3 PE=1 SV=2	5.4288817	3	4	3	100.244	7.55	520000		170000	15.25
Q99MR6	Serrate RNA effector molecule homolog OS=Mus musculus GN=Srrt PE=1 SV=1	3.8857143	3	4	3	100.39	5.97	5100000		3000000	10.76
Q99LG2	Transportin-2 OS=Mus musculus GN=Tnpo2 PE=1 SV=1	1.1273957	1	1	1	100.391	4.98	680000			2.19
Q8R3Q2	Serine/threonine-protein phosphatase 6 regulatory subunit 2 OS=Mus musculus GN=Ppp	1.7334778	1	1	1	100.397	4.82	1200000			3.52
Q9Z2W9	Glutamate receptor 3 OS=Mus musculus GN=Gria3 PE=1 SV=2	8.2207207	6	18	4	100.463	8.38	7000000	2600000	4900000	54.04
P56695	Wolframin OS=Mus musculus GN=Wfs1 PE=1 SV=1	3.8202247	3	7	3	100.514	7.97	3800000	6500000	11000000	19.40
Q8K596	Sodium/calcium exchanger 2 OS=Mus musculus GN=Slc8a2 PE=1 SV=1	18.023887	13	49	12	100.647	5.12	28000000	14000000	20000000	151.79
Q9D2V7	Coronin-7 OS=Mus musculus GN=Coro7 PE=1 SV=2	1.7353579	1	3	1	100.749	5.77	3200000	1400000	3300000	7.27
Q9Z2W8	Glutamate receptor 4 OS=Mus musculus GN=Gria4 PE=1 SV=2	4.2128603	3	7	1	100.782	8.07				23.42
Q8CCN5	Breast carcinoma-amplified sequence 3 homolog OS=Mus musculus GN=Bcas3 PE=1 SV	2.0474138	1	1	1	100.957	6.7	500000			2.57
Q80WT5	Aftiphilin OS=Mus musculus GN=Aftph PE=1 SV=2	3.7593985	2	2	2	101.068	4.5	590000			6.91
Q922D8	C-1-tetrahydrofolate synthase, cytoplasmic OS=Mus musculus GN=Mthfd1 PE=1 SV=4	8.4491979	7	12	7	101.136	7.14	3400000	1300000	2300000	28.51
Q6PE13	Proline-rich transmembrane protein 3 OS=Mus musculus GN=Prrt3 PE=1 SV=1	1.4418126	1	1	1	101.162	7.52		1400000		2.98
B9EJ86	Oxysterol-binding protein-related protein 8 OS=Mus musculus GN=Osbpl8 PE=1 SV=1	1.2373453	1	1	1	101.205	6.96	3000000			2.46
Q5QNQ6	Oxysterol-binding protein 2 OS=Mus musculus GN=Osbp2 PE=1 SV=1	1.3215859	1	1	1	101.289	7.14	850000			2.14
Q6PF93	Phosphatidylinositol 3-kinase catalytic subunit type 3 OS=Mus musculus GN=Pik3c3 PE=	1.8038331	1	3	1	101.423	6.73	1500000	430000	1100000	8.36
P23818	Glutamate receptor 1 OS=Mus musculus GN=Gria1 PE=1 SV=1	5.9536935	5	10	3	101.504	7.69	6000000	1300000	4100000	28.51
Q68EF4	Metabotropic glutamate receptor 4 OS=Mus musculus GN=Grm4 PE=1 SV=2	1.3157895	1	2	1	101.758	8.82	320000			4.83
Q8R3S6	Exocyst complex component 1 OS=Mus musculus GN=Exoc1 PE=1 SV=4	2.2371365	2	3	2	101.825	6.52	1400000	850000		4.68
Q810A7	ATP-dependent RNA helicase DDX42 OS=Mus musculus GN=Ddx42 PE=1 SV=3	3.0139935	2	2	2	101.902	6.98	2100000			6.02
Q78PY7	Staphylococcal nuclease domain-containing protein 1 OS=Mus musculus GN=Snd1 PE=1	9.7802198	7	14	7	102.025	7.43	6500000	1100000	2000000	39.76
Q62448	Eukaryotic translation initiation factor 4 gamma 2 OS=Mus musculus GN=Eif4g2 PE=1 SV	3.3112583	2	2	2	102.041	7.14	2300000			4.90
Q68ED2	Metabotropic glutamate receptor 7 OS=Mus musculus GN=Grm7 PE=1 SV=1	1.0928962	1	1	1	102.153	7.88	1500000			2.86
Q80TZ3	Putative tyrosine-protein phosphatase auxilin OS=Mus musculus GN=Dnajc6 PE=1 SV=2	8.7420043	7	17	7	102.235	7.23	7500000	1400000	4100000	45.92
Q8CGF6	WD repeat-containing protein 47 OS=Mus musculus GN=Wdr47 PE=1 SV=2	8.3695652	5	8	5	102.247	5.97	2300000	870000	1800000	23.79
O88704	Potassium/sodium hyperpolarization-activated cyclic nucleotide-gated channel 1 OS=Mus	2.1978022	1	1	1	102.366	8.37			420000	4.06
O55029	Coatomer subunit beta' OS=Mus musculus GN=Copb2 PE=1 SV=2	4.5303867	3	3	3	102.384	5.3		630000	1000000	7.34
P39087	Glutamate receptor ionotropic, kainate 2 OS=Mus musculus GN=Grik2 PE=1 SV=4	3.0837004	2	2	2	102.42	7.77	600000		940000	3.98
P46935	E3 ubiquitin-protein ligase NEDD4 OS=Mus musculus GN=Nedd4 PE=1 SV=3	7.5535513	5	11	4	102.642	5.26	2200000	800000	1100000	35.23
Q8VDP4	Cell cycle and apoptosis regulator protein 2 OS=Mus musculus GN=Ccar2 PE=1 SV=2	9.6529284	5	10	5	102.938	5.25	5600000	1100000	1600000	34.59
Q7TPR4	Alpha-actinin-1 OS=Mus musculus GN=Actn1 PE=1 SV=1	45.73991	34	133	18	103.004	5.38	26000000	14000000	28000000	439.20
Q9EQQ9	Protein O-GlcNAcase OS=Mus musculus GN=Mgea5 PE=1 SV=2	2.7292576	2	3	2	103.096	4.92	2100000	1300000	1800000	8.82
Q921G8	Gamma-tubulin complex component 2 OS=Mus musculus GN=Tubgcp2 PE=1 SV=2	1.878453	1	1	1	103.157	6.77	420000			2.11
Q922S4	cGMP-dependent 3',5'-cyclic phosphodiesterase OS=Mus musculus GN=Pde2a PE=1 SV	11.353712	9	21	9	103.183	5.38	11000000	3100000	8800000	73.28
Q11011	Puromycin-sensitive aminopeptidase OS=Mus musculus GN=Npepps PE=1 SV=2	4.5652174	4	8	4	103.26	5.9	3600000	1400000	4100000	22.25
Q9WV92	Band 4.1-like protein 3 OS=Mus musculus GN=Epb41l3 PE=1 SV=1	35.306781	26	76	23	103.274	5.31	43000000	11000000	33000000	243.70
Q3UHJ0	AP2-associated protein kinase 1 OS=Mus musculus GN=Aak1 PE=1 SV=2	31.178311	18	42	18	103.282	6.7	16000000	5600000	13000000	151.62
Q6NZL0	Protein SOGA3 OS=Mus musculus GN=Soga3 PE=1 SV=2	12.169312	10	20	10	103.416	6.14	5700000	2400000	4700000	57.60
Q8K1A6	Coiled-coil and C2 domain-containing protein 1A OS=Mus musculus GN=Cc2d1a PE=1 S	0.9544008	1	1	1	103.634	7.84	520000			2.08
Q9JI91	Alpha-actinin-2 OS=Mus musculus GN=Actn2 PE=1 SV=2	15.100671	12	32	1	103.768	5.45	1900000	690000	2400000	104.40
Q9ES97	Reticulon-3 OS=Mus musculus GN=Rtn3 PE=1 SV=2	32.883817	22	51	21	103.814	4.92	32000000	9300000	38000000	148.17
O35643	AP-1 complex subunit beta-1 OS=Mus musculus GN=Ap1b1 PE=1 SV=2	20.148462	18	49	6	103.869	5.17	12000000	1900000	8700000	140.38
Q9D4H1	Exocyst complex component 2 OS=Mus musculus GN=Exoc2 PE=1 SV=1	5.952381	5	5	5	103.894	7.18	2000000			14.57

Q0GA42	Metal transporter CNNM1 OS=Mus musculus GN=Cnnm1 PE=1 SV=5	1.4721346	1	1	1	103.915	6.51	1700000			3.54
P17427	AP-2 complex subunit alpha-2 OS=Mus musculus GN=Ap2a2 PE=1 SV=2	25.799574	22	61	16	103.951	6.93	20000000	7800000	10000000	167.44
Q80UP3	Diacylglycerol kinase zeta OS=Mus musculus GN=Dgkz PE=1 SV=2	3.444564	2	3	2	103.965	8.09	4800000		1800000	8.96
Q6NVF0	Inositol polyphosphate 5-phosphatase OCRL-1 OS=Mus musculus GN=Ocrl PE=1 SV=1	0.8888889	1	1	1	104.219	6.27				1.89
Q3UUQ7	GPI inositol-deacylase OS=Mus musculus GN=Pgap1 PE=1 SV=3	1.1930586	1	1	1	104.511	8.91	1800000			2.77
Q9DBG3	AP-2 complex subunit beta OS=Mus musculus GN=Ap2b1 PE=1 SV=1	31.376734	25	72	13	104.516	5.38	38000000	13000000	27000000	204.72
Q6P1D5	Seizure 6-like protein OS=Mus musculus GN=Sez6l PE=1 SV=1	1.5576324	1	1	1	104.76	4.69		920000		3.44
Q8K212	Phosphofurin acidic cluster sorting protein 1 OS=Mus musculus GN=Pacs1 PE=1 SV=2	11.550468	8	16	8	104.764	7.74	10000000	880000	3100000	32.85
P58501	PAX3- and PAX7-binding protein 1 OS=Mus musculus GN=Paxbp1 PE=1 SV=3	1.6322089	1	1	1	104.771	5.67	610000			3.17
P30999	Catenin delta-1 OS=Mus musculus GN=Ctnnd1 PE=1 SV=2	3.6247335	3	5	3	104.86	6.87	1800000		2500000	13.33
Q3UMB5	Smith-Magenis syndrome chromosomal region candidate gene 8 protein homolog OS=Mu	2.9946524	2	4	2	104.891	5.4	1000000		1100000	9.17
P57780	Alpha-actinin-4 OS=Mus musculus GN=Actn4 PE=1 SV=1	45.394737	36	131	22	104.911	5.41	17000000	8800000	21000000	424.18
P98203	Armadillo repeat protein deleted in velo-cardio-facial syndrome homolog OS=Mus muscul	1.8711019	1	1	1	105.002	6.64			1900000	3.25
D3YXK2	Scaffold attachment factor B1 OS=Mus musculus GN=Safb PE=1 SV=2	18.036286	10	21	10	105.04	5.35	7900000		4800000	68.57
O70589	Peripheral plasma membrane protein CASK OS=Mus musculus GN=Cask PE=1 SV=2	5.075594	4	7	4	105.042	6.43	5400000		3800000	16.35
Q61301	Catenin alpha-2 OS=Mus musculus GN=Ctnna2 PE=1 SV=3	22.035677	20	52	16	105.22	5.71	12000000	2000000	9100000	149.42
Q99K46	Ubiquitin carboxyl-terminal hydrolase 11 OS=Mus musculus GN=Usp11 PE=1 SV=4	4.3431053	2	4	2	105.318	4.96	1200000		560000	13.28
P35438	Glutamate receptor ionotropic, NMDA 1 OS=Mus musculus GN=Grin1 PE=1 SV=1	5.1172708	5	8	5	105.414	8.84	6000000	2200000	4800000	19.24
Q3UTQ8	Cyclin-dependent kinase-like 5 OS=Mus musculus GN=Cdkl5 PE=1 SV=1	3.9445629	3	3	3	105.425	9.58	440000		3400000	7.59
Q8R1B4	Eukaryotic translation initiation factor 3 subunit C OS=Mus musculus GN=Eif3c PE=1 SV=	8.1229418	6	10	6	105.465	5.78	4400000		3000000	31.76
Q9EPW0	Type I inositol 3,4-bisphosphate 4-phosphatase OS=Mus musculus GN=Inpp4a PE=1 SV=	7.028754	5	12	5	105.472	7.05	2400000	650000	1500000	32.30
Q80T41	Gamma-aminobutyric acid type B receptor subunit 2 OS=Mus musculus GN=Gabbr2 PE=	7.5531915	6	10	6	105.599	8.72	3300000	520000	2500000	25.56
Q0P678	Zinc finger CCCH domain-containing protein 18 OS=Mus musculus GN=Zc3h18 PE=1 SV	8.1223629	3	4	3	105.631	7.8	1900000		1400000	14.62
Q3V3R1	Monofunctional C1-tetrahydrofolate synthase, mitochondrial OS=Mus musculus GN=Mthfc	10.030706	9	12	9	105.662	7.02	2300000		1700000	29.44
Q3TXS7	26S proteasome non-ATPase regulatory subunit 1 OS=Mus musculus GN=Psmd1 PE=1 S	14.900315	10	20	10	105.663	5.39	4300000	830000	3400000	59.01
Q8CGK3	Lon protease homolog, mitochondrial OS=Mus musculus GN=Lonp1 PE=1 SV=2	10.32666	7	15	7	105.776	6.57	5400000	4000000	4300000	49.21
Q99JH7	Calsyntenin-3 OS=Mus musculus GN=Clstn3 PE=1 SV=1	2.1966527	1	1	1	105.805	5.34			1500000	2.89
Q6PFD5	Disks large-associated protein 3 OS=Mus musculus GN=Dlgap3 PE=1 SV=1	9.9283521	8	13	8	105.808	8.76	7400000		4000000	35.63
Q8K019	Bcl-2-associated transcription factor 1 OS=Mus musculus GN=Bclaf1 PE=1 SV=2	8.8139282	6	16	5	105.939	9.99	5100000		4100000	46.94
Q8CGZ0	Calcium homeostasis endoplasmic reticulum protein OS=Mus musculus GN=Cherp PE=1	1.4957265	1	1	1	106.102	9.14	970000			3.49
Q8K1S2	Netrin receptor UNC5D OS=Mus musculus GN=Unc5d PE=1 SV=1	1.6736402	1	3	1	106.284	6.14	350000		750000	8.13
Q3UHE1	Membrane-associated phosphatidylinositol transfer protein 3 OS=Mus musculus GN=Pitpi	2.0533881	1	1	1	106.395	7.12	820000			3.39
Q80TQ2	Ubiquitin carboxyl-terminal hydrolase CYLD OS=Mus musculus GN=Cyld PE=1 SV=2	1.2605042	1	1	1	106.517	5.63			880000	2.27
P60755	MAM domain-containing glycosylphosphatidylinositol anchor protein 2 OS=Mus musculus	1.1591149	1	2	1	106.622	7.02		660000	690000	3.72
Q8BH74	Nuclear pore complex protein Nup107 OS=Mus musculus GN=Nup107 PE=1 SV=1	1.2958963	1	1	1	106.65	5.43			390000	2.73
Q91YR7	Pre-mRNA-processing factor 6 OS=Mus musculus GN=Prpf6 PE=1 SV=1	5.6323061	5	7	5	106.655	8.09	1200000		540000	17.46
Q7SIG6	Arf-GAP with SH3 domain, ANK repeat and PH domain-containing protein 2 OS=Mus mus	3.5490605	2	3	2	106.738	6.65	1800000		730000	9.05
Q52KI8	Serine/arginine repetitive matrix protein 1 OS=Mus musculus GN=Srrm1 PE=1 SV=2	2.6427061	2	3	2	106.798	11.87	6600000		2800000	8.34
Q8BGQ7	Alanine--tRNA ligase, cytoplasmic OS=Mus musculus GN=Aars PE=1 SV=1	4.6487603	4	10	4	106.841	5.67	4100000	1300000	3600000	28.93
Q8BHN3	Neutral alpha-glucosidase AB OS=Mus musculus GN=Ganab PE=1 SV=1	7.6271186	5	8	5	106.844	6.06	2400000	2300000	2400000	26.60
Q9Z1Z0	General vesicular transport factor p115 OS=Mus musculus GN=Uso1 PE=1 SV=2	18.769552	12	26	12	106.917	4.93	4400000	1000000	4100000	80.53
Q9JIF7	Coatomer subunit beta OS=Mus musculus GN=Copb1 PE=1 SV=1	5.5613851	4	5	4	106.998	6	2700000	1100000	1600000	12.35
Q8K1R7	Serine/threonine-protein kinase Nek9 OS=Mus musculus GN=Nek9 PE=1 SV=2	1.4227642	1	1	1	107.075	5.63				3.20
A2AJA9	Uncharacterized protein C9orf172 homolog OS=Mus musculus GN=Gm996 PE=1 SV=1	6.3655031	5	9	5	107.119	9.11	2800000		2000000	22.90
P17426	AP-2 complex subunit alpha-1 OS=Mus musculus GN=Ap2a1 PE=1 SV=1	28.249744	23	67	17	107.596	7.03	30000000	6800000	19000000	181.15
Q91W86	Vacuolar protein sorting-associated protein 11 homolog OS=Mus musculus GN=Vps11 PE	1.1689692	1	2	1	107.65	7.01	1500000		1700000	5.81
Q8R0S2	IQ motif and SEC7 domain-containing protein 1 OS=Mus musculus GN=Iqsec1 PE=1 SV=	14.360042	12	21	9	107.948	7.14	26000000	32000000	17000000	54.93
P70414	Sodium/calcium exchanger 1 OS=Mus musculus GN=Slc8a1 PE=1 SV=1	9.0721649	6	14	6	107.967	5	6600000	3500000	6500000	39.09

B1AZP2	Disks large-associated protein 4 OS=Mus musculus GN=Dlgap4 PE=1 SV=1	6.8548387	5	12	4	107.97	7.08	4900000	770000	3900000	36.07
Q569Z6	Thyroid hormone receptor-associated protein 3 OS=Mus musculus GN=Thrap3 PE=1 SV=	10.304942	8	18	7	108.114	10.17	3800000		3600000	50.10
Q9WV18	Gamma-aminobutyric acid type B receptor subunit 1 OS=Mus musculus GN=Gabbr1 PE=	5.8333333	5	8	5	108.147	8.21	4000000		3400000	21.20
P17710	Hexokinase-1 OS=Mus musculus GN=Hk1 PE=1 SV=3	30.800821	28	88	28	108.234	6.8	120000000	36000000	75000000	257.17
P35123	Ubiquitin carboxyl-terminal hydrolase 4 OS=Mus musculus GN=Usp4 PE=1 SV=3	2.4948025	1	2	1	108.274	5.64	940000		810000	8.59
Q9DBR1	5'-3' exoribonuclease 2 OS=Mus musculus GN=Xrn2 PE=1 SV=1	1.2618297	1	1	1	108.618	7.59	580000			2.93
Q9EPL2	Calsyntenin-1 OS=Mus musculus GN=Clstn1 PE=1 SV=1	0.8171604	1	1	1	108.831	4.92			1800000	2.00
Q8BG95	Protein phosphatase 1 regulatory subunit 12B OS=Mus musculus GN=Ppp1r12b PE=1 SV	7.6844262	5	10	4	108.984	5.74	1100000	530000	960000	31.66
Q8BWQ6	UPF0505 protein C16orf62 homolog OS=Mus musculus PE=1 SV=2	1.3499481	1	1	1	109.007	7.31	620000			2.28
P28738	Kinesin heavy chain isoform 5C OS=Mus musculus GN=Kif5c PE=1 SV=3	19.246862	16	30	11	109.207	6.19	6100000	2400000	5100000	104.01
O08810	116 kDa U5 small nuclear ribonucleoprotein component OS=Mus musculus GN=Eftud2 P	5.4582904	5	8	4	109.291	5	3200000		1300000	17.36
Q61768	Kinesin-1 heavy chain OS=Mus musculus GN=Kif5b PE=1 SV=3	18.587747	14	22	9	109.484	6.44	1200000	210000	1400000	67.11
Q9JLI8	Squamous cell carcinoma antigen recognized by T-cells 3 OS=Mus musculus GN=Sart3 F	3.950104	2	4	2	109.55	5.24	1400000		960000	14.64
Q5DTT2	PH and SEC7 domain-containing protein 1 OS=Mus musculus GN=Psd PE=1 SV=2	1.0742188	1	1	1	109.619	7.05	470000			2.05
Q03137	Ephrin type-A receptor 4 OS=Mus musculus GN=Epha4 PE=1 SV=2	9.3306288	6	12	6	109.744	6.51	4500000	1600000	2800000	37.63
P54763	Ephrin type-B receptor 2 OS=Mus musculus GN=Ephb2 PE=1 SV=3	1.8255578	1	1	1	109.828	5.71			680000	2.75
O70318	Band 4.1-like protein 2 OS=Mus musculus GN=Epb41l2 PE=1 SV=2	19.939271	17	37	14	109.873	5.43	9000000	3500000	7900000	106.91
Q80UJ7	Rab3 GTPase-activating protein catalytic subunit OS=Mus musculus GN=Rab3gap1 PE=	4.1794088	3	5	3	110.128	5.73	2700000	880000	2600000	15.39
Q922P8	Transmembrane protein 132A OS=Mus musculus GN=Tmem132a PE=1 SV=2	1.9646365	1	2	1	110.171	5.63	1100000			5.68
Q9D415	Disks large-associated protein 1 OS=Mus musculus GN=Dlgap1 PE=1 SV=3	6.4516129	5	9	5	110.305	7.09	9400000		6500000	23.30
Q9ERK4	Exportin-2 OS=Mus musculus GN=Cse1l PE=1 SV=1	4.5314109	3	4	3	110.384	5.77	1900000		970000	12.84
O35382	Exocyst complex component 4 OS=Mus musculus GN=Exoc4 PE=1 SV=2	6.1538462	4	9	4	110.475	6.49	2500000	500000	1300000	27.82
Q6PH08	ERC protein 2 OS=Mus musculus GN=Erc2 PE=1 SV=2	15.987461	14	30	10	110.571	7.03	7900000	3200000	9400000	88.64
Q7TQH0	Ataxin-2-like protein OS=Mus musculus GN=Atxn2l PE=1 SV=1	3.5271687	3	6	3	110.58	8.85	620000		450000	14.18
A2A432	Cullin-4B OS=Mus musculus GN=Cul4b PE=1 SV=1	3.1958763	3	5	3	110.63	8.37	1300000		690000	12.04
Q8CI71	Syndetin OS=Mus musculus GN=Vps50 PE=1 SV=2	5.0829876	4	8	4	111.104	6.07	2100000	490000	1400000	22.22
Q9JKR6	Hypoxia up-regulated protein 1 OS=Mus musculus GN=Hyou1 PE=1 SV=1	4.2042042	4	9	4	111.112	5.19	3100000	1300000	1600000	22.31
P58281	Dynamin-like 120 kDa protein, mitochondrial OS=Mus musculus GN=Opa1 PE=1 SV=1	30.416667	27	85	27	111.27	7.55	25000000	13000000	22000000	256.05
P80560	Receptor-type tyrosine-protein phosphatase N2 OS=Mus musculus GN=Ptprn2 PE=1 SV=	0.999001	1	1	1	111.427	5.95		600000		2.70
Q8C7X2	ER membrane protein complex subunit 1 OS=Mus musculus GN=Emc1 PE=1 SV=1	1.3039117	1	2	1	111.535	7.43	2000000		1500000	5.62
Q7TPS5	C2 domain-containing protein 5 OS=Mus musculus GN=C2cd5 PE=1 SV=2	1.1811024	1	2	1	111.593	5.78	1500000		780000	6.34
Q6PIC6	Sodium/potassium-transporting ATPase subunit alpha-3 OS=Mus musculus GN=Atp1a3 F	49.062192	47	297	30	111.62	5.41	440000000	110000000	240000000	988.27
Q60875	Rho guanine nucleotide exchange factor 2 OS=Mus musculus GN=Arhgef2 PE=1 SV=4	11.472081	10	14	10	111.905	7.25	4600000		2400000	41.76
Q6PIE5	Sodium/potassium-transporting ATPase subunit alpha-2 OS=Mus musculus GN=Atp1a2 F	42.843137	40	226	21	112.145	5.55	100000000	25000000	58000000	735.09
Q9ESZ8	General transcription factor II-I OS=Mus musculus GN=Gtf2i PE=1 SV=3	0.9018036	1	1	1	112.195	6.55	1300000			2.41
Q8BIJ6	Isoleucine--tRNA ligase, mitochondrial OS=Mus musculus GN=Iars2 PE=1 SV=1	5.4347826	5	9	5	112.732	6.81	3000000	840000	1700000	23.29
Q505D1	Serine/threonine-protein phosphatase 6 regulatory ankyrin repeat subunit A OS=Mus mus	1.5194682	1	2	1	112.826	6.27	840000	600000		5.10
Q8VDN2	Sodium/potassium-transporting ATPase subunit alpha-1 OS=Mus musculus GN=Atp1a1 F	41.055718	38	224	23	112.91	5.45	180000000	49000000	110000000	742.06
Q61330	Contactin-2 OS=Mus musculus GN=Cntn2 PE=1 SV=2	4.1346154	3	7	3	113.146	7.62	1700000	1500000	1900000	23.45
Q07409	Contactin-3 OS=Mus musculus GN=Cntn3 PE=1 SV=2	4.766537	3	4	3	113.161	6.23	500000	500000		10.10
Q921M4	Golgin subfamily A member 2 OS=Mus musculus GN=Golga2 PE=1 SV=3	2.3023023	2	3	2	113.209	5	1600000		1500000	8.82
P12960	Contactin-1 OS=Mus musculus GN=Cntn1 PE=1 SV=1	39.509804	31	118	31	113.317	6.16	41000000	30000000	38000000	367.79
Q9JMB8	Contactin-6 OS=Mus musculus GN=Cntn6 PE=1 SV=2	3.4046693	1	1	1	113.69	6.64	790000			3.64
Q61941	NAD(P) transhydrogenase, mitochondrial OS=Mus musculus GN=Nnt PE=1 SV=2	5.4327808	5	10	5	113.765	7.64	6800000	2300000	3200000	27.73
Q6NZN0	RNA-binding protein 26 OS=Mus musculus GN=Rbm26 PE=1 SV=2	1.284585	1	2	1	114.073	9.16	940000		630000	6.50
Q7TSF1	Desmoglein-1-beta OS=Mus musculus GN=Dsg1b PE=1 SV=1	2.9245283	3	6	3	114.382	4.84	2100000	650000	1900000	16.93
Q80TM6	R3H domain-containing protein 2 OS=Mus musculus GN=R3hdm2 PE=1 SV=2	2.0114943	1	1	1	114.512	8.91	180000			2.86
Q2PFD7	PH and SEC7 domain-containing protein 3 OS=Mus musculus GN=Psd3 PE=1 SV=2	5.978785	6	14	6	114.651	6.24	15000000	2400000	10000000	38.13

O55143	Sarcoplasmic/endoplasmic reticulum calcium ATPase 2 OS=Mus musculus GN=Atp2a2 P	22.796935	20	66	20	114.784	5.34	51000000	11000000	29000000	194.22
Q9WV27	Sodium/potassium-transporting ATPase subunit alpha-4 OS=Mus musculus GN=Atp1a4 F	11.046512	9	51	1	114.813	5.71	560000			150.80
Q9DBR7	Protein phosphatase 1 regulatory subunit 12A OS=Mus musculus GN=Ppp1r12a PE=1 S	9.5238095	8	12	7	114.927	5.49	1200000	400000	1800000	33.14
Q8VD75	Huntingtin-interacting protein 1 OS=Mus musculus GN=Hip1 PE=1 SV=2	0.9718173	1	1	1	115.13	5.43	970000			2.70
Q8K135	Dyslexia-associated protein KIAA0319-like protein OS=Mus musculus GN=Kiaa0319l PE=	1.4312977	1	1	1	115.24	6.16	1000000			2.51
P43406	Integrin alpha-V OS=Mus musculus GN=Itgav PE=1 SV=2	2.394636	2	5	2	115.287	5.63	2200000	1400000	1600000	13.23
Q6WQJ1	Sn1-specific diacylglycerol lipase alpha OS=Mus musculus GN=Dagla PE=1 SV=2	3.0651341	3	5	3	115.301	6.42	630000		600000	11.51
Q8K224	RNA cytidine acetyltransferase OS=Mus musculus GN=Nat10 PE=1 SV=1	1.4648438	1	2	1	115.346	8.32	380000		210000	4.89
Q8CFI0	E3 ubiquitin-protein ligase NEDD4-like OS=Mus musculus GN=Nedd4l PE=1 SV=2	2.7888446	3	6	2	115.347	6.15	1500000	590000	1200000	14.25
Q9EQZ6	Rap guanine nucleotide exchange factor 4 OS=Mus musculus GN=Rapgef4 PE=1 SV=1	7.4183976	7	12	7	115.418	6.92	3600000	740000	2400000	27.74
Q9QVP9	Protein-tyrosine kinase 2-beta OS=Mus musculus GN=Ptk2b PE=1 SV=2	6.1446977	6	12	6	115.72	6.35	3000000	600000	2400000	28.36
Q9Z0H8	CAP-Gly domain-containing linker protein 2 OS=Mus musculus GN=Clip2 PE=1 SV=2	10.983763	10	16	9	115.84	6.48	2700000		2100000	45.26
Q5F2E8	Serine/threonine-protein kinase TAO1 OS=Mus musculus GN=Taok1 PE=1 SV=1	4.1958042	4	6	3	115.977	7.55	1700000	340000	1100000	15.87
Q91YE6	Importin-9 OS=Mus musculus GN=Ipo9 PE=1 SV=3	1.0566763	1	1	1	115.978	4.81	1100000			2.83
Q5SYD0	Unconventional myosin-Id OS=Mus musculus GN=Myo1d PE=1 SV=1	1.3916501	1	1	1	116.007	9.41	690000			2.66
Q99MR1	PERQ amino acid-rich with GYF domain-containing protein 1 OS=Mus musculus GN=Gigy	4.1187739	2	2	2	116.167	5.39	1300000			6.14
Q8BUH8	Sentrin-specific protease 7 OS=Mus musculus GN=Senp7 PE=1 SV=1	1.1571842	1	1	1	116.273	6.52				2.85
P97434	Myosin phosphatase Rho-interacting protein OS=Mus musculus GN=Mprip PE=1 SV=2	5.7617188	3	4	3	116.337	6.21	940000		590000	11.38
Q60597	2-oxoglutarate dehydrogenase, mitochondrial OS=Mus musculus GN=Ogdh PE=1 SV=3	31.085044	26	76	26	116.375	6.83	16000000	13000000	24000000	208.46
Q64727	Vinculin OS=Mus musculus GN=Vcl PE=1 SV=4	2.3452158	2	2	2	116.644	6	1900000			4.91
Q80X50	Ubiquitin-associated protein 2-like OS=Mus musculus GN=Ubap2l PE=1 SV=1	4.0650407	3	4	3	116.728	7.11	2100000		1700000	12.49
O88532	Zinc finger RNA-binding protein OS=Mus musculus GN=Zfr PE=1 SV=2	11.173184	9	13	9	116.786	9.04	2700000		1700000	37.03
Q8CH25	SAFB-like transcription modulator OS=Mus musculus GN=Sltm PE=1 SV=1	9.3113482	5	6	5	116.85	7.72	1600000		1000000	20.18
Q8CGY8	UDP-N-acetylglucosamine--peptide N-acetylglucosaminyltransferase 110 kDa subunit OS	3.1548757	3	6	3	116.877	6.7	1300000		1700000	14.86
Q61136	Serine/threonine-protein kinase PRP4 homolog OS=Mus musculus GN=Prpf4b PE=1 SV=	1.1916584	1	2	1	116.904	10.23	2600000		2200000	6.93
P19246	Neurofilament heavy polypeptide OS=Mus musculus GN=Nefh PE=1 SV=3	19.541284	19	44	16	116.924	5.81	8200000	1300000	10000000	124.98
P33175	Kinesin heavy chain isoform 5A OS=Mus musculus GN=Kif5a PE=1 SV=3	7.0107108	6	10	1	116.946	5.94				34.27
Q8C5W0	Calmin OS=Mus musculus GN=Clmn PE=1 SV=2	1.0456274	1	1	1	117.155	5.03	1000000			2.58
Q8C129	Leucyl-cystinyl aminopeptidase OS=Mus musculus GN=Lnpep PE=1 SV=1	1.8536585	1	1	1	117.229	5.96			420000	0.00
Q80YA9	Connector enhancer of kinase suppressor of ras 2 OS=Mus musculus GN=Cnksr2 PE=1	6.6860465	5	11	5	117.323	6.79	4100000	880000	2600000	32.73
P27546	Microtubule-associated protein 4 OS=Mus musculus GN=Map4 PE=1 SV=3	12.711111	10	18	10	117.357	4.98	2500000	540000	1500000	48.54
Q569Z5	Probable ATP-dependent RNA helicase DDX46 OS=Mus musculus GN=Ddx46 PE=1 SV=	5.3294574	4	7	4	117.376	9.26	2200000		880000	20.92
P59759	MKL/myocardin-like protein 2 OS=Mus musculus GN=Mkl2 PE=1 SV=1	1.0185185	1	1	1	117.474	6.16	1100000			2.18
Q9CZU3	Superkiller viralicidic activity 2-like 2 OS=Mus musculus GN=Skiv2l2 PE=1 SV=1	1.3461538	1	1	1	117.561	6.4				2.80
Q02053	Ubiquitin-like modifier-activating enzyme 1 OS=Mus musculus GN=Uba1 PE=1 SV=1	15.500945	13	34	13	117.734	5.66	15000000	4400000	9600000	114.78
Q8C7R4	Ubiquitin-like modifier-activating enzyme 6 OS=Mus musculus GN=Uba6 PE=1 SV=1	1.2345679	1	1	1	117.891	6.11	310000			3.19
Q8CH09	SURP and G-patch domain-containing protein 2 OS=Mus musculus GN=Sugp2 PE=1 SV=	3.0927835	2	2	2	118.029	8.31	1400000			7.49
Q3UH68	LIM and calponin homology domains-containing protein 1 OS=Mus musculus GN=Limch1	2.8382214	2	2	2	118.123	5.48	360000		1500000	6.76
Q6NV83	U2 snRNP-associated SURP motif-containing protein OS=Mus musculus GN=U2surp PE=	3.4985423	2	3	2	118.188	8.47	2100000		2900000	10.33
Q80U40	RIMS-binding protein 2 OS=Mus musculus GN=Rimbp2 PE=1 SV=3	2.9850746	2	3	2	118.269	5.35	2500000		1400000	9.09
O70228	Probable phospholipid-transporting ATPase IIA OS=Mus musculus GN=Atp9a PE=1 SV=3	3.6294174	3	3	3	118.532	7.69	2000000			7.87
F8VPU2	FERM, RhoGEF and pleckstrin domain-containing protein 1 OS=Mus musculus GN=Farp1	9.351145	7	11	7	118.801	7.88	4300000	1400000	3300000	33.35
Q8BJ42	Disks large-associated protein 2 OS=Mus musculus GN=Dlgap2 PE=1 SV=2	8.687441	6	14	5	118.998	6.81	6100000		4400000	46.45
Q9JME5	AP-3 complex subunit beta-2 OS=Mus musculus GN=Ap3b2 PE=1 SV=2	17.92976	15	31	15	119.118	5.63	12000000	1600000	5700000	90.02
Q7TMC8	L-fucose kinase OS=Mus musculus GN=Fuk PE=1 SV=1	1.3761468	1	1	1	119.191	6.6	660000			3.13
Q8R4U7	Leucine zipper protein 1 OS=Mus musculus GN=Luzp1 PE=1 SV=2	1.3108614	1	2	1	119.239	7.99				6.74
Q9JKY5	Huntingtin-interacting protein 1-related protein OS=Mus musculus GN=Hip1r PE=1 SV=2	2.7153558	2	3	2	119.353	6.52	2000000		1800000	10.81
P13595	Neural cell adhesion molecule 1 OS=Mus musculus GN=Ncam1 PE=1 SV=3	29.237668	24	101	24	119.353	4.83	86000000	50000000	79000000	316.98

Q9EPL8	Importin-7 OS=Mus musculus GN=Ipo7 PE=1 SV=2		2.2157996	2	3	2	119.41	4.82	1400000		1200000	8.87
Q91V92	ATP-citrate synthase OS=Mus musculus GN=Acly PE=1 SV=1		6.59945	6	10	6	119.651	7.44	4600000	1700000	2400000	27.28
Q920B9	FACT complex subunit SPT16 OS=Mus musculus GN=Supt16h PE=1 SV=2		5.3486151	5	6	5	119.749	5.66	2600000		1600000	16.17
Q3V0C5	Ubiquitin carboxyl-terminal hydrolase 48 OS=Mus musculus GN=Usp48 PE=1 SV=2		1.6159696	1	1	1	120.554	6.25	230000			2.17
O55201	Transcription elongation factor SPT5 OS=Mus musculus GN=Supt5h PE=1 SV=1		4.3438078	2	3	2	120.589	5.05	1100000		690000	9.84
A2AWA9	Rab GTPase-activating protein 1 OS=Mus musculus GN=Rabgap1 PE=1 SV=1		1.1278195	1	3	1	120.722	5.25	1900000	240000	1200000	9.28
Q91Z67	SLIT-ROBO Rho GTPase-activating protein 2 OS=Mus musculus GN=Srgap2 PE=1 SV=2		4.6685341	3	4	3	120.723	6.64	1100000		1100000	12.39
Q9Z1R2	Large proline-rich protein BAG6 OS=Mus musculus GN=Bag6 PE=1 SV=1		2.1663778	2	3	2	120.962	5.71	370000		540000	6.29
Q91Z69	SLIT-ROBO Rho GTPase-activating protein 1 OS=Mus musculus GN=Srgap1 PE=1 SV=2		1.0357815	1	1	1	121.354	6.74	2100000			3.26
O88343	Electrogenic sodium bicarbonate cotransporter 1 OS=Mus musculus GN=Slc4a4 PE=1 SV		5.3753475	5	14	5	121.406	6.84	8300000	2400000	5500000	42.32
Q6R0H7	Guanine nucleotide-binding protein G(s) subunit alpha isoforms XLas OS=Mus musculus		9.4439541	9	26	8	121.429	4.81	18000000	7400000	14000000	66.79
Q91ZW3	SWI/SNF-related matrix-associated actin-dependent regulator of chromatin subfamily A m		4.2816365	3	5	3	121.55	8.15	2600000	280000	2200000	17.91
Q6A0A9	Constitutive coactivator of PPAR-gamma-like protein 1 OS=Mus musculus GN=FAM120A		6.205036	5	8	5	121.569	8.92	3700000		1900000	25.45
Q8BTI9	Phosphatidylinositol 4,5-bisphosphate 3-kinase catalytic subunit beta isoform OS=Mus mu		1.4097744	1	1	1	121.633	7.09	430000			2.20
Q9JL26	Formin-like protein 1 OS=Mus musculus GN=Fmnl1 PE=1 SV=1		1.2797075	1	1	1	121.983	5.82	870000			2.78
Q9Z1L5	Voltage-dependent calcium channel subunit alpha-2/delta-3 OS=Mus musculus GN=Cacr		5.4995417	4	4	4	122.7	5.73	2200000			14.26
Q6P5F9	Exportin-1 OS=Mus musculus GN=Xpo1 PE=1 SV=1		2.1475257	2	4	2	123.013	6.07	2800000		1900000	12.97
B2RUP2	Protein unc-13 homolog D OS=Mus musculus GN=Unc13d PE=1 SV=2		1.4746544	1	1	1	123.042	6.62			420000	3.01
Q8BPQ7	Small G protein signaling modulator 1 OS=Mus musculus GN=Sgsm1 PE=1 SV=2		1.0064044	1	1	1	123.118	5.66	630000			2.31
Q80TL1	Adenylate cyclase type 2 OS=Mus musculus GN=Adcy2 PE=1 SV=2		1.559633	1	1	1	123.19	8.31	570000			4.34
O88444	Adenylate cyclase type 1 OS=Mus musculus GN=Adcy1 PE=1 SV=2		1.431127	1	2	1	123.293	8.47	1000000		640000	4.42
P34152	Focal adhesion kinase 1 OS=Mus musculus GN=Ptk2 PE=1 SV=3		1.6513761	2	3	2	123.458	6.68	2400000		1700000	6.89
Q8BKC5	Importin-5 OS=Mus musculus GN=Ipo5 PE=1 SV=3		2.461258	2	2	2	123.511	4.93	930000		2200000	7.32
Q8CGF7	Transcription elongation regulator 1 OS=Mus musculus GN=Tcerg1 PE=1 SV=2		3.9090909	3	3	3	123.71	8.65	1100000			7.42
Q9EPK7	Exportin-7 OS=Mus musculus GN=Xpo7 PE=1 SV=3		2.299908	2	2	2	123.731	6.38	2400000	330000		5.00
Q9EPU0	Regulator of nonsense transcripts 1 OS=Mus musculus GN=Upf1 PE=1 SV=2		6.4946619	6	9	6	123.889	6.61	3400000		1900000	24.03
Q812A2	SLIT-ROBO Rho GTPase-activating protein 3 OS=Mus musculus GN=Srgap3 PE=1 SV=1		11.101001	9	15	9	124.341	6.67	5300000	640000	2600000	44.67
Q3UHD9	Arf-GAP with GTPase, ANK repeat and PH domain-containing protein 2 OS=Mus musculu		8.3473862	8	15	8	124.434	9.89	4000000	790000	2300000	43.89
O08532	Voltage-dependent calcium channel subunit alpha-2/delta-1 OS=Mus musculus GN=Cacr		17.860381	15	60	15	124.551	5.3	13000000	8600000	12000000	186.77
P59281	Rho GTPase-activating protein 39 OS=Mus musculus GN=Arhgap39 PE=1 SV=2		1.0840108	1	1	1	125.127	7.97	830000			3.05
Q8K394	Inactive phospholipase C-like protein 2 OS=Mus musculus GN=Plcl2 PE=1 SV=2		1.2411348	1	1	1	125.692	6.92	1700000			3.14
Q9Z2V5	Histone deacetylase 6 OS=Mus musculus GN=Hdac6 PE=1 SV=3		1.2184508	1	3	1	125.706	5.78	1400000	300000	970000	8.99
Q5DTL9	Sodium-driven chloride bicarbonate exchanger OS=Mus musculus GN=Slc4a10 PE=1 SV		14.400716	13	34	13	125.736	6.51	16000000	3000000	6900000	100.20
Q91V14	Solute carrier family 12 member 5 OS=Mus musculus GN=Slc12a5 PE=1 SV=2		19.683656	16	44	16	126.189	6.74	52000000	13000000	33000000	121.63
Q99P72	Reticulon-4 OS=Mus musculus GN=Rtn4 PE=1 SV=2		19.104991	15	42	15	126.535	4.54	19000000	5000000	15000000	128.67
Q80U78	Pumilio homolog 1 OS=Mus musculus GN=Pum1 PE=1 SV=2		0.9251472	1	1	1	126.541	6.86	1900000			2.20
Q3U1J4	DNA damage-binding protein 1 OS=Mus musculus GN=Ddb1 PE=1 SV=2		0.9649123	1	1	1	126.772	5.26	1800000			2.97
Q9QWY8	Arf-GAP with SH3 domain, ANK repeat and PH domain-containing protein 1 OS=Mus mus		4.6207498	4	6	4	127.342	7.64	2600000	390000	1700000	16.90
Q8K400	Syntaxin-binding protein 5 OS=Mus musculus GN=Stxbp5 PE=1 SV=3		8.9409722	6	9	5	127.57	7.21	2700000	460000	860000	24.40
Q99MI1	ELKS/Rab6-interacting/CAST family member 1 OS=Mus musculus GN=Erc1 PE=1 SV=1		4.6428571	5	9	1	128.252	5.87	500000			24.93
Q80U19	Disheveled-associated activator of morphogenesis 2 OS=Mus musculus GN=Daam2 PE=		1.5246637	2	2	2	128.289	6.92	590000		320000	3.98
B2RXC1	Trafficking protein particle complex subunit 11 OS=Mus musculus GN=Trappc11 PE=1 SV		1.059135	1	1	1	128.314	7.58	1200000			1.99
Q6A4J8	Ubiquitin carboxyl-terminal hydrolase 7 OS=Mus musculus GN=Usp7 PE=1 SV=1		3.8077969	4	7	4	128.393	5.5	1500000		2100000	17.43
Q8R0G9	Nuclear pore complex protein Nup133 OS=Mus musculus GN=Nup133 PE=1 SV=2		1.2987013	1	1	1	128.539	5.2	620000			2.70
Q9EP53	Hamartin OS=Mus musculus GN=Tsc1 PE=1 SV=1		1.1197244	1	1	1	128.666	6.37	450000			2.96
P28660	Nck-associated protein 1 OS=Mus musculus GN=Nckap1 PE=1 SV=2		15.514184	17	33	17	128.7	6.62	12000000	2700000	8200000	85.11
Q3TES0	IQ motif and SEC7 domain-containing protein 3 OS=Mus musculus GN=Iqsec3 PE=1 SV=		5.1882845	4	5	3	129.038	6.19	1400000		960000	13.72
Q01149	Collagen alpha-2(I) chain OS=Mus musculus GN=Col1a2 PE=1 SV=2		6.4868805	6	19	6	129.478	9.19	38000000	28000000	16000000	57.40

Q05920	Pyruvate carboxylase, mitochondrial OS=Mus musculus GN=Pc PE=1 SV=1	17.911715	17	31	17	129.602	6.71	8200000	4100000	7800000	89.26
Q80VP0	Tectonin beta-propeller repeat-containing protein 1 OS=Mus musculus GN=Tecpr1 PE=1	6.6895369	5	12	5	130.184	6.48	1500000	1100000	2900000	40.25
Q6PHS9	Voltage-dependent calcium channel subunit alpha-2/delta-2 OS=Mus musculus GN=Cacr	5.1993068	5	11	5	130.303	5.66	1500000	900000	1100000	30.70
P55012	Solute carrier family 12 member 2 OS=Mus musculus GN=Slc12a2 PE=1 SV=2	0.9958506	1	1	1	130.95	7.33	2300000			2.45
Q9Z0U1	Tight junction protein ZO-2 OS=Mus musculus GN=Tjp2 PE=1 SV=2	1.9708655	2	3	2	131.2	6.79	480000		620000	7.19
P70704	Phospholipid-transporting ATPase IA OS=Mus musculus GN=Atp8a1 PE=1 SV=2	12.972509	13	27	13	131.33	6.84	15000000	3100000	6400000	81.31
Q7TQG1	Pleckstrin homology domain-containing family A member 6 OS=Mus musculus GN=Plekha	2.4722933	2	3	2	131.346	8.97	1500000		810000	8.10
Q68FH0	Plakophilin-4 OS=Mus musculus GN=Pkp4 PE=1 SV=1	3.697479	4	5	2	131.47	8.94	300000		600000	10.99
Q3UHC7	Disabled homolog 2-interacting protein OS=Mus musculus GN=Dab2ip PE=1 SV=1	1.8502944	2	3	1	131.644	8.72	2700000		1900000	6.99
Q5DQR4	Syntaxin-binding protein 5-like OS=Mus musculus GN=Stxbp5l PE=1 SV=1	3.8818565	2	3	1	131.76	6.86				7.45
Q3UVX5	Metabotropic glutamate receptor 5 OS=Mus musculus GN=Grm5 PE=1 SV=2	6.6500416	5	9	5	131.78	7.84	2600000	560000	2100000	27.61
Q3TVA9	Coiled-coil domain-containing protein 136 OS=Mus musculus GN=Ccdc136 PE=1 SV=2	8.5387324	6	11	6	131.847	4.84	1600000	590000	1400000	34.05
Q3UTJ2	Sorbin and SH3 domain-containing protein 2 OS=Mus musculus GN=Sorbs2 PE=1 SV=2	6.6101695	6	8	5	132.266	8.53	1400000		400000	20.34
Q68FE6	Protein FAM65A OS=Mus musculus GN=Fam65a PE=1 SV=2	1.2264922	1	1	1	132.27	5.88	370000			3.89
Q9EPE9	Manganese-transporting ATPase 13A1 OS=Mus musculus GN=Atp13a1 PE=1 SV=2	0.75	1	1	1	132.303	8.03	910000			2.19
Q9R0K7	Plasma membrane calcium-transporting ATPase 2 OS=Mus musculus GN=Atp2b2 PE=1	31.469115	32	124	22	132.503	5.96	76000000	15000000	53000000	414.94
Q6PDG5	SWI/SNF complex subunit SMARCC2 OS=Mus musculus GN=Smarcc2 PE=1 SV=2	13.849959	13	17	13	132.522	5.59	3800000		1500000	51.56
Q6Q477	Plasma membrane calcium-transporting ATPase 4 OS=Mus musculus GN=Atp2b4 PE=1	21.493776	21	87	11	132.984	6.13	38000000	11000000	21000000	311.74
P97772	Metabotropic glutamate receptor 1 OS=Mus musculus GN=Grm1 PE=1 SV=2	1.2510425	1	2	1	133.127	6.86	1300000			5.35
Q6GQT9	Nodal modulator 1 OS=Mus musculus GN=Nomo1 PE=1 SV=1	7.660626	7	14	7	133.336	6.09	3600000	2100000	2500000	41.85
P60469	Liprin-alpha-3 OS=Mus musculus GN=Ppfia3 PE=1 SV=2	14.740369	13	28	13	133.344	5.64	4900000	890000	4400000	80.75
Q3UPL0	Protein transport protein Sec31A OS=Mus musculus GN=Sec31a PE=1 SV=2	0.7317073	1	2	1	133.486	6.76	1800000		1100000	4.43
Q61543	Golgi apparatus protein 1 OS=Mus musculus GN=Glg1 PE=1 SV=1	12.425532	12	26	12	133.646	6.84	3000000	2200000	2200000	71.73
Q8BMJ2	Leucine--tRNA ligase, cytoplasmic OS=Mus musculus GN=Lars PE=1 SV=2	5.7724958	5	7	5	134.106	7.05	1500000		920000	19.89
Q08460	Calcium-activated potassium channel subunit alpha-1 OS=Mus musculus GN=Kcnma1 PE	2.4813896	2	3	2	134.31	7.15	2100000		1400000	9.41
Q8C419	Probable G-protein coupled receptor 158 OS=Mus musculus GN=Gpr158 PE=1 SV=2	14.083333	9	18	9	134.341	8.09	3700000	450000	2800000	54.63
Q8CDG3	Deubiquitinating protein VCIP135 OS=Mus musculus GN=Vcpip1 PE=1 SV=1	3.6885246	3	4	3	134.418	7.17	1400000		140000	10.52
Q148V7	LisH domain and HEAT repeat-containing protein KIAA1468 OS=Mus musculus GN=Kiaa	1.0690789	1	2	1	134.502	5.34	620000		1100000	6.13
G5E829	Plasma membrane calcium-transporting ATPase 1 OS=Mus musculus GN=Atp2b1 PE=1	24.672131	26	105	14	134.662	5.91	67000000	15000000	41000000	352.81
Q9QWI6	SRC kinase signaling inhibitor 1 OS=Mus musculus GN=Srcin1 PE=1 SV=2	21.44	19	41	19	134.776	9.32	14000000	2300000	9500000	125.44
O35954	Membrane-associated phosphatidylinositol transfer protein 1 OS=Mus musculus GN=Pitpr	3.0571199	2	3	2	134.855	6.06	860000		930000	9.74
O35927	Catenin delta-2 OS=Mus musculus GN=Ctnnd2 PE=1 SV=1	16.359262	15	29	13	134.916	7.65	6300000	770000	8000000	80.13
P70232	Neural cell adhesion molecule L1-like protein OS=Mus musculus GN=Chl1 PE=1 SV=2	5.3763441	4	7	4	134.99	5.57	1200000	1100000	1200000	21.26
O54774	AP-3 complex subunit delta-1 OS=Mus musculus GN=Ap3d1 PE=1 SV=1	7.9232694	8	17	8	134.996	7.37	5800000	780000	5200000	56.17
Q80VC9	Calmodulin-regulated spectrin-associated protein 3 OS=Mus musculus GN=Camsap3 PE	1.1182109	1	1	1	135.091	8.43	520000			2.78
P16283	Anion exchange protein 3 OS=Mus musculus GN=Slc4a3 PE=1 SV=2	2.607987	2	5	2	135.288	6.51	890000		590000	13.20
Q921M3	Splicing factor 3B subunit 3 OS=Mus musculus GN=Sf3b3 PE=1 SV=1	3.2046015	3	5	3	135.465	5.26	4600000		2600000	13.05
P70392	Ras-specific guanine nucleotide-releasing factor 2 OS=Mus musculus GN=Rasgrf2 PE=1	2.0185029	2	4	2	135.581	7.85	1100000		770000	11.26
Q6ZQ38	Cullin-associated NEDD8-dissociated protein 1 OS=Mus musculus GN=Cand1 PE=1 SV=	9.5121951	11	20	11	136.245	5.78	11000000	1900000	5500000	55.98
O70305	Ataxin-2 OS=Mus musculus GN=Atxn2 PE=1 SV=1	1.1673152	1	1	1	136.4	9.55			750000	2.67
P10493	Nidogen-1 OS=Mus musculus GN=Nid1 PE=1 SV=2	1.0441767	1	2	1	136.45	5.44		660000	740000	5.47
Q64455	Receptor-type tyrosine-protein phosphatase eta OS=Mus musculus GN=Ptprj PE=1 SV=2	1.3731826	1	1	1	136.683	5.57			1100000	2.37
P55066	Neurocan core protein OS=Mus musculus GN=Ncan PE=1 SV=1	13.958991	13	31	13	137.114	5.72	11000000	7700000	11000000	84.00
Q05D44	Eukaryotic translation initiation factor 5B OS=Mus musculus GN=Eif5b PE=1 SV=2	2.3848684	2	3	2	137.532	5.59	1200000		1300000	10.73
Q810U3	Neurofascin OS=Mus musculus GN=Nfasc PE=1 SV=1	17.258065	17	56	17	137.889	6.19	35000000	10000000	22000000	169.26
P11087	Collagen alpha-1(I) chain OS=Mus musculus GN=Col1a1 PE=1 SV=4	7.7770131	8	25	8	137.948	5.85	62000000	57000000	39000000	84.14
Q9Z1B3	1-phosphatidylinositol 4,5-bisphosphate phosphodiesterase beta-1 OS=Mus musculus GN	23.519737	23	62	23	138.309	6.13	16000000	5600000	14000000	194.66
Q8CIE6	Coatomer subunit alpha OS=Mus musculus GN=Copa PE=1 SV=2	5.9640523	6	6	6	138.344	7.65	3900000			17.13

P04258	SWISS-PROT:P04258 (Bos taurus) Similar to Collagen alpha 1(III) chain	x	2.7967258	3	3	3	138.354	6.43		4800000		9.75
Q810U4	Neuronal cell adhesion molecule OS=Mus musculus GN=Nrcam PE=1 SV=2		17.595541	19	49	19	138.435	5.91	9300000	8700000	12000000	149.56
Q8BIZ1	Ankyrin repeat and sterile alpha motif domain-containing protein 1B OS=Mus musculus G		2.7799841	3	7	3	138.961	6.29	11000000	3000000	7800000	19.76
Q69ZW3	EH domain-binding protein 1 OS=Mus musculus GN=Ehbp1 PE=1 SV=3		4.6303818	3	4	3	139.019	5.38	570000		720000	12.24
Q6ZQ29	Serine/threonine-protein kinase TAO2 OS=Mus musculus GN=Taok2 PE=1 SV=3		2.0967742	3	5	2	139.211	7.06	1100000		810000	10.94
Q5SV85	Synergin gamma OS=Mus musculus GN=Synrg PE=1 SV=1		1.914242	2	2	2	139.528	5.03		1000000	360000	6.00
Q64514	Tripeptidyl-peptidase 2 OS=Mus musculus GN=Tpp2 PE=1 SV=3		9.1917591	8	17	8	139.791	6.58	4500000	1900000	4700000	55.86
Q9Z1Q9	Valine--tRNA ligase OS=Mus musculus GN=Vars PE=1 SV=1		8.3135392	8	16	8	140.127	7.77	7900000	910000	4300000	48.75
P97820	Mitogen-activated protein kinase kinase kinase kinase 4 OS=Mus musculus GN=Map4k4		3.163017	3	4	1	140.515	7.47	210000			13.39
Q8BRT1	CLIP-associating protein 2 OS=Mus musculus GN=Clasp2 PE=1 SV=1		8.1648523	7	13	7	140.652	8.63	5700000	1000000	3600000	44.40
Q6ZPJ3	(E3-independent) E2 ubiquitin-conjugating enzyme UBE2O OS=Mus musculus GN=Ube2		2.3291925	2	2	2	140.746	5.06	1900000			5.90
Q9WVQ1	Membrane-associated guanylate kinase, WW and PDZ domain-containing protein 2 OS=M		2.745098	3	4	3	140.831	6.19	870000		1400000	9.10
Q6ZPZ3	Zinc finger CCCH domain-containing protein 4 OS=Mus musculus GN=Zc3h4 PE=1 SV=2		3.7576687	3	6	3	140.88	6.27	730000		390000	19.19
P11627	Neural cell adhesion molecule L1 OS=Mus musculus GN=L1cam PE=1 SV=1		11.746032	11	26	11	140.881	5.97	7300000	8200000	6700000	71.80
Q3V3V9	Capping protein, Arp2/3 and myosin-I linker protein 2 OS=Mus musculus GN=Carmil2 PE		1.3888889	1	2	1	141.284	7.18	320000		300000	5.21
O54988	STE20-like serine/threonine-protein kinase OS=Mus musculus GN=Slk PE=1 SV=2		10.624493	8	13	8	141.37	5.14	1400000	760000	1900000	35.90
Q9CW03	Structural maintenance of chromosomes protein 3 OS=Mus musculus GN=Smc3 PE=1 S\		2.7115859	2	3	2	141.468	7.18	630000		180000	11.71
O08788	Dynactin subunit 1 OS=Mus musculus GN=Dctn1 PE=1 SV=3		17.408275	18	45	18	141.588	5.9	10000000	2100000	7600000	139.67
Q8VDJ3	Vigilin OS=Mus musculus GN=Hdlbp PE=1 SV=1		5.9936909	6	14	6	141.655	6.87	3300000	470000	1600000	38.00
Q6PAJ1	Breakpoint cluster region protein OS=Mus musculus GN=Bcr PE=1 SV=3		1.5748031	1	1	1	142.982	6.92	950000			3.17
Q62417	Sorbin and SH3 domain-containing protein 1 OS=Mus musculus GN=Sorbs1 PE=1 SV=2		5.1937984	5	8	4	142.982	8.25	1900000		890000	19.62
Q9CU62	Structural maintenance of chromosomes protein 1A OS=Mus musculus GN=Smc1a PE=1		0.973236	1	1	1	143.146	7.64	550000			2.19
Q8BSS9	Liprin-alpha-2 OS=Mus musculus GN=Ppfia2 PE=1 SV=2		6.2848051	6	10	6	143.146	6.11	1500000	280000	1800000	27.04
Q99KY4	Cyclin-G-associated kinase OS=Mus musculus GN=Gak PE=1 SV=2		1.9923372	2	4	2	143.55	5.73	1900000		1200000	10.60
P27671	Ras-specific guanine nucleotide-releasing factor 1 OS=Mus musculus GN=Rasgrf1 PE=1		1.0301109	1	1	1	144.011	7.17	1300000			2.81
Q8BU30	Isoleucine--tRNA ligase, cytoplasmic OS=Mus musculus GN=Iars PE=1 SV=2		1.2678288	1	1	1	144.179	6.55	2300000			2.91
Q80YE4	Serine/threonine-protein kinase LMTK1 OS=Mus musculus GN=Aatk PE=1 SV=1		1.3919414	1	2	1	144.517	4.55	920000		470000	5.95
Q5SUR0	Phosphoribosylformylglycinamidine synthase OS=Mus musculus GN=Pfas PE=1 SV=1		0.8975318	1	1	1	144.538	5.67	910000			1.86
Q61137	Astrotactin-1 OS=Mus musculus GN=Astn1 PE=1 SV=4		6.5284178	5	11	5	144.791	5.21	1400000	2500000	2200000	34.87
E9PZ19	Protein turtle homolog B OS=Mus musculus GN=Igsf9b PE=1 SV=1		2.1084337	2	2	2	144.86	6.7	810000			4.81
A2AFR3	FERM and PDZ domain-containing protein 4 OS=Mus musculus GN=Frmpd4 PE=1 SV=1		1.5151515	1	1	1	144.887	5.25				2.63
Q7TMB8	Cytoplasmic FMR1-interacting protein 1 OS=Mus musculus GN=Cyfip1 PE=1 SV=1		11.492418	13	24	4	145.148	6.9	3700000	260000	2100000	58.82
Q6PGL7	WASH complex subunit FAM21 OS=Mus musculus GN=Fam21 PE=1 SV=1		3.1484258	3	3	3	145.224	4.77	2000000		1600000	8.05
Q5SQX6	Cytoplasmic FMR1-interacting protein 2 OS=Mus musculus GN=Cyfip2 PE=1 SV=2		18.11652	21	47	12	145.565	7.05	13000000	2300000	8000000	120.93
Q99NB9	Splicing factor 3B subunit 1 OS=Mus musculus GN=Sf3b1 PE=1 SV=1		4.0644172	4	9	4	145.724	7.09	3900000	310000	2900000	26.11
Q58A65	C-Jun-amino-terminal kinase-interacting protein 4 OS=Mus musculus GN=Spag9 PE=1 S		3.9364118	4	6	4	146.129	5.15	1700000		1500000	17.19
Q64331	Unconventional myosin-VI OS=Mus musculus GN=Myo6 PE=1 SV=1		5.2173913	5	8	5	146.317	8.85	1000000		900000	26.43
Q61527	Receptor tyrosine-protein kinase erbB-4 OS=Mus musculus GN=Erbb4 PE=1 SV=5		0.6880734	1	1	1	146.759	6.38				2.58
Q9JM52	Misshapen-like kinase 1 OS=Mus musculus GN=Mink1 PE=1 SV=3		6.8042813	8	11	6	147.203	7.44	2400000		1400000	28.39
Q9ESN9	C-Jun-amino-terminal kinase-interacting protein 3 OS=Mus musculus GN=Mapk8ip3 PE=		1.2715034	1	1	1	147.469	5.45			680000	3.26
Q6ZPQ6	Membrane-associated phosphatidylinositol transfer protein 2 OS=Mus musculus GN=Pitp		7.6404494	7	13	7	147.942	7.93	7000000	1600000	4700000	39.01
Q9CPW0	Contactin-associated protein-like 2 OS=Mus musculus GN=Cntnap2 PE=1 SV=2		8.7087087	10	19	10	148.103	6.77	3500000	1700000	2000000	50.18
F6SEU4	Ras/Rap GTPase-activating protein SynGAP OS=Mus musculus GN=Syngap1 PE=1 SV=		20.746269	23	52	22	148.145	8.98	18000000	1600000	14000000	153.78
Q80TK0	Uncharacterized protein KIAA1107 OS=Mus musculus GN=Kiaa1107 PE=1 SV=5		7.669832	7	11	7	148.649	6.51	1400000		1400000	29.18
Q8K4Q0	Regulatory-associated protein of mTOR OS=Mus musculus GN=Rptor PE=1 SV=1		2.8464419	3	4	3	149.375	6.87	1800000		1200000	10.56
O70133	ATP-dependent RNA helicase A OS=Mus musculus GN=Dhx9 PE=1 SV=2		12.608696	14	27	14	149.381	6.83	16000000	2500000	7700000	85.95
Q7TT37	Elongator complex protein 1 OS=Mus musculus GN=Ikbkap PE=1 SV=2		2.3255814	2	4	2	149.489	6	1200000	210000	490000	12.39
Q8BYI9	Tenascin-R OS=Mus musculus GN=Tnr PE=1 SV=2		31.07511	35	117	35	149.495	4.94	28000000	14000000	35000000	353.25

Q62077	1-phosphatidylinositol 4,5-bisphosphate phosphodiesterase gamma-1 OS=Mus musculus	2.2273425	2	2	2	149.573	5.8	1200000		1000000	5.39
Q6A026	Sister chromatid cohesion protein PDS5 homolog A OS=Mus musculus GN=Pds5a PE=1	1.2012012	1	1	1	150.232	7.85	340000			3.23
P83510	Traf2 and NCK-interacting protein kinase OS=Mus musculus GN=Tnik PE=1 SV=2	4.30839	4	7	2	150.274	7.27	3300000		680000	21.85
Q6P9K8	Caskin-1 OS=Mus musculus GN=Caskin1 PE=1 SV=2	13.207547	12	21	12	150.403	9.17	6200000	1000000	4900000	68.17
Q9JIX8	Apoptotic chromatin condensation inducer in the nucleus OS=Mus musculus GN=Acin1 P	7.7727952	7	13	7	150.629	5.91	1900000		1900000	42.29
Q5XJV6	Serine/threonine-protein kinase LMTK3 OS=Mus musculus GN=Lmtk3 PE=1 SV=1	0.6320225	1	1	1	150.798	4.88	1300000			2.05
P51830	Adenylate cyclase type 9 OS=Mus musculus GN=Adcy9 PE=1 SV=1	3.8433112	4	5	4	150.857	7.21	1800000		1500000	13.75
Q7TPV4	Myb-binding protein 1A OS=Mus musculus GN=Mybbp1a PE=1 SV=2	4.6130952	4	7	4	151.942	8.95	830000		640000	19.81
Q3UJB9	Enhancer of mRNA-decapping protein 4 OS=Mus musculus GN=Edc4 PE=1 SV=2	1.0668563	1	1	1	152.389	5.78			610000	2.61
Q8BMG7	Rab3 GTPase-activating protein non-catalytic subunit OS=Mus musculus GN=Rab3gap2	1.317716	2	4	2	152.438	6.23	2000000		1300000	9.28
Q61595	Kinectin OS=Mus musculus GN=Ktn1 PE=1 SV=1	4.6721929	6	8	6	152.498	5.86	2000000		1100000	24.83
Q80TJ1	Calcium-dependent secretion activator 1 OS=Mus musculus GN=Cadps PE=1 SV=3	23.02583	21	46	21	153.016	5.74	30000000	3000000	17000000	127.83
O88322	Nidogen-2 OS=Mus musculus GN=Nid2 PE=1 SV=2	3.7776194	3	5	3	153.816	5.38	2200000	1400000	3200000	15.71
O35099	Mitogen-activated protein kinase kinase kinase 5 OS=Mus musculus GN=Map3k5 PE=1 S	0.5072464	1	3	1	154.414	5.78	4900000	1700000	3300000	6.70
Q8BZH4	Pogo transposable element with ZNF domain OS=Mus musculus GN=Pogz PE=1 SV=2	1.8452803	2	2	2	154.812	7.52	710000			6.14
Q99P88	Nuclear pore complex protein Nup155 OS=Mus musculus GN=Nup155 PE=1 SV=1	0.9345794	1	1	1	155.019	6.15			650000	3.29
Q9R0I7	YLP motif-containing protein 1 OS=Mus musculus GN=Ylpm1 PE=2 SV=2	0.7936508	1	1	1	155.032	6.65	1300000			2.37
P15208	Insulin receptor OS=Mus musculus GN=Insr PE=1 SV=2	2.1137026	2	3	2	155.51	5.95	370000		670000	8.81
Q922J3	CAP-Gly domain-containing linker protein 1 OS=Mus musculus GN=Clip1 PE=1 SV=1	10.424155	11	20	10	155.718	5.24	3300000	510000	2500000	63.88
O54991	Contactin-associated protein 1 OS=Mus musculus GN=Cntnap1 PE=1 SV=2	4.9819495	6	11	6	156.212	7.06	6300000	2000000	2500000	27.34
Q6PB66	Leucine-rich PPR motif-containing protein, mitochondrial OS=Mus musculus GN=Lrpprc P	5.8908046	8	16	8	156.516	6.83	2700000	1100000	2700000	40.60
Q9WU60	Attractin OS=Mus musculus GN=Atrn PE=1 SV=3	1.8207283	2	2	2	157.954	7.27			370000	4.08
Q5SW75	Protein phosphatase Slingshot homolog 2 OS=Mus musculus GN=Ssh2 PE=1 SV=2	0.8432888	1	1	1	158.13	5.67	1700000			2.22
Q80Z38	SH3 and multiple ankyrin repeat domains protein 2 OS=Mus musculus GN=Shank2 PE=1	11.788618	11	23	11	158.871	7.18	6500000		4400000	70.29
Q9Z0J4	Nitric oxide synthase, brain OS=Mus musculus GN=Nos1 PE=1 SV=1	0.9797061	1	1	1	160.371	7.15	560000			3.11
P61406	Telomerase-binding protein EST1A OS=Mus musculus GN=Smg6 PE=1 SV=1	1.1283498	1	1	1	160.396	6.77				2.84
P70336	Rho-associated protein kinase 2 OS=Mus musculus GN=Rock2 PE=1 SV=1	10.302594	13	22	13	160.485	5.99	5600000	540000	5000000	57.75
P02463	Collagen alpha-1(IV) chain OS=Mus musculus GN=Col4a1 PE=1 SV=4	0.8987418	1	2	1	160.579	8.24	1700000		1200000	5.24
Q7TPM1	Protein PRRC2B OS=Mus musculus GN=Prrc2b PE=1 SV=1	3.5666218	3	3	3	160.814	8.16	250000			8.77
Q8BL66	Early endosome antigen 1 OS=Mus musculus GN=Eea1 PE=1 SV=2	6.2367116	8	11	8	160.817	5.77	1300000		1600000	29.06
B2RR83	Probable ATP-dependent RNA helicase YTHDC2 OS=Mus musculus GN=Ythdc2 PE=1 S	1.1072664	1	1	1	160.99	8.51	490000			3.67
Q80TR1	Adhesion G protein-coupled receptor L1 OS=Mus musculus GN=Adgrl1 PE=1 SV=2	6.4120055	7	13	7	161.583	6.48	2600000	3000000	3100000	42.29
Q5DU25	IQ motif and SEC7 domain-containing protein 2 OS=Mus musculus GN=Iqsec2 PE=1 SV=	14.749662	16	25	13	161.687	8.56	5900000		4000000	75.97
Q69ZH9	Rho GTPase-activating protein 23 OS=Mus musculus GN=Arhgap23 PE=1 SV=2	1.0788941	1	1	1	161.732	8.98				3.03
A2RSJ4	UHRF1-binding protein 1-like OS=Mus musculus GN=Uhrf1bp1l PE=1 SV=2	1.0981469	1	1	1	161.838	6.61	450000			1.98
P23116	Eukaryotic translation initiation factor 3 subunit A OS=Mus musculus GN=Eif3a PE=1 SV=	5.8779762	8	13	8	161.838	6.77	3300000		1900000	32.54
Q6RHR9	Membrane-associated guanylate kinase, WW and PDZ domain-containing protein 1 OS=M	2.8552005	2	2	2	161.876	7.36				6.91
Q6PAR5	GTPase-activating protein and VPS9 domain-containing protein 1 OS=Mus musculus GN=	2.8806584	2	3	2	162.3	5.19	1600000		850000	9.63
Q99M80	Receptor-type tyrosine-protein phosphatase T OS=Mus musculus GN=Ptprt PE=1 SV=2	0.7565337	1	1	1	162.909	6.84	840000			2.49
Q99NE5	Regulating synaptic membrane exocytosis protein 1 OS=Mus musculus GN=Rims1 PE=1	7.2453862	7	13	7	163.061	9.42	4400000	270000	4300000	38.92
Q920I9	WD repeat-containing protein 7 OS=Mus musculus GN=Wdr7 PE=1 SV=3	9.8723976	12	25	12	163.345	6.9	7300000	1100000	4100000	69.49
A2AP18	1-phosphatidylinositol 4,5-bisphosphate phosphodiesterase eta-2 OS=Mus musculus GN=	0.9327115	1	1	1	164.195	7.49			930000	3.24
Q8C1B1	Calmodulin-regulated spectrin-associated protein 2 OS=Mus musculus GN=Camsap2 PE	0.9582478	1	1	1	164.23	6.86				1.92
Q4VA53	Sister chromatid cohesion protein PDS5 homolog B OS=Mus musculus GN=Pds5b PE=1	1.3831259	1	2	1	164.315	8.5	1700000		890000	7.25
Q69ZA1	Cyclin-dependent kinase 13 OS=Mus musculus GN=Cdk13 PE=1 SV=3	1.0589014	1	1	1	164.452	9.69	190000			2.86
Q8BWT5	Disco-interacting protein 2 homolog A OS=Mus musculus GN=Dip2a PE=1 SV=3	0.9192383	1	2	1	165.149	7.61	1400000		500000	5.68
P35436	Glutamate receptor ionotropic, NMDA 2A OS=Mus musculus GN=Grin2a PE=1 SV=2	1.2295082	2	3	2	165.316	7.01	2400000		1200000	7.13
Q61838	Pregnancy zone protein OS=Mus musculus GN=Pzp PE=1 SV=3	0.6020067	1	2	1	165.748	6.68	1600000	720000		4.05

Q01097	Glutamate receptor ionotropic, NMDA 2B OS=Mus musculus GN=Grin2b PE=1 SV=3	7.219973	9	16	9	165.853	6.87	4600000		3300000	40.75
Q9CS84	Neurexin-1 OS=Mus musculus GN=Nrxn1 PE=1 SV=3	8.9828269	11	18	10	166.063	6.09	6900000	3600000	5700000	49.07
Q8CHG7	Rap guanine nucleotide exchange factor 2 OS=Mus musculus GN=Rapgef2 PE=1 SV=2	1.8048128	2	3	2	166.311	6.61	1000000		490000	8.04
Q80TE7	Leucine-rich repeat-containing protein 7 OS=Mus musculus GN=Lrrc7 PE=1 SV=2	9.7986577	10	15	10	166.796	6.61	4200000		3200000	43.23
P55937	Golgin subfamily A member 3 OS=Mus musculus GN=Golga3 PE=1 SV=3	2.8244788	3	6	3	167.118	5.4	510000		460000	17.62
P08122	Collagen alpha-2(IV) chain OS=Mus musculus GN=Col4a2 PE=1 SV=4	1.6988869	2	3	2	167.22	8.48	4800000		2100000	7.55
Q9JL04	Formin-2 OS=Mus musculus GN=Fmn2 PE=1 SV=2	3.2319392	3	7	3	167.282	5.48	1000000	290000	1200000	23.62
Q80TV8	CLIP-associating protein 1 OS=Mus musculus GN=Clasp1 PE=1 SV=2	3.3224756	3	3	3	169.123	9.03	1200000		1600000	8.65
Q8CGM1	Adhesion G protein-coupled receptor B2 OS=Mus musculus GN=Adgrb2 PE=1 SV=2	1.6015375	2	2	2	169.753	7.36	1600000	300000		4.10
Q8CGC7	Bifunctional glutamate/proline--tRNA ligase OS=Mus musculus GN=Eprs PE=1 SV=4	5.8201058	7	17	7	169.972	7.66	3400000	730000	1900000	52.53
Q91YM2	Rho GTPase-activating protein 35 OS=Mus musculus GN=Arhgap35 PE=1 SV=3	3.4022682	4	7	4	170.285	6.61	2300000	320000	1400000	18.89
Q9Z277	Tyrosine-protein kinase BAZ1B OS=Mus musculus GN=Baz1b PE=1 SV=2	2.2988506	2	2	2	170.544	8.6	1200000			5.96
Q80U49	Centrosomal protein of 170 kDa protein B OS=Mus musculus GN=Cep170b PE=1 SV=2	6.0355781	7	12	5	170.718	6.87	1200000		590000	36.31
Q6ZPY7	Lysine-specific demethylase 3B OS=Mus musculus GN=Kdm3b PE=1 SV=2	0.7682458	1	1	1	170.768	6.92				2.12
Q80TS3	Adhesion G protein-coupled receptor L3 OS=Mus musculus GN=Adgrl3 PE=1 SV=3	6.7013663	9	18	9	170.971	6.71	3800000	3600000	4800000	49.45
Q3UH60	Disco-interacting protein 2 homolog B OS=Mus musculus GN=Dip2b PE=1 SV=1	2.5412961	3	4	3	171.017	8.09	1800000		1600000	12.01
Q80ZF8	Adhesion G protein-coupled receptor B3 OS=Mus musculus GN=Adgrb3 PE=1 SV=2	1.9710907	2	2	2	171.202	7.02	1100000			5.35
P97393	Rho GTPase-activating protein 5 OS=Mus musculus GN=Arhgap5 PE=1 SV=2	1.8654231	2	2	2	172.004	6.34	1400000			6.05
Q8R4H2	Rho guanine nucleotide exchange factor 12 OS=Mus musculus GN=Arhgef12 PE=1 SV=2	4.4718082	5	7	5	172.242	5.74	440000		1000000	17.58
Q8CHC4	Synaptojanin-1 OS=Mus musculus GN=Synj1 PE=1 SV=3	11.689962	16	36	16	172.509	6.89	16000000	4200000	8300000	118.38
Q9EQZ7	Regulating synaptic membrane exocytosis protein 2 OS=Mus musculus GN=Rims2 PE=1	1.7647059	2	2	2	172.757	9.25	430000			5.63
Q99PL5	Ribosome-binding protein 1 OS=Mus musculus GN=Rrbp1 PE=1 SV=2	1.9937695	3	5	3	172.776	9.33	1700000		1300000	14.40
Q6P9K9	Neurexin-3 OS=Mus musculus GN=Nrxn3 PE=1 SV=2	4.6467218	6	17	5	173.318	6.14	4000000	1700000	3600000	55.04
Q80XI3	Eukaryotic translation initiation factor 4 gamma 3 OS=Mus musculus GN=Eif4g3 PE=1 SV	6.9664345	9	14	8	174.781	5.53	3200000		1600000	41.89
Q80TM9	Nischarin OS=Mus musculus GN=Nisch PE=1 SV=2	0.8788449	1	1	1	174.903	5.16	550000			2.64
Q6A065	Centrosomal protein of 170 kDa OS=Mus musculus GN=Cep170 PE=1 SV=2	2.7078086	3	5	1	174.943	7.17	1100000			16.21
Q80U28	MAP kinase-activating death domain protein OS=Mus musculus GN=Madd PE=1 SV=2	6.5948003	8	17	8	175.07	6.04	4200000	1100000	2700000	56.31
Q6NZJ6	Eukaryotic translation initiation factor 4 gamma 1 OS=Mus musculus GN=Eif4g1 PE=1 SV	4.5625	6	10	5	175.967	5.4	2500000		1800000	31.99
Q9QZC2	Plexin-C1 OS=Mus musculus GN=Plxnc1 PE=1 SV=1	0.6988564	1	2	1	176.362	7.75	240000		510000	4.67
P02468	Laminin subunit gamma-1 OS=Mus musculus GN=Lamc1 PE=1 SV=2	10.51649	12	28	12	177.185	5.21	3300000	840000	3700000	81.53
B9EJA2	Cortactin-binding protein 2 OS=Mus musculus GN=Cttnbp2 PE=1 SV=2	3.2160194	5	6	5	178.662	8.19	1800000		2100000	16.49
Q8K4P8	E3 ubiquitin-protein ligase HECW1 OS=Mus musculus GN=Hecw1 PE=1 SV=3	0.872818	1	1	1	179.356	5.39	620000			2.91
Q6DIC0	Probable global transcription activator SNF2L2 OS=Mus musculus GN=Smarca2 PE=1 SV	1.3950539	2	3	1	180.141	7.2	2500000		1600000	7.01
Q9WTQ5	A-kinase anchor protein 12 OS=Mus musculus GN=Akap12 PE=1 SV=1	8.3135392	8	13	8	180.586	4.44	1200000		1100000	39.50
Q3TKT4	Transcription activator BRG1 OS=Mus musculus GN=Smarca4 PE=1 SV=1	2.9758215	4	4	3	181.313	8	1800000			9.76
P58871	182 kDa tankyrase-1-binding protein OS=Mus musculus GN=Tnks1bp1 PE=1 SV=2	11.046512	11	16	11	181.714	4.88	1700000		2100000	47.20
Q64511	DNA topoisomerase 2-beta OS=Mus musculus GN=Top2b PE=1 SV=2	3.0397022	4	5	4	181.795	8.29	2100000		1500000	14.86
Q69ZK0	Phosphatidylinositol 3,4,5-trisphosphate-dependent Rac exchanger 1 protein OS=Mus mu	1.8787879	1	1	1	184.818	6.29	560000			2.20
Q6NSW3	A-kinase anchor protein SPHKAP OS=Mus musculus GN=Sphkap PE=1 SV=2	3.2009484	3	5	3	184.978	5.06	1100000		590000	14.20
Q6PB44	Tyrosine-protein phosphatase non-receptor type 23 OS=Mus musculus GN=Ptpn23 PE=1	1.0638298	1	1	1	185.1	6.8			400000	2.91
Q4ACU6	SH3 and multiple ankyrin repeat domains protein 3 OS=Mus musculus GN=Shank3 PE=1	5.433526	7	10	7	185.284	8.88	2700000	620000	2600000	27.76
Q9QXL2	Kinesin-like protein KIF21A OS=Mus musculus GN=Kif21a PE=1 SV=2	7.1172249	7	13	7	186.42	6.27	6800000	1100000	7700000	45.19
Q0KK55	Protein very KIND OS=Mus musculus GN=Kndc1 PE=1 SV=2	2.3536165	2	2	2	191.193	6.3	260000			5.60
Q68FD5	Clathrin heavy chain 1 OS=Mus musculus GN=Cltc PE=1 SV=3	42.985075	63	247	63	191.435	5.69	210000000	48000000	190000000	794.25
P33173	Kinesin-like protein KIF1A OS=Mus musculus GN=Kif1a PE=1 SV=2	3.480826	4	6	4	191.604	6.2	1800000		1400000	19.15
Q6ZPF3	T-lymphoma invasion and metastasis-inducing protein 2 OS=Mus musculus GN=Tiam2 PI	1.3411079	1	1	1	192.447	7.44			1700000	2.69
Q8BZ05	Arf-GAP with Rho-GAP domain, ANK repeat and PH domain-containing protein 2 OS=Mus	0.9395185	1	1	1	193.277	7.18	180000			2.41
Q4KUS2	Protein unc-13 homolog A OS=Mus musculus GN=Unc13a PE=1 SV=3	2.5116822	3	5	3	193.66	5.25	1400000		2000000	14.10

Q9Z0R4	Intersectin-1 OS=Mus musculus GN=Itsn1 PE=1 SV=2	4.7841307	7	12	7	194.176	7.91	2900000	1000000	2700000	36.05
Q8CHG3	GRIP and coiled-coil domain-containing protein 2 OS=Mus musculus GN=Gcc2 PE=1 SV=	0.8338297	1	2	1	194.325	5.12	400000		370000	5.58
P39447	Tight junction protein ZO-1 OS=Mus musculus GN=Tjp1 PE=1 SV=2	8.9398281	11	12	11	194.622	6.64	1900000		1600000	32.14
Q7TT50	Serine/threonine-protein kinase MRCK beta OS=Mus musculus GN=Cdc42bpb PE=1 SV=	3.1523643	4	11	4	194.63	6.46	2000000	260000	1400000	32.70
Q3UU96	Serine/threonine-protein kinase MRCK alpha OS=Mus musculus GN=Cdc42bpa PE=1 SV	0.7562536	1	1	1	195.413	6.51	1000000			2.05
Q61292	Laminin subunit beta-2 OS=Mus musculus GN=Lamb2 PE=1 SV=2	5.0027793	6	11	6	196.451	6.67	3300000	1000000	2500000	32.68
Q8C0T5	Signal-induced proliferation-associated 1-like protein 1 OS=Mus musculus GN=Sipa1l1 P	5.1627385	7	10	7	196.909	8.13	1200000		910000	28.97
Q6P5U7	NACHT and WD repeat domain-containing protein 2 OS=Mus musculus GN=Nwd2 PE=1	1.0907003	1	2	1	197.288	6.15	1800000		1000000	7.44
Q6A009	E3 ubiquitin-protein ligase listerin OS=Mus musculus GN=Ltn1 PE=1 SV=3	0.9620826	1	1	1	198.793	6.55	610000			3.67
P20357	Microtubule-associated protein 2 OS=Mus musculus GN=Map2 PE=1 SV=2	35.776805	55	185	55	199.01	4.91	57000000	14000000	59000000	574.31
P97927	Laminin subunit alpha-4 OS=Mus musculus GN=Lama4 PE=1 SV=2	0.8259912	1	1	1	201.692	6.21			670000	2.65
Q61037	Tuberin OS=Mus musculus GN=Tsc2 PE=1 SV=1	0.9371555	1	2	1	201.943	6.98	950000		390000	6.84
A2A5R2	Brefeldin A-inhibited guanine nucleotide-exchange protein 2 OS=Mus musculus GN=Arfge	0.8370536	1	1	1	202.11	6.55	640000			3.02
Q02357	Ankyrin-1 OS=Mus musculus GN=Ank1 PE=1 SV=2	4.887218	6	6	6	204.101	6.55	1200000		920000	16.63
B2RXS4	Plexin-B2 OS=Mus musculus GN=Plxnb2 PE=1 SV=1	0.9771987	1	1	1	206.099	5.87	1200000			3.70
Q9QZQ1	Afadin OS=Mus musculus GN=Mllt4 PE=1 SV=3	3.5164835	5	6	5	206.371	6.32	1500000		670000	16.15
A2ASQ1	Agrin OS=Mus musculus GN=Agrn PE=1 SV=1	4.6666667	7	18	7	207.403	6.32	1700000	1100000	2000000	56.27
Q6ZPE2	Myotubularin-related protein 5 OS=Mus musculus GN=Sbf1 PE=1 SV=2	3.2137118	4	6	4	208.561	7.12	2200000		1700000	18.67
P70206	Plexin-A1 OS=Mus musculus GN=Plxna1 PE=1 SV=1	3.3790919	6	10	3	210.965	6.9	2600000	920000		25.60
P70399	Tumor suppressor p53-binding protein 1 OS=Mus musculus GN=Tp53bp1 PE=1 SV=2	5.0076648	6	7	6	211.209	4.63	1400000		1200000	23.50
P70207	Plexin-A2 OS=Mus musculus GN=Plxna2 PE=1 SV=2	1.6895459	3	5	1	211.399	6.54				11.02
Q3UH93	Plexin-D1 OS=Mus musculus GN=Plxnd1 PE=1 SV=1	0.6753247	1	1	1	211.473	7.11			560000	1.86
B0V2N1	Receptor-type tyrosine-protein phosphatase S OS=Mus musculus GN=Ptprs PE=1 SV=1	9.0718406	12	23	9	211.771	7.23	3200000	2500000	4400000	72.30
Q80UG2	Plexin-A4 OS=Mus musculus GN=Plxna4 PE=1 SV=3	4.3317485	6	12	3	212.423	6.83	870000	290000	440000	33.65
Q80U93	Nuclear pore complex protein Nup214 OS=Mus musculus GN=Nup214 PE=1 SV=2	0.7194245	1	2	1	212.847	7.08	400000		310000	5.69
A2AQ25	Sickle tail protein OS=Mus musculus GN=Skt PE=1 SV=1	6.0637205	8	15	8	212.905	7.77	3300000	250000	1500000	43.69
Q8BI84	Melanoma inhibitory activity protein 3 OS=Mus musculus GN=Mia3 PE=1 SV=2	4.3005181	6	8	6	213.544	4.75	1300000		930000	18.71
G5E8K5	Ankyrin-3 OS=Mus musculus GN=Ank3 PE=1 SV=1	8.1591025	13	26	10	213.929	6.7	7900000	1100000	6000000	64.79
Q64487	Receptor-type tyrosine-protein phosphatase delta OS=Mus musculus GN=Ptprd PE=1 SV	9.0481172	12	32	9	214.274	6.55	2600000	2200000	2900000	98.42
Q99104	Unconventional myosin-Va OS=Mus musculus GN=Myo5a PE=1 SV=2	21.046951	35	80	35	215.402	8.63	23000000	2100000	15000000	226.82
Q6DFV3	Rho GTPase-activating protein 21 OS=Mus musculus GN=Arhgap21 PE=1 SV=1	1.6975309	2	5	2	215.611	7.64	600000		570000	18.43
Q5SNZ0	Girdin OS=Mus musculus GN=Ccdc88a PE=1 SV=2	1.4415376	2	2	2	215.786	6.24	100000		1100000	5.56
P08775	DNA-directed RNA polymerase II subunit RPB1 OS=Mus musculus GN=Polr2a PE=1 SV=	1.6751269	2	2	2	217.039	7.37	240000			5.36
Q6PDQ2	Chromodomain-helicase-DNA-binding protein 4 OS=Mus musculus GN=Chd4 PE=1 SV=	4.073107	5	6	4	217.614	5.81	2100000		1000000	19.46
Q61464	Zinc finger protein 638 OS=Mus musculus GN=Znf638 PE=1 SV=2	3.7244898	5	6	5	217.999	6.89	1600000		1100000	15.52
A2A690	Protein TANC2 OS=Mus musculus GN=Tanc2 PE=1 SV=1	1.1033099	2	2	2	220.125	7.97	860000			4.99
P70670	Nascent polypeptide-associated complex subunit alpha, muscle-specific form OS=Mus mu	1.920439	3	7	3	220.364	9.35	9800000	3500000	9800000	27.98
Q61282	Aggrecan core protein OS=Mus musculus GN=Acan PE=1 SV=2	1.1257036	2	4	2	221.805	4.3		640000	1600000	11.73
A2A8L1	Chromodomain-helicase-DNA-binding protein 5 OS=Mus musculus GN=Chd5 PE=1 SV=	1.2332991	2	2	1	222.375	6.24	1400000			5.73
Q8CJ19	Protein-methionine sulfoxide oxidase MICAL3 OS=Mus musculus GN=Mical3 PE=1 SV=2	4.6161565	6	8	6	223.583	5.47	1000000		970000	23.95
O88196	E3 ubiquitin-protein ligase TTC3 OS=Mus musculus GN=Ttc3 PE=1 SV=2	0.8590197	1	1	1	223.789	6.25	870000			2.84
G5E870	E3 ubiquitin-protein ligase TRIP12 OS=Mus musculus GN=Trip12 PE=1 SV=1	1.2839506	2	3	2	223.988	8.35	370000		380000	8.98
Q8C547	HEAT repeat-containing protein 5B OS=Mus musculus GN=Heatr5b PE=1 SV=3	0.6763285	1	1	1	224.176	7.14	400000			3.24
A2AGT5	Cytoskeleton-associated protein 5 OS=Mus musculus GN=Ckap5 PE=1 SV=1	4.8720472	5	7	5	225.492	7.96	2900000		1100000	22.82
Q62205	Sodium channel protein type 9 subunit alpha OS=Mus musculus GN=Scn9a PE=1 SV=2	1.4112903	2	2	2	225.666	6.52			2700000	4.49
D3YZU1	SH3 and multiple ankyrin repeat domains protein 1 OS=Mus musculus GN=Shank1 PE=1	4.1993539	7	10	7	226.178	8.34	2800000		1400000	25.96
Q8VDD5	Myosin-9 OS=Mus musculus GN=Myh9 PE=1 SV=4	17.704082	26	57	18	226.232	5.66	6600000	640000	6600000	187.16
Q8CJ40	Rootletin OS=Mus musculus GN=Crocc PE=1 SV=2	3.0861125	5	10	4	226.809	5.55	1900000	1900000	2300000	24.62

O08638	Myosin-11 OS=Mus musculus GN=Myh11 PE=1 SV=1		5.5273834	10	19	1	226.888	5.45	8700000		9900000	50.65
Q3UH66	Serine/threonine-protein kinase WNK2 OS=Mus musculus GN=Wnk2 PE=1 SV=2		3.6761284	4	4	4	227.386	5.91	810000		450000	12.16
Q6URW6	Myosin-14 OS=Mus musculus GN=Myh14 PE=1 SV=1		6.25	9	15	5	228.446	5.55	820000		1400000	49.33
A2APX8	Sodium channel protein type 1 subunit alpha OS=Mus musculus GN=Scn1a PE=1 SV=1		4.1811847	6	16	6	228.651	5.87	7000000	1200000	3900000	50.96
Q9R0L6	Pericentriolar material 1 protein OS=Mus musculus GN=Pcm1 PE=1 SV=2		0.7901235	1	1	1	228.706	5.01	180000			3.27
Q61879	Myosin-10 OS=Mus musculus GN=Myh10 PE=1 SV=2		32.540486	56	133	48	228.855	5.54	19000000	2500000	18000000	418.12
Q7TSC1	Protein PRRC2A OS=Mus musculus GN=Prrc2a PE=1 SV=1		1.1121409	2	2	2	229.063	9.39	590000		930000	4.26
Q6GYP7	Ral GTPase-activating protein subunit alpha-1 OS=Mus musculus GN=Ralgapa1 PE=1 SV		0.5896806	1	1	1	229.244	6.1	520000			2.69
Q811P8	Rho GTPase-activating protein 32 OS=Mus musculus GN=Arhgap32 PE=1 SV=2		4.1168023	7	9	7	229.576	6.86	3000000		2300000	22.82
Q80XK6	Autophagy-related protein 2 homolog B OS=Mus musculus GN=Atg2b PE=1 SV=3		0.8192771	1	2	1	231.253	5.88	880000			5.96
Q80YX1	Tenascin OS=Mus musculus GN=Tnc PE=1 SV=1		0.8056872	1	1	1	231.659	4.89		530000		3.22
Q9JMH9	Unconventional myosin-XVIIIa OS=Mus musculus GN=Myo18a PE=1 SV=2		11.170732	18	29	18	232.611	6.28	5500000	540000	4000000	87.92
Q8BIK4	Dedicator of cytokinesis protein 9 OS=Mus musculus GN=Dock9 PE=1 SV=2		1.4111922	2	3	2	235.162	7.25	630000		620000	8.04
P49025	Citron Rho-interacting kinase OS=Mus musculus GN=Cit PE=1 SV=3		3.6982968	7	10	7	235.242	6.54	1200000		2000000	24.96
E9Q3L2	Phosphatidylinositol 4-kinase alpha OS=Mus musculus GN=Pi4ka PE=1 SV=2		8.2185273	15	30	15	236.889	7.06	6300000	720000	3900000	82.55
Q3UGY8	Brefeldin A-inhibited guanine nucleotide-exchange protein 3 OS=Mus musculus GN=Arfge		1.1059908	2	2	2	239.938	5.88	1200000		1800000	5.63
Q8R1A4	Dedicator of cytokinesis protein 7 OS=Mus musculus GN=Dock7 PE=1 SV=3		0.7511737	1	1	1	241.286	6.71			220000	2.54
Q6JPI3	Mediator of RNA polymerase II transcription subunit 13-like OS=Mus musculus GN=Med1		0.724966	1	1	1	241.604	6.14		400000		2.19
B2RQC6	CAD protein OS=Mus musculus GN=Cad PE=1 SV=1		0.6741573	1	1	1	243.084	6.43	360000			2.58
Q6P4T2	U5 small nuclear ribonucleoprotein 200 kDa helicase OS=Mus musculus GN=Snrnp200 P		6.4138577	10	18	10	244.392	6.06	3900000		2000000	50.25
P15508	Spectrin beta chain, erythrocytic OS=Mus musculus GN=Sptb PE=1 SV=4		10.949248	18	43	14	245.098	5.33	4000000	590000	3700000	125.98
O88307	Sortilin-related receptor OS=Mus musculus GN=Sorl1 PE=1 SV=3		2.5282167	3	3	3	246.928	5.54	680000		530000	7.37
Q5D862	SWISS-PROT:Q5D862 Tax_Id=9606 Gene_Symbol=FLG2 Filaggrin-2	x	9.9121706	11	33	11	247.928	8.31	4800000	6200000	17000000	125.28
P83741	Serine/threonine-protein kinase WNK1 OS=Mus musculus GN=Wnk1 PE=1 SV=2		0.3365587	1	1	1	250.779	6.43		880000		2.42
Q71LX4	Talin-2 OS=Mus musculus GN=Tln2 PE=1 SV=3		4.8842105	9	12	8	253.462	5.8	3200000	240000	890000	35.06
B9EKR1	Receptor-type tyrosine-protein phosphatase zeta OS=Mus musculus GN=Ptprz1 PE=1 SV		8.5640138	18	57	18	254.247	4.88	40000000	39000000	54000000	175.81
Q61290	Voltage-dependent R-type calcium channel subunit alpha-1E OS=Mus musculus GN=Cac		2.0246479	4	5	4	257.071	8.18	2000000		960000	13.77
Q91VW5	Golgin subfamily A member 4 OS=Mus musculus GN=Golga4 PE=1 SV=2		0.6702413	1	1	1	257.406	5.36	400000			2.91
O55017	Voltage-dependent N-type calcium channel subunit alpha-1B OS=Mus musculus GN=Cac		1.7189514	3	4	3	261.314	8.59	880000			10.17
Q5SWU9	Acetyl-CoA carboxylase 1 OS=Mus musculus GN=Acaca PE=1 SV=1		0.3411514	1	1	1	265.088	6.39				1.82
Q9QX47	Protein SON OS=Mus musculus GN=Son PE=1 SV=2		2.6186579	4	6	4	265.483	5.6	820000		1000000	20.24
Q6ZQ08	CCR4-NOT transcription complex subunit 1 OS=Mus musculus GN=Cnot1 PE=1 SV=2		1.5157895	3	3	3	266.637	7.11	1300000			7.11
P97445	Voltage-dependent P/Q-type calcium channel subunit alpha-1A OS=Mus musculus GN=C		1.2668919	3	6	3	267.477	8.85	1000000		1000000	15.25
Q9WU42	Nuclear receptor corepressor 2 OS=Mus musculus GN=Ncor2 PE=1 SV=3		0.6877023	1	1	1	269.642	7.44	730000			2.50
P26039	Talin-1 OS=Mus musculus GN=Tln1 PE=1 SV=2		0.9051555	2	2	1	269.653	6.18	810000			4.54
P14873	Microtubule-associated protein 1B OS=Mus musculus GN=Map1b PE=1 SV=2		29.829545	48	157	47	270.089	4.83	33000000	12000000	28000000	519.37
P19096	Fatty acid synthase OS=Mus musculus GN=Fasn PE=1 SV=2		8.7460064	17	26	17	272.257	6.58	4400000	830000	3100000	68.32
Q99PV0	Pre-mRNA-processing-splicing factor 8 OS=Mus musculus GN=Prpf8 PE=1 SV=2		3.4689507	7	9	7	273.443	8.84	2600000		920000	24.86
F6ZDS4	Nucleoprotein TPR OS=Mus musculus GN=Tpr PE=1 SV=1		4.6894282	9	13	9	273.824	5.03	3000000		1800000	40.54
Q62261	Spectrin beta chain, non-erythrocytic 1 OS=Mus musculus GN=Sptbn1 PE=1 SV=2		54.380025	109	411	105	274.052	5.58	73000000	17000000	74000000	1299.29
Q61687	Transcriptional regulator ATRX OS=Mus musculus GN=Atrx PE=1 SV=3		3.19063	5	6	5	278.414	6.68	1400000		1400000	18.88
Q8BTM8	Filamin-A OS=Mus musculus GN=Flna PE=1 SV=5		0.6422365	1	1	1	281.046	6.04			450000	2.61
Q86YZ3	SWISS-PROT:Q86YZ3 Tax_Id=9606 Gene_Symbol=HRNR Hornerin	x	16.140351	15	37	15	282.228	10.04	1800000	690000	8600000	122.25
P16546	Spectrin alpha chain, non-erythrocytic 1 OS=Mus musculus GN=Sptan1 PE=1 SV=4		60.275081	141	476	141	284.422	5.33	95000000	40000000	120000000	1627.00
Q9JLN9	Serine/threonine-protein kinase mTOR OS=Mus musculus GN=Mtor PE=1 SV=2		2.0792468	4	6	4	288.605	7.17	1200000		420000	17.32
P70398	Probable ubiquitin carboxyl-terminal hydrolase FAF-X OS=Mus musculus GN=Usp9x PE=		7.0339977	13	23	13	290.526	5.87	3400000		2300000	68.01
Q80YV3	Transformation/transcription domain-associated protein OS=Mus musculus GN=Trrap PE		0.9746589	1	1	1	291.37	8.48			270000	2.56
E9PVA8	eIF-2-alpha kinase activator GCN1 OS=Mus musculus GN=Gcn1 PE=1 SV=1		1.0108574	2	2	2	292.834	7.36	450000		2300000	4.38

Q8BTI8	Serine/arginine repetitive matrix protein 2 OS=Mus musculus GN=Srrm2 PE=1 SV=3		2.7746948	5	7	5	294.666	12.03	2400000		790000	18.27
Q9QYR6	Microtubule-associated protein 1A OS=Mus musculus GN=Map1a PE=1 SV=2		27.917867	58	145	57	299.957	5	40000000	6000000	41000000	427.45
Q9WTS6	Teneurin-3 OS=Mus musculus GN=Tenm3 PE=1 SV=1		1.252302	2	4	1	302.874	6.51	390000			10.77
Q9WTS5	Teneurin-2 OS=Mus musculus GN=Tenm2 PE=1 SV=1		3.0028944	6	10	5	306.274	6.68	2200000		1200000	24.85
Q3UHK6	Teneurin-4 OS=Mus musculus GN=Tenm4 PE=1 SV=2		1.2630819	2	5	2	308.229	6.57	940000	550000	1200000	17.75
Q3TLH4	Protein PRRC2C OS=Mus musculus GN=Prrc2c PE=1 SV=3		1.054111	2	3	2	310.703	9.1	1600000		1900000	7.99
Q61315	Adenomatous polyposis coli protein OS=Mus musculus GN=Apc PE=1 SV=1		0.456942	1	1	1	310.899	7.58	200000			1.98
P11881	Inositol 1,4,5-trisphosphate receptor type 1 OS=Mus musculus GN=Itpr1 PE=1 SV=2		6.4750818	14	23	14	312.968	6.04	31000000	830000	3000000	67.54
Q9ESE1	Lipopolysaccharide-responsive and beige-like anchor protein OS=Mus musculus GN=Lrba		1.7857143	4	5	1	316.863	5.69	440000			13.40
Q04690	Neurofibromin OS=Mus musculus GN=Nf1 PE=1 SV=1		2.1471313	5	8	5	319.391	7.39	1300000		610000	21.93
Q9EPN1	Neurobeachin OS=Mus musculus GN=Nbea PE=1 SV=1		6.1307902	14	25	11	326.536	6.2	5600000	850000	2700000	74.13
E9Q557	Desmoplakin OS=Mus musculus GN=Dsp PE=1 SV=1		4.960111	15	24	15	332.706	6.8	2500000	900000	8000000	58.17
A2CG49	Kalirin OS=Mus musculus GN=Kalrn PE=1 SV=1		4.0148448	10	15	7	336.789	6.07	1300000		1300000	45.20
Q8BPN8	DmX-like protein 2 OS=Mus musculus GN=Dmxl2 PE=1 SV=3		11.345646	24	54	24	337.995	6.42	11000000	1600000	8000000	159.38
E9Q8I9	Protein furry homolog OS=Mus musculus GN=Fry PE=1 SV=1		1.192053	2	2	2	338.877	6.01	910000			4.44
Q52KR3	Protein prune homolog 2 OS=Mus musculus GN=Prune2 PE=1 SV=2		1.0700389	2	3	2	339.306	4.46	630000		880000	8.08
Q9ERU9	E3 SUMO-protein ligase RanBP2 OS=Mus musculus GN=Ranbp2 PE=1 SV=2		3.6357681	6	8	6	340.907	6.18	2300000		1100000	30.08
Q60675	Laminin subunit alpha-2 OS=Mus musculus GN=Lama2 PE=1 SV=2		0.5772931	1	2	1	343.593	6.09			520000	6.22
P42859	Huntingtin OS=Mus musculus GN=Htt PE=1 SV=2		1.3465854	3	3	3	344.471	6.29	3100000			8.96
Q0KL02	Triple functional domain protein OS=Mus musculus GN=Trio PE=1 SV=3		5.1901999	10	18	7	347.643	6.35	2800000	250000	1900000	53.25
Q5H8C4	Vacuolar protein sorting-associated protein 13A OS=Mus musculus GN=Vps13a PE=1 SV		1.0739103	3	5	3	359.173	6.19	1400000		670000	12.13
Q62059	Versican core protein OS=Mus musculus GN=Vcan PE=1 SV=2		3.6044087	9	17	9	366.56	4.64	3500000	5400000	7500000	50.58
Q05793	Basement membrane-specific heparan sulfate proteoglycan core protein OS=Mus muscul		3.5608309	9	20	9	398.039	6.32	1600000		1800000	56.02
Q61001	Laminin subunit alpha-5 OS=Mus musculus GN=Lama5 PE=1 SV=4		0.7799892	2	4	2	403.792	6.73	600000		580000	12.24
O88737	Protein bassoon OS=Mus musculus GN=Bsn PE=1 SV=4		25.190259	65	148	63	418.587	7.71	15000000	1400000	12000000	462.99
Q8BX70	Vacuolar protein sorting-associated protein 13C OS=Mus musculus GN=Vps13c PE=1 SV		0.9338314	3	4	3	419.824	6.81	1600000		840000	11.66
P11531	Dystrophin OS=Mus musculus GN=Dmd PE=1 SV=3		0.8972268	3	5	3	425.566	5.94	1300000	530000	720000	13.48
Q8C8R3	Ankyrin-2 OS=Mus musculus GN=Ank2 PE=1 SV=2		24.807594	60	164	57	425.999	5.17	29000000	5400000	21000000	527.99
P20930	SWISS-PROT:P20930 Tax_Id=9606 Gene_Symbol=FLG Filaggrin	x	1.2804728	3	6	3	434.922	9.25	2500000		11000000	23.06
Q7TMY8	E3 ubiquitin-protein ligase HUWE1 OS=Mus musculus GN=Huwe1 PE=1 SV=5		0.4112406	1	1	1	482.332	5.22	280000			2.54
Q91ZX7	Prolow-density lipoprotein receptor-related protein 1 OS=Mus musculus GN=Lrp1 PE=1 S		10.979098	34	73	34	504.411	5.36	3400000	1700000	4200000	229.90
Q9JHU4	Cytoplasmic dynein 1 heavy chain 1 OS=Mus musculus GN=Dync1h1 PE=1 SV=2		17.011197	69	155	69	531.71	6.42	17000000	1900000	11000000	441.48
O88738	Baculoviral IAP repeat-containing protein 6 OS=Mus musculus GN=Birc6 PE=1 SV=2		0.3277345	1	1	1	531.833	6.07	400000			3.86
Q9QXS1	Plectin OS=Mus musculus GN=Plec PE=1 SV=3		12.875719	51	91	50	533.861	5.96	6700000	920000	7200000	252.21
Q9QYX7	Protein piccolo OS=Mus musculus GN=Pclo PE=1 SV=4		9.7277032	40	67	38	550.496	6.51	5500000	480000	5900000	178.86
E9Q401	Ryanodine receptor 2 OS=Mus musculus GN=Ryr2 PE=1 SV=1		2.8393073	11	19	11	564.458	6.09	2900000		1700000	49.47
A2AN08	E3 ubiquitin-protein ligase UBR4 OS=Mus musculus GN=Ubr4 PE=1 SV=1		0.2123552	1	2	1	571.927	6.06	1100000		770000	4.46
Q9QXZ0	Microtubule-actin cross-linking factor 1 OS=Mus musculus GN=Macf1 PE=1 SV=2		5.847158	31	49	30	831.362	5.43	3700000		3000000	148.84
Q91ZU6	Dystonin OS=Mus musculus GN=Dst PE=1 SV=2		1.8260517	9	11	9	833.701	5.31	1000000		940000	32.75
Q6ZWR6	Nesprin-1 OS=Mus musculus GN=Syne1 PE=1 SV=2		1.2274122	8	12	8	1009.295	5.59	2400000		1400000	34.43
