# Supplementary material for: Ultra-High Mass Resolution MALDI Imaging Mass Spectrometry of Proteins and Metabolites in a Mouse Model of Glioblastoma
Source: Sci Rep. 2017 Apr 4;7:603. doi: 10.1038/s41598-017-00703-w (PMC5429601; doi:10.1038/s41598-017-00703-w)
Supplement: Supplementary file 3 — Supplementary Protein List PL2 [file 41598_2017_703_MOESM3_ESM.pdf]

Accession	Description	Contaminant	Coverage	# Peptides	# PSMs	# Unique Peptides	MW [kDa]	calc. pI	Area: F5: Sample	Area: F4: Sample	Area: F6: Sample	Score Sequest HT
P62892	60S ribosomal protein L39 OS=Mus musculus GN=Rpl39 PE=1 SV=2		19.60784	1	3	1	6.403	12.56	4500000	4200000	3300000	8.101977
Q8R1I1	Cytochrome b-c1 complex subunit 9 OS=Mus musculus GN=Uqcr10 PE=1 SV=1		26.5625	1	6	1	7.441	9.19	17000000	9100000	8200000	22.29919
P62858	40S ribosomal protein S28 OS=Mus musculus GN=Rps28 PE=1 SV=1		30.43478	2	3	2	7.836	10.7	10000000	21000000		10.04869
P63213	Guanine nucleotide-binding protein G(I)/G(S)/G(O) subunit gamma-2 OS=Mus musculus GN=Gng2 PE=1 SV=2		22.53521	1	3	1	7.845	7.99	2300000	820000	1100000	9.147856
Q9DAS9	Guanine nucleotide-binding protein G(I)/G(S)/G(O) subunit gamma-12 OS=Mus musculus GN=Gng12 PE=1 SV=3		15.27778	1	1	1	7.992	8.97	650000			2.037953
Q9CR64	Protein kish-A OS=Mus musculus GN=Tmem167a PE=1 SV=1		12.5	1	1	1	8.068	8.95		1500000		2.285073
Q9JJI8	60S ribosomal protein L38 OS=Mus musculus GN=Rpl38 PE=1 SV=3		28.57143	2	13	2	8.199	10.1	4200000	3000000	2900000	29.35109
P63216	Guanine nucleotide-binding protein G(I)/G(S)/G(O) subunit gamma-3 OS=Mus musculus GN=Gng3 PE=1 SV=1		20	1	3	1	8.299	7.78	2600000	820000	460000	7.921583
Q9CPQ1	Cytochrome c oxidase subunit 6C OS=Mus musculus GN=Cox6c PE=1 SV=3		28.94737	3	5	3	8.464	10.14	8300000	5600000	2800000	10.42596
P59648	FXYD domain-containing ion transport regulator 7 OS=Mus musculus GN=Fxyd7 PE=3 SV=1		16.25	1	3	1	8.481	7.84	4600000	2900000	2000000	6.971987
P62309	Small nuclear ribonucleoprotein G OS=Mus musculus GN=Snrpg PE=1 SV=1		15.78947	1	1	1	8.49	8.88	1100000			3.235302
P29595	NEDD8 OS=Mus musculus GN=Nedd8 PE=1 SV=2		13.58025	1	3	1	8.967	7.25	8000000	7600000	6500000	9.017698
Q9CR20	Immediate early response 3-interacting protein 1 OS=Mus musculus GN=Ier3ip1 PE=3 SV=1		24.39024	1	1	1	9.011	8.22		850000		3.230605
Q9CQR2	40S ribosomal protein S21 OS=Mus musculus GN=Rps21 PE=1 SV=1		12.04819	1	2	1	9.136	8.51		13000000	5500000	5.815672
Q62425	Cytochrome c oxidase subunit NDUFA4 OS=Mus musculus GN=Ndufa4 PE=1 SV=2		26.82927	2	3	2	9.321	9.52	3600000	3800000		7.781106
Q9D8X0	Protein MANBAL OS=Mus musculus GN=Manbal PE=3 SV=2		14.11765	1	1	1	9.334	9.38			190000	3.03163
Q6ZWU9	40S ribosomal protein S27 OS=Mus musculus GN=Rps27 PE=1 SV=3		38.09524	3	10	1	9.455	9.45	5500000	15000000	13000000	26.09497
Q6ZWY3	40S ribosomal protein S27-like OS=Mus musculus GN=Rps27l PE=1 SV=3		38.09524	3	9	1	9.471	9.45	8400000	9500000	8700000	21.86399
Q810Q5	Normal mucosa of esophagus-specific gene 1 protein OS=Mus musculus GN=Nmes1 PE=1 SV=1		18.07229	1	1	1	9.578	9.89		2600000		2.539128
P20491	High affinity immunoglobulin epsilon receptor subunit gamma OS=Mus musculus GN=Fcer1g PE=1 SV=1		23.25581	2	6	2	9.646	7.97	3400000	2800000	2100000	13.25079
Q9CQ69	Cytochrome b-c1 complex subunit 8 OS=Mus musculus GN=Uqcrq PE=1 SV=3		10.97561	1	3	1	9.762	10.26	2300000	3200000	1100000	7.01569
Q9CQS8	Protein transport protein Sec61 subunit beta OS=Mus musculus GN=Sec61b PE=1 SV=3		21.875	2	5	2	9.952	11.56	19000000	17000000	12000000	11.63555
P31786	Acyl-CoA-binding protein OS=Mus musculus GN=Dbi PE=1 SV=2		20.68966	1	1	1	9.994	8.82		5100000		3.347131
P56391	Cytochrome c oxidase subunit 6B1 OS=Mus musculus GN=Cox6b1 PE=1 SV=2		29.06977	2	3	2	10.065	8.72		2200000	840000	6.999919
Q923D4	Splicing factor 3B subunit 5 OS=Mus musculus GN=Sf3b5 PE=1 SV=1		17.44186	1	1	1	10.113	6.35		1000000		2.578246
Q9D1Q4	Dolichol-phosphate mannosyltransferase subunit 3 OS=Mus musculus GN=Dpm3 PE=1 SV=1		23.91304	2	2	2	10.132	7.08		1200000		2.562106
P18608	Non-histone chromosomal protein HMG-14 OS=Mus musculus GN=Hmgn1 PE=1 SV=2		31.25	1	2	1	10.146	9.76		570000	310000	7.319184
P61514	60S ribosomal protein L37a OS=Mus musculus GN=Rpl37a PE=1 SV=2		8.695652	1	1	1	10.268	10.43	3000000			1.865742
P62073	Mitochondrial import inner membrane translocase subunit Tim10 OS=Mus musculus GN=Timm10 PE=1 SV=1		14.44444	1	1	1	10.326	6.29		670000		3.336424
P56135	ATP synthase subunit f, mitochondrial OS=Mus musculus GN=Atp5j2 PE=1 SV=3		13.63636	1	3	1	10.337	9.95	14000000	7300000	7900000	7.131487
P63168	Dynein light chain 1, cytoplasmic OS=Mus musculus GN=Dynll1 PE=1 SV=1		37.07865	2	10	2	10.359	7.4	1500000	5300000	530000	22.98514
P99028	Cytochrome b-c1 complex subunit 6, mitochondrial OS=Mus musculus GN=Uqcrh PE=1 SV=2		44.94382	3	6	3	10.428	4.87	6800000	10000000	4500000	18.60857
P56565	Protein S100-A1 OS=Mus musculus GN=S100a1 PE=1 SV=2		13.82979	1	1	1	10.498	4.5		4400000		2.831232
Q9D638	Keratin-associated protein 3-2 OS=Mus musculus GN=Krtap3-2 PE=3 SV=2		17.17172	1	1	1	10.567	6.19		1900000		2.424828
O70554	Small proline-rich protein 2B OS=Mus musculus GN=Sprr2b PE=2 SV=1		9.183673	1	2	1	10.727	7.46	6900000			5.456643
P17515	C-X-C motif chemokine 10 OS=Mus musculus GN=Cxcl10 PE=1 SV=1		12.2449	1	1	1	10.782	9.82	1300000			2.39485
P62305	Small nuclear ribonucleoprotein E OS=Mus musculus GN=Snrpe PE=1 SV=1		13.04348	1	3	1	10.797	9.44	7700000	6600000	5600000	7.492098
Q8K215	LYR motif-containing protein 4 OS=Mus musculus GN=Lyrm4 PE=1 SV=1		12.08791	1	1	1	10.848	10.13	970000			2.817861
P61957	Small ubiquitin-related modifier 2 OS=Mus musculus GN=Sumo2 PE=1 SV=1		23.15789	2	6	2	10.864	5.5	20000000	18000000	23000000	12.95864
Q64433	10 kDa heat shock protein, mitochondrial OS=Mus musculus GN=Hspe1 PE=1 SV=2		9.803922	1	1	1	10.956	8.35		2900000		1.818684
P97352	Protein S100-A13 OS=Mus musculus GN=S100a13 PE=1 SV=1		11.22449	1	2	1	11.151	6.13		700000	620000	5.275613
P08207	Protein S100-A10 OS=Mus musculus GN=S100a10 PE=1 SV=2		17.52577	2	2	2	11.179	6.77		2100000		4.284756
P62806	Histone H4 OS=Mus musculus GN=Hist1h4a PE=1 SV=2		58.25243	12	100	12	11.36	11.36	1.4E+09	1.2E+09	1.5E+09	292.5049
Q9CPQ8	ATP synthase subunit g, mitochondrial OS=Mus musculus GN=Atp5l PE=1 SV=1		18.4466	1	2	1	11.417	9.74	1900000	1000000		4.707243
Q9D0J8	Parathymosin OS=Mus musculus GN=Ptms PE=1 SV=3		10.89109	1	3	1	11.423	4.22	5300000	6600000	4700000	8.318395
O70404	Vesicle-associated membrane protein 8 OS=Mus musculus GN=Vamp8 PE=1 SV=1		20.79208	2	5	2	11.444	8.19	1500000	1500000		10.80308
P47955	60S acidic ribosomal protein P1 OS=Mus musculus GN=Rplp1 PE=1 SV=1		14.03509	1	3	1	11.468	4.32	1200000	1500000	390000	9.223303
P63024	Vesicle-associated membrane protein 3 OS=Mus musculus GN=Vamp3 PE=1 SV=1		38.83495	4	12	1	11.473	8.5	1700000	1400000	1300000	43.25176
P63166	Small ubiquitin-related modifier 1 OS=Mus musculus GN=Sumo1 PE=1 SV=1		20.79208	2	3	2	11.55	5.52		1200000	3500000	7.405226
P62897	Cytochrome c, somatic OS=Mus musculus GN=Cycs PE=1 SV=2		20.95238	2	6	2	11.598	9.58	5000000	4100000	5000000	20.12097

P17095	High mobility group protein HMG-I/HMG-Y OS=Mus musculus GN=Hmga1 PE=1 SV=4		22.42991	2	7	2	11.607	10.32	11000000	12000000	11000000	19.70902
P99027	60S acidic ribosomal protein P2 OS=Mus musculus GN=Rplp2 PE=1 SV=3		60	5	15	5	11.644	4.54	74000000	59000000	55000000	58.72017
P10639	Thioredoxin OS=Mus musculus GN=Txn PE=1 SV=3		12.38095	1	3	1	11.668	4.92	10000000	12000000	8500000	11.73618
Q9CQZ6	NADH dehydrogenase [ubiquinone] 1 beta subcomplex subunit 3 OS=Mus musculus GN=Ndufb3 PE=1 SV=1		17.30769	2	4	2	11.685	9.04	9700000	11000000	9300000	9.735468
P62818	Protein S100-A3 OS=Mus musculus GN=S100a3 PE=2 SV=1		10.89109	1	2	1	11.739	4.78		970000	1600000	5.57858
P01837	Ig kappa chain C region OS=Mus musculus PE=1 SV=1		41.50943	3	9	3	11.771	5.41	9500000	9400000	7300000	32.36418
P52927	High mobility group protein HMGI-C OS=Mus musculus GN=Hmga2 PE=1 SV=1		31.48148	2	10	2	11.812	10.62	2600000	4900000	4500000	32.0354
P26883	Peptidyl-prolyl cis-trans isomerase FKBP1A OS=Mus musculus GN=Fkbp1a PE=1 SV=2		12.96296	1	1	1	11.915	8.16		1600000		3.767179
Q91WS0	CDGSH iron-sulfur domain-containing protein 1 OS=Mus musculus GN=Cisd1 PE=1 SV=1		35.18519	3	14	3	12.089	9.06	11000000	13000000	9800000	50.46018
O35143	ATPase inhibitor, mitochondrial OS=Mus musculus GN=Atpif1 PE=1 SV=2		6.603774	1	1	1	12.151	9.64	4100000			2.040064
P47964	60S ribosomal protein L36 OS=Mus musculus GN=Rpl36 PE=3 SV=2		18.09524	2	6	2	12.208	11.34	9000000	7700000	8000000	12.82858
P26350	Prothymosin alpha OS=Mus musculus GN=Ptma PE=1 SV=2		21.62162	3	14	3	12.247	3.79	6200000	6000000	5600000	47.23169
P84089	Enhancer of rudimentary homolog OS=Mus musculus GN=Erh PE=1 SV=1		26.92308	2	3	2	12.251	5.92	630000	3600000		7.364164
P01631	Ig kappa chain V-II region 26-10 OS=Mus musculus PE=1 SV=1		11.50442	1	2	1	12.265	8.88	10000000	7100000		6.690285
P62878	E3 ubiquitin-protein ligase RBX1 OS=Mus musculus GN=Rbx1 PE=1 SV=1		7.407407	1	2	1	12.266	6.96	4700000		4000000	4.841075
P19788	Matrix Gla protein OS=Mus musculus GN=Mgp PE=3 SV=1		23.07692	2	6	2	12.351	9.55	2500000	7400000	1100000	16.07163
P83870	PHD finger-like domain-containing protein 5A OS=Mus musculus GN=Phf5a PE=1 SV=1		32.72727	3	4	3	12.397	8.41	1300000	2400000	900000	10.76346
P83882	60S ribosomal protein L36a OS=Mus musculus GN=Rpl36a PE=1 SV=2		16.03774	2	3	2	12.433	10.58	5400000	10000000		7.080646
P51807	Dynein light chain Tctex-type 1 OS=Mus musculus GN=Dynlt1 PE=1 SV=1		14.15929	1	1	1	12.475	5.08		1200000		2.650357
P61804	Dolichyl-diphosphooligosaccharide--protein glycosyltransferase subunit DAD1 OS=Mus musculus GN=Dad1 PE=1 SV=3		19.46903	2	5	2	12.489	7.08	4000000	3800000	3500000	14.2747
P16254	Signal recognition particle 14 kDa protein OS=Mus musculus GN=Srp14 PE=1 SV=1		10.90909	1	2	1	12.502	10.17	5400000	6000000		5.685144
O55142	60S ribosomal protein L35a OS=Mus musculus GN=Rpl35a PE=1 SV=2		20.90909	3	8	3	12.546	10.89	29000000	25000000	26000000	15.54398
Q99LY9	NADH dehydrogenase [ubiquinone] iron-sulfur protein 5 OS=Mus musculus GN=Ndufs5 PE=1 SV=3		23.58491	2	4	2	12.639	8.92	1400000	1100000	1000000	12.05376
P63044	Vesicle-associated membrane protein 2 OS=Mus musculus GN=Vamp2 PE=1 SV=2		34.48276	4	12	1	12.683	8.13	4400000		1500000	40.58613
P48024	Eukaryotic translation initiation factor 1 OS=Mus musculus GN=Eif1 PE=1 SV=2		47.78761	3	5	3	12.739	7.44	8100000	5700000		14.2734
P48428	Tubulin-specific chaperone A OS=Mus musculus GN=Tbca PE=1 SV=3		7.407407	1	2	1	12.75	5.27	910000		1400000	5.29217
P62889	60S ribosomal protein L30 OS=Mus musculus GN=Rpl30 PE=1 SV=2		58.26087	6	18	6	12.776	9.63	21000000	22000000	20000000	56.92119
P62774	Myotrophin OS=Mus musculus GN=Mtpn PE=1 SV=2		14.40678	1	1	1	12.853	5.52		1500000		2.99752
P62855	40S ribosomal protein S26 OS=Mus musculus GN=Rps26 PE=1 SV=3		20.86957	2	6	2	13.007	11	24000000	24000000	24000000	19.19375
P03899	NADH-ubiquinone oxidoreductase chain 3 OS=Mus musculus GN=Mtnd3 PE=1 SV=3		13.04348	1	1	1	13.21	4.64		1500000		2.137059
P62315	Small nuclear ribonucleoprotein Sm D1 OS=Mus musculus GN=Snrpd1 PE=1 SV=1		20.16807	3	7	3	13.273	11.56	4300000	3200000	4500000	18.03035
Q9D1R9	60S ribosomal protein L34 OS=Mus musculus GN=Rpl34 PE=1 SV=2		20.51282	3	8	3	13.284	11.47	24000000	36000000	27000000	16.88366
P60840	Alpha-endosulfine OS=Mus musculus GN=Ensa PE=1 SV=1		11.57025	1	1	1	13.327	7.24		330000		2.403036
Q9CPP6	NADH dehydrogenase [ubiquinone] 1 alpha subcomplex subunit 5 OS=Mus musculus GN=Ndufa5 PE=1 SV=3		21.55172	2	4	2	13.351	8.1	4600000	1900000	38000000	9.652988
Q9D1K2	V-type proton ATPase subunit F OS=Mus musculus GN=Atp6v1f PE=1 SV=2		10.08403	1	3	1	13.362	5.82	2500000	1300000	1400000	8.114889
P60867	40S ribosomal protein S20 OS=Mus musculus GN=Rps20 PE=1 SV=1		25.21008	3	10	3	13.364	9.94	19000000	22000000	23000000	30.72694
Q8R404	MICOS complex subunit MIC13 OS=Mus musculus GN=Mic13 PE=1 SV=1		5.882353	1	1	1	13.365	8.63	5800000			1.855439
P62317	Small nuclear ribonucleoprotein Sm D2 OS=Mus musculus GN=Snrpd2 PE=1 SV=1		47.45763	5	16	5	13.518	9.91	16000000	16000000	13000000	42.06528
Q9D855	Cytochrome b-c1 complex subunit 7 OS=Mus musculus GN=Uqcrb PE=1 SV=3		24.32432	3	9	3	13.519	9.11	5800000	5900000	2700000	22.58943
O88653	Ragulator complex protein LAMTOR3 OS=Mus musculus GN=Lamtor3 PE=1 SV=1		8.870968	1	1	1	13.544	7.34	390000			1.942976
P0C0S6	Histone H2A.Z OS=Mus musculus GN=H2afz PE=1 SV=2		31.25	4	19	2	13.545	10.58	98000000	81000000	92000000	52.08552
Q9WTT4	V-type proton ATPase subunit G 2 OS=Mus musculus GN=Atp6v1g2 PE=1 SV=1		26.27119	2	4	2	13.643	10.26	560000	1600000		14.92192
Q9DBG9	Tax1-binding protein 3 OS=Mus musculus GN=Tax1bp3 PE=1 SV=1		13.70968	1	3	1	13.714	8.48	1800000	2400000	1800000	7.170875
Q9CR51	V-type proton ATPase subunit G 1 OS=Mus musculus GN=Atp6v1g1 PE=1 SV=3		23.72881	2	6	2	13.716	7.97	760000	7400000	5000000	21.67019
P62852	40S ribosomal protein S25 OS=Mus musculus GN=Rps25 PE=1 SV=1		22.4	3	8	3	13.734	10.11	10000000	11000000	9200000	20.58092
P70349	Histidine triad nucleotide-binding protein 1 OS=Mus musculus GN=Hint1 PE=1 SV=3		11.11111	1	3	1	13.768	6.87	2400000	3800000	2400000	7.72173
P01887	Beta-2-microglobulin OS=Mus musculus GN=B2m PE=1 SV=2		15.96639	2	5	2	13.77	8.44	8900000	16000000	7300000	12.91742
Q64253	Lymphocyte antigen 6E OS=Mus musculus GN=Ly6e PE=1 SV=2		10.76923	1	3	1	13.791	7.05	6500000	4900000	3900000	9.895208
P19536	Cytochrome c oxidase subunit 5B, mitochondrial OS=Mus musculus GN=Cox5b PE=1 SV=1		15.625	2	3	2	13.804	8.38	2700000	12000000		6.949335
P62320	Small nuclear ribonucleoprotein Sm D3 OS=Mus musculus GN=Snrpd3 PE=1 SV=1		24.60317	2	8	2	13.907	10.32	8200000	6700000	6100000	24.24367
P10853	Histone H2B type 1-F/J/L OS=Mus musculus GN=Hist1h2bf PE=1 SV=2		46.03175	8	51	5	13.928	10.32	22000000	6.5E+08	7E+08	164.3978
Q64524	Histone H2B type 2-E OS=Mus musculus GN=Hist2h2be PE=1 SV=3		34.92063	4	35	1	13.985	10.32	33000000	23000000	25000000	114.1996
P22752	Histone H2A type 1 OS=Mus musculus GN=Hist1h2ab PE=1 SV=3		32.30769	4	30	2	14.127	11.05	2.4E+08	2.1E+08	2.1E+08	88.00238

Q9DCG9	Multifunctional methyltransferase subunit TRM112-like protein OS=Mus musculus GN=Trmt112 PE=1 SV=1		10.4	1	2	1	14.132	5.27	850000		400000	6.154097
Q9D0T1	NHP2-like protein 1 OS=Mus musculus GN=Snu13 PE=1 SV=4		9.375	1	3	1	14.165	8.46	12000000	9600000	7400000	10.63295
P11031	Activated RNA polymerase II transcriptional coactivator p15 OS=Mus musculus GN=Sub1 PE=1 SV=3		32.28346	5	18	5	14.418	9.6	64000000	59000000	83000000	53.10147
P18340	C-X-C motif chemokine 9 OS=Mus musculus GN=Cxcl9 PE=2 SV=2		11.90476	1	1	1	14.435	10.52		640000		1.851094
P62900	60S ribosomal protein L31 OS=Mus musculus GN=Rpl31 PE=1 SV=1		28	4	11	4	14.454	10.54	36000000	48000000	31000000	27.14751
Q9D7S7	60S ribosomal protein L22-like 1 OS=Mus musculus GN=Rpl22l1 PE=1 SV=1		9.836066	1	1	1	14.459	9.45		3100000		2.578572
Q9CZL2	Uncharacterized protein C4orf32 homolog OS=Mus musculus PE=1 SV=1		8.396947	1	1	1	14.501	4.97		230000		2.039251
P63323	40S ribosomal protein S12 OS=Mus musculus GN=Rps12 PE=1 SV=2		12.87879	2	2	2	14.516	7.24	24000000	4700000		5.059968
Q6ZWV7	60S ribosomal protein L35 OS=Mus musculus GN=Rpl35 PE=1 SV=1		8.130081	1	3	1	14.544	11.05	40000000	48000000	46000000	9.339207
P59708	Splicing factor 3B subunit 6 OS=Mus musculus GN=Sf3b6 PE=1 SV=1		9.6	1	2	1	14.576	9.38	1700000	2000000		6.467608
Q9CQV6	Microtubule-associated proteins 1A/1B light chain 3B OS=Mus musculus GN=Map1lc3b PE=1 SV=3		5.6	1	3	1	14.608	8.43	3700000	2700000	2500000	5.648575
Q9WUC3	Lymphocyte antigen 6H OS=Mus musculus GN=Ly6h PE=1 SV=2		22.30216	3	8	3	14.659	7.53	4900000	5300000	4200000	21.90131
Q9DBX1	Regulator of cell cycle RGCC OS=Mus musculus GN=Rgcc PE=1 SV=1		8.029197	1	2	1	14.708	4.84	2700000		2400000	5.093468
P67984	60S ribosomal protein L22 OS=Mus musculus GN=Rpl22 PE=1 SV=2		31.25	4	12	4	14.75	9.19	40000000	35000000	36000000	32.82258
P62245	40S ribosomal protein S15a OS=Mus musculus GN=Rps15a PE=1 SV=2		29.23077	4	15	4	14.83	10.13	18000000	18000000	17000000	42.98471
P16045	Galectin-1 OS=Mus musculus GN=Lgals1 PE=1 SV=3		37.77778	4	11	4	14.856	5.49	2000000	5400000	1900000	34.3309
P62830	60S ribosomal protein L23 OS=Mus musculus GN=Rpl23 PE=1 SV=1		37.85714	4	13	4	14.856	10.51	19000000	17000000	16000000	33.33838
Q9CQW9	Interferon-induced transmembrane protein 3 OS=Mus musculus GN=Ifitm3 PE=1 SV=1		30.65693	3	7	2	14.945	7.4	2700000	2400000	2300000	23.87326
Q9JKC6	Cell cycle exit and neuronal differentiation protein 1 OS=Mus musculus GN=Cend1 PE=1 SV=1		19.46309	2	7	2	14.978	8.97	4600000	4100000	2000000	24.09263
Q9JJV2	Profilin-2 OS=Mus musculus GN=Pfn2 PE=1 SV=3		20	2	2	2	15.022	6.99		6300000		6.675588
P02584	SWISS-PROT:P02584 (Bos taurus) Profilin-1	x	18.57143	2	4	2	15.048	8.28	4800000	1200000	6900000	10.12133
Q9QXA5	U6 snRNA-associated Sm-like protein LSm4 OS=Mus musculus GN=Lsm4 PE=1 SV=1		7.29927	1	3	1	15.067	10.05	2000000	2100000	1500000	7.829657
Q9CQC7	NADH dehydrogenase [ubiquinone] 1 beta subcomplex subunit 4 OS=Mus musculus GN=Ndufb4 PE=1 SV=3		7.751938	1	3	1	15.072	9.89	4100000	4100000	2000000	8.66014
P01942	Hemoglobin subunit alpha OS=Mus musculus GN=Hba PE=1 SV=2		26.76056	3	11	3	15.076	8.22	15000000	22000000	3600000	28.65335
Q9CQR4	Acyl-coenzyme A thioesterase 13 OS=Mus musculus GN=Acot13 PE=1 SV=1		7.142857	1	1	1	15.173	8.82			2100000	2.574043
P56395	Cytochrome b5 OS=Mus musculus GN=Cyb5a PE=1 SV=2		24.62687	2	4	2	15.232	5.07	9400000	11000000	8000000	18.22511
Q9CQB5	CDGSH iron-sulfur domain-containing protein 2 OS=Mus musculus GN=Cisd2 PE=1 SV=1		5.185185	1	1	1	15.232	9.51				1.861145
Q9CQN7	39S ribosomal protein L41, mitochondrial OS=Mus musculus GN=Mrpl41 PE=1 SV=1		9.62963	1	2	1	15.252	9.82	1400000	1400000		4.961267
P02301	Histone H3.3C OS=Mus musculus GN=H3f3c PE=3 SV=3		21.32353	4	23	4	15.305	11.14	1.4E+09	1E+09	1.5E+09	51.05982
P45878	Peptidyl-prolyl cis-trans isomerase FKBP2 OS=Mus musculus GN=Fkbp2 PE=1 SV=1		10	1	3	1	15.334	8.88	2800000	3000000	1900000	8.172156
P84086	Complexin-2 OS=Mus musculus GN=Cplx2 PE=1 SV=1		17.16418	1	1	1	15.385	5.08			620000	2.589733
P62849	40S ribosomal protein S24 OS=Mus musculus GN=Rps24 PE=1 SV=1		19.54887	2	11	2	15.413	10.78	10000000	8200000	7600000	31.53478
P63276	40S ribosomal protein S17 OS=Mus musculus GN=Rps17 PE=1 SV=2		25.92593	4	14	4	15.514	9.85	11000000	13000000	11000000	60.03712
P21460	Cystatin-C OS=Mus musculus GN=Cst3 PE=1 SV=2		19.28571	2	6	2	15.521	9	5100000	4500000	4000000	18.92113
Q9CPQ3	Mitochondrial import receptor subunit TOM22 homolog OS=Mus musculus GN=Tomm22 PE=1 SV=3		22.53521	2	6	2	15.527	4.34	2800000	3100000	3300000	23.14245
Q3TGF2	Protein FAM107B OS=Mus musculus GN=Fam107b PE=1 SV=2		6.870229	1	1	1	15.562	8.31			440000	2.131271
P41105	60S ribosomal protein L28 OS=Mus musculus GN=Rpl28 PE=1 SV=2		16.05839	2	9	2	15.724	12.02	63000000	67000000	55000000	22.73002
Q99J93	Interferon-induced transmembrane protein 2 OS=Mus musculus GN=Ifitm2 PE=1 SV=1		18.05556	2	3	1	15.733	7.3		930000	710000	7.852386
Q62266	Cornifin-A OS=Mus musculus GN=Sprr1a PE=1 SV=1		16.66667	1	1	1	15.755	7.85			13000000	2.085301
P07309	Transthyretin OS=Mus musculus GN=Ttr PE=1 SV=1		8.843537	1	2	1	15.766	6.16	2100000	2200000		6.574722
P61358	60S ribosomal protein L27 OS=Mus musculus GN=Rpl27 PE=1 SV=2		34.55882	4	11	4	15.788	10.56	30000000	28000000	9800000	27.96385
P62267	40S ribosomal protein S23 OS=Mus musculus GN=Rps23 PE=1 SV=3		7.692308	1	5	1	15.798	10.49	12000000	14000000	9100000	10.73585
P02088	Hemoglobin subunit beta-1 OS=Mus musculus GN=Hbb-b1 PE=1 SV=2		38.09524	4	16	4	15.83	7.65	21000000	24000000	20000000	48.98452
P62911	60S ribosomal protein L32 OS=Mus musculus GN=Rpl32 PE=1 SV=2		33.33333	4	18	4	15.85	11.33	12000000	10000000	8000000	55.16064
Q9CQI6	Coactosin-like protein OS=Mus musculus GN=Cotl1 PE=1 SV=3		11.26761	2	3	2	15.934	5.4	3000000	2800000		6.263401
Q9CZX8	40S ribosomal protein S19 OS=Mus musculus GN=Rps19 PE=1 SV=3		37.93103	7	17	7	16.076	10.4	52000000	44000000	45000000	48.18167
P12787	Cytochrome c oxidase subunit 5A, mitochondrial OS=Mus musculus GN=Cox5a PE=1 SV=2		16.43836	2	7	2	16.091	6.54	4200000	4500000	2200000	21.76003
Q8K5B2	Multiple coagulation factor deficiency protein 2 homolog OS=Mus musculus GN=Mcfd2 PE=1 SV=1		8.965517	1	3	1	16.158	4.72	8100000	8800000	6200000	9.546401
Q9D7A6	Signal recognition particle 19 kDa protein OS=Mus musculus GN=Srp19 PE=1 SV=1		9.722222	1	3	1	16.181	9.96	2200000	1900000	2200000	10.58491
P62264	40S ribosomal protein S14 OS=Mus musculus GN=Rps14 PE=1 SV=3		32.45033	6	23	6	16.263	10.05	56000000	51000000	53000000	81.28122
Q9CPW4	Actin-related protein 2/3 complex subunit 5 OS=Mus musculus GN=Arpc5 PE=1 SV=3		35.76159	3	7	3	16.278	5.67	4000000	4500000	3500000	23.15726
Q9CQX2	Cytochrome b5 type B OS=Mus musculus GN=Cyb5b PE=1 SV=1		31.50685	2	5	2	16.308	4.89	3800000	5300000	2700000	16.68966
Q9CQY6	Ubiquinol-cytochrome-c reductase complex assembly factor 2 OS=Mus musculus GN=Uqcc2 PE=1 SV=1		9.558824	1	1	1	16.31	9.19		1600000		2.559959

Q9CR61	NADH dehydrogenase [ubiquinone] 1 beta subcomplex subunit 7 OS=Mus musculus GN=Ndufb7 PE=1 SV=3		16.78832	2	4	2	16.32	8.18	8900000	11000000	7700000	12.29208
O70480	Vesicle-associated membrane protein 4 OS=Mus musculus GN=Vamp4 PE=1 SV=1		9.929078	1	3	1	16.343	7.36	960000		590000	9.844923
Q9CZY3	Ubiquitin-conjugating enzyme E2 variant 1 OS=Mus musculus GN=Ube2v1 PE=1 SV=1		10.88435	1	3	1	16.344	7.96	1300000	1700000		9.919534
P14131	40S ribosomal protein S16 OS=Mus musculus GN=Rps16 PE=1 SV=4		39.0411	6	20	6	16.435	10.21	17000000	20000000	20000000	47.66302
Q60872	Eukaryotic translation initiation factor 1A OS=Mus musculus GN=Eif1a PE=2 SV=3		14.58333	2	5	2	16.492	5.24	26000000	14000000	13000000	15.11108
O70591	Prefoldin subunit 2 OS=Mus musculus GN=Pfdn2 PE=1 SV=2		7.792208	1	1	1	16.524	6.58		520000		3.018255
Q9D6K8	FUN14 domain-containing protein 2 OS=Mus musculus GN=Fundc2 PE=1 SV=1		15.23179	2	5	2	16.554	9.7	3100000	2700000	1400000	12.45938
O89086	RNA-binding protein 3 OS=Mus musculus GN=Rbm3 PE=1 SV=1		42.48366	6	18	6	16.595	7.5	19000000	17000000	15000000	53.72136
P14115	60S ribosomal protein L27a OS=Mus musculus GN=Rpl27a PE=1 SV=5		22.97297	4	13	4	16.595	11.12	32000000	28000000	30000000	36.86636
Q9CQI3	Glia maturation factor beta OS=Mus musculus GN=Gmfb PE=1 SV=3		14.08451	2	4	2	16.712	5.16	2100000	2900000	1500000	10.59421
P17897	Lysozyme C-1 OS=Mus musculus GN=Lyz1 PE=1 SV=1		8.108108	1	1	1	16.783	9.41			8000000	2.888494
P63242	Eukaryotic translation initiation factor 5A-1 OS=Mus musculus GN=Eif5a PE=1 SV=2		16.88312	2	5	2	16.821	5.24	5400000	2600000	1300000	18.96056
P62204	Calmodulin OS=Mus musculus GN=Calm1 PE=1 SV=2		41.61074	6	26	5	16.827	4.22	40000000	42000000	40000000	64.8691
Q9ERS2	NADH dehydrogenase [ubiquinone] 1 alpha subcomplex subunit 13 OS=Mus musculus GN=Ndufa13 PE=1 SV=3		18.05556	2	2	2	16.849	9.48	990000	4300000		5.44435
Q60605	Myosin light polypeptide 6 OS=Mus musculus GN=Myl6 PE=1 SV=3		46.35762	7	24	6	16.919	4.65	23000000	26000000	25000000	69.89691
Q9D898	Actin-related protein 2/3 complex subunit 5-like protein OS=Mus musculus GN=Arpc5l PE=1 SV=1		26.14379	2	2	2	16.97	6.8	3000000	1100000		7.942436
Q9CQ92	Mitochondrial fission 1 protein OS=Mus musculus GN=Fis1 PE=1 SV=1		17.10526	3	7	3	16.998	8.53	7500000	6100000	5300000	19.82989
P62743	AP-2 complex subunit sigma OS=Mus musculus GN=Ap2s1 PE=1 SV=1		12.67606	2	4	2	17.007	6.18	14000000	9500000		8.721389
P62843	40S ribosomal protein S15 OS=Mus musculus GN=Rps15 PE=1 SV=2		8.275862	1	8	1	17.029	10.39	14000000	2100000	11000000	26.44467
Q7TMF3	NADH dehydrogenase [ubiquinone] 1 alpha subcomplex subunit 12 OS=Mus musculus GN=Ndufa12 PE=1 SV=2		6.206897	1	2	1	17.076	9.36	10000000		7200000	4.246865
Q9CYL5	Golgi-associated plant pathogenesis-related protein 1 OS=Mus musculus GN=Glipr2 PE=1 SV=3		27.92208	3	7	3	17.08	9.51	4300000	3900000	4100000	25.08596
P61089	Ubiquitin-conjugating enzyme E2 N OS=Mus musculus GN=Ube2n PE=1 SV=1		7.236842	1	1	1	17.127	6.57	4700000			2.701234
Q923G2	DNA-directed RNA polymerases I, II, and III subunit RPABC3 OS=Mus musculus GN=Polr2h PE=1 SV=3		16	2	3	2	17.132	4.68		1400000	3000000	7.884645
P61327	Protein mago nashi homolog OS=Mus musculus GN=Magoh PE=2 SV=1		19.86301	3	8	3	17.153	6.11	9900000	6300000	5100000	21.15319
P15532	Nucleoside diphosphate kinase A OS=Mus musculus GN=Nme1 PE=1 SV=1		20.39474	3	6	1	17.197	7.37			4600000	13.59902
P62301	40S ribosomal protein S13 OS=Mus musculus GN=Rps13 PE=1 SV=2		33.77483	5	11	5	17.212	10.54	14000000	12000000	14000000	25.80446
Q9CRB2	H/ACA ribonucleoprotein complex subunit 2 OS=Mus musculus GN=Nhp2 PE=1 SV=1		18.95425	2	6	2	17.236	8.41	2600000	2300000	2200000	19.86506
P61255	60S ribosomal protein L26 OS=Mus musculus GN=Rpl26 PE=1 SV=1		17.24138	4	11	4	17.248	10.55	21000000	41000000	38000000	26.01002
Q9D3P8	Plasminogen receptor (KT) OS=Mus musculus GN=Plgrkt PE=1 SV=1		6.802721	1	1	1	17.25	9.5				2.236746
Q9CQH7	Transcription factor BTF3 homolog 4 OS=Mus musculus GN=Btf3l4 PE=1 SV=1		34.17722	3	7	3	17.26	6.35	3400000	5200000	3400000	22.17028
Q9CYR0	Single-stranded DNA-binding protein, mitochondrial OS=Mus musculus GN=Ssbp1 PE=1 SV=1		9.868421	1	6	1	17.308	9.92	4000000	3000000	2400000	20.63711
Q01768	Nucleoside diphosphate kinase B OS=Mus musculus GN=Nme2 PE=1 SV=1		23.02632	3	8	1	17.352	7.5	11000000	15000000	8900000	22.34031
O09111	NADH dehydrogenase [ubiquinone] 1 beta subcomplex subunit 11, mitochondrial OS=Mus musculus GN=Ndufb11 PE=1 SV=2		45.03311	5	22	5	17.433	5.22	7600000	6700000	5800000	66.05425
Q91VS7	Microsomal glutathione S-transferase 1 OS=Mus musculus GN=Mgst1 PE=1 SV=3		8.387097	1	4	1	17.54	9.66	5500000	5700000	4400000	9.991471
O55128	Histone deacetylase complex subunit SAP18 OS=Mus musculus GN=Sap18 PE=1 SV=1		7.843137	1	3	1	17.584	9.35	260000	2900000	2900000	8.702844
Q9CQL4	39S ribosomal protein L20, mitochondrial OS=Mus musculus GN=Mrpl20 PE=1 SV=1		14.09396	2	2	2	17.584	11.11		1200000		5.050592
Q9D3D9	ATP synthase subunit delta, mitochondrial OS=Mus musculus GN=Atp5d PE=1 SV=1		8.333333	1	3	1	17.589	5.08	860000	1900000	1300000	8.826919
P62751	60S ribosomal protein L23a OS=Mus musculus GN=Rpl23a PE=1 SV=1		34.61538	6	22	6	17.684	10.45	88000000	87000000	82000000	59.94338
P62270	40S ribosomal protein S18 OS=Mus musculus GN=Rps18 PE=1 SV=3		43.42105	8	22	8	17.708	10.99	37000000	36000000	31000000	59.13404
Q9D6U8	Protein FAM162A OS=Mus musculus GN=Fam162a PE=1 SV=1		10.96774	1	3	1	17.713	9.88	1600000	1900000		7.202684
Q8BP67	60S ribosomal protein L24 OS=Mus musculus GN=Rpl24 PE=1 SV=2		30.57325	5	13	5	17.768	11.25	10000000	12000000	6600000	35.79879
P35979	60S ribosomal protein L12 OS=Mus musculus GN=Rpl12 PE=1 SV=2		54.54545	6	22	6	17.794	9.42	25000000	17000000	25000000	75.11133
Q9ERR7	15 kDa selenoprotein OS=Mus musculus GN=Sep15 PE=1 SV=3		17.28395	2	4	2	17.796	5.35	3000000	1700000	2100000	10.98523
O88188	Lymphocyte antigen 86 OS=Mus musculus GN=Ly86 PE=1 SV=1		11.11111	1	2	1	17.8	5.22	830000	920000		5.080688
O54790	Transcription factor MafG OS=Mus musculus GN=Mafg PE=1 SV=1		4.320988	1	1	1	17.866	10.04		700000		1.945268
Q64339	Ubiquitin-like protein ISG15 OS=Mus musculus GN=Isg15 PE=1 SV=4		18.01242	2	7	2	17.886	7.9	8700000	9000000	6200000	22.43587
P62983	Ubiquitin-40S ribosomal protein S27a OS=Mus musculus GN=Rps27a PE=1 SV=2		51.92308	7	37	7	17.939	9.64	4.3E+08	3.8E+08	3.8E+08	121.9283
P17742	Peptidyl-prolyl cis-trans isomerase A OS=Mus musculus GN=Ppia PE=1 SV=2		30.4878	5	27	5	17.96	7.9	57000000	61000000	48000000	71.86454
P01592	Immunoglobulin J chain OS=Mus musculus GN=Jchain PE=1 SV=4		9.433962	1	4	1	18.002	4.89	860000		360000	9.566158
Q9CQ49	Nuclear cap-binding protein subunit 2 OS=Mus musculus GN=Ncbp2 PE=1 SV=1		7.051282	1	1	1	18.006	7.81		2400000		2.731159
P01831	Thy-1 membrane glycoprotein OS=Mus musculus GN=Thy1 PE=1 SV=1		31.48148	4	10	4	18.069	8.97	20000000	11000000	9400000	27.84378
P20801	Troponin C, skeletal muscle OS=Mus musculus GN=Tnnc2 PE=1 SV=2		9.375	2	4	1	18.098	4.2			870000	9.22579
P30355	Arachidonate 5-lipoxygenase-activating protein OS=Mus musculus GN=Alox5ap PE=1 SV=2		4.968944	1	1	1	18.124	8.47	4500000			1.867103

Q9D958	Signal peptidase complex subunit 1 OS=Mus musculus GN=Spcs1 PE=2 SV=3		7.453416	1	3	1	18.174	10.01	10000000	10000000	6600000	8.118435
P28574	Protein max OS=Mus musculus GN=Max PE=1 SV=1		6.25	1	3	1	18.186	5.87	610000	990000	920000	8.499267
O35912	Epithelial membrane protein 3 OS=Mus musculus GN=Emp3 PE=2 SV=2		4.907975	1	1	1	18.24	7.66	1100000			2.291501
Q9QUR7	Peptidyl-prolyl cis-trans isomerase NIMA-interacting 1 OS=Mus musculus GN=Pin1 PE=1 SV=1		12.12121	1	2	1	18.359	8.79		1100000	1000000	6.499946
P62281	40S ribosomal protein S11 OS=Mus musculus GN=Rps11 PE=1 SV=3		55.6962	10	26	10	18.419	10.3	39000000	43000000	39000000	65.32193
Q9D328	Transmembrane protein 35 OS=Mus musculus GN=Tmem35 PE=1 SV=1		8.982036	1	1	1	18.493	9.99	900000			2.331799
Q9R0P5	Destrin OS=Mus musculus GN=Dstn PE=1 SV=3		19.39394	3	10	3	18.509	7.97	6700000	5200000	6100000	32.65546
P18760	Cofilin-1 OS=Mus musculus GN=Cfl1 PE=1 SV=3		42.77108	6	20	6	18.548	8.09	53000000	49000000	38000000	79.28135
O09167	60S ribosomal protein L21 OS=Mus musculus GN=Rpl21 PE=1 SV=3		20	4	6	4	18.55	10.49	2600000	7200000	9300000	14.11063
Q64327	Male-enhanced antigen 1 OS=Mus musculus GN=Mea1 PE=1 SV=1		7.471264	1	2	1	18.573	4.08	700000	670000		5.759647
P60824	Cold-inducible RNA-binding protein OS=Mus musculus GN=Cirbp PE=1 SV=1		6.395349	1	1	1	18.596	9.61	1900000			2.522171
Q9WTX5	S-phase kinase-associated protein 1 OS=Mus musculus GN=Skp1 PE=1 SV=3		16.56442	4	11	4	18.66	4.54	7100000	6000000	4400000	33.7406
Q9R0Q7	Prostaglandin E synthase 3 OS=Mus musculus GN=Ptges3 PE=1 SV=1		38.75	5	12	5	18.709	4.55	5500000	16000000	4500000	39.40278
P61967	AP-1 complex subunit sigma-1A OS=Mus musculus GN=Ap1s1 PE=1 SV=1		10.12658	1	3	1	18.721	5.73	6000000	5700000	4900000	14.38324
Q9DCX2	ATP synthase subunit d, mitochondrial OS=Mus musculus GN=Atp5h PE=1 SV=3		29.19255	4	11	4	18.738	5.69	17000000	24000000	7000000	24.3462
O70492	Sorting nexin-3 OS=Mus musculus GN=Snx3 PE=1 SV=3		27.77778	5	11	3	18.746	8.66	3600000	2000000	2900000	29.57123
Q8K194	U4/U6.U5 small nuclear ribonucleoprotein 27 kDa protein OS=Mus musculus GN=Snrnp27 PE=1 SV=1		16.12903	2	4	2	18.874	11.62	1600000	2900000	2200000	10.35385
Q8R344	Coiled-coil domain-containing protein 12 OS=Mus musculus GN=Ccdc12 PE=1 SV=2		13.85542	1	1	1	18.88	7.21			890000	2.956863
P63325	40S ribosomal protein S10 OS=Mus musculus GN=Rps10 PE=1 SV=1		8.484848	1	3	1	18.904	10.15	24000000	24000000	17000000	12.42975
Q62186	Translocon-associated protein subunit delta OS=Mus musculus GN=Ssr4 PE=1 SV=1		25	3	12	3	18.924	5.78	5700000	7000000	4900000	33.77591
Q9CRB6	Tubulin polymerization-promoting protein family member 3 OS=Mus musculus GN=Tppp3 PE=1 SV=1		7.954545	1	3	1	18.954	9.11	1800000	890000	720000	10.65462
Q8C1M2	Zinc finger protein 428 OS=Mus musculus GN=Znf428 PE=1 SV=1		6.25	1	1	1	18.991	4.23			420000	2.584441
Q9CPP0	Nucleoplasmin-3 OS=Mus musculus GN=Npm3 PE=1 SV=3		8.571429	1	3	1	19.011	4.82		8900000	9200000	9.019164
O70493	Sorting nexin-12 OS=Mus musculus GN=Snx12 PE=1 SV=1		24.84848	4	6	2	19.104	7.34		1400000		16.23551
Q8R2Q8	Bone marrow stromal antigen 2 OS=Mus musculus GN=Bst2 PE=1 SV=1		15.69767	3	8	3	19.14	7.34	15000000	14000000	12000000	23.54575
Q63810	Calcineurin subunit B type 1 OS=Mus musculus GN=Ppp3r1 PE=1 SV=3		16.47059	2	5	2	19.288	4.81	3200000	1700000	2500000	12.05652
P84104	Serine/arginine-rich splicing factor 3 OS=Mus musculus GN=Srsf3 PE=1 SV=1		36.58537	6	22	5	19.318	11.65	17000000	20000000	14000000	64.00657
Q91WV0	Protein Dr1 OS=Mus musculus GN=Dr1 PE=1 SV=1		7.386364	1	2	1	19.419	4.75	2600000		2500000	5.797233
Q9CZH7	Matrix-remodeling-associated protein 7 OS=Mus musculus GN=Mxra7 PE=1 SV=2		8.988764	1	1	1	19.445	4.26		510000		3.140744
P63028	Translationally-controlled tumor protein OS=Mus musculus GN=Tpt1 PE=1 SV=1		55.81395	6	19	6	19.45	4.86	22000000	16000000	15000000	70.41846
Q9JIG8	PRA1 family protein 2 OS=Mus musculus GN=Praf2 PE=1 SV=1		6.179775	1	3	1	19.466	9.6	4900000	3100000	3000000	9.188937
Q9CR09	Ubiquitin-fold modifier-conjugating enzyme 1 OS=Mus musculus GN=Ufc1 PE=1 SV=1		4.790419	1	2	1	19.469	7.4	1200000	1100000		3.894522
Q8R2Y8	Peptidyl-tRNA hydrolase 2, mitochondrial OS=Mus musculus GN=Ptrh2 PE=1 SV=1		25.96685	3	6	3	19.514	7.42	2900000	2700000	2300000	19.54301
P19783	Cytochrome c oxidase subunit 4 isoform 1, mitochondrial OS=Mus musculus GN=Cox4i1 PE=1 SV=2		30.17751	5	17	5	19.518	9.23	30000000	33000000	16000000	48.90464
Q9D938	Transmembrane protein 160 OS=Mus musculus GN=Tmem160 PE=2 SV=1		10.6383	2	2	2	19.575	7.3	1800000		520000	4.60522
P53996	Cellular nucleic acid-binding protein OS=Mus musculus GN=Cnbp PE=1 SV=2		6.741573	1	1	1	19.579	7.58		2500000		2.624178
Q59J78	Mimitin, mitochondrial OS=Mus musculus GN=Ndufaf2 PE=1 SV=1		9.52381	1	1	1	19.616	8.25	5000000			2.5943
Q99LW6	YY1-associated factor 2 OS=Mus musculus GN=Yaf2 PE=1 SV=1		13.40782	1	3	1	19.643	9.58	970000	990000	830000	9.751854
P59999	Actin-related protein 2/3 complex subunit 4 OS=Mus musculus GN=Arpc4 PE=1 SV=3		17.85714	4	10	4	19.654	8.43	13000000	12000000	11000000	26.01749
Q3THE2	Myosin regulatory light chain 12B OS=Mus musculus GN=Myl12b PE=1 SV=2		34.88372	5	19	5	19.767	4.84	52000000	45000000	50000000	57.95821
Q9CXZ1	NADH dehydrogenase [ubiquinone] iron-sulfur protein 4, mitochondrial OS=Mus musculus GN=Ndufs4 PE=1 SV=3		13.71429	2	6	2	19.772	9.99	4900000	2700000	3100000	15.85791
Q9CWZ3	RNA-binding protein 8A OS=Mus musculus GN=Rbm8a PE=1 SV=3		21.83908	3	15	3	19.876	6.16	17000000	17000000	16000000	50.3733
Q9DCJ5	NADH dehydrogenase [ubiquinone] 1 alpha subcomplex subunit 8 OS=Mus musculus GN=Ndufa8 PE=1 SV=3		12.2093	2	2	2	19.979	8.46	960000	7700000		5.151415
Q9R0P4	Small acidic protein OS=Mus musculus GN=Smap PE=1 SV=1		21.54696	2	10	2	20.034	4.82	5800000	4600000	4400000	38.19312
P62331	ADP-ribosylation factor 6 OS=Mus musculus GN=Arf6 PE=1 SV=2		6.285714	1	3	1	20.069	8.95	4700000	4300000	3700000	8.24675
Q9CWU4	UPF0690 protein C1orf52 homolog OS=Mus musculus PE=1 SV=1		9.444444	1	1	1	20.089	5.11	260000			2.236649
Q9CZX9	ER membrane protein complex subunit 4 OS=Mus musculus GN=Emc4 PE=1 SV=1		8.743169	1	2	1	20.103	8.62		1900000	1200000	4.953184
Q9D365	Signal peptidase complex subunit 3 OS=Mus musculus GN=Spcs3 PE=1 SV=1		6.666667	1	1	1	20.134	9.6		1100000		2.738819
P28667	MARCKS-related protein OS=Mus musculus GN=Marcksl1 PE=1 SV=2		65	5	10	5	20.153	4.61	5500000	3500000	2500000	37.24055
P61924	Coatomer subunit zeta-1 OS=Mus musculus GN=Copz1 PE=1 SV=1		16.94915	2	2	2	20.185	4.81	470000		5000000	4.879141
Q9CXW4	60S ribosomal protein L11 OS=Mus musculus GN=Rpl11 PE=1 SV=4		16.85393	3	9	3	20.24	9.6	23000000	23000000	23000000	24.28854
O55013	Trafficking protein particle complex subunit 3 OS=Mus musculus GN=Trappc3 PE=1 SV=1		4.444444	1	3	1	20.289	4.96	910000	690000	670000	5.872689
Q8VE22	28S ribosomal protein S23, mitochondrial OS=Mus musculus GN=Mrps23 PE=1 SV=1		7.909605	1	3	1	20.335	8.59	1600000	1800000	1000000	8.812244

Q9CXI5	Mesencephalic astrocyte-derived neurotrophic factor OS=Mus musculus GN=Manf PE=1 SV=1		26.81564	4	10	4	20.361	8.07	6700000	5600000	5000000	33.39121
P61750	ADP-ribosylation factor 4 OS=Mus musculus GN=Arf4 PE=1 SV=2		13.88889	2	6	2	20.384	7.14	4600000	4800000	3300000	19.55995
P61211	ADP-ribosylation factor-like protein 1 OS=Mus musculus GN=Arl1 PE=1 SV=1		7.734807	1	2	1	20.398	5.72	2300000	2400000		7.705759
Q9QZ88	Vacuolar protein sorting-associated protein 29 OS=Mus musculus GN=Vps29 PE=1 SV=1		10.98901	2	4	2	20.483	6.79	15000000	9800000	10000000	11.03608
P61226	Ras-related protein Rap-2b OS=Mus musculus GN=Rap2b PE=1 SV=1		24.59016	4	7	3	20.491	4.81	700000	910000		17.7564
Q9JM76	Actin-related protein 2/3 complex subunit 3 OS=Mus musculus GN=Arpc3 PE=1 SV=3		7.303371	1	3	1	20.511	8.59	2500000	2800000	1700000	12.09214
P84084	ADP-ribosylation factor 5 OS=Mus musculus GN=Arf5 PE=1 SV=2		13.88889	2	6	1	20.517	6.79	1700000	2200000	1600000	19.89386
P55002	Microfibrillar-associated protein 2 OS=Mus musculus GN=Mfap2 PE=2 SV=1		5.464481	1	1	1	20.565	4.89			190000	2.281886
P97315	Cysteine and glycine-rich protein 1 OS=Mus musculus GN=Csrp1 PE=1 SV=3		39.37824	5	10	5	20.57	8.57	9300000	6100000	7200000	28.00432
P61205	ADP-ribosylation factor 3 OS=Mus musculus GN=Arf3 PE=2 SV=2		18.23204	3	5	2	20.588	7.43	17000000	5900000		14.95505
Q3UHX2	28 kDa heat- and acid-stable phosphoprotein OS=Mus musculus GN=Pdap1 PE=1 SV=1		32.59669	5	17	5	20.593	7.39	14000000	12000000	12000000	63.74282
Q9Z0S9	Prenylated Rab acceptor protein 1 OS=Mus musculus GN=Rabac1 PE=1 SV=1		8.108108	1	1	1	20.606	7.9	720000			2.304029
Q9R0P6	Signal peptidase complex catalytic subunit SEC11A OS=Mus musculus GN=Sec11a PE=1 SV=1		9.497207	2	4	2	20.613	9.33	2300000	1500000	2000000	8.742439
P62717	60S ribosomal protein L18a OS=Mus musculus GN=Rpl18a PE=1 SV=1		28.40909	5	9	5	20.719	10.71	21000000	14000000	11000000	30.21056
Q8BU31	Ras-related protein Rap-2c OS=Mus musculus GN=Rap2c PE=1 SV=1		14.20765	2	2	1	20.731	4.94		480000		5.023464
Q9CQ80	Vacuolar protein-sorting-associated protein 25 OS=Mus musculus GN=Vps25 PE=1 SV=1		4.545455	1	1	1	20.735	6.34		920000		2.210815
Q9QXT0	Protein canopy homolog 2 OS=Mus musculus GN=Cnpy2 PE=1 SV=1		15.38462	2	6	2	20.754	5.07	1300000	710000	1300000	16.7466
P29391	Ferritin light chain 1 OS=Mus musculus GN=Ftl1 PE=1 SV=2		28.4153	3	6	3	20.789	6	3000000	3300000	1800000	21.4364
Q99JI6	Ras-related protein Rap-1b OS=Mus musculus GN=Rap1b PE=1 SV=2		33.15217	8	26	3	20.812	5.78	15000000	19000000	4600000	73.27667
P55821	Stathmin-2 OS=Mus musculus GN=Stmn2 PE=1 SV=1		5.586592	1	3	1	20.815	8.32	9900000	8300000	8200000	7.586367
P70296	Phosphatidylethanolamine-binding protein 1 OS=Mus musculus GN=Pebp1 PE=1 SV=3		6.951872	1	3	1	20.817	5.4	2500000	3000000	2300000	10.32768
P23198	Chromobox protein homolog 3 OS=Mus musculus GN=Cbx3 PE=1 SV=2		36.61202	7	18	5	20.842	5.22	56000000	53000000	55000000	68.61187
P10922	Histone H1.0 OS=Mus musculus GN=H1f0 PE=2 SV=4		16.49485	3	11	3	20.848	10.9	19000000	19000000	15000000	32.79523
Q8C8T8	Pre-rRNA-processing protein TSR2 homolog OS=Mus musculus GN=Tsr2 PE=1 SV=1		6.806283	1	1	1	20.878	4.23	780000			3.319439
P61082	NEDD8-conjugating enzyme Ubc12 OS=Mus musculus GN=Ube2m PE=1 SV=1		4.918033	1	3	1	20.887	7.69	2600000	3300000	2800000	6.486334
P97314	Cysteine and glycine-rich protein 2 OS=Mus musculus GN=Csrp2 PE=1 SV=3		7.772021	1	1	1	20.912	8.62	660000			1.946186
P62835	Ras-related protein Rap-1A OS=Mus musculus GN=Rap1a PE=1 SV=1		25	6	21	1	20.974	6.67	16000000	13000000	12000000	57.33875
Q9DCS9	NADH dehydrogenase [ubiquinone] 1 beta subcomplex subunit 10 OS=Mus musculus GN=Ndufb10 PE=1 SV=3		24.43182	3	6	3	21.01	8.03	8900000	3300000	2400000	20.62638
Q60870	Receptor expression-enhancing protein 5 OS=Mus musculus GN=Reep5 PE=1 SV=1		5.405405	1	4	1	21.037	8.38	6600000	7300000	4400000	14.55217
Q9DCF9	Translocon-associated protein subunit gamma OS=Mus musculus GN=Ssr3 PE=1 SV=1		7.567568	1	5	1	21.051	9.61	12000000	1900000	11000000	18.63968
P09528	Ferritin heavy chain OS=Mus musculus GN=Fth1 PE=1 SV=2		32.41758	5	19	5	21.053	5.88	6400000	8100000	2900000	55.89722
O09114	Prostaglandin-H2 D-isomerase OS=Mus musculus GN=Ptgds PE=1 SV=1		8.994709	1	1	1	21.053	8.25			550000	2.58893
Q9CR27	WASH complex subunit CCDC53 OS=Mus musculus GN=Ccdc53 PE=1 SV=1		4.123711	1	1	1	21.08	4.46			210000	1.945026
Q9CPT5	Nucleolar protein 16 OS=Mus musculus GN=Nop16 PE=1 SV=1		4.494382	1	1	1	21.126	9.91	1200000			1.989397
Q921L3	Calcium load-activated calcium channel OS=Mus musculus GN=Tmco1 PE=1 SV=1		7.446809	1	2	1	21.161	9.74	2100000	740000		5.210736
Q91XE8	Transmembrane protein 205 OS=Mus musculus GN=Tmem205 PE=1 SV=1		8.994709	1	1	1	21.166	9.42		1600000		3.167513
P08556	GTPase NRas OS=Mus musculus GN=Nras PE=1 SV=1		19.57672	3	9	2	21.186	5.17	3900000	2800000	2200000	26.84037
P60766	Cell division control protein 42 homolog OS=Mus musculus GN=Cdc42 PE=1 SV=2		26.17801	4	11	3	21.245	6.55	1500000	4100000	3000000	31.68808
Q8K2H6	Anaphase-promoting complex subunit 10 OS=Mus musculus GN=Anapc10 PE=1 SV=1		6.486486	1	1	1	21.253	9.06			150000	1.977301
P84096	Rho-related GTP-binding protein RhoG OS=Mus musculus GN=Rhog PE=1 SV=1		45.54974	7	16	6	21.295	8.12	7000000	8200000	5200000	54.32089
Q8BK08	Transmembrane protein 11, mitochondrial OS=Mus musculus GN=Tmem11 PE=1 SV=1		4.736842	1	2	1	21.298	7.36	3400000	2300000		4.328761
P52482	Ubiquitin-conjugating enzyme E2 E1 OS=Mus musculus GN=Ube2e1 PE=1 SV=1		9.326425	1	1	1	21.32	8.53	950000			2.960333
P60764	Ras-related C3 botulinum toxin substrate 3 OS=Mus musculus GN=Rac3 PE=1 SV=1		9.895833	2	6	1	21.365	8.15	14000000	9600000	10000000	16.49901
Q8VEH3	ADP-ribosylation factor-like protein 8A OS=Mus musculus GN=Arl8a PE=1 SV=1		18.27957	3	10	2	21.376	7.77	5700000	4900000	3900000	29.21405
P83917	Chromobox protein homolog 1 OS=Mus musculus GN=Cbx1 PE=1 SV=1		30.81081	5	18	4	21.405	4.93	10000000	11000000	10000000	62.92983
Q9CPR4	60S ribosomal protein L17 OS=Mus musculus GN=Rpl17 PE=1 SV=3		22.82609	4	14	4	21.409	10.18	26000000	37000000	39000000	35.36381
Q9D291	Desumoylating isopeptidase 2 OS=Mus musculus GN=Desi2 PE=1 SV=1		8.247423	1	1	1	21.42	4.92				2.135327
O35988	Syndecan-4 OS=Mus musculus GN=Sdc4 PE=1 SV=1		12.12121	2	3	2	21.469	4.41	3200000	1900000		9.548775
Q9BCZ4	Selenoprotein S OS=Mus musculus GN=Vimp PE=1 SV=3		11.57895	2	4	2	21.497	9.39	680000	730000	910000	9.702255
P51910	Apolipoprotein D OS=Mus musculus GN=Apod PE=1 SV=1		6.878307	1	5	1	21.516	4.91	4300000	11000000	10000000	15.41338
Q9CQW2	ADP-ribosylation factor-like protein 8B OS=Mus musculus GN=Arl8b PE=1 SV=1		9.677419	2	6	1	21.525	8.43	18000000	13000000	12000000	14.37278
Q8R5J9	PRA1 family protein 3 OS=Mus musculus GN=Arl6ip5 PE=1 SV=2		5.851064	1	3	1	21.543	9.61	7900000	6000000	5700000	8.435929
P35980	60S ribosomal protein L18 OS=Mus musculus GN=Rpl18 PE=1 SV=3		33.51064	6	18	6	21.631	11.78	63000000	54000000	57000000	55.49859

Q2VPQ9	Chromatin modification-related protein MEAF6 OS=Mus musculus GN=Meaf6 PE=1 SV=1		9.947644	2	3	2	21.636	9.32	500000	1900000	790000	7.156549
P32883	GTPase KRas OS=Mus musculus GN=Kras PE=1 SV=1		11.11111	2	4	1	21.642	6.77	2100000			9.864336
O55022	Membrane-associated progesterone receptor component 1 OS=Mus musculus GN=Pgrmc1 PE=1 SV=4		27.69231	5	15	4	21.681	4.7	4600000	3700000	2400000	46.13694
Q9DB15	39S ribosomal protein L12, mitochondrial OS=Mus musculus GN=Mrpl12 PE=1 SV=2		6.965174	1	2	1	21.695	9.29				7.444701
Q9CQH3	NADH dehydrogenase [ubiquinone] 1 beta subcomplex subunit 5, mitochondrial OS=Mus musculus GN=Ndufb5 PE=1 SV=1		21.16402	4	10	4	21.696	9.41	6600000	6100000	3500000	26.50757
Q9DCR2	AP-3 complex subunit sigma-1 OS=Mus musculus GN=Ap3s1 PE=1 SV=2		13.4715	2	4	2	21.718	5.39		1400000	1200000	10.84594
Q9DCI9	39S ribosomal protein L32, mitochondrial OS=Mus musculus GN=Mrpl32 PE=1 SV=1		6.417112	1	1	1	21.719	9.7		1600000		2.271377
Q921Y2	U3 small nucleolar ribonucleoprotein protein IMP3 OS=Mus musculus GN=Imp3 PE=2 SV=1		8.152174	1	1	1	21.763	9.5	1500000			2.432849
Q61171	Peroxiredoxin-2 OS=Mus musculus GN=Prdx2 PE=1 SV=3		4.040404	1	1	1	21.765	5.41				1.841062
Q9QUI0	Transforming protein RhoA OS=Mus musculus GN=Rhoa PE=1 SV=1		29.53368	6	10	6	21.768	6.1	14000000	1600000	9800000	26.86607
P43275	Histone H1.1 OS=Mus musculus GN=Hist1h1a PE=1 SV=2		10.32864	3	6	1	21.772	10.93		27000000		16.90028
P35285	Ras-related protein Rab-22A OS=Mus musculus GN=Rab22a PE=1 SV=2		5.670103	1	1	1	21.788	8.15		740000		2.101998
P12815	Programmed cell death protein 6 OS=Mus musculus GN=Pdcd6 PE=1 SV=2		5.759162	1	2	1	21.854	5.4				5.122939
Q9D6J5	NADH dehydrogenase [ubiquinone] 1 beta subcomplex subunit 8, mitochondrial OS=Mus musculus GN=Ndufb8 PE=1 SV=1		17.74194	3	6	3	21.862	6.64	3900000	3700000	460000	14.00245
P51410	60S ribosomal protein L9 OS=Mus musculus GN=Rpl9 PE=2 SV=2		11.45833	2	5	2	21.868	9.95	43000000	23000000	43000000	13.50202
P99029	Peroxiredoxin-5, mitochondrial OS=Mus musculus GN=Prdx5 PE=1 SV=2		11.90476	2	5	2	21.884	8.85	4200000	3400000	5900000	14.10474
P43274	Histone H1.4 OS=Mus musculus GN=Hist1h1e PE=1 SV=2		15.52511	4	16	1	21.964	11.11	1.3E+08	1.2E+08	1.1E+08	42.91799
Q9WTQ8	Mitochondrial import inner membrane translocase subunit Tim23 OS=Mus musculus GN=Timm23 PE=1 SV=1		8.133971	1	3	1	21.964	9.13	1000000	810000	820000	11.10538
Q9CQJ8	NADH dehydrogenase [ubiquinone] 1 beta subcomplex subunit 9 OS=Mus musculus GN=Ndufb9 PE=1 SV=3		39.6648	4	7	4	21.97	7.8	2400000	1700000	2300000	22.56844
Q64152	Transcription factor BTF3 OS=Mus musculus GN=Btf3 PE=1 SV=3		12.2549	1	6	1	22.017	9.52	7000000	7600000	5300000	30.32412
Q08024	Core-binding factor subunit beta OS=Mus musculus GN=Cbfb PE=1 SV=1		17.64706	2	4	2	22.017	5.77	1000000	1200000	1200000	15.78013
Q91XV3	Brain acid soluble protein 1 OS=Mus musculus GN=Basp1 PE=1 SV=3		71.68142	11	57	11	22.074	4.51	1.1E+08	71000000	64000000	225.0312
P43277	Histone H1.3 OS=Mus musculus GN=Hist1h1d PE=1 SV=2		15.38462	4	17	1	22.086	11.03	1.7E+08	1.8E+08	1.5E+08	44.33175
P60904	DnaJ homolog subfamily C member 5 OS=Mus musculus GN=Dnajc5 PE=1 SV=1		24.24242	2	7	2	22.086	5.07	13000000	4800000	6000000	21.10595
Q60771	Claudin-11 OS=Mus musculus GN=Cldn11 PE=1 SV=1		6.763285	1	2	1	22.099	7.91	9900000	4300000		5.365395
P62082	40S ribosomal protein S7 OS=Mus musculus GN=Rps7 PE=2 SV=1		12.8866	3	7	3	22.113	10.1	19000000	21000000	16000000	15.94266
Q9D1M7	Peptidyl-prolyl cis-trans isomerase FKBP11 OS=Mus musculus GN=Fkbp11 PE=1 SV=1		4.975124	1	3	1	22.123	9.36	3000000	2300000	2400000	7.640538
P62761	Visinin-like protein 1 OS=Mus musculus GN=Vsnl1 PE=1 SV=2		26.70157	5	20	4	22.128	5.15	13000000	10000000	6200000	49.47676
Q9DBP5	UMP-CMP kinase OS=Mus musculus GN=Cmpk1 PE=1 SV=1		5.612245	1	2	1	22.151	5.83	1500000	1100000		4.432056
Q9CQJ6	Density-regulated protein OS=Mus musculus GN=Denr PE=1 SV=1		21.21212	3	7	3	22.152	5.3	3200000	3700000	2000000	24.4185
P35700	Peroxiredoxin-1 OS=Mus musculus GN=Prdx1 PE=1 SV=1		55.77889	10	30	8	22.162	8.12	16000000	16000000	13000000	77.1825
Q61686	Chromobox protein homolog 5 OS=Mus musculus GN=Cbx5 PE=1 SV=1		43.4555	8	28	7	22.172	5.86	23000000	25000000	26000000	83.12839
Q9D1G1	Ras-related protein Rab-1B OS=Mus musculus GN=Rab1b PE=1 SV=1		30.34826	5	14	2	22.173	5.73	4400000	7800000	3700000	38.71562
Q8BGZ1	Hippocalcin-like protein 4 OS=Mus musculus GN=Hpcal4 PE=1 SV=3		15.70681	3	7	2	22.201	4.89	6800000	3200000	4700000	19.00417
Q9D0B6	Protein PBDC1 OS=Mus musculus GN=Pbdc1 PE=1 SV=1		13.63636	2	3	2	22.21	4.55	1200000		960000	9.097236
Q91X97	Neurocalcin-delta OS=Mus musculus GN=Ncald PE=1 SV=4		22.27979	4	8	2	22.231	5.35	2500000			20.33552
Q9D6N5	Dr1-associated corepressor OS=Mus musculus GN=Drap1 PE=1 SV=3		15.60976	2	6	2	22.264	5.26	2800000	3100000	2800000	18.68966
P11352	Glutathione peroxidase 1 OS=Mus musculus GN=Gpx1 PE=1 SV=2		13.43284	2	8	2	22.316	7.21	5100000	4900000	2900000	22.53582
P62748	Hippocalcin-like protein 1 OS=Mus musculus GN=Hpcal1 PE=1 SV=2		9.326425	2	6	1	22.324	5.5	980000	660000	580000	13.09776
P36536	GTP-binding protein SAR1a OS=Mus musculus GN=Sar1a PE=1 SV=1		9.59596	2	6	2	22.357	6.93	2200000	1800000	1500000	13.54125
Q9WVA4	Transgelin-2 OS=Mus musculus GN=Tagln2 PE=1 SV=4		24.62312	4	8	4	22.381	8.24	2100000	4000000	1500000	23.67449
P61087	Ubiquitin-conjugating enzyme E2 K OS=Mus musculus GN=Ube2k PE=1 SV=3		6	1	3	1	22.393	5.44	970000	1100000	820000	8.609021
P09542	Myosin light chain 3 OS=Mus musculus GN=Myl3 PE=1 SV=4		9.803922	2	7	1	22.407	5.1	8900000	12000000	5500000	19.80314
P84075	Neuron-specific calcium-binding protein hippocalcin OS=Mus musculus GN=Hpca PE=1 SV=2		16.06218	3	8	1	22.413	4.97	2000000	1500000		18.46214
Q9JMG7	Hepatoma-derived growth factor-related protein 3 OS=Mus musculus GN=Hdgfrp3 PE=1 SV=2		7.920792	1	3	1	22.417	8.4	4300000	3100000	2500000	9.495946
P61759	Prefoldin subunit 3 OS=Mus musculus GN=Vbp1 PE=1 SV=2		9.693878	2	3	2	22.421	6.28		2500000		6.031665
Q8R0A5	Transcription elongation factor A protein-like 3 OS=Mus musculus GN=Tceal3 PE=1 SV=2		11	1	3	1	22.455	5.44	1100000	1200000	460000	9.264688
Q9R1Q8	Transgelin-3 OS=Mus musculus GN=Tagln3 PE=1 SV=1		13.06533	3	7	3	22.456	7.33	1300000	860000	1100000	15.88944
P37217	Early activation antigen CD69 OS=Mus musculus GN=Cd69 PE=2 SV=1		8.040201	1	1	1	22.503	7.02		940000		3.348228
P61027	Ras-related protein Rab-10 OS=Mus musculus GN=Rab10 PE=1 SV=1		37	7	20	5	22.527	8.38	11000000	10000000	8700000	55.46659
P43276	Histone H1.5 OS=Mus musculus GN=Hist1h1b PE=1 SV=2		15.24664	4	14	4	22.562	10.92	36000000	39000000	37000000	33.79545
Q6ZWN5	40S ribosomal protein S9 OS=Mus musculus GN=Rps9 PE=1 SV=3		34.02062	8	23	8	22.578	10.65	56000000	52000000	57000000	55.32329
Q9DCZ4	MICOS complex subunit Mic26 OS=Mus musculus GN=Apoo PE=1 SV=2		13.13131	2	2	2	22.59	9.25	4400000	7000000		6.168548

P62821	Ras-related protein Rab-1A OS=Mus musculus GN=Rab1A PE=1 SV=3		36.58537	6	23	3	22.663	6.21	7400000	6000000	5400000	73.06267
Q9CQ88	Tetraspanin-31 OS=Mus musculus GN=Tspan31 PE=1 SV=1		5.714286	1	1	1	22.679	8.44		1300000		2.238981
Q9R0Q3	Transmembrane emp24 domain-containing protein 2 OS=Mus musculus GN=Tmed2 PE=1 SV=1		10.44776	2	8	2	22.69	5.17	7600000	6700000	16000000	19.34628
Q673H1	Tumor suppressor candidate gene 1 protein homolog OS=Mus musculus GN=Tusc1 PE=1 SV=1		7.804878	1	1	1	22.699	11.09			1300000	2.336337
Q9DCT8	Cysteine-rich protein 2 OS=Mus musculus GN=Crip2 PE=1 SV=1		7.692308	1	2	1	22.712	8.63	360000	310000		5.049096
P63139	Nuclear transcription factor Y subunit beta OS=Mus musculus GN=Nfyb PE=1 SV=1		6.280193	1	2	1	22.773	4.59		340000	560000	5.206454
P30412	Peptidyl-prolyl cis-trans isomerase C OS=Mus musculus GN=Ppic PE=1 SV=1		6.132075	1	3	1	22.78	7.5	870000	820000	650000	7.630697
Q61599	Rho GDP-dissociation inhibitor 2 OS=Mus musculus GN=Arhgdib PE=1 SV=3		8	1	2	1	22.836	5.11	3200000	2800000		6.983486
P11672	Neutrophil gelatinase-associated lipocalin OS=Mus musculus GN=Lcn2 PE=1 SV=1		3.5	1	1	1	22.861	8.81			3300000	2.345358
P97461	40S ribosomal protein S5 OS=Mus musculus GN=Rps5 PE=1 SV=3		9.313725	3	8	3	22.875	9.72	18000000	11000000	8600000	20.55226
Q9R1P3	Proteasome subunit beta type-2 OS=Mus musculus GN=Psmb2 PE=1 SV=1		11.44279	2	5	2	22.892	7.02	2400000	2200000	1200000	14.83942
Q9R0M6	Ras-related protein Rab-9A OS=Mus musculus GN=Rab9a PE=1 SV=1		6.467662	1	2	1	22.895	5.66	660000	580000		5.311714
Q921Z5	Tumor necrosis factor alpha-induced protein 8 OS=Mus musculus GN=Tnfaip8 PE=1 SV=1		5.050505	1	1	1	22.946	7.93		890000		2.751731
Q9R1P1	Proteasome subunit beta type-3 OS=Mus musculus GN=Psmb3 PE=1 SV=1		4.878049	1	1	1	22.949	6.55	1100000			2.440578
Q9CQU3	Protein RER1 OS=Mus musculus GN=Rer1 PE=1 SV=1		9.183673	1	6	1	22.973	9.51	6900000	6700000	6900000	20.42805
O54879	High mobility group protein B3 OS=Mus musculus GN=Hmgb3 PE=1 SV=3		17.5	2	6	2	22.995	8.37	4400000	4100000	6700000	20.3393
Q6PHN9	Ras-related protein Rab-35 OS=Mus musculus GN=Rab35 PE=1 SV=1		15.42289	3	8	1	23.011	8.29	16000000		12000000	20.41235
P35293	Ras-related protein Rab-18 OS=Mus musculus GN=Rab18 PE=1 SV=2		32.03883	5	11	5	23.021	5.36	8700000	7200000	5600000	35.59102
P52651	Homeobox protein Rhox5 OS=Mus musculus GN=Rhox5 PE=2 SV=1		12.85714	1	1	1	23.031	6.25	510000			4.884413
Q9JM14	5'(3')-deoxyribonucleotidase, cytosolic type OS=Mus musculus GN=Nt5c PE=1 SV=1		6.5	1	1	1	23.062	5.49		530000		2.417355
O09044	Synaptosomal-associated protein 23 OS=Mus musculus GN=Snap23 PE=1 SV=1		12.85714	2	7	1	23.246	4.98	2600000	3500000		20.94213
O08734	Bcl-2 homologous antagonist/killer OS=Mus musculus GN=Bak1 PE=1 SV=3		11.00478	1	2	1	23.28	6.18		1500000	1300000	5.958138
P60879	Synaptosomal-associated protein 25 OS=Mus musculus GN=Snap25 PE=1 SV=1		30.09709	5	20	4	23.3	4.77	16000000	10000000	6500000	65.5112
Q80UU9	Membrane-associated progesterone receptor component 2 OS=Mus musculus GN=Pgrmc2 PE=1 SV=2		38.24885	5	15	4	23.32	5.15	3400000	4500000	3900000	45.40846
Q9CTH6	rRNA-processing protein FCF1 homolog OS=Mus musculus GN=Fcf1 PE=2 SV=2		10.10101	2	2	2	23.325	9.67			540000	3.958102
Q9JIW9	Ras-related protein Ral-B OS=Mus musculus GN=Ralb PE=1 SV=1		4.368932	1	2	1	23.335	6.62	370000	410000		4.0597
Q9DB20	ATP synthase subunit O, mitochondrial OS=Mus musculus GN=Atp5o PE=1 SV=1		20.65728	3	5	3	23.349	9.99	2500000	1500000	1100000	13.17522
Q9DD18	D-tyrosyl-tRNA(Tyr) deacylase 1 OS=Mus musculus GN=Dtd1 PE=1 SV=2		7.655502	1	2	1	23.37	7.87	1500000		1100000	7.712435
P28076	Proteasome subunit beta type-9 OS=Mus musculus GN=Psmb9 PE=1 SV=1		4.109589	1	2	1	23.382	5.22	6000000	5500000		4.086198
P62071	Ras-related protein R-Ras2 OS=Mus musculus GN=Rras2 PE=1 SV=1		20.09804	3	9	3	23.385	6.01	2000000	1900000	2200000	26.08617
Q99PT1	Rho GDP-dissociation inhibitor 1 OS=Mus musculus GN=Arhgdia PE=1 SV=3		25.4902	4	5	4	23.393	5.2	820000	4400000	3700000	12.31988
P35278	Ras-related protein Rab-5C OS=Mus musculus GN=Rab5c PE=1 SV=2		21.2963	4	12	2	23.398	8.41	16000000	20000000	20000000	33.94156
P0C0A3	Charged multivesicular body protein 6 OS=Mus musculus GN=Chmp6 PE=1 SV=2		19.5	3	4	3	23.401	5.44	1100000	1700000	2600000	12.39675
P19253	60S ribosomal protein L13a OS=Mus musculus GN=Rpl13a PE=1 SV=4		20.68966	4	20	4	23.449	11.02	48000000	37000000	34000000	50.14922
P84099	60S ribosomal protein L19 OS=Mus musculus GN=Rpl19 PE=1 SV=1		13.26531	2	8	2	23.451	11.47	71000000	70000000	68000000	28.03722
Q9CY66	H/ACA ribonucleoprotein complex subunit 1 OS=Mus musculus GN=Gar1 PE=1 SV=1		3.896104	1	2	1	23.46	11.02		1000000	1000000	4.140521
P51150	Ras-related protein Rab-7a OS=Mus musculus GN=Rab7a PE=1 SV=2		47.82609	8	27	8	23.475	6.7	16000000	14000000	13000000	89.88087
P53994	Ras-related protein Rab-2A OS=Mus musculus GN=Rab2a PE=1 SV=1		41.98113	7	15	7	23.533	6.54	16000000	19000000	9000000	46.06519
P63321	Ras-related protein Ral-A OS=Mus musculus GN=Rala PE=1 SV=1		13.1068	2	4	2	23.538	7.11	13000000	8900000	6500000	14.80858
Q9CR57	60S ribosomal protein L14 OS=Mus musculus GN=Rpl14 PE=1 SV=3		19.81567	4	14	4	23.549	11.02	20000000	20000000	19000000	36.7923
Q7TQD2	Tubulin polymerization-promoting protein OS=Mus musculus GN=Tppp PE=1 SV=1		12.38532	2	3	2	23.56	9.42	2500000	3200000		8.535874
Q8BR63	Protein FAM177A1 OS=Mus musculus GN=Fam177a1 PE=1 SV=1		12.07729	2	2	2	23.563	4.59	1600000			4.208966
P35279	Ras-related protein Rab-6A OS=Mus musculus GN=Rab6a PE=1 SV=4		23.07692	4	10	3	23.575	5.54	4000000	4300000	3800000	27.10963
P34022	Ran-specific GTPase-activating protein OS=Mus musculus GN=Ranbp1 PE=1 SV=2		27.58621	3	10	3	23.582	5.22	10000000	10000000	9500000	31.88674
Q9CQD1	Ras-related protein Rab-5A OS=Mus musculus GN=Rab5a PE=1 SV=1		21.39535	4	10	2	23.584	8.15	4200000	8200000		27.63569
P61028	Ras-related protein Rab-8B OS=Mus musculus GN=Rab8b PE=1 SV=1		23.18841	4	10	2	23.588	9.07	2000000	1400000	1100000	28.90488
P19157	Glutathione S-transferase P 1 OS=Mus musculus GN=Gstp1 PE=1 SV=2		20.47619	3	6	3	23.594	7.87	2800000	3300000	4800000	21.09103
Q9CPW7	Zinc finger matrin-type protein 2 OS=Mus musculus GN=Zmat2 PE=2 SV=1		6.030151	1	1	1	23.597	9.01		1000000		2.371313
P06837	Neuromodulin OS=Mus musculus GN=Gap43 PE=1 SV=1		39.64758	6	17	6	23.618	4.73	18000000	8500000	6600000	55.4507
Q91V08	C-type lectin domain family 2 member D OS=Mus musculus GN=Clec2d PE=1 SV=1		5.797101	1	1	1	23.631	7.62	730000			2.463006
P55258	Ras-related protein Rab-8A OS=Mus musculus GN=Rab8a PE=1 SV=2		17.3913	3	7	1	23.653	9.07	410000			18.6091
P61021	Ras-related protein Rab-5B OS=Mus musculus GN=Rab5b PE=1 SV=1		15.81395	3	7	1	23.692	8.13		2700000		18.99394
P24369	Peptidyl-prolyl cis-trans isomerase B OS=Mus musculus GN=Ppib PE=1 SV=2		42.12963	10	31	10	23.699	9.55	39000000	31000000	25000000	87.11706

Q7TMY4	THO complex subunit 7 homolog OS=Mus musculus GN=Thoc7 PE=1 SV=2		8.823529	1	1	1	23.7	5.67	850000			2.933666
Q62422	Osteoclast-stimulating factor 1 OS=Mus musculus GN=Ostf1 PE=1 SV=2		3.72093	1	1	1	23.768	5.68		1800000		1.912454
Q91V41	Ras-related protein Rab-14 OS=Mus musculus GN=Rab14 PE=1 SV=3		20.93023	4	12	3	23.882	6.21	11000000	7500000	8700000	35.08606
Q8BJF9	Charged multivesicular body protein 2b OS=Mus musculus GN=Chmp2b PE=1 SV=1		12.67606	3	4	2	23.919	8.78	3600000	3600000	970000	10.64308
Q8K3J1	NADH dehydrogenase [ubiquinone] iron-sulfur protein 8, mitochondrial OS=Mus musculus GN=Ndufs8 PE=1 SV=1		17.92453	3	7	3	24.023	6.21	3700000	1800000	3500000	19.22862
Q9CYZ2	Tumor protein D54 OS=Mus musculus GN=Tpd52l2 PE=1 SV=1		13.63636	2	5	2	24.028	6.15	1500000	1500000	1100000	15.49978
P35282	Ras-related protein Rab-21 OS=Mus musculus GN=Rab21 PE=1 SV=4		31.53153	5	12	5	24.091	7.94	4000000	3700000	3500000	37.16152
Q9CZM2	60S ribosomal protein L15 OS=Mus musculus GN=Rpl15 PE=2 SV=4		29.41176	6	14	6	24.131	11.62	39000000	44000000	40000000	35.20716
P30681	High mobility group protein B2 OS=Mus musculus GN=Hmgb2 PE=1 SV=3		38.09524	8	44	7	24.147	7.31	82000000	1E+08	93000000	147.7735
Q922J6	Tetraspanin-2 OS=Mus musculus GN=Tspan2 PE=1 SV=1		5.429864	1	5	1	24.165	7.96	7900000	2600000	2000000	16.45182
P62242	40S ribosomal protein S8 OS=Mus musculus GN=Rps8 PE=1 SV=2		51.92308	11	41	11	24.19	10.32	88000000	85000000	92000000	126.6225
Q9Z2Q5	39S ribosomal protein L40, mitochondrial OS=Mus musculus GN=Mrpl40 PE=1 SV=2		4.368932	1	1	1	24.286	9.47	960000			2.205929
P47963	60S ribosomal protein L13 OS=Mus musculus GN=Rpl13 PE=1 SV=3		24.17062	5	18	5	24.291	11.55	60000000	61000000	58000000	48.87027
Q99LP6	GrpE protein homolog 1, mitochondrial OS=Mus musculus GN=Grpel1 PE=1 SV=1		5.069124	1	3	1	24.292	8.38	1300000	980000	1000000	8.234342
Q8K386	Ras-related protein Rab-15 OS=Mus musculus GN=Rab15 PE=1 SV=1		29.24528	4	8	2	24.303	5.71	81000000	9500000	9400000	22.09103
Q9CYH2	Redox-regulatory protein FAM213A OS=Mus musculus GN=Fam213a PE=1 SV=2		13.76147	3	6	3	24.379	9.17	2100000	1800000	1300000	14.74474
P00761 SW	Trypsin - Sus scrofa (Pig).	x	25.10823	4	35	4	24.394	7.18	1E+09	2.3E+09	9.8E+08	131.5577
P62827	GTP-binding nuclear protein Ran OS=Mus musculus GN=Ran PE=1 SV=3		24.53704	5	14	5	24.408	7.49	19000000	22000000	15000000	38.72863
Q78PG9	Coiled-coil domain-containing protein 25 OS=Mus musculus GN=Ccdc25 PE=1 SV=1		6.25	1	2	1	24.464	6.95	2000000	1500000		5.937531
P46638	Ras-related protein Rab-11B OS=Mus musculus GN=Rab11b PE=1 SV=3		37.15596	8	20	8	24.474	5.94	17000000	15000000	11000000	54.09618
Q99JY3	GTPase IMAP family member 4 OS=Mus musculus GN=Gimap4 PE=1 SV=1		10.04566	1	1	1	24.539	7.02		310000		2.207124
Q99JH8	ER lumen protein-retaining receptor 1 OS=Mus musculus GN=Kdelr1 PE=1 SV=1		5.188679	1	3	1	24.544	8.62		4300000	4000000	6.975781
Q8R191	Synaptogyrin-3 OS=Mus musculus GN=Syngr3 PE=1 SV=1		6.550218	1	3	1	24.545	8.18	6500000	3300000	950000	9.794398
Q9D1X0	Nucleolar protein 3 OS=Mus musculus GN=Nol3 PE=1 SV=1		8.636364	1	3	1	24.552	4.07	290000	700000	470000	8.245847
P00493	Hypoxanthine-guanine phosphoribosyltransferase OS=Mus musculus GN=Hprt1 PE=1 SV=3		5.504587	1	3	1	24.555	6.68	1200000	620000	630000	7.220677
Q9D7S9	Charged multivesicular body protein 5 OS=Mus musculus GN=Chmp5 PE=1 SV=1		3.196347	1	1	1	24.56	4.79	1800000			1.96912
Q6ZWV3	60S ribosomal protein L10 OS=Mus musculus GN=Rpl10 PE=1 SV=3		20.09346	4	14	4	24.588	10.08	28000000	21000000	25000000	40.29755
P09671	Superoxide dismutase [Mn], mitochondrial OS=Mus musculus GN=Sod2 PE=1 SV=3		19.81982	4	9	4	24.588	8.62	11000000	8900000	10000000	27.24197
P63163	Small nuclear ribonucleoprotein-associated protein N OS=Mus musculus GN=Snrpn PE=2 SV=1		6.25	1	3	1	24.598	11.19		860000	1500000	9.351048
P23506	Protein-L-isoaspartate(D-aspartate) O-methyltransferase OS=Mus musculus GN=Pcmt1 PE=1 SV=3		9.251101	2	4	2	24.619	7.65	11000000	4300000	5700000	11.76766
Q9DC70	NADH dehydrogenase [ubiquinone] iron-sulfur protein 7, mitochondrial OS=Mus musculus GN=Ndufs7 PE=1 SV=1		14.28571	3	7	3	24.667	9.92	3400000	2600000	2100000	18.72735
O70251	Elongation factor 1-beta OS=Mus musculus GN=Eef1b PE=1 SV=5		33.33333	5	22	4	24.678	4.69	31000000	26000000	25000000	80.88232
Q8VE70	Programmed cell death protein 10 OS=Mus musculus GN=Pdcd10 PE=1 SV=1		16.98113	2	2	2	24.7	8.19		2200000		4.903047
Q8CJ26	Death domain-containing membrane protein NRADD OS=Mus musculus GN=Nradd PE=1 SV=1		7.017544	1	3	1	24.711	5.01	710000	730000	300000	9.423785
O08547	Vesicle-trafficking protein SEC22b OS=Mus musculus GN=Sec22b PE=1 SV=3		41.39535	7	23	7	24.725	8.51	7600000	7900000	7000000	72.06976
Q9R0P9	Ubiquitin carboxyl-terminal hydrolase isozyme L1 OS=Mus musculus GN=Uchl1 PE=1 SV=1		12.55605	2	6	2	24.822	5.24	5700000	5600000	4000000	22.56014
O08709	Peroxiredoxin-6 OS=Mus musculus GN=Prdx6 PE=1 SV=3		22.32143	4	11	4	24.855	6.01	3600000	4700000	3200000	29.46206
P63158	High mobility group protein B1 OS=Mus musculus GN=Hmgb1 PE=1 SV=2		46.51163	12	49	11	24.878	5.74	1.2E+08	1.3E+08	1E+08	172.1243
Q9D1D4	Transmembrane emp24 domain-containing protein 10 OS=Mus musculus GN=Tmed10 PE=1 SV=1		29.68037	7	22	7	24.895	6.7	10000000	10000000	14000000	64.38056
P53026	60S ribosomal protein L10a OS=Mus musculus GN=Rpl10a PE=1 SV=3		25.80645	6	21	6	24.901	9.98	25000000	20000000	21000000	58.86909
Q4VAE3	Transmembrane protein 65 OS=Mus musculus GN=Tmem65 PE=1 SV=1		4.273504	1	1	1	24.902	7.78		1400000		1.976064
Q9D8B3	Charged multivesicular body protein 4b OS=Mus musculus GN=Chmp4b PE=1 SV=2		4.910714	1	2	1	24.921	4.82	3000000		2600000	3.820038
P67871	Casein kinase II subunit beta OS=Mus musculus GN=Csnk2b PE=1 SV=1		17.67442	3	9	3	24.926	5.55	3400000	3000000	3900000	26.70641
P70280	Vesicle-associated membrane protein 7 OS=Mus musculus GN=Vamp7 PE=1 SV=1		8.636364	2	5	2	24.951	8.6	2000000	1600000	2100000	12.32198
P63011	Ras-related protein Rab-3A OS=Mus musculus GN=Rab3a PE=1 SV=1		29.54545	5	19	3	24.954	5.03	27000000	19000000	11000000	60.50397
Q9CYN2	Signal peptidase complex subunit 2 OS=Mus musculus GN=Spcs2 PE=1 SV=1		30.97345	4	9	4	24.962	8.57	2200000	2000000	1400000	32.1104
P63073	Eukaryotic translation initiation factor 4E OS=Mus musculus GN=Eif4e PE=1 SV=1		10.13825	2	4	2	25.037	6.15		1500000	1500000	9.09938
Q9CZE3	Ras-related protein Rab-32 OS=Mus musculus GN=Rab32 PE=1 SV=3		10.76233	2	2	2	25.053	6.68	1300000	2200000		6.02985
Q9DBZ5	Eukaryotic translation initiation factor 3 subunit K OS=Mus musculus GN=Eif3k PE=1 SV=1		5.045872	1	3	1	25.07	4.93	3400000	3300000	2700000	5.838413
Q9DB34	Charged multivesicular body protein 2a OS=Mus musculus GN=Chmp2a PE=1 SV=1		12.61261	2	4	2	25.118	5.97	2500000	1600000	1900000	16.28427
Q62446	Peptidyl-prolyl cis-trans isomerase FKBP3 OS=Mus musculus GN=Fkbp3 PE=1 SV=2		22.76786	4	9	4	25.132	9.28	5200000	6700000	4000000	26.2467
Q6IRU5	Clathrin light chain B OS=Mus musculus GN=Cltb PE=1 SV=1		16.15721	4	11	4	25.156	4.63	7700000	8600000	5500000	29.77965
P12979	Myogenin OS=Mus musculus GN=Myog PE=1 SV=2		4.017857	1	2	1	25.187	5.81	210000		30000000	4.288675

Q60631	Growth factor receptor-bound protein 2 OS=Mus musculus GN=Grb2 PE=1 SV=1		20.2765	4	7	4	25.222	6.32	6700000	8300000	6100000	20.04269
P40240	CD9 antigen OS=Mus musculus GN=Cd9 PE=1 SV=2		3.097345	1	3	1	25.241	7.23	35000000	27000000	25000000	6.828262
Q9CQI7	U2 small nuclear ribonucleoprotein B'' OS=Mus musculus GN=Snrpb2 PE=1 SV=1		4.888889	1	1	1	25.307	9.72		1900000		2.327171
Q9JKV5	Secretory carrier-associated membrane protein 4 OS=Mus musculus GN=Scamp4 PE=1 SV=1		4.782609	1	2	1	25.326	8.66		3600000	3600000	5.491157
Q9D8X2	Coiled-coil domain-containing protein 124 OS=Mus musculus GN=Ccdc124 PE=1 SV=1		8.294931	2	2	2	25.33	9.64		1800000	1600000	4.790138
Q60692	Proteasome subunit beta type-6 OS=Mus musculus GN=Psmb6 PE=1 SV=3		4.621849	1	1	1	25.362	5.11	3300000			3.000273
Q9DCG2	CD302 antigen OS=Mus musculus GN=Cd302 PE=1 SV=2		6.140351	1	1	1	25.406	4.61		510000		3.152957
P46412	Glutathione peroxidase 3 OS=Mus musculus GN=Gpx3 PE=1 SV=2		12.83186	2	2	2	25.409	8.22	2500000	1500000		4.861577
Q9D1C8	Vacuolar protein sorting-associated protein 28 homolog OS=Mus musculus GN=Vps28 PE=1 SV=1		6.78733	1	2	1	25.436	5.54		580000	250000	6.666197
Q8BIF0	CD99 antigen-like protein 2 OS=Mus musculus GN=Cd99l2 PE=1 SV=1		13.92405	2	2	2	25.447	4.86		2200000	160000	5.046483
Q78IS1	Transmembrane emp24 domain-containing protein 3 OS=Mus musculus GN=Tmed3 PE=1 SV=1		9.954751	2	4	2	25.449	5.96	4800000		4600000	11.34749
Q62093	Serine/arginine-rich splicing factor 2 OS=Mus musculus GN=Srsf2 PE=1 SV=4		14.93213	4	39	4	25.461	11.85	28000000	34000000	36000000	138.938
Q9JM93	ADP-ribosylation factor-like protein 6-interacting protein 4 OS=Mus musculus GN=Arl6ip4 PE=1 SV=1		12.22707	2	6	2	25.51	11.19	2300000	2100000	2100000	22.51936
Q8BVI4	Dihydropteridine reductase OS=Mus musculus GN=Qdpr PE=1 SV=2		7.46888	1	2	1	25.554	7.81	420000	290000		5.309165
O08585	Clathrin light chain A OS=Mus musculus GN=Clta PE=1 SV=2		18.29787	6	11	6	25.588	4.58	6200000	15000000	4200000	25.95606
Q9QYI6	DnaJ homolog subfamily B member 9 OS=Mus musculus GN=Dnajb9 PE=1 SV=2		6.756757	1	1	1	25.6	8.28			460000	2.811934
O55100	Synaptogyrin-1 OS=Mus musculus GN=Syngr1 PE=1 SV=2		5.128205	1	3	1	25.636	4.65	2600000	1000000	1200000	8.180473
Q9D0B0	Serine/arginine-rich splicing factor 9 OS=Mus musculus GN=Srsf9 PE=1 SV=1		25.67568	6	13	6	25.645	8.65	3800000	5200000	3400000	31.66737
Q9ET26	E3 ubiquitin-protein ligase RNF114 OS=Mus musculus GN=Rnf114 PE=1 SV=2		4.366812	1	1	1	25.728	7.03			910000	2.10266
P41731	CD63 antigen OS=Mus musculus GN=Cd63 PE=1 SV=2		4.621849	1	1	1	25.749	6.98		2100000		2.200985
Q9CR02	Translation machinery-associated protein 16 OS=Mus musculus GN=Tma16 PE=1 SV=1		21.26697	4	9	4	25.766	9.19	1900000	1800000	2800000	23.42917
P35762	CD81 antigen OS=Mus musculus GN=Cd81 PE=1 SV=2		21.18644	3	13	3	25.797	5.83	6900000	4700000	5200000	47.94801
Q9JIK9	28S ribosomal protein S34, mitochondrial OS=Mus musculus GN=Mrps34 PE=1 SV=1		6.880734	1	3	1	25.811	10.43	1400000	1600000	1200000	8.891376
P62823	Ras-related protein Rab-3C OS=Mus musculus GN=Rab3c PE=1 SV=1		14.97797	3	7	1	25.856	5.24	3700000	1600000		18.98239
P49722	Proteasome subunit alpha type-2 OS=Mus musculus GN=Psma2 PE=1 SV=3		4.700855	1	3	1	25.91	7.43	6400000	9300000	4800000	8.154828
P10649	Glutathione S-transferase Mu 1 OS=Mus musculus GN=Gstm1 PE=1 SV=2		19.26606	4	12	3	25.953	7.94	8600000	5700000	3900000	33.45445
P98086	Complement C1q subcomponent subunit A OS=Mus musculus GN=C1qa PE=1 SV=2		13.87755	3	10	3	25.958	9.11	17000000	22000000	20000000	30.32102
P00405	Cytochrome c oxidase subunit 2 OS=Mus musculus GN=Mtco2 PE=1 SV=1		3.0837	1	1	1	25.959	4.73			23000000	1.899357
Q02105	Complement C1q subcomponent subunit C OS=Mus musculus GN=C1qc PE=1 SV=2		20.3252	4	14	4	25.975	8.54	23000000	19000000	15000000	41.60524
Q8JZS0	Protein lin-7 homolog A OS=Mus musculus GN=Lin7a PE=1 SV=2		10.72961	3	5	3	25.977	8.72	3500000	4600000	4400000	13.03417
Q8R1V4	Transmembrane emp24 domain-containing protein 4 OS=Mus musculus GN=Tmed4 PE=1 SV=1		15.4185	3	9	2	26.005	8.18	11000000	8400000	8600000	27.04208
Q61189	Methylosome subunit pICln OS=Mus musculus GN=Clns1a PE=1 SV=1		5.508475	1	3	1	26.005	4.12	2900000	2500000	1600000	8.041607
Q6PEB6	MOB-like protein phocein OS=Mus musculus GN=Mob4 PE=1 SV=1		10.22222	2	6	2	26.016	5.78	4100000	3200000	2300000	14.62234
Q9JKD3	Secretory carrier-associated membrane protein 5 OS=Mus musculus GN=Scamp5 PE=1 SV=1		4.680851	1	3	1	26.051	8.54	9900000	5700000	4900000	9.31316
Q9D287	Pre-mRNA-splicing factor SPF27 OS=Mus musculus GN=Bcas2 PE=1 SV=1		5.333333	1	2	1	26.115	5.66		2200000	1100000	6.620747
P50518	V-type proton ATPase subunit E 1 OS=Mus musculus GN=Atp6v1e1 PE=1 SV=2		20.35398	4	11	4	26.141	8.43	8400000	7900000	4800000	34.32203
Q9CXE7	Transmembrane emp24 domain-containing protein 5 OS=Mus musculus GN=Tmed5 PE=1 SV=1		14.41048	3	5	3	26.155	4.93	2300000	1900000		12.2774
Q91YJ3	Thymocyte nuclear protein 1 OS=Mus musculus GN=Thyn1 PE=1 SV=1		9.292035	2	2	2	26.161	9.11		710000	990000	5.210595
Q62348	Translin OS=Mus musculus GN=Tsn PE=1 SV=1		6.578947	1	3	1	26.185	6.44	2800000	4500000		8.608363
Q9CQ79	Thioredoxin domain-containing protein 9 OS=Mus musculus GN=Txndc9 PE=1 SV=1		5.752212	1	2	1	26.243	5.95	2900000		3100000	7.443305
P51859	Hepatoma-derived growth factor OS=Mus musculus GN=Hdgf PE=1 SV=2		26.58228	5	11	5	26.253	4.83	4000000	4200000	2600000	25.93027
Q3UBX0	Transmembrane protein 109 OS=Mus musculus GN=Tmem109 PE=1 SV=2		4.938272	1	3	1	26.289	9.89	6100000	4300000	3400000	10.05573
Q80XU3	Nuclear ubiquitous casein and cyclin-dependent kinase substrate 1 OS=Mus musculus GN=Nucks1 PE=1 SV=1		20.08547	4	13	4	26.298	5.14	8200000	7600000	9300000	41.52556
Q9CRB9	MICOS complex subunit Mic19 OS=Mus musculus GN=Chchd3 PE=1 SV=1		27.31278	5	11	5	26.318	8.37	14000000	10000000	18000000	32.82347
O09061	Proteasome subunit beta type-1 OS=Mus musculus GN=Psmb1 PE=1 SV=1		9.583333	2	6	2	26.355	7.81	3800000	2800000	3000000	16.47435
Q9Z2U1	Proteasome subunit alpha type-5 OS=Mus musculus GN=Psma5 PE=1 SV=1		16.59751	3	4	3	26.394	4.79	2900000	2100000	2700000	13.24066
Q8BH50	Uncharacterized protein C18orf25 homolog OS=Mus musculus PE=1 SV=1		5.714286	1	1	1	26.43	5.19	1100000			2.909492
Q9D1D6	Collagen triple helix repeat-containing protein 1 OS=Mus musculus GN=Cthrc1 PE=2 SV=2		3.265306	1	3	1	26.443	7.5	2000000	1700000	1600000	7.148525
O55135	Eukaryotic translation initiation factor 6 OS=Mus musculus GN=Eif6 PE=1 SV=2		17.55102	3	6	3	26.494	4.74	2700000	2100000	490000	16.04449
Q9CXW3	Calcyclin-binding protein OS=Mus musculus GN=Cacybp PE=1 SV=1		11.79039	3	6	3	26.494	7.87	5800000	5000000	2500000	15.09918
Q9QY36	N-alpha-acetyltransferase 10 OS=Mus musculus GN=Naa10 PE=1 SV=1		14.46809	2	5	2	26.503	5.64	3400000	2800000	2200000	17.04702
Q9CY57	Chromatin target of PRMT1 protein OS=Mus musculus GN=Chtop PE=1 SV=2		10.44177	2	5	2	26.568	12.23	880000	1200000	1100000	11.95871
P50171	Estradiol 17-beta-dehydrogenase 8 OS=Mus musculus GN=Hsd17b8 PE=1 SV=2		5.019305	1	2	1	26.572	6.54	1000000		1100000	4.280788

Q9JHS9	Spliceosome-associated protein CWC15 homolog OS=Mus musculus GN=Cwc15 PE=1 SV=1		3.49345	1	3	1	26.608	5.71	1800000	1800000	1600000	6.085351
P48774	Glutathione S-transferase Mu 5 OS=Mus musculus GN=Gstm5 PE=1 SV=1		13.83929	3	4	2	26.617	7.21		950000	1500000	9.191757
P62908	40S ribosomal protein S3 OS=Mus musculus GN=Rps3 PE=1 SV=1		55.14403	12	39	12	26.657	9.66	52000000	52000000	47000000	111.8877
O88384	Vesicle transport through interaction with t-SNAREs homolog 1B OS=Mus musculus GN=Vti1b PE=1 SV=1		9.913793	2	3	2	26.697	8.79	1100000	1000000		6.327054
P14106	Complement C1q subcomponent subunit B OS=Mus musculus GN=C1qb PE=1 SV=2		16.60079	4	11	4	26.701	8.15	19000000	30000000	14000000	34.50111
Q9D8Y0	EF-hand domain-containing protein D2 OS=Mus musculus GN=Efhd2 PE=1 SV=1		20	5	10	5	26.775	5.06	5200000	4500000	3800000	31.79098
Q9DCI3	MLN64 N-terminal domain homolog OS=Mus musculus GN=Stard3nl PE=1 SV=2		4.255319	1	1	1	26.794	5.02			1100000	1.951964
Q9JIQ3	Diablo homolog, mitochondrial OS=Mus musculus GN=Diablo PE=1 SV=2		8.438819	1	1	1	26.804	6.37		940000		4.221525
Q8VI63	MOB kinase activator 2 OS=Mus musculus GN=Mob2 PE=1 SV=1		4.680851	1	1	1	26.834	6.52				2.152776
O88983	Syntaxin-8 OS=Mus musculus GN=Stx8 PE=1 SV=1		3.389831	1	1	1	26.908	5.01	900000			2.312785
O08583	THO complex subunit 4 OS=Mus musculus GN=Alyref PE=1 SV=3		10.98039	2	9	2	26.924	11.15	3100000	2600000	1600000	29.59436
Q9QY76	Vesicle-associated membrane protein-associated protein B OS=Mus musculus GN=Vapb PE=1 SV=3		15.22634	3	10	3	26.929	7.78	14000000	8000000	8800000	28.43764
O35130	Ribosomal RNA small subunit methyltransferase NEP1 OS=Mus musculus GN=Emg1 PE=1 SV=1		5.737705	1	1	1	26.957	8.91				3.44533
Q9JMI7	Testis-expressed sequence 101 protein OS=Mus musculus GN=Tex101 PE=1 SV=1		15.2	3	8	3	26.98	6.47	2000000	2200000	1800000	22.46838
Q9Z1Q5	Chloride intracellular channel protein 1 OS=Mus musculus GN=Clic1 PE=1 SV=3		33.19502	4	10	4	26.996	5.17	2700000	4300000	3400000	27.12908
P97372	Proteasome activator complex subunit 2 OS=Mus musculus GN=Psme2 PE=1 SV=4		20.08368	5	17	5	27.04	5.76	13000000	17000000	11000000	50.43765
Q99KF1	Transmembrane emp24 domain-containing protein 9 OS=Mus musculus GN=Tmed9 PE=1 SV=2		25.10638	4	7	3	27.11	8.41	17000000	7300000	13000000	22.07309
P04370	Myelin basic protein OS=Mus musculus GN=Mbp PE=1 SV=2		18.4	5	41	5	27.151	9.58	2E+08	91000000	93000000	114.3981
Q9D6J6	NADH dehydrogenase [ubiquinone] flavoprotein 2, mitochondrial OS=Mus musculus GN=Ndufv2 PE=1 SV=2		12.09677	2	6	2	27.268	7.4	1700000	1600000	1100000	16.59209
Q9ERU3	Zinc finger protein 22 OS=Mus musculus GN=Znf22 PE=1 SV=2		4.219409	1	1	1	27.277	10.29	250000			2.015611
P08882	Granzyme C OS=Mus musculus GN=Gzmc PE=1 SV=1		10.48387	2	5	2	27.293	9.22	2600000	1900000	2200000	16.90236
P35283	Ras-related protein Rab-12 OS=Mus musculus GN=Rab12 PE=1 SV=3		10.28807	2	5	1	27.311	8.41		1300000		13.62256
Q9WUK2	Eukaryotic translation initiation factor 4H OS=Mus musculus GN=Eif4h PE=1 SV=3		13.70968	3	6	3	27.324	7.23	23000000	9900000	2400000	15.72932
Q9QUM9	Proteasome subunit alpha type-6 OS=Mus musculus GN=Psma6 PE=1 SV=1		27.64228	6	10	6	27.355	6.76	3900000	4800000	2300000	26.33703
P18181	CD48 antigen OS=Mus musculus GN=Cd48 PE=1 SV=1		6.25	1	1	1	27.366	7.69				2.414094
Q9D1E6	Tubulin-folding cofactor B OS=Mus musculus GN=Tbcb PE=1 SV=2		7.377049	1	3	1	27.368	5.24	1400000	1100000		9.995395
O08756	3-hydroxyacyl-CoA dehydrogenase type-2 OS=Mus musculus GN=Hsd17b10 PE=1 SV=4		12.64368	2	5	2	27.402	8.41	700000	960000	850000	16.4257
Q9CPS7	RNA-binding protein PNO1 OS=Mus musculus GN=Pno1 PE=1 SV=1		6.451613	1	1	1	27.437	9.79		84000		2.509561
P04187	Granzyme B(G,H) OS=Mus musculus GN=Gzmb PE=1 SV=1		5.668016	1	1	1	27.452	9.76		490000		2.274461
Q9ESX4	Nucleolar protein of 40 kDa OS=Mus musculus GN=Zcchc17 PE=1 SV=1		5.809129	1	1	1	27.454	9.63	740000			2.823309
Q9D1F4	Proline-rich AKT1 substrate 1 OS=Mus musculus GN=Akt1s1 PE=1 SV=1		7.392996	1	2	1	27.466	4.72	390000	250000		6.25966
P16110	Galectin-3 OS=Mus musculus GN=Lgals3 PE=1 SV=3		9.469697	2	3	2	27.498	8.38		3500000		7.403241
Q9CXS4	Centromere protein V OS=Mus musculus GN=Cenpv PE=1 SV=2		32.53968	5	12	5	27.524	9.79	3000000	4500000	2800000	40.81536
Q62283	Tetraspanin-7 OS=Mus musculus GN=Tspan7 PE=1 SV=2		6.024096	1	1	1	27.526	7.2	3100000			2.494726
Q9D0I8	mRNA turnover protein 4 homolog OS=Mus musculus GN=Mrto4 PE=1 SV=1		12.13389	3	5	3	27.528	8.54	2300000	1200000	4300000	12.47142
Q9CRD0	OCIA domain-containing protein 1 OS=Mus musculus GN=Ociad1 PE=1 SV=1		16.59919	3	6	3	27.593	7.81	5100000	4300000	4200000	22.42357
Q9DCW4	Electron transfer flavoprotein subunit beta OS=Mus musculus GN=Etfb PE=1 SV=3		8.627451	2	4	2	27.606	8.1		2500000	1400000	9.710609
Q6PDM2	Serine/arginine-rich splicing factor 1 OS=Mus musculus GN=Srsf1 PE=1 SV=3		38.70968	10	38	10	27.728	10.36	53000000	58000000	60000000	97.42715
Q9R0H2	Endomucin OS=Mus musculus GN=Emcn PE=1 SV=1		6.51341	1	1	1	27.739	6.39				2.903577
P63101	14-3-3 protein zeta/delta OS=Mus musculus GN=Ywhaz PE=1 SV=1		64.89796	17	59	13	27.754	4.79	1.3E+08	1.2E+08	97000000	188.6143
P68254	14-3-3 protein theta OS=Mus musculus GN=Ywhaq PE=1 SV=1		51.42857	13	40	8	27.761	4.78	22000000	25000000	18000000	129.2776
Q9CQK7	RWD domain-containing protein 1 OS=Mus musculus GN=Rwdd1 PE=1 SV=1		3.703704	1	3	1	27.768	4.26	3600000	2900000	3100000	7.558844
Q9D883	Splicing factor U2AF 35 kDa subunit OS=Mus musculus GN=U2af1 PE=1 SV=4		12.5523	3	10	3	27.797	8.81	8600000	13000000	15000000	25.61624
Q9WV55	Vesicle-associated membrane protein-associated protein A OS=Mus musculus GN=Vapa PE=1 SV=2		17.67068	4	21	4	27.837	8.4	16000000	15000000	15000000	52.04171
Q9Z2U0	Proteasome subunit alpha type-7 OS=Mus musculus GN=Psma7 PE=1 SV=1		20.56452	5	11	5	27.838	8.46	9500000	7600000	7400000	31.63481
Q9EQQ2	Protein YIPF5 OS=Mus musculus GN=Yipf5 PE=1 SV=1		4.669261	1	3	1	27.855	4.36	2300000	3200000	2800000	8.470575
Q61335	B-cell receptor-associated protein 31 OS=Mus musculus GN=Bcap31 PE=1 SV=4		17.95918	4	9	4	27.939	8.7	2500000	5600000	9200000	23.80926
Q61334	B-cell receptor-associated protein 29 OS=Mus musculus GN=Bcap29 PE=1 SV=1		5.833333	1	2	1	27.946	9.72		4500000	5000000	6.390614
P04925	Major prion protein OS=Mus musculus GN=Prnp PE=1 SV=2		12.20472	3	5	3	27.96	9.33	2200000	750000		13.62611
P40630	Transcription factor A, mitochondrial OS=Mus musculus GN=Tfam PE=1 SV=2		16.04938	4	8	4	27.97	9.69	2700000	3000000	2400000	20.16838
P62918	60S ribosomal protein L8 OS=Mus musculus GN=Rpl8 PE=1 SV=2		15.1751	4	14	4	28.007	11.03	35000000	45000000	34000000	44.40296
Q8K201	Keratinocyte-associated transmembrane protein 2 OS=Mus musculus GN=Kct2 PE=1 SV=2		8.880309	2	5	2	28.033	5.07	3200000	2400000	2300000	12.83977
Q80X85	28S ribosomal protein S7, mitochondrial OS=Mus musculus GN=Mrps7 PE=1 SV=1		4.958678	1	1	1	28.045	9.94			1100000	2.628201

Q9CQV8	14-3-3 protein beta/alpha OS=Mus musculus GN=Ywhab PE=1 SV=3		60.56911	15	52	8	28.069	4.83	40000000	33000000	27000000	170.5274
Q9D172	ES1 protein homolog, mitochondrial OS=Mus musculus GN=D10Jhu81e PE=1 SV=1		3.759398	1	1	1	28.073	8.78			2400000	2.387598
P14434	H-2 class II histocompatibility antigen, A-B alpha chain OS=Mus musculus GN=H2-Aa PE=1 SV=2		20.3125	4	17	4	28.075	4.78	27000000	22000000	18000000	57.04684
Q8BQ47	Protein canopy homolog 4 OS=Mus musculus GN=Cnpy4 PE=1 SV=1		8.571429	1	1	1	28.076	4.77				3.150492
P20108	Thioredoxin-dependent peroxide reductase, mitochondrial OS=Mus musculus GN=Prdx3 PE=1 SV=1		3.11284	1	2	1	28.109	7.58	3800000	3300000		4.749416
Q9CQE8	UPF0568 protein C14orf166 homolog OS=Mus musculus PE=1 SV=1		14.34426	3	8	3	28.135	6.89	5100000	3900000	3900000	22.71078
P68510	14-3-3 protein eta OS=Mus musculus GN=Ywhah PE=1 SV=2		64.22764	14	48	9	28.194	4.89	54000000	40000000	28000000	152.1268
O35566	CD151 antigen OS=Mus musculus GN=Cd151 PE=1 SV=2		5.928854	1	3	1	28.227	7.47		4300000		9.480971
Q8BXV2	BRI3-binding protein OS=Mus musculus GN=Bri3bp PE=1 SV=1		9.090909	2	5	2	28.245	9.52	2200000	2600000	2100000	12.86826
Q61885	Myelin-oligodendrocyte glycoprotein OS=Mus musculus GN=Mog PE=1 SV=1		13.00813	3	4	3	28.253	7.96	5000000	3800000		10.15292
Q9CZQ6	Retinoic acid early-inducible protein 1-epsilon OS=Mus musculus GN=Raet1e PE=1 SV=1		5.179283	1	1	1	28.266	8.41	1000000			2.809928
Q9CQE0	E3 ubiquitin-protein ligase RNF138 OS=Mus musculus GN=Rnf138 PE=2 SV=1		3.673469	1	2	1	28.28	7.31	2100000	2200000		5.033343
P61982	14-3-3 protein gamma OS=Mus musculus GN=Ywhag PE=1 SV=2		54.65587	13	51	6	28.285	4.89	67000000	49000000	46000000	157.5699
Q9CQE1	Protein NipSnap homolog 3B OS=Mus musculus GN=Nipsnap3b PE=1 SV=1		3.643725	1	2	1	28.291	9.48	1700000	1500000		4.56099
P57784	U2 small nuclear ribonucleoprotein A' OS=Mus musculus GN=Snrpa1 PE=1 SV=2		8.235294	1	1	1	28.34	8.62	480000			3.198054
P57746	V-type proton ATPase subunit D OS=Mus musculus GN=Atp6v1d PE=1 SV=1		17.81377	3	10	3	28.351	9.45	3500000	2900000	3100000	31.70785
Q8BTW3	Exosome complex component MTR3 OS=Mus musculus GN=Exosc6 PE=1 SV=1		5.860806	1	1	1	28.353	6.11		1500000		3.019186
Q8R0A0	General transcription factor IIF subunit 2 OS=Mus musculus GN=Gtf2f2 PE=1 SV=1		7.228916	2	2	2	28.364	9.22	1100000	500000		4.859083
O70435	Proteasome subunit alpha type-3 OS=Mus musculus GN=Psma3 PE=1 SV=3		12.15686	3	9	3	28.387	5.44	11000000	9900000	9800000	21.3454
O88456	Calpain small subunit 1 OS=Mus musculus GN=Capns1 PE=1 SV=1		16.72862	4	13	4	28.445	5.63	9500000	8800000	5800000	41.58068
Q6IRU2	Tropomyosin alpha-4 chain OS=Mus musculus GN=Tpm4 PE=1 SV=3		45.56452	13	44	10	28.45	4.68	33000000	31000000	35000000	135.3251
Q8C0D7	Inhibitor of growth protein 4 OS=Mus musculus GN=Ing4 PE=1 SV=2		5.220884	1	1	1	28.51	7.62				2.567516
O35381	Acidic leucine-rich nuclear phosphoprotein 32 family member A OS=Mus musculus GN=Anp32a PE=1 SV=1		14.5749	5	15	1	28.52	4.07	30000000	26000000	28000000	37.12145
Q9D1J1	Adaptin ear-binding coat-associated protein 2 OS=Mus musculus GN=Necap2 PE=1 SV=1		5.263158	1	1	1	28.58	7.94	290000			2.854959
P11032	Granzyme A OS=Mus musculus GN=Gzma PE=1 SV=2		6.923077	1	3	1	28.581	9.36	1400000	1400000	1100000	11.4131
P97371	Proteasome activator complex subunit 1 OS=Mus musculus GN=Psme1 PE=1 SV=2		30.52209	6	18	6	28.655	5.97	14000000	13000000	9200000	64.86527
P62754	40S ribosomal protein S6 OS=Mus musculus GN=Rps6 PE=1 SV=1		18.8755	5	25	5	28.663	10.84	51000000	49000000	51000000	72.49126
Q91WE2	Protein FAM192A OS=Mus musculus GN=Fam192a PE=1 SV=1		5.905512	1	3	1	28.684	5.14	1200000	830000	1000000	8.285443
Q9D198	Pre-mRNA-splicing factor SYF2 OS=Mus musculus GN=Syf2 PE=2 SV=1		4.545455	1	1	1	28.695	8.66			820000	2.414316
Q9QYB1	Chloride intracellular channel protein 4 OS=Mus musculus GN=Clic4 PE=1 SV=3		44.26877	8	19	8	28.711	5.59	8300000	4800000	4300000	58.23299
P70122	Ribosome maturation protein SBDS OS=Mus musculus GN=Sbds PE=1 SV=4		16.8	4	7	4	28.762	8.76	1300000	3800000	890000	17.45116
P17918	Proliferating cell nuclear antigen OS=Mus musculus GN=Pcna PE=1 SV=2		19.54023	4	15	4	28.766	4.77	11000000	12000000	8600000	51.67541
P57759	Endoplasmic reticulum resident protein 29 OS=Mus musculus GN=Erp29 PE=1 SV=2		19.08397	4	8	4	28.805	6.15	3800000	4700000	2700000	21.40897
Q9DBJ1	Phosphoglycerate mutase 1 OS=Mus musculus GN=Pgam1 PE=1 SV=3		35.43307	6	17	6	28.814	7.18	9400000	7900000	8700000	53.76023
Q9CZ28	Vacuolar-sorting protein SNF8 OS=Mus musculus GN=Snf8 PE=1 SV=1		4.651163	1	2	1	28.868	6.65	2000000	1300000		6.110387
P35737	Class II histocompatibility antigen, M beta 1 chain OS=Mus musculus GN=H2-DMb1 PE=2 SV=1		2.681992	1	1	1	28.88	7.14			2900000	1.888412
O09117	Synaptophysin-like protein 1 OS=Mus musculus GN=Sypl1 PE=1 SV=2		13.02682	2	3	2	28.881	8.79	2400000	1400000	2300000	7.130985
Q8VCL2	Protein SCO2 homolog, mitochondrial OS=Mus musculus GN=Sco2 PE=1 SV=1		3.921569	1	2	1	28.926	8.29		580000		5.319819
P13717	SWISS-PROT:P13717 Nuclease - Serratia marcescens.	x	34.21053	7	24	7	28.927	7.42	7500000	24000000	6100000	76.45025
Q9CQQ7	ATP synthase F(0) complex subunit B1, mitochondrial OS=Mus musculus GN=Atp5f1 PE=1 SV=1		18.75	5	21	5	28.93	9.06	29000000	28000000	17000000	58.97079
P28078	Class II histocompatibility antigen, M alpha chain OS=Mus musculus GN=H2-DMa PE=1 SV=1		3.448276	1	3	1	28.931	4.74	5900000	6600000	5900000	7.764122
Q8BIG7	Catechol O-methyltransferase domain-containing protein 1 OS=Mus musculus GN=Comtd1 PE=1 SV=1		4.580153	1	1	1	28.943	8.32		990000		1.838744
Q61581	Insulin-like growth factor-binding protein 7 OS=Mus musculus GN=Igfbp7 PE=1 SV=3		7.829181	1	1	1	28.951	8.31				3.192776
Q8BVF7	Gamma-secretase subunit APH-1A OS=Mus musculus GN=Aph1a PE=2 SV=2		4.90566	1	2	1	28.967	7.9	1400000		840000	4.935853
Q9JKK1	Syntaxin-6 OS=Mus musculus GN=Stx6 PE=1 SV=1		7.45098	1	1	1	28.979	4.92		580000		4.364038
P00920	Carbonic anhydrase 2 OS=Mus musculus GN=Ca2 PE=1 SV=4		13.84615	2	2	2	29.015	7.01		1600000	1700000	5.937029
Q9D6S7	Ribosome-recycling factor, mitochondrial OS=Mus musculus GN=Mrrf PE=1 SV=1		6.10687	1	2	1	29.032	9.85		250000	1000000	6.17758
O35955	Proteasome subunit beta type-10 OS=Mus musculus GN=Psmb10 PE=1 SV=1		13.91941	3	8	3	29.045	6.87	3800000	4700000	6200000	22.10573
Q64008	Ras-related protein Rab-34 OS=Mus musculus GN=Rab34 PE=1 SV=2		3.088803	1	4	1	29.082	8.27	910000	680000	770000	9.64028
P99026	Proteasome subunit beta type-4 OS=Mus musculus GN=Psmb4 PE=1 SV=1		25.37879	4	11	4	29.097	5.64	4700000	5100000	3100000	32.42829
P97376	Protein FRG1 OS=Mus musculus GN=Frg1 PE=1 SV=2		4.263566	1	3	1	29.109	8.92	930000	890000	660000	7.350332
P62259	14-3-3 protein epsilon OS=Mus musculus GN=Ywhae PE=1 SV=1		56.86275	16	83	13	29.155	4.74	64000000	61000000	49000000	255.7532
O70152	Dolichol-phosphate mannosyltransferase subunit 1 OS=Mus musculus GN=Dpm1 PE=1 SV=1		5.384615	1	3	1	29.156	9.51	2600000	2500000	2500000	10.74425

Q99KK1	Receptor expression-enhancing protein 3 OS=Mus musculus GN=Reep3 PE=1 SV=1		5.11811	1	2	1	29.195	9.58	1600000	1500000		5.722991
Q91XR9	Phospholipid hydroperoxide glutathione peroxidase, nuclear OS=Mus musculus GN=Gpx4 PE=1 SV=3		17.3913	4	9	4	29.234	10.2	3100000	1300000	2900000	23.68643
Q78IK4	MICOS complex subunit Mic27 OS=Mus musculus GN=Apool PE=1 SV=1		8.301887	2	3	2	29.242	9.31	1000000	1700000	2500000	7.563535
Q9CR68	Cytochrome b-c1 complex subunit Rieske, mitochondrial OS=Mus musculus GN=Uqcrfs1 PE=1 SV=1		14.23358	5	15	5	29.349	8.7	17000000	11000000	11000000	41.73874
Q9D6K7	Tetratricopeptide repeat protein 33 OS=Mus musculus GN=Ttc33 PE=1 SV=1		5.343511	1	2	1	29.353	5.35	480000	750000		6.236035
Q9CRA9	FGFR1 oncogene partner 2 homolog OS=Mus musculus GN=Fgfr1op2 PE=1 SV=1		5.533597	1	1	1	29.356	5.83	410000			2.489137
O08579	Emerin OS=Mus musculus GN=Emd PE=1 SV=1		32.81853	7	21	7	29.417	5.01	12000000	11000000	11000000	62.47858
Q9DC71	28S ribosomal protein S15, mitochondrial OS=Mus musculus GN=Mrps15 PE=1 SV=2		5.813953	1	3	1	29.445	10.13	1600000	1800000	1700000	8.568746
Q9R1P0	Proteasome subunit alpha type-4 OS=Mus musculus GN=Psma4 PE=1 SV=1		22.60536	6	15	6	29.452	7.72	7600000	7100000	6200000	38.62415
O88587	Catechol O-methyltransferase OS=Mus musculus GN=Comt PE=1 SV=2		3.773585	1	1	1	29.467	5.83		2200000		2.39199
Q66JS6	Eukaryotic translation initiation factor 3 subunit J-B OS=Mus musculus GN=Eif3j2 PE=1 SV=1		28.13688	7	18	7	29.468	4.81	14000000	13000000	14000000	50.00505
Q7TQK4	Exosome complex component RRP40 OS=Mus musculus GN=Exosc3 PE=1 SV=3		6.20438	1	1	1	29.527	8.06		640000		3.453759
Q9R1P4	Proteasome subunit alpha type-1 OS=Mus musculus GN=Psma1 PE=1 SV=1		18.25095	4	14	4	29.528	6.46	13000000	12000000	8500000	44.01404
P47758	Signal recognition particle receptor subunit beta OS=Mus musculus GN=Srprb PE=1 SV=1		23.79182	5	11	5	29.561	9.28	4600000	5100000	2500000	34.41327
P62702	40S ribosomal protein S4, X isoform OS=Mus musculus GN=Rps4x PE=1 SV=2		51.3308	14	42	14	29.579	10.15	41000000	43000000	42000000	118.816
P97822	Acidic leucine-rich nuclear phosphoprotein 32 family member E OS=Mus musculus GN=Anp32e PE=1 SV=2		21.53846	3	8	3	29.604	3.88	7200000	5500000	4700000	27.5008
P40237	CD82 antigen OS=Mus musculus GN=Cd82 PE=1 SV=1		8.646617	2	2	2	29.609	5.02	1500000		890000	5.963275
P26645	Myristoylated alanine-rich C-kinase substrate OS=Mus musculus GN=Marcks PE=1 SV=2		67.31392	12	55	12	29.644	4.34	44000000	27000000	23000000	236.3527
Q8BHL8	Proteasome inhibitor PI31 subunit OS=Mus musculus GN=Psmf1 PE=1 SV=1		4.797048	1	1	1	29.646	5.25		1000000		2.782624
Q62193	Replication protein A 32 kDa subunit OS=Mus musculus GN=Rpa2 PE=1 SV=1		8.148148	1	1	1	29.7	6.15		200000		3.129519
Q8K2Y7	39S ribosomal protein L47, mitochondrial OS=Mus musculus GN=Mrpl47 PE=1 SV=2		4.365079	1	1	1	29.707	10.21	1300000			2.321002
Q4VAA2	Protein CDV3 OS=Mus musculus GN=Cdv3 PE=1 SV=2		6.761566	1	2	1	29.711	6.1	1400000		920000	5.923716
O88441	Metaxin-2 OS=Mus musculus GN=Mtx2 PE=1 SV=1		4.942966	1	1	1	29.739	5.63			390000	2.872656
Q9CQA5	Mediator of RNA polymerase II transcription subunit 4 OS=Mus musculus GN=Med4 PE=2 SV=1		3.333333	1	3	1	29.763	5.05	6700000	4500000	5500000	6.190449
Q6NZB0	DnaJ homolog subfamily C member 8 OS=Mus musculus GN=Dnajc8 PE=1 SV=2		16.60079	4	10	4	29.794	9.06	4300000	3400000	3400000	23.167
P67778	Prohibitin OS=Mus musculus GN=Phb PE=1 SV=1		18.75	5	11	5	29.802	5.76	12000000	13000000	11000000	29.93121
O70439	Syntaxin-7 OS=Mus musculus GN=Stx7 PE=1 SV=3		14.17625	3	6	3	29.802	5.78	5400000	3300000	3400000	18.22502
Q91VN4	MICOS complex subunit Mic25 OS=Mus musculus GN=Chchd6 PE=1 SV=2		5.128205	1	3	1	29.833	8.41	2000000	1200000		10.91117
P97351	40S ribosomal protein S3a OS=Mus musculus GN=Rps3a PE=1 SV=3		45.83333	11	39	11	29.866	9.73	44000000	47000000	39000000	110.8248
P00416	Cytochrome c oxidase subunit 3 OS=Mus musculus GN=mt-Co3 PE=1 SV=2		5.363985	1	4	1	29.903	7.3	6600000	6200000	8500000	10.8219
P12970	60S ribosomal protein L7a OS=Mus musculus GN=Rpl7a PE=1 SV=2		37.59398	10	36	10	29.958	10.56	71000000	72000000	69000000	100.7978
Q61792	LIM and SH3 domain protein 1 OS=Mus musculus GN=Lasp1 PE=1 SV=1		26.23574	6	13	6	29.975	7.05	5400000	4000000	4900000	37.28008
Q61166	Microtubule-associated protein RP/EB family member 1 OS=Mus musculus GN=Mapre1 PE=1 SV=3		20.52239	4	16	3	29.997	5.22	5300000	4900000	4000000	53.6872
Q91WN1	DnaJ homolog subfamily C member 9 OS=Mus musculus GN=Dnajc9 PE=1 SV=2		34.36293	8	21	8	30.04	5.94	9000000	7300000	4600000	61.18767
P60202	Myelin proteolipid protein OS=Mus musculus GN=Plp1 PE=1 SV=2		18.77256	5	19	5	30.057	8.35	1.5E+08	54000000	72000000	50.39801
P14483	H-2 class II histocompatibility antigen, A beta chain OS=Mus musculus GN=H2-Ab1 PE=1 SV=1		44.15094	9	27	9	30.109	7.24	41000000	35000000	25000000	96.03018
Q9DCT2	NADH dehydrogenase [ubiquinone] iron-sulfur protein 3, mitochondrial OS=Mus musculus GN=Ndufs3 PE=1 SV=2		15.20913	3	10	3	30.131	7.17	8700000	3700000	5000000	33.92153
Q9D1B9	39S ribosomal protein L28, mitochondrial OS=Mus musculus GN=Mrpl28 PE=1 SV=3		2.723735	1	1	1	30.151	9.29			500000	1.950735
Q9CZ04	COP9 signalosome complex subunit 7a OS=Mus musculus GN=Cops7a PE=1 SV=2		5.090909	1	2	1	30.206	7.87	1300000	1200000		7.034119
P18468	H-2 class II histocompatibility antigen, I-A beta chain OS=Mus musculus GN=H2-Eb1 PE=1 SV=1		15.90909	5	13	5	30.212	6.68	8900000	6900000	6600000	31.00223
O89051	Integral membrane protein 2B OS=Mus musculus GN=Itm2b PE=1 SV=1		16.16541	3	7	3	30.24	5.3	4800000	4100000	3500000	19.14364
P28063	Proteasome subunit beta type-8 OS=Mus musculus GN=Psmb8 PE=1 SV=2		17.75362	4	12	4	30.241	6.68	13000000	13000000	9700000	40.79702
Q921C1	Gap junction gamma-3 protein OS=Mus musculus GN=Gjc3 PE=1 SV=2		10.03717	2	4	2	30.273	8.37	4300000	6100000	8200000	14.6157
Q9R078	5'-AMP-activated protein kinase subunit beta-1 OS=Mus musculus GN=Prkab1 PE=1 SV=2		6.666667	1	2	1	30.289	6.23		1500000	1600000	6.90488
Q64310	Surfeit locus protein 4 OS=Mus musculus GN=Surf4 PE=1 SV=1		3.345725	1	3	1	30.361	7.78	930000	650000	710000	6.415946
Q9CX86	Heterogeneous nuclear ribonucleoprotein A0 OS=Mus musculus GN=Hnrnpa0 PE=1 SV=1		11.47541	2	9	2	30.512	9.31	11000000	5000000	3500000	31.52236
Q9DAU1	Protein canopy homolog 3 OS=Mus musculus GN=Cnpy3 PE=1 SV=1		6.884058	1	3	1	30.519	5.62		5300000	7200000	11.24729
Q91VJ5	Polyglutamine-binding protein 1 OS=Mus musculus GN=Pqbp1 PE=1 SV=1		16.73004	3	9	3	30.579	6.23	2000000	1700000	1300000	36.60788
Q9QYI5	DnaJ homolog subfamily B member 2 OS=Mus musculus GN=Dnajb2 PE=1 SV=2		7.942238	2	4	1	30.596	4.98				11.42048
Q00623	Apolipoprotein A-I OS=Mus musculus GN=Apoa1 PE=1 SV=2		20.45455	5	15	5	30.597	5.73	11000000	15000000	9700000	36.61595
Q60931	Voltage-dependent anion-selective channel protein 3 OS=Mus musculus GN=Vdac3 PE=1 SV=1		13.78092	3	11	2	30.733	8.79	5300000	2800000	2700000	33.94022
Q8BL97	Serine/arginine-rich splicing factor 7 OS=Mus musculus GN=Srsf7 PE=1 SV=1		21.34831	6	17	5	30.799	11.9	12000000	14000000	16000000	51.89882
Q99020	Heterogeneous nuclear ribonucleoprotein A/B OS=Mus musculus GN=Hnrnpab PE=1 SV=1		23.85965	5	19	4	30.812	7.91	46000000	38000000	37000000	57.70129

O35326	Serine/arginine-rich splicing factor 5 OS=Mus musculus GN=Srsf5 PE=1 SV=2		25.65056	9	25	8	30.873	11.56	14000000	11000000	15000000	82.17419
P53702	Cytochrome c-type heme lyase OS=Mus musculus GN=Hccs PE=1 SV=2		15.44118	3	8	3	30.958	7.12	4700000	4000000	3800000	25.35639
O35658	Complement component 1 Q subcomponent-binding protein, mitochondrial OS=Mus musculus GN=C1qbp PE=1 SV=1		24.82014	5	13	5	30.994	4.92	6800000	7800000	7200000	41.74383
O08807	Peroxiredoxin-4 OS=Mus musculus GN=Prdx4 PE=1 SV=1		10.58394	3	11	1	31.033	7.15	7700000	10000000	4300000	27.74471
Q9EST5	Acidic leucine-rich nuclear phosphoprotein 32 family member B OS=Mus musculus GN=Anp32b PE=1 SV=1		18.38235	6	19	2	31.06	4.01	25000000	23000000	36000000	44.41246
Q5NBU8	XIAP-associated factor 1 OS=Mus musculus GN=Xaf1 PE=1 SV=3		9.89011	2	3	2	31.096	8.54	840000	480000		7.216036
Q80UU2	Ribonuclease P protein subunit p38 OS=Mus musculus GN=Rpp38 PE=1 SV=1		5.714286	1	1	1	31.11	9.73			270000	2.323415
P35802	Neuronal membrane glycoprotein M6-a OS=Mus musculus GN=Gpm6a PE=1 SV=1		12.58993	4	13	4	31.128	5.27	36000000	12000000	14000000	33.46098
P24668	Cation-dependent mannose-6-phosphate receptor OS=Mus musculus GN=M6pr PE=1 SV=1		24.82014	6	14	6	31.152	5.39	18000000	12000000	7400000	42.00351
Q80X71	Transmembrane protein 106B OS=Mus musculus GN=Tmem106b PE=1 SV=1		5.090909	1	2	1	31.153	6.68	260000	640000		4.946715
Q9ER00	Syntaxin-12 OS=Mus musculus GN=Stx12 PE=1 SV=1		8.759124	2	5	2	31.176	5.44	3300000	2500000	2300000	14.35902
Q3KNM2	E3 ubiquitin-protein ligase MARCH5 OS=Mus musculus GN=March5 PE=1 SV=1		4.676259	1	1	1	31.211	8.7		220000		1.839313
P25444	40S ribosomal protein S2 OS=Mus musculus GN=Rps2 PE=1 SV=3		34.81229	10	37	10	31.212	10.24	30000000	33000000	36000000	100.6294
Q3U2S8	Voltage-gated hydrogen channel 1 OS=Mus musculus GN=Hvcn1 PE=1 SV=2		13.3829	1	2	1	31.223	7.06	3900000	2500000		10.4302
O54901	OX-2 membrane glycoprotein OS=Mus musculus GN=Cd200 PE=1 SV=1		4.316547	1	2	1	31.236	8.79	3800000	3000000		4.928986
Q7TQI3	Ubiquitin thioesterase OTUB1 OS=Mus musculus GN=Otub1 PE=1 SV=2		9.9631	2	3	2	31.25	4.94	3000000	770000		10.30013
P57776	Elongation factor 1-delta OS=Mus musculus GN=Eef1d PE=1 SV=3		38.07829	8	31	7	31.274	5.02	48000000	38000000	38000000	96.44639
Q9R0U0	Serine/arginine-rich splicing factor 10 OS=Mus musculus GN=Srsf10 PE=1 SV=2		8.396947	2	8	2	31.282	11.27	8000000	8600000	8300000	24.54855
P97760	DNA-directed RNA polymerase II subunit RPB3 OS=Mus musculus GN=Polr2c PE=1 SV=1		13.45455	2	4	2	31.292	4.97	2800000	1600000		10.73864
Q9R0P3	S-formylglutathione hydrolase OS=Mus musculus GN=Esd PE=1 SV=1		4.255319	1	3	1	31.299	7.12	2400000	2100000	1900000	7.494921
Q8CIL4	Uncharacterized protein C1orf131 homolog OS=Mus musculus PE=1 SV=1		9.252669	2	4	2	31.325	9.85	1000000		880000	10.59983
P47757	F-actin-capping protein subunit beta OS=Mus musculus GN=Capzb PE=1 SV=3		32.85199	8	21	8	31.326	5.74	14000000	12000000	10000000	55.18633
Q08331	Calretinin OS=Mus musculus GN=Calb2 PE=1 SV=3		9.9631	2	3	2	31.353	5.02	910000	260000	960000	8.58493
Q9CWS0	N(G),N(G)-dimethylarginine dimethylaminohydrolase 1 OS=Mus musculus GN=Ddah1 PE=1 SV=3		18.24561	4	14	4	31.361	5.97	4400000	3600000	4100000	43.90276
Q8VBT0	Thioredoxin-related transmembrane protein 1 OS=Mus musculus GN=Tmx1 PE=1 SV=1		11.15108	3	9	3	31.376	5.29	8700000	8800000	8400000	24.26266
P14148	60S ribosomal protein L7 OS=Mus musculus GN=Rpl7 PE=1 SV=2		37.03704	12	38	12	31.4	10.89	60000000	57000000	48000000	99.93075
Q8BH95	Enoyl-CoA hydratase, mitochondrial OS=Mus musculus GN=Echs1 PE=1 SV=1		15.51724	4	9	4	31.454	8.48	3000000	3100000	2600000	26.16001
P53811	Phosphatidylinositol transfer protein beta isoform OS=Mus musculus GN=Pitpnb PE=1 SV=2		4.797048	1	1	1	31.467	6.95		1400000		3.028388
Q01730	Ras suppressor protein 1 OS=Mus musculus GN=Rsu1 PE=1 SV=3		6.859206	2	4	2	31.531	8.88	890000	1300000	1100000	8.046801
P04441	H-2 class II histocompatibility antigen gamma chain OS=Mus musculus GN=Cd74 PE=1 SV=3		16.84588	5	15	5	31.537	8.34	6100000	5400000	3700000	37.55461
Q9CZT6	Protein CMSS1 OS=Mus musculus GN=Cmss1 PE=2 SV=1		4.710145	1	1	1	31.574	8.46		700000		2.446385
O35083	1-acyl-sn-glycerol-3-phosphate acyltransferase alpha OS=Mus musculus GN=Agpat1 PE=1 SV=1		4.912281	1	3	1	31.689	9.14	2700000	1800000	2200000	8.985074
Q9CWE0	Mitochondrial fission regulator 1-like OS=Mus musculus GN=Mtfr1l PE=1 SV=1		10.0346	2	2	2	31.706	6.1	970000			5.597733
Q60930	Voltage-dependent anion-selective channel protein 2 OS=Mus musculus GN=Vdac2 PE=1 SV=2		22.0339	5	13	5	31.713	7.49	46000000	30000000	31000000	43.2565
Q9CQA3	Succinate dehydrogenase [ubiquinone] iron-sulfur subunit, mitochondrial OS=Mus musculus GN=Sdhb PE=1 SV=1		17.7305	5	9	5	31.793	8.68	10000000	3300000	7200000	22.89029
Q9D0M0	Exosome complex exonuclease RRP42 OS=Mus musculus GN=Exosc7 PE=1 SV=2		9.621993	2	3	2	31.805	5.19	1700000	1400000	1200000	8.036978
Q9CXX9	CUE domain-containing protein 2 OS=Mus musculus GN=Cuedc2 PE=1 SV=1		5.633803	1	1	1	31.832	4.98	370000			2.997156
Q9D8C4	Interferon-induced 35 kDa protein homolog OS=Mus musculus GN=Ifi35 PE=1 SV=3		9.090909	2	2	2	31.856	5.87		1700000		5.764001
P97447	Four and a half LIM domains protein 1 OS=Mus musculus GN=Fhl1 PE=1 SV=3		4.285714	1	1	1	31.867	8.37		1300000		2.299057
P53810	Phosphatidylinositol transfer protein alpha isoform OS=Mus musculus GN=Pitpna PE=1 SV=2		7.01107	1	2	1	31.873	6.37	2700000		3600000	6.500436
Q6PER3	Microtubule-associated protein RP/EB family member 3 OS=Mus musculus GN=Mapre3 PE=1 SV=1		12.45552	3	9	2	31.946	5.54	2900000	1900000	1200000	22.67164
Q8BX10	Serine/threonine-protein phosphatase PGAM5, mitochondrial OS=Mus musculus GN=Pgam5 PE=1 SV=1		3.125	1	3	1	31.975	9.04	440000	320000	400000	7.179098
Q9Z1R4	Uncharacterized protein C6orf47 homolog OS=Mus musculus GN=D17h6s53e PE=1 SV=1		4.095563	1	3	1	31.995	6.89	1400000	1300000	1200000	7.372927
Q9CY50	Translocon-associated protein subunit alpha OS=Mus musculus GN=Ssr1 PE=1 SV=1		17.13287	3	9	3	32.045	4.45	10000000	16000000	15000000	31.70099
P27512	Tumor necrosis factor receptor superfamily member 5 OS=Mus musculus GN=Cd40 PE=1 SV=3		5.882353	1	2	1	32.071	6.74	1100000		780000	5.621374
Q3TDK6	Protein rogdi homolog OS=Mus musculus GN=Rogdi PE=1 SV=2		5.226481	1	1	1	32.08	8.18				3.940774
Q91XB7	Protein YIF1A OS=Mus musculus GN=Yif1a PE=1 SV=1		5.119454	1	2	1	32.114	9.09	120000			4.440864
Q5EBJ4	Ermin OS=Mus musculus GN=Ermn PE=1 SV=1		4.982206	1	3	1	32.128	4.59	2700000	1100000	1400000	10.71569
Q9Z2D8	Methyl-CpG-binding domain protein 3 OS=Mus musculus GN=Mbd3 PE=1 SV=1		7.368421	1	2	1	32.148	5.82			1200000	7.376273
P17751	Triosephosphate isomerase OS=Mus musculus GN=Tpi1 PE=1 SV=4		17.05686	4	15	4	32.171	5.74	6300000	5300000	5500000	43.60084
Q8CDN6	Thioredoxin-like protein 1 OS=Mus musculus GN=Txnl1 PE=1 SV=3		19.37716	4	8	4	32.217	4.96	1900000	2100000	1400000	25.05378
Q9QZ47	Troponin T, fast skeletal muscle OS=Mus musculus GN=Tnnt3 PE=1 SV=3		2.941176	1	1	1	32.221	5.31			3300000	2.175696
P42125	Enoyl-CoA delta isomerase 1, mitochondrial OS=Mus musculus GN=Eci1 PE=1 SV=2		4.49827	1	1	1	32.23	8.98	1200000			2.942576

Q8CCS6	Polyadenylate-binding protein 2 OS=Mus musculus GN=Pabpn1 PE=1 SV=3		18.54305	2	5	2	32.277	5.17	4500000	3900000	3200000	14.73271
Q924T2	28S ribosomal protein S2, mitochondrial OS=Mus musculus GN=Mrps2 PE=1 SV=1		7.216495	1	3	1	32.293	9.14	1100000	910000	630000	9.291337
Q6PFR5	Transformer-2 protein homolog alpha OS=Mus musculus GN=Tra2a PE=1 SV=1		13.879	4	7	3	32.297	11.28	2300000	3300000	2200000	19.06112
Q6ZQI3	Malectin OS=Mus musculus GN=Mlec PE=1 SV=2		24.74227	7	19	7	32.322	6.05	12000000	11000000	9800000	66.18576
Q60932	Voltage-dependent anion-selective channel protein 1 OS=Mus musculus GN=Vdac1 PE=1 SV=3		32.43243	8	33	7	32.331	8.43	90000000	54000000	54000000	114.4444
O88792	Junctional adhesion molecule A OS=Mus musculus GN=F11r PE=1 SV=2		17	3	5	3	32.403	6.77	1800000	1700000	880000	16.96161
Q61937	Nucleophosmin OS=Mus musculus GN=Npm1 PE=1 SV=1		38.0137	11	68	11	32.54	4.77	1.8E+08	2E+08	1.8E+08	251.0992
Q9DC16	Endoplasmic reticulum-Golgi intermediate compartment protein 1 OS=Mus musculus GN=Ergic1 PE=1 SV=1		6.896552	2	4	2	32.541	7.06	3700000	4100000	2300000	10.36805
Q9D819	Inorganic pyrophosphatase OS=Mus musculus GN=Ppa1 PE=1 SV=1		5.536332	1	1	1	32.646	5.6	200000			2.571636
Q9JLJ5	Elongation of very long chain fatty acids protein 1 OS=Mus musculus GN=Elovl1 PE=1 SV=1		5.017921	1	3	1	32.656	9.63	1800000	1100000	1200000	9.341904
P58771	Tropomyosin alpha-1 chain OS=Mus musculus GN=Tpm1 PE=1 SV=1		33.80282	10	42	4	32.661	4.74	47000000	45000000	53000000	126.5964
Q9EP73	Programmed cell death 1 ligand 1 OS=Mus musculus GN=Cd274 PE=1 SV=1		3.793103	1	2	1	32.76	6.23	5000000		3400000	4.852645
P58774	Tropomyosin beta chain OS=Mus musculus GN=Tpm2 PE=1 SV=1		32.04225	10	28	3	32.817	4.7	3300000	2800000	3400000	85.34738
P14206	40S ribosomal protein SA OS=Mus musculus GN=Rpsa PE=1 SV=4		24.40678	5	20	5	32.817	4.87	15000000	12000000	13000000	63.48742
Q9EQ06	Estradiol 17-beta-dehydrogenase 11 OS=Mus musculus GN=Hsd17b11 PE=1 SV=1		3.691275	1	3	1	32.86	8.66	4700000	4300000	3500000	7.839579
Q91VR2	ATP synthase subunit gamma, mitochondrial OS=Mus musculus GN=Atp5c1 PE=1 SV=1		18.12081	5	17	5	32.865	9.01	24000000	18000000	18000000	46.6852
Q3UL36	Arginine and glutamate-rich protein 1 OS=Mus musculus GN=Arglu1 PE=1 SV=2		5.904059	2	4	2	32.868	10.36	12000000	5700000		8.777703
P48962	ADP/ATP translocase 1 OS=Mus musculus GN=Slc25a4 PE=1 SV=4		29.86577	9	27	6	32.883	9.72	54000000	32000000	36000000	73.83211
P18828	Syndecan-1 OS=Mus musculus GN=Sdc1 PE=1 SV=1		9.646302	2	4	2	32.884	4.79	550000	650000	240000	10.05916
Q8R088	Golgi phosphoprotein 3-like OS=Mus musculus GN=Golph3l PE=1 SV=1		9.824561	2	5	2	32.885	5.99	1500000	1400000	1100000	15.92731
O88271	Craniofacial development protein 1 OS=Mus musculus GN=Cfdp1 PE=1 SV=1		16.94915	4	6	4	32.901	4.86	1600000	2100000	3500000	16.8713
Q9QZE7	Translin-associated protein X OS=Mus musculus GN=Tsnax PE=1 SV=1		9.655172	2	4	2	32.906	6.55	1600000	760000	710000	12.09258
P51881	ADP/ATP translocase 2 OS=Mus musculus GN=Slc25a5 PE=1 SV=3		26.84564	8	22	5	32.91	9.73	30000000	22000000	33000000	57.26764
O55126	Protein NipSnap homolog 2 OS=Mus musculus GN=Gbas PE=1 SV=1		4.982206	1	3	1	32.912	9.26	240000	450000	320000	7.015285
P20352	Tissue factor OS=Mus musculus GN=F3 PE=1 SV=2		11.56463	2	4	2	32.914	9.32	1300000	640000	860000	11.14378
P47753	F-actin-capping protein subunit alpha-1 OS=Mus musculus GN=Capza1 PE=1 SV=4		17.83217	3	6	2	32.919	5.55	11000000	6500000	8100000	19.48426
P47754	F-actin-capping protein subunit alpha-2 OS=Mus musculus GN=Capza2 PE=1 SV=3		36.01399	6	15	5	32.947	5.85	6700000	6600000	6900000	54.22261
Q69ZQ2	Pre-mRNA-splicing factor ISY1 homolog OS=Mus musculus GN=Isy1 PE=1 SV=2		4.912281	1	3	1	32.969	5.17	2000000	2100000	1800000	10.4212
P21107	Tropomyosin alpha-3 chain OS=Mus musculus GN=Tpm3 PE=1 SV=3		31.22807	10	45	4	32.974	4.72	71000000	61000000	70000000	131.282
Q8CAK3	Repressor of yield of DENV protein homolog OS=Mus musculus GN=Ryden PE=2 SV=1		6.551724	1	2	1	32.994	7.28	980000	510000		4.469101
O35526	Syntaxin-1A OS=Mus musculus GN=Stx1a PE=1 SV=3		31.25	8	24	7	33.034	5.24	12000000	10000000	8100000	71.15268
Q9DCU6	39S ribosomal protein L4, mitochondrial OS=Mus musculus GN=Mrpl4 PE=1 SV=1		3.741497	1	1	1	33.052	9.82	840000			2.431231
Q61735	Leukocyte surface antigen CD47 OS=Mus musculus GN=Cd47 PE=1 SV=2		10.89109	3	8	3	33.076	8.63	9200000	2900000	3600000	21.64149
Q08093	Calponin-2 OS=Mus musculus GN=Cnn2 PE=1 SV=1		12.13115	3	5	2	33.134	7.62		1100000	940000	15.38292
Q64012	RNA-binding protein Raly OS=Mus musculus GN=Raly PE=1 SV=3		33.01282	8	25	8	33.168	8.84	10000000	10000000	10000000	76.90936
Q9DB05	Alpha-soluble NSF attachment protein OS=Mus musculus GN=Napa PE=1 SV=1		35.59322	9	22	8	33.168	5.45	8000000	7200000	5300000	66.5629
Q64704	Syntaxin-3 OS=Mus musculus GN=Stx3 PE=1 SV=1		3.806228	1	1	1	33.222	5.45	730000			2.501001
P61264	Syntaxin-1B OS=Mus musculus GN=Stx1b PE=1 SV=1		37.15278	9	33	8	33.224	5.38	35000000	17000000	15000000	106.3701
Q91Z38	Tetratricopeptide repeat protein 1 OS=Mus musculus GN=Ttc1 PE=1 SV=1		15.75342	3	5	3	33.242	5.01	1100000	1500000	1100000	16.16511
P49615	Cyclin-dependent-like kinase 5 OS=Mus musculus GN=Cdk5 PE=1 SV=1		7.876712	2	7	1	33.267	7.66	1200000	890000	1000000	17.01234
O35129	Prohibitin-2 OS=Mus musculus GN=Phb2 PE=1 SV=1		23.74582	6	13	6	33.276	9.83	14000000	16000000	9600000	39.19361
Q9D0L7	Armadillo repeat-containing protein 10 OS=Mus musculus GN=Armc10 PE=1 SV=1		11.76471	3	9	3	33.29	7.99	1700000	1900000	1800000	24.02273
Q9CPV4	Glyoxalase domain-containing protein 4 OS=Mus musculus GN=Glod4 PE=1 SV=1		5.033557	1	2	1	33.296	5.47	2400000	2400000		5.236706
Q99PQ1	Tripartite motif-containing protein 12A OS=Mus musculus GN=Trim12a PE=2 SV=1		7.394366	2	4	2	33.299	7.27	1900000	1900000	1700000	10.5936
P14231	Sodium/potassium-transporting ATPase subunit beta-2 OS=Mus musculus GN=Atp1b2 PE=1 SV=2		15.51724	5	10	5	33.322	8.31	8500000	4600000	3500000	30.3451
O55125	Protein NipSnap homolog 1 OS=Mus musculus GN=Nipsnap1 PE=1 SV=1		3.169014	1	3	1	33.342	9.44	6500000	4700000	4900000	7.757509
Q9EQU5	Protein SET OS=Mus musculus GN=Set PE=1 SV=1		25.95156	6	21	6	33.358	4.32	36000000	34000000	32000000	78.15812
O35678	Monoglyceride lipase OS=Mus musculus GN=Mgll PE=1 SV=1		4.620462	1	1	1	33.366	7.15				2.757062
Q9JLZ3	Methylglutaconyl-CoA hydratase, mitochondrial OS=Mus musculus GN=Auh PE=1 SV=1		4.458599	1	2	1	33.374	9.51	1200000	900000		4.906563
Q8WTY4	Anamorsin OS=Mus musculus GN=Ciapin1 PE=1 SV=1		3.883495	1	1	1	33.408	5.2		840000		2.419666
Q9QZH3	Peptidyl-prolyl cis-trans isomerase E OS=Mus musculus GN=Ppie PE=1 SV=2		2.990033	1	2	1	33.428	5.6		760000	640000	5.11058
Q791V5	Mitochondrial carrier homolog 2 OS=Mus musculus GN=Mtch2 PE=1 SV=1		15.18152	4	8	4	33.477	8.25	6500000	6000000	4300000	22.00448
P47911	60S ribosomal protein L6 OS=Mus musculus GN=Rpl6 PE=1 SV=3		29.05405	12	39	12	33.489	10.7	41000000	57000000	38000000	94.23089

Q9CY64	Biliverdin reductase A OS=Mus musculus GN=Blvra PE=1 SV=1		4.745763	1	2	1	33.504	7.02	800000	980000		5.077569
P28663	Beta-soluble NSF attachment protein OS=Mus musculus GN=Napb PE=1 SV=2		35.57047	8	20	7	33.535	5.47	9200000	4400000	4000000	62.7898
Q9Z130	Heterogeneous nuclear ribonucleoprotein D-like OS=Mus musculus GN=Hnrnpdl PE=1 SV=1		15.94684	4	17	3	33.538	7.31	15000000	11000000	12000000	53.11015
Q61069	Upstream stimulatory factor 1 OS=Mus musculus GN=Usf1 PE=1 SV=1		2.903226	1	1	1	33.55	5.54		310000		2.385608
Q922Q4	Pyrroline-5-carboxylate reductase 2 OS=Mus musculus GN=Pycr2 PE=1 SV=1		9.0625	2	5	2	33.638	7.77	2000000	2100000	1700000	14.88286
P62996	Transformer-2 protein homolog beta OS=Mus musculus GN=Tra2b PE=1 SV=1		22.91667	6	15	5	33.646	11.25	3600000	9600000	5700000	44.5576
Q80UW2	F-box only protein 2 OS=Mus musculus GN=Fbxo2 PE=1 SV=1		17.84512	3	6	3	33.655	4.28	990000	1200000	900000	16.76926
Q9CRA5	Golgi phosphoprotein 3 OS=Mus musculus GN=Golph3 PE=1 SV=1		5.033557	1	2	1	33.731	6.44	6900000	6000000		6.227347
Q9ERF3	WD repeat-containing protein 61 OS=Mus musculus GN=Wdr61 PE=1 SV=1		2.622951	1	1	1	33.752	5.36		1400000		2.013358
P10711	Transcription elongation factor A protein 1 OS=Mus musculus GN=Tcea1 PE=1 SV=2		25.5814	6	17	6	33.859	8.38	8200000	9000000	8200000	53.33721
O35295	Transcriptional activator protein Pur-beta OS=Mus musculus GN=Purb PE=1 SV=3		12.03704	2	8	2	33.881	5.43	11000000	11000000	8200000	35.98172
Q8JZU2	Tricarboxylate transport protein, mitochondrial OS=Mus musculus GN=Slc25a1 PE=1 SV=1		3.536977	1	1	1	33.91	9.89			320000	3.037882
Q99K95	Protein RTF2 homolog OS=Mus musculus GN=Rtfdc1 PE=1 SV=1		3.257329	1	2	1	33.913	8.81				4.726472
Q9D710	Thioredoxin-related transmembrane protein 2 OS=Mus musculus GN=Tmx2 PE=1 SV=1		7.118644	2	4	2	33.921	8.75	3600000	2300000	3000000	10.7288
Q9WUU7	Cathepsin Z OS=Mus musculus GN=Ctsz PE=1 SV=1		8.169935	2	5	2	33.974	6.6	2200000	3900000	2100000	14.41081
P31230	Aminoacyl tRNA synthase complex-interacting multifunctional protein 1 OS=Mus musculus GN=Aimp1 PE=1 SV=2		26.77419	7	12	7	33.976	8.35	7700000	5200000	5500000	35.98336
Q9QXV9	Protein sprouty homolog 1 OS=Mus musculus GN=Spry1 PE=2 SV=1		6.709265	2	2	2	33.982	8.15	540000		860000	4.443645
Q99L04	Dehydrogenase/reductase SDR family member 1 OS=Mus musculus GN=Dhrs1 PE=1 SV=1		29.07348	8	20	8	33.983	8.35	20000000	19000000	16000000	55.64626
Q62000	Mimecan OS=Mus musculus GN=Ogn PE=1 SV=1		2.684564	1	1	1	33.991	5.74			380000	2.532214
Q62277	Synaptophysin OS=Mus musculus GN=Syp PE=1 SV=2		9.55414	3	5	3	34.002	4.94	7800000	19000000	15000000	12.08403
Q91YL2	E3 ubiquitin-protein ligase RNF126 OS=Mus musculus GN=Rnf126 PE=1 SV=1		5.43131	1	1	1	34.059	5.17		770000		2.843304
Q99KB8	Hydroxyacylglutathione hydrolase, mitochondrial OS=Mus musculus GN=Hagh PE=1 SV=2		12.62136	3	7	3	34.062	7.75	1500000	1600000	1200000	20.08941
P11440	Cyclin-dependent kinase 1 OS=Mus musculus GN=Cdk1 PE=1 SV=3		14.14141	4	11	3	34.085	8.43	2500000	4000000	2200000	29.80283
Q9DCN2	NADH-cytochrome b5 reductase 3 OS=Mus musculus GN=Cyb5r3 PE=1 SV=3		34.88372	8	24	8	34.106	8.38	19000000	17000000	14000000	71.29315
Q9DB73	NADH-cytochrome b5 reductase 1 OS=Mus musculus GN=Cyb5r1 PE=1 SV=1		3.934426	1	2	1	34.113	8.87	1400000	1500000		5.885711
Q9CR62	Mitochondrial 2-oxoglutarate/malate carrier protein OS=Mus musculus GN=Slc25a11 PE=1 SV=3		13.05732	4	7	4	34.133	9.94	1300000	2700000	7900000	17.51794
P70452	Syntaxin-4 OS=Mus musculus GN=Stx4 PE=1 SV=1		13.42282	3	9	3	34.144	6.14	1900000	1800000	1500000	29.23721
Q9DB41	Mitochondrial glutamate carrier 2 OS=Mus musculus GN=Slc25a18 PE=1 SV=4		7.1875	2	5	1	34.144	9.19		1800000	1300000	13.30324
P49312	Heterogeneous nuclear ribonucleoprotein A1 OS=Mus musculus GN=Hnrnpa1 PE=1 SV=2		37.8125	11	45	11	34.175	9.23	91000000	67000000	69000000	154.1413
Q99M28	RNA-binding protein with serine-rich domain 1 OS=Mus musculus GN=Rnps1 PE=1 SV=1		8.852459	2	10	2	34.188	11.84	17000000	17000000	17000000	31.25995
P14869	60S acidic ribosomal protein P0 OS=Mus musculus GN=Rplp0 PE=1 SV=3		38.17035	10	34	10	34.195	6.25	36000000	34000000	29000000	113.5468
Q9CYG7	Mitochondrial import receptor subunit TOM34 OS=Mus musculus GN=Tomm34 PE=1 SV=1		7.76699	2	2	2	34.257	9.14	39000000	610000		5.120617
P35550	rRNA 2'-O-methyltransferase fibrillarin OS=Mus musculus GN=Fbl PE=1 SV=2		14.37309	4	7	4	34.286	10.24	12000000	2000000	12000000	17.07733
Q8BJU0	Small glutamine-rich tetratricopeptide repeat-containing protein alpha OS=Mus musculus GN=Sgta PE=1 SV=2		7.936508	2	3	2	34.301	5.06	4100000	2200000		6.314539
Q64444	Carbonic anhydrase 4 OS=Mus musculus GN=Ca4 PE=1 SV=1		4.262295	1	1	1	34.33	8.21	1400000			2.295415
Q9CVB6	Actin-related protein 2/3 complex subunit 2 OS=Mus musculus GN=Arpc2 PE=1 SV=3		36.33333	11	26	11	34.336	7.36	28000000	25000000	19000000	71.01717
Q9Z204	Heterogeneous nuclear ribonucleoproteins C1/C2 OS=Mus musculus GN=Hnrnpc PE=1 SV=1		29.39297	9	28	9	34.364	5.05	47000000	48000000	46000000	79.66092
P47962	60S ribosomal protein L5 OS=Mus musculus GN=Rpl5 PE=1 SV=3		35.69024	11	59	11	34.379	9.77	55000000	53000000	40000000	139.3919
Q9DBX2	Phosducin-like protein OS=Mus musculus GN=Pdcl PE=1 SV=1		3.986711	1	2	1	34.385	4.87			610000	5.168906
P55088	Aquaporin-4 OS=Mus musculus GN=Aqp4 PE=1 SV=2		3.095975	1	1	1	34.414	7.42	2800000			2.157353
P07214	SPARC OS=Mus musculus GN=Sparc PE=1 SV=1		17.21854	3	4	3	34.428	4.86	1400000	1200000	1500000	11.15807
Q8K274	Ketosamine-3-kinase OS=Mus musculus GN=Fn3krp PE=1 SV=2		6.472492	1	2	1	34.446	7.87	1400000	1200000		8.645935
O88531	Palmitoyl-protein thioesterase 1 OS=Mus musculus GN=Ppt1 PE=1 SV=2		4.901961	1	3	1	34.467	8	5300000	4500000	3000000	10.80781
Q9ES74	Serine/threonine-protein kinase Nek7 OS=Mus musculus GN=Nek7 PE=1 SV=1		8.609272	3	4	3	34.514	8.25	1300000		860000	10.01043
O89079	Coatomer subunit epsilon OS=Mus musculus GN=Cope PE=1 SV=3		21.42857	5	11	5	34.545	5.06	1700000	2600000	3500000	33.60771
O35593	26S proteasome non-ATPase regulatory subunit 14 OS=Mus musculus GN=Psmd14 PE=1 SV=2		2.903226	1	1	1	34.555	6.52				2.060246
Q9D6M3	Mitochondrial glutamate carrier 1 OS=Mus musculus GN=Slc25a22 PE=1 SV=1		9.287926	3	7	2	34.648	9.09	2500000	2500000	2400000	19.99547
Q9D903	Probable rRNA-processing protein EBP2 OS=Mus musculus GN=Ebna1bp2 PE=2 SV=1		19.28105	5	12	5	34.681	10.08	5500000	6500000	7400000	30.54324
Q9CWZ7	Gamma-soluble NSF attachment protein OS=Mus musculus GN=Napg PE=1 SV=1		11.53846	3	7	3	34.71	5.41	6600000	6000000	5100000	19.30227
O70503	Very-long-chain 3-oxoacyl-CoA reductase OS=Mus musculus GN=Hsd17b12 PE=1 SV=1		13.46154	4	10	4	34.719	9.52	12000000	7700000	9200000	29.79081
Q9CS42	Ribose-phosphate pyrophosphokinase 2 OS=Mus musculus GN=Prps2 PE=1 SV=4		4.716981	1	2	1	34.764	6.61	1000000	1400000		6.916173
P52875	Transmembrane protein 165 OS=Mus musculus GN=Tmem165 PE=1 SV=2		18.88545	3	7	3	34.768	7.46	2300000	2200000	1500000	26.95092
Q61176	Arginase-1 OS=Mus musculus GN=Arg1 PE=1 SV=1		2.47678	1	1	1	34.786	7.01			680000	1.928875

P31996	Macrosialin OS=Mus musculus GN=Cd68 PE=1 SV=1		2.760736	1	1	1	34.796	8.85	2800000			1.904014
Q9D7G0	Ribose-phosphate pyrophosphokinase 1 OS=Mus musculus GN=Prps1 PE=1 SV=4		8.805031	2	6	2	34.812	6.98	1500000	1400000	1100000	14.87035
Q9D8B7	Junctional adhesion molecule C OS=Mus musculus GN=Jam3 PE=1 SV=2		10.32258	2	5	2	34.816	7.03	4100000	3400000	3000000	18.01337
Q922Q8	Leucine-rich repeat-containing protein 59 OS=Mus musculus GN=Lrrc59 PE=1 SV=1		18.89251	5	15	5	34.856	9.52	17000000	20000000	16000000	46.37577
P42669	Transcriptional activator protein Pur-alpha OS=Mus musculus GN=Pura PE=1 SV=1		21.49533	4	8	4	34.862	6.44	4200000	3000000	4000000	26.05562
Q8C4Q6	Axin interactor, dorsalization-associated protein OS=Mus musculus GN=Aida PE=1 SV=1		5.901639	1	3	1	34.866	6.74	830000	780000	600000	11.349
Q9WUD1	STIP1 homology and U box-containing protein 1 OS=Mus musculus GN=Stub1 PE=1 SV=1		4.934211	1	1	1	34.887	6.01		520000		2.67325
Q9CRD2	ER membrane protein complex subunit 2 OS=Mus musculus GN=Emc2 PE=1 SV=1		3.367003	1	3	1	34.912	6.81	4400000	4200000	3600000	8.949343
Q8C9E8	Protein FAM26F OS=Mus musculus GN=Fam26f PE=2 SV=1		2.236422	1	3	1	34.933	8.28	1500000	1400000	1500000	6.712953
Q99J47	Dehydrogenase/reductase SDR family member 7B OS=Mus musculus GN=Dhrs7b PE=1 SV=1		4.643963	1	2	1	34.964	9.63			1700000	6.786398
Q9JJZ4	Ubiquitin-conjugating enzyme E2 J1 OS=Mus musculus GN=Ube2j1 PE=1 SV=2		9.119497	2	6	2	34.968	6.99	2500000	2700000	2500000	20.13021
Q99LC5	Electron transfer flavoprotein subunit alpha, mitochondrial OS=Mus musculus GN=Etfa PE=1 SV=2		9.309309	2	3	2	34.988	8.38	1600000	2700000		10.03134
P68040	Receptor of activated protein C kinase 1 OS=Mus musculus GN=Rack1 PE=1 SV=3		41.95584	11	41	11	35.055	7.69	53000000	52000000	53000000	123.539
P97470	Serine/threonine-protein phosphatase 4 catalytic subunit OS=Mus musculus GN=Ppp4c PE=1 SV=2		4.234528	1	2	1	35.057	5.06		1300000	950000	5.42897
Q8K1Z0	Ubiquinone biosynthesis protein COQ9, mitochondrial OS=Mus musculus GN=Coq9 PE=1 SV=1		6.709265	1	1	1	35.061	5.92	2100000			5.411795
Q9QYF1	Retinol dehydrogenase 11 OS=Mus musculus GN=Rdh11 PE=1 SV=2		7.594937	2	4	2	35.125	8.91	1400000	890000	1100000	11.56128
Q9CQR6	Serine/threonine-protein phosphatase 6 catalytic subunit OS=Mus musculus GN=Ppp6c PE=1 SV=1		8.196721	2	4	2	35.136	5.69	1200000	810000	670000	9.619712
P14094	Sodium/potassium-transporting ATPase subunit beta-1 OS=Mus musculus GN=Atp1b1 PE=1 SV=1		26.64474	7	26	7	35.172	8.65	63000000	25000000	24000000	77.08927
Q99JY8	Phospholipid phosphatase 3 OS=Mus musculus GN=Plpp3 PE=1 SV=1		6.730769	2	4	2	35.193	9.07	2900000	2000000	2300000	9.426414
O35309	N-myc-interactor OS=Mus musculus GN=Nmi PE=1 SV=1		14.64968	4	10	4	35.213	5.05	2800000	3100000	2400000	30.13491
Q8K097	Protein lifeguard 2 OS=Mus musculus GN=Faim2 PE=2 SV=1		6.940063	1	1	1	35.235	6.92	1300000			2.844512
P47199	Quinone oxidoreductase OS=Mus musculus GN=Cryz PE=1 SV=1		4.229607	1	1	1	35.246	8.07		520000		2.855765
O35682	Myeloid-associated differentiation marker OS=Mus musculus GN=Myadm PE=1 SV=2		5.3125	1	1	1	35.261	8.31	2300000			4.695815
Q922B1	O-acetyl-ADP-ribose deacetylase MACROD1 OS=Mus musculus GN=Macrod1 PE=1 SV=2		3.405573	1	1	1	35.272	8.85			670000	2.475086
Q810U5	Coiled-coil domain-containing protein 50 OS=Mus musculus GN=Ccdc50 PE=1 SV=1		7.540984	2	3	2	35.3	6.89	1400000	750000	920000	8.037574
Q9D0M3	Cytochrome c1, heme protein, mitochondrial OS=Mus musculus GN=Cyc1 PE=1 SV=1		23.69231	6	23	6	35.305	9.16	5800000	5200000	4900000	54.59193
Q9CZR8	Elongation factor Ts, mitochondrial OS=Mus musculus GN=Tsfm PE=1 SV=1		6.790123	2	3	2	35.312	7.06		670000	1000000	6.098416
Q9JJ80	Ribosome production factor 2 homolog OS=Mus musculus GN=Rpf2 PE=2 SV=2		11.76471	3	8	3	35.341	10.04	2700000	3200000	2800000	21.42598
Q8R010	Aminoacyl tRNA synthase complex-interacting multifunctional protein 2 OS=Mus musculus GN=Aimp2 PE=1 SV=2		5.3125	1	3	1	35.355	7.83	1200000	1000000	970000	10.17274
Q91V61	Sideroflexin-3 OS=Mus musculus GN=Sfxn3 PE=1 SV=1		25.8567	6	14	5	35.384	9.51	6800000	5500000	4600000	44.0347
Q9D0Q7	39S ribosomal protein L45, mitochondrial OS=Mus musculus GN=Mrpl45 PE=1 SV=1		3.267974	1	2	1	35.388	9.23	1200000	880000		5.095431
P35456	Urokinase plasminogen activator surface receptor OS=Mus musculus GN=Plaur PE=1 SV=1		4.892966	1	1	1	35.405	6.83		370000		1.897521
P28352	DNA-(apurinic or apyrimidinic site) lyase OS=Mus musculus GN=Apex1 PE=1 SV=2		14.19558	3	8	3	35.468	7.91	8900000	8200000	11000000	28.8497
Q9D1M0	Protein SEC13 homolog OS=Mus musculus GN=Sec13 PE=1 SV=3		8.074534	2	6	2	35.543	5.38	4700000	4100000	4100000	19.34387
Q922H9	Zinc finger protein 330 OS=Mus musculus GN=Znf330 PE=1 SV=1		5.379747	1	1	1	35.584	6.16		2100000		3.777232
P63330	Serine/threonine-protein phosphatase 2A catalytic subunit alpha isoform OS=Mus musculus GN=Ppp2ca PE=1 SV=1		26.86084	6	21	6	35.585	5.54	8800000	7000000	6000000	79.60307
P08249	Malate dehydrogenase, mitochondrial OS=Mus musculus GN=Mdh2 PE=1 SV=3		16.56805	5	12	5	35.589	8.68	10000000	8300000	7000000	32.7856
P47802	Metaxin-1 OS=Mus musculus GN=Mtx1 PE=1 SV=1		9.148265	2	7	2	35.601	6.18	1400000	1800000	1400000	22.15766
Q9Z1D1	Eukaryotic translation initiation factor 3 subunit G OS=Mus musculus GN=Eif3g PE=1 SV=2		15.3125	4	17	4	35.616	5.9	16000000	14000000	13000000	53.35665
Q99JR1	Sideroflexin-1 OS=Mus musculus GN=Sfxn1 PE=1 SV=3		10.55901	3	6	2	35.626	9.23	4300000	4300000	3600000	16.67379
Q9DB96	Neuroguidin OS=Mus musculus GN=Ngdn PE=1 SV=1		4.444444	1	2	1	35.637	9.58		1700000	1800000	5.866894
P62960	Nuclease-sensitive element-binding protein 1 OS=Mus musculus GN=Ybx1 PE=1 SV=3		47.82609	11	61	8	35.709	9.88	27000000	24000000	25000000	196.0217
O70252	Heme oxygenase 2 OS=Mus musculus GN=Hmox2 PE=1 SV=1		10.15873	3	10	3	35.716	5.87	3800000	3400000	3000000	24.43631
P48036	Annexin A5 OS=Mus musculus GN=Anxa5 PE=1 SV=1		39.81191	12	32	12	35.73	4.96	23000000	17000000	17000000	96.00951
P09450	Transcription factor jun-B OS=Mus musculus GN=Junb PE=1 SV=1		7.267442	1	1	1	35.743	9.22		290000		2.860577
O70400	PDZ and LIM domain protein 1 OS=Mus musculus GN=Pdlim1 PE=1 SV=4		11.92661	2	2	2	35.752	6.84		2900000		5.313521
P16858	Glyceraldehyde-3-phosphate dehydrogenase OS=Mus musculus GN=Gapdh PE=1 SV=2		44.74474	11	56	11	35.787	8.25	1.8E+08	1.5E+08	1.4E+08	198.6539
P51949	CDK-activating kinase assembly factor MAT1 OS=Mus musculus GN=Mnat1 PE=1 SV=2		4.20712	1	1	1	35.825	5.82	130000			2.088196
Q9DCJ1	Target of rapamycin complex subunit LST8 OS=Mus musculus GN=Mlst8 PE=1 SV=1		5.521472	1	1	1	35.828	5.86			290000	2.628762
P08226	Apolipoprotein E OS=Mus musculus GN=Apoe PE=1 SV=2		28.29582	7	20	7	35.844	5.68	11000000	12000000	6000000	57.42241
O88545	COP9 signalosome complex subunit 6 OS=Mus musculus GN=Cops6 PE=1 SV=1		4.62963	1	2	1	35.857	5.73	1700000		790000	4.140566
Q6WVG3	BTB/POZ domain-containing protein KCTD12 OS=Mus musculus GN=Kctd12 PE=1 SV=1		16.81957	4	8	4	35.87	5.81	3700000	2100000	2300000	26.45987
Q9JJ00	Phospholipid scramblase 1 OS=Mus musculus GN=Plscr1 PE=1 SV=1		7.012195	2	4	2	35.89	5.06	1200000	3700000	1900000	9.990509

P97429	Annexin A4 OS=Mus musculus GN=Anxa4 PE=1 SV=4		37.93103	11	30	10	35.893	5.57	17000000	14000000	11000000	98.00572
E9PUL5	Proline-rich transmembrane protein 2 OS=Mus musculus GN=Prrt2 PE=1 SV=1		32.36994	4	11	4	35.902	4.63	4300000	3000000	2300000	41.14694
Q80WW9	DDRGK domain-containing protein 1 OS=Mus musculus GN=Ddrgk1 PE=1 SV=2		3.174603	1	1	1	35.956	5.35		2500000		2.019623
P10518	Delta-aminolevulinic acid dehydratase OS=Mus musculus GN=Alad PE=1 SV=1		5.757576	1	1	1	36	6.79	400000			3.042962
Q9CY27	Very-long-chain enoyl-CoA reductase OS=Mus musculus GN=Tecr PE=1 SV=1		5.844156	2	6	2	36.067	9.55	10000000	9100000	7400000	13.05374
Q6ZWX6	Eukaryotic translation initiation factor 2 subunit 1 OS=Mus musculus GN=Eif2s1 PE=1 SV=3		33.33333	9	30	9	36.085	5.08	15000000	14000000	13000000	87.6959
O35459	Delta(3,5)-Delta(2,4)-dienoyl-CoA isomerase, mitochondrial OS=Mus musculus GN=Ech1 PE=1 SV=1		7.95107	2	3	2	36.095	7.71	880000	460000	370000	6.809618
Q9Z0G0	PDZ domain-containing protein GIPC1 OS=Mus musculus GN=Gipc1 PE=1 SV=1		4.204204	1	3	1	36.107	5.91	820000	1000000	500000	8.367893
Q925N2	Sideroflexin-2 OS=Mus musculus GN=Sfxn2 PE=1 SV=1		7.763975	1	1	1	36.117	8.82		2300000		2.409861
Q9WUM5	Succinate--CoA ligase [ADP/GDP-forming] subunit alpha, mitochondrial OS=Mus musculus GN=Suclg1 PE=1 SV=4		4.33526	1	2	1	36.132	9.39	4500000	4300000		5.804232
P70372	ELAV-like protein 1 OS=Mus musculus GN=Elavl1 PE=1 SV=2		21.16564	5	11	5	36.146	9.04	12000000	8500000	14000000	31.03774
P35803	Neuronal membrane glycoprotein M6-b OS=Mus musculus GN=Gpm6b PE=1 SV=2		3.353659	1	3	1	36.186	6.14	9900000	5400000	4500000	9.768085
Q91VE6	MKI67 FHA domain-interacting nucleolar phosphoprotein OS=Mus musculus GN=Nifk PE=1 SV=1		14.8265	3	8	3	36.242	10.24	1900000	1500000	1400000	25.2349
O55091	Protein IMPACT OS=Mus musculus GN=Impact PE=1 SV=2		3.459119	1	3	1	36.253	5.05	1900000	2400000	690000	6.149927
Q9ERI6	Retinol dehydrogenase 14 OS=Mus musculus GN=Rdh14 PE=1 SV=1		7.48503	2	3	2	36.343	8.18	560000		410000	6.176892
O35639	Annexin A3 OS=Mus musculus GN=Anxa3 PE=1 SV=4		11.45511	3	8	3	36.362	5.76	1800000	1700000	1300000	21.79433
Q9DAW9	Calponin-3 OS=Mus musculus GN=Cnn3 PE=1 SV=1		30.30303	8	18	7	36.406	5.72	8600000	4900000	6500000	50.28578
Q9QZD9	Eukaryotic translation initiation factor 3 subunit I OS=Mus musculus GN=Eif3i PE=1 SV=1		27.07692	8	22	8	36.438	5.64	12000000	11000000	10000000	63.53778
Q8CEE7	Retinol dehydrogenase 13 OS=Mus musculus GN=Rdh13 PE=1 SV=1		3.892216	1	3	1	36.441	8.85	920000	940000	610000	7.220531
Q9ERN0	Secretory carrier-associated membrane protein 2 OS=Mus musculus GN=Scamp2 PE=1 SV=1		3.647416	1	1	1	36.441	6.32		3000000		3.139907
P06151	L-lactate dehydrogenase A chain OS=Mus musculus GN=Ldha PE=1 SV=3		23.79518	7	25	6	36.475	7.74	19000000	25000000	14000000	74.10563
P14152	Malate dehydrogenase, cytoplasmic OS=Mus musculus GN=Mdh1 PE=1 SV=3		24.8503	8	20	8	36.488	6.58	21000000	21000000	21000000	53.85393
P26516	26S proteasome non-ATPase regulatory subunit 7 OS=Mus musculus GN=Psmd7 PE=1 SV=2		13.70717	3	8	3	36.517	6.77	4000000	4400000	3300000	22.68604
P16125	L-lactate dehydrogenase B chain OS=Mus musculus GN=Ldhb PE=1 SV=2		24.2515	7	15	6	36.549	6.05	17000000	13000000	14000000	48.4845
Q9JII6	Alcohol dehydrogenase [NADP(+)] OS=Mus musculus GN=Akr1a1 PE=1 SV=3		19.38462	5	11	5	36.564	7.39	5100000	4100000	3000000	32.19096
P61965	WD repeat-containing protein 5 OS=Mus musculus GN=Wdr5 PE=1 SV=1		4.491018	1	1	1	36.565	8.27		840000		2.68865
P01864	Ig gamma-2A chain C region secreted form OS=Mus musculus PE=1 SV=1		9.850746	2	8	2	36.573	8.22	3500000	2700000	2300000	24.37089
P08101	Low affinity immunoglobulin gamma Fc region receptor II OS=Mus musculus GN=Fcgr2 PE=1 SV=2		5.151515	1	3	1	36.673	6.73	4400000	5800000	2400000	10.25098
P25916	Polycomb complex protein BMI-1 OS=Mus musculus GN=Bmi1 PE=1 SV=1		13.58025	2	3	2	36.684	8.65	1800000	1100000		7.841833
P58389	Serine/threonine-protein phosphatase 2A activator OS=Mus musculus GN=Ppp2r4 PE=1 SV=1		4.024768	1	1	1	36.687	6.39		2000000		3.128493
Q6SJQ7	CMRF35-like molecule 1 OS=Mus musculus GN=Cd300lf PE=1 SV=1		3.857567	1	1	1	36.691	5.29	1100000			2.33559
P19973	Lymphocyte-specific protein 1 OS=Mus musculus GN=Lsp1 PE=1 SV=2		36.06061	7	16	7	36.692	4.82	5400000	5100000	2400000	55.35267
Q9CWU9	Nucleoporin Nup37 OS=Mus musculus GN=Nup37 PE=1 SV=2		4.601227	1	1	1	36.708	6.09	1000000			3.505811
Q921M7	Protein FAM49B OS=Mus musculus GN=Fam49b PE=1 SV=1		16.97531	4	10	3	36.753	6.06	1900000	1500000	1600000	28.37868
Q9DB25	Dolichyl-phosphate beta-glucosyltransferase OS=Mus musculus GN=Alg5 PE=1 SV=1		3.703704	1	3	1	36.767	8.6	5400000	5900000	6200000	8.906664
P01878	Ig alpha chain C region OS=Mus musculus PE=1 SV=1		4.360465	1	4	1	36.852	5.06	3900000	2900000	2800000	13.87075
Q9CSU0	Regulation of nuclear pre-mRNA domain-containing protein 1B OS=Mus musculus GN=Rprd1b PE=1 SV=2		4.907975	1	1	1	36.861	5.97			2500000	3.108315
Q920Q6	RNA-binding protein Musashi homolog 2 OS=Mus musculus GN=Msi2 PE=1 SV=1		2.890173	1	3	1	36.915	8.47	2400000	1700000	1800000	8.66397
Q9WVA3	Mitotic checkpoint protein BUB3 OS=Mus musculus GN=Bub3 PE=1 SV=2		27.91411	7	17	7	36.931	6.84	12000000	6700000	7500000	49.78278
P63087	Serine/threonine-protein phosphatase PP1-gamma catalytic subunit OS=Mus musculus GN=Ppp1cc PE=1 SV=1		6.19195	2	6	2	36.96	6.54	4000000	3600000	3300000	14.89418
O35887	Calumenin OS=Mus musculus GN=Calu PE=1 SV=1		44.7619	11	43	11	37.041	4.67	29000000	27000000	28000000	127.0922
P21995	Embigin OS=Mus musculus GN=Emb PE=1 SV=2		6.666667	2	2	2	37.041	6.02	440000	2900000		4.947199
Q8C0L0	Thioredoxin-related transmembrane protein 4 OS=Mus musculus GN=Tmx4 PE=1 SV=2		3.58209	1	3	1	37.108	4.37	2200000	2100000	2300000	7.990436
P62141	Serine/threonine-protein phosphatase PP1-beta catalytic subunit OS=Mus musculus GN=Ppp1cb PE=1 SV=3		6.116208	2	5	1	37.163	6.19	4600000		5900000	11.8654
Q99JW4	LIM and senescent cell antigen-like-containing domain protein 1 OS=Mus musculus GN=Lims1 PE=1 SV=3		8.307692	2	2	2	37.215	8.05	1900000	2200000		2.102888
Q9D020	Cytosolic 5'-nucleotidase 3A OS=Mus musculus GN=Nt5c3a PE=1 SV=4		7.55287	2	4	2	37.228	6.65	1200000	1800000	1600000	13.31387
Q8BP92	Reticulocalbin-2 OS=Mus musculus GN=Rcn2 PE=1 SV=1		4.6875	1	1	1	37.248	4.42			2500000	2.314611
Q91VC9	Growth hormone-inducible transmembrane protein OS=Mus musculus GN=Ghitm PE=1 SV=1		3.179191	1	2	1	37.25	9.8	1000000	740000		5.224179
P10605	Cathepsin B OS=Mus musculus GN=Ctsb PE=1 SV=2		14.45428	4	18	4	37.256	5.91	11000000	7500000	7700000	48.20756
Q5U4D9	THO complex subunit 6 homolog OS=Mus musculus GN=Thoc6 PE=1 SV=1		3.225806	1	1	1	37.291	7.12	280000			2.494646
O88736	3-keto-steroid reductase OS=Mus musculus GN=Hsd17b7 PE=1 SV=1		2.994012	1	1	1	37.293	6.73			510000	1.928748
P29699	Alpha-2-HS-glycoprotein OS=Mus musculus GN=Ahsg PE=1 SV=1		11.01449	2	2	2	37.302	6.51		1000000		9.39505
P62880	Guanine nucleotide-binding protein G(I)/G(S)/G(T) subunit beta-2 OS=Mus musculus GN=Gnb2 PE=1 SV=3		17.64706	5	16	2	37.307	6	21000000	10000000	14000000	52.01389

Q9CXI0	2-methoxy-6-polyprenyl-1,4-benzoquinol methylase, mitochondrial OS=Mus musculus GN=Coq5 PE=1 SV=2		3.669725	1	1	1	37.312	7.49	890000			2.784724
Q8BHZ0	Protein FAM49A OS=Mus musculus GN=Fam49a PE=1 SV=1		7.120743	2	4	1	37.319	6.01		1100000	720000	10.0688
Q9R020	Zinc finger Ran-binding domain-containing protein 2 OS=Mus musculus GN=Zranb2 PE=1 SV=2		14.54545	4	9	4	37.328	9.89	5500000	5900000	4400000	25.46718
Q8BVA5	Lipid droplet-associated hydrolase OS=Mus musculus GN=Ldah PE=1 SV=1		3.98773	1	1	1	37.349	8.28		2600000		2.13082
P62874	Guanine nucleotide-binding protein G(I)/G(S)/G(T) subunit beta-1 OS=Mus musculus GN=Gnb1 PE=1 SV=3		21.47059	6	19	3	37.353	6	20000000	11000000	13000000	59.21294
Q18PI6	SLAM family member 5 OS=Mus musculus GN=Cd84 PE=1 SV=1		3.647416	1	1	1	37.354	7.36		1000000		2.131004
P29387	Guanine nucleotide-binding protein subunit beta-4 OS=Mus musculus GN=Gnb4 PE=1 SV=4		12.35294	3	5	2	37.355	6.16	400000			13.30793
Q93092	Transaldolase OS=Mus musculus GN=Taldo1 PE=1 SV=2		6.824926	2	3	2	37.363	7.03	2200000	1200000		8.020023
O88569	Heterogeneous nuclear ribonucleoproteins A2/B1 OS=Mus musculus GN=Hnrnpa2b1 PE=1 SV=2		59.2068	21	135	21	37.38	8.95	2.3E+08	1.8E+08	2E+08	426.9259
P14430	H-2 class I histocompatibility antigen, Q8 alpha chain OS=Mus musculus GN=H2-Q8 PE=3 SV=1		20.55215	7	34	1	37.387	5.94				98.4491
Q4FK66	Pre-mRNA-splicing factor 38A OS=Mus musculus GN=Prpf38a PE=1 SV=1		11.53846	3	8	3	37.413	10.01	1800000	1700000	3600000	22.15296
P60335	Poly(rC)-binding protein 1 OS=Mus musculus GN=Pcbp1 PE=1 SV=1		27.24719	8	22	4	37.474	7.09	6000000	4200000	6900000	63.90648
Q8R0F5	RNA-binding motif protein, X-linked 2 OS=Mus musculus GN=Rbmx2 PE=1 SV=1		3.98773	1	1	1	37.513	9.72	540000			3.078701
P62137	Serine/threonine-protein phosphatase PP1-alpha catalytic subunit OS=Mus musculus GN=Ppp1ca PE=1 SV=1		6.060606	2	4	1	37.516	6.33		5200000		9.604762
Q99N96	39S ribosomal protein L1, mitochondrial OS=Mus musculus GN=Mrpl1 PE=1 SV=2		4.761905	1	4	1	37.573	8.72	1100000	660000	740000	10.52786
Q9ERD8	Gamma-parvin OS=Mus musculus GN=Parvg PE=1 SV=2		6.344411	2	3	2	37.58	5.66	1800000	1800000		7.484401
O08915	AH receptor-interacting protein OS=Mus musculus GN=Aip PE=1 SV=1		10.30303	2	5	2	37.581	6.4	1500000	920000	880000	15.06855
Q9WUP7	Ubiquitin carboxyl-terminal hydrolase isozyme L5 OS=Mus musculus GN=Uchl5 PE=1 SV=2		16.10942	4	10	4	37.593	5.33	2100000	1800000	1000000	25.52194
Q9CQJ4	E3 ubiquitin-protein ligase RING2 OS=Mus musculus GN=Rnf2 PE=1 SV=1		7.738095	2	5	2	37.6	6.84	1400000	1500000	1200000	12.79556
Q9QYS9	Protein quaking OS=Mus musculus GN=Qki PE=1 SV=1		12.90323	4	11	4	37.647	8.56	7100000	4900000	5600000	26.83741
Q9CWK3	CD2 antigen cytoplasmic tail-binding protein 2 OS=Mus musculus GN=Cd2bp2 PE=1 SV=1		7.017544	2	4	2	37.671	4.6	1800000	2200000	1600000	10.63281
Q6PIU9	Uncharacterized protein FLJ45252 homolog OS=Mus musculus PE=1 SV=2		16.66667	4	8	4	37.681	5.07	1600000	2200000	1800000	25.68249
A6H5X4	PHD finger protein 11 OS=Mus musculus GN=Phf11 PE=2 SV=1		15.727	3	18	3	37.704	6.07	6300000	6600000	3500000	69.81184
Q8K221	Arfaptin-2 OS=Mus musculus GN=Arfip2 PE=1 SV=2		7.331378	2	2	2	37.749	5.87	1000000			5.551447
Q9CQM9	Glutaredoxin-3 OS=Mus musculus GN=Glrx3 PE=1 SV=1		10.68249	3	6	3	37.754	5.59	4600000	3800000	3500000	21.31594
Q9D832	DnaJ homolog subfamily B member 4 OS=Mus musculus GN=Dnajb4 PE=1 SV=1		11.27596	3	6	2	37.758	8.59	2000000	3000000	2700000	17.00712
Q8VDY9	Caspase activity and apoptosis inhibitor 1 OS=Mus musculus GN=Caap1 PE=1 SV=2		4.775281	1	1	1	37.802	4.72			550000	3.04493
Q62313	Trans-Golgi network integral membrane protein 1 OS=Mus musculus GN=Tgoln1 PE=1 SV=1		25.77904	4	7	4	37.826	5.34	2300000	1300000	780000	20.82732
Q9JM96	Cdc42 effector protein 4 OS=Mus musculus GN=Cdc42ep4 PE=1 SV=1		4.297994	1	1	1	37.846	5.36	410000			2.995313
Q8BFZ9	Erlin-2 OS=Mus musculus GN=Erlin2 PE=1 SV=1		19.11765	6	13	4	37.849	5.5	7500000	9200000	6100000	36.77972
Q9QYA2	Mitochondrial import receptor subunit TOM40 homolog OS=Mus musculus GN=Tomm40 PE=1 SV=3		9.972299	3	10	3	37.871	7.74	3400000	1600000	1200000	29.21231
Q80Z24	Neuronal growth regulator 1 OS=Mus musculus GN=Negr1 PE=1 SV=1		5.747126	1	4	1	37.876	6.52	710000	620000	880000	12.62503
P14429	H-2 class I histocompatibility antigen, Q7 alpha chain OS=Mus musculus GN=H2-Q7 PE=1 SV=1		19.46108	6	30	1	37.9	6.29	1100000	3600000	760000	85.38514
Q9CQY5	Magnesium transporter protein 1 OS=Mus musculus GN=Magt1 PE=1 SV=1		5.671642	2	4	2	37.944	9.76	4500000		5100000	9.331123
Q99PJ0	Neurotrimin OS=Mus musculus GN=Ntm PE=1 SV=2		8.72093	2	9	2	37.96	7.81	7900000	4100000	3600000	26.20668
Q9DCH4	Eukaryotic translation initiation factor 3 subunit F OS=Mus musculus GN=Eif3f PE=1 SV=2		6.925208	2	5	2	37.96	5.58	2400000	3500000	2400000	16.56455
Q8R1Z9	RING finger protein 121 OS=Mus musculus GN=Rnf121 PE=1 SV=1		5.198777	1	2	1	37.961	7.06	710000			4.663744
Q8BH97	Reticulocalbin-3 OS=Mus musculus GN=Rcn3 PE=1 SV=1		16.46341	4	7	4	37.978	4.88	940000	1000000	2400000	21.06826
Q99L27	GMP reductase 2 OS=Mus musculus GN=Gmpr2 PE=1 SV=2		4.885057	1	1	1	37.994	7.44			950000	0
Q8K021	Secretory carrier-associated membrane protein 1 OS=Mus musculus GN=Scamp1 PE=1 SV=1		7.100592	2	3	2	38.004	7.71	2300000		1300000	9.620121
Q8BLK3	Limbic system-associated membrane protein OS=Mus musculus GN=Lsamp PE=1 SV=1		15.83578	4	6	4	38.063	6.65	3500000	1400000	680000	18.3665
Q99L45	Eukaryotic translation initiation factor 2 subunit 2 OS=Mus musculus GN=Eif2s2 PE=1 SV=1		43.20242	12	39	12	38.068	5.8	16000000	14000000	12000000	123.02
Q8BR65	Sin3 histone deacetylase corepressor complex component SDS3 OS=Mus musculus GN=Suds3 PE=1 SV=1		7.621951	2	3	2	38.083	5.66	460000		520000	8.74532
Q05186	Reticulocalbin-1 OS=Mus musculus GN=Rcn1 PE=1 SV=1		22.46154	7	13	7	38.09	4.84	6200000	6700000	6900000	42.99046
P40336	Vacuolar protein sorting-associated protein 26A OS=Mus musculus GN=Vps26a PE=1 SV=1		9.174312	2	5	2	38.09	6.57	9100000	6700000	7000000	18.77296
Q8BK64	Activator of 90 kDa heat shock protein ATPase homolog 1 OS=Mus musculus GN=Ahsa1 PE=1 SV=2		7.39645	2	3	2	38.093	5.53		770000		7.740734
Q6P6J9	Thioredoxin domain-containing protein 15 OS=Mus musculus GN=Txndc15 PE=1 SV=1		6.104651	1	3	1	38.122	4.77	800000	520000	600000	5.062422
Q9CX34	Protein SGT1 homolog OS=Mus musculus GN=Sugt1 PE=1 SV=3		15.77381	4	9	4	38.135	5.45	2900000	1800000	1500000	25.70407
Q9QYJ3	DnaJ homolog subfamily B member 1 OS=Mus musculus GN=Dnajb1 PE=1 SV=3		18.82353	5	9	4	38.143	8.63	2700000	940000	2100000	25.79836
Q9CXR1	Dehydrogenase/reductase SDR family member 7 OS=Mus musculus GN=Dhrs7 PE=1 SV=2		5.91716	1	2	1	38.143	8.32	3200000	2200000		8.9784
Q922Q1	Mitochondrial amidoxime reducing component 2 OS=Mus musculus GN=Marc2 PE=1 SV=1		9.467456	3	8	3	38.17	8.68	7100000	7800000	6300000	23.18638
Q8R2Y0	Monoacylglycerol lipase ABHD6 OS=Mus musculus GN=Abhd6 PE=1 SV=1		4.464286	1	3	1	38.18	8.47	2600000	1900000	1900000	12.71053
Q9CYA0	Cysteine-rich with EGF-like domain protein 2 OS=Mus musculus GN=Creld2 PE=1 SV=1		4.571429	1	3	1	38.194	4.58	1900000	1800000	2100000	10.75737

Q61990	Poly(rC)-binding protein 2 OS=Mus musculus GN=Pcbp2 PE=1 SV=1		16.0221	5	18	1	38.197	6.79	4900000	7800000	6000000	54.26385
P51885	Lumican OS=Mus musculus GN=Lum PE=1 SV=2		11.83432	3	5	3	38.241	6.43	7700000	4600000	6900000	13.62608
Q80XN0	D-beta-hydroxybutyrate dehydrogenase, mitochondrial OS=Mus musculus GN=Bdh1 PE=1 SV=2		10.20408	3	7	3	38.274	9.01	2600000	2300000	1100000	19.70187
Q60668	Heterogeneous nuclear ribonucleoprotein D0 OS=Mus musculus GN=Hnrnpd PE=1 SV=2		22.8169	6	22	5	38.33	7.81	47000000	40000000	40000000	63.42538
O35685	Nuclear migration protein nudC OS=Mus musculus GN=Nudc PE=1 SV=1		26.80723	8	21	8	38.334	5.26	5500000	6000000	4700000	60.60597
Q8K4R4	Cytoplasmic phosphatidylinositol transfer protein 1 OS=Mus musculus GN=Pitpnc1 PE=1 SV=1		3.614458	1	1	1	38.359	6.32	1100000			1.817876
Q99JB2	Stomatin-like protein 2, mitochondrial OS=Mus musculus GN=Stoml2 PE=1 SV=1		21.24646	6	14	6	38.361	8.87	5700000	6300000	4000000	45.34358
Q9Z1Z2	Serine-threonine kinase receptor-associated protein OS=Mus musculus GN=Strap PE=1 SV=2		23.71429	6	15	6	38.418	5.12	5700000	4100000	3800000	43.25776
O35609	Secretory carrier-associated membrane protein 3 OS=Mus musculus GN=Scamp3 PE=1 SV=3		9.169054	2	4	2	38.433	7.64	1700000	1600000		12.01932
O70370	Cathepsin S OS=Mus musculus GN=Ctss PE=1 SV=2		8.823529	2	5	2	38.449	6.96	3400000	2500000	1100000	15.99046
Q8R3G1	Nuclear inhibitor of protein phosphatase 1 OS=Mus musculus GN=Ppp1r8 PE=1 SV=1		10.25641	2	5	2	38.504	7.37	810000	5300000	770000	17.58696
Q9JKF7	39S ribosomal protein L39, mitochondrial OS=Mus musculus GN=Mrpl39 PE=1 SV=4		3.571429	1	1	1	38.525	7.94	1800000			2.68607
P70441	Na(+)/H(+) exchange regulatory cofactor NHE-RF1 OS=Mus musculus GN=Slc9a3r1 PE=1 SV=3		9.295775	2	4	2	38.577	5.9	3800000	2800000		13.45981
Q80V26	Inositol monophosphatase 3 OS=Mus musculus GN=Impad1 PE=1 SV=1		3.932584	1	1	1	38.592	6.47		1300000		2.840431
Q9R1T2	SUMO-activating enzyme subunit 1 OS=Mus musculus GN=Sae1 PE=1 SV=1		8.857143	2	3	2	38.596	5.36		620000	660000	12.26846
Q9DBU6	Serine/Arginine-related protein 53 OS=Mus musculus GN=Rsrc1 PE=1 SV=1		8.383234	1	3	1	38.614	11.15	660000	1800000	1100000	11.43872
P07356	Annexin A2 OS=Mus musculus GN=Anxa2 PE=1 SV=2		42.77286	15	44	15	38.652	7.69	15000000	14000000	17000000	145.61
Q91XC9	Peroxisomal membrane protein PEX16 OS=Mus musculus GN=Pex16 PE=1 SV=2		6.845238	1	1	1	38.653	9.72		940000		2.685119
Q9WUK4	Replication factor C subunit 2 OS=Mus musculus GN=Rfc2 PE=1 SV=1		3.724928	1	1	1	38.7	6.44	2000000			3.143086
P10107	Annexin A1 OS=Mus musculus GN=Anxa1 PE=1 SV=2		30.63584	9	25	9	38.71	7.37	20000000	16000000	16000000	81.56007
Q9R226	KH domain-containing, RNA-binding, signal transduction-associated protein 3 OS=Mus musculus GN=Khdrbs3 PE=1 SV=1		5.780347	2	5	1	38.784	8.1	560000		310000	9.614548
Q9JKB3	Y-box-binding protein 3 OS=Mus musculus GN=Ybx3 PE=1 SV=2		28.25485	7	24	4	38.79	9.69	3900000	3300000	3300000	84.93537
Q99LD9	Translation initiation factor eIF-2B subunit beta OS=Mus musculus GN=Eif2b2 PE=1 SV=1		5.128205	1	1	1	38.873	6.24		350000		2.352975
Q8BK63	Casein kinase I isoform alpha OS=Mus musculus GN=Csnk1a1 PE=1 SV=2		12.16617	3	5	3	38.89	9.57	830000	1400000	1100000	13.26671
Q9D051	Pyruvate dehydrogenase E1 component subunit beta, mitochondrial OS=Mus musculus GN=Pdhb PE=1 SV=1		31.47632	9	23	9	38.912	6.87	17000000	14000000	8500000	71.89628
Q91X78	Erlin-1 OS=Mus musculus GN=Erlin1 PE=1 SV=1		13.58382	5	8	3	38.912	7.21		1600000	2100000	19.48194
Q69ZC8	GPALPP motifs-containing protein 1 OS=Mus musculus GN=Gpalpp1 PE=1 SV=2		3.757225	1	1	1	38.931	5.41				2.511145
Q61249	Immunoglobulin-binding protein 1 OS=Mus musculus GN=Igbp1 PE=1 SV=1		4.411765	1	3	1	38.947	6.18	1300000	1300000	1600000	10.27026
Q9DAR7	m7GpppX diphosphatase OS=Mus musculus GN=Dcps PE=1 SV=1		13.31361	3	4	3	38.964	6.48	820000	1600000	530000	13.06385
P30993	C5a anaphylatoxin chemotactic receptor 1 OS=Mus musculus GN=C5ar1 PE=1 SV=2		3.418803	1	3	1	38.998	8.79	880000	1100000	780000	7.179169
Q3TWW8	Serine/arginine-rich splicing factor 6 OS=Mus musculus GN=Srsf6 PE=1 SV=1		22.71386	8	29	5	39.002	11.46	14000000	15000000	16000000	79.77219
Q9CYN9	Renin receptor OS=Mus musculus GN=Atp6ap2 PE=1 SV=2		3.714286	1	1	1	39.067	5.54	730000			2.435432
P52432	DNA-directed RNA polymerases I and III subunit RPAC1 OS=Mus musculus GN=Polr1c PE=1 SV=3		8.381503	2	5	2	39.082	5.21	1100000	940000	1500000	17.61065
Q8BYZ1	ABI gene family member 3 OS=Mus musculus GN=Abi3 PE=1 SV=3		4.087193	1	2	1	39.082	5.54	1100000	1300000		6.141532
Q8C0E2	Vacuolar protein sorting-associated protein 26B OS=Mus musculus GN=Vps26b PE=1 SV=1		11.0119	3	5	3	39.1	7.37	5700000	4500000	4000000	15.84825
P24452	Macrophage-capping protein OS=Mus musculus GN=Capg PE=1 SV=2		19.03409	6	18	6	39.216	7.2	16000000	16000000	14000000	60.03792
Q6PE01	U5 small nuclear ribonucleoprotein 40 kDa protein OS=Mus musculus GN=Snrnp40 PE=1 SV=1		10.3352	4	8	4	39.251	8.1	3300000	2900000	2100000	23.71313
Q8CFE2	Histone PARylation factor 1 OS=Mus musculus GN=Hpf1 PE=1 SV=1		10.11561	3	7	3	39.266	7.69	2600000	1600000	2000000	15.3726
Q62087	Serum paraoxonase/lactonase 3 OS=Mus musculus GN=Pon3 PE=1 SV=2		7.344633	2	5	2	39.326	5.74	2500000	1600000	3100000	11.31972
P05064	Fructose-bisphosphate aldolase A OS=Mus musculus GN=Aldoa PE=1 SV=2		55.76923	20	72	18	39.331	8.09	1.1E+08	92000000	75000000	231.8192
Q9EPK2	Protein XRP2 OS=Mus musculus GN=Rp2 PE=1 SV=3		2.305476	1	1	1	39.351	5.24		1200000		1.833826
O70126	Aurora kinase B OS=Mus musculus GN=Aurkb PE=1 SV=2		3.478261	1	1	1	39.36	9.44		1100000		1.999176
P05063	Fructose-bisphosphate aldolase C OS=Mus musculus GN=Aldoc PE=1 SV=4		42.97521	13	37	11	39.37	7.12	24000000	19000000	20000000	115.131
P23906	Interferon regulatory factor 2 OS=Mus musculus GN=Irf2 PE=1 SV=1		3.724928	1	2	1	39.428	7.49	830000	650000		5.914982
Q9Z0P5	Twinfilin-2 OS=Mus musculus GN=Twf2 PE=1 SV=1		10.88825	2	2	2	39.446	6.8		2700000	340000	8.427547
Q9JHJ0	Tropomodulin-3 OS=Mus musculus GN=Tmod3 PE=1 SV=1		14.77273	4	5	4	39.478	5.14	1100000	5200000	2300000	12.47968
Q9JKK7	Tropomodulin-2 OS=Mus musculus GN=Tmod2 PE=1 SV=2		8.262108	2	4	2	39.487	5.35	1700000	2300000	1400000	13.77969
Q62086	Serum paraoxonase/arylesterase 2 OS=Mus musculus GN=Pon2 PE=1 SV=2		14.40678	4	8	4	39.592	5.83	3900000	3300000	2000000	24.36095
Q8VEM8	Phosphate carrier protein, mitochondrial OS=Mus musculus GN=Slc25a3 PE=1 SV=1		16.80672	6	19	6	39.606	9.26	42000000	42000000	46000000	48.50335
Q9D6R2	Isocitrate dehydrogenase [NAD] subunit alpha, mitochondrial OS=Mus musculus GN=Idh3a PE=1 SV=1		22.6776	7	15	7	39.613	6.73	11000000	7000000	6300000	38.21211
Q8BG05	Heterogeneous nuclear ribonucleoprotein A3 OS=Mus musculus GN=Hnrnpa3 PE=1 SV=1		38.78628	14	68	14	39.628	9.01	1.2E+08	1.1E+08	1.3E+08	222.4214
P70460	Vasodilator-stimulated phosphoprotein OS=Mus musculus GN=Vasp PE=1 SV=4		24.26667	7	18	7	39.642	8.53	5300000	4100000	4400000	44.36422
Q3UMR5	Calcium uniporter protein, mitochondrial OS=Mus musculus GN=Mcu PE=1 SV=2		7.428571	2	4	2	39.657	8.56	2700000	1900000	1400000	10.49778

Q8K1E0	Syntaxin-5 OS=Mus musculus GN=Stx5 PE=1 SV=3		5.070423	1	2	1	39.689	8.92		550000	650000	6.332336
Q9QZQ8	Core histone macro-H2A.1 OS=Mus musculus GN=H2afy PE=1 SV=3		25	8	30	7	39.71	9.8	21000000	18000000	19000000	100.8379
Q8R2U0	Nucleoporin SEH1 OS=Mus musculus GN=Seh1l PE=2 SV=1		7.222222	2	5	2	39.749	8.05	2000000	1500000	1400000	14.29838
Q9D880	Mitochondrial import inner membrane translocase subunit TIM50 OS=Mus musculus GN=Timm50 PE=1 SV=1		12.46459	3	7	3	39.752	8.13	5400000	5700000	3300000	22.64361
O54946	DnaJ homolog subfamily B member 6 OS=Mus musculus GN=Dnajb6 PE=1 SV=4		6.30137	2	4	1	39.783	9.36	6900000			12.09608
Q91WK2	Eukaryotic translation initiation factor 3 subunit H OS=Mus musculus GN=Eif3h PE=1 SV=1		13.92045	4	13	4	39.807	6.67	6500000	7400000	6800000	43.48849
Q06138	Calcium-binding protein 39 OS=Mus musculus GN=Cab39 PE=1 SV=2		4.398827	1	1	1	39.818	6.89	430000			2.232907
Q9QXV1	Chromobox protein homolog 8 OS=Mus musculus GN=Cbx8 PE=1 SV=1		6.077348	1	1	1	39.836	9.94		1100000		5.198928
Q99J62	Replication factor C subunit 4 OS=Mus musculus GN=Rfc4 PE=1 SV=1		5.769231	2	4	2	39.842	6.7	1400000	1600000	1700000	10.91086
Q9JHK5	Pleckstrin OS=Mus musculus GN=Plek PE=1 SV=1		7.714286	2	4	2	39.876	8.34	2500000	2800000	2300000	9.260718
Q8BTZ7	Mannose-1-phosphate guanyltransferase beta OS=Mus musculus GN=Gmppb PE=1 SV=1		6.666667	2	4	2	39.891	6.74	1000000	860000	800000	12.81852
Q9CX56	26S proteasome non-ATPase regulatory subunit 8 OS=Mus musculus GN=Psmd8 PE=1 SV=2		2.832861	1	2	1	39.905	9.58	3300000	2900000		2.073785
Q62420	Endophilin-A1 OS=Mus musculus GN=Sh3gl2 PE=1 SV=2		21.59091	6	21	4	39.93	5.39	9700000	6600000	6600000	59.36609
O08573	Galectin-9 OS=Mus musculus GN=Lgals9 PE=1 SV=1		3.68272	1	3	1	40.01	9.31	4700000	3800000	4000000	9.862791
Q7TND5	Ribosome production factor 1 OS=Mus musculus GN=Rpf1 PE=2 SV=2		7.163324	1	1	1	40.012	9.99		620000		3.06327
Q9Z1S5	Neuronal-specific septin-3 OS=Mus musculus GN=Sept3 PE=1 SV=2		9.428571	3	3	3	40.013	6.81	1300000	3000000		7.555472
P15702	Leukosialin OS=Mus musculus GN=Spn PE=1 SV=1		10.37975	2	5	2	40.014	4.96	1900000	4400000	3100000	19.68178
Q8C7Q4	RNA-binding protein 4 OS=Mus musculus GN=Rbm4 PE=1 SV=1		4.432133	1	3	1	40.02	7.06	760000	590000	750000	9.77931
Q91YR1	Twinfilin-1 OS=Mus musculus GN=Twf1 PE=1 SV=2		10.57143	3	6	3	40.054	6.67	2800000	2300000	3100000	19.83006
P18872	Guanine nucleotide-binding protein G(o) subunit alpha OS=Mus musculus GN=Gnao1 PE=1 SV=3		30.50847	10	45	9	40.059	5.53	77000000	39000000	34000000	120.4923
Q8CCK0	Core histone macro-H2A.2 OS=Mus musculus GN=H2afy2 PE=1 SV=3		8.333333	2	3	1	40.067	9.69		1200000		9.199392
Q61559	IgG receptor FcRn large subunit p51 OS=Mus musculus GN=Fcgrt PE=1 SV=1		2.465753	1	1	1	40.067	5.29	1100000			2.13216
P55264	Adenosine kinase OS=Mus musculus GN=Adk PE=1 SV=2		2.216066	1	3	1	40.123	6.21		1800000	920000	7.184405
Q8R409	Protein HEXIM1 OS=Mus musculus GN=Hexim1 PE=1 SV=1		4.775281	1	2	1	40.219	5.38	860000	1100000		8.088418
P51863	V-type proton ATPase subunit d 1 OS=Mus musculus GN=Atp6v0d1 PE=1 SV=2		14.24501	5	11	5	40.275	5	8800000	9600000	7700000	27.61844
P21855	B-cell differentiation antigen CD72 OS=Mus musculus GN=Cd72 PE=1 SV=2		3.389831	1	2	1	40.322	6.43	1500000	970000		5.988733
B2RSH2	Guanine nucleotide-binding protein G(i) subunit alpha-1 OS=Mus musculus GN=Gnai1 PE=1 SV=1		26.55367	8	24	4	40.335	5.97	4300000	1100000	2200000	63.20175
Q9D8N2	Protein FAM45A OS=Mus musculus GN=Fam45a PE=1 SV=2		9.243697	2	2	2	40.395	6.47		1100000	650000	6.170751
Q9DBH5	Vesicular integral-membrane protein VIP36 OS=Mus musculus GN=Lman2 PE=1 SV=2		17.87709	5	13	5	40.404	6.95	6400000	2800000	7200000	38.56522
Q99K85	Phosphoserine aminotransferase OS=Mus musculus GN=Psat1 PE=1 SV=1		11.62162	4	9	4	40.447	8.03	3600000	3700000	3700000	23.68916
Q9QUP5	Hyaluronan and proteoglycan link protein 1 OS=Mus musculus GN=Hapln1 PE=1 SV=1		8.146067	3	4	3	40.452	7.8	2200000	1300000		9.670561
P08752	Guanine nucleotide-binding protein G(i) subunit alpha-2 OS=Mus musculus GN=Gnai2 PE=1 SV=5		31.26761	9	38	5	40.463	5.45	30000000	21000000	15000000	122.0488
Q8VHL1	Histone-lysine N-methyltransferase SETD7 OS=Mus musculus GN=Setd7 PE=1 SV=2		5.464481	1	1	1	40.481	4.65				1.848361
P32233	Developmentally-regulated GTP-binding protein 1 OS=Mus musculus GN=Drg1 PE=1 SV=1		11.71662	3	7	3	40.487	8.9	1100000	1400000	1400000	27.4387
Q8R323	Replication factor C subunit 3 OS=Mus musculus GN=Rfc3 PE=1 SV=1		2.52809	1	1	1	40.5	8.38		1800000		2.264391
Q9DC51	Guanine nucleotide-binding protein G(k) subunit alpha OS=Mus musculus GN=Gnai3 PE=1 SV=3		16.94915	5	17	2	40.512	5.69	2800000	2200000	1800000	44.92998
Q99KV1	DnaJ homolog subfamily B member 11 OS=Mus musculus GN=Dnajb11 PE=1 SV=1		30.44693	7	24	7	40.53	6.32	12000000	12000000	10000000	83.00828
P05132	cAMP-dependent protein kinase catalytic subunit alpha OS=Mus musculus GN=Prkaca PE=1 SV=3		9.401709	3	4	1	40.545	8.79	190000			8.214645
Q99LC3	NADH dehydrogenase [ubiquinone] 1 alpha subcomplex subunit 10, mitochondrial OS=Mus musculus GN=Ndufa10 PE=1 SV=1		9.859155	3	5	3	40.578	7.78	7900000	1900000	1700000	13.18146
O35226	26S proteasome non-ATPase regulatory subunit 4 OS=Mus musculus GN=Psmd4 PE=1 SV=1		14.89362	3	9	3	40.678	4.79	2100000	4800000	4300000	36.68654
P68181	cAMP-dependent protein kinase catalytic subunit beta OS=Mus musculus GN=Prkacb PE=1 SV=2		9.401709	3	5	1	40.682	8.56	690000		360000	11.10716
Q9CZ44	NSFL1 cofactor p47 OS=Mus musculus GN=Nsfl1c PE=1 SV=1		34.32432	9	26	9	40.685	5.15	7300000	5100000	5200000	85.12438
Q3UND0	Src kinase-associated phosphoprotein 2 OS=Mus musculus GN=Skap2 PE=1 SV=2		18.15642	5	10	5	40.687	4.7	2300000	2500000	2100000	22.03733
Q8VC70	RNA-binding motif, single-stranded-interacting protein 2 OS=Mus musculus GN=Rbms2 PE=1 SV=1		5.744125	1	1	1	40.698	9.07		130000		2.860252
Q9CR16	Peptidyl-prolyl cis-trans isomerase D OS=Mus musculus GN=Ppid PE=1 SV=3		13.51351	4	14	4	40.717	7.43	6600000	4700000	4200000	39.09943
Q9D5T0	ATPase family AAA domain-containing protein 1 OS=Mus musculus GN=Atad1 PE=1 SV=1		9.418283	2	4	2	40.718	6.9	1900000	2000000	3300000	10.08538
Q5SS80	Dehydrogenase/reductase SDR family member 13 OS=Mus musculus GN=Dhrs13 PE=1 SV=1		9.308511	1	1	1	40.719	7.93		1300000		2.952105
Q8C1E7	Transmembrane protein 120A OS=Mus musculus GN=Tmem120a PE=1 SV=1		3.206997	1	1	1	40.725	8.88		350000		2.455332
Q9QYG0	Protein NDRG2 OS=Mus musculus GN=Ndrg2 PE=1 SV=1		12.93801	3	5	3	40.763	5.4	3500000	690000	1800000	16.20125
P01899	H-2 class I histocompatibility antigen, D-B alpha chain OS=Mus musculus GN=H2-D1 PE=1 SV=2		47.23757	20	92	15	40.81	6.73	77000000	62000000	59000000	271.9714
Q9JK48	Endophilin-B1 OS=Mus musculus GN=Sh3glb1 PE=1 SV=1		9.589041	4	10	3	40.83	6.04	4500000	2900000	2600000	23.83542
P06339	H-2 class I histocompatibility antigen, D-37 alpha chain OS=Mus musculus GN=H2-T23 PE=1 SV=1		9.52381	3	10	2	40.849	6.35	4500000	6100000	3100000	28.29234
Q8R574	Phosphoribosyl pyrophosphate synthase-associated protein 2 OS=Mus musculus GN=Prpsap2 PE=1 SV=1		2.710027	1	3	1	40.854	7.17	460000	420000	320000	6.811187

Q8R1N4	NudC domain-containing protein 3 OS=Mus musculus GN=Nudcd3 PE=1 SV=3		4.132231	1	1	1	40.865	5.26		960000		2.593429
P13011	Acyl-CoA desaturase 2 OS=Mus musculus GN=Scd2 PE=1 SV=2		8.100559	2	3	2	40.89	9.01	450000	340000		9.412795
Q8C570	mRNA export factor OS=Mus musculus GN=Rae1 PE=1 SV=1		6.25	2	3	2	40.939	7.83	900000			6.691265
Q64314	Hematopoietic progenitor cell antigen CD34 OS=Mus musculus GN=Cd34 PE=1 SV=1		2.094241	1	1	1	40.957	5.3		740000		2.002149
P62482	Voltage-gated potassium channel subunit beta-2 OS=Mus musculus GN=Kcnab2 PE=1 SV=1		2.724796	1	1	1	40.995	9	1100000			2.600045
Q8VEK0	Cell cycle control protein 50A OS=Mus musculus GN=Tmem30a PE=1 SV=1		4.67033	1	3	1	41.035	8.37	1900000	1500000	1200000	11.06643
Q9WV32	Actin-related protein 2/3 complex subunit 1B OS=Mus musculus GN=Arpc1b PE=1 SV=4		25	6	23	6	41.037	8.35	31000000	36000000	26000000	84.56065
Q9CXW2	28S ribosomal protein S22, mitochondrial OS=Mus musculus GN=Mrps22 PE=1 SV=1		6.128134	2	4	2	41.167	8.56	1300000	860000	990000	10.40528
Q9R0A0	Peroxisomal membrane protein PEX14 OS=Mus musculus GN=Pex14 PE=1 SV=1		17.81915	3	7	3	41.183	5.11	1900000	2300000	1500000	25.01206
O54833	Casein kinase II subunit alpha' OS=Mus musculus GN=Csnk2a2 PE=1 SV=1		10	2	5	2	41.189	8.56	1800000	1300000	620000	14.03114
P70279	Surfeit locus protein 6 OS=Mus musculus GN=Surf6 PE=1 SV=1		14.08451	5	8	5	41.21	10.52	2500000	3200000	3700000	18.24383
Q9DCA5	Ribosome biogenesis protein BRX1 homolog OS=Mus musculus GN=Brix1 PE=1 SV=3		3.116147	1	1	1	41.215	10.02		930000		3.023992
P63085	Mitogen-activated protein kinase 1 OS=Mus musculus GN=Mapk1 PE=1 SV=3		13.96648	5	15	3	41.249	6.98	8800000	7700000	6300000	40.60252
P47811	Mitogen-activated protein kinase 14 OS=Mus musculus GN=Mapk14 PE=1 SV=3		2.222222	1	1	1	41.261	5.88		930000		1.800438
P01901	H-2 class I histocompatibility antigen, K-B alpha chain OS=Mus musculus GN=H2-K1 PE=1 SV=1		42.54743	13	63	4	41.276	6.39	41000000	34000000	33000000	204.2889
O88741	Ganglioside-induced differentiation-associated protein 1 OS=Mus musculus GN=Gdap1 PE=1 SV=1		4.189944	1	2	1	41.285	8.37	3100000	1200000		7.645643
Q8C5L7	RNA-binding protein 34 OS=Mus musculus GN=Rbm34 PE=1 SV=1		5.6	1	2	1	41.301	9.64			1800000	6.356618
Q9D5V6	Synapse-associated protein 1 OS=Mus musculus GN=Syap1 PE=1 SV=1		4.657534	1	2	1	41.324	4.54	790000	880000		7.138016
Q9Z2Y3	Homer protein homolog 1 OS=Mus musculus GN=Homer1 PE=1 SV=2		3.278689	1	3	1	41.388	5.53	1600000	1400000	1300000	8.075177
P60762	Mortality factor 4-like protein 1 OS=Mus musculus GN=Morf4l1 PE=1 SV=2		3.867403	1	2	1	41.467	9.32		1300000	730000	6.276323
Q62419	Endophilin-A2 OS=Mus musculus GN=Sh3gl1 PE=1 SV=1		16.30435	5	13	3	41.492	5.72	2200000	1200000	1500000	33.87878
P42208	Septin-2 OS=Mus musculus GN=Sept2 PE=1 SV=2		36.84211	10	28	10	41.499	6.55	16000000	8800000	12000000	89.26351
Q9CYH6	Ribosome biogenesis regulatory protein homolog OS=Mus musculus GN=Rrs1 PE=1 SV=1		15.34247	4	10	4	41.526	10.77	3700000	4100000	4300000	29.37663
Q9QYF9	Protein NDRG3 OS=Mus musculus GN=Ndrg3 PE=1 SV=1		5.866667	1	1	1	41.529	5.25	970000			3.224146
Q3TVP5	Inactive ubiquitin thioesterase FAM105A OS=Mus musculus GN=Fam105a PE=1 SV=1		2.266289	1	2	1	41.549	9.22		750000	660000	3.808248
Q9Z0P4	Paralemmin-1 OS=Mus musculus GN=Palm PE=1 SV=1		33.94256	8	23	8	41.589	4.84	10000000	5700000	3500000	79.82589
Q9R0Q6	Actin-related protein 2/3 complex subunit 1A OS=Mus musculus GN=Arpc1a PE=1 SV=1		2.162162	1	1	1	41.6	8.18			2000000	1.830558
P28653	Biglycan OS=Mus musculus GN=Bgn PE=1 SV=1		25.74526	8	19	8	41.613	7.27	14000000	15000000	13000000	60.11317
P04223	H-2 class I histocompatibility antigen, K-K alpha chain OS=Mus musculus GN=H2-K1 PE=1 SV=1		25.20325	8	26	1	41.62	7.03			380000	80.43783
Q99L47	Hsc70-interacting protein OS=Mus musculus GN=St13 PE=1 SV=1		18.86792	6	22	6	41.629	5.26	23000000	16000000	20000000	67.44459
P49586	Choline-phosphate cytidylyltransferase A OS=Mus musculus GN=Pcyt1a PE=1 SV=1		7.901907	2	5	2	41.641	7.03	2700000	1800000	1700000	13.89426
Q9ES46	Beta-parvin OS=Mus musculus GN=Parvb PE=1 SV=1		7.39726	2	4	1	41.643	6.33		450000		12.26346
P60710	Actin, cytoplasmic 1 OS=Mus musculus GN=Actb PE=1 SV=1	x	62.4	24	248	10	41.71	5.48	1.5E+09	1.5E+09	1.1E+09	722.5032
Q9D8V0	Minor histocompatibility antigen H13 OS=Mus musculus GN=Hm13 PE=1 SV=1		18.25397	6	18	6	41.721	6.04	6500000	8500000	8700000	50.37245
Q9JKN1	Zinc transporter 7 OS=Mus musculus GN=Slc30a7 PE=1 SV=1		3.968254	1	1	1	41.763	6.84				2.177146
P48759	Pentraxin-related protein PTX3 OS=Mus musculus GN=Ptx3 PE=1 SV=2		12.07349	4	7	4	41.785	5.38	3400000	3000000	5100000	17.48293
P97441	Zinc transporter 3 OS=Mus musculus GN=Slc30a3 PE=1 SV=1		3.865979	1	1	1	41.797	6.38	1900000			2.021428
Q8BWT1	3-ketoacyl-CoA thiolase, mitochondrial OS=Mus musculus GN=Acaa2 PE=1 SV=3		11.33501	3	6	3	41.803	8.09	2700000	1900000	2000000	18.65993
Q91VA6	Polymerase delta-interacting protein 2 OS=Mus musculus GN=Poldip2 PE=1 SV=1		3.26087	1	2	1	41.844	8.63	750000	480000		4.913447
Q5SSK3	Transcription elongation factor, mitochondrial OS=Mus musculus GN=Tefm PE=1 SV=1		2.197802	1	1	1	41.847	9.51		550000		2.482957
Q9QYI4	DnaJ homolog subfamily B member 12 OS=Mus musculus GN=Dnajb12 PE=1 SV=2		9.042553	2	4	2	41.962	8.51	1200000	1300000		14.22456
P59235	Nucleoporin Nup43 OS=Mus musculus GN=Nup43 PE=1 SV=2		4.210526	1	2	1	41.963	5.39		530000	480000	6.994075
Q6NSU3	Glycosyltransferase 8 domain-containing protein 1 OS=Mus musculus GN=Glt8d1 PE=1 SV=1		3.773585	1	3	1	41.964	9.41	520000	540000	530000	7.674221
Q8R2G4	Ecto-ADP-ribosyltransferase 3 OS=Mus musculus GN=Art3 PE=1 SV=2		3.773585	1	2	1	41.988	6.02	400000	270000		4.231016
P68033	Actin, alpha cardiac muscle 1 OS=Mus musculus GN=Actc1 PE=1 SV=1		43.23607	18	198	4	41.992	5.39	1.3E+08	1.5E+08	1.5E+08	511.6285
P21278	Guanine nucleotide-binding protein subunit alpha-11 OS=Mus musculus GN=Gna11 PE=1 SV=1		16.71309	5	9	2	41.997	5.97	1000000	1200000	630000	23.45828
Q9JKV1	Proteasomal ubiquitin receptor ADRM1 OS=Mus musculus GN=Adrm1 PE=1 SV=2		8.599509	2	7	2	42.034	5.07	5800000	3100000	2600000	20.03599
Q61112	45 kDa calcium-binding protein OS=Mus musculus GN=Sdf4 PE=1 SV=1		3.601108	1	2	1	42.037	4.96	1700000		1700000	5.838755
Q9DCE5	p21-activated protein kinase-interacting protein 1 OS=Mus musculus GN=Pak1ip1 PE=1 SV=2		6.544503	2	4	2	42.089	8.47	2400000	2400000	1800000	13.40377
P15105	Glutamine synthetase OS=Mus musculus GN=Glul PE=1 SV=6		26.27346	7	19	7	42.092	7.08	15000000	10000000	8800000	61.23182
Q61048	WW domain-binding protein 4 OS=Mus musculus GN=Wbp4 PE=1 SV=4		3.191489	1	2	1	42.111	7.36	1000000	1800000		4.530951
P21279	Guanine nucleotide-binding protein G(q) subunit alpha OS=Mus musculus GN=Gnaq PE=1 SV=4		20.05571	6	14	3	42.131	5.68	4200000	1900000	2200000	36.28956
P55302	Alpha-2-macroglobulin receptor-associated protein OS=Mus musculus GN=Lrpap1 PE=1 SV=1		10.55556	3	8	3	42.189	7.87	2300000	2000000	2300000	24.14184

Q9QXY9	Peroxisomal biogenesis factor 3 OS=Mus musculus GN=Pex3 PE=1 SV=1		2.688172	1	2	1	42.197	7.77				5.459937
Q8BVQ5	Protein phosphatase methylesterase 1 OS=Mus musculus GN=Ppme1 PE=1 SV=5		3.108808	1	2	1	42.229	5.97		330000	760000	4.851942
Q8R5C5	Beta-centractin OS=Mus musculus GN=Actr1b PE=1 SV=1		17.28723	5	9	1	42.255	6.4		3000000	2200000	25.34994
Q9WV02	RNA-binding motif protein, X chromosome OS=Mus musculus GN=Rbmx PE=1 SV=1		17.13555	6	15	6	42.275	10.05	21000000	25000000	16000000	37.36424
P39749	Flap endonuclease 1 OS=Mus musculus GN=Fen1 PE=1 SV=1		10.05291	3	9	3	42.288	8.34	4300000	7100000	7400000	30.97718
Q9EPC1	Alpha-parvin OS=Mus musculus GN=Parva PE=1 SV=1		11.02151	3	8	2	42.304	5.95	3600000	3100000	2400000	27.86357
Q9JJA9	General receptor for phosphoinositides 1-associated scaffold protein OS=Mus musculus GN=Grasp PE=1 SV=2		1.785714	1	1	1	42.311	9.1		3900000		2.450954
P11928	2'-5'-oligoadenylate synthase 1A OS=Mus musculus GN=Oas1a PE=1 SV=2		25.3406	7	17	7	42.402	8.09	7200000	6000000	6500000	48.28705
P49443	Protein phosphatase 1A OS=Mus musculus GN=Ppm1a PE=1 SV=1		4.712042	1	1	1	42.406	5.36	2200000			2.853265
Q9JIF0	Protein arginine N-methyltransferase 1 OS=Mus musculus GN=Prmt1 PE=1 SV=1		10.78167	4	10	4	42.408	5.43	10000000	9500000	8400000	27.58548
P18572	Basigin OS=Mus musculus GN=Bsg PE=1 SV=2		18.76607	6	21	6	42.418	5.85	16000000	13000000	12000000	62.20904
Q9DC69	NADH dehydrogenase [ubiquinone] 1 alpha subcomplex subunit 9, mitochondrial OS=Mus musculus GN=Ndufa9 PE=1 SV=2		5.30504	2	6	2	42.498	9.74	3600000	4800000	2400000	15.17329
Q9DBZ1	Inhibitor of nuclear factor kappa-B kinase-interacting protein OS=Mus musculus GN=Ikbip PE=1 SV=2		11.52815	4	7	4	42.505	5.1	3800000	2400000	2200000	20.92715
Q91V12	Cytosolic acyl coenzyme A thioester hydrolase OS=Mus musculus GN=Acot7 PE=1 SV=2		3.149606	1	2	1	42.51	8.68	1700000		1200000	5.855264
P19426	Negative elongation factor E OS=Mus musculus GN=Nelfe PE=1 SV=2		13.33333	3	3	3	42.528	9.38	1100000	1100000		9.290947
P61164	Alpha-centractin OS=Mus musculus GN=Actr1a PE=1 SV=1		15.69149	5	9	1	42.587	6.64	2400000	460000	780000	23.98668
Q8BHS6	Armadillo repeat-containing X-linked protein 3 OS=Mus musculus GN=Armcx3 PE=1 SV=1		4.485488	1	3	1	42.593	8.68	570000	620000	680000	8.474073
O35730	E3 ubiquitin-protein ligase RING1 OS=Mus musculus GN=Ring1 PE=1 SV=2		12.80788	3	7	3	42.604	5.74	470000	750000	790000	25.21896
Q8VCG3	WD repeat-containing protein 74 OS=Mus musculus GN=Wdr74 PE=2 SV=1		9.635417	3	5	3	42.61	7.78	1200000	250000		11.89291
Q78ZA7	Nucleosome assembly protein 1-like 4 OS=Mus musculus GN=Nap1l4 PE=1 SV=1		12.26667	3	8	2	42.653	4.67	6500000	4500000	4100000	24.59047
Q04447	Creatine kinase B-type OS=Mus musculus GN=Ckb PE=1 SV=1		37.27034	12	56	12	42.686	5.67	83000000	55000000	68000000	191.0006
Q8VDV3	Guanine nucleotide exchange factor for Rab-3A OS=Mus musculus GN=Rab3il1 PE=1 SV=1		8.093995	1	1	1	42.686	6.38				2.192412
P70274	Selenoprotein P OS=Mus musculus GN=Sepp1 PE=1 SV=3		2.105263	1	1	1	42.689	7.09	260000			1.818849
Q8R464	Cell adhesion molecule 4 OS=Mus musculus GN=Cadm4 PE=1 SV=1		8.505155	2	3	2	42.697	6.3	1000000	3400000	3500000	11.91591
Q9Z2Q6	Septin-5 OS=Mus musculus GN=Sept5 PE=1 SV=2		18.15718	5	14	5	42.721	6.67	7800000	4300000	3900000	41.76708
Q99J95	Cyclin-dependent kinase 9 OS=Mus musculus GN=Cdk9 PE=1 SV=1		8.333333	3	10	2	42.734	8.79	760000	440000	570000	25.31774
P70404	Isocitrate dehydrogenase [NAD] subunit gamma 1, mitochondrial OS=Mus musculus GN=Idh3g PE=1 SV=1		7.888041	2	5	2	42.758	9.01	5100000	2300000	6000000	13.80859
Q9WVJ2	26S proteasome non-ATPase regulatory subunit 13 OS=Mus musculus GN=Psmd13 PE=1 SV=1		12.5	4	8	4	42.782	5.71	3500000	1500000	2100000	20.19292
Q3TBT3	Stimulator of interferon genes protein OS=Mus musculus GN=Tmem173 PE=1 SV=2		12.43386	3	7	3	42.802	7.42	4800000	5200000		22.13957
Q99M51	Cytoplasmic protein NCK1 OS=Mus musculus GN=Nck1 PE=1 SV=1		5.039788	2	5	2	42.863	6.47	1000000	1100000	1100000	11.73416
Q99N28	Cell adhesion molecule 3 OS=Mus musculus GN=Cadm3 PE=1 SV=1		6.565657	2	5	2	42.938	5.8	4100000	1900000	1500000	19.54998
P23242	Gap junction alpha-1 protein OS=Mus musculus GN=Gja1 PE=1 SV=2		14.92147	3	6	3	42.977	8.76	1700000	1100000	1200000	16.75455
Q8QZS1	3-hydroxyisobutyryl-CoA hydrolase, mitochondrial OS=Mus musculus GN=Hibch PE=1 SV=1		3.116883	1	1	1	43.01	8.06			1400000	3.126658
Q91V04	Translocating chain-associated membrane protein 1 OS=Mus musculus GN=Tram1 PE=1 SV=3		5.080214	1	3	1	43.012	9.69	900000	1300000	1200000	11.40466
P07310	Creatine kinase M-type OS=Mus musculus GN=Ckm PE=1 SV=1		20.47244	7	10	7	43.018	7.06	1300000		6300000	24.1171
Q9CXY6	Interleukin enhancer-binding factor 2 OS=Mus musculus GN=Ilf2 PE=1 SV=1		18.97436	4	8	4	43.035	5.26	1900000	2300000	2400000	25.90227
Q63844	Mitogen-activated protein kinase 3 OS=Mus musculus GN=Mapk3 PE=1 SV=5		14.73684	6	12	4	43.039	6.61	1400000	1400000	1700000	30.42719
Q76KJ5	DNA-directed RNA polymerase I subunit RPA34 OS=Mus musculus GN=Cd3eap PE=1 SV=2		4.260652	1	1	1	43.055	9.61		220000		2.336489
Q62465	Synaptic vesicle membrane protein VAT-1 homolog OS=Mus musculus GN=Vat1 PE=1 SV=3		9.852217	3	7	3	43.069	6.37	3300000	3000000	2800000	23.26929
Q91W92	Cdc42 effector protein 1 OS=Mus musculus GN=Cdc42ep1 PE=1 SV=1		3.91198	1	2	1	43.069	7.21	970000	830000		5.366867
Q8K0G5	Protein TSSC1 OS=Mus musculus GN=Eipr1 PE=1 SV=2		4.92228	1	1	1	43.1	5.14		480000		3.449553
Q8K2C9	Very-long-chain (3R)-3-hydroxyacyl-CoA dehydratase 3 OS=Mus musculus GN=Hacd3 PE=1 SV=2		8.563536	3	8	3	43.103	9.13	5700000	1800000	5200000	22.73722
P02340	Cellular tumor antigen p53 OS=Mus musculus GN=Tp53 PE=1 SV=3		3.359173	1	3	1	43.127	7.23	1600000	180000	1600000	8.514143
Q7TNV0	Protein DEK OS=Mus musculus GN=Dek PE=1 SV=1		17.63158	6	21	6	43.132	6.86	16000000	16000000	15000000	60.51596
Q924Z5	Translocating chain-associated membrane protein 2 OS=Mus musculus GN=Tram2 PE=1 SV=1		2.702703	1	1	1	43.155	9.39			1300000	2.126964
Q9DBC7	cAMP-dependent protein kinase type I-alpha regulatory subunit OS=Mus musculus GN=Prkar1a PE=1 SV=3		17.5853	5	10	4	43.158	5.35	6800000	6300000	3700000	27.38286
Q6NTA4	Ras-related GTP-binding protein B OS=Mus musculus GN=Rragb PE=1 SV=1		2.673797	1	2	1	43.164	6.38	2200000	1900000		4.435689
Q9CQE7	Endoplasmic reticulum-Golgi intermediate compartment protein 3 OS=Mus musculus GN=Ergic3 PE=1 SV=1		9.921671	3	3	3	43.181	6.47	1000000	770000		7.950351
P00158	Cytochrome b OS=Mus musculus GN=Mt-Cyb PE=1 SV=1		2.362205	1	1	1	43.181	7.97		590000		1.855096
Q8BP48	Methionine aminopeptidase 1 OS=Mus musculus GN=Metap1 PE=1 SV=1		2.849741	1	1	1	43.193	7.17	1000000			2.321111
P12849	cAMP-dependent protein kinase type I-beta regulatory subunit OS=Mus musculus GN=Prkar1b PE=1 SV=2		5.511811	2	4	1	43.197	5.96	1000000			9.847291
P35486	Pyruvate dehydrogenase E1 component subunit alpha, somatic form, mitochondrial OS=Mus musculus GN=Pdha1 PE=1 SV=1		18.71795	7	24	7	43.204	8.19	27000000	14000000	17000000	65.30573
Q9JJT9	Phosphorylated adapter RNA export protein OS=Mus musculus GN=Phax PE=1 SV=1		2.857143	1	2	1	43.221	5.34	1100000	1500000		5.322866

Q8VDQ8	NAD-dependent protein deacetylase sirtuin-2 OS=Mus musculus GN=Sirt2 PE=1 SV=2		9.254499	2	4	2	43.228	5.35	7200000	2200000	2100000	15.04614
Q9WUR2	Enoyl-CoA delta isomerase 2, mitochondrial OS=Mus musculus GN=Eci2 PE=1 SV=2		3.069054	1	1	1	43.24	8.92		2100000		2.40204
Q9D517	1-acyl-sn-glycerol-3-phosphate acyltransferase gamma OS=Mus musculus GN=Agpat3 PE=1 SV=2		2.393617	1	1	1	43.268	8.51			2300000	1.976639
Q80X73	Protein pelota homolog OS=Mus musculus GN=Pelo PE=1 SV=3		6.493506	2	4	2	43.322	5.99	370000	750000	570000	9.607926
Q91VL8	Telomeric repeat-binding factor 2-interacting protein 1 OS=Mus musculus GN=Terf2ip PE=1 SV=1		4.071247	1	2	1	43.326	4.81		190000	690000	4.968033
P70318	Nucleolysin TIAR OS=Mus musculus GN=Tial1 PE=1 SV=1		2.806122	1	1	1	43.361	7.99	1400000			1.964885
Q9JJX6	P2X purinoceptor 4 OS=Mus musculus GN=P2rx4 PE=1 SV=1		3.865979	1	2	1	43.41	7.99	2100000		950000	6.429245
P31938	Dual specificity mitogen-activated protein kinase kinase 1 OS=Mus musculus GN=Map2k1 PE=1 SV=2		6.615776	3	6	3	43.446	6.7	1300000	1200000	440000	15.71428
Q99N69	Leupaxin OS=Mus musculus GN=Lpxn PE=1 SV=2		6.735751	1	1	1	43.45	6.43			470000	2.240121
Q9Z2E1	Methyl-CpG-binding domain protein 2 OS=Mus musculus GN=Mbd2 PE=2 SV=2		2.415459	1	3	1	43.474	10.04	1300000	1200000	1400000	6.463597
P54728	UV excision repair protein RAD23 homolog B OS=Mus musculus GN=Rad23b PE=1 SV=2		2.163462	1	1	1	43.486	4.83		1500000		2.337149
O35465	Peptidyl-prolyl cis-trans isomerase FKBP8 OS=Mus musculus GN=Fkbp8 PE=1 SV=2		15.17413	4	13	4	43.501	5.16	5100000	4400000	4300000	43.42136
Q8BGA5	KRR1 small subunit processome component homolog OS=Mus musculus GN=Krr1 PE=2 SV=1		13.68421	4	9	4	43.511	9.79	1400000	2000000	1500000	25.72881
P62881	Guanine nucleotide-binding protein subunit beta-5 OS=Mus musculus GN=Gnb5 PE=1 SV=1		9.367089	2	2	2	43.537	6.46		1700000	1100000	6.702231
Q3THS6	S-adenosylmethionine synthase isoform type-2 OS=Mus musculus GN=Mat2a PE=1 SV=2		12.91139	3	5	3	43.661	6.48	5800000	3300000	3200000	17.35805
P50580	Proliferation-associated protein 2G4 OS=Mus musculus GN=Pa2g4 PE=1 SV=3		39.08629	13	48	13	43.671	6.86	31000000	32000000	27000000	137.896
Q91XD6	Vacuolar protein-sorting-associated protein 36 OS=Mus musculus GN=Vps36 PE=1 SV=1		8.031088	3	8	3	43.708	7.15	1500000	1300000	1300000	20.75456
Q08288	Cell growth-regulating nucleolar protein OS=Mus musculus GN=Lyar PE=1 SV=2		21.13402	5	11	5	43.709	9.5	3800000	3300000	1200000	36.3269
Q9ER38	Torsin-3A OS=Mus musculus GN=Tor3a PE=1 SV=2		11.16883	4	8	4	43.785	6.28	1600000	390000	1400000	21.10971
P11438	Lysosome-associated membrane glycoprotein 1 OS=Mus musculus GN=Lamp1 PE=1 SV=2		8.374384	3	12	3	43.837	8.4	25000000	23000000	15000000	39.92233
A2RTL5	Arginine/serine-rich coiled-coil protein 2 OS=Mus musculus GN=Rsrc2 PE=2 SV=1		2.659574	1	1	1	43.85	11.46			760000	2.283496
Q9Z1G3	V-type proton ATPase subunit C 1 OS=Mus musculus GN=Atp6v1c1 PE=1 SV=4		18.06283	7	17	7	43.86	7.46	5100000	4700000	3900000	47.12652
Q9CYI4	Putative RNA-binding protein Luc7-like 1 OS=Mus musculus GN=Luc7l PE=1 SV=2		16.17251	5	12	2	43.907	9.88	2900000	1000000	1000000	37.05273
Q921H8	3-ketoacyl-CoA thiolase A, peroxisomal OS=Mus musculus GN=Acaa1a PE=1 SV=1		8.962264	2	5	2	43.926	8.44	2600000	890000	1900000	16.33055
O54724	Polymerase I and transcript release factor OS=Mus musculus GN=Ptrf PE=1 SV=1		20.15306	5	13	5	43.927	5.52	3100000	1900000	3000000	45.3635
Q80YQ8	Protein RMD5 homolog A OS=Mus musculus GN=Rmnd5a PE=1 SV=2		2.813299	1	1	1	43.964	6.06			500000	2.287555
P27601	Guanine nucleotide-binding protein subunit alpha-13 OS=Mus musculus GN=Gna13 PE=1 SV=1		17.77188	6	19	5	44.027	8.21	4900000	4300000	3700000	41.68381
P47809	Dual specificity mitogen-activated protein kinase kinase 4 OS=Mus musculus GN=Map2k4 PE=1 SV=2		3.02267	1	1	1	44.085	8.07		670000		2.085501
Q99KJ8	Dynactin subunit 2 OS=Mus musculus GN=Dctn2 PE=1 SV=3		22.88557	6	14	6	44.09	5.26	3800000	4000000	2900000	42.7461
Q99K70	Ras-related GTP-binding protein C OS=Mus musculus GN=Rragc PE=1 SV=1		11.80905	4	9	4	44.093	5.1	2600000	3000000	1900000	26.8125
Q61187	Tumor susceptibility gene 101 protein OS=Mus musculus GN=Tsg101 PE=1 SV=2		14.32225	4	6	4	44.096	6.71	2100000	2200000	1800000	16.90914
Q9Z0H3	SWI/SNF-related matrix-associated actin-dependent regulator of chromatin subfamily B member 1 OS=Mus musculus GN=Smarcb		11.16883	3	12	3	44.113	6.23	2600000	2200000	2500000	40.645
P62334	26S protease regulatory subunit 10B OS=Mus musculus GN=Psmc6 PE=1 SV=1		21.33676	7	18	7	44.145	7.49	5900000	4900000	4100000	48.51243
Q61103	Zinc finger protein ubi-d4 OS=Mus musculus GN=Dpf2 PE=1 SV=1		9.71867	2	3	2	44.201	6.47		2900000	1800000	8.810675
P01867	Ig gamma-2B chain C region OS=Mus musculus GN=Igh-3 PE=1 SV=3		8.168317	2	2	2	44.231	6.52		3700000		5.678185
Q91XA2	Golgi membrane protein 1 OS=Mus musculus GN=Golm1 PE=1 SV=2		3.816794	1	2	1	44.299	5.27		920000	620000	6.224717
Q9QY24	Z-DNA-binding protein 1 OS=Mus musculus GN=Zbp1 PE=1 SV=1		19.22141	4	9	4	44.304	5.8	810000	900000	520000	25.29016
P70429	Ena/VASP-like protein OS=Mus musculus GN=Evl PE=1 SV=2		14.49275	5	17	5	44.31	8.85	2900000	3400000	2500000	61.13819
Q8QZY9	Splicing factor 3B subunit 4 OS=Mus musculus GN=Sf3b4 PE=1 SV=1		3.301887	1	3	1	44.327	8.56	9400000	8400000	8600000	10.99886
P97300	Neuroplastin OS=Mus musculus GN=Nptn PE=1 SV=3		10.83123	4	11	4	44.345	7.74	15000000	8000000	8100000	29.25286
Q8BIZ6	Smad nuclear-interacting protein 1 OS=Mus musculus GN=Snip1 PE=1 SV=1		3.133159	1	1	1	44.389	9.89				1.973805
Q9CYI0	Protein Njmu-R1 OS=Mus musculus PE=1 SV=2		3.562341	1	1	1	44.394	5.05	320000			2.424721
Q9DBY5	Chromobox protein homolog 6 OS=Mus musculus GN=Cbx6 PE=1 SV=2		4.589372	1	2	1	44.432	9.96	300000	190000		6.070963
Q8R3V5	Endophilin-B2 OS=Mus musculus GN=Sh3glb2 PE=1 SV=2		5.25	2	6	1	44.476	5.82	3500000		1500000	15.27497
Q921F2	TAR DNA-binding protein 43 OS=Mus musculus GN=Tardbp PE=1 SV=1		23.18841	10	30	10	44.519	6.7	20000000	19000000	19000000	89.52348
P09411	Phosphoglycerate kinase 1 OS=Mus musculus GN=Pgk1 PE=1 SV=4		57.07434	17	41	17	44.522	7.9	17000000	14000000	12000000	134.3919
Q9D646	Keratin, type I cuticular Ha4 OS=Mus musculus GN=Krt34 PE=2 SV=1	x	38.52041	17	46	1	44.531	4.79	4000000	7400000	12000000	117.4456
A2ADY9	Protein DDI1 homolog 2 OS=Mus musculus GN=Ddi2 PE=1 SV=1		2.255639	1	1	1	44.562	5.05	1200000			2.574224
Q61081	Hsp90 co-chaperone Cdc37 OS=Mus musculus GN=Cdc37 PE=1 SV=1		24.53826	7	17	7	44.565	5.34	13000000	13000000	11000000	52.514
Q09014	Neutrophil cytosol factor 1 OS=Mus musculus GN=Ncf1 PE=1 SV=3		7.179487	2	6	2	44.639	9.01		1300000	1500000	19.10521
Q9WV54	Acid ceramidase OS=Mus musculus GN=Asah1 PE=1 SV=1		9.390863	4	6	4	44.641	8.46	3000000	3000000	5200000	14.84342
Q9Z0W1	Tumor necrosis factor receptor superfamily member 16 OS=Mus musculus GN=Ngfr PE=1 SV=1		16.30695	3	6	3	44.657	4.64	1100000	1000000	710000	15.71994
Q9CY58	Plasminogen activator inhibitor 1 RNA-binding protein OS=Mus musculus GN=Serbp1 PE=1 SV=2		34.88943	11	39	11	44.687	8.54	9500000	14000000	8600000	119.3594

Q00899	Transcriptional repressor protein YY1 OS=Mus musculus GN=Yy1 PE=1 SV=1		6.521739	2	5	2	44.689	6.29	2500000	2600000	2000000	18.63159
Q8K339	DNA/RNA-binding protein KIN17 OS=Mus musculus GN=Kin PE=1 SV=1		6.393862	2	3	2	44.694	9.04	1400000		910000	9.283685
Q9CZ30	Obg-like ATPase 1 OS=Mus musculus GN=Ola1 PE=1 SV=1		8.838384	4	7	4	44.701	7.81	1700000	2200000	2200000	14.908
P61161	Actin-related protein 2 OS=Mus musculus GN=Actr2 PE=1 SV=1		26.14213	9	30	9	44.732	6.74	32000000	19000000	23000000	85.95263
Q9DBS1	Transmembrane protein 43 OS=Mus musculus GN=Tmem43 PE=1 SV=1		5.5	2	5	2	44.755	7.36	5600000	5900000	4900000	12.10658
Q8VE47	Ubiquitin-like modifier-activating enzyme 5 OS=Mus musculus GN=Uba5 PE=1 SV=2		5.955335	2	3	2	44.761	4.96	980000	670000	400000	6.525517
Q8QZT1	Acetyl-CoA acetyltransferase, mitochondrial OS=Mus musculus GN=Acat1 PE=1 SV=1		21.46226	7	23	7	44.787	8.51	12000000	9600000	7100000	82.33775
P63037	DnaJ homolog subfamily A member 1 OS=Mus musculus GN=Dnaja1 PE=1 SV=1		24.93703	6	18	6	44.839	7.08	12000000	10000000	9200000	61.75827
P26151	High affinity immunoglobulin gamma Fc receptor I OS=Mus musculus GN=Fcgr1 PE=1 SV=1		4.455446	1	2	1	44.86	6.09	840000		580000	5.433373
Q07417	Short-chain specific acyl-CoA dehydrogenase, mitochondrial OS=Mus musculus GN=Acads PE=1 SV=2		11.40777	3	5	3	44.861	8.47	2300000	1800000	2100000	16.09083
Q9CXY9	GPI-anchor transamidase OS=Mus musculus GN=Pigk PE=1 SV=2		2.78481	1	2	1	44.867	6.54	810000	520000		4.34373
Q9D7N3	28S ribosomal protein S9, mitochondrial OS=Mus musculus GN=Mrps9 PE=1 SV=3		2.820513	1	1	1	44.901	8.81	1700000			3.21515
Q8VE37	Regulator of chromosome condensation OS=Mus musculus GN=Rcc1 PE=1 SV=1		18.52732	6	14	6	44.903	8.1	4800000	5400000	4900000	36.2954
P18242	Cathepsin D OS=Mus musculus GN=Ctsd PE=1 SV=1		15.60976	5	15	5	44.925	7.15	9300000	1500000	6100000	43.04566
Q3THG9	Alanyl-tRNA editing protein Aarsd1 OS=Mus musculus GN=Aarsd1 PE=1 SV=2		3.15534	1	3	1	44.943	6.42	2700000	1700000	1600000	8.849759
Q9CY18	Sorting nexin-7 OS=Mus musculus GN=Snx7 PE=1 SV=1		3.100775	1	3	1	44.971	5.07	710000	750000	520000	8.058148
Q924Z4	Ceramide synthase 2 OS=Mus musculus GN=Cers2 PE=1 SV=1		2.368421	1	3	1	44.995	8.75	11000000	9500000	10000000	7.96061
Q8K2M0	39S ribosomal protein L38, mitochondrial OS=Mus musculus GN=Mrpl38 PE=1 SV=2		9.473684	2	2	2	45.002	8.1	190000			5.484147
Q8BUB4	WD repeat and FYVE domain-containing protein 2 OS=Mus musculus GN=Wdfy2 PE=1 SV=2		4	1	1	1	45.065	6.81			620000	2.744879
Q3URS9	Coiled-coil domain-containing protein 51 OS=Mus musculus GN=Ccdc51 PE=1 SV=1		2.70936	1	1	1	45.104	8.09	440000			3.479792
Q60737	Casein kinase II subunit alpha OS=Mus musculus GN=Csnk2a1 PE=1 SV=2		18.92583	6	13	6	45.105	7.74	6800000	3100000	4100000	34.96343
Q8CGE8	Interferon-activable protein 205-A OS=Mus musculus GN=Ifi205a PE=2 SV=1		19.30693	6	14	4	45.148	8.48	4100000	2700000	3200000	38.16839
Q80TL4	PHD finger protein 24 OS=Mus musculus GN=Phf24 PE=1 SV=2		13	4	10	4	45.194	5.77	1900000	2100000	2300000	32.65655
Q99LR1	Monoacylglycerol lipase ABHD12 OS=Mus musculus GN=Abhd12 PE=1 SV=2		19.34673	6	12	6	45.241	8.72	2400000	2100000	770000	41.16998
Q9EPJ9	ADP-ribosylation factor GTPase-activating protein 1 OS=Mus musculus GN=Arfgap1 PE=1 SV=2		29.4686	7	18	7	45.26	5.57	3800000	3300000	2600000	60.15283
Q8JZX4	Splicing factor 45 OS=Mus musculus GN=Rbm17 PE=1 SV=1		20.74074	7	17	6	45.276	5.82	3700000	3700000	3200000	44.23628
O89112	LanC-like protein 1 OS=Mus musculus GN=Lancl1 PE=1 SV=1		5.764411	2	4	2	45.312	7.77	2600000	4000000	2900000	11.09117
P28656	Nucleosome assembly protein 1-like 1 OS=Mus musculus GN=Nap1l1 PE=1 SV=2		11.50895	3	8	2	45.317	4.46	3800000	2000000	8000000	25.88147
Q9JL35	High mobility group nucleosome-binding domain-containing protein 5 OS=Mus musculus GN=Hmgn5 PE=1 SV=2		7.635468	2	6	2	45.317	4.37	2000000	1300000	790000	18.27349
Q80VD1	Protein FAM98B OS=Mus musculus GN=Fam98b PE=1 SV=1		8.857809	3	6	3	45.321	8.5	2800000	3100000	1400000	18.99146
P12367	cAMP-dependent protein kinase type II-alpha regulatory subunit OS=Mus musculus GN=Prkar2a PE=1 SV=2		4.987531	1	2	1	45.361	4.89	5000000		4900000	8.352031
Q99JI4	26S proteasome non-ATPase regulatory subunit 6 OS=Mus musculus GN=Psmd6 PE=1 SV=1		17.99486	8	19	8	45.507	5.52	6000000	5900000	3900000	49.74124
Q8BU14	Translocation protein SEC62 OS=Mus musculus GN=Sec62 PE=1 SV=1		11.55779	6	11	6	45.552	7.31	1700000	2500000	1900000	29.6551
P62196	26S protease regulatory subunit 8 OS=Mus musculus GN=Psmc5 PE=1 SV=1		25.61576	8	20	8	45.597	7.55	6000000	5600000	5700000	63.33074
Q8VCF1	Soluble calcium-activated nucleotidase 1 OS=Mus musculus GN=Cant1 PE=2 SV=1		3.970223	1	1	1	45.624	6.77			570000	2.517747
P17047	Lysosome-associated membrane glycoprotein 2 OS=Mus musculus GN=Lamp2 PE=1 SV=2		12.04819	5	14	5	45.652	7.39	8000000	8800000	7700000	32.61994
Q8VE62	Polyadenylate-binding protein-interacting protein 1 OS=Mus musculus GN=Paip1 PE=1 SV=1		3	1	1	1	45.673	4.55		820000		3.156974
Q91XD7	Cysteine-rich with EGF-like domain protein 1 OS=Mus musculus GN=Creld1 PE=1 SV=1		7.619048	2	4	2	45.687	5.02	1500000	1100000	1400000	14.30878
Q9Z2X1	Heterogeneous nuclear ribonucleoprotein F OS=Mus musculus GN=Hnrnpf PE=1 SV=3		31.08434	10	29	8	45.701	5.49	21000000	20000000	29000000	93.54511
Q8BLF1	Neutral cholesterol ester hydrolase 1 OS=Mus musculus GN=Nceh1 PE=1 SV=1		2.941176	1	3	1	45.711	7.05	7900000	6900000	6800000	10.25583
Q9QYJ0	DnaJ homolog subfamily A member 2 OS=Mus musculus GN=Dnaja2 PE=1 SV=1		29.36893	9	20	9	45.717	6.48	8600000	7300000	7300000	57.74461
Q8BK62	Olfactomedin-like protein 3 OS=Mus musculus GN=Olfml3 PE=2 SV=2		8.128079	2	4	2	45.717	6.23	570000	590000	570000	12.24008
O35984	Pre-B-cell leukemia transcription factor 2 OS=Mus musculus GN=Pbx2 PE=1 SV=1		3.488372	1	1	1	45.781	7.56	410000			2.772403
Q00898	Alpha-1-antitrypsin 1-5 OS=Mus musculus GN=Serpina1e PE=1 SV=1		6.53753	2	6	1	45.862	5.73	820000	570000	600000	17.99116
Q99KU0	Vacuole membrane protein 1 OS=Mus musculus GN=Vmp1 PE=1 SV=2		3.940887	1	1	1	45.931	6.95		3700000		2.904893
Q9JIY0	Pleckstrin homology domain-containing family O member 1 OS=Mus musculus GN=Plekho1 PE=1 SV=1		3.186275	1	2	1	45.969	8.97		1500000	1200000	5.274388
P07758	Alpha-1-antitrypsin 1-1 OS=Mus musculus GN=Serpina1a PE=1 SV=4		6.53753	2	6	1	45.974	5.72	5400000	3700000	3200000	20.67957
P27659	60S ribosomal protein L3 OS=Mus musculus GN=Rpl3 PE=1 SV=3		28.53598	11	38	11	46.081	10.21	42000000	45000000	38000000	111.3178
P70295	Ancient ubiquitous protein 1 OS=Mus musculus GN=Aup1 PE=1 SV=1		2.926829	1	2	1	46.092	8.4	3100000	2500000		6.847631
Q8K0Y2	Keratin, type I cuticular Ha3-I OS=Mus musculus GN=Krt33a PE=1 SV=1		49.75248	22	54	5	46.107	4.83	4100000	7900000	11000000	145.5233
P60843	Eukaryotic initiation factor 4A-I OS=Mus musculus GN=Eif4a1 PE=1 SV=1		28.81773	12	55	8	46.125	5.48	26000000	24000000	21000000	156.0924
P31324	cAMP-dependent protein kinase type II-beta regulatory subunit OS=Mus musculus GN=Prkar2b PE=1 SV=3		4.807692	1	3	1	46.138	4.98	6000000	2800000	3200000	15.504
Q9CYD3	Cartilage-associated protein OS=Mus musculus GN=Crtap PE=1 SV=3		4	1	1	1	46.14	5.71				2.715906

Q9QZB7	Actin-related protein 10 OS=Mus musculus GN=Actr10 PE=1 SV=2		7.913669	2	3	2	46.178	7.61		400000	410000	8.61925
Q3USH1	Protein FAM196A OS=Mus musculus GN=Fam196a PE=1 SV=1		4.50237	1	1	1	46.193	7.14				2.591757
Q922H4	Mannose-1-phosphate guanyltransferase alpha OS=Mus musculus GN=Gmppa PE=1 SV=1		3.333333	1	1	1	46.215	7.62			900000	2.271612
P05201	Aspartate aminotransferase, cytoplasmic OS=Mus musculus GN=Got1 PE=1 SV=3		8.232446	3	8	3	46.219	7.14	3900000	3400000	2900000	21.17346
O88544	COP9 signalosome complex subunit 4 OS=Mus musculus GN=Cops4 PE=1 SV=1		7.142857	2	6	2	46.256	5.83	1800000	1700000	1500000	18.2662
Q3UPH1	Protein PRRC1 OS=Mus musculus GN=Prrc1 PE=1 SV=1		2.934537	1	1	1	46.268	5.95	1900000			3.050979
O35368	Interferon-activable protein 203 OS=Mus musculus GN=Ifi203 PE=1 SV=1		13.72549	4	10	3	46.27	9.35	1500000	970000	630000	29.73907
Q91YI4	Beta-arrestin-2 OS=Mus musculus GN=Arrb2 PE=1 SV=1		2.926829	1	1	1	46.285	7.66				2.942062
Q8R0A7	Uncharacterized protein KIAA0513 OS=Mus musculus GN=Kiaa0513 PE=1 SV=1		3.194103	1	2	1	46.289	5.02	2400000	1400000		6.31752
Q9JHI5	Isovaleryl-CoA dehydrogenase, mitochondrial OS=Mus musculus GN=Ivd PE=1 SV=1		8.254717	3	5	3	46.296	8.29	2800000	2200000	2000000	15.34229
Q9ER73	Elongator complex protein 4 OS=Mus musculus GN=Elp4 PE=1 SV=2		3.554502	1	1	1	46.296	8.78			510000	2.116771
Q9CZC8	Secernin-1 OS=Mus musculus GN=Scrn1 PE=1 SV=1		4.589372	1	3	1	46.297	4.79	2800000	1700000	2300000	9.696533
Q640R3	Hepatocyte cell adhesion molecule OS=Mus musculus GN=Hepacam PE=1 SV=2		4.545455	1	3	1	46.338	9.42	2000000	990000		7.300106
P10630	Eukaryotic initiation factor 4A-II OS=Mus musculus GN=Eif4a2 PE=1 SV=2		16.70762	6	28	2	46.373	5.48	3000000	3300000	2600000	89.01018
Q91W90	Thioredoxin domain-containing protein 5 OS=Mus musculus GN=Txndc5 PE=1 SV=2		20.38369	6	11	6	46.386	5.78	4300000	5400000	5400000	32.87334
Q9D7N9	Adipocyte plasma membrane-associated protein OS=Mus musculus GN=Apmap PE=1 SV=1		14.6988	5	12	5	46.405	6.32	8300000	5900000	7200000	34.46293
Q0VGB7	Serine/threonine-protein phosphatase 4 regulatory subunit 2 OS=Mus musculus GN=Ppp4r2 PE=1 SV=1		3.117506	1	2	1	46.45	4.56				5.273862
P45952	Medium-chain specific acyl-CoA dehydrogenase, mitochondrial OS=Mus musculus GN=Acadm PE=1 SV=1		8.07601	3	6	3	46.452	8.37	2600000	3100000	2900000	16.02146
Q91WL8	WW domain-containing oxidoreductase OS=Mus musculus GN=Wwox PE=1 SV=1		3.381643	1	1	1	46.483	7.02	400000			2.19035
Q8R570	Synaptosomal-associated protein 47 OS=Mus musculus GN=Snap47 PE=1 SV=1		12.83293	4	8	4	46.495	5.76	1700000	2200000	1100000	22.65805
P19324	Serpin H1 OS=Mus musculus GN=Serpinh1 PE=1 SV=3		23.98082	8	30	8	46.504	8.82	22000000	17000000	22000000	83.71676
Q3U1F9	Phosphoprotein associated with glycosphingolipid-enriched microdomains 1 OS=Mus musculus GN=Pag1 PE=1 SV=2		6.759907	1	2	1	46.52	4.81	1300000			6.805506
Q60766	Immunity-related GTPase family M protein 1 OS=Mus musculus GN=Irgm1 PE=1 SV=1		22.24939	10	25	10	46.522	8.28	47000000	49000000	40000000	74.43967
Q9ERI5	Bifunctional arginine demethylase and lysyl-hydroxylase JMJD6 OS=Mus musculus GN=Jmjd6 PE=1 SV=2		2.233251	1	2	1	46.538	8.9	850000		680000	4.202381
Q8VCS3	Glycosaminoglycan xylosylkinase OS=Mus musculus GN=Fam20b PE=1 SV=1		7.334963	2	5	2	46.551	6.95	2500000	1800000	1600000	15.47602
Q7TNC4	Putative RNA-binding protein Luc7-like 2 OS=Mus musculus GN=Luc7l2 PE=1 SV=1		24.23469	8	20	5	46.555	10.1	4900000	7100000	7500000	59.48552
P16460	Argininosuccinate synthase OS=Mus musculus GN=Ass1 PE=1 SV=1		17.71845	9	19	9	46.555	8.22	14000000	13000000	13000000	46.99968
O54941	SWI/SNF-related matrix-associated actin-dependent regulator of chromatin subfamily E member 1 OS=Mus musculus GN=Smarce1		10.7056	4	6	3	46.61	4.88	940000	1800000	1300000	15.16876
Q6P8X1	Sorting nexin-6 OS=Mus musculus GN=Snx6 PE=1 SV=2		20.68966	9	18	8	46.62	6.16	3500000	4400000	5400000	40.98576
P63005	Platelet-activating factor acetylhydrolase IB subunit alpha OS=Mus musculus GN=Pafah1b1 PE=1 SV=2		24.63415	8	26	7	46.64	7.37	13000000	11000000	10000000	76.4938
O88844	Isocitrate dehydrogenase [NADP] cytoplasmic OS=Mus musculus GN=Idh1 PE=1 SV=2		8.695652	3	4	3	46.644	7.17	1500000	1500000		10.17904
Q9WV60	Glycogen synthase kinase-3 beta OS=Mus musculus GN=Gsk3b PE=1 SV=2		13.33333	3	5	2	46.681	8.78	2200000	3200000	1300000	10.48802
Q91WP6	Serine protease inhibitor A3N OS=Mus musculus GN=Serpina3n PE=1 SV=1		10.28708	3	8	2	46.688	5.82	1200000	1900000	1300000	24.87414
P59017	Bcl-2-like protein 13 OS=Mus musculus GN=Bcl2l13 PE=1 SV=2		5.990783	2	3	2	46.691	4.59	2800000	3000000		8.913993
Q9D8U8	Sorting nexin-5 OS=Mus musculus GN=Snx5 PE=1 SV=1		20.79208	8	15	7	46.768	6.62	9700000	7600000	4400000	42.1659
Q91VC3	Eukaryotic initiation factor 4A-III OS=Mus musculus GN=Eif4a3 PE=1 SV=3		18.24818	8	26	8	46.81	6.73	11000000	9400000	12000000	75.48108
Q9Z2I8	Succinate--CoA ligase [GDP-forming] subunit beta, mitochondrial OS=Mus musculus GN=Suclg2 PE=1 SV=3		9.930716	3	8	3	46.811	7.02	2400000	2100000	1600000	27.17051
Q9D1Q6	Endoplasmic reticulum resident protein 44 OS=Mus musculus GN=Erp44 PE=1 SV=1		25.61576	9	31	8	46.823	5.27	7000000	5800000	6300000	97.36986
P07759	Serine protease inhibitor A3K OS=Mus musculus GN=Serpina3k PE=1 SV=2		11.24402	4	10	3	46.85	5.16	920000	2000000	2700000	28.63136
Q8BV49	Pyrin and HIN domain-containing protein 1 OS=Mus musculus GN=Pyhin1 PE=1 SV=1		20.2381	7	19	7	46.857	9.45	7200000	7000000	6000000	58.12559
Q8BWL5	RNA-binding motif, single-stranded-interacting protein 3 OS=Mus musculus GN=Rbms3 PE=2 SV=2		3.480278	1	1	1	46.897	7.99		2200000		2.98375
Q3TCJ1	BRISC complex subunit Abro1 OS=Mus musculus GN=Fam175b PE=1 SV=1		3.13253	1	3	1	46.914	6.18	1700000	2000000	270000	5.853742
Q9ESW4	Acylglycerol kinase, mitochondrial OS=Mus musculus GN=Agk PE=1 SV=1		8.551069	3	6	3	46.946	8.4	1600000	1500000	1600000	14.9464
P21183	Interleukin-5 receptor subunit alpha OS=Mus musculus GN=Il5ra PE=2 SV=1		3.13253	1	2	1	46.959	7.84				4.86632
P30275	Creatine kinase U-type, mitochondrial OS=Mus musculus GN=Ckmt1 PE=1 SV=1		12.67943	4	11	4	46.974	8.16	5600000	3200000	3400000	36.00767
Q9DCL9	Multifunctional protein ADE2 OS=Mus musculus GN=Paics PE=1 SV=4		27.05882	10	36	10	46.976	7.23	16000000	18000000	12000000	111.4568
Q8BSE0	Regulator of microtubule dynamics protein 2 OS=Mus musculus GN=Rmdn2 PE=1 SV=1		6.585366	2	5	2	46.986	7.56	980000	970000	840000	12.1047
Q60634	Flotillin-2 OS=Mus musculus GN=Flot2 PE=1 SV=2		5.841121	2	4	2	47.009	5.2	1900000	2300000	1700000	11.602
Q61765	Keratin, type I cuticular Ha1 OS=Mus musculus GN=Krt31 PE=1 SV=2	x	43.50962	20	55	2	47.087	4.89	1100000	2100000	1900000	144.3936
Q62443	Neuronal pentraxin-1 OS=Mus musculus GN=Nptx1 PE=1 SV=1		9.490741	2	5	2	47.088	6.44	1900000	810000	1100000	13.35677
P16330	2',3'-cyclic-nucleotide 3'-phosphodiesterase OS=Mus musculus GN=Cnp PE=1 SV=3		36.66667	17	54	17	47.094	8.97	60000000	37000000	35000000	171.9367
P17182	Alpha-enolase OS=Mus musculus GN=Eno1 PE=1 SV=3		23.04147	9	30	8	47.111	6.8	15000000	12000000	11000000	84.80939
Q9D8E6	60S ribosomal protein L4 OS=Mus musculus GN=Rpl4 PE=1 SV=3		27.20764	13	50	13	47.124	11	60000000	49000000	64000000	144.7724

Q4VBE8	WD repeat-containing protein 18 OS=Mus musculus GN=Wdr18 PE=1 SV=1		7.424594	3	6	3	47.181	6.89	1600000	1100000	990000	16.3474
Q64345	Interferon-induced protein with tetratricopeptide repeats 3 OS=Mus musculus GN=Ifit3 PE=1 SV=1		40.44665	14	38	14	47.192	5.64	15000000	14000000	9900000	124.0909
Q9D0F4	NF-kappa-B-activating protein OS=Mus musculus GN=Nkap PE=2 SV=1		3.373494	1	1	1	47.199	10.13			2000000	2.453612
Q9DBG5	Perilipin-3 OS=Mus musculus GN=Plin3 PE=1 SV=1		5.034325	2	2	2	47.233	5.62	1400000	1800000		4.95992
P17183	Gamma-enolase OS=Mus musculus GN=Eno2 PE=1 SV=2		8.525346	3	9	2	47.267	5.11	1900000	2200000	2000000	24.10876
Q80XI4	Phosphatidylinositol 5-phosphate 4-kinase type-2 beta OS=Mus musculus GN=Pip4k2b PE=1 SV=1		7.932692	3	9	3	47.289	7.33	4100000	3000000	2900000	26.93236
P06336	Ig epsilon chain C region OS=Mus musculus PE=4 SV=2		7.125891	2	5	2	47.291	7.44	470000	1000000	1000000	12.67805
Q9JMK2	Casein kinase I isoform epsilon OS=Mus musculus GN=Csnk1e PE=1 SV=2		2.644231	1	1	1	47.292	9.66			1300000	0
Q8BP71	RNA binding protein fox-1 homolog 2 OS=Mus musculus GN=Rbfox2 PE=1 SV=2		4.899777	1	2	1	47.301	6.55	1100000			6.75958
Q99JY9	Actin-related protein 3 OS=Mus musculus GN=Actr3 PE=1 SV=3		31.81818	10	37	10	47.327	5.88	33000000	30000000	27000000	115.6654
Q06180	Tyrosine-protein phosphatase non-receptor type 2 OS=Mus musculus GN=Ptpn2 PE=1 SV=2		11.08374	3	5	3	47.33	8.37	2900000	1700000	2500000	15.86023
Q91ZN5	Adenosine 3'-phospho 5'-phosphosulfate transporter 1 OS=Mus musculus GN=Slc35b2 PE=1 SV=1		2.320186	1	2	1	47.339	9.29		2500000	3600000	5.815048
Q9ERS5	Pleckstrin homology domain-containing family A member 2 OS=Mus musculus GN=Plekha2 PE=1 SV=1		2.588235	1	1	1	47.35	8.47	490000			2.741481
Q9DBE8	Alpha-1,3/1,6-mannosyltransferase ALG2 OS=Mus musculus GN=Alg2 PE=1 SV=2		6.024096	2	4	2	47.374	7.97	510000	2000000	3100000	10.51969
P54775	26S protease regulatory subunit 6B OS=Mus musculus GN=Psmc4 PE=1 SV=2		19.37799	8	14	8	47.379	5.21	4000000	2600000	3800000	36.86314
P05202	Aspartate aminotransferase, mitochondrial OS=Mus musculus GN=Got2 PE=1 SV=1		21.62791	10	30	10	47.381	9	29000000	23000000	27000000	87.21028
P46096	Synaptotagmin-1 OS=Mus musculus GN=Syt1 PE=1 SV=1		26.60333	10	24	10	47.388	8.53	20000000	9500000	6900000	64.3194
Q8BG32	26S proteasome non-ATPase regulatory subunit 11 OS=Mus musculus GN=Psmd11 PE=1 SV=3		21.09005	9	18	9	47.407	6.48	5800000	5800000	3900000	56.96076
Q9Z2N8	Actin-like protein 6A OS=Mus musculus GN=Actl6a PE=1 SV=2		9.324009	3	5	3	47.417	5.6	1200000		980000	15.19721
Q7TQ95	Protein lunapark OS=Mus musculus GN=Lnp PE=1 SV=1		16.94118	5	11	5	47.47	5.27	2400000	1900000	1600000	30.13588
O08917	Flotillin-1 OS=Mus musculus GN=Flot1 PE=1 SV=1		6.074766	2	4	2	47.484	7.15	1000000	1400000	1800000	11.32304
Q9CPV7	Palmitoyltransferase ZDHHC6 OS=Mus musculus GN=Zdhhc6 PE=2 SV=1		2.905569	1	1	1	47.496	8.54	1100000			2.518075
Q8BLQ9	Cell adhesion molecule 2 OS=Mus musculus GN=Cadm2 PE=1 SV=2		10.34483	3	10	3	47.529	5.41	5300000	2800000	3000000	40.33079
Q9QZ85	Interferon-inducible GTPase 1 OS=Mus musculus GN=Iigp1 PE=1 SV=2		51.81598	17	64	17	47.542	6.33	37000000	37000000	29000000	218.0612
P97814	Proline-serine-threonine phosphatase-interacting protein 1 OS=Mus musculus GN=Pstpip1 PE=1 SV=1		2.168675	1	3	1	47.561	5.53	1000000	970000	670000	7.014765
Q60972	Histone-binding protein RBBP4 OS=Mus musculus GN=Rbbp4 PE=1 SV=5		26.82353	9	28	5	47.626	4.89	15000000	9400000	14000000	90.79294
P50247	Adenosylhomocysteinase OS=Mus musculus GN=Ahcy PE=1 SV=3		14.12037	5	13	4	47.657	6.54	3300000	4200000	3800000	34.5557
O88712	C-terminal-binding protein 1 OS=Mus musculus GN=Ctbp1 PE=1 SV=2		15.19274	6	11	5	47.715	6.77	2900000	3000000	3000000	27.91358
P32067	Lupus La protein homolog OS=Mus musculus GN=Ssb PE=1 SV=1		24.09639	10	29	10	47.727	9.77	21000000	20000000	19000000	93.30378
Q8BK72	28S ribosomal protein S27, mitochondrial OS=Mus musculus GN=Mrps27 PE=1 SV=2		9.156627	3	6	3	47.748	5.5	1500000	1700000	1100000	16.34455
Q60973	Histone-binding protein RBBP7 OS=Mus musculus GN=Rbbp7 PE=1 SV=1		28.23529	9	26	5	47.76	5.05	7100000	8300000	6900000	83.13981
O88543	COP9 signalosome complex subunit 3 OS=Mus musculus GN=Cops3 PE=1 SV=3		3.546099	1	3	1	47.801	6.65	1200000	1600000		9.526465
O08736	Caspase-12 OS=Mus musculus GN=Casp12 PE=1 SV=1		2.625298	1	1	1	47.823	6.37			850000	2.208715
P51174	Long-chain specific acyl-CoA dehydrogenase, mitochondrial OS=Mus musculus GN=Acadl PE=1 SV=2		22.09302	7	22	7	47.877	8.31	8700000	7400000	8500000	77.94835
Q922H2	[Pyruvate dehydrogenase (acetyl-transferring)] kinase isozyme 3, mitochondrial OS=Mus musculus GN=Pdk3 PE=1 SV=1		5.542169	2	7	2	47.893	8.82	1700000	1600000	1500000	17.85952
O55040	Neuromedin-U receptor 1 OS=Mus musculus GN=Nmur1 PE=2 SV=2		3.738318	1	1	1	47.903	8.72		57000000		1.870761
Q99KH8	Serine/threonine-protein kinase 24 OS=Mus musculus GN=Stk24 PE=1 SV=1		12.06497	4	8	4	47.924	5.43	2900000	2500000	2400000	25.59879
O88522	NF-kappa-B essential modulator OS=Mus musculus GN=Ikbkg PE=1 SV=2		1.941748	1	1	1	47.942	5.85				2.086301
P47226	Testin OS=Mus musculus GN=Tes PE=1 SV=1		2.600473	1	1	1	47.951	8.31		680000		3.102876
Q8VD58	Protein EVI2B OS=Mus musculus GN=Evi2b PE=1 SV=1		3.828829	1	2	1	47.964	4.61	430000		400000	6.005379
P14211	Calreticulin OS=Mus musculus GN=Calr PE=1 SV=1		56.73077	23	126	23	47.965	4.49	1.1E+08	1.1E+08	1.1E+08	433.725
Q9CQC6	Basic leucine zipper and W2 domain-containing protein 1 OS=Mus musculus GN=Bzw1 PE=1 SV=1		16.46778	9	22	6	48.013	5.92	14000000	8400000	13000000	53.69767
Q99KK2	N-acylneuraminate cytidylyltransferase OS=Mus musculus GN=Cmas PE=1 SV=2		14.81481	5	14	5	48.028	8.1	3800000	5100000	5000000	42.68156
Q91VK1	Basic leucine zipper and W2 domain-containing protein 2 OS=Mus musculus GN=Bzw2 PE=1 SV=1		5.966587	4	11	1	48.033	6.68	2700000	3400000	3200000	30.45337
Q922R8	Protein disulfide-isomerase A6 OS=Mus musculus GN=Pdia6 PE=1 SV=3		29.31818	9	31	9	48.07	5.14	62000000	59000000	51000000	101.0461
Q04695	SWISS-PROT:Q04695 Tax_Id=9606 Gene_Symbol=KRT17 Keratin, type I cytoskeletal 17	x	45.83333	20	41	3	48.076	5.02			8300000	110.2948
Q9C075	SWISS-PROT:Q9C075 Tax_Id=9606 Gene_Symbol=KRT23 Keratin, type I cytoskeletal 23	x	6.635071	2	2	1	48.101	6.54			1900000	4.214563
Q9QWL7	SWISS-PROT:Q9QWL7 Tax_Id=10090 Gene_Symbol=Krt17 Keratin, type I cytoskeletal 17	x	40.8776	18	42	1	48.132	5.06			1800000	110.8862
Q64337	Sequestosome-1 OS=Mus musculus GN=Sqstm1 PE=1 SV=1		26.24434	6	13	6	48.132	5.21	3700000	3000000	1700000	46.44239
Q9D6K9	Ceramide synthase 5 OS=Mus musculus GN=Cers5 PE=1 SV=1		1.690821	1	1	1	48.135	8.07			1000000	2.225555
Q60780	Growth arrest-specific protein 7 OS=Mus musculus GN=Gas7 PE=1 SV=1		7.83848	3	7	3	48.143	7.83	3100000	1700000	1900000	16.36012
Q8BGA9	Mitochondrial inner membrane protein OXA1L OS=Mus musculus GN=Oxa1l PE=1 SV=1		2.309469	1	2	1	48.189	9.61		1200000	1400000	5.36963
Q9DB77	Cytochrome b-c1 complex subunit 2, mitochondrial OS=Mus musculus GN=Uqcrc2 PE=1 SV=1		31.34658	11	35	11	48.205	9.25	16000000	15000000	12000000	113.6243

Q9D964	Glycine amidinotransferase, mitochondrial OS=Mus musculus GN=Gatm PE=1 SV=1		8.510638	3	4	3	48.266	7.88	1800000	1800000	1100000	11.92948
Q3TIV5	Zinc finger CCCH domain-containing protein 15 OS=Mus musculus GN=Zc3h15 PE=1 SV=2		10.32864	5	10	5	48.297	5.3	3000000	2200000	2200000	28.72417
Q921S7	39S ribosomal protein L37, mitochondrial OS=Mus musculus GN=Mrpl37 PE=1 SV=1		4.255319	1	1	1	48.31	8.84			1100000	2.948414
P29758	Ornithine aminotransferase, mitochondrial OS=Mus musculus GN=Oat PE=1 SV=1		11.38952	3	6	3	48.324	6.62	5300000	2400000	2400000	19.86758
Q60749	KH domain-containing, RNA-binding, signal transduction-associated protein 1 OS=Mus musculus GN=Khdrbs1 PE=1 SV=2		5.191874	3	16	2	48.34	8.72	13000000	13000000	10000000	48.87837
Q99LC2	Cleavage stimulation factor subunit 1 OS=Mus musculus GN=Cstf1 PE=1 SV=1		7.888631	2	6	2	48.351	6.58	1500000	1200000	1600000	22.12265
P35585	AP-1 complex subunit mu-1 OS=Mus musculus GN=Ap1m1 PE=1 SV=3		12.52955	4	10	4	48.512	7.3	3700000	3200000	3200000	27.13226
Q99JB8	Protein kinase C and casein kinase II substrate protein 3 OS=Mus musculus GN=Pacsin3 PE=1 SV=1		3.773585	1	2	1	48.555	6.1	250000	670000		4.143542
Q60759	Glutaryl-CoA dehydrogenase, mitochondrial OS=Mus musculus GN=Gcdh PE=1 SV=2		6.392694	2	3	2	48.575	8.73		770000	1100000	8.769929
P46471	26S protease regulatory subunit 7 OS=Mus musculus GN=Psmc2 PE=1 SV=5		25.86605	10	23	10	48.617	5.95	10000000	9400000	8600000	65.0839
Q62418	Drebrin-like protein OS=Mus musculus GN=Dbnl PE=1 SV=2		27.75229	8	28	8	48.67	4.92	7500000	7400000	6000000	94.3293
Q8VD31	Tapasin-related protein OS=Mus musculus GN=Tapbpl PE=1 SV=2		4.21286	2	4	2	48.702	6.46	8400000	4800000	5600000	9.443196
Q9CZ91	Serum response factor-binding protein 1 OS=Mus musculus GN=Srfbp1 PE=1 SV=1		4.761905	1	5	1	48.716	9.54	2100000	700000	1200000	17.50549
Q9DBF7	Pre-mRNA-splicing factor CWC25 homolog OS=Mus musculus GN=Cwc25 PE=2 SV=2		2.884615	1	1	1	48.782	10.23			300000	1.910066
Q9JHI7	Exosome complex component RRP45 OS=Mus musculus GN=Exosc9 PE=1 SV=1		5.479452	2	5	2	48.906	5.11	1100000	1500000	1100000	13.91919
P56546	C-terminal-binding protein 2 OS=Mus musculus GN=Ctbp2 PE=1 SV=2		4.269663	2	2	1	48.926	6.95	1500000			4.659512
P59325	Eukaryotic translation initiation factor 5 OS=Mus musculus GN=Eif5 PE=1 SV=1		11.42191	4	8	4	48.938	5.52	4200000	3000000	3800000	21.56996
Q9D2G2	Dihydrolipoyllysine-residue succinyltransferase component of 2-oxoglutarate dehydrogenase complex, mitochondrial OS=Mus mu		7.048458	3	7	3	48.963	8.95	4100000	2300000	4100000	19.27876
O54734	Dolichyl-diphosphooligosaccharide--protein glycosyltransferase 48 kDa subunit OS=Mus musculus GN=Ddost PE=1 SV=2		12.01814	5	13	5	48.997	5.83	18000000	15000000	18000000	35.10423
Q8BWY3	Eukaryotic peptide chain release factor subunit 1 OS=Mus musculus GN=Etf1 PE=1 SV=4		12.12815	5	15	5	49	5.71	6500000	7300000	6700000	41.81423
Q9Z1N5	Spliceosome RNA helicase Ddx39b OS=Mus musculus GN=Ddx39b PE=1 SV=1		22.8972	9	31	4	49.004	5.67	5900000	8000000	9500000	85.89221
Q8VDW0	ATP-dependent RNA helicase DDX39A OS=Mus musculus GN=Ddx39a PE=1 SV=1		17.79859	8	19	3	49.036	5.68	6600000	6900000	8600000	51.13371
P62192	26S protease regulatory subunit 4 OS=Mus musculus GN=Psmc1 PE=1 SV=1		22.04545	7	21	7	49.154	6.21	4200000	4100000	2800000	66.48805
O35737	Heterogeneous nuclear ribonucleoprotein H OS=Mus musculus GN=Hnrnph1 PE=1 SV=3		32.07127	10	38	4	49.168	6.3	17000000	15000000	21000000	124.6744
Q810D6	Glutamate-rich WD repeat-containing protein 1 OS=Mus musculus GN=Grwd1 PE=1 SV=2		11.88341	3	4	3	49.193	4.81	1300000	1100000		11.96214
Q8BY71	Histone acetyltransferase type B catalytic subunit OS=Mus musculus GN=Hat1 PE=1 SV=1		3.846154	1	5	1	49.247	6.33	1300000	1100000	800000	16.22449
P70333	Heterogeneous nuclear ribonucleoprotein H2 OS=Mus musculus GN=Hnrnph2 PE=1 SV=1		22.049	7	30	2	49.248	6.3	5000000	3700000	5100000	102.3349
Q63912	Oligodendrocyte-myelin glycoprotein OS=Mus musculus GN=Omg PE=1 SV=1		4.545455	2	3	2	49.252	8.41	9000000	5700000		8.133835
P20664	DNA primase small subunit OS=Mus musculus GN=Prim1 PE=1 SV=1		2.63789	1	1	1	49.264	8.6		630000		2.39085
Q9JIY5	Serine protease HTRA2, mitochondrial OS=Mus musculus GN=Htra2 PE=1 SV=2		2.620087	1	2	1	49.318	9.6	1300000	1100000		5.41678
Q9D2V8	Major facilitator superfamily domain-containing protein 10 OS=Mus musculus GN=Mfsd10 PE=1 SV=1		5.701754	2	5	2	49.337	9.95	4200000	3500000	6900000	12.85612
O89017	Legumain OS=Mus musculus GN=Lgmn PE=1 SV=1		6.666667	2	3	2	49.341	6.39		800000	1400000	7.756741
P62484	Abl interactor 2 OS=Mus musculus GN=Abi2 PE=1 SV=1		7.847534	3	6	2	49.356	6.01	1800000	820000	720000	15.96784
Q8VCM7	Fibrinogen gamma chain OS=Mus musculus GN=Fgg PE=1 SV=1		50	16	37	16	49.36	5.86	14000000	17000000	15000000	107.283
P46467	Vacuolar protein sorting-associated protein 4B OS=Mus musculus GN=Vps4b PE=1 SV=2		1.801802	1	2	1	49.389	7.11	830000		1100000	4.533396
Q9WVJ9	EGF-containing fibulin-like extracellular matrix protein 2 OS=Mus musculus GN=Efemp2 PE=1 SV=1		12.18962	4	8	4	49.392	4.89	1400000	1700000	2000000	22.66582
Q9JIH2	Nuclear pore complex protein Nup50 OS=Mus musculus GN=Nup50 PE=1 SV=3		5.364807	1	1	1	49.455	6.24				2.675808
Q8BFR5	Elongation factor Tu, mitochondrial OS=Mus musculus GN=Tufm PE=1 SV=1		13.0531	5	13	5	49.477	7.56	6500000	4200000	4900000	35.16869
O88685	26S protease regulatory subunit 6A OS=Mus musculus GN=Psmc3 PE=1 SV=2		26.92308	8	26	8	49.518	5.19	9400000	8300000	7000000	79.62004
Q9R099	Transducin beta-like protein 2 OS=Mus musculus GN=Tbl2 PE=1 SV=2		9.276018	4	6	4	49.552	9.04	2100000	1900000	2300000	14.55165
Q9D6F9	Tubulin beta-4A chain OS=Mus musculus GN=Tubb4a PE=1 SV=3		41.21622	15	133	2	49.554	4.88	29000000	21000000	13000000	396.9235
P35821	Tyrosine-protein phosphatase non-receptor type 1 OS=Mus musculus GN=Ptpn1 PE=1 SV=2		23.14815	7	16	7	49.561	6.16	6500000	4400000	3200000	42.49456
Q9D0D5	General transcription factor IIE subunit 1 OS=Mus musculus GN=Gtf2e1 PE=1 SV=1		2.727273	1	3	1	49.562	4.86	2200000	2200000	1900000	7.707129
Q9EST1	Gasdermin-A OS=Mus musculus GN=Gsdma PE=2 SV=1		2.017937	1	1	1	49.562	5.57			2400000	1.80865
Q91ZE0	Trimethyllysine dioxygenase, mitochondrial OS=Mus musculus GN=Tmlhe PE=1 SV=2		2.375297	1	1	1	49.578	8.25		1700000		1.984209
Q9R1T4	Septin-6 OS=Mus musculus GN=Sept6 PE=1 SV=4		23.73272	8	22	3	49.588	6.43	7100000	4800000	3700000	64.61614
P84091	AP-2 complex subunit mu OS=Mus musculus GN=Ap2m1 PE=1 SV=1		16.32184	7	13	7	49.623	9.54	6600000	4200000	4000000	37.98065
P99024	Tubulin beta-5 chain OS=Mus musculus GN=Tubb5 PE=1 SV=1		50	19	160	3	49.639	4.89	75000000	32000000	32000000	494.1948
Q8C1B7	Septin-11 OS=Mus musculus GN=Sept11 PE=1 SV=4		34.57077	13	35	6	49.663	6.68	20000000	15000000	14000000	98.23488
P36552	Oxygen-dependent coproporphyrinogen-III oxidase, mitochondrial OS=Mus musculus GN=Cpox PE=1 SV=2		3.386005	1	2	1	49.683	8.53	1100000	750000		6.629893
Q9R233	Tapasin OS=Mus musculus GN=Tapbp PE=1 SV=2		18.70968	8	28	8	49.705	8.5	31000000	25000000	25000000	81.9357
Q80X41	Serine/threonine-protein kinase VRK1 OS=Mus musculus GN=Vrk1 PE=1 SV=2		12.95455	3	6	3	49.709	8.94	3000000	3000000	780000	18.05919
O54825	Bystin OS=Mus musculus GN=Bysl PE=1 SV=3		3.440367	1	3	1	49.752	7.78	2900000	3200000	2800000	9.778944

Q8R5M8	Cell adhesion molecule 1 OS=Mus musculus GN=Cadm1 PE=1 SV=2		8.333333	2	9	2	49.757	5.03	7100000	4900000	5200000	37.1313
Q99PL6	UBX domain-containing protein 6 OS=Mus musculus GN=Ubxn6 PE=1 SV=1		5.429864	1	3	1	49.765	8.54	1000000	220000	590000	8.326593
Q8K358	Phosphatidylinositol glycan anchor biosynthesis class U protein OS=Mus musculus GN=Pigu PE=1 SV=4		2.304147	1	1	1	49.772	7.72	1300000			2.576029
Q8CHH9	Septin-8 OS=Mus musculus GN=Sept8 PE=1 SV=4		14.91841	6	14	2	49.781	6	8800000	2200000	4200000	36.39564
Q91VI7	Ribonuclease inhibitor OS=Mus musculus GN=Rnh1 PE=1 SV=1		5.263158	2	4	2	49.784	4.78	2300000	1800000	2200000	11.94204
P68372	Tubulin beta-4B chain OS=Mus musculus GN=Tubb4b PE=1 SV=1		52.80899	19	153	1	49.799	4.89	80000000	44000000	47000000	471.5279
P03995	Glial fibrillary acidic protein OS=Mus musculus GN=Gfap PE=1 SV=4		59.30233	35	133	31	49.87	5.34	1.8E+08	1.1E+08	1.1E+08	386.7526
Q7TMM9	Tubulin beta-2A chain OS=Mus musculus GN=Tubb2a PE=1 SV=1		53.70787	21	163	1	49.875	4.89	80000000	42000000	49000000	501.6692
P68373	Tubulin alpha-1C chain OS=Mus musculus GN=Tuba1c PE=1 SV=1		48.77506	17	78	1	49.877	5.1	16000000	15000000		233.7272
Q62203	Splicing factor 3A subunit 2 OS=Mus musculus GN=Sf3a2 PE=1 SV=2		2.315789	1	2	1	49.88	9.54	5400000		4000000	6.23041
P68368	Tubulin alpha-4A chain OS=Mus musculus GN=Tuba4a PE=1 SV=1		49.10714	16	64	5	49.892	5.06	37000000	18000000	21000000	198.1749
Q07076	Annexin A7 OS=Mus musculus GN=Anxa7 PE=1 SV=2		10.79914	4	14	4	49.893	6.18	3900000	2700000	2900000	36.08479
P19182	Interferon-related developmental regulator 1 OS=Mus musculus GN=Ifrd1 PE=1 SV=2		6.904232	2	2	2	49.903	7.18		300000	970000	6.118073
Q04888	Transcription factor SOX-10 OS=Mus musculus GN=Sox10 PE=2 SV=2		5.579399	1	4	1	49.918	6.6	5200000	5300000	4000000	20.92147
Q9CWF2	Tubulin beta-2B chain OS=Mus musculus GN=Tubb2b PE=1 SV=1		53.70787	21	164	1	49.921	4.89	36000000	30000000	27000000	503.302
P01872	Ig mu chain C region OS=Mus musculus GN=Ighm PE=1 SV=2		15.19824	6	14	6	49.94	7.01	6500000	7300000	6800000	44.28052
Q3UX10	Tubulin alpha chain-like 3 OS=Mus musculus GN=Tubal3 PE=2 SV=2		5.381166	2	7	1	49.956	5.58	780000			15.92858
Q4PJX1	Protein odr-4 homolog OS=Mus musculus GN=Odr4 PE=1 SV=2		8.277405	3	4	3	49.966	6.14	1100000	350000		10.57119
Q3U5Q7	UMP-CMP kinase 2, mitochondrial OS=Mus musculus GN=Cmpk2 PE=1 SV=2		5.816555	2	4	2	50.005	7.27		1700000	1500000	13.17001
Q9D8N0	Elongation factor 1-gamma OS=Mus musculus GN=Eef1g PE=1 SV=3		20.59497	10	46	10	50.029	6.74	46000000	38000000	39000000	139.9644
Q922F4	Tubulin beta-6 chain OS=Mus musculus GN=Tubb6 PE=1 SV=1		38.47875	15	68	5	50.058	4.89	3800000	3500000	2300000	206.424
P10126	Elongation factor 1-alpha 1 OS=Mus musculus GN=Eef1a1 PE=1 SV=3		25.97403	11	61	5	50.082	9.01	1.1E+08	59000000	65000000	169.2116
Q9Z2I9	Succinate--CoA ligase [ADP-forming] subunit beta, mitochondrial OS=Mus musculus GN=Sucla2 PE=1 SV=2		15.55076	7	27	7	50.082	7.01	8400000	6300000	5600000	81.63161
P68369	Tubulin alpha-1A chain OS=Mus musculus GN=Tuba1a PE=1 SV=1		54.32373	18	81	1	50.104	5.06	4500000	6400000	9400000	247.524
Q8BVK9	Sp110 nuclear body protein OS=Mus musculus GN=Sp110 PE=1 SV=1		25.8427	9	21	9	50.109	9.86	5000000	6000000	6600000	63.74743
Q61398	Procollagen C-endopeptidase enhancer 1 OS=Mus musculus GN=Pcolce PE=1 SV=2		5.982906	2	3	2	50.136	8.41	1900000	1900000		8.516149
P30204	Macrophage scavenger receptor types I and II OS=Mus musculus GN=Msr1 PE=1 SV=3		2.401747	1	4	1	50.138	6.54	220000	700000	720000	9.583202
Q9WVH9	Fibulin-5 OS=Mus musculus GN=Fbln5 PE=1 SV=1		6.473214	2	7	2	50.16	4.7	1400000	1500000	820000	13.80721
P60122	RuvB-like 1 OS=Mus musculus GN=Ruvbl1 PE=1 SV=1		25	8	21	8	50.182	6.42	6500000	5800000	4500000	69.30861
Q8BIW1	Protein prune homolog OS=Mus musculus GN=Prune PE=1 SV=1		2.422907	1	1	1	50.208	5.11		1200000		2.070529
Q9R112	Sulfide:quinone oxidoreductase, mitochondrial OS=Mus musculus GN=Sqrdl PE=1 SV=3		8.888889	3	5	3	50.25	9.09	800000	1700000	1200000	13.82495
P81117	Nucleobindin-2 OS=Mus musculus GN=Nucb2 PE=1 SV=2		6.190476	2	4	2	50.273	5.15		870000	580000	11.56099
P11680	Properdin OS=Mus musculus GN=Cfp PE=2 SV=2		4.094828	1	1	1	50.293	7.84	260000			3.6918
Q61029	Lamina-associated polypeptide 2, isoforms beta/delta/epsilon/gamma OS=Mus musculus GN=Tmpo PE=1 SV=4		44.46903	14	39	6	50.342	9.45	22000000	18000000	19000000	133.1204
Q5FWK3	Rho GTPase-activating protein 1 OS=Mus musculus GN=Arhgap1 PE=1 SV=1		5.23918	2	5	2	50.379	6.44	2100000	1500000	1500000	16.28321
Q9ERD7	Tubulin beta-3 chain OS=Mus musculus GN=Tubb3 PE=1 SV=1		43.55556	17	115	4	50.386	4.93	26000000	18000000	17000000	379.6028
Q8BVY0	Ribosomal L1 domain-containing protein 1 OS=Mus musculus GN=Rsl1d1 PE=1 SV=1		15.48673	4	18	4	50.39	9.98	12000000	13000000	13000000	67.41956
P62631	Elongation factor 1-alpha 2 OS=Mus musculus GN=Eef1a2 PE=1 SV=1		19.00648	9	34	3	50.422	9.03	3600000	3200000	6800000	91.42937
P47739	Aldehyde dehydrogenase, dimeric NADP-preferring OS=Mus musculus GN=Aldh3a1 PE=1 SV=2		1.545254	1	2	1	50.449	6.95	24000000	27000000		4.879407
Q9QZI8	Serine incorporator 1 OS=Mus musculus GN=Serinc1 PE=1 SV=1		3.752759	1	2	1	50.475	6.28	540000	510000		6.548812
P50396	Rab GDP dissociation inhibitor alpha OS=Mus musculus GN=Gdi1 PE=1 SV=3		26.39821	10	25	6	50.489	5.08	10000000	7400000	8400000	79.28933
Q6KB66-1	SWISS-PROT:Q6KB66-1 Tax_Id=9606 Gene_Symbol=KRT80 Isoform 1 of Keratin, type II cytoskeletal 80	x	4.646018	2	2	2	50.494	5.67			830000	5.185039
Q61598	Rab GDP dissociation inhibitor beta OS=Mus musculus GN=Gdi2 PE=1 SV=1		31.46067	11	29	7	50.505	6.25	7800000	8000000	6700000	84.84831
Q8K2F8	Protein LSM14 homolog A OS=Mus musculus GN=Lsm14a PE=1 SV=1		9.95671	3	8	3	50.515	9.52	2600000	2700000	1500000	20.92413
O55131	Septin-7 OS=Mus musculus GN=Sept7 PE=1 SV=1		30.04587	12	43	11	50.518	8.57	50000000	38000000	37000000	116.9605
Q9R0X4	Acyl-coenzyme A thioesterase 9, mitochondrial OS=Mus musculus GN=Acot9 PE=1 SV=1		6.605923	3	5	3	50.528	8.59	1900000	1300000	1200000	10.75134
Q9Z1J3	Cysteine desulfurase, mitochondrial OS=Mus musculus GN=Nfs1 PE=1 SV=3		2.614379	1	2	1	50.538	8.16	460000		450000	5.026082
Q61644	Protein kinase C and casein kinase substrate in neurons protein 1 OS=Mus musculus GN=Pacsin1 PE=1 SV=1		17.0068	6	17	6	50.544	5.24	15000000	4700000	5200000	58.5154
Q9CWX9	Probable ATP-dependent RNA helicase DDX47 OS=Mus musculus GN=Ddx47 PE=2 SV=2		5.054945	2	4	2	50.607	9.1	2500000	500000	2000000	10.31064
Q8BH60	Golgi-associated PDZ and coiled-coil motif-containing protein OS=Mus musculus GN=Gopc PE=1 SV=1		8.423326	2	6	2	50.631	6.25	1500000	1700000	1700000	21.42837
Q924S8	Sprouty-related, EVH1 domain-containing protein 1 OS=Mus musculus GN=Spred1 PE=1 SV=1		4.279279	1	1	1	50.631	6.47				3.505178
P41241	Tyrosine-protein kinase CSK OS=Mus musculus GN=Csk PE=1 SV=2		7.111111	2	4	2	50.684	7.06	1500000	1800000	2100000	12.63773
Q9JJK2	LanC-like protein 2 OS=Mus musculus GN=Lancl2 PE=1 SV=1		4.888889	2	6	2	50.745	7.28	1800000	2100000	1500000	16.25569

Q8K3G5	Inactive serine/threonine-protein kinase VRK3 OS=Mus musculus GN=Vrk3 PE=1 SV=2		9.933775	2	4	2	50.798	8.57	1100000	1200000	1100000	16.47592
Q91YT0	NADH dehydrogenase [ubiquinone] flavoprotein 1, mitochondrial OS=Mus musculus GN=Ndufv1 PE=1 SV=1		20.0431	8	16	8	50.802	8.21	7900000	5600000	5200000	46.56507
P54071	Isocitrate dehydrogenase [NADP], mitochondrial OS=Mus musculus GN=Idh2 PE=1 SV=3		10.61947	4	12	4	50.874	8.69	5800000	4200000	3400000	33.33736
Q8R071	Inositol-trisphosphate 3-kinase A OS=Mus musculus GN=Itpka PE=1 SV=1		2.178649	1	1	1	50.903	7.72	290000			2.197409
P70347	TRAF family member-associated NF-kappa-B activator OS=Mus musculus GN=Tank PE=1 SV=1		3.348214	1	1	1	50.907	5.88	740000			2.097717
Q8CI32	BAG family molecular chaperone regulator 5 OS=Mus musculus GN=Bag5 PE=1 SV=1		6.935123	2	5	2	50.911	6.05	1200000	890000	1300000	12.9305
Q99J72	DNA dC->dU-editing enzyme APOBEC-3 OS=Mus musculus GN=Apobec3 PE=1 SV=2		9.79021	4	7	4	50.915	8.94	2500000	1300000	1300000	19.14211
P61979	Heterogeneous nuclear ribonucleoprotein K OS=Mus musculus GN=Hnrnpk PE=1 SV=1		33.26134	16	69	16	50.944	5.54	76000000	66000000	62000000	201.0945
O89053	Coronin-1A OS=Mus musculus GN=Coro1a PE=1 SV=5		28.85033	14	50	14	50.957	6.48	56000000	50000000	41000000	134.2528
Q9R111	Guanine deaminase OS=Mus musculus GN=Gda PE=1 SV=1		11.89427	5	12	5	50.981	5.53	3300000	2000000	3200000	29.44256
Q921V5	Alpha-1,6-mannosyl-glycoprotein 2-beta-N-acetylglucosaminyltransferase OS=Mus musculus GN=Mgat2 PE=1 SV=1		3.393665	1	1	1	50.997	8.59		1900000		2.269545
Q9Z0N1	Eukaryotic translation initiation factor 2 subunit 3, X-linked OS=Mus musculus GN=Eif2s3x PE=1 SV=2		19.70339	7	17	7	51.033	8.4	5900000	9900000	8800000	51.07522
Q8BLY1	SPARC-related modular calcium-binding protein 1 OS=Mus musculus GN=Smoc1 PE=2 SV=2		2.37581	1	1	1	51.043	8.37		510000		2.14625
O35857	Mitochondrial import inner membrane translocase subunit TIM44 OS=Mus musculus GN=Timm44 PE=1 SV=2		3.097345	1	2	1	51.059	8.13	2300000		830000	5.300995
P83887	Tubulin gamma-1 chain OS=Mus musculus GN=Tubg1 PE=1 SV=1		1.995565	1	3	1	51.069	6.02	2300000	1600000	1900000	6.942805
Q9WTM5	RuvB-like 2 OS=Mus musculus GN=Ruvbl2 PE=1 SV=3		21.38229	8	17	8	51.081	5.64	9700000	10000000	8900000	50.53304
Q76LS9	Ubiquitin carboxyl-terminal hydrolase MINDY-1 OS=Mus musculus GN=Fam63a PE=1 SV=1		4.273504	1	1	1	51.194	4.73		1400000		3.545945
P08779	SWISS-PROT:P08779 Tax_Id=9606 Gene_Symbol=KRT16 Keratin, type I cytoskeletal 16	x	54.75687	24	67	11	51.236	5.05	3100000	2400000	26000000	187.3994
P06332	T-cell surface glycoprotein CD4 OS=Mus musculus GN=Cd4 PE=1 SV=1		3.063457	1	1	1	51.264	9.16	510000			3.62666
Q99L43	Phosphatidate cytidylyltransferase 2 OS=Mus musculus GN=Cds2 PE=1 SV=1		4.279279	1	6	1	51.28	7.05	12000000	7400000	9000000	21.98853
Q91X72	Hemopexin OS=Mus musculus GN=Hpx PE=1 SV=2		3.26087	1	3	1	51.285	7.8	630000	630000	890000	10.47535
O55222	Integrin-linked protein kinase OS=Mus musculus GN=Ilk PE=1 SV=2		8.628319	4	5	4	51.34	8.07	2200000	2900000		10.70155
Q99JY0	Trifunctional enzyme subunit beta, mitochondrial OS=Mus musculus GN=Hadhb PE=1 SV=1		18.52632	8	21	8	51.353	9.38	9000000	5500000	7900000	54.81621
Q5SUF2	Luc7-like protein 3 OS=Mus musculus GN=Luc7l3 PE=1 SV=1		13.65741	4	9	4	51.419	9.77	3800000	4200000	2800000	30.44261
Q6PAC3	DDB1- and CUL4-associated factor 13 OS=Mus musculus GN=Dcaf13 PE=2 SV=2		9.438202	3	7	3	51.423	9.32	980000	1800000	1200000	18.55675
P40124	Adenylyl cyclase-associated protein 1 OS=Mus musculus GN=Cap1 PE=1 SV=4		28.05907	9	32	9	51.532	7.52	11000000	11000000	9500000	108.643
P30416	Peptidyl-prolyl cis-trans isomerase FKBP4 OS=Mus musculus GN=Fkbp4 PE=1 SV=5		26.63755	11	29	11	51.54	5.72	12000000	11000000	9600000	84.91546
P61202	COP9 signalosome complex subunit 2 OS=Mus musculus GN=Cops2 PE=1 SV=1		5.417607	2	4	2	51.564	5.53	2200000	2200000	360000	11.63576
P58242	Acid sphingomyelinase-like phosphodiesterase 3b OS=Mus musculus GN=Smpdl3b PE=1 SV=1		1.973684	1	2	1	51.567	6.43	2300000	1900000		4.998693
Q8BTJ4	Bis(5'-adenosyl)-triphosphatase enpp4 OS=Mus musculus GN=Enpp4 PE=1 SV=1		2.631579	1	2	1	51.578	6.73	2200000	1500000		5.449318
P02533	SWISS-PROT:P02533 Tax_Id=9606 Gene_Symbol=KRT14 Keratin, type I cytoskeletal 14	x	54.23729	25	80	5	51.589	5.16	16000000	7800000	70000000	228.052
Q06890	Clusterin OS=Mus musculus GN=Clu PE=1 SV=1		29.6875	11	35	11	51.623	5.67	14000000	11000000	9700000	112.8728
Q8VDD8	WAS protein family homolog 1 OS=Mus musculus GN=Wash1 PE=1 SV=1		12.21053	4	6	4	51.627	5.44	1400000	1300000	1400000	16.21618
Q2NL51	Glycogen synthase kinase-3 alpha OS=Mus musculus GN=Gsk3a PE=1 SV=2		4.897959	2	2	1	51.628	8.81				4.308114
P27808	Alpha-1,3-mannosyl-glycoprotein 2-beta-N-acetylglucosaminyltransferase OS=Mus musculus GN=Mgat1 PE=1 SV=1		3.579418	1	3	1	51.658	8.9	1100000	1200000	1600000	11.24547
Q6P1F6	Serine/threonine-protein phosphatase 2A 55 kDa regulatory subunit B alpha isoform OS=Mus musculus GN=Ppp2r2a PE=1 SV=1		8.277405	3	7	3	51.659	6.2	4800000	3500000	4600000	20.95514
Q8BM72	Heat shock 70 kDa protein 13 OS=Mus musculus GN=Hspa13 PE=1 SV=1		10.4034	4	10	4	51.677	5.63	1500000	1000000	1000000	24.22155
Q9CZU6	Citrate synthase, mitochondrial OS=Mus musculus GN=Cs PE=1 SV=1		20.25862	10	24	10	51.703	8.57	36000000	33000000	26000000	63.44998
Q91YJ2	Sorting nexin-4 OS=Mus musculus GN=Snx4 PE=1 SV=1		8.666667	3	5	3	51.745	5.8	1900000	1800000	540000	14.67233
Q3UFK8	FERM domain-containing protein 8 OS=Mus musculus GN=Frmd8 PE=1 SV=2		9.871245	3	7	3	51.795	6.44	1100000	1400000	1500000	16.4374
P97855	Ras GTPase-activating protein-binding protein 1 OS=Mus musculus GN=G3bp1 PE=1 SV=1		20.43011	7	17	7	51.797	5.59	6000000	6200000	5000000	53.90018
Q9DA19	Corepressor interacting with RBPJ 1 OS=Mus musculus GN=Cir1 PE=1 SV=2		3.111111	1	1	1	51.806	9.92		520000		2.178396
Q8CI78	Required for meiotic nuclear division protein 1 homolog OS=Mus musculus GN=Rmnd1 PE=1 SV=1		5.555556	2	2	2	51.81	7.99	440000	360000		5.48125
Q8BXZ1	Protein disulfide-isomerase TMX3 OS=Mus musculus GN=Tmx3 PE=1 SV=2		10.52632	4	11	4	51.815	5.16	7700000	8200000	7900000	36.45691
Q8BGH2	Sorting and assembly machinery component 50 homolog OS=Mus musculus GN=Samm50 PE=1 SV=1		6.609808	2	5	2	51.831	6.8	2300000	710000	610000	9.230119
Q9CQU1	Microfibrillar-associated protein 1 OS=Mus musculus GN=Mfap1 PE=1 SV=1		16.6287	4	9	4	51.923	4.98	1200000	2000000	2400000	22.48174
Q62376	U1 small nuclear ribonucleoprotein 70 kDa OS=Mus musculus GN=Snrnp70 PE=1 SV=2		36.38393	12	36	12	51.961	9.94	16000000	15000000	15000000	111.008
Q3UJU9	Regulator of microtubule dynamics protein 3 OS=Mus musculus GN=Rmdn3 PE=1 SV=2		15.31915	5	14	5	51.997	5.21	3500000	3700000	3700000	42.28756
P28659	CUGBP Elav-like family member 1 OS=Mus musculus GN=Celf1 PE=1 SV=2		2.674897	1	1	1	52.074	8.46		2600000		2.820326
Q91WM3	U3 small nucleolar RNA-interacting protein 2 OS=Mus musculus GN=Rrp9 PE=1 SV=1		11.57895	5	9	5	52.075	7.88	1500000	2400000	2200000	23.21111
Q8BKE6	Cytochrome P450 20A1 OS=Mus musculus GN=Cyp20a1 PE=1 SV=1		19.48052	7	11	7	52.116	6.95	3600000	4300000	2700000	31.77752
Q6P5G6	UBX domain-containing protein 7 OS=Mus musculus GN=Ubxn7 PE=1 SV=2		2.997859	1	1	1	52.132	5.03			780000	2.899275
Q9Z2W0	Aspartyl aminopeptidase OS=Mus musculus GN=Dnpep PE=1 SV=2		10.99366	4	9	4	52.174	7.25	2300000	2100000	1900000	24.47684

P60229	Eukaryotic translation initiation factor 3 subunit E OS=Mus musculus GN=Eif3e PE=1 SV=1		27.19101	11	29	11	52.187	6.04	12000000	12000000	7400000	78.68414
P70290	55 kDa erythrocyte membrane protein OS=Mus musculus GN=Mpp1 PE=1 SV=1		1.93133	1	1	1	52.194	7.2	790000			2.437427
P61620	Protein transport protein Sec61 subunit alpha isoform 1 OS=Mus musculus GN=Sec61a1 PE=1 SV=2		8.613445	4	8	4	52.231	8.06	5400000	860000	7200000	16.93311
Q8CBW3	Abl interactor 1 OS=Mus musculus GN=Abi1 PE=1 SV=3		6.860707	3	6	2	52.255	7.64	2900000	2400000	1700000	18.4411
Q9Z2D6	Methyl-CpG-binding protein 2 OS=Mus musculus GN=Mecp2 PE=1 SV=1		19.00826	6	16	6	52.275	9.96	2900000	3200000	2100000	57.07639
Q920L1	Fatty acid desaturase 1 OS=Mus musculus GN=Fads1 PE=1 SV=1		2.013423	1	1	1	52.289	9.29		450000		2.261339
P46938	Transcriptional coactivator YAP1 OS=Mus musculus GN=Yap1 PE=1 SV=2		3.483607	1	1	1	52.351	5.11		120000		3.094217
Q8C650	Septin-10 OS=Mus musculus GN=Sept10 PE=1 SV=1		4.646018	2	7	1	52.388	6.6	3800000	3400000	3100000	18.91764
Q9EPK6	Nucleotide exchange factor SIL1 OS=Mus musculus GN=Sil1 PE=1 SV=2		3.655914	1	1	1	52.397	5.27	420000			2.270218
Q99M87	DnaJ homolog subfamily A member 3, mitochondrial OS=Mus musculus GN=Dnaja3 PE=1 SV=1		5.833333	2	5	2	52.41	9.22	2100000	1400000	710000	12.96625
Q3TDN2	FAS-associated factor 2 OS=Mus musculus GN=Faf2 PE=1 SV=2		4.719101	2	5	2	52.438	5.47	1400000	1800000	1200000	14.47376
Q8CFE3	REST corepressor 1 OS=Mus musculus GN=Rcor1 PE=1 SV=2		6.498952	2	4	2	52.461	7.03	1300000	1500000	1900000	13.60282
Q8R016	Bleomycin hydrolase OS=Mus musculus GN=Blmh PE=1 SV=1		5.494505	2	5	2	52.477	6.48	2200000	1700000	5100000	14.30822
Q99K23	Ufm1-specific protease 2 OS=Mus musculus GN=Ufsp2 PE=1 SV=1		2.386117	1	1	1	52.482	6.76		2100000		2.118114
D3Z6Q9	Bridging integrator 2 OS=Mus musculus GN=Bin2 PE=1 SV=1		7.157464	3	7	2	52.522	5.34	3500000	1500000	290000	21.61505
Q9CT10	Ran-binding protein 3 OS=Mus musculus GN=Ranbp3 PE=1 SV=2		6.924644	2	4	2	52.541	5.12	260000	390000	220000	15.20976
Q9D379	Epoxide hydrolase 1 OS=Mus musculus GN=Ephx1 PE=1 SV=2		1.978022	1	3	1	52.543	8.35	870000	690000	550000	6.42619
Q91WD5	NADH dehydrogenase [ubiquinone] iron-sulfur protein 2, mitochondrial OS=Mus musculus GN=Ndufs2 PE=1 SV=1		6.695464	3	9	3	52.592	6.99	5200000	4000000	4200000	25.30415
P08414	Calcium/calmodulin-dependent protein kinase type IV OS=Mus musculus GN=Camk4 PE=1 SV=2		3.411514	1	1	1	52.594	4.93	140000			2.360332
P49769	Presenilin-1 OS=Mus musculus GN=Psen1 PE=1 SV=1		4.068522	1	1	1	52.606	5.38			430000	2.57953
P70402	Myosin-binding protein H OS=Mus musculus GN=Mybph PE=2 SV=2		1.656315	1	2	1	52.609	6.1	290000	4900000		3.933234
Q99JR5	Tubulointerstitial nephritis antigen-like OS=Mus musculus GN=Tinagl1 PE=1 SV=1		6.866953	2	6	2	52.63	6.77	2700000	2500000	2100000	20.09169
P56959	RNA-binding protein FUS OS=Mus musculus GN=Fus PE=1 SV=1		14.09266	5	23	5	52.642	9.36	37000000	30000000	24000000	62.71459
Q9WTJ4	Flt3-interacting zinc finger protein 1 OS=Mus musculus GN=Fiz1 PE=1 SV=1		6	1	1	1	52.652	7.94		460000		3.181532
Q08857	Platelet glycoprotein 4 OS=Mus musculus GN=Cd36 PE=1 SV=2		2.542373	1	2	1	52.664	8.35	1500000	1400000		5.326244
Q91YU8	Suppressor of SWI4 1 homolog OS=Mus musculus GN=Ppan PE=1 SV=2		5.744681	2	2	2	52.724	10.32			4200000	6.160155
Q9D1A2	Cytosolic non-specific dipeptidase OS=Mus musculus GN=Cndp2 PE=1 SV=1		6.105263	2	6	2	52.734	5.66	1700000	1100000	760000	15.73093
Q9JMD0	BUB3-interacting and GLEBS motif-containing protein ZNF207 OS=Mus musculus GN=Znf207 PE=1 SV=1		2.626263	1	3	1	52.758	9.1	2700000	2700000	2200000	9.445307
Q9CZ13	Cytochrome b-c1 complex subunit 1, mitochondrial OS=Mus musculus GN=Uqcrc1 PE=1 SV=2		28.33333	11	28	11	52.819	6.21	24000000	16000000	14000000	90.45197
Q9CYT6	Adenylyl cyclase-associated protein 2 OS=Mus musculus GN=Cap2 PE=1 SV=1		8.403361	3	6	3	52.829	6.43	2000000	1100000	1100000	18.14563
Q9D8W5	26S proteasome non-ATPase regulatory subunit 12 OS=Mus musculus GN=Psmd12 PE=1 SV=4		14.91228	5	11	5	52.861	7.06	12000000	11000000	10000000	36.73282
Q8BMD8	Calcium-binding mitochondrial carrier protein SCaMC-1 OS=Mus musculus GN=Slc25a24 PE=1 SV=1		1.684211	1	2	1	52.868	7.43		6000000	5400000	4.500354
O08663	Methionine aminopeptidase 2 OS=Mus musculus GN=Metap2 PE=1 SV=1		25.52301	10	37	10	52.888	5.82	12000000	12000000	10000000	115.6377
D3YZP9	Coiled-coil domain-containing protein 6 OS=Mus musculus GN=Ccdc6 PE=1 SV=1		15.13859	6	9	6	52.906	7.34	3100000	2200000	1300000	21.8118
Q9D394	Protein RUFY3 OS=Mus musculus GN=Rufy3 PE=1 SV=1		10.66098	4	8	4	52.973	5.49	2400000	1600000	1800000	23.37296
Q8CBY8	Dynactin subunit 4 OS=Mus musculus GN=Dctn4 PE=1 SV=1		5.353319	1	1	1	53.023	7.72	210000			0
Q9WUM4	Coronin-1C OS=Mus musculus GN=Coro1c PE=1 SV=2		15.82278	7	18	7	53.087	7.08	8400000	8400000	8200000	50.97341
Q9JI44	DNA methyltransferase 1-associated protein 1 OS=Mus musculus GN=Dmap1 PE=1 SV=1		7.264957	2	3	2	53.097	9.5	1400000		1800000	9.961209
Q8VEJ4	Notchless protein homolog 1 OS=Mus musculus GN=Nle1 PE=1 SV=4		2.474227	1	1	1	53.101	7.17	550000			1.878667
A2A6T1	Cerebellar degeneration-related protein 2-like OS=Mus musculus GN=Cdr2l PE=1 SV=1		2.150538	1	1	1	53.18	5.76	460000			2.533263
Q9D8T2	Gasdermin-D OS=Mus musculus GN=Gsdmdc1 PE=1 SV=1		4.517454	2	3	2	53.204	5.1	730000		880000	6.071541
Q9DCD0	6-phosphogluconate dehydrogenase, decarboxylating OS=Mus musculus GN=Pgd PE=1 SV=3		24.84472	9	24	9	53.213	7.23	10000000	9600000	5000000	72.05903
P53395	Lipoamide acyltransferase component of branched-chain alpha-keto acid dehydrogenase complex, mitochondrial OS=Mus muscul		1.86722	1	3	1	53.213	8.6	1600000	1100000	960000	8.069823
P97861	Keratin, type II cuticular Hb6 OS=Mus musculus GN=Krt86 PE=2 SV=2		52.26337	27	81	8	53.217	5.76	11000000	14000000	27000000	242.6163
Q63850	Nuclear pore glycoprotein p62 OS=Mus musculus GN=Nup62 PE=1 SV=2		4.562738	2	6	2	53.222	5.31	1500000	1400000	1500000	18.06622
Q00493	Carboxypeptidase E OS=Mus musculus GN=Cpe PE=1 SV=2		3.781513	2	3	2	53.222	5.19	1500000		1100000	7.198116
P53986	Monocarboxylate transporter 1 OS=Mus musculus GN=Slc16a1 PE=1 SV=1		6.693712	2	6	2	53.232	7.47	6600000	6400000	6200000	18.96536
Q9D0L8	mRNA cap guanine-N7 methyltransferase OS=Mus musculus GN=Rnmt PE=1 SV=1		9.247312	4	9	4	53.258	6.48	1900000	1900000	1600000	22.92646
Q9QZL0	Receptor-interacting serine/threonine-protein kinase 3 OS=Mus musculus GN=Ripk3 PE=1 SV=2		2.880658	1	2	1	53.289	7.53	880000	990000		6.628897
Q9R0M4	Podocalyxin OS=Mus musculus GN=Podxl PE=1 SV=2		2.982107	1	1	1	53.356	4.97	1800000			2.752218
P70257	Nuclear factor 1 X-type OS=Mus musculus GN=Nfix PE=1 SV=2		2.04918	1	1	1	53.361	8.68			410000	2.304252
Q9JI13	Something about silencing protein 10 OS=Mus musculus GN=Utp3 PE=1 SV=1		11.30064	3	7	3	53.367	5.54	1500000	1700000	1800000	24.11856
Q02819	Nucleobindin-1 OS=Mus musculus GN=Nucb1 PE=1 SV=2		30.50109	11	29	11	53.376	5.07	5500000	7300000	6200000	93.42273

Q99LD4	COP9 signalosome complex subunit 1 OS=Mus musculus GN=Gps1 PE=1 SV=1		7.643312	3	7	3	53.408	6.84	2600000	2500000	1800000	18.81662
P31001	Desmin OS=Mus musculus GN=Des PE=1 SV=3		11.51386	6	28	2	53.465	5.27	900000	1300000	990000	78.5442
Q9JLJ2	4-trimethylaminobutyraldehyde dehydrogenase OS=Mus musculus GN=Aldh9a1 PE=1 SV=1		3.846154	1	3	1	53.48	6.98	1900000	2000000	2000000	9.646425
P26369	Splicing factor U2AF 65 kDa subunit OS=Mus musculus GN=U2af2 PE=1 SV=3		12.84211	5	15	5	53.483	9.09	6000000	5600000	5200000	36.70462
Q8CD15	Bifunctional lysine-specific demethylase and histidyl-hydroxylase MINA OS=Mus musculus GN=Mina PE=1 SV=2		6.236559	2	6	2	53.483	6.38	2300000	1900000	2100000	15.6424
Q3TKY6	Peptidyl-prolyl cis-trans isomerase CWC27 homolog OS=Mus musculus GN=Cwc27 PE=1 SV=1		8.955224	2	3	2	53.51	5.53	1100000		1700000	10.4561
P21614	Vitamin D-binding protein OS=Mus musculus GN=Gc PE=1 SV=2		6.302521	2	4	2	53.565	5.5	1700000	1600000	1200000	9.728937
Q9EPM5	Syncoilin OS=Mus musculus GN=Sync PE=1 SV=1		2.12766	1	1	1	53.597	4.53				2.461499
P20152	Vimentin OS=Mus musculus GN=Vim PE=1 SV=3		79.82833	50	338	39	53.655	5.12	9.8E+08	9.6E+08	9E+08	1003.603
P28658	Ataxin-10 OS=Mus musculus GN=Atxn10 PE=1 SV=2		9.052632	4	7	4	53.673	5.25	1800000	2700000	1600000	19.80994
REFSEQ:XP	Tax_Id=10090 Gene_Symbol=Krt33b keratin complex 1, acidic, gene 3	x	34.66387	17	45	3	53.694	5.29	2100000	3000000	4700000	120.4206
Q64282	Interferon-induced protein with tetratricopeptide repeats 1 OS=Mus musculus GN=Ifit1 PE=1 SV=2		33.47732	14	40	14	53.703	7.52	29000000	27000000	26000000	130.3405
Q64735	Complement component receptor 1-like protein OS=Mus musculus GN=Cr1l PE=1 SV=1		2.277433	1	2	1	53.728	6.65		4600000		5.338518
P13379	T-cell surface glycoprotein CD5 OS=Mus musculus GN=Cd5 PE=1 SV=1		2.42915	1	1	1	53.815	8.21	180000			1.840122
Q8K124	Pleckstrin homology domain-containing family O member 2 OS=Mus musculus GN=Plekho2 PE=1 SV=1		7.272727	2	2	2	53.839	5.5	310000			4.97532
Q8VDL4	ADP-dependent glucokinase OS=Mus musculus GN=Adpgk PE=1 SV=2		10.68548	3	5	3	53.869	5.62	990000	1200000	1700000	17.13618
Q9WUM3	Coronin-1B OS=Mus musculus GN=Coro1b PE=1 SV=1		18.38843	8	21	8	53.878	5.78	10000000	10000000	10000000	67.81333
Q61655	ATP-dependent RNA helicase DDX19A OS=Mus musculus GN=Ddx19a PE=1 SV=2		3.138075	1	2	1	53.899	6.67		2900000	2500000	6.736555
P47740	Fatty aldehyde dehydrogenase OS=Mus musculus GN=Aldh3a2 PE=1 SV=2		12.19008	5	11	5	53.936	8.35	3800000	3100000	3000000	30.88998
P17809	Solute carrier family 2, facilitated glucose transporter member 1 OS=Mus musculus GN=Slc2a1 PE=1 SV=4		2.03252	1	3	1	53.949	8.87	2800000	3000000	3200000	7.903014
Q8BKZ9	Pyruvate dehydrogenase protein X component, mitochondrial OS=Mus musculus GN=Pdhx PE=1 SV=1		7.58483	3	6	3	53.965	7.75	1400000	1600000	780000	18.18117
O88947	Coagulation factor X OS=Mus musculus GN=F10 PE=1 SV=1		2.286902	1	2	1	53.983	5.66	380000	430000		4.227038
Q6NSQ7	Protein LTV1 homolog OS=Mus musculus GN=Ltv1 PE=1 SV=2		2.553191	1	1	1	53.99	4.92	760000			2.637816
O35114	Lysosome membrane protein 2 OS=Mus musculus GN=Scarb2 PE=1 SV=3		8.786611	3	7	3	54.009	5.1	1400000	1100000	1300000	17.12673
Q8BH43	Wiskott-Aldrich syndrome protein family member 2 OS=Mus musculus GN=Wasf2 PE=1 SV=1		8.450704	4	10	4	54.04	5.53	2700000	7500000	6700000	27.2411
Q8R180	ERO1-like protein alpha OS=Mus musculus GN=Ero1a PE=1 SV=2		5.172414	2	4	2	54.05	6.54	2500000	3700000		13.87662
P97379	Ras GTPase-activating protein-binding protein 2 OS=Mus musculus GN=G3bp2 PE=1 SV=2		17.63485	6	15	6	54.055	5.62	4700000	3600000	3100000	43.25739
Q4FZF3	Probable ATP-dependent RNA helicase DDX49 OS=Mus musculus GN=Ddx49 PE=2 SV=1		2.708333	1	1	1	54.06	8.78		900000		2.783075
P11798	Calcium/calmodulin-dependent protein kinase type II subunit alpha OS=Mus musculus GN=Camk2a PE=1 SV=2		30.33473	12	31	8	54.081	7.08	30000000	18000000	12000000	96.94721
P54823	Probable ATP-dependent RNA helicase DDX6 OS=Mus musculus GN=Ddx6 PE=1 SV=1		14.07867	5	12	5	54.157	8.66	1700000	3700000	3300000	32.77655
P70315	Wiskott-Aldrich syndrome protein homolog OS=Mus musculus GN=Was PE=1 SV=1		2.884615	1	3	1	54.158	6.7	1600000	1100000	1200000	9.463088
Q2TPA8	Hydroxysteroid dehydrogenase-like protein 2 OS=Mus musculus GN=Hsdl2 PE=1 SV=1		2.244898	1	2	1	54.174	6.74	2100000		1400000	5.0633
Q6PDL0	Cytoplasmic dynein 1 light intermediate chain 2 OS=Mus musculus GN=Dync1li2 PE=1 SV=2		18.08943	6	15	5	54.185	6.28	3500000	2800000	2000000	40.61767
P49710	Hematopoietic lineage cell-specific protein OS=Mus musculus GN=Hcls1 PE=1 SV=2		16.25514	6	22	6	54.207	4.84	6000000	6900000	3200000	72.26649
P15331	Peripherin OS=Mus musculus GN=Prph PE=1 SV=2		27.57895	14	47	10	54.235	5.52	7900000	9000000	8600000	127.6689
Q9Z0H4	CUGBP Elav-like family member 2 OS=Mus musculus GN=Celf2 PE=1 SV=1		3.543307	1	4	1	54.236	8.76	1200000	1600000	670000	12.44571
O08749	Dihydrolipoyl dehydrogenase, mitochondrial OS=Mus musculus GN=Dld PE=1 SV=2		15.71709	8	14	8	54.238	7.9	8100000	6300000	6100000	39.1842
Q91YD9	Neural Wiskott-Aldrich syndrome protein OS=Mus musculus GN=Wasl PE=1 SV=1		5.788423	2	2	2	54.24	7.93	840000			4.893657
Q9D2Y4	Mixed lineage kinase domain-like protein OS=Mus musculus GN=Mlkl PE=1 SV=1		14.61864	6	13	6	54.283	8.25	3500000	2400000	2900000	32.61688
O55026	Ectonucleoside triphosphate diphosphohydrolase 2 OS=Mus musculus GN=Entpd2 PE=1 SV=2		2.626263	1	1	1	54.285	8.37	580000			2.395683
O88746	Target of Myb protein 1 OS=Mus musculus GN=Tom1 PE=1 SV=1		2.03252	1	1	1	54.291	4.94				1.800294
Q8VCX5	Calcium uptake protein 1, mitochondrial OS=Mus musculus GN=Micu1 PE=1 SV=1		2.725367	1	1	1	54.318	8.59			1200000	3.169581
P97807	Fumarate hydratase, mitochondrial OS=Mus musculus GN=Fh PE=1 SV=3		5.128205	2	6	2	54.322	9.04	6400000	4600000	5100000	20.76997
P32921	Tryptophan--tRNA ligase, cytoplasmic OS=Mus musculus GN=Wars PE=1 SV=2		22.86902	8	25	8	54.323	6.89	16000000	18000000	10000000	80.17342
Q8BYU6	Torsin-1A-interacting protein 2 OS=Mus musculus GN=Tor1aip2 PE=1 SV=1		17.13147	5	9	5	54.463	4.86	2300000	2300000	1600000	27.92367
Q61553	Fascin OS=Mus musculus GN=Fscn1 PE=1 SV=4		5.070994	2	3	2	54.474	6.89	1600000	1100000	3000000	7.454178
Q9Z2H2	Regulator of G-protein signaling 6 OS=Mus musculus GN=Rgs6 PE=1 SV=2		2.542373	1	2	1	54.497	7.42	1500000	810000		5.904659
Q99K48	Non-POU domain-containing octamer-binding protein OS=Mus musculus GN=Nono PE=1 SV=3		44.18605	17	53	16	54.506	8.95	19000000	18000000	20000000	152.1404
Q6IMF0	Keratin, type II cuticular Hb3 OS=Mus musculus GN=Krt83 PE=2 SV=2		46.06061	23	66	3	54.594	6.06	3500000	4900000	7500000	188.0773
Q99M31	Heat shock 70 kDa protein 14 OS=Mus musculus GN=Hspa14 PE=1 SV=2		3.929273	2	3	2	54.616	5.92	1400000	1900000	2200000	7.97002
Q61151	Serine/threonine-protein phosphatase 2A 56 kDa regulatory subunit epsilon isoform OS=Mus musculus GN=Ppp2r5e PE=1 SV=3		2.783726	1	4	1	54.679	6.95	440000	1900000	1500000	10.07508
O35892	Nuclear autoantigen Sp-100 OS=Mus musculus GN=Sp100 PE=1 SV=2		13.48548	5	11	5	54.692	8.66	5000000	6900000	6600000	33.17615
Q80W54	CAAX prenyl protease 1 homolog OS=Mus musculus GN=Zmpste24 PE=1 SV=2		6.736842	3	7	3	54.699	6.95	3200000	3900000	4900000	22.89551

Q8K0E8	Fibrinogen beta chain OS=Mus musculus GN=Fgb PE=1 SV=1		38.25364	16	40	16	54.718	7.08	17000000	18000000	15000000	118.2919
P56183	Ribosomal RNA processing protein 1 homolog A OS=Mus musculus GN=Rrp1 PE=1 SV=2		7.692308	3	6	3	54.743	5.17	780000	1500000	1400000	18.76981
Q3UHL1	CaM kinase-like vesicle-associated protein OS=Mus musculus GN=Camkv PE=1 SV=2		29.10156	7	17	7	54.785	5.54	4600000	1500000	1500000	60.44136
O70475	UDP-glucose 6-dehydrogenase OS=Mus musculus GN=Ugdh PE=1 SV=1		25.35497	10	24	10	54.797	7.56	11000000	12000000	8900000	68.55003
P29788	Vitronectin OS=Mus musculus GN=Vtn PE=1 SV=2		3.974895	2	2	2	54.814	5.88	5600000	1300000		4.191104
P54822	Adenylosuccinate lyase OS=Mus musculus GN=Adsl PE=1 SV=2		6.404959	3	6	3	54.831	7.27	1500000	1400000	440000	15.41293
Q8VEH8	Endoplasmic reticulum lectin 1 OS=Mus musculus GN=Erlec1 PE=1 SV=1		6.832298	3	6	3	54.871	6.25	3700000	2100000	2000000	15.8511
P28661	Septin-4 OS=Mus musculus GN=Sept4 PE=1 SV=1		4.393305	1	1	1	54.901	5.87	150000			2.524114
Q8C0J6	Ankyrin repeat domain-containing protein SOWAHC OS=Mus musculus GN=Sowahc PE=1 SV=2		3.320313	1	3	1	54.905	6	1800000	1300000	1200000	10.66027
Q9CWN7	CCR4-NOT transcription complex subunit 11 OS=Mus musculus GN=Cnot11 PE=1 SV=1		3.366337	1	1	1	54.924	6.55	250000			2.359242
Q9Z1X2	Phosphatidylserine synthase 2 OS=Mus musculus GN=Ptdss2 PE=1 SV=2		2.748414	1	1	1	54.985	7.09		670000		1.981493
Q64112	Interferon-induced protein with tetratricopeptide repeats 2 OS=Mus musculus GN=Ifit2 PE=1 SV=1		7.415254	4	12	4	54.986	7.43	7000000	6400000	5300000	33.97546
Q8CBE3	WD repeat-containing protein 37 OS=Mus musculus GN=Wdr37 PE=1 SV=1		2.016129	1	1	1	55.012	7.23	1700000			1.940831
O09106	Histone deacetylase 1 OS=Mus musculus GN=Hdac1 PE=1 SV=1		8.091286	3	7	2	55.04	5.48	2000000	750000		19.03672
Q80YV2	Nuclear-interacting partner of ALK OS=Mus musculus GN=Zc3hc1 PE=1 SV=1		5.988024	2	4	2	55.161	5.38	310000	230000	650000	10.03336
Q8VHI6	Wiskott-Aldrich syndrome protein family member 3 OS=Mus musculus GN=Wasf3 PE=1 SV=1		2.39521	1	2	1	55.17	6.37	1100000	790000		4.519866
Q99KP6	Pre-mRNA-processing factor 19 OS=Mus musculus GN=Prpf19 PE=1 SV=1		13.09524	5	19	5	55.204	6.61	11000000	12000000	13000000	58.9073
Q99LF4	tRNA-splicing ligase RtcB homolog OS=Mus musculus GN=Rtcb PE=1 SV=1		20.79208	9	27	9	55.214	7.23	18000000	16000000	15000000	74.87379
Q8C522	Endonuclease domain-containing 1 protein OS=Mus musculus GN=Endod1 PE=1 SV=2		2.59481	1	3	1	55.227	6.16	5400000	4100000	4000000	10.02079
Q3TZM9	GDP-Man:Man(3)GlcNAc(2)-PP-Dol alpha-1,2-mannosyltransferase OS=Mus musculus GN=Alg11 PE=2 SV=1		2.845528	1	1	1	55.234	8.29				3.317605
P50096	Inosine-5'-monophosphate dehydrogenase 1 OS=Mus musculus GN=Impdh1 PE=1 SV=2		3.891051	2	3	1	55.243	6.8	1100000	990000		7.779828
P70288	Histone deacetylase 2 OS=Mus musculus GN=Hdac2 PE=1 SV=1		6.352459	2	4	1	55.267	5.91	720000			11.47096
P46660	Alpha-internexin OS=Mus musculus GN=Ina PE=1 SV=3		34.13174	17	48	14	55.349	5.4	19000000	7700000	8600000	140.3899
Q8CCF0	U4/U6 small nuclear ribonucleoprotein Prp31 OS=Mus musculus GN=Prpf31 PE=1 SV=3		7.214429	3	6	3	55.395	5.67	1500000	1700000	1800000	16.10511
Q99KQ4	Nicotinamide phosphoribosyltransferase OS=Mus musculus GN=Nampt PE=1 SV=1		14.66395	6	15	6	55.413	7.15	8100000	7400000	6200000	47.23067
Q5SRX1	TOM1-like protein 2 OS=Mus musculus GN=Tom1l2 PE=1 SV=1		2.95858	1	2	1	55.628	4.82	5200000		3500000	6.708646
P14576	Signal recognition particle 54 kDa protein OS=Mus musculus GN=Srp54 PE=1 SV=2		9.722222	4	9	4	55.684	8.75	4100000	6100000	2600000	27.41374
Q8BTS4	Nuclear pore complex protein Nup54 OS=Mus musculus GN=Nup54 PE=1 SV=1		2.54902	1	2	1	55.697	7.02	1000000	780000		5.798808
Q9Z2T6	Keratin, type II cuticular Hb5 OS=Mus musculus GN=Krt85 PE=1 SV=2		30.17751	17	45	2	55.723	6.42	1700000		1900000	115.6729
P24547	Inosine-5'-monophosphate dehydrogenase 2 OS=Mus musculus GN=Impdh2 PE=1 SV=2		20.03891	9	14	8	55.78	7.28	6400000	3600000	6000000	38.90067
Q9WVE8	Protein kinase C and casein kinase substrate in neurons protein 2 OS=Mus musculus GN=Pacsin2 PE=1 SV=1		12.96296	6	13	6	55.798	5.2	12000000	9700000	13000000	35.83673
Q9D8M7	PHD finger protein 10 OS=Mus musculus GN=Phf10 PE=1 SV=4		2.012072	1	2	1	55.804	6.61				4.95348
Q9D024	Coiled-coil domain-containing protein 47 OS=Mus musculus GN=Ccdc47 PE=1 SV=2		15.52795	6	13	6	55.808	4.84	3100000	3900000	4300000	36.79371
Q8BVE3	V-type proton ATPase subunit H OS=Mus musculus GN=Atp6v1h PE=1 SV=1		17.80538	6	14	6	55.819	6.61	5400000	5700000	4900000	45.58037
Q9Z127	Large neutral amino acids transporter small subunit 1 OS=Mus musculus GN=Slc7a5 PE=1 SV=2		8.398438	3	8	3	55.836	7.9	4700000	5300000	4400000	29.79941
Q14CN4-1	SWISS-PROT:Q14CN4-1 Tax_Id=9606 Gene_Symbol=KRT72 Isoform 1 of Keratin, type II cytoskeletal 72	x	7.436399	4	13	1	55.842	6.89			5500000	36.09409
Q99PP6	Tripartite motif-containing protein 34A OS=Mus musculus GN=Trim34a PE=2 SV=2		2.061856	1	2	1	55.872	8.09	2600000	2400000		4.781641
Q8BWF0	Succinate-semialdehyde dehydrogenase, mitochondrial OS=Mus musculus GN=Aldh5a1 PE=1 SV=1		10.51625	4	11	4	55.933	8.25	4500000	2700000	3100000	35.65258
Q8BJY1	26S proteasome non-ATPase regulatory subunit 5 OS=Mus musculus GN=Psmd5 PE=1 SV=4		1.388889	1	1	1	55.937	5.21	1200000			2.180315
Q8VE97	Serine/arginine-rich splicing factor 4 OS=Mus musculus GN=Srsf4 PE=2 SV=1		12.47444	6	20	3	55.947	11.4	2200000	6600000	5700000	54.67149
Q8BK67	Protein RCC2 OS=Mus musculus GN=Rcc2 PE=1 SV=1		20	8	20	8	55.948	8.72	6900000	8300000	6900000	59.10978
Q9D0K2	Succinyl-CoA:3-ketoacid coenzyme A transferase 1, mitochondrial OS=Mus musculus GN=Oxct1 PE=1 SV=1		13.07692	6	12	6	55.953	8.53	7200000	5700000	5400000	28.00671
P24529	Tyrosine 3-monooxygenase OS=Mus musculus GN=Th PE=1 SV=3		3.012048	1	1	1	55.958	6.15		670000		2.210867
Q9JMA1	Ubiquitin carboxyl-terminal hydrolase 14 OS=Mus musculus GN=Usp14 PE=1 SV=3		15.01014	5	10	5	55.966	5.24	3200000	4000000	2800000	28.4965
O35874	Neutral amino acid transporter A OS=Mus musculus GN=Slc1a4 PE=1 SV=1		10.71429	3	9	2	56.026	5.87	4200000	3000000	2900000	31.80684
Q9CPY7	Cytosol aminopeptidase OS=Mus musculus GN=Lap3 PE=1 SV=3		18.88247	8	22	8	56.106	7.72	6500000	7400000	4200000	64.53678
P56480	ATP synthase subunit beta, mitochondrial OS=Mus musculus GN=Atp5b PE=1 SV=2		41.96597	19	109	19	56.265	5.34	93000000	71000000	66000000	345.4039
Q6PD03	Serine/threonine-protein phosphatase 2A 56 kDa regulatory subunit alpha isoform OS=Mus musculus GN=Ppp2r5a PE=1 SV=1		4.938272	2	2	2	56.311	7.03		1000000		5.396792
Q6PHZ2	Calcium/calmodulin-dependent protein kinase type II subunit delta OS=Mus musculus GN=Camk2d PE=1 SV=1		21.84369	9	21	5	56.334	7.25	7500000	4700000	5100000	68.00638
Q3UYC0	Protein phosphatase 1H OS=Mus musculus GN=Ppm1h PE=1 SV=1		1.754386	1	1	1	56.345	6.73	910000			2.495507
P97360	Transcription factor ETV6 OS=Mus musculus GN=Etv6 PE=1 SV=1		1.649485	1	3	1	56.37	7.33	1800000	1600000	2000000	7.048251
P97797	Tyrosine-protein phosphatase non-receptor type substrate 1 OS=Mus musculus GN=Sirpa PE=1 SV=1		3.898635	2	4	2	56.39	8.15	980000	830000	520000	8.874052
P61922	4-aminobutyrate aminotransferase, mitochondrial OS=Mus musculus GN=Abat PE=1 SV=1		7.6	3	10	3	56.416	8.09	4500000	3300000	4400000	30.23283

Q8VCH8	UBX domain-containing protein 4 OS=Mus musculus GN=Ubxn4 PE=1 SV=1		1.383399	1	3	1	56.437	6.61	1600000	1500000	1100000	6.867962
Q9QYI3	DnaJ homolog subfamily C member 7 OS=Mus musculus GN=Dnajc7 PE=1 SV=2		13.76518	6	13	6	56.44	6.49	4200000	4000000	4000000	37.12646
P17225	Polypyrimidine tract-binding protein 1 OS=Mus musculus GN=Ptbp1 PE=1 SV=2		7.779886	3	8	2	56.443	8.34	7100000	5900000	5300000	24.13691
Q9CQF9	Prenylcysteine oxidase OS=Mus musculus GN=Pcyox1 PE=1 SV=1		8.514851	3	9	3	56.459	6.92	1900000	2000000	2100000	28.16345
P47738	Aldehyde dehydrogenase, mitochondrial OS=Mus musculus GN=Aldh2 PE=1 SV=1		15.60694	6	15	6	56.502	7.62	4300000	2300000	4700000	44.09972
P62814	V-type proton ATPase subunit B, brain isoform OS=Mus musculus GN=Atp6v1b2 PE=1 SV=1		24.46184	12	32	12	56.515	5.81	20000000	13000000	13000000	88.62255
Q61753	D-3-phosphoglycerate dehydrogenase OS=Mus musculus GN=Phgdh PE=1 SV=3		7.129456	3	9	3	56.549	6.54	6000000	6300000	4600000	28.51374
Q99K28	ADP-ribosylation factor GTPase-activating protein 2 OS=Mus musculus GN=Arfgap2 PE=1 SV=1		8.653846	4	8	4	56.563	8.18	1800000	1800000	1100000	21.39395
Q5XG73	Acyl-CoA-binding domain-containing protein 5 OS=Mus musculus GN=Acbd5 PE=1 SV=1		2.165354	1	1	1	56.578	5.41				2.383377
Q8R1Q8	Cytoplasmic dynein 1 light intermediate chain 1 OS=Mus musculus GN=Dync1li1 PE=1 SV=1		18.73805	7	15	6	56.579	6.42	3400000	5100000	2900000	44.08744
Q8BGQ1	Spermatogenesis-defective protein 39 homolog OS=Mus musculus GN=Vipas39 PE=1 SV=1		3.05499	1	3	1	56.59	7.17	1100000	900000	1100000	8.736958
P27773	Protein disulfide-isomerase A3 OS=Mus musculus GN=Pdia3 PE=1 SV=2		60	29	135	29	56.643	6.21	1.5E+08	1.4E+08	1.5E+08	413.6389
Q8BHD7	Polypyrimidine tract-binding protein 3 OS=Mus musculus GN=Ptbp3 PE=1 SV=1		3.632887	2	4	1	56.665	9.13		2700000	4200000	10.45398
Q8BZX4	Splicing regulatory glutamine/lysine-rich protein 1 OS=Mus musculus GN=Srek1 PE=2 SV=1		8.704453	3	6	3	56.73	10.48	2500000	1300000	1300000	20.93002
Q8K0C4	Lanosterol 14-alpha demethylase OS=Mus musculus GN=Cyp51a1 PE=1 SV=1		2.385686	1	1	1	56.739	8.41			810000	2.065296
O35841	Apoptosis inhibitor 5 OS=Mus musculus GN=Api5 PE=1 SV=2		9.722222	4	9	4	56.75	5.92	6700000	5600000	4200000	25.50858
Q9Z2A7	Diacylglycerol O-acyltransferase 1 OS=Mus musculus GN=Dgat1 PE=1 SV=1		5.02008	1	1	1	56.753	9.39	2000000			4.48274
Q9QXE7	F-box-like/WD repeat-containing protein TBL1X OS=Mus musculus GN=Tbl1x PE=1 SV=2		10.81594	4	7	4	56.767	5.72	2100000	2000000	1200000	20.48891
Q9JI10	Serine/threonine-protein kinase 3 OS=Mus musculus GN=Stk3 PE=1 SV=1		2.012072	1	1	1	56.819	4.98		630000		1.799347
Q9JM13	Rab5 GDP/GTP exchange factor OS=Mus musculus GN=Rabgef1 PE=1 SV=1		2.240326	1	2	1	56.833	6.79	520000	680000		4.606768
Q9D2E2	Target of EGR1 protein 1 OS=Mus musculus GN=Toe1 PE=1 SV=1		2.348337	1	1	1	56.835	6.64	480000			2.708559
Q60676	Serine/threonine-protein phosphatase 5 OS=Mus musculus GN=Ppp5c PE=1 SV=3		5.410822	2	3	2	56.84	6.2		1200000	1600000	7.392565
O70310	Glycylpeptide N-tetradecanoyltransferase 1 OS=Mus musculus GN=Nmt1 PE=1 SV=1		6.854839	3	10	3	56.852	8	3600000	3300000	2800000	30.21638
Q922V4	Pleiotropic regulator 1 OS=Mus musculus GN=Plrg1 PE=1 SV=1		2.339181	1	2	1	56.902	9.17	1300000		330000	4.931069
Q8N1N4-2	SWISS-PROT:Q8N1N4-2 Tax_Id=9606 Gene_Symbol=KRT78 Isoform 2 of Keratin, type II cytoskeletal 78	x	19.00192	10	20	6	56.93	6.02	1600000		4900000	52.74564
Q8CIM7	Cytochrome P450 2D26 OS=Mus musculus GN=Cyp2d26 PE=1 SV=1		2.4	1	1	1	56.939	6.64	370000			2.77735
Q61790	Lymphocyte activation gene 3 protein OS=Mus musculus GN=Lag3 PE=2 SV=1		2.111324	1	1	1	56.942	8.25	430000			2.247377
Q91ZJ5	UTP--glucose-1-phosphate uridylyltransferase OS=Mus musculus GN=Ugp2 PE=1 SV=3		4.724409	2	3	2	56.944	7.61	3900000	2400000		6.339203
Q99LM2	CDK5 regulatory subunit-associated protein 3 OS=Mus musculus GN=Cdk5rap3 PE=1 SV=1		1.988072	1	1	1	56.955	4.83		970000		2.85131
Q9ERA0	Alpha-globin transcription factor CP2 OS=Mus musculus GN=Tfcp2 PE=1 SV=1		2.390438	1	3	1	56.996	5.54	160000	230000	400000	8.750796
P09103	Protein disulfide-isomerase OS=Mus musculus GN=P4hb PE=1 SV=2		38.70334	17	49	17	57.023	4.88	36000000	35000000	31000000	158.6982
Q6NVG1	Lysophospholipid acyltransferase LPCAT4 OS=Mus musculus GN=Lpcat4 PE=1 SV=1		3.053435	1	1	1	57.107	8.75		360000		2.816766
Q922B2	Aspartate--tRNA ligase, cytoplasmic OS=Mus musculus GN=Dars PE=1 SV=2		37.72455	16	41	16	57.111	6.49	9100000	8100000	12000000	115.2811
P55772	Ectonucleoside triphosphate diphosphohydrolase 1 OS=Mus musculus GN=Entpd1 PE=1 SV=1		6.078431	3	10	3	57.169	5.86	5200000	2700000	3100000	22.07209
Q5XJY5	Coatomer subunit delta OS=Mus musculus GN=Arcn1 PE=1 SV=2		22.70059	11	33	11	57.193	6.21	5500000	9300000	6700000	87.56384
Q9CXG3	Peptidyl-prolyl cis-trans isomerase-like 4 OS=Mus musculus GN=Ppil4 PE=1 SV=2		2.642276	1	3	1	57.195	6.11	2500000	2400000	2600000	8.498278
Q3THK3	General transcription factor IIF subunit 1 OS=Mus musculus GN=Gtf2f1 PE=1 SV=2		11.61417	4	10	4	57.206	7.01	3200000	3300000	2700000	32.38661
P15533	Tripartite motif-containing protein 30A OS=Mus musculus GN=Trim30a PE=1 SV=2		2.419355	1	3	1	57.293	6.98	1100000	990000	980000	7.195528
Q03267	DNA-binding protein Ikaros OS=Mus musculus GN=Ikzf1 PE=1 SV=2		6.382979	3	3	2	57.299	6.52	1100000	350000		6.929445
O35633	Vesicular inhibitory amino acid transporter OS=Mus musculus GN=Slc32a1 PE=1 SV=3		4.380952	1	2	1	57.344	6.64	740000		360000	7.592052
Q9ESX5	H/ACA ribonucleoprotein complex subunit 4 OS=Mus musculus GN=Dkc1 PE=1 SV=4		13.35953	6	17	6	57.365	9.28	7400000	7800000	7100000	51.09983
Q61035	Histidine--tRNA ligase, cytoplasmic OS=Mus musculus GN=Hars PE=1 SV=2		10.21611	5	12	5	57.396	6	3100000	2700000	1800000	29.22297
Q91YW3	DnaJ homolog subfamily C member 3 OS=Mus musculus GN=Dnajc3 PE=1 SV=1		10.31746	3	5	3	57.428	5.85	1100000	590000	1800000	16.55539
P80314	T-complex protein 1 subunit beta OS=Mus musculus GN=Cct2 PE=1 SV=4		30.46729	13	35	13	57.441	6.4	15000000	13000000	11000000	111.747
Q6DVA0	LEM domain-containing protein 2 OS=Mus musculus GN=Lemd2 PE=1 SV=1		3.32681	1	3	1	57.471	9.01	670000	580000	620000	8.529951
P09242	Alkaline phosphatase, tissue-nonspecific isozyme OS=Mus musculus GN=Alpl PE=1 SV=2		1.908397	1	1	1	57.478	7.01	530000			2.062876
Q9WUB0	RanBP-type and C3HC4-type zinc finger-containing protein 1 OS=Mus musculus GN=Rbck1 PE=1 SV=2		2.362205	1	1	1	57.497	6.19		550000		2.67461
Q3UKJ7	WD40 repeat-containing protein SMU1 OS=Mus musculus GN=Smu1 PE=2 SV=2		4.483431	2	2	2	57.507	7.18	340000	3600000		5.523892
Q8C0C7	Phenylalanine--tRNA ligase alpha subunit OS=Mus musculus GN=Farsa PE=1 SV=1		9.448819	4	11	4	57.563	8.28	4100000	4200000	3900000	34.20204
Q61749	Translation initiation factor eIF-2B subunit delta OS=Mus musculus GN=Eif2b4 PE=1 SV=2		3.816794	1	2	1	57.588	9.25	510000		590000	4.958858
P00687	Alpha-amylase 1 OS=Mus musculus GN=Amy1 PE=1 SV=2	x	2.348337	1	1	1	57.607	6.96			3400000	2.587677
Q3U7U3	F-box only protein 7 OS=Mus musculus GN=Fbxo7 PE=1 SV=2		7.456979	2	4	2	57.613	5.83	950000	700000	600000	12.21275
Q9CXC9	ETS translocation variant 5 OS=Mus musculus GN=Etv5 PE=2 SV=1		5.882353	1	1	1	57.675	5.63	3300000			2.353956

O35344	Importin subunit alpha-4 OS=Mus musculus GN=Kpna3 PE=1 SV=1		7.677543	3	7	2	57.737	4.94	2800000	2800000	1600000	20.50366
Q9D0F3	Protein ERGIC-53 OS=Mus musculus GN=Lman1 PE=1 SV=1		20.88975	6	14	6	57.753	6.34	12000000	5500000	5600000	42.20599
Q5XKE5	SWISS-PROT:Q5XKE5 Tax_Id=9606 Gene_Symbol=KRT79 Keratin, type II cytoskeletal 79	x	10.46729	8	25	1	57.774	7.2			1300000	60.04013
P52480	Pyruvate kinase PKM OS=Mus musculus GN=Pkm PE=1 SV=4		52.16573	22	92	22	57.808	7.47	1.5E+08	1.4E+08	1.2E+08	330.3075
Q9EQ20	Methylmalonate-semialdehyde dehydrogenase [acylating], mitochondrial OS=Mus musculus GN=Aldh6a1 PE=1 SV=1		3.738318	1	3	1	57.879	8.07	1400000	960000	370000	9.521549
O35343	Importin subunit alpha-3 OS=Mus musculus GN=Kpna4 PE=1 SV=1		9.40499	4	9	3	57.887	4.94	1400000	1500000	1300000	21.33425
P52293	Importin subunit alpha-1 OS=Mus musculus GN=Kpna2 PE=1 SV=2		8.506616	3	8	3	57.892	5.68	2400000	2700000	1800000	25.971
Q8CIN4	Serine/threonine-protein kinase PAK 2 OS=Mus musculus GN=Pak2 PE=1 SV=1		8.969466	3	6	1	57.894	5.77	1000000	1200000	740000	15.79273
P06240	Proto-oncogene tyrosine-protein kinase LCK OS=Mus musculus GN=Lck PE=1 SV=4		5.89391	3	7	1	57.906	5.25			460000	16.81393
O08900	Zinc finger protein Aiolos OS=Mus musculus GN=Ikzf3 PE=1 SV=2		4.733728	2	2	1	57.93	6.33				4.254487
Q9JHP7	KDEL motif-containing protein 1 OS=Mus musculus GN=Kdelc1 PE=1 SV=1		7.569721	3	4	3	57.948	7.9	1700000	660000	2900000	10.78775
P80317	T-complex protein 1 subunit zeta OS=Mus musculus GN=Cct6a PE=1 SV=3		19.96234	10	31	10	57.968	7.08	19000000	15000000	18000000	89.33209
P80315	T-complex protein 1 subunit delta OS=Mus musculus GN=Cct4 PE=1 SV=3		23.37662	11	33	11	58.03	8.02	12000000	9000000	10000000	91.10856
Q9DC61	Mitochondrial-processing peptidase subunit alpha OS=Mus musculus GN=Pmpca PE=1 SV=1		3.625954	1	2	1	58.242	6.83	460000		410000	5.728467
Q03963	Interferon-induced, double-stranded RNA-activated protein kinase OS=Mus musculus GN=Eif2ak2 PE=1 SV=2		8.737864	4	8	4	58.243	8.57	4300000	3900000	3900000	26.08643
P10852	4F2 cell-surface antigen heavy chain OS=Mus musculus GN=Slc3a2 PE=1 SV=1		23.95437	11	34	11	58.3	5.91	19000000	19000000	18000000	96.93368
Q9DAW6	U4/U6 small nuclear ribonucleoprotein Prp4 OS=Mus musculus GN=Prpf4 PE=1 SV=1		5.182342	2	6	2	58.333	7.28	3200000	2900000	3000000	20.61656
P26638	Serine--tRNA ligase, cytoplasmic OS=Mus musculus GN=Sars PE=1 SV=3		6.054688	2	5	2	58.352	6.3	4100000	4700000	3700000	16.36642
Q99MN9	Propionyl-CoA carboxylase beta chain, mitochondrial OS=Mus musculus GN=Pccb PE=1 SV=2		5.360444	2	6	2	58.372	7.66	2200000	1900000	1700000	19.54549
P33610	DNA primase large subunit OS=Mus musculus GN=Prim2 PE=1 SV=1		2.376238	1	2	1	58.372	8.32	880000	850000		5.37997
Q9CWK8	Sorting nexin-2 OS=Mus musculus GN=Snx2 PE=1 SV=2		26.39692	12	21	10	58.435	5.12	5500000	4100000	4300000	56.96512
P51912	Neutral amino acid transporter B(0) OS=Mus musculus GN=Slc1a5 PE=1 SV=2		4.882459	2	4	2	58.445	7.84	1300000	1500000	1700000	14.70304
Q99J23	GH3 domain-containing protein OS=Mus musculus GN=Ghdc PE=2 SV=2		3.383459	1	1	1	58.471	7.56			350000	2.317839
P31266	Recombining binding protein suppressor of hairless OS=Mus musculus GN=Rbpj PE=1 SV=1		3.041825	1	2	1	58.5	8.13		1300000	1000000	5.883921
Q8BW75	Amine oxidase [flavin-containing] B OS=Mus musculus GN=Maob PE=1 SV=4		7.692308	3	5	2	58.52	8.29	1800000	2600000	580000	11.91156
Q8C729	Protein FAM126B OS=Mus musculus GN=Fam126b PE=1 SV=1		3.018868	1	1	1	58.55	7.72			1500000	2.422554
P63328	Serine/threonine-protein phosphatase 2B catalytic subunit alpha isoform OS=Mus musculus GN=Ppp3ca PE=1 SV=1		19.19386	9	25	5	58.606	5.86	10000000	6200000	6800000	73.65409
Q61074	Protein phosphatase 1G OS=Mus musculus GN=Ppm1g PE=1 SV=3		22.87823	7	15	7	58.691	4.39	2600000	3000000	2000000	41.97311
Q8R326	Paraspeckle component 1 OS=Mus musculus GN=Pspc1 PE=1 SV=1		8.030593	4	9	4	58.721	6.67	3800000	3500000	3600000	22.7677
Q9Z2F2	2'-5'-oligoadenylate synthase-like protein 2 OS=Mus musculus GN=Oasl2 PE=1 SV=2		10.62992	5	14	5	58.73	6.4	5100000	4400000	4300000	37.11777
O08795	Glucosidase 2 subunit beta OS=Mus musculus GN=Prkcsh PE=1 SV=1		26.67946	10	22	10	58.756	4.46	16000000	15000000	16000000	68.65864
P25911	Tyrosine-protein kinase Lyn OS=Mus musculus GN=Lyn PE=1 SV=4		8.398438	4	9	3	58.775	7.15		1700000	1600000	22.73113
Q9D554	Splicing factor 3A subunit 3 OS=Mus musculus GN=Sf3a3 PE=1 SV=2		16.16766	6	14	6	58.805	5.34	9000000	8700000	7100000	55.07952
P13597	Intercellular adhesion molecule 1 OS=Mus musculus GN=Icam1 PE=1 SV=1		2.607076	1	3	1	58.807	6.13	8000000	7300000	4900000	11.23505
Q9DBF1	Alpha-aminoadipic semialdehyde dehydrogenase OS=Mus musculus GN=Aldh7a1 PE=1 SV=4		3.153989	1	3	1	58.824	7.47	2000000	2300000	2100000	9.432285
P14234	Tyrosine-protein kinase Fgr OS=Mus musculus GN=Fgr PE=1 SV=2		4.255319	2	5	1	58.829	5.38			980000	12.56336
Q8C166	Copine-1 OS=Mus musculus GN=Cpne1 PE=1 SV=1		6.716418	4	7	3	58.849	5.66	830000	570000	1200000	16.5123
Q32MB2	TREMBL:Q32MB2;Q86Y46 Tax_Id=9606 Gene_Symbol=KRT73 Keratin-73	x	6.851852	4	14	0	58.887	7.23				37.69719
Q80SW1	Putative adenosylhomocysteinase 2 OS=Mus musculus GN=Ahcyl1 PE=1 SV=1		20.37736	10	23	1	58.913	6.89	2500000	1800000	1100000	57.66125
Q9WV80	Sorting nexin-1 OS=Mus musculus GN=Snx1 PE=1 SV=1		22.41379	10	23	8	58.916	5.22	9000000	7200000	5600000	63.69844
Q8K1N4	Spermatogenesis-associated serine-rich protein 2 OS=Mus musculus GN=Spats2 PE=1 SV=1		7.155963	3	7	3	58.92	9	1400000	1300000	1200000	20.7392
P70459	ETS domain-containing transcription factor ERF OS=Mus musculus GN=Erf PE=1 SV=1		7.259528	2	3	2	59.014	7.28	600000	420000		8.8813
Q9D787	Peptidyl-prolyl cis-trans isomerase-like 2 OS=Mus musculus GN=Ppil2 PE=1 SV=2		3.071017	1	4	1	59.028	8.38	940000	1100000	880000	12.70606
Q99JR8	SWI/SNF-related matrix-associated actin-dependent regulator of chromatin subfamily D member 2 OS=Mus musculus GN=Smarcd		6.403013	2	2	2	59.048	9.64		1000000		6.052085
Q3TLD5	Unconventional prefoldin RPB5 interactor OS=Mus musculus GN=Uri1 PE=1 SV=2		4.33145	1	1	1	59.048	5.07	310000			2.512231
Q8BX09	Retinoblastoma-binding protein 5 OS=Mus musculus GN=Rbbp5 PE=1 SV=2		2.230483	1	2	1	59.061	5.1	870000		900000	5.320784
Q91WQ3	Tyrosine--tRNA ligase, cytoplasmic OS=Mus musculus GN=Yars PE=1 SV=3		20.64394	10	21	10	59.068	7.01	3800000	3200000	3000000	52.60448
Q6NVF9	Cleavage and polyadenylation specificity factor subunit 6 OS=Mus musculus GN=Cpsf6 PE=1 SV=1		9.437387	4	13	4	59.116	7.15	4200000	3900000	3300000	42.91523
P48453	Serine/threonine-protein phosphatase 2B catalytic subunit beta isoform OS=Mus musculus GN=Ppp3cb PE=1 SV=2		12.19048	6	16	2	59.136	5.91	920000	860000	1000000	42.35341
Q09200	Beta-1,4 N-acetylgalactosaminyltransferase 1 OS=Mus musculus GN=B4galnt1 PE=1 SV=1		2.06379	1	2	1	59.175	8.59		490000	310000	4.48529
Q8BKX1	Brain-specific angiogenesis inhibitor 1-associated protein 2 OS=Mus musculus GN=Baiap2 PE=1 SV=2		8.971963	4	9	4	59.2	9.04	3400000	1700000	2400000	28.23295
Q00612	Glucose-6-phosphate 1-dehydrogenase X OS=Mus musculus GN=G6pdx PE=1 SV=3		8.349515	4	6	4	59.225	6.49	780000	2400000	3100000	13.17039
Q921X9	Protein disulfide-isomerase A5 OS=Mus musculus GN=Pdia5 PE=1 SV=1		5.415861	2	5	2	59.229	7.5	1400000	3500000	2000000	13.56648

E9Q1P8	Interferon regulatory factor 2-binding protein 2 OS=Mus musculus GN=Irf2bp2 PE=1 SV=1		12.10526	4	7	4	59.255	8.69	1200000	680000	510000	27.83088
Q8C7V3	U3 small nucleolar RNA-associated protein 15 homolog OS=Mus musculus GN=Utp15 PE=1 SV=1		5.30303	2	2	2	59.338	8.79	850000		410000	5.934638
Q8VH51	RNA-binding protein 39 OS=Mus musculus GN=Rbm39 PE=1 SV=2		16.22642	7	17	7	59.37	10.1	11000000	12000000	13000000	49.90477
Q9JKX4	Protein AATF OS=Mus musculus GN=Aatf PE=1 SV=1		4.372624	2	4	2	59.446	4.93	2400000	1300000	2600000	8.68712
O70145	Neutrophil cytosol factor 2 OS=Mus musculus GN=Ncf2 PE=1 SV=1		8	2	3	2	59.448	6.61	1600000	1200000		10.2223
P13645	SWISS-PROT:P13645 Tax_Id=9606 Gene_Symbol=KRT10 Keratin, type I cytoskeletal 10	x	44.01349	26	145	22	59.475	5.21	3.6E+08	1.2E+08	1.4E+09	451.9607
P42932	T-complex protein 1 subunit theta OS=Mus musculus GN=Cct8 PE=1 SV=3		36.67883	17	41	17	59.518	5.62	13000000	9400000	10000000	117.1262
Q8C0P5	Coronin-2A OS=Mus musculus GN=Coro2a PE=2 SV=1		4.580153	2	2	1	59.535	7.71			1000000	5.17326
Q8BT60	Copine-3 OS=Mus musculus GN=Cpne3 PE=1 SV=2		7.317073	4	6	2	59.547	5.78			1400000	14.30373
Q64133	Amine oxidase [flavin-containing] A OS=Mus musculus GN=Maoa PE=1 SV=3		14.8289	7	11	6	59.564	7.81	5200000	3700000	5800000	28.09283
Q923T9	Calcium/calmodulin-dependent protein kinase type II subunit gamma OS=Mus musculus GN=Camk2g PE=1 SV=1		10.20794	4	12	1	59.569	7.58	4600000	3300000	2300000	41.26781
P56564	Excitatory amino acid transporter 1 OS=Mus musculus GN=Slc1a3 PE=1 SV=2		12.70718	5	23	4	59.584	8.4	19000000	11000000	11000000	87.43222
P80316	T-complex protein 1 subunit epsilon OS=Mus musculus GN=Cct5 PE=1 SV=1		29.02033	13	35	13	59.586	6.02	16000000	14000000	12000000	95.88838
P80313	T-complex protein 1 subunit eta OS=Mus musculus GN=Cct7 PE=1 SV=1		23.52941	11	27	11	59.614	7.84	16000000	14000000	14000000	71.35174
Q99JF8	PC4 and SFRS1-interacting protein OS=Mus musculus GN=Psip1 PE=1 SV=1		26.13636	10	41	10	59.66	9.13	19000000	15000000	17000000	138.6125
Q8C5L3	CCR4-NOT transcription complex subunit 2 OS=Mus musculus GN=Cnot2 PE=1 SV=2		5.555556	2	4	2	59.673	7.66	2400000	1200000	1800000	11.78683
Q8BGZ7	Keratin, type II cytoskeletal 75 OS=Mus musculus GN=Krt75 PE=1 SV=1	x	15.24501	11	38	1	59.704	8.31			4200000	96.98369
Q8BI72	CDKN2A-interacting protein OS=Mus musculus GN=Cdkn2aip PE=1 SV=1		14.03197	5	12	5	59.708	9.16	1800000	2000000	1100000	42.10822
Q03265	ATP synthase subunit alpha, mitochondrial OS=Mus musculus GN=Atp5a1 PE=1 SV=1		35.44304	19	68	19	59.716	9.19	1.3E+08	95000000	98000000	212.1319
Q6ZQM8	UDP-glucuronosyltransferase 1-7C OS=Mus musculus GN=Ugt1a7c PE=1 SV=1		5.461394	2	4	2	59.719	8.35	1900000	2900000	1800000	12.18248
P24270	Catalase OS=Mus musculus GN=Cat PE=1 SV=4		5.12334	2	5	2	59.758	7.88	860000	970000	3100000	14.80088
Q99N16	Leukotriene-B(4) omega-hydroxylase 2 OS=Mus musculus GN=Cyp4f3 PE=1 SV=2		2.290076	1	2	1	59.804	8.53		1700000	1300000	4.548967
Q9CWP6	Motile sperm domain-containing protein 2 OS=Mus musculus GN=Mospd2 PE=1 SV=2		5.984556	2	5	2	59.817	5.67	3200000	2000000	2900000	17.8547
O35345	Importin subunit alpha-7 OS=Mus musculus GN=Kpna6 PE=1 SV=2		12.31343	4	12	2	59.926	4.94	820000	880000	1700000	40.10889
P04259	SWISS-PROT:P04259 Tax_Id=9606 Gene_Symbol=KRT6B Keratin, type II cytoskeletal 6B	x	39.1844	25	58	2	59.962	8	13000000		12000000	149.6746
P02538	SWISS-PROT:P02538 Tax_Id=9606 Gene_Symbol=KRT6A Keratin, type II cytoskeletal 6A	x	40.95745	26	60	3	60.008	8	3000000		12000000	154.6831
P36895	Bone morphogenetic protein receptor type-1A OS=Mus musculus GN=Bmpr1a PE=2 SV=1		2.819549	1	1	1	60.024	7.3	220000			2.62796
Q8BMP6	Golgi resident protein GCP60 OS=Mus musculus GN=Acbd3 PE=1 SV=3		15.2381	5	12	5	60.144	5.11	3500000	2600000	3800000	40.84406
Q60960	Importin subunit alpha-5 OS=Mus musculus GN=Kpna1 PE=1 SV=2		12.26766	4	10	2	60.144	5.01	1000000	1000000		33.40221
Q8CI33	CWF19-like protein 1 OS=Mus musculus GN=Cwf19l1 PE=1 SV=2		5.400372	2	4	2	60.156	7.14	1100000	1100000	980000	13.17545
Q04207	Transcription factor p65 OS=Mus musculus GN=Rela PE=1 SV=1		4.735883	2	3	2	60.174	5.76	870000	540000	440000	6.477543
Q80VP1	Epsin-1 OS=Mus musculus GN=Epn1 PE=1 SV=3		1.565217	1	1	1	60.175	4.81				2.127821
Q3UEB3	Poly(U)-binding-splicing factor PUF60 OS=Mus musculus GN=Puf60 PE=1 SV=2		19.85816	9	22	9	60.211	5.29	10000000	7600000	6400000	63.08702
Q8BYI6	Lysophosphatidylcholine acyltransferase 2 OS=Mus musculus GN=Lpcat2 PE=1 SV=1		11.02941	5	11	5	60.215	5.83	3300000	3300000	3100000	29.64895
O35144	Telomeric repeat-binding factor 2 OS=Mus musculus GN=Terf2 PE=1 SV=3		2.5878	1	1	1	60.226	9.5		1000000		2.929505
Q8VBW6	NEDD8-activating enzyme E1 regulatory subunit OS=Mus musculus GN=Nae1 PE=1 SV=1		3.183521	1	1	1	60.236	5.52	1200000			2.261897
O88939	Zinc finger and BTB domain-containing protein 7A OS=Mus musculus GN=Zbtb7a PE=1 SV=2		4.042179	1	1	1	60.243	5.12	420000			3.219591
Q9Z1W9	STE20/SPS1-related proline-alanine-rich protein kinase OS=Mus musculus GN=Stk39 PE=1 SV=1		4.496403	2	7	2	60.282	6.29	1900000	1600000	960000	18.36707
Q6DFW4	Nucleolar protein 58 OS=Mus musculus GN=Nop58 PE=1 SV=1		27.79851	12	41	12	60.305	8.34	11000000	15000000	14000000	141.1388
P36423	Thromboxane-A synthase OS=Mus musculus GN=Tbxas1 PE=1 SV=2		2.251407	1	1	1	60.352	7.15		510000		3.081873
Q71M36	Chondroitin sulfate proteoglycan 5 OS=Mus musculus GN=Cspg5 PE=1 SV=2		7.95053	3	9	3	60.368	4.54	3100000	2500000	1800000	27.64145
P11983	T-complex protein 1 subunit alpha OS=Mus musculus GN=Tcp1 PE=1 SV=3		24.64029	12	32	12	60.411	6.16	15000000	15000000	14000000	93.13641
P28652	Calcium/calmodulin-dependent protein kinase type II subunit beta OS=Mus musculus GN=Camk2b PE=1 SV=2		21.40221	9	21	5	60.423	7.28	6300000	4200000	3700000	69.55936
Q8K2T8	RNA polymerase II-associated factor 1 homolog OS=Mus musculus GN=Paf1 PE=1 SV=1		15.88785	6	12	6	60.481	4.65	2200000	2600000	2000000	36.71439
D0QMC3	Myeloid cell nuclear differentiation antigen-like protein OS=Mus musculus GN=Mndal PE=1 SV=1		24.53532	8	23	6	60.488	9.28	5800000	5300000	6800000	64.25607
Q64429	Cytochrome P450 1B1 OS=Mus musculus GN=Cyp1b1 PE=1 SV=3		2.209945	1	1	1	60.499	8.43		760000		2.527119
Q62523	Zyxin OS=Mus musculus GN=Zyx PE=1 SV=2		12.94326	5	10	5	60.507	6.4	3000000	3500000	1900000	32.3255
Q91YH5	Atlastin-3 OS=Mus musculus GN=Atl3 PE=1 SV=1		7.763401	3	7	3	60.537	6.1	1500000	4500000	5000000	24.5314
Q9WVK4	EH domain-containing protein 1 OS=Mus musculus GN=Ehd1 PE=1 SV=1		11.23596	5	13	4	60.565	6.83	5400000	5300000	3800000	38.35788
Q99LI2	Chloride channel CLIC-like protein 1 OS=Mus musculus GN=Clcc1 PE=1 SV=1		7.606679	3	4	3	60.582	5.68	970000	1300000		11.53957
P80318	T-complex protein 1 subunit gamma OS=Mus musculus GN=Cct3 PE=1 SV=1		35.22936	19	57	19	60.591	6.7	13000000	12000000	11000000	164.2267
P05480	Neuronal proto-oncogene tyrosine-protein kinase Src OS=Mus musculus GN=Src PE=1 SV=4		6.284658	3	6	2	60.606	7.84	2100000	1200000	2200000	15.81721
Q9EP89	Serine beta-lactamase-like protein LACTB, mitochondrial OS=Mus musculus GN=Lactb PE=1 SV=1		2.722323	1	2	1	60.667	8.9			600000	6.296052

P14685	26S proteasome non-ATPase regulatory subunit 3 OS=Mus musculus GN=Psmd3 PE=1 SV=3		23.20755	11	38	11	60.68	8.44	11000000	9500000	9400000	111.3258
O88643	Serine/threonine-protein kinase PAK 1 OS=Mus musculus GN=Pak1 PE=1 SV=1		13.94495	5	10	3	60.699	5.74	1800000	2800000	1800000	29.84752
Q8CI11	Guanine nucleotide-binding protein-like 3 OS=Mus musculus GN=Gnl3 PE=1 SV=2		13.3829	6	11	6	60.749	9.11	3900000	3700000	3800000	29.75474
Q9QXY6	EH domain-containing protein 3 OS=Mus musculus GN=Ehd3 PE=1 SV=2		13.08411	6	10	4	60.783	6.46	2500000	1900000		30.01419
Q60996	Serine/threonine-protein phosphatase 2A 56 kDa regulatory subunit gamma isoform OS=Mus musculus GN=Ppp2r5c PE=1 SV=2		2.480916	1	1	1	60.785	6.64		1100000		2.151478
Q3TEA8	Heterochromatin protein 1-binding protein 3 OS=Mus musculus GN=Hp1bp3 PE=1 SV=1		28.51986	13	34	13	60.829	9.7	13000000	15000000	15000000	90.60674
Q60715	Prolyl 4-hydroxylase subunit alpha-1 OS=Mus musculus GN=P4ha1 PE=1 SV=2		18.91386	8	24	8	60.872	5.9	6500000	5700000	5000000	74.62272
P63038	60 kDa heat shock protein, mitochondrial OS=Mus musculus GN=Hspd1 PE=1 SV=1		40.48866	22	69	22	60.917	6.18	42000000	51000000	29000000	228.2064
Q3UHD6	Sorting nexin-27 OS=Mus musculus GN=Snx27 PE=1 SV=2		8.90538	5	7	5	60.95	6.4	1600000	1600000	1200000	18.22071
Q60716	Prolyl 4-hydroxylase subunit alpha-2 OS=Mus musculus GN=P4ha2 PE=1 SV=1		2.607076	1	3	1	60.964	5.8	1200000	1300000	970000	8.147555
Q9D281	Protein Noxp20 OS=Mus musculus GN=Fam114a1 PE=1 SV=1		2.636204	1	2	1	60.975	4.56	740000		590000	4.900565
P59108	Copine-2 OS=Mus musculus GN=Cpne2 PE=1 SV=1		3.10219	2	2	1	60.997	5.96		460000		3.993275
Q99J27	Acetyl-coenzyme A transporter 1 OS=Mus musculus GN=Slc33a1 PE=1 SV=1		4.545455	2	2	2	61.036	8.16		130000		4.020675
P20060	Beta-hexosaminidase subunit beta OS=Mus musculus GN=Hexb PE=1 SV=2		15.67164	7	14	7	61.077	8.12	3600000	5700000	2600000	33.55567
Q8BH64	EH domain-containing protein 2 OS=Mus musculus GN=Ehd2 PE=1 SV=1		2.025783	1	1	1	61.136	6.51		470000		1.961124
Q8BFR4	N-acetylglucosamine-6-sulfatase OS=Mus musculus GN=Gns PE=1 SV=1		1.286765	1	1	1	61.136	8.24				1.934239
Q3UV70	[Pyruvate dehydrogenase [acetyl-transferring]]-phosphatase 1, mitochondrial OS=Mus musculus GN=Pdp1 PE=1 SV=1		2.973978	1	2	1	61.142	6.67	1600000		1000000	6.489214
Q9D0R4	Probable ATP-dependent RNA helicase DDX56 OS=Mus musculus GN=Ddx56 PE=2 SV=1		2.564103	1	4	1	61.174	9.17				13.09639
Q5SSI6	U3 small nucleolar RNA-associated protein 18 homolog OS=Mus musculus GN=Utp18 PE=1 SV=1		4.710145	2	4	2	61.18	8.78	2100000	2000000	3000000	15.53541
Q64516	Glycerol kinase OS=Mus musculus GN=Gk PE=1 SV=2		1.431127	1	1	1	61.188	5.87			960000	2.521912
Q60598	Src substrate cortactin OS=Mus musculus GN=Cttn PE=1 SV=2		23.80952	9	17	9	61.212	5.4	5400000	6800000	5000000	44.31494
E9QAG8	Zinc finger protein 431 OS=Mus musculus GN=Znf431 PE=1 SV=1		1.520913	1	1	1	61.241	9.03			600000	2.217375
P26443	Glutamate dehydrogenase 1, mitochondrial OS=Mus musculus GN=Glud1 PE=1 SV=1		40.14337	18	69	18	61.298	8	49000000	44000000	42000000	206.5587
Q8BIQ5	Cleavage stimulation factor subunit 2 OS=Mus musculus GN=Cstf2 PE=1 SV=2		16.2069	6	10	6	61.302	6.83	1600000	1700000	2700000	28.79877
Q3ULD5	Methylcrotonoyl-CoA carboxylase beta chain, mitochondrial OS=Mus musculus GN=Mccc2 PE=1 SV=1		3.552398	1	1	1	61.34	8			590000	3.517359
Q9D0F9	Phosphoglucomutase-1 OS=Mus musculus GN=Pgm1 PE=1 SV=4		4.270463	2	4	2	61.38	6.57	1600000	750000	1400000	11.92134
Q61207	Prosaposin OS=Mus musculus GN=Psap PE=1 SV=2		12.56732	7	17	7	61.381	5.19	9700000	9500000	7400000	42.14961
Q8VC31	Coiled-coil domain-containing protein 9 OS=Mus musculus GN=Ccdc9 PE=2 SV=1		2.578269	1	2	1	61.408	5	570000		100000	4.689723
O88447	Kinesin light chain 1 OS=Mus musculus GN=Klc1 PE=1 SV=3		11.27542	5	9	5	61.412	5.68	2600000	1800000	1900000	26.42505
Q9CSN1	SNW domain-containing protein 1 OS=Mus musculus GN=Snw1 PE=1 SV=3		15.48507	6	14	6	61.438	9.48	4400000	4400000	3600000	39.38284
Q9EQP2	EH domain-containing protein 4 OS=Mus musculus GN=Ehd4 PE=1 SV=1		16.63586	7	14	6	61.441	6.76	4600000	5000000	3800000	43.95088
P08551	Neurofilament light polypeptide OS=Mus musculus GN=Nefl PE=1 SV=5		35.17495	18	49	17	61.471	4.64	16000000	7100000	8400000	136.7804
Q8R5H6	Wiskott-Aldrich syndrome protein family member 1 OS=Mus musculus GN=Wasf1 PE=1 SV=2		2.146691	1	2	1	61.471	6.37	2800000	1400000		6.71451
Q9EQF6	Dihydropyrimidinase-related protein 5 OS=Mus musculus GN=Dpysl5 PE=1 SV=1		4.964539	2	3	2	61.477	7.09	1600000			6.476785
Q6A0D4	Raftlin OS=Mus musculus GN=Rftn1 PE=1 SV=4		2.707581	1	1	1	61.499	7.68			950000	2.646146
Q9WV34	MAGUK p55 subfamily member 2 OS=Mus musculus GN=Mpp2 PE=1 SV=1		1.811594	1	1	1	61.517	6.44	730000			2.750991
Q3TXX4	Vesicular glutamate transporter 1 OS=Mus musculus GN=Slc17a7 PE=1 SV=2		8.035714	4	7	4	61.597	7.34	6600000	3200000	4900000	17.0481
Q91WJ7	SPATS2-like protein OS=Mus musculus GN=Spats2l PE=2 SV=2		1.792115	1	3	1	61.631	9.69	840000	970000	1000000	8.683054
Q8R3Y8	Interferon regulatory factor 2-binding protein 1 OS=Mus musculus GN=Irf2bp1 PE=1 SV=2		2.568493	1	1	1	61.712	8.18		170000		2.042948
Q8BMC3	SHC-transforming protein 2 OS=Mus musculus GN=Shc2 PE=1 SV=3		2.966841	1	1	1	61.732	6.7		380000		2.130631
Q7Z794	SWISS-PROT:Q7Z794 Tax_Id=9606 Gene_Symbol=KRT77 Keratin 77	x	24.56747	14	30	10	61.764	5.85			13000000	85.63704
Q5U4E2	Replication initiator 1 OS=Mus musculus GN=Repin1 PE=1 SV=1		2.018349	1	1	1	61.79	9.91		260000		2.150865
Q9DBA9	General transcription factor IIH subunit 1 OS=Mus musculus GN=Gtf2h1 PE=1 SV=2		3.107861	1	1	1	61.812	8.76		250000		3.389296
Q9JLV1	BAG family molecular chaperone regulator 3 OS=Mus musculus GN=Bag3 PE=1 SV=2		6.759099	3	4	3	61.822	7.27		1000000	1300000	11.45447
Q5SU73	Coilin OS=Mus musculus GN=Coil PE=1 SV=1		5.263158	2	7	2	61.879	9	850000	510000	550000	17.88537
Q62188	Dihydropyrimidinase-related protein 3 OS=Mus musculus GN=Dpysl3 PE=1 SV=1		18.24561	8	23	5	61.897	6.49	2600000	1900000	2100000	75.30193
O35098	Dihydropyrimidinase-related protein 4 OS=Mus musculus GN=Dpysl4 PE=1 SV=1		7.867133	3	6	2	61.922	6.98	1600000	1100000		19.56303
Q8R317	Ubiquilin-1 OS=Mus musculus GN=Ubqln1 PE=1 SV=1		2.57732	1	3	1	61.937	4.94	1600000	1600000	1600000	7.616127
Q91VU6	DDB1- and CUL4-associated factor 11 OS=Mus musculus GN=Dcaf11 PE=1 SV=1		3.278689	1	3	1	61.953	6.44	300000	310000		8.605833
P06683	Complement component C9 OS=Mus musculus GN=C9 PE=1 SV=2		2.189781	1	2	1	61.962	5.78	700000		400000	4.782071
P43006	Excitatory amino acid transporter 2 OS=Mus musculus GN=Slc1a2 PE=1 SV=1		14.68531	8	26	8	61.99	6.68	38000000	19000000	16000000	75.73976
Q8BUV8	Protein GPR107 OS=Mus musculus GN=Gpr107 PE=1 SV=2		1.633394	1	1	1	62.016	7.59		730000		1.849473
P35527	SWISS-PROT:P35527 Tax_Id=9606 Gene_Symbol=KRT9 Keratin, type I cytoskeletal 9	x	49.91974	23	152	22	62.092	5.3	3.3E+08	44000000	7.5E+08	511.7361

Q80VL1	Tudor and KH domain-containing protein OS=Mus musculus GN=Tdrkh PE=1 SV=1		7.857143	3	7	3	62.095	4.94	1000000	750000	1000000	19.72142
P97427	Dihydropyrimidinase-related protein 1 OS=Mus musculus GN=Crmp1 PE=1 SV=1		16.78322	7	25	3	62.129	7.12	6000000	3300000	3000000	86.4008
Q8BXA5	Cleft lip and palate transmembrane protein 1-like protein OS=Mus musculus GN=Clptm1l PE=1 SV=1		4.823748	2	4	2	62.143	8.84	2000000	2500000	2900000	11.82993
Q6DID7	Protein wntless homolog OS=Mus musculus GN=Wls PE=1 SV=1		3.512015	2	4	2	62.147	7.65	1800000	1500000	2000000	9.73711
O08553	Dihydropyrimidinase-related protein 2 OS=Mus musculus GN=Dpysl2 PE=1 SV=2		49.12587	19	90	15	62.239	6.38	72000000	48000000	52000000	300.3316
Q91YT7	YTH domain-containing family protein 2 OS=Mus musculus GN=Ythdf2 PE=1 SV=1		3.972366	2	6	1	62.242	8.79	4400000		3000000	17.52973
Q9CQG2	Methyltransferase-like protein 16 OS=Mus musculus GN=Mettl16 PE=1 SV=1		2.169982	1	2	1	62.301	7.36	740000		820000	5.142928
Q6PAM1	Alpha-taxilin OS=Mus musculus GN=Txlna PE=1 SV=1		23.10469	8	17	8	62.33	6.74	2600000	2900000	2500000	52.55897
P13647	SWISS-PROT:P13647 Tax_Id=9606 Gene_Symbol=KRT5 Keratin, type II cytoskeletal 5	x	41.01695	27	82	14	62.34	7.74	26000000	6800000	1.3E+08	220.912
Q9CZX0	Elongator complex protein 3 OS=Mus musculus GN=Elp3 PE=1 SV=1		2.559415	1	3	1	62.345	8.9	11000000		300000	8.632321
P34914	Bifunctional epoxide hydrolase 2 OS=Mus musculus GN=Ephx2 PE=1 SV=2		3.429603	1	2	1	62.475	6.19	300000		170000	4.54978
Q60864	Stress-induced-phosphoprotein 1 OS=Mus musculus GN=Stip1 PE=1 SV=1		24.49355	11	34	11	62.542	6.8	12000000	13000000	10000000	100.4123
Q3U0K8	Prolyl 3-hydroxylase OGFOD1 OS=Mus musculus GN=Ogfod1 PE=1 SV=1		3.302752	1	3	1	62.695	4.91	590000	610000	410000	10.36647
P03975	IgE-binding protein OS=Mus musculus GN=Iap PE=2 SV=1		10.23339	4	16	4	62.708	9.31	27000000	31000000	30000000	52.73227
P06745	Glucose-6-phosphate isomerase OS=Mus musculus GN=Gpi PE=1 SV=4		16.84588	8	25	8	62.727	8.13	11000000	9900000	7600000	73.0092
Q99PN3	Tripartite motif-containing protein 26 OS=Mus musculus GN=Trim26 PE=2 SV=3		2.018349	1	1	1	62.769	5.05	430000			2.456306
Q61211	Eukaryotic translation initiation factor 2D OS=Mus musculus GN=Eif2d PE=1 SV=3		8.421053	3	7	3	62.79	8.24	2500000	2100000	2100000	23.10499
Q61508	Extracellular matrix protein 1 OS=Mus musculus GN=Ecm1 PE=1 SV=2		7.692308	3	8	3	62.792	6.8	2000000	1500000	970000	22.92065
Q3UV17	Keratin, type II cytoskeletal 2 oral OS=Mus musculus GN=Krt76 PE=1 SV=1		8.754209	7	22	1	62.806	8.43	5.1E+08	97000000	1.6E+09	52.21707
Q8VCM8	Nicalin OS=Mus musculus GN=Ncln PE=1 SV=2		6.039076	3	5	3	62.868	6.49	2200000	2000000		12.49263
Q91WG5	5'-AMP-activated protein kinase subunit gamma-2 OS=Mus musculus GN=Prkag2 PE=1 SV=2		1.766784	1	1	1	62.91	9.36		1100000		2.259486
P97363	Serine palmitoyltransferase 2 OS=Mus musculus GN=Sptlc2 PE=1 SV=2		2.142857	1	1	1	62.941	8.18	1500000			2.789302
Q8BRG8	Transmembrane protein 209 OS=Mus musculus GN=Tmem209 PE=2 SV=1		1.782531	1	2	1	62.942	8.73		480000	270000	4.218041
Q9WVM3	Anaphase-promoting complex subunit 7 OS=Mus musculus GN=Anapc7 PE=1 SV=3		2.123894	1	1	1	62.981	5.72			630000	2.145911
Q8VEG6	CCR4-NOT transcription complex subunit 6-like OS=Mus musculus GN=Cnot6l PE=1 SV=2		2.522523	1	1	1	62.983	6.57				2.049494
Q9Z1Q2	Protein ABHD16A OS=Mus musculus GN=Abhd16a PE=1 SV=3		7.706093	4	7	4	63.046	8.25	1500000	2000000	2100000	16.81097
Q3UHB1	5'-nucleotidase domain-containing protein 3 OS=Mus musculus GN=Nt5dc3 PE=1 SV=1		2.197802	1	2	1	63.13	8.56	1300000		970000	5.473676
O08914	Fatty-acid amide hydrolase 1 OS=Mus musculus GN=Faah PE=1 SV=1		1.554404	1	1	1	63.18	7.87	350000			2.276165
Q5U458	DnaJ homolog subfamily C member 11 OS=Mus musculus GN=Dnajc11 PE=1 SV=2		2.146691	1	1	1	63.194	8.32			910000	2.568801
Q3UUG6	TBC1 domain family member 24 OS=Mus musculus GN=Tbc1d24 PE=1 SV=2		4.634581	2	3	2	63.195	7.24	1100000	670000		7.841077
Q8C854	Myelin expression factor 2 OS=Mus musculus GN=Myef2 PE=1 SV=1		13.36717	8	18	8	63.254	8.87	8000000	7100000	6400000	57.83409
Q8CI51	PDZ and LIM domain protein 5 OS=Mus musculus GN=Pdlim5 PE=1 SV=4		15.56684	6	18	6	63.259	8.25	5100000	4300000	2400000	58.461
Q64332	Synapsin-2 OS=Mus musculus GN=Syn2 PE=1 SV=2		17.23549	7	18	6	63.333	8.43	6900000	3200000	3000000	60.95357
Q8BH66	Atlastin-1 OS=Mus musculus GN=Atl1 PE=1 SV=1		2.508961	1	2	1	63.337	6.49	2700000	1400000		7.287272
Q8BXN9	Transmembrane protein 87A OS=Mus musculus GN=Tmem87a PE=1 SV=1		7.027027	3	6	3	63.339	6.67	4400000	3100000	2100000	17.869
P28798	Granulins OS=Mus musculus GN=Grn PE=1 SV=2		9.50764	4	8	4	63.413	6.8	1200000	2200000	990000	20.31039
Q8CHU3	Epsin-2 OS=Mus musculus GN=Epn2 PE=1 SV=1		4.369748	2	3	2	63.433	8.16	1400000		640000	8.475637
P46061	Ran GTPase-activating protein 1 OS=Mus musculus GN=Rangap1 PE=1 SV=2		18.16638	10	18	10	63.491	4.68	4700000	6400000	6800000	54.33163
Q9JHW4	Selenocysteine-specific elongation factor OS=Mus musculus GN=Eefsec PE=1 SV=2		2.744425	1	2	1	63.498	8.29	850000		830000	6.061346
Q9CPN8	Insulin-like growth factor 2 mRNA-binding protein 3 OS=Mus musculus GN=Igf2bp3 PE=1 SV=1		13.29879	5	11	4	63.535	8.87	1800000	1200000	1900000	30.74994
Q505F5	Leucine-rich repeat-containing protein 47 OS=Mus musculus GN=Lrrc47 PE=1 SV=1		8.261618	4	8	4	63.551	8.1	3600000	3100000	2300000	22.54792
Q9JIA1	Leucine-rich glioma-inactivated protein 1 OS=Mus musculus GN=Lgi1 PE=1 SV=1		3.949731	2	4	2	63.603	8.02	1100000	600000		9.485981
Q99P91	Transmembrane glycoprotein NMB OS=Mus musculus GN=Gpnmb PE=1 SV=2		3.484321	2	4	2	63.635	7.61	2400000	2400000	3600000	11.00407
Q8R0X7	Sphingosine-1-phosphate lyase 1 OS=Mus musculus GN=Sgpl1 PE=1 SV=1		14.78873	7	19	7	63.636	9.1	12000000	9800000	9800000	58.81057
Q8BMK4	Cytoskeleton-associated protein 4 OS=Mus musculus GN=Ckap4 PE=1 SV=2		43.65217	23	75	23	63.654	5.64	23000000	23000000	21000000	238.5036
Q80SY5	Pre-mRNA-splicing factor 38B OS=Mus musculus GN=Prpf38b PE=1 SV=1		2.214022	1	3	1	63.715	10.54	3000000	2800000	2700000	8.82166
Q61263	Sterol O-acyltransferase 1 OS=Mus musculus GN=Soat1 PE=1 SV=2		2.962963	1	1	1	63.757	9.01			800000	3.188209
Q5NCR9	Nuclear speckle splicing regulatory protein 1 OS=Mus musculus GN=Nsrp1 PE=1 SV=1		2.398524	1	3	1	63.761	8.76	1300000	1100000	1300000	7.115392
Q80WJ7	Protein LYRIC OS=Mus musculus GN=Mtdh PE=1 SV=1		32.8152	17	52	17	63.808	9.33	13000000	14000000	13000000	149.6445
Q61712	DnaJ homolog subfamily C member 1 OS=Mus musculus GN=Dnajc1 PE=1 SV=1		4.166667	2	4	2	63.83	8.97	750000	1100000	1100000	11.26485
Q4VBD2	Transmembrane anterior posterior transformation protein 1 OS=Mus musculus GN=Tapt1 PE=1 SV=2		1.77305	1	1	1	63.853	8.22		1500000		2.59529
Q69Z99	Zinc finger protein 512 OS=Mus musculus GN=Znf512 PE=2 SV=2		14.59075	5	8	5	63.867	9.51	640000	2600000	1800000	24.00371
Q5EG47	5'-AMP-activated protein kinase catalytic subunit alpha-1 OS=Mus musculus GN=Prkaa1 PE=1 SV=2		3.041145	1	3	1	63.889	8.12	1400000	1300000	830000	7.771606

P06801	NADP-dependent malic enzyme OS=Mus musculus GN=Me1 PE=1 SV=2		8.041958	5	11	5	63.913	7.44	3700000	2400000	2600000	27.11506
Q8BYK6	YTH domain-containing family protein 3 OS=Mus musculus GN=Ythdf3 PE=1 SV=2		4.273504	2	6	1	63.922	9.04	1700000	1600000	1800000	15.25472
Q8R081	Heterogeneous nuclear ribonucleoprotein L OS=Mus musculus GN=Hnrnpl PE=1 SV=2		40.78498	16	63	16	63.923	8.1	65000000	56000000	56000000	215.8132
O70194	Eukaryotic translation initiation factor 3 subunit D OS=Mus musculus GN=Eif3d PE=1 SV=2		25.36496	11	32	11	63.948	6.05	13000000	12000000	12000000	99.52233
Q9Z1K5	E3 ubiquitin-protein ligase ARIH1 OS=Mus musculus GN=Arih1 PE=1 SV=3		2.522523	1	3	1	63.975	5.08		2600000	1700000	9.350566
Q921F4	Heterogeneous nuclear ribonucleoprotein L-like OS=Mus musculus GN=Hnrnpll PE=1 SV=3		21.15059	8	22	8	64.084	5.85	6400000	6000000	6000000	75.30607
Q9CWJ9	Bifunctional purine biosynthesis protein PURH OS=Mus musculus GN=Atic PE=1 SV=2		12.33108	4	6	4	64.177	6.76	3200000	1100000		19.1753
O08912	Polypeptide N-acetylgalactosaminyltransferase 1 OS=Mus musculus GN=Galnt1 PE=1 SV=1		2.504472	1	1	1	64.213	7.71	1000000			2.228726
Q8BP47	Asparagine--tRNA ligase, cytoplasmic OS=Mus musculus GN=Nars PE=1 SV=2		14.84794	6	16	6	64.238	5.86	6900000	7300000	6500000	48.33369
Q61024	Asparagine synthetase [glutamine-hydrolyzing] OS=Mus musculus GN=Asns PE=1 SV=3		3.743316	2	2	2	64.241	6.58		2000000		4.443497
Q3UDK1	TRAF-type zinc finger domain-containing protein 1 OS=Mus musculus GN=Trafd1 PE=1 SV=1		3.62069	1	3	1	64.242	5.48	1900000	2000000	1700000	10.48855
Q9EP97	Sentrin-specific protease 3 OS=Mus musculus GN=Senp3 PE=1 SV=1		5.633803	2	5	2	64.362	8.75	1300000	1700000	1500000	16.33182
Q8BJW6	Eukaryotic translation initiation factor 2A OS=Mus musculus GN=Eif2a PE=1 SV=2		9.466437	4	7	4	64.363	8.91	2000000	4700000	1700000	19.46792
Q9D6Z1	Nucleolar protein 56 OS=Mus musculus GN=Nop56 PE=1 SV=2		33.62069	17	34	17	64.424	9.14	21000000	23000000	22000000	100.9763
O08539	Myc box-dependent-interacting protein 1 OS=Mus musculus GN=Bin1 PE=1 SV=1		16.83673	7	14	6	64.43	5.03	7700000	5600000	5200000	44.5956
Q8VI36	Paxillin OS=Mus musculus GN=Pxn PE=1 SV=1		4.399323	2	3	2	64.435	6.02	650000	710000		7.573042
Q07797	Galectin-3-binding protein OS=Mus musculus GN=Lgals3bp PE=1 SV=1		18.37088	7	17	7	64.45	5.14	7500000	6100000	6100000	58.51624
Q6PB93	Polypeptide N-acetylgalactosaminyltransferase 2 OS=Mus musculus GN=Galnt2 PE=1 SV=1		11.92982	6	11	6	64.473	8.53	1800000	2800000	3200000	31.25411
Q61481	Calcium/calmodulin-dependent 3',5'-cyclic nucleotide phosphodiesterase 1A OS=Mus musculus GN=Pde1a PE=1 SV=2		1.946903	1	3	1	64.487	5.97	790000	510000	610000	7.484131
Q61576	Peptidyl-prolyl cis-trans isomerase FKBP10 OS=Mus musculus GN=Fkbp10 PE=1 SV=2		7.57315	3	5	3	64.656	5.64	2500000	1900000	1900000	13.62472
Q05CL8	La-related protein 7 OS=Mus musculus GN=Larp7 PE=1 SV=2		10.87719	5	10	5	64.762	9.54	480000	490000	1100000	26.30124
Q921I2	Kelch domain-containing protein 4 OS=Mus musculus GN=Klhdc4 PE=2 SV=2		6.678082	2	2	2	64.82	5.73	690000	160000		5.485547
Q9D824	Pre-mRNA 3'-end-processing factor FIP1 OS=Mus musculus GN=Fip1l1 PE=1 SV=1		7.745267	3	5	3	64.92	5.77		2100000	2200000	12.04343
Q9CWL8	Beta-catenin-like protein 1 OS=Mus musculus GN=Ctnnbl1 PE=1 SV=1		9.236234	4	13	4	64.939	5.07	2900000	2900000	2100000	47.43748
Q9JHK4	Geranylgeranyl transferase type-2 subunit alpha OS=Mus musculus GN=Rabggta PE=1 SV=1		1.763668	1	1	1	64.948	5.8	1000000			2.088521
Q8R366	Immunoglobulin superfamily member 8 OS=Mus musculus GN=Igsf8 PE=1 SV=2		7.855974	3	6	3	64.97	7.99	1200000	570000	650000	17.19337
P97390	Vacuolar protein sorting-associated protein 45 OS=Mus musculus GN=Vps45 PE=1 SV=1		4.736842	3	6	3	65.012	8.25	1500000	1500000	1.3E+08	14.25879
Q61490	CD166 antigen OS=Mus musculus GN=Alcam PE=1 SV=3		4.631218	2	2	2	65.051	6.15	860000			5.333592
Q3TIX9	U4/U6.U5 tri-snRNP-associated protein 2 OS=Mus musculus GN=Usp39 PE=1 SV=2		4.964539	3	5	3	65.106	8.9	2600000	2300000	3300000	11.29081
Q8BLH7	HIRA-interacting protein 3 OS=Mus musculus GN=Hirip3 PE=1 SV=1		6.821963	4	7	4	65.176	7.84	410000	980000	590000	23.86645
O88291	DBIRD complex subunit ZNF326 OS=Mus musculus GN=Znf326 PE=1 SV=1		12.06897	5	11	5	65.185	5.19	6600000	5000000	6700000	31.62796
Q8BX57	PX domain-containing protein kinase-like protein OS=Mus musculus GN=Pxk PE=1 SV=2		2.061856	1	3	1	65.19	9.38	1000000	1200000	1200000	8.893564
P55194	SH3 domain-binding protein 1 OS=Mus musculus GN=Sh3bp1 PE=1 SV=2		9.650582	4	13	4	65.244	5.68	1500000	1600000	1800000	38.11757
Q61093	Cytochrome b-245 heavy chain OS=Mus musculus GN=Cybb PE=1 SV=1		9.649123	4	13	4	65.262	7.72	6700000	7100000	4400000	40.83404
Q91W39	Nuclear receptor coactivator 5 OS=Mus musculus GN=Ncoa5 PE=1 SV=1		10.18998	3	5	3	65.28	9.82	1400000	1900000	1400000	13.42912
Q76MZ3	Serine/threonine-protein phosphatase 2A 65 kDa regulatory subunit A alpha isoform OS=Mus musculus GN=Ppp2r1a PE=1 SV=3		13.24278	6	17	6	65.281	5.11	11000000	13000000	9100000	50.65027
Q07643	Collagen alpha-2(IX) chain OS=Mus musculus GN=Col9a2 PE=2 SV=1		3.924419	2	9	2	65.281	9.36	5400000	4600000	4300000	27.65471
Q9DC48	Pre-mRNA-processing factor 17 OS=Mus musculus GN=Cdc40 PE=2 SV=1		6.908463	3	5	3	65.42	7.06	810000	1400000	500000	13.15711
Q80UG5	Septin-9 OS=Mus musculus GN=Sept9 PE=1 SV=1		27.61578	13	37	13	65.534	8.9	12000000	14000000	9600000	114.9744
Q5SF07	Insulin-like growth factor 2 mRNA-binding protein 2 OS=Mus musculus GN=Igf2bp2 PE=1 SV=1		10.13514	4	8	3	65.543	8.03	2200000	1200000	3900000	21.65128
Q9Z1M8	Protein Red OS=Mus musculus GN=Ik PE=1 SV=2		10.41293	5	11	5	65.576	6.64	2100000	2700000	2600000	27.38992
Q8BQ30	Phostensin OS=Mus musculus GN=Ppp1r18 PE=1 SV=1		9.59596	4	8	4	65.59	5.39	1900000	2500000	1700000	24.63408
Q8C4Y3	Negative elongation factor B OS=Mus musculus GN=Nelfb PE=1 SV=2		4.137931	2	2	2	65.594	6.02		1100000	1200000	5.308868
Q9WUA2	Phenylalanine--tRNA ligase beta subunit OS=Mus musculus GN=Farsb PE=1 SV=2		11.03565	6	16	6	65.655	7.12	3800000	2600000	2800000	43.04067
Q8BXQ2	GPI transamidase component PIG-T OS=Mus musculus GN=Pigt PE=1 SV=2		2.061856	1	1	1	65.663	8.4			6500000	3.502191
Q61730	Interleukin-1 receptor accessory protein OS=Mus musculus GN=Il1rap PE=1 SV=1		3.333333	2	3	2	65.699	7.77	1700000		1300000	6.124537
Q99KE1	NAD-dependent malic enzyme, mitochondrial OS=Mus musculus GN=Me2 PE=1 SV=1		2.546689	1	2	1	65.757	7.61	1500000	1200000		6.572536
P35908	SWISS-PROT:P35908 Tax_Id=9606 Gene_Symbol=KRT2 Keratin, type II cytoskeletal 2 epidermal	x	56.27907	36	161	26	65.825	8	1.9E+08	32000000	6.3E+08	499.0657
Q8BFW7	Lipoma-preferred partner homolog OS=Mus musculus GN=Lpp PE=1 SV=1		10.27732	4	7	4	65.848	7.37	2400000	2400000	790000	18.71028
Q60862	Origin recognition complex subunit 2 OS=Mus musculus GN=Orc2 PE=1 SV=1		2.256944	1	1	1	65.853	6.81		770000		3.061909
Q8BU11	TOX high mobility group box family member 4 OS=Mus musculus GN=Tox4 PE=1 SV=3		3.877221	2	3	2	65.92	5.01	1200000		1200000	8.015034
O70293	G protein-coupled receptor kinase 6 OS=Mus musculus GN=Grk6 PE=1 SV=1		2.430556	1	2	1	65.936	8.1	1700000	1200000		5.263599
P04264	SWISS-PROT:P04264 Tax_Id=9606 Gene_Symbol=KRT1 Keratin, type II cytoskeletal 1	x	54.50311	39	217	31	65.978	8.12	6.6E+08	1.1E+08	2.2E+09	634.3299

Q8N7N5	DDB1- and CUL4-associated factor 8 OS=Mus musculus GN=Dcaf8 PE=1 SV=1		7.445008	3	8	3	65.991	5.87	520000	800000	440000	21.12116
Q9QXN3	Activating signal cointegrator 1 OS=Mus musculus GN=Trip4 PE=1 SV=2		1.72117	1	1	1	66.156	7.55		730000		2.389147
Q60775	ETS-related transcription factor Elf-1 OS=Mus musculus GN=Elf1 PE=1 SV=1		2.124183	1	3	1	66.18	4.97	1200000	1100000	750000	8.989172
Q64324	Syntaxin-binding protein 2 OS=Mus musculus GN=Stxbp2 PE=1 SV=1		8.094435	3	6	3	66.315	6.74	1800000	790000	760000	16.81947
O88342	WD repeat-containing protein 1 OS=Mus musculus GN=Wdr1 PE=1 SV=3		24.25743	10	25	10	66.365	6.6	10000000	9800000	8400000	71.61643
Q91VH2	Sorting nexin-9 OS=Mus musculus GN=Snx9 PE=1 SV=1		3.865546	2	2	2	66.504	5.52	430000		1400000	4.670436
O08832	Polypeptide N-acetylgalactosaminyltransferase 4 OS=Mus musculus GN=Galnt4 PE=2 SV=1		1.903114	1	1	1	66.513	7.37	930000			2.285153
Q8QZY1	Eukaryotic translation initiation factor 3 subunit L OS=Mus musculus GN=Eif3l PE=1 SV=1		22.69504	10	25	10	66.57	6.44	19000000	21000000	9500000	79.44798
P48318	Glutamate decarboxylase 1 OS=Mus musculus GN=Gad1 PE=1 SV=2		4.047218	2	2	2	66.605	7.17	960000			4.313135
O88448	Kinesin light chain 2 OS=Mus musculus GN=Klc2 PE=1 SV=1		5.008347	2	2	2	66.621	7.21			550000	4.903059
P70698	CTP synthase 1 OS=Mus musculus GN=Ctps1 PE=1 SV=2		10.99831	5	10	5	66.64	6.58	2700000	3400000	2600000	28.27939
Q6PIP5	NudC domain-containing protein 1 OS=Mus musculus GN=Nudcd1 PE=1 SV=2		1.890034	1	1	1	66.662	5.3		1400000		3.00513
Q9Z0E6	Guanylate-binding protein 1 OS=Mus musculus GN=Gbp2 PE=1 SV=1		21.73175	11	46	11	66.697	5.71	78000000	81000000	64000000	158.593
Q925I1	ATPase family AAA domain-containing protein 3 OS=Mus musculus GN=Atad3 PE=1 SV=1		8.798646	4	7	4	66.701	9.29	3400000	1900000	1200000	16.14456
Q9Z0X1	Apoptosis-inducing factor 1, mitochondrial OS=Mus musculus GN=Aifm1 PE=1 SV=1		14.05229	6	14	6	66.724	9.17	4000000	3400000	4200000	42.81475
Q9JLZ6	Hypermethylated in cancer 2 protein OS=Mus musculus GN=Hic2 PE=2 SV=4		1.615509	1	3	1	66.724	6.35	5000000	3100000	3600000	8.689398
Q921T2	Torsin-1A-interacting protein 1 OS=Mus musculus GN=Tor1aip1 PE=1 SV=3		24.53782	9	24	9	66.74	7.05	9700000	7800000	9400000	76.95042
P14733	Lamin-B1 OS=Mus musculus GN=Lmnb1 PE=1 SV=3		58.67347	36	151	32	66.745	5.16	48000000	46000000	49000000	446.4928
Q68FL4	Putative adenosylhomocysteinase 3 OS=Mus musculus GN=Ahcyl2 PE=1 SV=1		18.43393	10	22	1	66.857	7.36	2900000	2200000		56.99337
Q8CFB4	Guanylate-binding protein 5 OS=Mus musculus GN=Gbp5 PE=1 SV=2		17.79661	10	27	10	66.888	6.09	20000000	15000000	15000000	76.49922
Q9EP69	Phosphatidylinositide phosphatase SAC1 OS=Mus musculus GN=Sacm1l PE=1 SV=1		4.258944	3	5	3	66.901	7.3	1600000	3300000	2600000	10.64768
Q8VCW4	Protein unc-93 homolog B1 OS=Mus musculus GN=Unc93b1 PE=1 SV=2		2.842809	1	3	1	66.937	6.42	3400000	2800000	2100000	11.26072
P31648	Sodium- and chloride-dependent GABA transporter 1 OS=Mus musculus GN=Slc6a1 PE=1 SV=2		1.669449	1	3	1	66.957	7.96	3000000	1400000	1500000	7.898378
Q924K8	Metastasis-associated protein MTA3 OS=Mus musculus GN=Mta3 PE=1 SV=1		2.876481	1	3	1	67.035	8.88	3300000	3300000	2400000	11.85294
Q9JMH6	Thioredoxin reductase 1, cytoplasmic OS=Mus musculus GN=Txnrd1 PE=1 SV=3		1.794454	1	3	1	67.042	7.44	2900000	2600000	1800000	9.096159
Q6VN19	Ran-binding protein 10 OS=Mus musculus GN=Ranbp10 PE=1 SV=2		2.741935	1	1	1	67.146	6.58		470000		2.320061
P35564	Calnexin OS=Mus musculus GN=Canx PE=1 SV=1		37.39425	21	85	21	67.236	4.64	1.5E+08	1.2E+08	1.2E+08	265.4151
P61222	ATP-binding cassette sub-family E member 1 OS=Mus musculus GN=Abce1 PE=1 SV=1		9.18197	5	14	5	67.271	8.34	5700000	6100000	7100000	39.67107
P21619	Lamin-B2 OS=Mus musculus GN=Lmnb2 PE=1 SV=2		30.36913	18	47	14	67.277	5.5	12000000	11000000	11000000	137.0585
Q8CHY6	Transcriptional repressor p66 alpha OS=Mus musculus GN=Gatad2a PE=1 SV=2		7.154213	3	10	3	67.292	9.89	1800000	1800000	1700000	28.76851
Q8CE64	Zinc finger protein 276 OS=Mus musculus GN=Znf276 PE=2 SV=3		2.442997	1	1	1	67.296	8.29	190000			2.801921
Q9QZM0	Ubiquilin-2 OS=Mus musculus GN=Ubqln2 PE=1 SV=2		2.351097	1	2	1	67.308	5.22	1500000		880000	5.303054
Q80YN3	Breast carcinoma-amplified sequence 1 homolog OS=Mus musculus GN=Bcas1 PE=1 SV=3		14.84992	6	19	6	67.336	6.21	3600000	5200000	4400000	59.23462
Q9ET30	Transmembrane 9 superfamily member 3 OS=Mus musculus GN=Tm9sf3 PE=1 SV=1		5.621806	4	13	4	67.5	7.21	6400000	6600000	4800000	34.02401
Q9D2N9	Vacuolar protein sorting-associated protein 33A OS=Mus musculus GN=Vps33a PE=1 SV=2		1.839465	1	1	1	67.512	7.08		1500000		2.491272
P29351	Tyrosine-protein phosphatase non-receptor type 6 OS=Mus musculus GN=Ptpn6 PE=1 SV=2		29.2437	14	42	14	67.517	7.81	16000000	15000000	13000000	121.8303
O08599	Syntaxin-binding protein 1 OS=Mus musculus GN=Stxbp1 PE=1 SV=2		30.47138	18	63	18	67.526	6.96	42000000	26000000	25000000	184.8836
Q9CZW5	Mitochondrial import receptor subunit TOM70 OS=Mus musculus GN=Tomm70 PE=1 SV=2		12.43863	8	19	8	67.547	7.53	10000000	8700000	8100000	50.98514
P40142	Transketolase OS=Mus musculus GN=Tkt PE=1 SV=1		23.11396	11	37	11	67.588	7.5	14000000	13000000	10000000	114.1914
P26041	Moesin OS=Mus musculus GN=Msn PE=1 SV=3		42.28769	23	77	15	67.725	6.6	21000000	28000000	19000000	226.7853
Q9EQ61	Pescadillo homolog OS=Mus musculus GN=Pes1 PE=1 SV=1		9.246575	3	6	3	67.753	6.84	1200000	910000	500000	18.64789
Q99MN1	Lysine--tRNA ligase OS=Mus musculus GN=Kars PE=1 SV=1		26.55462	13	35	13	67.796	5.94	7900000	6900000	6400000	97.68621
Q91ZR2	Sorting nexin-18 OS=Mus musculus GN=Snx18 PE=1 SV=1		4.397394	2	4	2	67.862	6.67	1100000	1700000	300000	11.19563
Q8BMF4	Dihydrolipoyllysine-residue acetyltransferase component of pyruvate dehydrogenase complex, mitochondrial OS=Mus musculus G		11.05919	5	8	5	67.899	8.57	7700000	2200000	5900000	23.27503
Q60770	Syntaxin-binding protein 3 OS=Mus musculus GN=Stxbp3 PE=1 SV=1		6.081081	3	7	3	67.899	8.02	1500000	1400000	1900000	22.61352
Q8BIA4	F-box/WD repeat-containing protein 8 OS=Mus musculus GN=Fbxw8 PE=1 SV=2		1.839465	1	1	1	67.913	6.16	390000			2.143739
P60670	Nuclear protein localization protein 4 homolog OS=Mus musculus GN=Nploc4 PE=1 SV=3		5.098684	3	4	3	67.974	6.46	2500000	1900000		11.00097
Q9WU79	Proline dehydrogenase 1, mitochondrial OS=Mus musculus GN=Prodh PE=1 SV=2		1.669449	1	1	1	67.993	8.24	500000			2.247401
Q8BHJ9	Pre-mRNA-splicing factor SLU7 OS=Mus musculus GN=Slu7 PE=1 SV=1		7.179487	3	3	3	68.038	7.08	770000	3500000	670000	9.99529
Q921G7	Electron transfer flavoprotein-ubiquinone oxidoreductase, mitochondrial OS=Mus musculus GN=Etfdh PE=1 SV=1		9.090909	5	11	5	68.048	7.58	4300000	3800000	4700000	30.13492
Q61010	E3 ubiquitin-protein ligase DTX1 OS=Mus musculus GN=Dtx1 PE=1 SV=2		4.14673	1	1	1	68.076	9.55			1100000	2.556886
P15636	SWISS-PROT:P15636 Protease I precursor Lysyl endopeptidase Achromobacter lyticus.	x	24.65544	11	209	11	68.083	7.24	3.9E+09	3.9E+09	2.7E+09	854.1845
Q91X20	Set1/Ash2 histone methyltransferase complex subunit ASH2 OS=Mus musculus GN=Ash2l PE=1 SV=1		1.284109	1	1	1	68.207	5.69			1100000	1.877327

P50516	V-type proton ATPase catalytic subunit A OS=Mus musculus GN=Atp6v1a PE=1 SV=2		20.25932	12	39	12	68.283	5.58	16000000	10000000	10000000	105.7069
Q9Z1M0	P2X purinoceptor 7 OS=Mus musculus GN=P2rx7 PE=1 SV=2		4.705882	2	8	2	68.344	8.22	1400000	2600000	1700000	22.86084
O88487	Cytoplasmic dynein 1 intermediate chain 2 OS=Mus musculus GN=Dync1i2 PE=1 SV=1		12.5817	5	17	5	68.352	5.29	16000000	13000000	10000000	61.41895
P35235	Tyrosine-protein phosphatase non-receptor type 11 OS=Mus musculus GN=Ptpn11 PE=1 SV=2		4.522613	2	3	2	68.417	7.3	1400000	2200000		9.163301
Q61545	RNA-binding protein EWS OS=Mus musculus GN=Ewsr1 PE=1 SV=2		7.175573	3	12	3	68.42	9.33	6500000	6600000	5700000	39.97691
Q6ZPL9	ATP-dependent RNA helicase DDX55 OS=Mus musculus GN=Ddx55 PE=1 SV=2		1.5	1	2	1	68.421	9.38	500000	410000		4.07005
Q8CJ53	Cdc42-interacting protein 4 OS=Mus musculus GN=Trip10 PE=1 SV=2		2.155887	1	1	1	68.447	5.9	430000			2.156428
Q99KN9	Clathrin interactor 1 OS=Mus musculus GN=Clint1 PE=1 SV=2		10.14263	5	8	5	68.469	6.25	4800000	3000000	3700000	24.08694
Q91YQ5	Dolichyl-diphosphooligosaccharide--protein glycosyltransferase subunit 1 OS=Mus musculus GN=Rpn1 PE=1 SV=1		22.20395	12	37	12	68.486	6.46	31000000	28000000	30000000	107.2003
Q91WJ8	Far upstream element-binding protein 1 OS=Mus musculus GN=Fubp1 PE=1 SV=1		8.448541	4	11	3	68.497	7.93	2200000	1600000	1400000	31.25564
P26043	Radixin OS=Mus musculus GN=Rdx PE=1 SV=3		22.81304	13	34	4	68.5	6.2	2400000	4700000	3200000	95.41341
Q8BGZ4	Cell division cycle protein 23 homolog OS=Mus musculus GN=Cdc23 PE=1 SV=2		2.177554	1	3	1	68.518	7.18	2600000	2000000	1700000	8.113169
Q8R050	Eukaryotic peptide chain release factor GTP-binding subunit ERF3A OS=Mus musculus GN=Gspt1 PE=1 SV=2		7.54717	3	3	3	68.582	5.21		3500000		9.395604
P07724	Serum albumin OS=Mus musculus GN=Alb PE=1 SV=3		63.98026	33	132	31	68.648	6.07	98000000	94000000	97000000	398.6003
Q8JZN5	Acyl-CoA dehydrogenase family member 9, mitochondrial OS=Mus musculus GN=Acad9 PE=1 SV=2		3.36	2	4	2	68.679	7.46	2600000	2500000	2000000	10.21772
P36916	Guanine nucleotide-binding protein-like 1 OS=Mus musculus GN=Gnl1 PE=1 SV=4		5.601318	3	4	3	68.729	5.68	1200000	1300000	1100000	9.596326
Q8BGD9	Eukaryotic translation initiation factor 4B OS=Mus musculus GN=Eif4b PE=1 SV=1		21.76759	11	24	11	68.799	5.67	7900000	10000000	6000000	76.76806
Q3ULA2	F-box/WD repeat-containing protein 1A OS=Mus musculus GN=Btrc PE=1 SV=2		1.983471	1	1	1	68.878	7.88			580000	2.513061
Q9DBU0	Transmembrane 9 superfamily member 1 OS=Mus musculus GN=Tm9sf1 PE=2 SV=2		1.980198	1	2	1	68.883	7.09		2100000	2800000	5.137907
Q60596	DNA repair protein XRCC1 OS=Mus musculus GN=Xrcc1 PE=1 SV=2		3.328051	2	4	2	68.929	6.33	1900000	1600000	1300000	11.75496
P35922	Synaptic functional regulator FMR1 OS=Mus musculus GN=Fmr1 PE=1 SV=1		7.81759	4	8	3	68.947	7.62	2200000	2300000	2400000	24.86885
Q6A028	Switch-associated protein 70 OS=Mus musculus GN=Swap70 PE=1 SV=2		2.051282	1	1	1	68.953	6.05			1900000	3.221696
Q8VEE4	Replication protein A 70 kDa DNA-binding subunit OS=Mus musculus GN=Rpa1 PE=1 SV=1		2.086677	1	1	1	68.994	7.91		3900000		2.670572
Q9Z0H1	WD repeat-containing protein 46 OS=Mus musculus GN=Wdr46 PE=2 SV=1		3.376206	1	1	1	69.005	9.77			440000	2.237817
P24527	Leukotriene A-4 hydrolase OS=Mus musculus GN=Lta4h PE=1 SV=4		8.674304	4	8	4	69.007	6.42	2000000	2400000	2500000	24.43334
Q9DBG6	Dolichyl-diphosphooligosaccharide--protein glycosyltransferase subunit 2 OS=Mus musculus GN=Rpn2 PE=1 SV=1		15.21395	7	19	7	69.02	5.81	13000000	13000000	10000000	58.82475
Q8JZN7	Mitochondrial Rho GTPase 2 OS=Mus musculus GN=Rhot2 PE=1 SV=1		2.258065	1	1	1	69.027	6		990000		3.160822
Q9JHS4	ATP-dependent Clp protease ATP-binding subunit clpX-like, mitochondrial OS=Mus musculus GN=Clpx PE=1 SV=2		8.675079	4	13	4	69.186	7.71	1100000	960000	1000000	39.18907
P20917	Myelin-associated glycoprotein OS=Mus musculus GN=Mag PE=1 SV=2		2.236422	1	1	1	69.216	5.1	4600000			3.178081
Q61656	Probable ATP-dependent RNA helicase DDX5 OS=Mus musculus GN=Ddx5 PE=1 SV=2		35.66775	24	75	17	69.247	8.92	31000000	30000000	31000000	197.709
O89103	Complement component C1q receptor OS=Mus musculus GN=Cd93 PE=1 SV=1		1.863354	1	2	1	69.309	5.07	1100000		1000000	5.792049
P02768-1	SWISS-PROT:P02768-1 Tax_Id=9606 Gene_Symbol=ALB Isoform 1 of Serum albumin precursor	x	14.28571	8	14	6	69.321	6.28	1400000	1200000	4000000	35.44424
P26040	Ezrin OS=Mus musculus GN=Ezr PE=1 SV=3		26.96246	17	49	9	69.364	6.1	11000000	8800000	8600000	143.653
Q8C2Q3	RNA-binding protein 14 OS=Mus musculus GN=Rbm14 PE=1 SV=1		18.23617	10	20	10	69.406	9.67	3400000	3500000	3300000	51.42654
P23475	X-ray repair cross-complementing protein 6 OS=Mus musculus GN=Xrcc6 PE=1 SV=5		16.28289	8	17	8	69.441	6.79	3100000	3300000	3200000	54.20989
Q9Z2X8	Kelch-like ECH-associated protein 1 OS=Mus musculus GN=Keap1 PE=1 SV=1		4.00641	2	2	2	69.508	6.44	1700000	590000		4.732821
Q8R361	Rab11 family-interacting protein 5 OS=Mus musculus GN=Rab11fip5 PE=1 SV=2		2.790698	1	4	1	69.51	9.07	260000	310000	73000	8.580914
Q6P1B1	Xaa-Pro aminopeptidase 1 OS=Mus musculus GN=Xpnpep1 PE=1 SV=1		2.889246	1	2	1	69.547	5.54	460000	390000		5.321411
P10404	MLV-related proviral Env polyprotein OS=Mus musculus PE=1 SV=3		16.22465	9	24	7	69.569	7.96	32000000	24000000	27000000	77.36435
Q9DBG7	Signal recognition particle receptor subunit alpha OS=Mus musculus GN=Srpra PE=1 SV=1		8.490566	5	11	5	69.579	8.95	1900000	3000000	3600000	25.71014
Q7TMK9	Heterogeneous nuclear ribonucleoprotein Q OS=Mus musculus GN=Syncrip PE=1 SV=2		31.13965	15	46	15	69.59	8.59	22000000	18000000	18000000	130.376
P17156	Heat shock-related 70 kDa protein 2 OS=Mus musculus GN=Hspa2 PE=1 SV=2		24.32859	14	60	4	69.599	5.67	3400000	1800000	1700000	191.4953
Q9CXI3	DBH-like monooxygenase protein 1 OS=Mus musculus GN=Moxd1 PE=1 SV=1		10.27732	6	18	6	69.633	6.67	9000000	9200000	9100000	48.74402
Q68ED3	Non-canonical poly(A) RNA polymerase PAPD5 OS=Mus musculus GN=Papd5 PE=1 SV=2		2.369668	1	1	1	69.661	8.78		130000		2.053637
Q99LJ0	CTTNBP2 N-terminal-like protein OS=Mus musculus GN=Cttnbp2nl PE=1 SV=1		8.46395	5	12	5	69.798	7.71	1500000	1500000	1200000	33.41071
Q923D5	WW domain-binding protein 11 OS=Mus musculus GN=Wbp11 PE=1 SV=2		7.4883	4	10	4	69.831	8.4	2900000	2600000	2800000	27.24802
Q8C0E3	Tripartite motif-containing protein 47 OS=Mus musculus GN=Trim47 PE=1 SV=2		2.340094	1	1	1	69.868	6.4				3.27973
P31650	Sodium- and chloride-dependent GABA transporter 3 OS=Mus musculus GN=Slc6a11 PE=1 SV=2		5.741627	3	5	3	69.914	6.98	3600000		2600000	12.66533
Q8BZR9	Nuclear cap-binding protein subunit 3 OS=Mus musculus GN=Ncbp3 PE=1 SV=1		5.691057	2	4	2	70	5.8	1000000	1700000	1200000	12.02081
Q8R4C2	RUN and FYVE domain-containing protein 2 OS=Mus musculus GN=Rufy2 PE=1 SV=2		1.815182	1	1	1	70.049	5.83			3500000	2.771088
P43404	Tyrosine-protein kinase ZAP-70 OS=Mus musculus GN=Zap70 PE=1 SV=3		2.427184	1	1	1	70.066	7.83		780000		3.086769
Q61233	Plastin-2 OS=Mus musculus GN=Lcp1 PE=1 SV=4		28.70813	13	34	11	70.105	5.33	6400000	5700000	4900000	101.9366
P17879	Heat shock 70 kDa protein 1B OS=Mus musculus GN=Hspa1b PE=1 SV=3		11.99377	6	23	1	70.133	5.72	1800000	850000	470000	70.7727

Q99JP7	Gamma-glutamyltransferase 7 OS=Mus musculus GN=Ggt7 PE=1 SV=2		2.719033	1	3	1	70.208	5.06	1100000	820000	820000	7.308992
Q9DC50	Peroxisomal carnitine O-octanoyltransferase OS=Mus musculus GN=Crot PE=1 SV=1		2.941176	1	1	1	70.219	6.73		490000		2.568528
P19221	Prothrombin OS=Mus musculus GN=F2 PE=1 SV=1		9.87055	5	11	5	70.224	6.43	2400000	2900000	2600000	29.38406
Q99K43	Protein regulator of cytokinesis 1 OS=Mus musculus GN=Prc1 PE=1 SV=2		6.633499	4	8	3	70.246	7.58	1100000	1300000	1000000	16.72253
Q8BYY4	Tetratricopeptide repeat protein 39B OS=Mus musculus GN=Ttc39b PE=1 SV=1		1.296596	1	1	1	70.248	6.64		560000		2.116921
Q99JX7	Nuclear RNA export factor 1 OS=Mus musculus GN=Nxf1 PE=1 SV=3		1.618123	1	1	1	70.256	8.73		1800000		2.738546
P54729	NEDD8 ultimate buster 1 OS=Mus musculus GN=Nub1 PE=1 SV=2		6.351792	3	6	3	70.263	5.88	2000000	2400000	920000	16.46148
O88428	Bifunctional 3'-phosphoadenosine 5'-phosphosulfate synthase 2 OS=Mus musculus GN=Papss2 PE=1 SV=2		4.991948	3	5	3	70.306	7.58	1800000	1400000	1400000	11.96484
Q6P9R1	ATP-dependent RNA helicase DDX51 OS=Mus musculus GN=Ddx51 PE=1 SV=1		13.14554	4	9	4	70.324	9.47	630000	650000	780000	26.23428
Q64213	Splicing factor 1 OS=Mus musculus GN=Sf1 PE=1 SV=6		4.134763	3	9	3	70.358	8.98	4100000	3700000	1300000	28.80887
Q8VCY6	U3 small nucleolar RNA-associated protein 6 homolog OS=Mus musculus GN=Utp6 PE=2 SV=1		2.345059	1	3	1	70.384	7.71	880000	1000000	1200000	9.256124
Q9JLQ0	CD2-associated protein OS=Mus musculus GN=Cd2ap PE=1 SV=3		9.733124	4	6	4	70.407	6.38	1000000	1100000	1200000	17.59394
P59016	Vacuolar protein sorting-associated protein 33B OS=Mus musculus GN=Vps33b PE=1 SV=1		4.376013	2	4	2	70.481	6.86	1500000	890000	1300000	11.55695
Q8BH04	Phosphoenolpyruvate carboxykinase [GTP], mitochondrial OS=Mus musculus GN=Pck2 PE=1 SV=1		5.78125	3	5	3	70.482	7.28	1500000	890000	1300000	13.36866
Q9Z1F9	SUMO-activating enzyme subunit 2 OS=Mus musculus GN=Uba2 PE=1 SV=1		14.10658	6	10	6	70.525	5.24	3300000	4700000	1300000	32.664
Q8BMA6	Signal recognition particle subunit SRP68 OS=Mus musculus GN=Srp68 PE=1 SV=2		30.56	15	32	15	70.53	8.57	6200000	4700000	5800000	90.84052
Q5SVQ0	Histone acetyltransferase KAT7 OS=Mus musculus GN=Kat7 PE=1 SV=1		4.893964	2	5	2	70.597	8.72		1400000	1400000	13.48823
P29341	Polyadenylate-binding protein 1 OS=Mus musculus GN=Pabpc1 PE=1 SV=2		22.01258	13	39	13	70.626	9.5	17000000	14000000	14000000	109.3265
Q99PG2	Opioid growth factor receptor OS=Mus musculus GN=Ogfr PE=1 SV=1		23.5387	6	17	6	70.636	4.77	1600000	2400000	980000	56.50102
Q924H7	WW domain-containing adapter protein with coiled-coil OS=Mus musculus GN=Wac PE=1 SV=2		2.631579	1	1	1	70.637	9.45		1500000		3.443511
O88485	Cytoplasmic dynein 1 intermediate chain 1 OS=Mus musculus GN=Dync1i1 PE=1 SV=2		7.324841	3	6	3	70.681	5.12	610000		270000	18.70071
Q7TNG5	Echinoderm microtubule-associated protein-like 2 OS=Mus musculus GN=Eml2 PE=1 SV=1		3.081664	2	3	2	70.689	6.28	1800000	710000	1500000	6.485986
Q99K51	Plastin-3 OS=Mus musculus GN=Pls3 PE=1 SV=3		13.33333	6	13	4	70.697	5.62	1000000	1500000	1100000	38.07757
Q60967	Bifunctional 3'-phosphoadenosine 5'-phosphosulfate synthase 1 OS=Mus musculus GN=Papss1 PE=1 SV=1		3.205128	2	2	2	70.749	6.77	4000000	2100000		4.198499
Q61107	Guanylate-binding protein 4 OS=Mus musculus GN=Gbp4 PE=1 SV=1		16.12903	8	31	8	70.757	6.64	9500000	5900000	7600000	100.235
P47934	Carnitine O-acetyltransferase OS=Mus musculus GN=Crat PE=1 SV=3		1.916933	1	2	1	70.794	8.44	880000		540000	4.014158
Q9JIG7	Coiled-coil domain-containing protein 22 OS=Mus musculus GN=Ccdc22 PE=1 SV=1		9.250399	4	6	4	70.8	6.01	560000	1100000	910000	16.96333
P63017	Heat shock cognate 71 kDa protein OS=Mus musculus GN=Hspa8 PE=1 SV=1		60.21672	36	199	25	70.827	5.52	2.2E+08	1.7E+08	1.6E+08	635.9082
P50544	Very long-chain specific acyl-CoA dehydrogenase, mitochondrial OS=Mus musculus GN=Acadvl PE=1 SV=3		9.756098	5	9	5	70.831	8.75	3500000	1900000	2800000	27.27788
Q6ZPU9	KIF1-binding protein OS=Mus musculus GN=Kif1bp PE=1 SV=2		7.941653	2	4	2	71.007	5.48	1200000	380000		13.22271
Q8K297	Procollagen galactosyltransferase 1 OS=Mus musculus GN=Colgalt1 PE=1 SV=2		10.69692	7	13	7	71.015	7.28	3000000	3200000	3300000	38.08342
Q60714	Long-chain fatty acid transport protein 1 OS=Mus musculus GN=Slc27a1 PE=1 SV=1		5.727554	3	3	3	71.231	8.38	740000	670000		8.695629
Q80TY0	Formin-binding protein 1 OS=Mus musculus GN=Fnbp1 PE=1 SV=2		16.55844	7	11	7	71.299	5.67	1800000	2500000	1400000	24.93632
Q8K2Q9	Shootin-1 OS=Mus musculus GN=Shtn1 PE=1 SV=1		3.169572	1	2	1	71.299	5.44		360000	490000	5.039536
P48025	Tyrosine-protein kinase SYK OS=Mus musculus GN=Syk PE=1 SV=2		1.90779	1	2	1	71.331	7.88	990000	870000		4.392788
Q3U9G9	Lamin-B receptor OS=Mus musculus GN=Lbr PE=1 SV=2		15.17572	9	22	9	71.395	9.36	13000000	16000000	16000000	59.4906
Q9R0L7	A-kinase anchor protein 8-like OS=Mus musculus GN=Akap8l PE=1 SV=1		4.049844	2	4	2	71.408	5.05	2400000	1500000	1100000	12.71561
Q8R349	Cell division cycle protein 16 homolog OS=Mus musculus GN=Cdc16 PE=1 SV=1		5.483871	3	7	3	71.414	5.76	1600000	1300000	1700000	20.3828
Q7M6Y3	Phosphatidylinositol-binding clathrin assembly protein OS=Mus musculus GN=Picalm PE=1 SV=1		3.333333	2	2	1	71.498	7.91				4.742513
Q8R2Y2	Cell surface glycoprotein MUC18 OS=Mus musculus GN=Mcam PE=1 SV=1		23.61111	12	35	12	71.501	5.83	21000000	20000000	9500000	115.9355
Q8VDG3	Poly(A)-specific ribonuclease PARN OS=Mus musculus GN=Parn PE=1 SV=1		4.166667	2	6	2	71.514	5.97	2300000	2200000	2100000	19.16841
P15092	Interferon-activable protein 204 OS=Mus musculus GN=Ifi204 PE=1 SV=2		19.21875	9	27	8	71.605	8.69	6000000	5400000	6000000	89.29941
Q8C0I1	Alkyldihydroxyacetonephosphate synthase, peroxisomal OS=Mus musculus GN=Agps PE=1 SV=1		7.44186	3	6	3	71.638	7.5		880000	1000000	16.87559
P54103	DnaJ homolog subfamily C member 2 OS=Mus musculus GN=Dnajc2 PE=1 SV=2		16.58615	8	15	8	71.677	8.7	2800000	3700000	3000000	41.72254
Q61510	E3 ubiquitin/ISG15 ligase TRIM25 OS=Mus musculus GN=Trim25 PE=1 SV=2		6.309148	3	9	3	71.68	8.28	1400000	1400000	1400000	24.99367
Q99LE6	ATP-binding cassette sub-family F member 2 OS=Mus musculus GN=Abcf2 PE=1 SV=1		9.713376	5	14	5	71.736	7.05	4900000	4800000	4800000	40.36081
Q91XU0	ATPase WRNIP1 OS=Mus musculus GN=Wrnip1 PE=1 SV=2		1.969697	1	1	1	71.749	6.18				2.90282
P08003	Protein disulfide-isomerase A4 OS=Mus musculus GN=Pdia4 PE=1 SV=3		46.55172	24	87	24	71.938	5.31	35000000	37000000	32000000	274.6146
Q8K3A9	7SK snRNA methylphosphate capping enzyme OS=Mus musculus GN=Mepce PE=1 SV=2		8.858859	3	4	3	72.006	9.25	570000	1600000		14.74752
Q8CH72	E3 ubiquitin-protein ligase TRIM32 OS=Mus musculus GN=Trim32 PE=1 SV=2		2.900763	2	4	2	72.011	6.9	2500000	4000000	750000	9.236437
Q61550	Double-strand-break repair protein rad21 homolog OS=Mus musculus GN=Rad21 PE=1 SV=3		17.00787	9	19	9	72.038	4.64	6500000	4400000	5100000	55.23701
Q8BG51	Mitochondrial Rho GTPase 1 OS=Mus musculus GN=Rhot1 PE=1 SV=1		5.388273	2	4	2	72.196	6.49	2700000	2200000	1600000	11.68743
Q8CB44	GRAM domain-containing protein 4 OS=Mus musculus GN=Gramd4 PE=1 SV=1		4.739336	2	3	2	72.218	9.07	460000	300000		7.827994

P70663	SPARC-like protein 1 OS=Mus musculus GN=Sparcl1 PE=1 SV=3		12.30769	5	17	5	72.243	4.6	2300000	4000000	890000	49.41711
O08582	GTP-binding protein 1 OS=Mus musculus GN=Gtpbp1 PE=1 SV=2		3.592814	2	2	2	72.255	8.29	1300000	2100000		6.04075
Q91VE0	Long-chain fatty acid transport protein 4 OS=Mus musculus GN=Slc27a4 PE=1 SV=1		5.909798	3	4	3	72.272	8.59	1400000	2500000	1900000	10.94907
Q8BRF7	Sec1 family domain-containing protein 1 OS=Mus musculus GN=Scfd1 PE=1 SV=1		9.076682	4	10	4	72.277	6.38	4300000	4100000	3900000	33.29501
Q501J6	Probable ATP-dependent RNA helicase DDX17 OS=Mus musculus GN=Ddx17 PE=1 SV=1		36.92308	24	61	17	72.354	8.59	11000000	13000000	12000000	170.1901
O70161	Phosphatidylinositol 4-phosphate 5-kinase type-1 gamma OS=Mus musculus GN=Pip5k1c PE=1 SV=2		4.236006	2	2	2	72.363	5.59			560000	5.80612
Q8VCT3	Aminopeptidase B OS=Mus musculus GN=Rnpep PE=1 SV=2		2.769231	1	2	1	72.37	5.35	1600000	950000		5.062127
P20029	78 kDa glucose-regulated protein OS=Mus musculus GN=Hspa5 PE=1 SV=3		49.46565	35	168	33	72.377	5.16	1.7E+08	1.7E+08	1.4E+08	553.8892
Q9Z129	ATP-dependent DNA helicase Q1 OS=Mus musculus GN=Recql PE=1 SV=2		1.234568	1	3	1	72.438	8.34	750000	640000	590000	7.42008
Q3UDW8	Heparan-alpha-glucosaminide N-acetyltransferase OS=Mus musculus GN=Hgsnat PE=1 SV=2		3.658537	2	6	2	72.458	8.27	3700000	2600000	3200000	17.78651
P97494	Glutamate--cysteine ligase catalytic subunit OS=Mus musculus GN=Gclc PE=1 SV=4		2.825746	1	3	1	72.525	5.9	250000		540000	9.588662
Q8K2B3	Succinate dehydrogenase [ubiquinone] flavoprotein subunit, mitochondrial OS=Mus musculus GN=Sdha PE=1 SV=1		17.31928	9	20	9	72.539	7.37	14000000	9600000	10000000	60.20595
Q7TQK5	Coiled-coil domain-containing protein 93 OS=Mus musculus GN=Ccdc93 PE=1 SV=1		5.246423	3	4	3	72.558	8.29	1500000		1500000	8.415634
Q60710	Deoxynucleoside triphosphate triphosphohydrolase SAMHD1 OS=Mus musculus GN=Samhd1 PE=1 SV=2		37.32057	20	57	20	72.604	7.96	27000000	27000000	22000000	155.5017
Q8CH02	SURP and G-patch domain-containing protein 1 OS=Mus musculus GN=Sugp1 PE=1 SV=1		5.132193	2	4	2	72.604	7.64		740000	730000	11.44961
Q8CIG8	Protein arginine N-methyltransferase 5 OS=Mus musculus GN=Prmt5 PE=1 SV=3		6.122449	3	5	3	72.634	6.42	670000	1600000	1300000	11.43522
Q8BKS9	Pumilio homolog 3 OS=Mus musculus GN=Pum3 PE=1 SV=2		5.718702	4	10	4	72.754	9.66	2000000	1900000	1700000	20.60344
O08688	Calpain-5 OS=Mus musculus GN=Capn5 PE=1 SV=1		1.71875	1	1	1	72.909	7.36	1300000			2.021188
O70551	SRSF protein kinase 1 OS=Mus musculus GN=Srpk1 PE=1 SV=2		8.641975	4	5	3	73.043	6.19		2400000	1900000	14.12841
Q62167	ATP-dependent RNA helicase DDX3X OS=Mus musculus GN=Ddx3x PE=1 SV=3		19.78852	12	34	1	73.056	7.18	1900000	800000	910000	97.68412
O08677	Kininogen-1 OS=Mus musculus GN=Kng1 PE=1 SV=1		7.564297	4	8	4	73.056	6.54	1800000	1600000	1400000	25.52272
Q9R049	E3 ubiquitin-protein ligase AMFR OS=Mus musculus GN=Amfr PE=1 SV=2		2.332815	1	3	1	73.058	6.46	970000	1100000	1300000	6.910671
Q3TWF6	WD repeat-containing protein 70 OS=Mus musculus GN=Wdr70 PE=1 SV=1		2.283105	1	2	1	73.073	5.97	1100000	1300000		6.361663
Q3UM18	Large subunit GTPase 1 homolog OS=Mus musculus GN=Lsg1 PE=1 SV=2		3.726708	2	3	2	73.111	6.51		920000	890000	7.846689
Q8BYW1	Rho GTPase-activating protein 25 OS=Mus musculus GN=Arhgap25 PE=1 SV=2		4.783951	1	1	1	73.337	6.38		990000		3.985105
Q8CEC6	Peptidylprolyl isomerase domain and WD repeat-containing protein 1 OS=Mus musculus GN=Ppwd1 PE=1 SV=2		5.882353	2	4	2	73.338	7.15	3200000	3000000	1500000	14.24641
Q62095	ATP-dependent RNA helicase DDX3Y OS=Mus musculus GN=Ddx3y PE=1 SV=2		19.75684	12	32	1	73.382	7.53	470000			92.12945
A2ALS5	Rap1 GTPase-activating protein 1 OS=Mus musculus GN=Rap1gap PE=1 SV=2		4.223228	2	4	2	73.387	5.87	1400000	1400000	690000	11.1983
P38647	Stress-70 protein, mitochondrial OS=Mus musculus GN=Hspa9 PE=1 SV=3		39.17526	23	75	23	73.416	6.07	26000000	23000000	20000000	241.1181
Q00780	Collagen alpha-1(VIII) chain OS=Mus musculus GN=Col8a1 PE=1 SV=3		1.747312	1	2	1	73.559	9.55		1500000	620000	4.096906
O88845	A-kinase anchor protein 10, mitochondrial OS=Mus musculus GN=Akap10 PE=1 SV=3		2.416918	1	1	1	73.586	6.79	780000			2.865756
Q9WVR4	Fragile X mental retardation syndrome-related protein 2 OS=Mus musculus GN=Fxr2 PE=1 SV=1		8.618128	5	12	4	73.698	6.23	2800000	2800000	2100000	37.45636
O35492	Dual specificity protein kinase CLK3 OS=Mus musculus GN=Clk3 PE=1 SV=2		2.037618	1	2	1	73.751	9.94	520000	550000		4.289148
Q8K3G9	DCC-interacting protein 13-beta OS=Mus musculus GN=Appl2 PE=1 SV=1		2.114804	1	1	1	73.807	5.03	560000			2.886931
Q91WC9	Sn1-specific diacylglycerol lipase beta OS=Mus musculus GN=Daglb PE=1 SV=2		2.541106	1	2	1	73.857	6.73		670000	590000	4.869922
D3Z7P3	Glutaminase kidney isoform, mitochondrial OS=Mus musculus GN=Gls PE=1 SV=1		8.902077	4	9	4	73.916	7.99	2800000	5000000	3500000	29.12795
P52825	Carnitine O-palmitoyltransferase 2, mitochondrial OS=Mus musculus GN=Cpt2 PE=1 SV=2		3.951368	2	2	2	73.934	8.37	600000			4.807206
O88665	Bromodomain-containing protein 7 OS=Mus musculus GN=Brd7 PE=1 SV=1		1.843318	1	2	1	73.954	6.38	390000		620000	5.414691
Q9D706	RNA polymerase II-associated protein 3 OS=Mus musculus GN=Rpap3 PE=1 SV=1		7.424242	4	8	4	74.05	7.99	1600000	1400000	1100000	26.8281
O88935	Synapsin-1 OS=Mus musculus GN=Syn1 PE=1 SV=2		25.21246	12	37	11	74.052	9.8	15000000	6700000	5100000	113.9565
Q99ME9	Nucleolar GTP-binding protein 1 OS=Mus musculus GN=Gtpbp4 PE=1 SV=3		11.19874	7	15	7	74.066	9.52	2900000	4100000	2600000	36.53604
Q9QWT9	Kinesin-like protein KIFC1 OS=Mus musculus GN=Kifc1 PE=1 SV=2		2.077151	1	3	1	74.107	8.72	1000000	1100000	950000	9.540281
Q8K363	ATP-dependent RNA helicase DDX18 OS=Mus musculus GN=Ddx18 PE=1 SV=1		19.69697	8	23	8	74.134	9.52	4700000	5300000	4900000	80.89988
P48678	Prelamin-A/C OS=Mus musculus GN=Lmna PE=1 SV=2		53.23308	35	119	34	74.193	6.98	32000000	36000000	31000000	335.8477
Q3UMU9	Hepatoma-derived growth factor-related protein 2 OS=Mus musculus GN=Hdgfrp2 PE=1 SV=1		3.28849	2	6	2	74.246	8.66	7600000	7600000	6800000	17.25995
Q62192	CD180 antigen OS=Mus musculus GN=Cd180 PE=1 SV=2		2.420575	1	3	1	74.255	5.88	7500000	7500000	4900000	11.99613
Q8R5A3	Amyloid beta A4 precursor protein-binding family B member 1-interacting protein OS=Mus musculus GN=Apbb1ip PE=1 SV=2		2.835821	2	5	2	74.272	5.35	1500000	1800000	1000000	10.02568
P11370	Retrovirus-related Env polyprotein from Fv-4 locus OS=Mus musculus GN=Fv4 PE=1 SV=2		8.247423	4	14	2	74.406	7.81	1600000	1900000	990000	42.77931
Q9QXX4	Calcium-binding mitochondrial carrier protein Aralar2 OS=Mus musculus GN=Slc25a13 PE=1 SV=1		3.402367	3	5	1	74.42	8.6	800000		1000000	11.58008
Q8BH59	Calcium-binding mitochondrial carrier protein Aralar1 OS=Mus musculus GN=Slc25a12 PE=1 SV=1		15.06647	10	19	8	74.523	8.25	8200000	5600000	5200000	51.68012
P82198	Transforming growth factor-beta-induced protein ig-h3 OS=Mus musculus GN=Tgfbi PE=1 SV=1		25.18302	15	43	15	74.55	7.06	22000000	22000000	22000000	130.5283
Q9R0H0	Peroxisomal acyl-coenzyme A oxidase 1 OS=Mus musculus GN=Acox1 PE=1 SV=5		2.269289	1	2	1	74.601	8.48	1400000		530000	5.687672
Q8BH24	Transmembrane 9 superfamily member 4 OS=Mus musculus GN=Tm9sf4 PE=1 SV=1		5.287714	2	6	2	74.644	7.23	3500000	3400000	3600000	20.02067

Q8R480	Nuclear pore complex protein Nup85 OS=Mus musculus GN=Nup85 PE=1 SV=1		5.182927	3	7	3	74.728	5.57	2000000	1500000	1600000	18.15418
Q0VEE6	Zinc finger protein 800 OS=Mus musculus GN=Znf800 PE=1 SV=1		3.47432	1	1	1	74.767	9.42		1000000		3.563832
Q8K0U4	Heat shock 70 kDa protein 12A OS=Mus musculus GN=Hspa12a PE=1 SV=1		11.25926	6	14	6	74.825	6.77	3700000	1700000	1900000	42.97478
Q7TQF7	Amphiphysin OS=Mus musculus GN=Amph PE=1 SV=1		15.01458	8	20	7	74.967	4.63	6000000	3100000	3100000	63.67389
Q9R190	Metastasis-associated protein MTA2 OS=Mus musculus GN=Mta2 PE=1 SV=1		11.82635	7	16	7	74.983	9.67	6100000	6300000	6000000	45.88993
Q62172	RalA-binding protein 1 OS=Mus musculus GN=Ralbp1 PE=1 SV=4		5.092593	3	3	3	74.997	5.92	450000		1800000	7.314363
Q61033	Lamina-associated polypeptide 2, isoforms alpha/zeta OS=Mus musculus GN=Tmpo PE=1 SV=4		26.40693	14	37	6	75.122	8.05	6800000	6700000	6800000	123.5567
Q8VBZ3	Cleft lip and palate transmembrane protein 1 homolog OS=Mus musculus GN=Clptm1 PE=1 SV=1		14.60843	5	17	5	75.243	6.3	3500000	4200000	3800000	61.53916
P58021	Transmembrane 9 superfamily member 2 OS=Mus musculus GN=Tm9sf2 PE=1 SV=1		6.797583	3	9	3	75.28	7.43	4300000	3700000	5000000	25.83344
Q6ZQL4	WD repeat-containing protein 43 OS=Mus musculus GN=Wdr43 PE=2 SV=2		6.351551	3	5	3	75.334	5.38	4400000	1100000	2900000	15.66799
Q8VIJ6	Splicing factor, proline- and glutamine-rich OS=Mus musculus GN=Sfpq PE=1 SV=1		26.32332	18	68	17	75.394	9.44	21000000	18000000	18000000	193.5578
P55096	ATP-binding cassette sub-family D member 3 OS=Mus musculus GN=Abcd3 PE=1 SV=2		4.400607	2	3	2	75.426	9.26	2500000		4100000	8.564005
P47708	Rabphilin-3A OS=Mus musculus GN=Rph3a PE=1 SV=2		4.845815	2	4	2	75.442	8.27	1700000	140000	660000	14.04901
Q5XJE5	RNA polymerase-associated protein LEO1 OS=Mus musculus GN=Leo1 PE=1 SV=2		1.949025	1	3	1	75.552	4.46	2800000	2600000	2300000	7.28491
Q8K1B8	Fermitin family homolog 3 OS=Mus musculus GN=Fermt3 PE=1 SV=1		14.73684	8	19	8	75.587	7.05	8800000	8200000	7900000	57.16056
Q5F2E7	Nuclear fragile X mental retardation-interacting protein 2 OS=Mus musculus GN=Nufip2 PE=1 SV=1		11.41618	5	8	5	75.611	8.7	1000000	1100000	1000000	20.87893
Q9D0I9	Arginine--tRNA ligase, cytoplasmic OS=Mus musculus GN=Rars PE=1 SV=2		18.48485	11	28	11	75.625	7.55	4400000	6900000	6100000	73.52556
P14824	Annexin A6 OS=Mus musculus GN=Anxa6 PE=1 SV=3		48.88559	28	87	27	75.837	5.5	28000000	22000000	23000000	281.1846
Q60649	Caseinolytic peptidase B protein homolog OS=Mus musculus GN=Clpb PE=1 SV=1		1.624815	1	1	1	75.956	8.51				2.731215
Q8K2C7	Protein OS-9 OS=Mus musculus GN=Os9 PE=1 SV=2		7.291667	4	6	4	76.061	4.84	2000000	710000	1000000	15.79398
Q8JZR0	Long-chain-fatty-acid--CoA ligase 5 OS=Mus musculus GN=Acsl5 PE=1 SV=1		10.2489	6	14	5	76.157	7.09	2000000	2200000	1200000	37.75358
Q61584	Fragile X mental retardation syndrome-related protein 1 OS=Mus musculus GN=Fxr1 PE=1 SV=2		23.33826	11	26	10	76.175	6.98	6700000	6000000	9100000	84.9186
P10637	Microtubule-associated protein tau OS=Mus musculus GN=Mapt PE=1 SV=3		11.05048	5	9	5	76.197	6.79	3000000	2300000	970000	21.85454
Q9DBR0	A-kinase anchor protein 8 OS=Mus musculus GN=Akap8 PE=1 SV=1		3.930131	2	5	2	76.247	5.14	2500000	3000000	3200000	15.00105
Q9CXF4	TBC1 domain family member 15 OS=Mus musculus GN=Tbc1d15 PE=1 SV=1		7.451565	4	8	4	76.478	5.3	1500000	1600000	1800000	20.30746
P28571	Sodium- and chloride-dependent glycine transporter 1 OS=Mus musculus GN=Slc6a9 PE=1 SV=3		3.179191	1	3	1	76.494	7.81	3500000	2400000	2300000	9.234369
Q501J7	Phosphatase and actin regulator 4 OS=Mus musculus GN=Phactr4 PE=1 SV=2		1.873199	1	1	1	76.585	6.48		600000		2.772363
Q8R3L2	Transcription factor 25 OS=Mus musculus GN=Tcf25 PE=1 SV=2		4.43787	2	2	2	76.637	6.51	700000	310000		4.289427
Q99J87	Probable ATP-dependent RNA helicase DHX58 OS=Mus musculus GN=Dhx58 PE=1 SV=2		4.719764	3	5	3	76.66	8.19	1900000	940000	1300000	13.43102
Q921I1	Serotransferrin OS=Mus musculus GN=Tf PE=1 SV=1		27.83357	16	43	16	76.674	7.18	14000000	5000000	11000000	126.2833
Q3THK7	GMP synthase [glutamine-hydrolyzing] OS=Mus musculus GN=Gmps PE=1 SV=2		6.926407	4	7	4	76.675	6.73	2300000	1800000	2000000	17.91591
P09405	Nucleolin OS=Mus musculus GN=Ncl PE=1 SV=2		30.55163	20	76	20	76.677	4.75	1.1E+08	1.2E+08	1.1E+08	218.3934
P68404	Protein kinase C beta type OS=Mus musculus GN=Prkcb PE=1 SV=4		14.00894	7	17	6	76.701	7.01	6400000	2800000	1400000	47.00786
O54781	SRSF protein kinase 2 OS=Mus musculus GN=Srpk2 PE=1 SV=2		7.929515	5	11	4	76.709	4.91	2400000	2400000	2600000	31.11818
Q3U0V1	Far upstream element-binding protein 2 OS=Mus musculus GN=Khsrp PE=1 SV=2		16.84492	8	15	7	76.728	7.33	2700000	2400000	2500000	41.24192
Q8BXA1	Golgi integral membrane protein 4 OS=Mus musculus GN=Golim4 PE=1 SV=1		16.18321	6	12	6	76.739	4.83	150000	2800000	1300000	37.47701
Q9QYY0	GRB2-associated-binding protein 1 OS=Mus musculus GN=Gab1 PE=1 SV=2		1.870504	1	1	1	76.764	5.67	340000			2.896545
Q6P9S0	MTSS1-like protein OS=Mus musculus GN=Mtss1l PE=1 SV=1		1.958042	1	1	1	76.797	6.99		650000		2.189593
P20444	Protein kinase C alpha type OS=Mus musculus GN=Prkca PE=1 SV=3		6.25	3	5	1	76.802	7.14	370000			16.03667
Q8R2N2	U3 small nucleolar RNA-associated protein 4 homolog OS=Mus musculus GN=Utp4 PE=2 SV=3		2.332362	2	3	2	76.861	8.87			1500000	6.120466
Q6NZQ2	Probable ATP-dependent RNA helicase DDX31 OS=Mus musculus GN=Ddx31 PE=2 SV=2		1.310044	1	2	1	76.865	9.74		430000	410000	4.034906
Q9Z2Z9	Glutamine--fructose-6-phosphate aminotransferase [isomerizing] 2 OS=Mus musculus GN=Gfpt2 PE=1 SV=3		3.372434	2	5	1	76.96	7.15	2000000	1800000	1400000	14.5336
P37040	NADPH--cytochrome P450 reductase OS=Mus musculus GN=Por PE=1 SV=2		10.76696	6	11	6	76.995	5.53	2700000	2900000	3600000	27.84718
P21981	Protein-glutamine gamma-glutamyltransferase 2 OS=Mus musculus GN=Tgm2 PE=1 SV=4		42.5656	23	79	23	77.012	5.1	86000000	92000000	84000000	231.8561
Q9QXS6	Drebrin OS=Mus musculus GN=Dbn1 PE=1 SV=4		19.97167	8	21	8	77.239	4.49	8600000	6700000	5000000	69.31745
Q08189	Protein-glutamine gamma-glutamyltransferase E OS=Mus musculus GN=Tgm3 PE=1 SV=2		1.875902	1	1	1	77.261	6.81			3900000	2.667471
P36371	Antigen peptide transporter 2 OS=Mus musculus GN=Tap2 PE=1 SV=1		19.80057	13	41	13	77.396	7.24	23000000	33000000	28000000	128.8006
Q8BG39	Synaptic vesicle glycoprotein 2B OS=Mus musculus GN=Sv2b PE=1 SV=1		6.881406	4	7	4	77.406	5.57	7300000	4400000	3800000	21.1649
Q922U1	U4/U6 small nuclear ribonucleoprotein Prp3 OS=Mus musculus GN=Prpf3 PE=1 SV=1		2.928258	2	3	2	77.407	9.5	3300000	3200000	3200000	5.921993
Q9QXK7	Cleavage and polyadenylation specificity factor subunit 3 OS=Mus musculus GN=Cpsf3 PE=1 SV=2		2.923977	2	4	2	77.455	5.6	1600000	1200000	1900000	9.745456
Q9D906	Ubiquitin-like modifier-activating enzyme ATG7 OS=Mus musculus GN=Atg7 PE=1 SV=1		1.719198	1	2	1	77.47	6.4	570000		350000	4.925999
P70302	Stromal interaction molecule 1 OS=Mus musculus GN=Stim1 PE=1 SV=2		5.839416	2	3	2	77.518	6.54	1500000	1900000		11.29958
Q9D0E1	Heterogeneous nuclear ribonucleoprotein M OS=Mus musculus GN=Hnrnpm PE=1 SV=3		32.92181	20	85	20	77.597	8.63	26000000	27000000	27000000	249.4814

Q8BY02	NF-kappa-B-repressing factor OS=Mus musculus GN=Nkrf PE=2 SV=3		1.15942	1	1	1	77.69	8.97			440000	2.357163
Q14C51	Pentatricopeptide repeat domain-containing protein 3, mitochondrial OS=Mus musculus GN=Ptcd3 PE=1 SV=2		2.335766	1	1	1	77.747	5.88	790000			3.380072
Q8CIB5	Fermitin family homolog 2 OS=Mus musculus GN=Fermt2 PE=1 SV=1		8.235294	5	11	5	77.751	6.7	4200000	3700000	4300000	30.68808
P53351	Serine/threonine-protein kinase PLK2 OS=Mus musculus GN=Plk2 PE=1 SV=1		3.812317	1	1	1	77.762	8.24		230000		2.366941
P41216	Long-chain-fatty-acid--CoA ligase 1 OS=Mus musculus GN=Acsl1 PE=1 SV=2		5.722461	4	10	3	77.901	7.15	1200000	1200000	1300000	25.33738
Q80U04	E3 ubiquitin-protein ligase Praja-2 OS=Mus musculus GN=Pja2 PE=1 SV=2		3.253182	1	1	1	77.91	4.44				2.915702
Q91WC3	Long-chain-fatty-acid--CoA ligase 6 OS=Mus musculus GN=Acsl6 PE=1 SV=1		1.578192	1	1	1	77.967	7.34	630000			2.511362
Q8C1A5	Thimet oligopeptidase OS=Mus musculus GN=Thop1 PE=1 SV=1		2.765648	1	3	1	77.976	6.06	210000	1800000	1600000	11.89054
Q08879	Fibulin-1 OS=Mus musculus GN=Fbln1 PE=1 SV=2		12.34043	6	13	6	77.981	5.16	2100000	2000000	1300000	40.78193
Q60865	Caprin-1 OS=Mus musculus GN=Caprin1 PE=1 SV=2		16.5488	8	23	8	78.121	5.25	16000000	15000000	12000000	80.76087
Q8R550	SH3 domain-containing kinase-binding protein 1 OS=Mus musculus GN=Sh3kbp1 PE=1 SV=1		21.01551	10	18	10	78.122	7.55	3300000	2800000	1900000	56.84227
Q80VY9	Putative ATP-dependent RNA helicase DHX33 OS=Mus musculus GN=Dhx33 PE=1 SV=1		2.005731	1	2	1	78.297	8.94		410000	460000	4.872038
P63318	Protein kinase C gamma type OS=Mus musculus GN=Prkcg PE=1 SV=1		13.62984	6	9	5	78.307	7.46	3500000	1300000	2100000	31.27484
A1L314	Macrophage-expressed gene 1 protein OS=Mus musculus GN=Mpeg1 PE=1 SV=1		2.664797	1	1	1	78.34	5.92			900000	2.915333
P57716	Nicastrin OS=Mus musculus GN=Ncstn PE=1 SV=3		3.248588	2	4	2	78.443	6.09	1900000		1300000	9.550573
P47856	Glutamine--fructose-6-phosphate aminotransferase [isomerizing] 1 OS=Mus musculus GN=Gfpt1 PE=1 SV=3		10.47346	6	13	5	78.489	6.84	4900000	4900000	4000000	36.78134
Q9ESJ4	NCK-interacting protein with SH3 domain OS=Mus musculus GN=Nckipsd PE=1 SV=2		2.10084	1	1	1	78.523	6.05	250000			2.70954
Q8BKT7	THO complex subunit 5 homolog OS=Mus musculus GN=Thoc5 PE=1 SV=2		2.196193	1	3	1	78.636	7.12	1100000	890000	1200000	8.87803
Q810V0	U3 small nucleolar ribonucleoprotein protein MPP10 OS=Mus musculus GN=Mphosph10 PE=1 SV=2		6.314244	3	6	3	78.687	4.87	1400000	900000	1700000	16.85274
Q91YR5	Methyltransferase-like protein 13 OS=Mus musculus GN=Mettl13 PE=1 SV=1		1.432665	1	3	1	78.707	6.83	1400000	1100000	780000	6.7117
Q9JLQ2	ARF GTPase-activating protein GIT2 OS=Mus musculus GN=Git2 PE=1 SV=2		7.768362	4	8	4	78.717	7.72	900000	1300000	1100000	22.91591
Q9QYB5	Gamma-adducin OS=Mus musculus GN=Add3 PE=1 SV=2		12.03966	7	15	7	78.728	5.95	2600000	1700000	1500000	43.86063
P21958	Antigen peptide transporter 1 OS=Mus musculus GN=Tap1 PE=1 SV=3		22.51381	15	47	15	78.814	8.79	25000000	29000000	30000000	135.732
Q9Z0E0	Neurochondrin OS=Mus musculus GN=Ncdn PE=1 SV=1		7.407407	5	7	5	78.845	5.54	2800000	1700000	2000000	19.44297
Q9QUJ7	Long-chain-fatty-acid--CoA ligase 4 OS=Mus musculus GN=Acsl4 PE=1 SV=2		6.751055	4	7	3	79.026	8.28	1400000		1000000	18.59417
Q3UZ39	Leucine-rich repeat flightless-interacting protein 1 OS=Mus musculus GN=Lrrfip1 PE=1 SV=2		12.34568	5	9	5	79.201	4.82	1100000	1200000	1400000	30.05757
Q8K3H0	DCC-interacting protein 13-alpha OS=Mus musculus GN=Appl1 PE=1 SV=1		1.55587	1	1	1	79.278	5.41	1400000			2.385106
Q99MR8	Methylcrotonoyl-CoA carboxylase subunit alpha, mitochondrial OS=Mus musculus GN=Mccc1 PE=1 SV=2		2.09205	1	1	1	79.293	7.83	500000			1.988329
Q9CW46	Ribonucleoprotein PTB-binding 1 OS=Mus musculus GN=Raver1 PE=1 SV=2		12.70053	5	9	5	79.333	8.72	1700000	1600000	1400000	26.87974
D3YVF0	A-kinase anchor protein 5 OS=Mus musculus GN=Akap5 PE=1 SV=2		17.31544	2	3	2	79.35	4.75		2000000	1100000	13.24835
P51660	Peroxisomal multifunctional enzyme type 2 OS=Mus musculus GN=Hsd17b4 PE=1 SV=3		10.34014	6	14	6	79.432	8.57	4100000	5500000	5700000	41.73706
Q80VI1	E3 ubiquitin-protein ligase TRIM56 OS=Mus musculus GN=Trim56 PE=1 SV=1		8.446866	4	7	4	79.463	7.96	1000000	1900000	760000	20.20135
Q80UZ2	Protein SDA1 homolog OS=Mus musculus GN=Sdad1 PE=1 SV=1		4.657933	3	7	3	79.508	9.2	1700000	1600000	1900000	15.98673
Q99MK8	Beta-adrenergic receptor kinase 1 OS=Mus musculus GN=Adrbk1 PE=1 SV=2		4.354136	2	4	2	79.588	7.28	810000	890000	1600000	10.05059
Q91VX9	Transmembrane protein 168 OS=Mus musculus GN=Tmem168 PE=1 SV=1		2.439024	1	3	1	79.621	7.96	1000000	1100000		8.917758
P28740	Kinesin-like protein KIF2A OS=Mus musculus GN=Kif2a PE=1 SV=2		8.368794	5	12	5	79.707	6.73	3200000	2400000	2200000	30.79898
Q8K2F0	Bromodomain-containing protein 3 OS=Mus musculus GN=Brd3 PE=1 SV=2		1.790634	1	1	1	79.712	9.36		940000		3.057413
Q8BWW4	La-related protein 4 OS=Mus musculus GN=Larp4 PE=1 SV=2		10.57024	6	12	6	79.713	6.39	720000	2500000	2200000	31.19327
Q91VD9	NADH-ubiquinone oxidoreductase 75 kDa subunit, mitochondrial OS=Mus musculus GN=Ndufs1 PE=1 SV=2		20.22008	14	40	14	79.726	5.72	12000000	8100000	7000000	115.1429
Q5U3K5	Rab-like protein 6 OS=Mus musculus GN=Rabl6 PE=1 SV=2		6.068966	3	9	3	79.782	5.53	1800000	1500000	1100000	29.2067
O08529	Calpain-2 catalytic subunit OS=Mus musculus GN=Capn2 PE=1 SV=4		24.42857	14	39	14	79.822	4.96	8900000	6200000	5400000	102.0847
Q8BZ20	Poly [ADP-ribose] polymerase 12 OS=Mus musculus GN=Parp12 PE=1 SV=3		8.438819	4	6	4	79.866	8.54		1900000	2300000	17.57676
Q91ZA3	Propionyl-CoA carboxylase alpha chain, mitochondrial OS=Mus musculus GN=Pcca PE=1 SV=2		5.38674	3	8	3	79.871	7.25	2300000	1800000	1800000	23.14994
O35250	Exocyst complex component 7 OS=Mus musculus GN=Exoc7 PE=1 SV=2		3.873745	2	3	2	79.911	6.98		1900000	1600000	8.512765
O88967	ATP-dependent zinc metalloprotease YME1L1 OS=Mus musculus GN=Yme1l1 PE=1 SV=1		2.097902	1	1	1	79.978	8.97			620000	2.126961
Q5RJG1	Nucleolar protein 10 OS=Mus musculus GN=Nol10 PE=2 SV=1		1.455604	1	1	1	80.027	8.19		630000		1.916075
Q8BY89	Choline transporter-like protein 2 OS=Mus musculus GN=Slc44a2 PE=1 SV=2		2.691218	2	2	2	80.057	8.79	2300000	1500000		4.606938
P28741	Kinesin-like protein KIF3A OS=Mus musculus GN=Kif3a PE=1 SV=2		4.707561	3	5	3	80.12	6.54	530000	760000	590000	11.66743
Q9CQN1	Heat shock protein 75 kDa, mitochondrial OS=Mus musculus GN=Trap1 PE=1 SV=1		8.215297	5	17	4	80.159	6.68	1E+08	82000000	77000000	52.12011
Q61216	Double-strand break repair protein MRE11A OS=Mus musculus GN=Mre11a PE=1 SV=1		4.249292	3	7	3	80.173	6.01	1200000	1200000	1300000	16.96195
Q8BIJ7	RUN and FYVE domain-containing protein 1 OS=Mus musculus GN=Rufy1 PE=1 SV=1		1.825843	1	1	1	80.326	5.68		560000		2.06123
Q99PU5	Long-chain-fatty-acid--CoA ligase ACSBG1 OS=Mus musculus GN=Acsbg1 PE=1 SV=1		15.67268	8	17	8	80.374	5.94	3600000	2600000	1900000	49.88495
Q06335	Amyloid-like protein 2 OS=Mus musculus GN=Aplp2 PE=1 SV=4		2.545969	1	1	1	80.416	4.7			490000	2.526754

Q62108	Disks large homolog 4 OS=Mus musculus GN=Dlg4 PE=1 SV=1		5.110497	3	7	3	80.423	5.87	2900000	1000000	1000000	18.88441
Q9CZW4	Long-chain-fatty-acid--CoA ligase 3 OS=Mus musculus GN=Acsl3 PE=1 SV=2		5	3	8	2	80.441	8.54	3400000	3300000	2500000	23.09235
P59114	Phosphorylated CTD-interacting factor 1 OS=Mus musculus GN=Pcif1 PE=1 SV=1		2.266289	1	1	1	80.453	7.64			800000	2.056464
O70582	Arachidonate 12-lipoxygenase, 12R-type OS=Mus musculus GN=Alox12b PE=1 SV=1		1.28388	1	1	1	80.525	6.71				2.480427
Q91YK2	Ribosomal RNA processing protein 1 homolog B OS=Mus musculus GN=Rrp1b PE=1 SV=2		8.839779	4	8	4	80.532	9.72	1500000	1700000	1400000	22.81821
P46978	Dolichyl-diphosphooligosaccharide--protein glycosyltransferase subunit STT3A OS=Mus musculus GN=Stt3a PE=1 SV=1		9.64539	7	15	7	80.545	8.1	8600000	11000000	14000000	38.00268
Q8C267	Histone-lysine N-methyltransferase SETDB2 OS=Mus musculus GN=Setdb2 PE=2 SV=2		2.103787	1	1	1	80.585	8.02		260000		2.277014
Q9QYB8	Beta-adducin OS=Mus musculus GN=Add2 PE=1 SV=4		5.37931	3	6	3	80.591	6.21	4400000	3000000	1800000	16.07266
Q9QYC0	Alpha-adducin OS=Mus musculus GN=Add1 PE=1 SV=2		16.32653	9	18	9	80.596	5.9	8100000	3300000	5500000	52.54677
O89090	Transcription factor Sp1 OS=Mus musculus GN=Sp1 PE=1 SV=2		6.25	2	3	2	80.683	7.34		500000	360000	11.8671
Q9QUR6	Prolyl endopeptidase OS=Mus musculus GN=Prep PE=1 SV=1		1.830986	1	2	1	80.7	5.73	1600000	1300000		4.91121
Q9R1R2	Tripartite motif-containing protein 3 OS=Mus musculus GN=Trim3 PE=1 SV=1		1.478495	1	2	1	80.724	7.81	2100000	1600000		5.018698
Q8K4B0	Metastasis-associated protein MTA1 OS=Mus musculus GN=Mta1 PE=1 SV=1		7.692308	4	5	4	80.747	9.29		1300000	1300000	13.58732
A2AQ19	RNA polymerase-associated protein RTF1 homolog OS=Mus musculus GN=Rtf1 PE=1 SV=1		4.755245	3	6	3	80.748	8.16	2300000	2600000	1800000	17.74891
Q8BJW5	Nucleolar protein 11 OS=Mus musculus GN=Nol11 PE=2 SV=1		5.255878	3	4	3	80.766	5.92	540000	390000	1700000	9.19161
Q08943	FACT complex subunit SSRP1 OS=Mus musculus GN=Ssrp1 PE=1 SV=2		15.39548	10	21	10	80.81	6.76	7700000	8100000	8200000	58.09517
A2RSY6	TRMT1-like protein OS=Mus musculus GN=Trmt1l PE=1 SV=1		8.241758	3	5	3	80.81	7.5	1300000	1100000	2000000	20.78084
P97350	Plakophilin-1 OS=Mus musculus GN=Pkp1 PE=1 SV=1		3.021978	2	2	2	80.844	8.91			1200000	5.169135
Q64521	Glycerol-3-phosphate dehydrogenase, mitochondrial OS=Mus musculus GN=Gpd2 PE=1 SV=2		23.65887	15	33	15	80.902	6.61	9300000	5500000	6400000	94.32233
Q6PGF7	Exocyst complex component 8 OS=Mus musculus GN=Exoc8 PE=1 SV=1		6.284916	3	9	3	80.984	5.4	1100000	970000	1000000	27.73355
Q8BJI1	Sodium-dependent neutral amino acid transporter SLC6A17 OS=Mus musculus GN=Slc6a17 PE=1 SV=1		1.788171	1	1	1	81.018	6.23	1900000			3.595463
Q8C561	LMBR1 domain-containing protein 2 OS=Mus musculus GN=Lmbrd2 PE=1 SV=1		5.907781	3	5	3	81.049	7.34	3000000	1500000	1600000	14.13743
Q3TVI8	Pre-B-cell leukemia transcription factor-interacting protein 1 OS=Mus musculus GN=Pbxip1 PE=1 SV=2		9.903714	5	12	5	81.115	5.36	2100000	2000000	1600000	34.60725
Q61881	DNA replication licensing factor MCM7 OS=Mus musculus GN=Mcm7 PE=1 SV=1		15.02086	9	14	9	81.16	6.37	7300000	5300000	5400000	40.06036
Q91YJ5	Translation initiation factor IF-2, mitochondrial OS=Mus musculus GN=Mtif2 PE=1 SV=2		1.650619	1	1	1	81.238	7.24			720000	1.863673
P29533	Vascular cell adhesion protein 1 OS=Mus musculus GN=Vcam1 PE=1 SV=1		18.67388	10	20	10	81.265	5.3	4900000	2600000	2400000	57.30618
Q9ESN6	Tripartite motif-containing protein 2 OS=Mus musculus GN=Trim2 PE=1 SV=1		1.478495	1	3	1	81.394	6.96	2400000	1200000	1600000	7.837379
O35613	Death domain-associated protein 6 OS=Mus musculus GN=Daxx PE=1 SV=1		2.571042	1	3	1	81.439	4.7	1300000	1500000	1300000	8.457568
Q8R146	Acylamino-acid-releasing enzyme OS=Mus musculus GN=Apeh PE=1 SV=3		1.639344	1	1	1	81.529	5.59	700000			2.495932
Q8BJS4	SUN domain-containing protein 2 OS=Mus musculus GN=Sun2 PE=1 SV=3		9.98632	5	12	5	81.555	7.02	2400000	1200000	2100000	34.04624
P58404	Striatin-4 OS=Mus musculus GN=Strn4 PE=1 SV=2		5.263158	3	7	3	81.595	5.38	1600000	1500000	1300000	19.74063
Q69Z98	Serine/threonine-protein kinase BRSK2 OS=Mus musculus GN=Brsk2 PE=1 SV=2		1.904762	1	1	1	81.682	8.79	670000			2.92852
Q02257	Junction plakoglobin OS=Mus musculus GN=Jup PE=1 SV=3		11.81208	7	13	6	81.749	6.14	1900000		8000000	36.93432
Q8BH15	CCR4-NOT transcription complex subunit 10 OS=Mus musculus GN=Cnot10 PE=1 SV=1		6.182796	3	7	3	81.766	7.68	940000	920000	900000	22.35857
P48410	ATP-binding cassette sub-family D member 1 OS=Mus musculus GN=Abcd1 PE=1 SV=1		2.717391	1	4	1	81.807	8.7	100000	1500000	1000000	13.06758
Q9CZD3	Glycine--tRNA ligase OS=Mus musculus GN=Gars PE=1 SV=1		10.42524	7	14	7	81.826	6.65	3900000	5100000	2000000	35.81318
Q8K0V4	CCR4-NOT transcription complex subunit 3 OS=Mus musculus GN=Cnot3 PE=1 SV=1		2.52996	1	1	1	81.896	6.2				3.18332
Q921L5	Conserved oligomeric Golgi complex subunit 2 OS=Mus musculus GN=Cog2 PE=1 SV=2		2.599179	1	3	1	81.988	6.21	410000	550000	450000	9.240847
Q8C754	Vacuolar protein sorting-associated protein 52 homolog OS=Mus musculus GN=Vps52 PE=1 SV=1		1.936376	1	1	1	81.993	5.9			280000	1.805988
Q8VDQ9	Protein KRI1 homolog OS=Mus musculus GN=Kri1 PE=1 SV=3		1.704545	1	3	1	82.007	5.12	1200000	1100000	880000	9.184277
O35350	Calpain-1 catalytic subunit OS=Mus musculus GN=Capn1 PE=1 SV=1		17.25105	11	24	11	82.054	5.87	7800000	5900000	5400000	68.78632
Q99MJ9	ATP-dependent RNA helicase DDX50 OS=Mus musculus GN=Ddx50 PE=2 SV=1		2.043597	1	1	1	82.125	9.25			1200000	2.331188
Q8CHT1	Ephexin-1 OS=Mus musculus GN=Ngef PE=1 SV=1		1.549296	1	1	1	82.148	5.99	670000			2.714154
O55047	Serine/threonine-protein kinase tousled-like 2 OS=Mus musculus GN=Tlk2 PE=1 SV=2		1.392758	1	2	1	82.21	8.27	1100000	1500000		4.897829
Q8C156	Condensin complex subunit 2 OS=Mus musculus GN=Ncaph PE=1 SV=1		5.608755	2	2	2	82.251	4.96		1300000	2600000	7.451934
P49718	DNA replication licensing factor MCM5 OS=Mus musculus GN=Mcm5 PE=1 SV=1		15.68895	9	17	9	82.29	8.43	1600000	5800000	4200000	47.8766
O70494	Transcription factor Sp3 OS=Mus musculus GN=Sp3 PE=1 SV=2		3.57599	2	2	2	82.312	5.26	250000	1900000		6.424081
Q9QYE6	Golgin subfamily A member 5 OS=Mus musculus GN=Golga5 PE=1 SV=2		3.566529	1	3	1	82.318	6.23	2300000	2000000	2000000	12.9102
Q8BJ05	Zinc finger CCCH domain-containing protein 14 OS=Mus musculus GN=Zc3h14 PE=1 SV=1		2.857143	2	4	2	82.357	7.37	1400000	1700000	1500000	9.73915
O35691	Pinin OS=Mus musculus GN=Pnn PE=1 SV=4		19.86207	10	24	10	82.386	7.01	5900000	8900000	8300000	84.45313
Q91VR5	ATP-dependent RNA helicase DDX1 OS=Mus musculus GN=Ddx1 PE=1 SV=1		22.56757	13	30	13	82.448	7.21	12000000	10000000	11000000	77.85273
P97452	Ribosome biogenesis protein BOP1 OS=Mus musculus GN=Bop1 PE=1 SV=1		7.377049	4	6	4	82.494	6.28	270000	2200000	1300000	14.80319
Q61102	ATP-binding cassette sub-family B member 7, mitochondrial OS=Mus musculus GN=Abcb7 PE=1 SV=3		1.595745	1	3	1	82.529	9.32	4600000	4400000	3800000	7.874932

P46460	Vesicle-fusing ATPase OS=Mus musculus GN=Nsf PE=1 SV=2		27.95699	19	54	18	82.561	6.95	27000000	16000000	15000000	157.8413
Q9JIS5	Synaptic vesicle glycoprotein 2A OS=Mus musculus GN=Sv2a PE=1 SV=1		7.681941	4	12	4	82.594	5.57	6100000	3400000	3300000	37.03918
Q8K1M6	Dynamin-1-like protein OS=Mus musculus GN=Dnm1l PE=1 SV=2		12.26415	6	10	6	82.606	7.05	2300000	3100000	570000	30.47317
Q8BMS1	Trifunctional enzyme subunit alpha, mitochondrial OS=Mus musculus GN=Hadha PE=1 SV=1		15.85845	10	25	10	82.617	9.14	16000000	12000000	12000000	76.35916
P16332	Methylmalonyl-CoA mutase, mitochondrial OS=Mus musculus GN=Mut PE=1 SV=2		1.737968	1	3	1	82.792	6.89	2000000	1500000	1200000	8.760977
Q99LI7	Cleavage stimulation factor subunit 3 OS=Mus musculus GN=Cstf3 PE=1 SV=1		5.718271	4	7	4	82.824	8.12	2500000	2000000	2100000	17.29736
Q9Z2I0	LETM1 and EF-hand domain-containing protein 1, mitochondrial OS=Mus musculus GN=Letm1 PE=1 SV=1		13.14363	8	16	8	82.937	6.52	3600000	5400000	5600000	47.11817
Q80U70	Polycomb protein Suz12 OS=Mus musculus GN=Suz12 PE=1 SV=2		11.06613	5	8	5	82.974	8.81	1400000	850000	840000	24.22896
Q3UIR3	E3 ubiquitin-protein ligase DTX3L OS=Mus musculus GN=Dtx3l PE=1 SV=1		23.12834	11	36	11	82.991	8.09	4400000	3500000	3800000	100.9275
Q8BSY0	Aspartyl/asparaginyl beta-hydroxylase OS=Mus musculus GN=Asph PE=1 SV=1		22.80702	9	22	9	82.991	5.08	7300000	6900000	7900000	79.67094
P27641	X-ray repair cross-complementing protein 5 OS=Mus musculus GN=Xrcc5 PE=1 SV=4		9.289617	5	9	5	83.004	5.16	2500000	2400000	6500000	26.11947
A2A8Z1	Oxysterol-binding protein-related protein 9 OS=Mus musculus GN=Osbpl9 PE=1 SV=1		1.766304	1	1	1	83.057	6.07		940000		3.426275
Q8BLN5	Lanosterol synthase OS=Mus musculus GN=Lss PE=1 SV=2		1.773533	1	1	1	83.088	6.4				2.440705
Q8C167	Prolyl endopeptidase-like OS=Mus musculus GN=Prepl PE=1 SV=1		1.931034	1	1	1	83.141	6.65	500000			3.059724
Q8BH61	Coagulation factor XIII A chain OS=Mus musculus GN=F13a1 PE=1 SV=3		5.601093	3	7	3	83.155	5.92	11000000	9800000	6900000	24.99021
Q8BUK6	Protein Hook homolog 3 OS=Mus musculus GN=Hook3 PE=1 SV=2		10.02786	5	9	5	83.166	5.19	1600000	1300000	960000	26.9441
P11499	Heat shock protein HSP 90-beta OS=Mus musculus GN=Hsp90ab1 PE=1 SV=3		52.62431	38	186	24	83.229	5.03	2.3E+08	2.1E+08	2E+08	575.0457
Q8BUV3	Gephyrin OS=Mus musculus GN=Gphn PE=1 SV=2		6.371912	3	7	3	83.23	5.6	1800000	1300000	880000	21.59353
Q99LH1	Nucleolar GTP-binding protein 2 OS=Mus musculus GN=Gnl2 PE=1 SV=2		2.884615	2	3	2	83.294	9.2	620000	1200000	430000	7.297606
Q9D0R2	Threonine--tRNA ligase, cytoplasmic OS=Mus musculus GN=Tars PE=1 SV=2		19.9446	12	31	12	83.303	7.36	6400000	6100000	4000000	87.21594
P63154	Crooked neck-like protein 1 OS=Mus musculus GN=Crnkl1 PE=1 SV=1		5.072464	3	6	3	83.363	6.93	2100000	2500000	2000000	15.30808
Q9WUN2	Serine/threonine-protein kinase TBK1 OS=Mus musculus GN=Tbk1 PE=1 SV=1		8.641975	4	5	4	83.372	6.87	840000	410000		13.8437
P26450	Phosphatidylinositol 3-kinase regulatory subunit alpha OS=Mus musculus GN=Pik3r1 PE=1 SV=2		2.762431	2	2	2	83.465	6.28		540000	310000	4.298019
Q9WTX8	Mitotic spindle assembly checkpoint protein MAD1 OS=Mus musculus GN=Mad1l1 PE=1 SV=1		4.04463	2	2	2	83.49	5.66		350000	880000	4.409986
Q8K0D5	Elongation factor G, mitochondrial OS=Mus musculus GN=Gfm1 PE=1 SV=1		1.198402	1	3	1	83.496	6.92	1100000	860000	750000	6.793861
P16054	Protein kinase C epsilon type OS=Mus musculus GN=Prkce PE=1 SV=1		6.377205	3	4	3	83.507	7.03	1400000	470000	590000	9.660074
Q8K0T0	Reticulon-1 OS=Mus musculus GN=Rtn1 PE=1 SV=1		3.717949	3	8	2	83.521	4.58	6100000	8200000	4100000	20.03206
Q9R0E2	Procollagen-lysine,2-oxoglutarate 5-dioxygenase 1 OS=Mus musculus GN=Plod1 PE=1 SV=1		3.021978	2	3	2	83.542	6.54	3100000	3200000		6.869874
Q3V1T4	Prolyl 3-hydroxylase 1 OS=Mus musculus GN=P3h1 PE=1 SV=2		5.683356	3	5	3	83.598	5.14	2600000	2400000	1900000	14.48046
Q811U4	Mitofusin-1 OS=Mus musculus GN=Mfn1 PE=1 SV=3		2.024291	1	1	1	83.673	6.51			740000	2.705495
Q9Z175	Lysyl oxidase homolog 3 OS=Mus musculus GN=Loxl3 PE=2 SV=2		3.97878	2	3	2	83.686	7.01	680000	1500000		9.271298
Q61164	Transcriptional repressor CTCF OS=Mus musculus GN=Ctcf PE=1 SV=2		7.336957	3	7	3	83.692	6.9	1700000	1900000	1500000	23.52486
Q3V0M2	Leucine-rich repeat-containing protein 36 OS=Mus musculus GN=Lrrc36 PE=1 SV=2		1.059603	1	1	1	83.711	7.31			1200000	1.838497
Q8BHL5	Engulfment and cell motility protein 2 OS=Mus musculus GN=Elmo2 PE=1 SV=1		7.103825	3	8	2	83.833	5.95	2200000	2500000	1600000	24.10374
Q8CAQ8	MICOS complex subunit Mic60 OS=Mus musculus GN=Immt PE=1 SV=1		26.02378	17	46	17	83.848	6.61	26000000	20000000	21000000	138.7222
P83093	Stromal interaction molecule 2 OS=Mus musculus GN=Stim2 PE=1 SV=2		7.104558	3	5	3	83.872	6.79	360000	460000	1200000	16.6898
Q8BPU7	Engulfment and cell motility protein 1 OS=Mus musculus GN=Elmo1 PE=1 SV=2		10.59147	5	10	4	83.882	6.28	3000000	2000000	1200000	23.16076
Q6Y685	Transforming acidic coiled-coil-containing protein 1 OS=Mus musculus GN=Tacc1 PE=1 SV=1		5.684755	3	3	3	83.9	5.03	1400000	1300000		7.20843
Q99MD9	Nuclear autoantigenic sperm protein OS=Mus musculus GN=Nasp PE=1 SV=2		5.562743	4	4	4	83.903	4.37	1900000	1900000		10.16502
O35598	Disintegrin and metalloproteinase domain-containing protein 10 OS=Mus musculus GN=Adam10 PE=1 SV=2		3.604806	2	3	2	83.914	7.94	1200000	1200000	820000	8.584552
Q9ERG0	LIM domain and actin-binding protein 1 OS=Mus musculus GN=Lima1 PE=1 SV=3		12.6162	6	13	6	84.008	6.6	9200000	8800000	2700000	40.07147
Q9D2N4	Dystrobrevin alpha OS=Mus musculus GN=Dtna PE=1 SV=2		3.08311	2	2	2	84.014	6.76	990000	1600000		7.128868
Q9JLM8	Serine/threonine-protein kinase DCLK1 OS=Mus musculus GN=Dclk1 PE=1 SV=1		1.719577	1	3	1	84.101	8.87	2600000	2200000	2200000	9.723764
Q8CGC6	RNA-binding protein 28 OS=Mus musculus GN=Rbm28 PE=1 SV=4		5.066667	3	3	3	84.162	9.54			1100000	6.820519
Q8R2M2	Deoxynucleotidyltransferase terminal-interacting protein 2 OS=Mus musculus GN=Dnttip2 PE=1 SV=1		11.6095	4	11	4	84.226	6.44	920000	1400000	1500000	40.9229
Q03141	MAP/microtubule affinity-regulating kinase 3 OS=Mus musculus GN=Mark3 PE=1 SV=2		4.249668	2	5	1	84.337	9.51	1000000	660000		16.65119
Q9R0B9	Procollagen-lysine,2-oxoglutarate 5-dioxygenase 2 OS=Mus musculus GN=Plod2 PE=1 SV=2		3.527815	2	5	2	84.435	6.89	720000	1200000	1100000	14.29247
Q571I9	Aldehyde dehydrogenase family 16 member A1 OS=Mus musculus GN=Aldh16a1 PE=1 SV=2		3.117207	2	5	2	84.703	6.32	1300000	1200000	1000000	14.40554
Q924C6	Lysyl oxidase homolog 4 OS=Mus musculus GN=Loxl4 PE=2 SV=2		1.585205	1	1	1	84.724	7.99				3.05384
P07901	Heat shock protein HSP 90-alpha OS=Mus musculus GN=Hsp90aa1 PE=1 SV=4		43.92906	32	122	20	84.735	5.01	72000000	57000000	53000000	366.3012
Q8R1F1	Niban-like protein 1 OS=Mus musculus GN=Fam129b PE=1 SV=2		2.803738	2	2	2	84.765	5.94		2100000		4.264666
Q9R0E1	Procollagen-lysine,2-oxoglutarate 5-dioxygenase 3 OS=Mus musculus GN=Plod3 PE=1 SV=1		5.398111	3	7	3	84.869	6.23	1300000	3600000	3700000	21.37901
P51125	Calpastatin OS=Mus musculus GN=Cast PE=1 SV=2		1.649746	1	2	1	84.871	5.52		930000		6.947218

Q8CEC0	Nuclear pore complex protein Nup88 OS=Mus musculus GN=Nup88 PE=1 SV=1		3.718459	2	3	2	84.884	6.27		1100000	1100000	8.256951
Q00PI9	Heterogeneous nuclear ribonucleoprotein U-like protein 2 OS=Mus musculus GN=Hnrnpul2 PE=1 SV=2		24.83221	14	53	14	84.888	4.89	16000000	15000000	13000000	156.4319
P04186	Complement factor B OS=Mus musculus GN=Cfb PE=1 SV=2		1.576873	1	1	1	84.951	7.37			1200000	2.580644
P11835	Integrin beta-2 OS=Mus musculus GN=Itgb2 PE=1 SV=2		28.40467	17	40	16	84.97	7.12	12000000	13000000	9300000	124.3487
P47713	Cytosolic phospholipase A2 OS=Mus musculus GN=Pla2g4a PE=1 SV=1		5.347594	4	8	4	85.168	5.43	1600000	1900000	1700000	19.84805
Q8C1D8	Protein IWS1 homolog OS=Mus musculus GN=Iws1 PE=1 SV=1		4.699739	3	9	3	85.197	4.67	1500000	1500000	1200000	22.68905
P47857	ATP-dependent 6-phosphofructokinase, muscle type OS=Mus musculus GN=Pfkm PE=1 SV=3		7.948718	5	11	4	85.215	8	1800000	820000	2600000	31.04393
Q61771	Kinesin-like protein KIF3B OS=Mus musculus GN=Kif3b PE=1 SV=1		3.212851	2	2	2	85.236	7.69	950000			4.710132
Q68FF6	ARF GTPase-activating protein GIT1 OS=Mus musculus GN=Git1 PE=1 SV=1		7.662338	4	10	4	85.247	6.93	2200000	1800000	2300000	26.9008
P12382	ATP-dependent 6-phosphofructokinase, liver type OS=Mus musculus GN=Pfkl PE=1 SV=4		5.641026	4	11	2	85.305	7.17		1400000	1800000	34.64369
Q9WV70	Nucleolar complex protein 2 homolog OS=Mus musculus GN=Noc2l PE=1 SV=2		4.685408	3	5	3	85.371	5.97	1300000	1100000	1700000	16.54119
Q1HFZ0	tRNA (cytosine(34)-C(5))-methyltransferase OS=Mus musculus GN=Nsun2 PE=1 SV=2		8.190225	3	3	3	85.397	6.58		1400000		8.628748
Q9WUA3	ATP-dependent 6-phosphofructokinase, platelet type OS=Mus musculus GN=Pfkp PE=1 SV=1		10.71429	7	21	5	85.4	7.11	5500000	4700000	4200000	63.32037
Q99KI0	Aconitate hydratase, mitochondrial OS=Mus musculus GN=Aco2 PE=1 SV=1		32.4359	21	68	21	85.41	7.93	41000000	35000000	41000000	197.597
Q02248	Catenin beta-1 OS=Mus musculus GN=Ctnnb1 PE=1 SV=1		7.554417	4	10	3	85.416	5.86	5000000	6900000	5400000	31.99909
P15379	CD44 antigen OS=Mus musculus GN=Cd44 PE=1 SV=3		6.55527	4	16	4	85.565	4.96	6100000	5500000	2100000	49.80819
E9Q5K9	YTH domain-containing protein 1 OS=Mus musculus GN=Ythdc1 PE=1 SV=2		1.494565	1	2	1	85.598	6.16	260000	880000		4.286761
Q9Z2B9	Ribosomal protein S6 kinase alpha-4 OS=Mus musculus GN=Rps6ka4 PE=1 SV=2		1.681759	1	1	1	85.598	8.27				2.835564
Q91WR3	Activating signal cointegrator 1 complex subunit 2 OS=Mus musculus GN=Ascc2 PE=1 SV=1		1.735648	1	1	1	85.599	5.03				1.9091
Q9QYH6	Melanoma-associated antigen D1 OS=Mus musculus GN=Maged1 PE=1 SV=1		1.935484	1	2	1	85.617	7.5		290000	170000	5.081599
Q8K1R3	Polyribonucleotide nucleotidyltransferase 1, mitochondrial OS=Mus musculus GN=Pnpt1 PE=1 SV=1		2.554278	2	3	2	85.628	8.03	850000		630000	6.735148
Q62351	Transferrin receptor protein 1 OS=Mus musculus GN=Tfrc PE=1 SV=1		6.159895	4	11	4	85.677	6.57	1500000	1800000	1300000	30.36131
Q8R3B1	1-phosphatidylinositol 4,5-bisphosphate phosphodiesterase delta-1 OS=Mus musculus GN=Plcd1 PE=1 SV=2		3.571429	2	2	2	85.819	6.21			630000	5.205724
Q921N6	Probable ATP-dependent RNA helicase DDX27 OS=Mus musculus GN=Ddx27 PE=1 SV=3		11.71053	7	17	7	85.885	9.25	2100000	2900000	3100000	45.02644
P13020	Gelsolin OS=Mus musculus GN=Gsn PE=1 SV=3		17.05128	9	24	9	85.888	6.18	16000000	13000000	9200000	77.33207
Q9WTR1	Transient receptor potential cation channel subfamily V member 2 OS=Mus musculus GN=Trpv2 PE=1 SV=2		5.952381	3	4	3	85.91	6.99	1400000		790000	13.7977
O55106	Striatin OS=Mus musculus GN=Strn PE=1 SV=2		9.358974	5	9	5	85.913	5.27	2100000	1600000	1400000	26.9081
Q99LI8	Hepatocyte growth factor-regulated tyrosine kinase substrate OS=Mus musculus GN=Hgs PE=1 SV=2		5.677419	4	7	4	85.961	6.16	1200000	1200000	1100000	18.45296
P06537	Glucocorticoid receptor OS=Mus musculus GN=Nr3c1 PE=1 SV=1		4.214559	2	2	2	85.998	6.35	180000			5.287182
Q01405	Protein transport protein Sec23A OS=Mus musculus GN=Sec23a PE=1 SV=2		7.45098	4	10	4	86.106	7.08		1800000	1800000	24.05905
Q80U63	Mitofusin-2 OS=Mus musculus GN=Mfn2 PE=1 SV=3		3.830911	2	4	2	86.133	6.77	1500000	690000	1100000	12.95248
Q3UVL4	Vacuolar protein sorting-associated protein 51 homolog OS=Mus musculus GN=Vps51 PE=1 SV=2		2.046036	1	1	1	86.133	6.24	510000			3.215105
Q8BGC0	HIV Tat-specific factor 1 homolog OS=Mus musculus GN=Htatsf1 PE=1 SV=1		14.53104	6	10	6	86.188	4.4	1100000	1200000	780000	25.61431
Q05512	Serine/threonine-protein kinase MARK2 OS=Mus musculus GN=Mark2 PE=1 SV=3		3.865979	2	5	1	86.252	9.67	750000	770000	370000	12.3915
Q8BVU0	Leucine-rich repeat and calponin homology domain-containing protein 3 OS=Mus musculus GN=Lrch3 PE=1 SV=3		7.197943	4	8	4	86.287	7.03	760000	830000	920000	20.60649
Q9D662	Protein transport protein Sec23B OS=Mus musculus GN=Sec23b PE=1 SV=1		5.47588	3	5	3	86.382	6.96	4200000	3400000	3200000	13.59238
Q6KAR6	Exocyst complex component 3 OS=Mus musculus GN=Exoc3 PE=1 SV=2		2.251656	1	1	1	86.4	6.2		350000		2.543067
Q6NZF1	Zinc finger CCCH domain-containing protein 11A OS=Mus musculus GN=Zc3h11a PE=1 SV=1		14.89899	9	22	9	86.438	8.13	2500000	4600000	5400000	67.92367
P35831	Tyrosine-protein phosphatase non-receptor type 12 OS=Mus musculus GN=Ptpn12 PE=1 SV=3		4.387097	2	7	2	86.473	5.99	1100000	1000000	450000	23.53666
Q3UA37	Glutamine-rich protein 1 OS=Mus musculus GN=Qrich1 PE=1 SV=1		2.316602	1	1	1	86.502	5.96				3.03476
Q8C0V0	Serine/threonine-protein kinase tousled-like 1 OS=Mus musculus GN=Tlk1 PE=1 SV=2		2.480418	1	1	1	86.57	8.79	56000			2.815978
Q8BZI0	Actin filament-associated protein 1-like 1 OS=Mus musculus GN=Afap1l1 PE=1 SV=1		2.473958	1	1	1	86.584	6.77				2.311008
O88351	Inhibitor of nuclear factor kappa-B kinase subunit beta OS=Mus musculus GN=Ikbkb PE=1 SV=1		4.887715	3	4	3	86.635	6.4	610000		560000	12.08256
P12023	Amyloid beta A4 protein OS=Mus musculus GN=App PE=1 SV=3		20.77922	11	25	11	86.667	4.79	4900000	4100000	3900000	74.0617
Q922K7	Probable 28S rRNA (cytosine-C(5))-methyltransferase OS=Mus musculus GN=Nop2 PE=1 SV=1		20.42875	11	24	11	86.699	9.22	5900000	5800000	6600000	74.4862
Q8CBY3	Leukocyte receptor cluster member 8 homolog OS=Mus musculus GN=Leng8 PE=1 SV=1		1.783439	1	1	1	86.712	9.32				2.788587
Q9D4H8	Cullin-2 OS=Mus musculus GN=Cul2 PE=1 SV=2		1.342282	1	1	1	86.821	7.01		2100000		2.006403
Q810J8	Zinc finger FYVE domain-containing protein 1 OS=Mus musculus GN=Zfyve1 PE=1 SV=2		1.930502	1	1	1	86.884	7.34		360000		2.293997
Q80YD1	ATP-dependent RNA helicase SUPV3L1, mitochondrial OS=Mus musculus GN=Supv3l1 PE=1 SV=1		2.053915	1	1	1	86.95	7.84	670000			2.78863
P52479	Ubiquitin carboxyl-terminal hydrolase 10 OS=Mus musculus GN=Usp10 PE=1 SV=3		3.156566	1	1	1	86.968	5.17		660000		3.591327
Q8K4I3	Rho guanine nucleotide exchange factor 6 OS=Mus musculus GN=Arhgef6 PE=1 SV=1		9.338521	5	10	3	86.996	5.87	970000		790000	28.66385
Q61410	cGMP-dependent protein kinase 2 OS=Mus musculus GN=Prkg2 PE=1 SV=1		1.83727	1	1	1	87.03	8.22		3300000		1.913679
Q99KC8	von Willebrand factor A domain-containing protein 5A OS=Mus musculus GN=Vwa5a PE=1 SV=2		19.04161	11	28	11	87.087	6.58	7900000	7600000	6400000	81.51134

Q9ERG2	Striatin-3 OS=Mus musculus GN=Strn3 PE=1 SV=1		11.55779	6	12	6	87.096	5.29	2900000	2700000	1900000	39.75012
Q8CB77	Transcription elongation factor B polypeptide 3 OS=Mus musculus GN=Tceb3 PE=1 SV=3		3.492885	2	4	2	87.108	9.61	730000	940000		10.06897
Q80W68	Kin of IRRE-like protein 1 OS=Mus musculus GN=Kirrel PE=1 SV=1		1.39417	1	3	1	87.122	5.92	820000	370000		7.350197
P42225	Signal transducer and activator of transcription 1 OS=Mus musculus GN=Stat1 PE=1 SV=1		33.37784	21	83	21	87.142	5.58	46000000	43000000	34000000	271.2705
Q6ZQK5	Arf-GAP with coiled-coil, ANK repeat and PH domain-containing protein 2 OS=Mus musculus GN=Acap2 PE=1 SV=2		1.818182	1	1	1	87.156	6.68	200000			3.087508
P27612	Phospholipase A-2-activating protein OS=Mus musculus GN=Plaa PE=1 SV=4		16.62469	9	18	9	87.166	6.14	2900000	2200000	2400000	57.65014
Q640M1	U3 small nucleolar RNA-associated protein 14 homolog A OS=Mus musculus GN=Utp14a PE=1 SV=1		10.95176	6	10	6	87.212	9.2	1600000	1700000	1600000	27.41562
Q9Z110	Delta-1-pyrroline-5-carboxylate synthase OS=Mus musculus GN=Aldh18a1 PE=1 SV=2		6.540881	4	9	4	87.212	7.55	3600000	3100000	4300000	26.24345
Q99K01	Pyridoxal-dependent decarboxylase domain-containing protein 1 OS=Mus musculus GN=Pdxdc1 PE=1 SV=2		2.92249	2	5	2	87.281	5.48	2200000	1300000	840000	12.88169
E9PV24	Fibrinogen alpha chain OS=Mus musculus GN=Fga PE=1 SV=1		19.01141	14	46	14	87.375	6.11	16000000	18000000	17000000	134.0857
Q8K327	Chromosome alignment-maintaining phosphoprotein 1 OS=Mus musculus GN=Champ1 PE=1 SV=1		2.119701	1	1	1	87.506	7.85			790000	3.716861
Q3TCH7	Cullin-4A OS=Mus musculus GN=Cul4a PE=1 SV=1		4.084321	3	8	1	87.697	8.35	290000		630000	22.34386
Q8VHE0	Translocation protein SEC63 homolog OS=Mus musculus GN=Sec63 PE=1 SV=4		11.71053	7	16	7	87.815	5.38	1800000	3000000	2600000	46.89792
O70309	Integrin beta-5 OS=Mus musculus GN=Itgb5 PE=1 SV=2		4.636591	3	6	2	87.851	6.16	4000000	570000	1900000	13.96627
Q8VEK3	Heterogeneous nuclear ribonucleoprotein U OS=Mus musculus GN=Hnrnpu PE=1 SV=1		25.25	17	60	17	87.863	6.24	71000000	72000000	67000000	198.2605
P42227	Signal transducer and activator of transcription 3 OS=Mus musculus GN=Stat3 PE=1 SV=2		19.87013	11	28	11	87.997	6.3	5400000	5400000	4200000	89.05754
O54692	Centromere/kinetochore protein zw10 homolog OS=Mus musculus GN=Zw10 PE=1 SV=3		2.310655	1	3	1	88.006	5.92		640000	280000	9.19981
Q7JJ13	Bromodomain-containing protein 2 OS=Mus musculus GN=Brd2 PE=1 SV=1		4.135338	2	4	2	88.012	9.13	670000	520000	760000	13.82061
P55288	Cadherin-11 OS=Mus musculus GN=Cdh11 PE=1 SV=1		1.758794	1	1	1	88.058	4.89			3200000	2.49717
P09055	Integrin beta-1 OS=Mus musculus GN=Itgb1 PE=1 SV=1		20.80201	16	45	16	88.173	5.94	13000000	15000000	11000000	134.3998
P97742	Carnitine O-palmitoyltransferase 1, liver isoform OS=Mus musculus GN=Cpt1a PE=1 SV=4		5.174644	2	3	2	88.195	8.62	1100000		1400000	8.7673
Q8C4J7	Transducin beta-like protein 3 OS=Mus musculus GN=Tbl3 PE=2 SV=1		8.2397	5	9	5	88.21	6.81	2300000	1600000	2200000	28.32661
Q8VDF2	E3 ubiquitin-protein ligase UHRF1 OS=Mus musculus GN=Uhrf1 PE=1 SV=2		6.393862	4	8	4	88.248	8.31	890000	1600000	1500000	20.11899
A2AR02	Peptidyl-prolyl cis-trans isomerase G OS=Mus musculus GN=Ppig PE=1 SV=1		5.053191	3	7	3	88.272	10.27	2300000	1900000	2500000	16.35692
Q3TDD9	Protein phosphatase 1 regulatory subunit 21 OS=Mus musculus GN=Ppp1r21 PE=1 SV=2		3.076923	2	3	2	88.282	6.9		480000	1400000	7.707717
Q9Z2G6	Protein sel-1 homolog 1 OS=Mus musculus GN=Sel1l PE=1 SV=2		3.670886	2	6	2	88.285	5.57	4900000	4000000	3800000	21.05046
O35218	Cleavage and polyadenylation specificity factor subunit 2 OS=Mus musculus GN=Cpsf2 PE=1 SV=1		4.475703	2	4	2	88.327	5.11	3900000	3500000	2000000	14.31586
Q80Y56	Rabenosyn-5 OS=Mus musculus GN=Rbsn PE=1 SV=1		1.660281	1	2	1	88.436	5.3	740000		650000	5.784811
Q8K4Z5	Splicing factor 3A subunit 1 OS=Mus musculus GN=Sf3a1 PE=1 SV=1		16.81416	13	34	13	88.489	5.22	6100000	4100000	5900000	96.36809
O08739	AMP deaminase 3 OS=Mus musculus GN=Ampd3 PE=1 SV=2		5.221932	3	5	3	88.596	7.33	2200000	1300000	1300000	13.37779
Q8R1U1	Conserved oligomeric Golgi complex subunit 4 OS=Mus musculus GN=Cog4 PE=1 SV=1		1.019108	1	1	1	88.604	5.15		380000		2.21098
O70496	H(+)/Cl(-) exchange transporter 7 OS=Mus musculus GN=Clcn7 PE=1 SV=1		2.366127	1	3	1	88.656	7.27	1000000	930000	820000	11.34441
Q91W50	Cold shock domain-containing protein E1 OS=Mus musculus GN=Csde1 PE=1 SV=1		15.91479	10	24	10	88.735	6.37	3200000	4000000	3100000	67.66236
Q3B7Z2	Oxysterol-binding protein 1 OS=Mus musculus GN=Osbp PE=1 SV=3		3.602484	1	1	1	88.741	7.2			740000	3.509276
Q8BTT6	Digestive organ expansion factor homolog OS=Mus musculus GN=Diexf PE=2 SV=2		4.274611	2	3	2	88.762	5.74		640000	650000	9.669814
Q62318	Transcription intermediary factor 1-beta OS=Mus musculus GN=Trim28 PE=1 SV=3		25.77938	12	50	12	88.791	5.77	21000000	18000000	24000000	177.7398
Q9JLV5	Cullin-3 OS=Mus musculus GN=Cul3 PE=1 SV=1		7.8125	5	6	5	88.891	8.46	3600000	2600000	2000000	16.38105
Q14DK4	Glycerol-3-phosphate acyltransferase 2, mitochondrial OS=Mus musculus GN=Gpat2 PE=1 SV=3		1.872659	1	1	1	89.092	6.99	2000000			2.010871
Q9EQC5	N-terminal kinase-like protein OS=Mus musculus GN=Scyl1 PE=1 SV=1		3.846154	2	5	2	89.104	6.44	4200000	1500000	4200000	15.87103
Q01853	Transitional endoplasmic reticulum ATPase OS=Mus musculus GN=Vcp PE=1 SV=4		43.17618	28	98	27	89.266	5.26	44000000	35000000	37000000	321.7463
A2BE28	Ribosomal biogenesis protein LAS1L OS=Mus musculus GN=Las1l PE=1 SV=1		5.541237	2	2	2	89.359	4.44	580000		190000	5.468674
Q9QUN7	Toll-like receptor 2 OS=Mus musculus GN=Tlr2 PE=1 SV=1		1.147959	1	1	1	89.392	6.64	210000			2.147707
P25976	Nucleolar transcription factor 1 OS=Mus musculus GN=Ubtf PE=1 SV=1		11.37255	7	12	7	89.453	5.76	7600000	6400000	5700000	34.54602
Q8JZQ2	AFG3-like protein 2 OS=Mus musculus GN=Afg3l2 PE=1 SV=1		11.84539	9	17	9	89.463	8.6	1700000	3500000	2100000	50.3158
Q8CCJ3	E3 UFM1-protein ligase 1 OS=Mus musculus GN=Ufl1 PE=1 SV=2		7.440101	5	9	5	89.464	6.67	2400000	890000	1500000	22.34
Q6R891	Neurabin-2 OS=Mus musculus GN=Ppp1r9b PE=1 SV=1		6.119951	4	12	4	89.466	4.92	4500000	3200000	3000000	37.18115
Q9WTX6	Cullin-1 OS=Mus musculus GN=Cul1 PE=1 SV=1		3.221649	2	5	2	89.635	8	1500000	1800000	2000000	15.53294
Q9JLF6	Protein-glutamine gamma-glutamyltransferase K OS=Mus musculus GN=Tgm1 PE=1 SV=2		9.815951	6	14	6	89.769	6.51	5300000	2700000	2700000	40.99842
O35601	FYN-binding protein OS=Mus musculus GN=Fyb PE=1 SV=2		7.692308	4	9	4	90.001	6.98	1900000	2000000	1600000	28.86977
Q6PFQ7	Ras GTPase-activating protein 4 OS=Mus musculus GN=Rasa4 PE=1 SV=1		1.745636	1	1	1	90.004	8		360000		2.319217
P07742	Ribonucleoside-diphosphate reductase large subunit OS=Mus musculus GN=Rrm1 PE=1 SV=2		3.409091	2	2	2	90.153	6.7	1900000		1400000	5.75182
Q8BX02	KN motif and ankyrin repeat domain-containing protein 2 OS=Mus musculus GN=Kank2 PE=1 SV=1		3.795967	2	3	2	90.19	5.55		300000	710000	9.566786
Q9Z0M6	CD97 antigen OS=Mus musculus GN=Cd97 PE=1 SV=2		2.07824	1	3	1	90.354	7.39	240000	390000	120000	8.552752

Q8C9X6	Enhancer of polycomb homolog 1 OS=Mus musculus GN=Epc1 PE=1 SV=1		2.214022	1	1	1	90.355	8.34		95000		0
Q9DC23	DnaJ homolog subfamily C member 10 OS=Mus musculus GN=Dnajc10 PE=1 SV=2		4.287516	3	6	3	90.525	6.96	1900000	1200000	1200000	13.86304
Q9CS00	Cactin OS=Mus musculus GN=Cactin PE=1 SV=2		1.943005	1	3	1	90.606	9.79	1200000	1400000	1200000	8.010291
P20918	Plasminogen OS=Mus musculus GN=Plg PE=1 SV=3		16.25616	9	20	9	90.749	6.6	2500000	2000000	2100000	57.09129
Q8VCC9	Spondin-1 OS=Mus musculus GN=Spon1 PE=1 SV=1		2.106568	1	2	1	90.761	6.02	710000		690000	6.324765
Q04750	DNA topoisomerase 1 OS=Mus musculus GN=Top1 PE=1 SV=2		15.25424	10	27	10	90.819	9.33	8600000	9800000	8800000	79.96385
Q9Z315	U4/U6.U5 tri-snRNP-associated protein 1 OS=Mus musculus GN=Sart1 PE=1 SV=1		22.95285	14	26	14	90.83	5.82	3400000	3300000	3300000	82.63136
Q9EQ32	Phosphoinositide 3-kinase adapter protein 1 OS=Mus musculus GN=Pik3ap1 PE=1 SV=1		3.575832	2	5	2	90.871	5.17	960000	770000	1000000	13.35825
O35286	Pre-mRNA-splicing factor ATP-dependent RNA helicase DHX15 OS=Mus musculus GN=Dhx15 PE=1 SV=2		15.59748	11	22	11	90.949	7.46	11000000	11000000	13000000	63.46767
Q9Z218	Dipeptidyl aminopeptidase-like protein 6 OS=Mus musculus GN=Dpp6 PE=1 SV=1		3.9801	3	4	3	91.203	6.68	1200000			10.41616
P70188	Kinesin-associated protein 3 OS=Mus musculus GN=Kifap3 PE=1 SV=1		1.513241	1	2	1	91.233	5.11	900000		720000	5.063601
P22892	AP-1 complex subunit gamma-1 OS=Mus musculus GN=Ap1g1 PE=1 SV=3		5.596107	3	8	3	91.292	6.8	2000000	1600000	2000000	20.41978
Q8JZQ9	Eukaryotic translation initiation factor 3 subunit B OS=Mus musculus GN=Eif3b PE=1 SV=1		33.12578	18	46	18	91.313	5.02	17000000	16000000	14000000	144.647
Q61165	Sodium/hydrogen exchanger 1 OS=Mus musculus GN=Slc9a1 PE=1 SV=1		1.585366	1	1	1	91.41	7.12	1100000			3.071238
P24788	Cyclin-dependent kinase 11B OS=Mus musculus GN=Cdk11b PE=1 SV=2		2.295918	2	4	2	91.458	5.39	1500000	1400000	1100000	8.686164
P70218	Mitogen-activated protein kinase kinase kinase kinase 1 OS=Mus musculus GN=Map4k1 PE=1 SV=1		2.297461	1	1	1	91.476	7.85		280000		3.571058
P25206	DNA replication licensing factor MCM3 OS=Mus musculus GN=Mcm3 PE=1 SV=2		24.38424	13	30	13	91.489	5.55	6900000	6600000	4800000	100.1183
Q8BMQ2	General transcription factor 3C polypeptide 4 OS=Mus musculus GN=Gtf3c4 PE=1 SV=2		2.815177	2	3	2	91.554	6.73	420000	630000	510000	8.666628
Q91W96	Anaphase-promoting complex subunit 4 OS=Mus musculus GN=Anapc4 PE=1 SV=1		1.486989	1	3	1	91.649	5.4	1100000	980000	1200000	6.959923
Q9EQH3	Vacuolar protein sorting-associated protein 35 OS=Mus musculus GN=Vps35 PE=1 SV=1		10.80402	8	24	8	91.655	5.44	19000000	18000000	18000000	84.74142
Q08509	Epidermal growth factor receptor kinase substrate 8 OS=Mus musculus GN=Eps8 PE=1 SV=2		11.93666	8	20	8	91.68	7.65	1900000	1800000	2000000	61.31636
P40645	Transcription factor SOX-6 OS=Mus musculus GN=Sox6 PE=1 SV=2		1.330109	1	1	1	91.746	7.78			440000	1.917879
Q80UM7	Mannosyl-oligosaccharide glucosidase OS=Mus musculus GN=Mogs PE=1 SV=1		1.438849	1	2	1	91.774	9	1100000		740000	4.467278
A2A6Q5	Cell division cycle protein 27 homolog OS=Mus musculus GN=Cdc27 PE=1 SV=1		2.181818	1	1	1	91.784	7.02	120000			2.910261
Q61548	Clathrin coat assembly protein AP180 OS=Mus musculus GN=Snap91 PE=1 SV=1		4.106548	4	7	3	91.794	4.88	5600000	3900000	2800000	16.08554
Q3UYV9	Nuclear cap-binding protein subunit 1 OS=Mus musculus GN=Ncbp1 PE=1 SV=2		6.075949	3	8	3	91.868	6.64	4200000	3300000	3500000	23.3226
Q9DBT5	AMP deaminase 2 OS=Mus musculus GN=Ampd2 PE=1 SV=1		2.506266	2	2	2	91.965	6.23	1900000		720000	4.815529
Q61072	Disintegrin and metalloproteinase domain-containing protein 9 OS=Mus musculus GN=Adam9 PE=1 SV=2		5.088757	3	5	3	92.02	7.47	530000	1100000	890000	14.23863
Q05722	Collagen alpha-1(IX) chain OS=Mus musculus GN=Col9a1 PE=2 SV=2		1.411509	1	3	1	92.035	8.95	1500000	1800000	1300000	8.004802
Q5SWD9	Pre-rRNA-processing protein TSR1 homolog OS=Mus musculus GN=Tsr1 PE=1 SV=1		3.237858	2	3	2	92.047	6.99	880000	780000		8.979152
P15209	BDNF/NT-3 growth factors receptor OS=Mus musculus GN=Ntrk2 PE=1 SV=1		1.948843	1	2	1	92.074	6.55	1000000	500000		6.067244
Q6ZQF7	Protein Jade-2 OS=Mus musculus GN=Jade2 PE=1 SV=2		2.050663	1	1	1	92.116	5.77				2.289115
Q5DTU0	Actin filament-associated protein 1-like 2 OS=Mus musculus GN=Afap1l2 PE=1 SV=2		5.575758	3	4	3	92.118	5.44	1400000	1500000	620000	10.58325
Q6A068	Cell division cycle 5-like protein OS=Mus musculus GN=Cdc5l PE=1 SV=2		10.84788	6	17	6	92.133	8.02	7100000	6400000	5800000	56.31
Q3UIA2	Rho GTPase-activating protein 17 OS=Mus musculus GN=Arhgap17 PE=1 SV=1		5.555556	4	8	4	92.145	7.56	1700000	2300000	1700000	22.74597
Q91YE7	RNA-binding protein 5 OS=Mus musculus GN=Rbm5 PE=1 SV=1		6.01227	4	7	4	92.254	6.21	940000	820000	620000	18.83301
Q8R502	Volume-regulated anion channel subunit LRRC8C OS=Mus musculus GN=Lrrc8c PE=1 SV=1		7.596513	5	9	5	92.312	7.81	1500000	3400000	3000000	23.67236
P08113	Endoplasmin OS=Mus musculus GN=Hsp90b1 PE=1 SV=2		41.02244	32	113	30	92.418	4.82	1.1E+08	98000000	92000000	355.4729
Q8VD04	GRIP1-associated protein 1 OS=Mus musculus GN=Gripap1 PE=1 SV=1		2.233251	1	1	1	92.658	5.25	480000			3.656302
Q80Y81	Zinc phosphodiesterase ELAC protein 2 OS=Mus musculus GN=Elac2 PE=1 SV=1		3.008424	1	1	1	92.66	7.34	310000			2.931156
Q8BUY8	G-protein coupled receptor-associated sorting protein 2 OS=Mus musculus GN=Gprasp2 PE=1 SV=2		2.058111	1	1	1	92.744	5.11			390000	2.709587
Q6ZQ88	Lysine-specific histone demethylase 1A OS=Mus musculus GN=Kdm1a PE=1 SV=2		1.289566	1	2	1	92.793	6.52	1700000	2000000		3.869003
P97311	DNA replication licensing factor MCM6 OS=Mus musculus GN=Mcm6 PE=1 SV=1		12.42387	9	22	9	92.809	5.45	6300000	5800000	6200000	69.20172
P97484	Leukocyte immunoglobulin-like receptor subfamily B member 3 OS=Mus musculus GN=Lilrb3 PE=1 SV=1		1.545779	1	1	1	92.995	6.87		550000		2.444864
Q8R313	Exocyst complex component 6 OS=Mus musculus GN=Exoc6 PE=1 SV=2		1.371571	1	1	1	93.017	6.15			330000	2.292065
Q8BRN9	Coiled-coil and C2 domain-containing protein 1B OS=Mus musculus GN=Cc2d1b PE=1 SV=1		5.542453	3	4	3	93.034	5.41	510000		660000	13.21634
Q91WG4	Elongator complex protein 2 OS=Mus musculus GN=Elp2 PE=1 SV=1		3.008424	1	2	1	93.034	5.82		490000		7.201355
Q62009	Periostin OS=Mus musculus GN=Postn PE=1 SV=2		5.011933	3	6	3	93.085	7.53	2900000	2800000	2500000	16.32282
O35136	Neural cell adhesion molecule 2 OS=Mus musculus GN=Ncam2 PE=1 SV=1		7.168459	5	9	5	93.145	6.07	2700000	900000	700000	28.05899
Q8VI84	Nucleolar complex protein 3 homolog OS=Mus musculus GN=Noc3l PE=2 SV=2		3.593556	1	1	1	93.153	9.09		1100000		4.760808
Q3TDQ1	Dolichyl-diphosphooligosaccharide--protein glycosyltransferase subunit STT3B OS=Mus musculus GN=Stt3b PE=1 SV=2		5.467801	4	10	4	93.185	8.95	6400000	6900000	6300000	27.01461
A2AJI0	MAP7 domain-containing protein 1 OS=Mus musculus GN=Map7d1 PE=1 SV=1		1.77305	1	2	1	93.22	10.15	830000	1400000		5.4966
Q8BJ71	Nuclear pore complex protein Nup93 OS=Mus musculus GN=Nup93 PE=1 SV=1		11.35531	7	16	7	93.222	5.72	2600000	3800000	2800000	46.56623

Q921C5	Protein bicaudal D homolog 2 OS=Mus musculus GN=Bicd2 PE=1 SV=1		1.463415	1	2	1	93.334	5.44	620000		690000	5.027482
Q99LG0	Ubiquitin carboxyl-terminal hydrolase 16 OS=Mus musculus GN=Usp16 PE=1 SV=2		3.636364	2	5	2	93.376	6.18	470000	760000	540000	14.4128
P70175	Disks large homolog 3 OS=Mus musculus GN=Dlg3 PE=1 SV=1		3.180212	2	2	2	93.424	6.79	420000	370000		5.369985
Q9JIK5	Nucleolar RNA helicase 2 OS=Mus musculus GN=Ddx21 PE=1 SV=3		26.90952	21	64	21	93.493	9.11	28000000	30000000	30000000	199.4406
Q3UKC1	Tax1-binding protein 1 homolog OS=Mus musculus GN=Tax1bp1 PE=1 SV=2		1.351351	1	1	1	93.572	5.33		640000		2.203323
P18052	Receptor-type tyrosine-protein phosphatase alpha OS=Mus musculus GN=Ptpra PE=1 SV=3		3.136309	2	3	2	93.638	7.05	1200000	580000		6.559162
P16879	Tyrosine-protein kinase Fes/Fps OS=Mus musculus GN=Fes PE=1 SV=2		1.459854	1	1	1	93.72	6.8			410000	2.764599
Q6P2K6	Serine/threonine-protein phosphatase 4 regulatory subunit 3A OS=Mus musculus GN=Ppp4r3a PE=1 SV=1		5.487805	3	5	3	93.783	4.89	1100000	1800000	700000	13.51751
Q3U821	WD repeat-containing protein 75 OS=Mus musculus GN=Wdr75 PE=1 SV=1		4.216867	3	7	3	93.977	6.18	1000000	1300000	810000	17.7807
Q61316	Heat shock 70 kDa protein 4 OS=Mus musculus GN=Hspa4 PE=1 SV=1		34.48276	21	72	21	94.073	5.24	15000000	12000000	12000000	230.5434
Q3TZZ7	Extended synaptotagmin-2 OS=Mus musculus GN=Esyt2 PE=1 SV=1		4.615385	3	5	3	94.081	7.75	850000	3000000	2900000	13.59788
Q99K10	Neuroligin-1 OS=Mus musculus GN=Nlgn1 PE=1 SV=2		2.846975	2	4	2	94.09	5.99	1500000	770000	660000	8.680275
Q80W00	Serine/threonine-protein phosphatase 1 regulatory subunit 10 OS=Mus musculus GN=Ppp1r10 PE=1 SV=1		5.18018	3	4	3	94.313	9.16	380000	480000	600000	11.10538
P48722	Heat shock 70 kDa protein 4L OS=Mus musculus GN=Hspa4l PE=1 SV=2		9.904535	5	15	5	94.322	5.74	3200000	2300000	2200000	51.51759
Q9DBY8	Nuclear valosin-containing protein-like OS=Mus musculus GN=Nvl PE=1 SV=1		7.836257	5	12	5	94.417	6.35	1900000	1700000	2200000	34.18116
Q7TSI3	Serine/threonine-protein phosphatase 6 regulatory subunit 1 OS=Mus musculus GN=Ppp6r1 PE=1 SV=1		3.38785	2	5	2	94.468	4.64	1000000	2000000	2600000	16.84539
Q8K310	Matrin-3 OS=Mus musculus GN=Matr3 PE=1 SV=1		17.8487	13	37	13	94.572	6.25	24000000	20000000	23000000	117.8966
Q922D4	Serine/threonine-protein phosphatase 6 regulatory subunit 3 OS=Mus musculus GN=Ppp6r3 PE=1 SV=1		8.412322	5	12	5	94.593	4.58	1400000	1700000	1200000	34.47278
Q9ER72	Cysteine--tRNA ligase, cytoplasmic OS=Mus musculus GN=Cars PE=1 SV=2		5.174489	3	4	3	94.8	6.76	1500000	1700000	1200000	10.32994
Q91XM9	Disks large homolog 2 OS=Mus musculus GN=Dlg2 PE=1 SV=2		2.934272	2	2	2	94.821	6.24	1300000	780000		5.762625
P35951	Low-density lipoprotein receptor OS=Mus musculus GN=Ldlr PE=1 SV=2		1.160093	1	1	1	94.885	5.02	310000			2.11833
Q6P542	ATP-binding cassette sub-family F member 1 OS=Mus musculus GN=Abcf1 PE=1 SV=1		15.05376	9	26	9	94.887	6.51	5100000	7200000	6100000	83.42025
Q8BPM2	Mitogen-activated protein kinase kinase kinase kinase 5 OS=Mus musculus GN=Map4k5 PE=1 SV=2		5.66706	3	6	3	94.985	7.83	1500000	920000	1600000	17.78116
Q60848	Lymphocyte-specific helicase OS=Mus musculus GN=Hells PE=1 SV=2		3.532278	2	3	2	95.065	8	760000	860000	410000	9.209435
Q8BZQ7	Anaphase-promoting complex subunit 2 OS=Mus musculus GN=Anapc2 PE=1 SV=2		1.911589	1	3	1	95.247	5.31	510000	800000	590000	7.28094
P58252	Elongation factor 2 OS=Mus musculus GN=Eef2 PE=1 SV=2		34.73193	31	128	30	95.253	6.83	96000000	97000000	86000000	396.6051
Q3UNH4	G protein-regulated inducer of neurite outgrowth 1 OS=Mus musculus GN=Gprin1 PE=1 SV=2		6.437768	4	6	4	95.437	7.93	1000000	630000		17.75328
Q9DBE9	pre-rRNA processing protein FTSJ3 OS=Mus musculus GN=Ftsj3 PE=1 SV=1		21.47971	13	29	13	95.474	8.38	5200000	5700000	6100000	87.29037
Q8C079	Striatin-interacting protein 1 OS=Mus musculus GN=Strip1 PE=1 SV=2		3.106332	1	1	1	95.524	6.25			990000	2.87466
Q9DBC3	Cap-specific mRNA (nucleoside-2'-O-)-methyltransferase 1 OS=Mus musculus GN=Cmtr1 PE=1 SV=1		7.407407	5	11	5	95.615	7.27	1400000	1900000	1600000	27.58194
Q8VC03	Echinoderm microtubule-associated protein-like 3 OS=Mus musculus GN=Eml3 PE=1 SV=1		3.67893	2	2	2	95.636	7.05	230000	190000		4.858675
Q61361	Brevican core protein OS=Mus musculus GN=Bcan PE=1 SV=2		20.9513	14	31	14	95.755	4.93	11000000	6900000	5800000	90.90433
P56399	Ubiquitin carboxyl-terminal hydrolase 5 OS=Mus musculus GN=Usp5 PE=1 SV=1		10.60606	6	13	6	95.772	5.01	3700000	2600000	2500000	39.46553
Q4KMM3	Oxidation resistance protein 1 OS=Mus musculus GN=Oxr1 PE=1 SV=3		8.083141	4	10	4	95.852	5.33	1800000	930000	520000	32.63655
P48193	Protein 4.1 OS=Mus musculus GN=Epb41 PE=1 SV=2		3.729604	2	2	2	95.853	5.6		1200000		6.094768
P08553	Neurofilament medium polypeptide OS=Mus musculus GN=Nefm PE=1 SV=4		26.06132	19	51	16	95.859	4.77	17000000	6700000	6700000	150.4688
Q9EQK5	Major vault protein OS=Mus musculus GN=Mvp PE=1 SV=4		24.15796	17	41	17	95.865	5.59	12000000	11000000	12000000	113.4151
Q3UFM5	Nucleolar MIF4G domain-containing protein 1 OS=Mus musculus GN=Nom1 PE=1 SV=2		2.576112	1	2	1	95.9	8.87	360000		230000	5.416507
Q8VDM6	Heterogeneous nuclear ribonucleoprotein U-like protein 1 OS=Mus musculus GN=Hnrnpul1 PE=1 SV=1		7.217695	5	14	5	95.942	6.58	3100000	5300000	5300000	42.22131
Q9Z1X4	Interleukin enhancer-binding factor 3 OS=Mus musculus GN=Ilf3 PE=1 SV=2		22.16036	14	38	14	95.961	8.76	8600000	8300000	8900000	110.9854
Q9WU78	Programmed cell death 6-interacting protein OS=Mus musculus GN=Pdcd6ip PE=1 SV=3		15.88032	10	20	10	95.964	6.52	2400000	2000000	3200000	49.97485
P58069	Ras GTPase-activating protein 2 OS=Mus musculus GN=Rasa2 PE=1 SV=2		2.243211	2	3	2	96.34	7.59		2400000	1100000	7.321538
Q61699	Heat shock protein 105 kDa OS=Mus musculus GN=Hsph1 PE=1 SV=2		25.75758	14	31	14	96.346	5.53	5100000	4000000	4000000	88.98017
Q9ESV0	ATP-dependent RNA helicase DDX24 OS=Mus musculus GN=Ddx24 PE=1 SV=2		7.234539	4	11	4	96.369	9.36	4000000	4000000	3600000	33.11039
Q7TSJ2	Microtubule-associated protein 6 OS=Mus musculus GN=Map6 PE=1 SV=2		19.31567	11	29	11	96.391	9.5	4300000	3100000	2400000	102.1025
Q9Z1G4	V-type proton ATPase 116 kDa subunit a isoform 1 OS=Mus musculus GN=Atp6v0a1 PE=1 SV=3		14.18355	10	29	10	96.404	6.76	16000000	6300000	6400000	89.55339
Q8BZ03	Serine/threonine-protein kinase D2 OS=Mus musculus GN=Prkd2 PE=1 SV=1		1.828571	1	1	1	96.481	6.89	480000			2.351743
Q80YR9	RNA-binding protein 12B-A OS=Mus musculus GN=Rbm12b1 PE=1 SV=2		1.674641	1	1	1	96.535	8.07		420000		2.549384
Q8CAS9	Poly [ADP-ribose] polymerase 9 OS=Mus musculus GN=Parp9 PE=1 SV=2		17.78291	13	28	13	96.597	7.09	4800000	5200000	6200000	78.26091
Q99LI5	Zinc finger protein 281 OS=Mus musculus GN=Znf281 PE=1 SV=1		1.791713	1	1	1	96.625	8.56				3.403276
Q8CI94	Glycogen phosphorylase, brain form OS=Mus musculus GN=Pygb PE=1 SV=3		19.57295	13	29	10	96.668	6.73	8600000	6400000	6000000	78.01214
P49717	DNA replication licensing factor MCM4 OS=Mus musculus GN=Mcm4 PE=1 SV=1		9.860789	8	19	8	96.676	7.2	5400000	4000000	4600000	58.27928
P70261	Paladin OS=Mus musculus GN=Pald1 PE=1 SV=1		1.280559	1	1	1	96.679	6.58		700000		2.258142

Q9WTK5	Nuclear factor NF-kappa-B p100 subunit OS=Mus musculus GN=Nfkb2 PE=1 SV=1		6.340378	4	8	4	96.772	6.37	2300000	2200000	1500000	28.53224
Q62165	Dystroglycan OS=Mus musculus GN=Dag1 PE=1 SV=4		3.023516	2	8	2	96.844	8.44	2800000	2600000	3000000	21.94866
Q3URD3	Sarcolemmal membrane-associated protein OS=Mus musculus GN=Slmap PE=1 SV=2		3.786982	2	3	2	96.873	5.33	510000	600000	690000	9.22897
Q60625	Intercellular adhesion molecule 5 OS=Mus musculus GN=Icam5 PE=1 SV=2		1.417666	1	1	1	96.885	6.32	1100000			2.769943
Q9DBS9	Oxysterol-binding protein-related protein 3 OS=Mus musculus GN=Osbpl3 PE=1 SV=2		1.637427	1	1	1	96.905	6.51	350000			3.291616
Q9ES28	Rho guanine nucleotide exchange factor 7 OS=Mus musculus GN=Arhgef7 PE=1 SV=2		6.728538	5	12	3	96.995	6.8	2800000	2100000	3900000	32.73768
P70168	Importin subunit beta-1 OS=Mus musculus GN=Kpnb1 PE=1 SV=2		13.69863	10	24	10	97.122	4.78	12000000	9600000	10000000	68.93052
Q3UMC0	Spermatogenesis-associated protein 5 OS=Mus musculus GN=Spata5 PE=1 SV=2		4.591265	3	5	2	97.195	8.24	1200000	380000		14.95769
Q9WUB3	Glycogen phosphorylase, muscle form OS=Mus musculus GN=Pygm PE=1 SV=3		6.769596	5	9	2	97.225	7.11	1500000		700000	20.89617
Q6PIX5	Inactive rhomboid protein 1 OS=Mus musculus GN=Rhbdf1 PE=1 SV=2		1.635514	1	2	1	97.229	8.59				4.950971
Q8CJG0	Protein argonaute-2 OS=Mus musculus GN=Ago2 PE=1 SV=3		2.790698	2	4	2	97.242	9.19		1800000	1400000	9.2297
B1AZA5	Transmembrane protein 245 OS=Mus musculus GN=Tmem245 PE=1 SV=1		0.913242	1	2	1	97.295	8.75				4.349407
Q9QZE5	Coatomer subunit gamma-1 OS=Mus musculus GN=Copg1 PE=1 SV=1		15.44622	10	16	9	97.45	5.35	4900000	6300000	5400000	49.65672
Q5SSL4	Active breakpoint cluster region-related protein OS=Mus musculus GN=Abr PE=1 SV=1		4.307334	2	3	2	97.605	6.58	2000000	2300000		8.927358
Q9QXK3	Coatomer subunit gamma-2 OS=Mus musculus GN=Copg2 PE=1 SV=1		6.08496	4	10	3	97.617	5.8	1600000	2200000	1800000	29.39487
Q8K4L0	ATP-dependent RNA helicase DDX54 OS=Mus musculus GN=Ddx54 PE=1 SV=1		1.258581	1	1	1	97.687	10.01			1300000	2.476184
P39053	Dynamin-1 OS=Mus musculus GN=Dnm1 PE=1 SV=2		26.18224	20	44	14	97.741	7.74	23000000	12000000	11000000	137.6536
Q8VHH5	Arf-GAP with GTPase, ANK repeat and PH domain-containing protein 3 OS=Mus musculus GN=Agap3 PE=1 SV=1		1.758242	1	1	1	97.905	7.75	2300000			3.86217
Q8BVG4	Dipeptidyl peptidase 9 OS=Mus musculus GN=Dpp9 PE=1 SV=2		1.276102	1	1	1	97.939	6.65	540000			2.300429
Q3V1V3	ESF1 homolog OS=Mus musculus GN=Esf1 PE=1 SV=1		4.970414	3	4	3	97.988	5.05	660000		1600000	10.95707
Q8BGR2	Volume-regulated anion channel subunit LRRC8D OS=Mus musculus GN=Lrrc8d PE=1 SV=1		2.328289	1	1	1	98.05	7.44		560000		2.220653
P28271	Cytoplasmic aconitate hydratase OS=Mus musculus GN=Aco1 PE=1 SV=3		2.474691	1	1	1	98.063	7.5		670000		3.863647
P27870	Proto-oncogene vav OS=Mus musculus GN=Vav1 PE=1 SV=1		2.366864	2	3	2	98.074	6.51	3400000		1200000	9.053568
P15920	V-type proton ATPase 116 kDa subunit a isoform 2 OS=Mus musculus GN=Atp6v0a2 PE=1 SV=2		2.102804	1	2	1	98.081	6.64	880000		1200000	5.601257
P39054	Dynamin-2 OS=Mus musculus GN=Dnm2 PE=1 SV=2		18.62069	14	36	8	98.084	7.43	11000000	7400000	6200000	112.1032
Q00993	Tyrosine-protein kinase receptor UFO OS=Mus musculus GN=Axl PE=1 SV=2		3.828829	3	8	3	98.128	5.74	2900000	2900000	2500000	22.46316
Q60953	Protein PML OS=Mus musculus GN=Pml PE=1 SV=3		12.65537	7	15	7	98.18	5.63	4100000	3400000	4200000	48.07614
Q9Z2H5	Band 4.1-like protein 1 OS=Mus musculus GN=Epb41l1 PE=1 SV=2		5.23322	3	5	3	98.254	5.62	1400000	1000000		15.66906
P42567	Epidermal growth factor receptor substrate 15 OS=Mus musculus GN=Eps15 PE=1 SV=1		11.14827	7	10	7	98.41	4.6	1700000	1400000	1500000	27.96553
O54931	A-kinase anchor protein 2 OS=Mus musculus GN=Akap2 PE=1 SV=3		1.119821	1	3	1	98.519	5.21	1300000	1100000	760000	7.031648
P23819	Glutamate receptor 2 OS=Mus musculus GN=Gria2 PE=1 SV=3		2.718007	2	5	2	98.599	7.39	2600000	860000	890000	14.77883
Q8R0Y6	Cytosolic 10-formyltetrahydrofolate dehydrogenase OS=Mus musculus GN=Aldh1l1 PE=1 SV=1		23.50333	16	41	16	98.647	5.91	9000000	6500000	5700000	127.4996
Q8R3N1	Nucleolar protein 14 OS=Mus musculus GN=Nop14 PE=1 SV=2		5.697674	4	10	4	98.708	7.59	1900000	1700000	2300000	31.41555
Q9R1E6	Ectonucleotide pyrophosphatase/phosphodiesterase family member 2 OS=Mus musculus GN=Enpp2 PE=1 SV=3		1.392111	1	1	1	98.821	7.27			480000	2.025453
Q80YR4	Zinc finger protein 598 OS=Mus musculus GN=Znf598 PE=1 SV=1		3.854626	2	3	2	99.13	8.34	450000		470000	7.980035
Q60902	Epidermal growth factor receptor substrate 15-like 1 OS=Mus musculus GN=Eps15l1 PE=1 SV=3		14.22271	9	18	9	99.248	5.02	3200000	2700000	1700000	57.42564
Q61704	Inter-alpha-trypsin inhibitor heavy chain H3 OS=Mus musculus GN=Itih3 PE=1 SV=3		11.81102	7	20	7	99.296	6.05	14000000	14000000	13000000	61.94093
O35551	Rab GTPase-binding effector protein 1 OS=Mus musculus GN=Rabep1 PE=1 SV=2		2.784223	2	4	2	99.462	5.01	1300000	750000	910000	10.32512
Q8CC35	Synaptopodin OS=Mus musculus GN=Synpo PE=1 SV=2		1.829925	1	3	1	99.49	9.42	2200000	1900000	1400000	9.826266
B2RY56	RNA-binding protein 25 OS=Mus musculus GN=Rbm25 PE=1 SV=2		18.73508	12	26	12	99.491	6.32	6200000	6400000	9700000	91.60327
Q6PCM2	Integrator complex subunit 6 OS=Mus musculus GN=Ints6 PE=1 SV=1		1.698754	1	1	1	99.598	8.78			480000	2.427056
Q9R1V6	Disintegrin and metalloproteinase domain-containing protein 22 OS=Mus musculus GN=Adam22 PE=1 SV=2		3.539823	2	3	2	99.651	7.83	470000	540000		8.587853
P15116	Cadherin-2 OS=Mus musculus GN=Cdh2 PE=1 SV=2		3.752759	3	6	3	99.734	4.78	2700000	1800000	1200000	23.97148
O08759	Ubiquitin-protein ligase E3A OS=Mus musculus GN=Ube3a PE=1 SV=2		3.103448	2	6	2	99.756	5.08	1100000	980000	950000	15.09674
P97329	Kinesin-like protein KIF20A OS=Mus musculus GN=Kif20a PE=2 SV=1		4.735062	3	4	3	99.814	6.99	420000	1000000	650000	10.56443
Q60992	Guanine nucleotide exchange factor VAV2 OS=Mus musculus GN=Vav2 PE=1 SV=1		1.958525	1	1	1	99.851	6.84	350000			2.480351
Q9DCD2	Pre-mRNA-splicing factor SYF1 OS=Mus musculus GN=Xab2 PE=1 SV=1		3.74269	3	7	3	99.924	6.23	840000	1500000	1000000	18.56709
Q60591	Nuclear factor of activated T-cells, cytoplasmic 2 OS=Mus musculus GN=Nfatc2 PE=1 SV=3		1.510248	1	1	1	99.957	6.76			440000	2.166946
Q3TRM8	Hexokinase-3 OS=Mus musculus GN=Hk3 PE=1 SV=2		3.470716	2	4	2	100.037	5.94	1100000	780000		10.19273
P26231	Catenin alpha-1 OS=Mus musculus GN=Ctnna1 PE=1 SV=1		15.23179	12	25	8	100.044	6.23	4600000	4400000	4300000	77.33126
Q811D0	Disks large homolog 1 OS=Mus musculus GN=Dlg1 PE=1 SV=1		1.878453	1	3	1	100.058	5.8	5100000	2900000	2900000	9.409895
Q3UVK0	Endoplasmic reticulum metallopeptidase 1 OS=Mus musculus GN=Ermp1 PE=1 SV=2		1.113586	1	2	1	100.084	7.49		820000	1100000	5.701212
Q8VDM4	26S proteasome non-ATPase regulatory subunit 2 OS=Mus musculus GN=Psmd2 PE=1 SV=1		11.67401	9	24	9	100.139	5.17	7500000	7100000	5500000	73.9603

Q9WU40	Inner nuclear membrane protein Man1 OS=Mus musculus GN=Lemd3 PE=1 SV=2		8.686211	5	10	5	100.244	7.55	1000000	810000	810000	36.0643
Q91XY4	Protocadherin gamma-A4 OS=Mus musculus GN=Pcdhga4 PE=2 SV=1		1.935484	1	1	1	100.297	4.87				2.578254
Q99MR6	Serrate RNA effector molecule homolog OS=Mus musculus GN=Srrt PE=1 SV=1		9.828571	8	18	8	100.39	5.97	4700000	5900000	7100000	51.21757
P56695	Wolframin OS=Mus musculus GN=Wfs1 PE=1 SV=1		1.235955	1	3	1	100.514	7.97	1200000	1100000	730000	7.39094
Q8K596	Sodium/calcium exchanger 2 OS=Mus musculus GN=Slc8a2 PE=1 SV=1		5.537459	4	7	4	100.647	5.12	3700000	230000	1900000	19.83476
Q80Y44	Probable ATP-dependent RNA helicase DDX10 OS=Mus musculus GN=Ddx10 PE=1 SV=2		8	5	10	5	100.676	7.75	1600000	2500000	2000000	34.50772
Q9D2V7	Coronin-7 OS=Mus musculus GN=Coro7 PE=1 SV=2		6.724512	4	8	4	100.749	5.77	4800000	2300000	3000000	19.54633
Q8BJL0	SWI/SNF-related matrix-associated actin-dependent regulator of chromatin subfamily A-like protein 1 OS=Mus musculus GN=Smar		2.307692	1	2	1	100.777	8.9	240000			5.64379
P56960	Exosome component 10 OS=Mus musculus GN=Exosc10 PE=1 SV=2		1.352875	1	2	1	100.879	8.29	620000	620000		4.490657
Q80UM3	N-alpha-acetyltransferase 15, NatA auxiliary subunit OS=Mus musculus GN=Naa15 PE=1 SV=1		9.017341	8	15	8	100.897	7.62	4500000	4300000	3900000	33.7762
Q61702	Inter-alpha-trypsin inhibitor heavy chain H1 OS=Mus musculus GN=Itih1 PE=1 SV=2		15.4355	10	26	9	101.004	6.96	11000000	9800000	9200000	77.08621
Q99JP0	Mitogen-activated protein kinase kinase kinase kinase 3 OS=Mus musculus GN=Map4k3 PE=1 SV=4		1.901566	1	1	1	101.054	7.64	300000			3.119617
Q80WT5	Aftiphilin OS=Mus musculus GN=Aftph PE=1 SV=2		1.718582	1	3	1	101.068	4.5		480000	280000	8.131066
Q922D8	C-1-tetrahydrofolate synthase, cytoplasmic OS=Mus musculus GN=Mthfd1 PE=1 SV=4		11.01604	9	22	9	101.136	7.14	6100000	5800000	5000000	63.12632
Q0VCM5	TREMBL:Q0VCM5 (Bos taurus) Similar to Inter-alpha-trypsin inhibitor heavy chain H1	x	1.87638	2	3	1	101.173	7.4				6.318283
B9EJ86	Oxysterol-binding protein-related protein 8 OS=Mus musculus GN=Osbpl8 PE=1 SV=1		2.137233	1	1	1	101.205	6.96	2700000			2.889274
Q68FL6	Methionine--tRNA ligase, cytoplasmic OS=Mus musculus GN=Mars PE=1 SV=1		4.988914	4	7	4	101.366	7.14	1200000	4600000	780000	16.96848
Q6PF93	Phosphatidylinositol 3-kinase catalytic subunit type 3 OS=Mus musculus GN=Pik3c3 PE=1 SV=1		1.803833	1	3	1	101.423	6.73	1200000	910000	780000	9.626356
Q6NVE8	WD repeat-containing protein 44 OS=Mus musculus GN=Wdr44 PE=1 SV=1		1.748634	1	3	1	101.493	5.24		500000	1000000	9.714502
P23818	Glutamate receptor 1 OS=Mus musculus GN=Gria1 PE=1 SV=1		1.653804	1	1	1	101.504	7.69	430000			2.792026
Q8K009	Mitochondrial 10-formyltetrahydrofolate dehydrogenase OS=Mus musculus GN=Aldh1l2 PE=1 SV=2		17.98483	13	24	13	101.526	6.29	4600000	4200000	4000000	68.1121
O88508	DNA (cytosine-5)-methyltransferase 3A OS=Mus musculus GN=Dnmt3a PE=1 SV=2		1.982379	1	3	1	101.607	6.65	620000	1000000	850000	10.64777
Q8R5L3	Vam6/Vps39-like protein OS=Mus musculus GN=Vps39 PE=1 SV=1		3.273138	2	4	2	101.628	6.99	1600000	1200000	1000000	12.53741
Q8R3S6	Exocyst complex component 1 OS=Mus musculus GN=Exoc1 PE=1 SV=4		3.467562	3	5	3	101.825	6.52	880000	1200000	820000	11.5992
Q810A7	ATP-dependent RNA helicase DDX42 OS=Mus musculus GN=Ddx42 PE=1 SV=3		9.472551	7	21	7	101.902	6.98	4100000	3800000	3900000	57.51208
Q9D666	SUN domain-containing protein 1 OS=Mus musculus GN=Sun1 PE=1 SV=2		1.533406	1	2	1	101.912	6.81	280000			3.059823
P97310	DNA replication licensing factor MCM2 OS=Mus musculus GN=Mcm2 PE=1 SV=3		12.27876	9	20	9	102.013	5.72	8100000	7900000	4300000	59.18849
Q78PY7	Staphylococcal nuclease domain-containing protein 1 OS=Mus musculus GN=Snd1 PE=1 SV=1		25.05495	18	54	18	102.025	7.43	25000000	25000000	25000000	167.7046
Q62448	Eukaryotic translation initiation factor 4 gamma 2 OS=Mus musculus GN=Eif4g2 PE=1 SV=2		10.2649	7	14	7	102.041	7.14	5400000	7500000	4000000	40.55884
P37913	DNA ligase 1 OS=Mus musculus GN=Lig1 PE=1 SV=2		6.659389	4	5	4	102.226	6.81	410000	1800000	950000	10.24458
Q6KAQ7	ZZ-type zinc finger-containing protein 3 OS=Mus musculus GN=Zzz3 PE=1 SV=2		1.868132	1	1	1	102.243	5.86		3200000		2.588492
Q8BFY9	Transportin-1 OS=Mus musculus GN=Tnpo1 PE=1 SV=2		13.14031	7	13	7	102.291	4.98	4200000	4900000	4000000	37.05684
O55029	Coatomer subunit beta' OS=Mus musculus GN=Copb2 PE=1 SV=2		18.34254	13	25	13	102.384	5.3	7200000	6400000	8000000	71.98208
O08528	Hexokinase-2 OS=Mus musculus GN=Hk2 PE=1 SV=1		12.759	9	17	8	102.469	6.11	3300000	3000000	2900000	51.22593
Q8R3B7	Bromodomain-containing protein 8 OS=Mus musculus GN=Brd8 PE=1 SV=2		1.68244	1	3	1	102.546	4.72	590000	520000	630000	9.184064
Q3UW53	Protein Niban OS=Mus musculus GN=Fam129a PE=1 SV=2		2.699784	2	4	2	102.585	4.78	3300000	2400000	2100000	13.70858
P46935	E3 ubiquitin-protein ligase NEDD4 OS=Mus musculus GN=Nedd4 PE=1 SV=3		14.99436	10	28	10	102.642	5.26	4300000	3300000	3400000	87.33286
Q8VBT6	Apolipoprotein B receptor OS=Mus musculus GN=Apobr PE=1 SV=1		2.972399	2	2	2	102.643	4.36	890000			5.283274
Q61210	Rho guanine nucleotide exchange factor 1 OS=Mus musculus GN=Arhgef1 PE=1 SV=2		14.56522	8	12	8	102.741	5.6	1300000	2500000	2000000	37.56872
Q8BU03	Periodic tryptophan protein 2 homolog OS=Mus musculus GN=Pwp2 PE=1 SV=1		2.829162	2	7	2	102.845	6.23	1100000	760000	1100000	17.85979
Q8C052	Microtubule-associated protein 1S OS=Mus musculus GN=Map1s PE=1 SV=2		1.027749	1	1	1	102.875	7.02		490000		2.027651
P97333	Neuropilin-1 OS=Mus musculus GN=Nrp1 PE=1 SV=2		5.633803	3	7	3	102.935	5.9	1100000	1100000	1100000	18.65611
Q8VDP4	Cell cycle and apoptosis regulator protein 2 OS=Mus musculus GN=Ccar2 PE=1 SV=2		8.56833	4	8	4	102.938	5.25	3100000	2300000	3100000	26.30083
Q7TPR4	Alpha-actinin-1 OS=Mus musculus GN=Actn1 PE=1 SV=1		37.89238	28	101	15	103.004	5.38	15000000	12000000	11000000	320.1853
Q9EQQ9	Protein O-GlcNAcase OS=Mus musculus GN=Mgea5 PE=1 SV=2		3.49345	3	6	3	103.096	4.92	2000000	3200000	1900000	14.32758
Q8BG79	CWF19-like protein 2 OS=Mus musculus GN=Cwf19l2 PE=1 SV=1		1.578354	1	1	1	103.108	8.5		71000		1.896783
P06802	Ectonucleotide pyrophosphatase/phosphodiesterase family member 1 OS=Mus musculus GN=Enpp1 PE=1 SV=4		3.532009	3	5	3	103.109	6.6	1800000	1500000		12.67027
Q921G8	Gamma-tubulin complex component 2 OS=Mus musculus GN=Tubgcp2 PE=1 SV=2		1.878453	1	3	1	103.157	6.77	930000	590000	570000	9.455177
Q922S4	cGMP-dependent 3',5'-cyclic phosphodiesterase OS=Mus musculus GN=Pde2a PE=1 SV=3		1.637555	1	2	1	103.183	5.38	1600000	680000		5.765125
Q8CFE4	SCY1-like protein 2 OS=Mus musculus GN=Scyl2 PE=1 SV=1		3.11828	2	2	2	103.251	8.02	670000		920000	6.500274
Q11011	Puromycin-sensitive aminopeptidase OS=Mus musculus GN=Npepps PE=1 SV=2		2.5	2	5	2	103.26	5.9	2900000	2300000	3100000	15.0087
Q9WV92	Band 4.1-like protein 3 OS=Mus musculus GN=Epb41l3 PE=1 SV=1		22.60495	14	31	12	103.274	5.31	6600000	3000000	3700000	99.48203
Q3UHJ0	AP2-associated protein kinase 1 OS=Mus musculus GN=Aak1 PE=1 SV=2		15.22419	9	17	9	103.282	6.7	3800000	3000000	2700000	53.03912

Q6NZL0	Protein SOGA3 OS=Mus musculus GN=Soga3 PE=1 SV=2		4.973545	4	5	4	103.416	6.14	1600000	320000	1300000	13.79862
Q99KG3	RNA-binding protein 10 OS=Mus musculus GN=Rbm10 PE=1 SV=1		3.978495	3	7	3	103.43	5.92	2400000	2000000	1700000	19.98202
Q3U0S6	Ras-interacting protein 1 OS=Mus musculus GN=Rasip1 PE=1 SV=3		3.01769	2	3	2	103.494	7.83		340000	480000	8.10523
Q99MB1	Toll-like receptor 3 OS=Mus musculus GN=Tlr3 PE=1 SV=2		1.325967	1	2	1	103.606	7.93	1300000		1200000	5.535624
Q80T85	DDB1- and CUL4-associated factor 5 OS=Mus musculus GN=Dcaf5 PE=1 SV=2		1.585624	1	1	1	103.612	5.8	150000			2.420544
Q9QZD4	DNA repair endonuclease XPF OS=Mus musculus GN=Ercc4 PE=1 SV=3		1.308615	1	1	1	103.625	7.71	590000			2.449982
Q8K1A6	Coiled-coil and C2 domain-containing protein 1A OS=Mus musculus GN=Cc2d1a PE=1 SV=2		0.954401	1	2	1	103.634	7.84	700000		570000	4.321098
Q9ES97	Reticulon-3 OS=Mus musculus GN=Rtn3 PE=1 SV=2		4.253112	3	8	2	103.814	4.92	11000000	8000000	5900000	24.15471
O35643	AP-1 complex subunit beta-1 OS=Mus musculus GN=Ap1b1 PE=1 SV=2		11.45281	11	27	4	103.869	5.17	2000000	3400000	5700000	70.53575
Q9D4H1	Exocyst complex component 2 OS=Mus musculus GN=Exoc2 PE=1 SV=1		2.922078	2	4	2	103.894	7.18	660000	1300000	620000	11.23807
P17427	AP-2 complex subunit alpha-2 OS=Mus musculus GN=Ap2a2 PE=1 SV=2		11.9403	10	26	8	103.951	6.93	6600000	6700000	6200000	69.20802
Q80UP3	Diacylglycerol kinase zeta OS=Mus musculus GN=Dgkz PE=1 SV=2		3.444564	2	4	2	103.965	8.09	740000	1200000		9.934878
Q05895	Thrombospondin-3 OS=Mus musculus GN=Thbs3 PE=1 SV=2		2.719665	1	1	1	104.053	4.64		870000		2.491683
P43247	DNA mismatch repair protein Msh2 OS=Mus musculus GN=Msh2 PE=1 SV=1		3.850267	3	5	3	104.085	5.96	1700000	1400000	700000	13.11218
Q8BRH0	Transmembrane and TPR repeat-containing protein 3 OS=Mus musculus GN=Tmtc3 PE=1 SV=2		2.826087	2	3	2	104.131	8.53		1200000	1900000	6.268331
P70268	Serine/threonine-protein kinase N1 OS=Mus musculus GN=Pkn1 PE=1 SV=3		1.374207	1	1	1	104.346	6.27				1.926614
Q7TSG2	RNA polymerase II subunit A C-terminal domain phosphatase OS=Mus musculus GN=Ctdp1 PE=1 SV=1		3.229167	2	3	2	104.49	5.39	270000	240000		8.771295
Q3UUQ7	GPI inositol-deacylase OS=Mus musculus GN=Pgap1 PE=1 SV=3		1.193059	1	1	1	104.511	8.91		550000		2.153604
Q9DBG3	AP-2 complex subunit beta OS=Mus musculus GN=Ap2b1 PE=1 SV=1		12.70011	12	34	5	104.516	5.38	10000000	7700000	8600000	90.28792
A6X935	Inter alpha-trypsin inhibitor, heavy chain 4 OS=Mus musculus GN=Itih4 PE=1 SV=2		3.078556	2	4	2	104.594	6.4	1300000	1100000	830000	11.55678
Q8C5N3	Pre-mRNA-splicing factor CWC22 homolog OS=Mus musculus GN=Cwc22 PE=1 SV=1		1.211454	1	1	1	104.709	8.09			1900000	2.434024
Q8K212	Phosphofurin acidic cluster sorting protein 1 OS=Mus musculus GN=Pacs1 PE=1 SV=2		2.601457	2	5	2	104.764	7.74	2100000	970000	1400000	12.83167
P58501	PAX3- and PAX7-binding protein 1 OS=Mus musculus GN=Paxbp1 PE=1 SV=3		3.155604	2	3	2	104.771	5.67		1800000	1200000	8.924626
P30999	Catenin delta-1 OS=Mus musculus GN=Ctnnd1 PE=1 SV=2		10.76759	8	20	8	104.86	6.87	4200000	5700000	4700000	57.82165
P57780	Alpha-actinin-4 OS=Mus musculus GN=Actn4 PE=1 SV=1		51.53509	40	147	27	104.911	5.41	29000000	25000000	26000000	494.4208
Q9R1S3	GPI ethanolamine phosphate transferase 1 OS=Mus musculus GN=Pign PE=2 SV=2		1.503759	1	2	1	104.977	7.58	670000	740000		4.94649
D3YXK2	Scaffold attachment factor B1 OS=Mus musculus GN=Safb PE=1 SV=2		22.51868	16	49	10	105.04	5.35	9400000	9400000	7400000	157.2987
O70589	Peripheral plasma membrane protein CASK OS=Mus musculus GN=Cask PE=1 SV=2		1.727862	1	2	1	105.042	6.43	730000	380000		5.253078
Q3URQ0	Testis-expressed sequence 10 protein OS=Mus musculus GN=Tex10 PE=1 SV=1		1.077586	1	1	1	105.143	9.16		1200000		2.432589
Q61301	Catenin alpha-2 OS=Mus musculus GN=Ctnna2 PE=1 SV=3		8.814271	7	14	3	105.22	5.71	760000			42.26773
Q8BYC6	Serine/threonine-protein kinase TAO3 OS=Mus musculus GN=Taok3 PE=1 SV=2		8.017817	6	8	5	105.27	7.36	1600000	1200000	1200000	22.20392
Q8CHP6	Polyhomeotic-like protein 3 OS=Mus musculus GN=Phc3 PE=1 SV=2		1.325178	1	3	1	105.287	6.6		1100000	1500000	8.58216
Q9WVL2	Signal transducer and activator of transcription 2 OS=Mus musculus GN=Stat2 PE=1 SV=1		7.150596	6	15	6	105.351	5.27	6100000	6200000	5100000	40.63093
Q8R1B4	Eukaryotic translation initiation factor 3 subunit C OS=Mus musculus GN=Eif3c PE=1 SV=1		18.22173	15	49	15	105.465	5.78	16000000	17000000	15000000	155.6834
Q0P678	Zinc finger CCCH domain-containing protein 18 OS=Mus musculus GN=Zc3h18 PE=1 SV=1		14.9789	7	14	7	105.631	7.8	4400000	4400000	5000000	56.27529
Q3V3R1	Monofunctional C1-tetrahydrofolate synthase, mitochondrial OS=Mus musculus GN=Mthfd1l PE=1 SV=2		13.81781	12	23	12	105.662	7.02	5400000	3300000	4000000	62.6163
Q3TXS7	26S proteasome non-ATPase regulatory subunit 1 OS=Mus musculus GN=Psmd1 PE=1 SV=1		20.25184	13	33	13	105.663	5.39	8300000	6400000	6500000	109.1407
Q8BHB4	WD repeat-containing protein 3 OS=Mus musculus GN=Wdr3 PE=1 SV=1		7.430998	4	9	4	105.708	6.64	2200000	1900000	1400000	28.49126
Q8CGK3	Lon protease homolog, mitochondrial OS=Mus musculus GN=Lonp1 PE=1 SV=2		10.85353	8	13	8	105.776	6.57	3600000	4700000	4000000	40.23609
Q61703	Inter-alpha-trypsin inhibitor heavy chain H2 OS=Mus musculus GN=Itih2 PE=1 SV=1		11.20507	7	15	7	105.861	7.27	6400000	4800000	5300000	45.31596
Q6Q899	Probable ATP-dependent RNA helicase DDX58 OS=Mus musculus GN=Ddx58 PE=1 SV=2		6.37149	6	12	6	105.908	6.65	4500000	3600000	2900000	28.91644
Q8K019	Bcl-2-associated transcription factor 1 OS=Mus musculus GN=Bclaf1 PE=1 SV=2		16.64853	11	36	10	105.939	9.99	14000000	16000000	16000000	110.2447
Q8R3C6	Probable RNA-binding protein 19 OS=Mus musculus GN=Rbm19 PE=1 SV=1		4.516807	3	3	3	106.018	6.57	170000		510000	7.105948
Q8CGZ0	Calcium homeostasis endoplasmic reticulum protein OS=Mus musculus GN=Cherp PE=1 SV=1		3.418803	3	7	3	106.102	9.14	3300000	3300000	3300000	18.48351
Q6P1H6	Ankyrin repeat and LEM domain-containing protein 2 OS=Mus musculus GN=Ankle2 PE=1 SV=2		4.875519	3	5	3	106.132	7.37	850000	970000		15.77775
Q6P9J9	Anoctamin-6 OS=Mus musculus GN=Ano6 PE=1 SV=1		2.854007	2	6	2	106.186	6.76	2400000	2500000	2600000	17.97032
Q14B71	Cell division cycle-associated protein 2 OS=Mus musculus GN=Cdca2 PE=1 SV=2		3.156823	2	2	2	106.3	8.65		310000		5.777756
F7BJB9	MORC family CW-type zinc finger protein 3 OS=Mus musculus GN=Morc3 PE=1 SV=1		1.061571	1	1	1	106.455	5.68	810000			2.102331
Q80TQ2	Ubiquitin carboxyl-terminal hydrolase CYLD OS=Mus musculus GN=Cyld PE=1 SV=2		2.731092	2	2	2	106.517	5.63		1100000		4.489256
Q9EQH2	Endoplasmic reticulum aminopeptidase 1 OS=Mus musculus GN=Erap1 PE=1 SV=2		2.150538	2	3	2	106.531	6.2	1000000	1900000	1900000	7.432761
Q3UPF5	Zinc finger CCCH-type antiviral protein 1 OS=Mus musculus GN=Zc3hav1 PE=1 SV=1		12.15645	7	15	7	106.619	8.27	3600000	3700000	4100000	50.46609
Q8BH74	Nuclear pore complex protein Nup107 OS=Mus musculus GN=Nup107 PE=1 SV=1		10.90713	7	17	7	106.65	5.43	1800000	1800000	2800000	52.97211
Q91YR7	Pre-mRNA-processing factor 6 OS=Mus musculus GN=Prpf6 PE=1 SV=1		13.60255	11	27	11	106.655	8.09	3600000	3900000	4000000	85.63538

O08746	Matrilin-2 OS=Mus musculus GN=Matn2 PE=2 SV=2		21.0251	16	38	16	106.678	6.58	8300000	10000000	8900000	124.3715
Q8BJD1	Inter-alpha-trypsin inhibitor heavy chain H5 OS=Mus musculus GN=Itih5 PE=1 SV=1		1.365546	1	1	1	106.683	8.16				2.173194
Q7SIG6	Arf-GAP with SH3 domain, ANK repeat and PH domain-containing protein 2 OS=Mus musculus GN=Asap2 PE=1 SV=3		4.175365	3	5	3	106.738	6.65	730000	730000	750000	14.66166
Q99N50	Synaptotagmin-like protein 2 OS=Mus musculus GN=Sytl2 PE=1 SV=2		1.789474	1	1	1	106.74	6.57		350000		3.175174
Q7TQK1	Integrator complex subunit 7 OS=Mus musculus GN=Ints7 PE=1 SV=1		1.345756	1	2	1	106.793	8.22	820000		970000	4.552812
Q52KI8	Serine/arginine repetitive matrix protein 1 OS=Mus musculus GN=Srrm1 PE=1 SV=2		4.968288	4	11	4	106.798	11.87	9200000	11000000	9900000	32.55108
Q8BGQ7	Alanine--tRNA ligase, cytoplasmic OS=Mus musculus GN=Aars PE=1 SV=1		6.714876	5	8	5	106.841	5.67	1400000	1900000	1800000	17.67218
Q8BHN3	Neutral alpha-glucosidase AB OS=Mus musculus GN=Ganab PE=1 SV=1		10.80508	8	20	8	106.844	6.06	8800000	11000000	8600000	59.84887
Q9Z1Z0	General vesicular transport factor p115 OS=Mus musculus GN=Uso1 PE=1 SV=2		22.73201	15	38	15	106.917	4.93	6400000	6800000	5800000	127.6698
Q9JIF7	Coatomer subunit beta OS=Mus musculus GN=Copb1 PE=1 SV=1		13.01154	11	32	11	106.998	6	15000000	14000000	15000000	98.78698
Q8K1R7	Serine/threonine-protein kinase Nek9 OS=Mus musculus GN=Nek9 PE=1 SV=2		4.471545	2	3	2	107.075	5.63	1200000	940000		9.60533
Q91Y44	Bromodomain testis-specific protein OS=Mus musculus GN=Brdt PE=1 SV=3		1.569038	1	1	1	107.188	8.97				2.063365
B2RXR6	Serine/threonine-protein phosphatase 6 regulatory ankyrin repeat subunit B OS=Mus musculus GN=Ankrd44 PE=1 SV=1		3.222558	2	4	2	107.317	6.42	1800000	910000	1700000	14.89276
Q64737	Trifunctional purine biosynthetic protein adenosine-3 OS=Mus musculus GN=Gart PE=1 SV=3		3.366337	3	3	3	107.436	6.68	1800000		1400000	6.989492
Q6PGG2	GEM-interacting protein OS=Mus musculus GN=Gmip PE=1 SV=1		3.707518	2	3	2	107.478	5.77	520000		260000	8.720665
Q99K41	EMILIN-1 OS=Mus musculus GN=Emilin1 PE=1 SV=1		23.69715	18	52	18	107.518	5.3	21000000	17000000	17000000	160.8721
P17426	AP-2 complex subunit alpha-1 OS=Mus musculus GN=Ap2a1 PE=1 SV=1		18.21904	14	30	12	107.596	7.03	6300000	4600000	3100000	75.73462
Q91W86	Vacuolar protein sorting-associated protein 11 homolog OS=Mus musculus GN=Vps11 PE=1 SV=3		1.594049	1	3	1	107.65	7.01	2000000	1400000	1300000	9.724142
Q8R0S2	IQ motif and SEC7 domain-containing protein 1 OS=Mus musculus GN=Iqsec1 PE=1 SV=2		8.42872	7	7	5	107.948	7.14	1100000	920000	770000	17.03548
Q569Z6	Thyroid hormone receptor-associated protein 3 OS=Mus musculus GN=Thrap3 PE=1 SV=1		18.08623	14	42	13	108.114	10.17	8600000	14000000	9800000	124.4565
Q8CIM8	Integrator complex subunit 4 OS=Mus musculus GN=Ints4 PE=1 SV=1		2.489627	2	5	2	108.124	6.42	890000	740000	750000	13.63656
P17710	Hexokinase-1 OS=Mus musculus GN=Hk1 PE=1 SV=3		26.07803	23	60	22	108.234	6.8	30000000	21000000	19000000	173.8267
P35123	Ubiquitin carboxyl-terminal hydrolase 4 OS=Mus musculus GN=Usp4 PE=1 SV=3		9.87526	6	10	6	108.274	5.64	860000	1000000	640000	30.96517
Q9R1C7	Pre-mRNA-processing factor 40 homolog A OS=Mus musculus GN=Prpf40a PE=1 SV=1		7.974816	7	13	7	108.412	7.69	6100000	7100000	3900000	34.54805
Q04857	Collagen alpha-1(VI) chain OS=Mus musculus GN=Col6a1 PE=1 SV=1		18.43902	14	35	14	108.422	5.36	17000000	14000000	16000000	118.7392
Q8C8U0	Liprin-beta-1 OS=Mus musculus GN=Ppfibp1 PE=1 SV=3		11.24871	8	18	8	108.472	5.49	2400000	3100000	2000000	51.46279
Q9DBR1	5'-3' exoribonuclease 2 OS=Mus musculus GN=Xrn2 PE=1 SV=1		7.255521	5	15	5	108.618	7.59	3500000	3200000	3300000	42.59222
Q9CSH3	Exosome complex exonuclease RRP44 OS=Mus musculus GN=Dis3 PE=1 SV=4		6.471816	4	5	4	108.769	7.53	540000	650000	700000	14.74165
Q9EP71	Ankycorbin OS=Mus musculus GN=Rai14 PE=1 SV=1		7.048008	5	8	5	108.785	6.27	1200000	710000	1100000	21.01977
Q8BG95	Protein phosphatase 1 regulatory subunit 12B OS=Mus musculus GN=Ppp1r12b PE=1 SV=2		3.278689	2	5	1	108.984	5.74	580000	230000		17.19184
Q8BWQ6	UPF0505 protein C16orf62 homolog OS=Mus musculus PE=1 SV=2		1.142264	1	3	1	109.007	7.31	920000	790000		7.221817
P28738	Kinesin heavy chain isoform 5C OS=Mus musculus GN=Kif5c PE=1 SV=3		12.02929	9	19	4	109.207	6.19	1400000	630000	1100000	62.91438
O08810	116 kDa U5 small nuclear ribonucleoprotein component OS=Mus musculus GN=Eftud2 PE=1 SV=1		22.24511	16	41	15	109.291	5	8800000	7900000	7800000	106.1
Q61768	Kinesin-1 heavy chain OS=Mus musculus GN=Kif5b PE=1 SV=3		28.14123	21	54	16	109.484	6.44	6100000	6500000	4500000	161.6674
Q9JLI8	Squamous cell carcinoma antigen recognized by T-cells 3 OS=Mus musculus GN=Sart3 PE=1 SV=1		7.796258	5	13	5	109.55	5.24	3300000	3400000	1800000	43.26588
P97449	Aminopeptidase N OS=Mus musculus GN=Anpep PE=1 SV=4		13.25052	10	21	10	109.582	5.9	13000000	4600000	6800000	64.167
Q8VE18	Protein SMG8 OS=Mus musculus GN=Smg8 PE=1 SV=1		2.21998	1	1	1	109.597	7.77				3.484906
O70318	Band 4.1-like protein 2 OS=Mus musculus GN=Epb41l2 PE=1 SV=2		33.19838	26	71	24	109.873	5.43	13000000	11000000	8900000	227.9462
Q3UMY5	Echinoderm microtubule-associated protein-like 4 OS=Mus musculus GN=Eml4 PE=1 SV=1		5.668016	4	10	4	109.958	6.54	2300000	2300000	2000000	23.89396
Q9EQY0	Serine/threonine-protein kinase/endoribonuclease IRE1 OS=Mus musculus GN=Ern1 PE=1 SV=1		2.865916	2	4	2	110.115	6.51	770000	1500000	1700000	11.51205
Q80UJ7	Rab3 GTPase-activating protein catalytic subunit OS=Mus musculus GN=Rab3gap1 PE=1 SV=4		6.116208	5	11	5	110.128	5.73	2300000	1500000	1700000	27.39014
Q8R307	Vacuolar protein sorting-associated protein 18 homolog OS=Mus musculus GN=Vps18 PE=1 SV=2		0.719424	1	2	1	110.149	6.09	460000		290000	4.141219
Q02788	Collagen alpha-2(VI) chain OS=Mus musculus GN=Col6a2 PE=1 SV=3		12.47582	13	35	13	110.266	6.42	13000000	13000000	12000000	98.04969
Q9ERK4	Exportin-2 OS=Mus musculus GN=Cse1l PE=1 SV=1		8.444902	7	10	7	110.384	5.77	2500000	2600000	1500000	30.61438
O35382	Exocyst complex component 4 OS=Mus musculus GN=Exoc4 PE=1 SV=2		5.74359	4	10	4	110.475	6.49	1900000	1300000	1600000	32.41617
Q6PH08	ERC protein 2 OS=Mus musculus GN=Erc2 PE=1 SV=2		0.835946	1	1	1	110.571	7.03	750000			1.874302
Q7TQH0	Ataxin-2-like protein OS=Mus musculus GN=Atxn2l PE=1 SV=1		12.39276	10	23	10	110.58	8.85	1900000	3100000	2200000	63.47925
A2A432	Cullin-4B OS=Mus musculus GN=Cul4b PE=1 SV=1		13.40206	8	24	6	110.63	8.37	2600000	2400000	3400000	76.17444
Q6NZC7	SEC23-interacting protein OS=Mus musculus GN=Sec23ip PE=1 SV=2		4.90982	3	7	3	110.711	5.94	2000000	1800000	1800000	17.76119
Q8CI71	Syndetin OS=Mus musculus GN=Vps50 PE=1 SV=2		2.593361	2	3	2	111.104	6.07	640000	540000		4.975998
Q9JKR6	Hypoxia up-regulated protein 1 OS=Mus musculus GN=Hyou1 PE=1 SV=1		22.62262	17	45	17	111.112	5.19	8100000	7800000	7100000	131.1697
Q6ZQ03	Formin-binding protein 4 OS=Mus musculus GN=Fnbp4 PE=1 SV=2		1.454898	1	1	1	111.177	4.73		200000		2.711693
P58281	Dynamin-like 120 kDa protein, mitochondrial OS=Mus musculus GN=Opa1 PE=1 SV=1		18.125	15	37	15	111.27	7.55	6900000	4700000	3900000	108.6895

Q8C7X2	ER membrane protein complex subunit 1 OS=Mus musculus GN=Emc1 PE=1 SV=1		2.306921	2	3	2	111.535	7.43	2400000	2200000	570000	8.610621
Q8BWW9	Serine/threonine-protein kinase N2 OS=Mus musculus GN=Pkn2 PE=1 SV=3		2.746694	2	3	2	111.537	6.32		930000	740000	7.46866
Q6PIC6	Sodium/potassium-transporting ATPase subunit alpha-3 OS=Mus musculus GN=Atp1a3 PE=1 SV=1		39.88154	32	125	18	111.62	5.41	68000000	29000000	26000000	430.8851
Q80YR5	Scaffold attachment factor B2 OS=Mus musculus GN=Safb2 PE=1 SV=2		20.48436	16	39	10	111.77	6.38	2500000	1900000	2900000	132.6918
O55098	Serine/threonine-protein kinase 10 OS=Mus musculus GN=Stk10 PE=1 SV=2		7.556936	6	8	5	111.836	7.15	320000	850000	1600000	20.15646
Q60875	Rho guanine nucleotide exchange factor 2 OS=Mus musculus GN=Arhgef2 PE=1 SV=4		17.86802	14	25	14	111.905	7.25	3900000	3800000	3700000	73.87925
Q6PIE5	Sodium/potassium-transporting ATPase subunit alpha-2 OS=Mus musculus GN=Atp1a2 PE=1 SV=1		37.54902	31	123	15	112.145	5.55	36000000	18000000	22000000	405.3734
Q9ESZ8	General transcription factor II-I OS=Mus musculus GN=Gtf2i PE=1 SV=3		9.418838	8	14	8	112.195	6.55	3500000	3000000	2200000	38.13838
Q8R5H1	Ubiquitin carboxyl-terminal hydrolase 15 OS=Mus musculus GN=Usp15 PE=1 SV=1		3.465851	3	3	3	112.254	5.17	1700000		2100000	7.545375
Q8K337	Type II inositol 1,4,5-trisphosphate 5-phosphatase OS=Mus musculus GN=Inpp5b PE=1 SV=1		1.309164	1	1	1	112.69	5.64	410000			1.868943
Q8BIJ6	Isoleucine--tRNA ligase, mitochondrial OS=Mus musculus GN=Iars2 PE=1 SV=1		2.865613	2	3	2	112.732	6.81	2600000	1900000	610000	9.146911
Q505D1	Serine/threonine-protein phosphatase 6 regulatory ankyrin repeat subunit A OS=Mus musculus GN=Ankrd28 PE=1 SV=1		1.519468	1	2	1	112.826	6.27		990000	1200000	6.783145
F2Z461	E3 ISG15--protein ligase Herc6 OS=Mus musculus GN=Herc6 PE=2 SV=1		10.06979	9	19	9	112.856	7.52	4200000	3200000	3100000	49.95145
Q8VDN2	Sodium/potassium-transporting ATPase subunit alpha-1 OS=Mus musculus GN=Atp1a1 PE=1 SV=1		36.26588	30	122	17	112.91	5.45	43000000	25000000	27000000	406.4519
P97386	DNA ligase 3 OS=Mus musculus GN=Lig3 PE=1 SV=2		3.152709	3	6	3	113	8.98	1000000	900000	870000	16.24038
Q9D071	MMS19 nucleotide excision repair protein homolog OS=Mus musculus GN=Mms19 PE=1 SV=1		0.775946	1	2	1	113.017	6.21	360000	370000		4.587611
P11103	Poly [ADP-ribose] polymerase 1 OS=Mus musculus GN=Parp1 PE=1 SV=3		11.94472	10	19	10	113.028	8.95	5800000	6300000	5700000	50.86067
Q921M4	Golgin subfamily A member 2 OS=Mus musculus GN=Golga2 PE=1 SV=3		6.406406	5	11	5	113.209	5	1100000	1800000	1400000	30.73696
P12960	Contactin-1 OS=Mus musculus GN=Cntn1 PE=1 SV=1		17.05882	12	24	12	113.317	6.16	9600000	2700000	2300000	71.82528
Q5DTM8	E3 ubiquitin-protein ligase BRE1A OS=Mus musculus GN=Rnf20 PE=1 SV=2		5.344296	4	8	4	113.45	5.96	1800000	1500000	1700000	22.88204
P23249	Putative helicase MOV-10 OS=Mus musculus GN=Mov10 PE=1 SV=2		14.24303	11	25	11	113.512	9.01	4500000	4800000	5300000	70.75963
Q61941	NAD(P) transhydrogenase, mitochondrial OS=Mus musculus GN=Nnt PE=1 SV=2		8.287293	7	14	7	113.765	7.64	3600000	3500000	5900000	44.02197
Q8BIE6	FERM domain-containing protein 4A OS=Mus musculus GN=Frmd4a PE=1 SV=2		1.666667	1	1	1	113.809	8.92		850000		2.881995
Q3U319	E3 ubiquitin-protein ligase BRE1B OS=Mus musculus GN=Rnf40 PE=1 SV=2		2.397602	2	4	2	113.897	6.48	1100000	980000	1000000	11.84828
Q64436	Potassium-transporting ATPase alpha chain 1 OS=Mus musculus GN=Atp4a PE=1 SV=3		4.259439	4	16	1	113.942	5.88		3700000		51.14547
Q6NZN0	RNA-binding protein 26 OS=Mus musculus GN=Rbm26 PE=1 SV=2		10.96838	8	14	7	114.073	9.16	1600000	1700000	1600000	38.81586
Q80U58	Pumilio homolog 2 OS=Mus musculus GN=Pum2 PE=1 SV=2		2.908068	3	7	3	114.243	7.08	2700000	1600000	2000000	15.53877
Q7TSF1	Desmoglein-1-beta OS=Mus musculus GN=Dsg1b PE=1 SV=1		2.735849	3	5	3	114.382	4.84	740000		2400000	13.38263
O09159	Lysosomal alpha-mannosidase OS=Mus musculus GN=Man2b1 PE=1 SV=4		1.283317	1	1	1	114.576	8.13	830000			3.081783
Q2PFD7	PH and SEC7 domain-containing protein 3 OS=Mus musculus GN=Psd3 PE=1 SV=2		5.593057	6	9	6	114.651	6.24	4500000	3100000	1800000	22.46996
Q8C2K5	RAS protein activator like-3 OS=Mus musculus GN=Rasal3 PE=1 SV=1		1.056676	1	1	1	114.71	8.98	490000			2.282007
O55143	Sarcoplasmic/endoplasmic reticulum calcium ATPase 2 OS=Mus musculus GN=Atp2a2 PE=1 SV=2		22.79693	21	61	21	114.784	5.34	31000000	25000000	25000000	176.2951
Q9DBR7	Protein phosphatase 1 regulatory subunit 12A OS=Mus musculus GN=Ppp1r12a PE=1 SV=2		17.0068	14	38	13	114.927	5.49	5900000	5000000	5200000	111.8808
P11688	Integrin alpha-5 OS=Mus musculus GN=Itga5 PE=1 SV=3		2.754036	2	2	2	114.971	5.95	360000	320000		5.602353
Q8VD75	Huntingtin-interacting protein 1 OS=Mus musculus GN=Hip1 PE=1 SV=2		5.63654	4	6	3	115.13	5.43	1800000	4800000	2800000	15.64684
Q8K135	Dyslexia-associated protein KIAA0319-like protein OS=Mus musculus GN=Kiaa0319l PE=1 SV=1		1.431298	1	1	1	115.24	6.16	870000			2.348583
P43406	Integrin alpha-V OS=Mus musculus GN=Itgav PE=1 SV=2		10.24904	8	13	8	115.287	5.63	3100000	3600000	2100000	39.85493
Q8K224	RNA cytidine acetyltransferase OS=Mus musculus GN=Nat10 PE=1 SV=1		5.859375	5	9	5	115.346	8.32	1000000	1600000	1900000	27.34438
Q9Z0H8	CAP-Gly domain-containing linker protein 2 OS=Mus musculus GN=Clip2 PE=1 SV=2		8.6915	8	16	6	115.84	6.48	1300000	900000	1800000	42.56106
Q8R5F7	Interferon-induced helicase C domain-containing protein 1 OS=Mus musculus GN=Ifih1 PE=1 SV=1		11.21951	8	21	8	115.898	6.25	2700000	3100000	2800000	65.64256
Q5F2E8	Serine/threonine-protein kinase TAO1 OS=Mus musculus GN=Taok1 PE=1 SV=1		4.595405	4	7	3	115.977	7.55	940000	440000	590000	19.71736
Q91YE6	Importin-9 OS=Mus musculus GN=Ipo9 PE=1 SV=3		2.785783	2	3	2	115.978	4.81		1700000	1200000	8.165702
Q5SYD0	Unconventional myosin-Id OS=Mus musculus GN=Myo1d PE=1 SV=1		2.683897	2	2	2	116.007	9.41		560000	920000	6.555291
Q8BUH8	Sentrin-specific protease 7 OS=Mus musculus GN=Senp7 PE=1 SV=1		2.989392	2	6	2	116.273	6.52	730000	640000	890000	16.05751
P97434	Myosin phosphatase Rho-interacting protein OS=Mus musculus GN=Mprip PE=1 SV=2		12.30469	8	15	8	116.337	6.21	1600000	1200000	1100000	43.71472
Q60597	2-oxoglutarate dehydrogenase, mitochondrial OS=Mus musculus GN=Ogdh PE=1 SV=3		20.0391	16	34	16	116.375	6.83	7700000	5700000	4900000	91.82229
Q64727	Vinculin OS=Mus musculus GN=Vcl PE=1 SV=4		14.1651	12	26	12	116.644	6	4500000	4200000	4300000	77.52469
Q8VDP3	Protein-methionine sulfoxide oxidase MICAL1 OS=Mus musculus GN=Mical1 PE=1 SV=1		6.20229	5	11	5	116.711	6.05	1500000	2400000	2400000	35.36162
Q80X50	Ubiquitin-associated protein 2-like OS=Mus musculus GN=Ubap2l PE=1 SV=1		13.00813	8	15	8	116.728	7.11	3800000	3700000	2600000	45.49382
O88532	Zinc finger RNA-binding protein OS=Mus musculus GN=Zfr PE=1 SV=2		9.962756	8	20	8	116.786	9.04	2400000	2200000	2600000	63.57395
Q8CH25	SAFB-like transcription modulator OS=Mus musculus GN=Sltm PE=1 SV=1		9.990301	6	16	6	116.85	7.72	3900000	5000000	4600000	50.201
Q8CGY8	UDP-N-acetylglucosamine--peptide N-acetylglucosaminyltransferase 110 kDa subunit OS=Mus musculus GN=Ogt PE=1 SV=2		2.294455	2	3	2	116.877	6.7	1200000	1000000	580000	6.988626
Q61136	Serine/threonine-protein kinase PRP4 homolog OS=Mus musculus GN=Prpf4b PE=1 SV=3		7.050645	6	12	6	116.904	10.23	2800000	3300000	3200000	32.71113

P19246	Neurofilament heavy polypeptide OS=Mus musculus GN=Nefh PE=1 SV=3		9.082569	9	24	7	116.924	5.81	4500000	1000000	1700000	66.12466
Q7TMY7	Importin-8 OS=Mus musculus GN=Ipo8 PE=1 SV=3		1.287129	1	1	1	117.003	5.16	230000			2.853015
Q5I012	Putative sodium-coupled neutral amino acid transporter 10 OS=Mus musculus GN=Slc38a10 PE=1 SV=2		2.93578	2	6	2	117.12	5.96	1100000	980000	1300000	19.87758
Q5SUA5	Unconventional myosin-Ig OS=Mus musculus GN=Myo1g PE=1 SV=1		10.83984	7	14	7	117.153	8.59	2300000	2600000	1800000	38.05479
Q8C129	Leucyl-cystinyl aminopeptidase OS=Mus musculus GN=Lnpep PE=1 SV=1		16.09756	11	26	11	117.229	5.96	3100000	4700000	4600000	75.46812
Q8K411	Presequence protease, mitochondrial OS=Mus musculus GN=Pitrm1 PE=1 SV=1		2.220077	2	4	2	117.297	7.2	1600000	1300000	1000000	12.66521
P27546	Microtubule-associated protein 4 OS=Mus musculus GN=Map4 PE=1 SV=3		33.77778	26	110	26	117.357	4.98	22000000	19000000	18000000	342.2572
Q569Z5	Probable ATP-dependent RNA helicase DDX46 OS=Mus musculus GN=Ddx46 PE=1 SV=2		17.63566	12	32	12	117.376	9.26	6300000	6300000	6100000	109.6434
Q8BKG3	Inactive tyrosine-protein kinase 7 OS=Mus musculus GN=Ptk7 PE=1 SV=1		1.224105	1	1	1	117.457	6.84		310000		2.459316
Q9CZU3	Superkiller viralicidic activity 2-like 2 OS=Mus musculus GN=Skiv2l2 PE=1 SV=1		4.326923	4	6	4	117.561	6.4		3100000	2800000	16.01803
Q9JHR7	Insulin-degrading enzyme OS=Mus musculus GN=Ide PE=1 SV=1		2.257115	2	2	2	117.697	6.54		920000		5.62986
Q02053	Ubiquitin-like modifier-activating enzyme 1 OS=Mus musculus GN=Uba1 PE=1 SV=1		14.08318	12	37	12	117.734	5.66	10000000	9600000	8300000	123.4122
Q7TPD0	Integrator complex subunit 3 OS=Mus musculus GN=Ints3 PE=1 SV=2		2.59366	1	3	1	117.862	5.8	450000	410000	320000	11.6277
Q8CDM1	ATPase family AAA domain-containing protein 2 OS=Mus musculus GN=Atad2 PE=1 SV=1		4.134615	3	7	3	117.869	6.89	1900000	1700000	1600000	25.29371
Q8C7R4	Ubiquitin-like modifier-activating enzyme 6 OS=Mus musculus GN=Uba6 PE=1 SV=1		3.703704	3	7	3	117.891	6.11	1000000	890000	370000	20.44052
Q5SXY1	Cytospin-B OS=Mus musculus GN=Specc1 PE=1 SV=2		3.280225	2	4	2	118.029	6.64		770000	1100000	11.73187
E9Q735	Ubiquitin conjugation factor E4 A OS=Mus musculus GN=Ube4a PE=1 SV=1		1.361868	1	2	1	118.123	5.33	480000	1000000		6.259836
Q6NV83	U2 snRNP-associated SURP motif-containing protein OS=Mus musculus GN=U2surp PE=1 SV=3		6.122449	3	9	3	118.188	8.47	4500000	5000000	4800000	38.93649
Q5SFM8	RNA-binding protein 27 OS=Mus musculus GN=Rbm27 PE=1 SV=3		4.90566	4	8	3	118.479	9.19	860000	1200000	820000	21.45328
Q3U2P1	Protein transport protein Sec24A OS=Mus musculus GN=Sec24a PE=1 SV=1		1.743119	2	4	2	118.707	7.83	910000	960000	1200000	9.424447
F8VPU2	FERM, RhoGEF and pleckstrin domain-containing protein 1 OS=Mus musculus GN=Farp1 PE=1 SV=1		10.68702	7	17	7	118.801	7.88	2900000	2300000	1800000	51.57301
Q9JME5	AP-3 complex subunit beta-2 OS=Mus musculus GN=Ap3b2 PE=1 SV=2		5.268022	4	7	4	119.118	5.63	1500000	710000	450000	18.67387
Q8VI75	Importin-4 OS=Mus musculus GN=Ipo4 PE=1 SV=1		1.571165	1	3	1	119.198	5.03	1600000	1700000	1400000	9.945671
Q924T7	E3 ubiquitin-protein ligase RNF31 OS=Mus musculus GN=Rnf31 PE=1 SV=2		1.688555	1	1	1	119.238	6.52		360000		1.90325
Q8R4U7	Leucine zipper protein 1 OS=Mus musculus GN=Luzp1 PE=1 SV=2		1.310861	1	1	1	119.239	7.99			270000	2.984565
Q69ZS0	E3 ubiquitin-protein ligase PDZRN3 OS=Mus musculus GN=Pdzrn3 PE=1 SV=3		1.034807	1	2	1	119.326	6.05		750000	560000	4.973473
P13595	Neural cell adhesion molecule 1 OS=Mus musculus GN=Ncam1 PE=1 SV=3		14.97758	14	38	14	119.353	4.83	15000000	8100000	7000000	112.4875
Q9JKY5	Huntingtin-interacting protein 1-related protein OS=Mus musculus GN=Hip1r PE=1 SV=2		4.775281	4	7	4	119.353	6.52	940000	900000	1200000	18.65722
Q9EPL8	Importin-7 OS=Mus musculus GN=Ipo7 PE=1 SV=2		3.853565	3	10	3	119.41	4.82	1300000	1500000	1200000	29.09492
Q91V92	ATP-citrate synthase OS=Mus musculus GN=Acly PE=1 SV=1		11.09074	10	21	10	119.651	7.44	4400000	3900000	3800000	52.37289
Q920B9	FACT complex subunit SPT16 OS=Mus musculus GN=Supt16h PE=1 SV=2		17.57402	17	39	17	119.749	5.66	8100000	11000000	11000000	111.3034
B1AQJ2	Ubiquitin carboxyl-terminal hydrolase 36 OS=Mus musculus GN=Usp36 PE=1 SV=1		1.730419	1	1	1	119.839	9.82		120000		2.331981
Q640N3	Rho GTPase-activating protein 30 OS=Mus musculus GN=Arhgap30 PE=1 SV=3		3.269755	2	2	2	120.04	4.84	570000	270000		5.68298
P53569	CCAAT/enhancer-binding protein zeta OS=Mus musculus GN=Cebpz PE=1 SV=2		6.749049	5	11	5	120.187	5.74	1200000	980000	1200000	32.04517
Q62036	Centrosomal protein of 131 kDa OS=Mus musculus GN=Cep131 PE=1 SV=2		1.132075	1	1	1	120.24	8.72		260000		2.281698
Q9JIS8	Solute carrier family 12 member 4 OS=Mus musculus GN=Slc12a4 PE=1 SV=2		0.737327	1	3	1	120.546	6.67	1800000	3500000	3700000	7.103465
Q3V0C5	Ubiquitin carboxyl-terminal hydrolase 48 OS=Mus musculus GN=Usp48 PE=1 SV=2		1.61597	1	3	1	120.554	6.25	1100000	1300000	1000000	12.22654
O55201	Transcription elongation factor SPT5 OS=Mus musculus GN=Supt5h PE=1 SV=1		17.28281	11	26	11	120.589	5.05	2500000	2500000	2500000	88.41743
A2AWA9	Rab GTPase-activating protein 1 OS=Mus musculus GN=Rabgap1 PE=1 SV=1		1.12782	1	3	1	120.722	5.25	830000		770000	8.894099
Q91Z67	SLIT-ROBO Rho GTPase-activating protein 2 OS=Mus musculus GN=Srgap2 PE=1 SV=2		2.054155	2	2	2	120.723	6.64		640000		4.841993
Q9Z1R2	Large proline-rich protein BAG6 OS=Mus musculus GN=Bag6 PE=1 SV=1		0.693241	1	1	1	120.962	5.71			790000	1.994192
Q6ZQ58	La-related protein 1 OS=Mus musculus GN=Larp1 PE=1 SV=3		2.238806	2	4	2	121.05	8.79	6300000	3500000	4800000	11.618
Q61147	Ceruloplasmin OS=Mus musculus GN=Cp PE=1 SV=2		6.409048	6	13	6	121.074	5.85	2000000	2300000	2700000	33.38637
Q8CCP0	Nuclear export mediator factor Nemf OS=Mus musculus GN=Nemf PE=1 SV=2		3.383459	3	3	3	121.112	6.8	2300000	950000		8.792335
O88343	Electrogenic sodium bicarbonate cotransporter 1 OS=Mus musculus GN=Slc4a4 PE=1 SV=2		1.204819	1	1	1	121.406	6.84	1000000			2.614978
Q6R0H7	Guanine nucleotide-binding protein G(s) subunit alpha isoforms XLas OS=Mus musculus GN=Gnas PE=1 SV=1		7.855252	7	19	6	121.429	4.81	12000000	7100000	9100000	51.09094
Q3U7R1	Extended synaptotagmin-1 OS=Mus musculus GN=Esyt1 PE=1 SV=2		14.74359	14	34	14	121.478	5.95	6800000	6900000	7000000	91.1161
Q91ZW3	SWI/SNF-related matrix-associated actin-dependent regulator of chromatin subfamily A member 5 OS=Mus musculus GN=Smarca5		11.79829	11	29	11	121.55	8.15	18000000	17000000	19000000	93.24235
Q6A0A9	Constitutive coactivator of PPAR-gamma-like protein 1 OS=Mus musculus GN=FAM120A PE=1 SV=2		11.69065	9	19	9	121.569	8.92	7600000	5000000	6800000	62.73837
Q8BTI9	Phosphatidylinositol 4,5-bisphosphate 3-kinase catalytic subunit beta isoform OS=Mus musculus GN=Pik3cb PE=1 SV=2		1.409774	1	1	1	121.633	7.09			400000	3.182452
Q99ME2	WD repeat-containing protein 6 OS=Mus musculus GN=Wdr6 PE=1 SV=1		1.244444	1	1	1	121.82	6.98		330000		3.161793
Q9WTI7	Unconventional myosin-Ic OS=Mus musculus GN=Myo1c PE=1 SV=2		6.020696	4	4	4	121.868	9.35	1200000	1100000		10.42216
Q9JL26	Formin-like protein 1 OS=Mus musculus GN=Fmnl1 PE=1 SV=1		3.199269	3	6	3	121.983	5.82	1500000	1000000	1100000	16.43171

Q61739	Integrin alpha-6 OS=Mus musculus GN=Itga6 PE=1 SV=3		6.782768	5	14	5	122.082	7.03	2400000	2400000	2000000	40.04307
Q80U87	Ubiquitin carboxyl-terminal hydrolase 8 OS=Mus musculus GN=Usp8 PE=1 SV=2		1.388889	1	2	1	122.534	8.47	590000	900000		7.864779
P51829	Adenylate cyclase type 7 OS=Mus musculus GN=Adcy7 PE=2 SV=2		1.637853	1	1	1	122.628	7.87			260000	2.251718
Q9Z1T1	AP-3 complex subunit beta-1 OS=Mus musculus GN=Ap3b1 PE=1 SV=2		5.61086	6	15	6	122.664	5.66	3300000	3600000	3100000	38.54547
Q8K298	Actin-binding protein anillin OS=Mus musculus GN=Anln PE=1 SV=2		7.93934	7	12	7	122.718	6.98	1400000	1800000	1700000	36.57955
P97496	SWI/SNF complex subunit SMARCC1 OS=Mus musculus GN=Smarcc1 PE=1 SV=2		12.40942	10	26	7	122.813	5.73	3300000	3000000	1700000	70.70669
Q3TBD2	Minor histocompatibility protein HA-1 OS=Mus musculus GN=Hmha1 PE=1 SV=2		4.032258	4	10	4	122.825	5.94	890000	860000	1200000	35.08237
Q6P5F9	Exportin-1 OS=Mus musculus GN=Xpo1 PE=1 SV=1		8.496732	8	14	8	123.013	6.07	3300000	5200000	4900000	42.52028
A2APV2	Formin-like protein 2 OS=Mus musculus GN=Fmnl2 PE=1 SV=2		1.289134	1	1	1	123.024	7.53	950000			2.286639
P34152	Focal adhesion kinase 1 OS=Mus musculus GN=Ptk2 PE=1 SV=3		3.761468	4	6	4	123.458	6.68	890000	1000000		14.25843
Q8BKC5	Importin-5 OS=Mus musculus GN=Ipo5 PE=1 SV=3		7.383774	5	14	5	123.511	4.93	3500000	4000000	3400000	48.16071
Q8CGF7	Transcription elongation regulator 1 OS=Mus musculus GN=Tcerg1 PE=1 SV=2		8.727273	10	22	10	123.71	8.65	2800000	3100000	3000000	60.42112
P52431	DNA polymerase delta catalytic subunit OS=Mus musculus GN=Pold1 PE=1 SV=2		1.538462	1	2	1	123.712	7.61	2700000	2200000		7.223793
Q9EPK7	Exportin-7 OS=Mus musculus GN=Xpo7 PE=1 SV=3		1.287948	1	1	1	123.731	6.38			530000	3.008579
Q9EPU0	Regulator of nonsense transcripts 1 OS=Mus musculus GN=Upf1 PE=1 SV=2		10.67616	9	20	9	123.889	6.61	3600000	3500000	3600000	54.60206
Q80U95	Ubiquitin-protein ligase E3C OS=Mus musculus GN=Ube3c PE=1 SV=2		0.923361	1	1	1	123.896	6.39				1.806488
P59328	WD repeat and HMG-box DNA-binding protein 1 OS=Mus musculus GN=Wdhd1 PE=1 SV=2		1.253357	1	1	1	124.176	5.63		690000		2.399252
Q9Z103	Activity-dependent neuroprotector homeobox protein OS=Mus musculus GN=Adnp PE=1 SV=2		5.505415	5	11	5	124.229	6.86	2200000	2500000	2200000	27.80571
O08532	Voltage-dependent calcium channel subunit alpha-2/delta-1 OS=Mus musculus GN=Cacna2d1 PE=1 SV=1		2.629193	2	2	2	124.551	5.3	1800000			5.450298
O70566	Protein diaphanous homolog 2 OS=Mus musculus GN=Diaph2 PE=1 SV=2		2.003643	2	2	2	124.792	6.92	7000000		850000	5.242061
Q923Q2	StAR-related lipid transfer protein 13 OS=Mus musculus GN=Stard13 PE=1 SV=5		2.246181	2	3	2	124.982	7.53	400000	350000	560000	6.704082
Q5NC05	Transcription termination factor 2 OS=Mus musculus GN=Ttf2 PE=1 SV=2		1.142355	1	3	1	125.452	8.9	320000	380000	400000	6.827679
Q8K394	Inactive phospholipase C-like protein 2 OS=Mus musculus GN=Plcl2 PE=1 SV=2		1.241135	1	1	1	125.692	6.92	630000			1.8608
Q8C0D5	Elongation factor-like GTPase 1 OS=Mus musculus GN=Efl1 PE=1 SV=1		5.767524	4	4	4	125.697	6.16	2700000	340000	580000	10.62759
Q9Z2V5	Histone deacetylase 6 OS=Mus musculus GN=Hdac6 PE=1 SV=3		1.218451	1	3	1	125.706	5.78	1200000	1000000	1000000	10.32543
P70248	Unconventional myosin-If OS=Mus musculus GN=Myo1f PE=1 SV=1		5.186533	5	12	3	125.868	8.92	2300000	1900000	1200000	32.6337
P35601	Replication factor C subunit 1 OS=Mus musculus GN=Rfc1 PE=1 SV=2		6.189213	5	12	5	125.907	9.33	1600000	1600000	1500000	39.83547
Q91V14	Solute carrier family 12 member 5 OS=Mus musculus GN=Slc12a5 PE=1 SV=2		3.866432	4	7	4	126.189	6.74	4800000	2300000	1300000	16.84027
Q8VI93	2'-5'-oligoadenylate synthase 3 OS=Mus musculus GN=Oas3 PE=1 SV=1		2.811951	2	5	2	126.253	8.72	2000000	1500000	1600000	13.94967
Q99P72	Reticulon-4 OS=Mus musculus GN=Rtn4 PE=1 SV=2		14.80207	10	27	10	126.535	4.54	19000000	14000000	12000000	93.06523
Q9ESC8	AF4/FMR2 family member 4 OS=Mus musculus GN=Aff4 PE=1 SV=1		1.12069	1	1	1	126.562	9.36			370000	2.163867
Q8CHC8	ADNP homeobox protein 2 OS=Mus musculus GN=Adnp2 PE=2 SV=2		1.716738	1	2	1	126.687	9		690000		5.607748
E9Q634	Unconventional myosin-Ie OS=Mus musculus GN=Myo1e PE=1 SV=1		3.161698	3	10	1	126.738	9.07	350000	460000	430000	32.82359
Q3U1J4	DNA damage-binding protein 1 OS=Mus musculus GN=Ddb1 PE=1 SV=2		2.45614	2	5	2	126.772	5.26	720000	2300000	490000	15.4431
Q924W5	Structural maintenance of chromosomes protein 6 OS=Mus musculus GN=Smc6 PE=1 SV=1		1.002735	1	1	1	127.118	7.17	720000			1.85557
Q9QWY8	Arf-GAP with SH3 domain, ANK repeat and PH domain-containing protein 1 OS=Mus musculus GN=Asap1 PE=1 SV=2		1.307759	1	2	1	127.342	7.64	1300000	1500000		6.777847
P05555	Integrin alpha-M OS=Mus musculus GN=Itgam PE=1 SV=2		2.515178	3	7	3	127.4	7.27	3200000	2600000	2900000	16.3056
P24063	Integrin alpha-L OS=Mus musculus GN=Itgal PE=1 SV=2		9.88822	9	22	9	128.247	6.11	3700000	3000000	3400000	64.85117
Q99MI1	ELKS/Rab6-interacting/CAST family member 1 OS=Mus musculus GN=Erc1 PE=1 SV=1		4.464286	4	9	4	128.252	5.87	1200000	880000	690000	24.27982
Q640N1	Adipocyte enhancer-binding protein 1 OS=Mus musculus GN=Aebp1 PE=1 SV=1		11.79078	9	19	9	128.284	5.08	3800000	3400000	3200000	58.43533
Q6A4J8	Ubiquitin carboxyl-terminal hydrolase 7 OS=Mus musculus GN=Usp7 PE=1 SV=1		7.887579	8	16	8	128.393	5.5	2900000	2700000	3200000	41.22472
Q9DBV3	Probable ATP-dependent RNA helicase DHX34 OS=Mus musculus GN=Dhx34 PE=1 SV=2		2.358079	1	1	1	128.427	8.16				2.658601
P46735	Unconventional myosin-Ib OS=Mus musculus GN=Myo1b PE=1 SV=3		2.168022	2	3	2	128.483	9.26		1600000	1600000	6.840855
Q8R0G9	Nuclear pore complex protein Nup133 OS=Mus musculus GN=Nup133 PE=1 SV=2		8.311688	7	15	7	128.539	5.2	2000000	1700000	1500000	42.75343
Q810B6	Rabankyrin-5 OS=Mus musculus GN=Ankfy1 PE=1 SV=2		1.283148	1	3	1	128.571	5.91	1100000	1200000	1200000	10.54433
P28660	Nck-associated protein 1 OS=Mus musculus GN=Nckap1 PE=1 SV=2		6.826241	7	12	7	128.7	6.62	4200000	3300000	2900000	36.987
Q9EPR5	VPS10 domain-containing receptor SorCS2 OS=Mus musculus GN=Sorcs2 PE=1 SV=2		0.69025	1	1	1	128.822	7.34	440000			2.325137
P98195	Probable phospholipid-transporting ATPase IIB OS=Mus musculus GN=Atp9b PE=1 SV=4		2.792321	2	3	2	128.934	7.64			550000	8.780249
Q9QXH4	Integrin alpha-X OS=Mus musculus GN=Itgax PE=1 SV=1		4.27716	4	8	4	129.069	6.64	2500000	2700000	1900000	23.47733
Q8R5K4	Nucleolar protein 6 OS=Mus musculus GN=Nol6 PE=2 SV=2		3.559028	3	6	3	129.146	6.79	1500000	1100000	1400000	12.4478
Q01149	Collagen alpha-2(I) chain OS=Mus musculus GN=Col1a2 PE=1 SV=2		10.86006	11	39	11	129.478	9.19	94000000	1.6E+08	91000000	111.0358
P35441	Thrombospondin-1 OS=Mus musculus GN=Thbs1 PE=1 SV=1		7.264957	6	9	6	129.564	4.96	990000	1500000	970000	26.26412
Q05920	Pyruvate carboxylase, mitochondrial OS=Mus musculus GN=Pc PE=1 SV=1		1.697793	2	4	2	129.602	6.71	2100000	2300000	2500000	9.633676

Q6P6L0	Filamin A-interacting protein 1-like OS=Mus musculus GN=Filip1l PE=1 SV=2		1.768347	2	2	2	129.692	6.37	690000		630000	5.196905
Q03350	Thrombospondin-2 OS=Mus musculus GN=Thbs2 PE=1 SV=2		1.706485	1	2	1	129.798	4.82	300000	490000		4.746555
Q80VP0	Tectonin beta-propeller repeat-containing protein 1 OS=Mus musculus GN=Tecpr1 PE=1 SV=1		3.430532	2	4	2	130.184	6.48	810000	460000		13.02361
Q9D2D7	Zinc finger protein 687 OS=Mus musculus GN=Znf687 PE=1 SV=1		1.212611	1	2	1	130.268	8.12	220000	440000		5.215075
Q99MU3	Double-stranded RNA-specific adenosine deaminase OS=Mus musculus GN=Adar PE=1 SV=2		3.480475	2	4	2	130.365	8.7	1500000	1300000	1600000	11.4729
Q3V3R4	Integrin alpha-1 OS=Mus musculus GN=Itga1 PE=1 SV=2		2.8838	3	5	3	130.727	6.2	2800000	2000000	1000000	12.99503
Q6DFV1	Condensin-2 complex subunit G2 OS=Mus musculus GN=Ncapg2 PE=1 SV=2		1.230228	1	1	1	130.809	6.93		210000		2.357386
P55012	Solute carrier family 12 member 2 OS=Mus musculus GN=Slc12a2 PE=1 SV=2		4.39834	4	8	4	130.95	7.33	1400000	990000	1100000	21.42509
Q9Z0U1	Tight junction protein ZO-2 OS=Mus musculus GN=Tjp2 PE=1 SV=2		11.13967	10	17	10	131.2	6.79	1800000	1700000	1000000	48.2029
P70704	Phospholipid-transporting ATPase IA OS=Mus musculus GN=Atp8a1 PE=1 SV=2		3.092784	3	7	3	131.33	6.84	4400000	2700000	2500000	21.1564
P27046	Alpha-mannosidase 2 OS=Mus musculus GN=Man2a1 PE=1 SV=2		1.130435	1	3	1	131.548	8.03	800000	790000	1000000	9.241861
P37889	Fibulin-2 OS=Mus musculus GN=Fbln2 PE=1 SV=2		3.603604	3	4	3	131.746	4.68	810000	590000	460000	11.27339
Q8BX90	Fibronectin type-III domain-containing protein 3A OS=Mus musculus GN=Fndc3a PE=1 SV=3		2.003339	2	2	2	131.874	6.64			1400000	4.482981
Q8CH18	Cell division cycle and apoptosis regulator protein 1 OS=Mus musculus GN=Ccar1 PE=1 SV=1		13.08901	9	18	9	131.979	5.76	4400000	4900000	4600000	51.01702
Q9EPE9	Manganese-transporting ATPase 13A1 OS=Mus musculus GN=Atp13a1 PE=1 SV=2		10.58333	11	26	11	132.303	8.03	4800000	5500000	5500000	67.20984
Q9R0K7	Plasma membrane calcium-transporting ATPase 2 OS=Mus musculus GN=Atp2b2 PE=1 SV=2		18.36394	16	38	11	132.503	5.96	8900000	4400000	3200000	126.0956
Q6PDG5	SWI/SNF complex subunit SMARCC2 OS=Mus musculus GN=Smarcc2 PE=1 SV=2		15.41632	16	36	13	132.522	5.59	3600000	4600000	3400000	101.6093
P59997	Lysine-specific demethylase 2A OS=Mus musculus GN=Kdm2a PE=1 SV=2		2.239449	2	3	2	132.594	7.44	300000	420000		7.073794
Q6NWW9	Fibronectin type III domain-containing protein 3B OS=Mus musculus GN=Fndc3b PE=1 SV=1		2.402651	2	3	2	132.679	6.29	1500000	1500000	730000	7.257413
Q8BHG1	Nardilysin OS=Mus musculus GN=Nrdc PE=1 SV=1		1.464255	1	2	1	132.808	4.87		660000	660000	6.046897
Q6P9Q6	FK506-binding protein 15 OS=Mus musculus GN=Fkbp15 PE=1 SV=2		9.621711	8	13	8	132.878	5.07	2100000	1200000	970000	40.75704
Q6Q477	Plasma membrane calcium-transporting ATPase 4 OS=Mus musculus GN=Atp2b4 PE=1 SV=1		15.93361	13	35	6	132.984	6.13	5800000	2700000	1800000	122.6448
Q8BYA0	Tubulin-specific chaperone D OS=Mus musculus GN=Tbcd PE=1 SV=1		1.923077	2	4	2	133.236	6.51	880000	1100000	1000000	12.94579
P52332	Tyrosine-protein kinase JAK1 OS=Mus musculus GN=Jak1 PE=1 SV=1		1.040763	1	1	1	133.282	7.59			1700000	2.855462
Q62018	RNA polymerase-associated protein CTR9 homolog OS=Mus musculus GN=Ctr9 PE=1 SV=2		3.239557	3	6	3	133.326	6.49	1700000	2000000	1600000	16.63023
Q6GQT9	Nodal modulator 1 OS=Mus musculus GN=Nomo1 PE=1 SV=1		8.896211	8	17	8	133.336	6.09	6100000	5100000	6000000	53.03015
P60469	Liprin-alpha-3 OS=Mus musculus GN=Ppfia3 PE=1 SV=2		1.005025	1	1	1	133.344	5.64	470000			2.897202
Q80UK8	Integrator complex subunit 2 OS=Mus musculus GN=Ints2 PE=1 SV=2		2.253756	1	1	1	133.429	6.11			470000	3.579299
Q9ES52	Phosphatidylinositol 3,4,5-trisphosphate 5-phosphatase 1 OS=Mus musculus GN=Inpp5d PE=1 SV=2		6.968934	7	13	7	133.458	7.8	2000000	1900000	1700000	42.17499
Q3UPL0	Protein transport protein Sec31A OS=Mus musculus GN=Sec31a PE=1 SV=2		5.121951	4	9	4	133.486	6.76	2000000	2400000	2600000	26.90719
Q61543	Golgi apparatus protein 1 OS=Mus musculus GN=Glg1 PE=1 SV=1		16	15	33	15	133.646	6.84	5000000	4000000	3100000	92.49106
Q5U4C3	Splicing factor, arginine/serine-rich 19 OS=Mus musculus GN=Scaf1 PE=1 SV=1		2.229299	2	4	2	133.761	9.45	460000	2200000	3800000	9.405379
Q8CFI7	DNA-directed RNA polymerase II subunit RPB2 OS=Mus musculus GN=Polr2b PE=1 SV=2		7.666099	6	15	6	133.825	6.87	2500000	3600000	3500000	50.10248
Q8BMI0	F-box only protein 38 OS=Mus musculus GN=Fbxo38 PE=1 SV=1		1.59129	1	3	1	133.842	6.09	1100000	1000000	950000	12.0921
Q8BND3	WD repeat-containing protein 35 OS=Mus musculus GN=Wdr35 PE=1 SV=3		1.27011	1	1	1	133.905	6.34	210000			2.215543
Q65Z40	Wings apart-like protein homolog OS=Mus musculus GN=Wapl PE=1 SV=2		5.5	4	6	4	133.986	5.41	1100000	1000000	1000000	13.71204
Q8C2E7	WASH complex subunit strumpellin OS=Mus musculus GN=Kiaa0196 PE=1 SV=2		2.157032	2	2	2	134.025	7.12		680000		5.534799
Q8BMJ2	Leucine--tRNA ligase, cytoplasmic OS=Mus musculus GN=Lars PE=1 SV=2		6.791171	7	17	7	134.106	7.05	4700000	4000000	3900000	52.00894
Q8CG48	Structural maintenance of chromosomes protein 2 OS=Mus musculus GN=Smc2 PE=1 SV=2		10.83123	11	23	11	134.156	8.41	3800000	4100000	3800000	64.43835
Q8C419	Probable G-protein coupled receptor 158 OS=Mus musculus GN=Gpr158 PE=1 SV=2		1.333333	1	1	1	134.341	8.09				2.467273
Q8CDG3	Deubiquitinating protein VCIP135 OS=Mus musculus GN=Vcpip1 PE=1 SV=1		2.377049	2	4	2	134.418	7.17	1400000	1000000	1700000	10.90785
G5E829	Plasma membrane calcium-transporting ATPase 1 OS=Mus musculus GN=Atp2b1 PE=1 SV=1		20.08197	19	54	12	134.662	5.91	15000000	8100000	7800000	185.0052
Q6NWV3	Intraflagellar transport protein 122 homolog OS=Mus musculus GN=Ift122 PE=1 SV=1		1.43824	1	1	1	134.712	6.98				2.490688
Q9QWI6	SRC kinase signaling inhibitor 1 OS=Mus musculus GN=Srcin1 PE=1 SV=2		4.56	4	7	4	134.776	9.32	1800000	780000	1000000	17.52816
O35954	Membrane-associated phosphatidylinositol transfer protein 1 OS=Mus musculus GN=Pitpnm1 PE=1 SV=1		2.735318	1	1	1	134.855	6.06	570000			4.088769
O35927	Catenin delta-2 OS=Mus musculus GN=Ctnnd2 PE=1 SV=1		2.405774	2	2	2	134.916	7.65	600000			4.879312
O08784	Treacle protein OS=Mus musculus GN=Tcof1 PE=1 SV=1		7.878788	8	16	8	134.921	9.35	5600000	6100000	5900000	47.13845
P70232	Neural cell adhesion molecule L1-like protein OS=Mus musculus GN=Chl1 PE=1 SV=2		11.91067	8	16	8	134.99	5.57	3700000	2700000	2300000	44.81159
O54774	AP-3 complex subunit delta-1 OS=Mus musculus GN=Ap3d1 PE=1 SV=1		7.923269	8	23	8	134.996	7.37	6200000	5700000	7000000	69.64563
Q921M3	Splicing factor 3B subunit 3 OS=Mus musculus GN=Sf3b3 PE=1 SV=1		13.72227	14	36	14	135.465	5.26	8300000	7500000	12000000	99.35413
Q8K1X1	WD repeat-containing protein 11 OS=Mus musculus GN=Wdr11 PE=1 SV=1		2.861815	3	4	3	135.851	7.11	980000	1000000	1000000	8.628666
Q6ZQ38	Cullin-associated NEDD8-dissociated protein 1 OS=Mus musculus GN=Cand1 PE=1 SV=2		10.0813	11	23	11	136.245	5.78	9600000	8600000	6900000	67.39534
Q3UMB9	WASH complex subunit 7 OS=Mus musculus GN=Kiaa1033 PE=1 SV=2		5.11509	5	8	5	136.283	7.37	1000000	1500000	1800000	22.80892

O70305	Ataxin-2 OS=Mus musculus GN=Atxn2 PE=1 SV=1		2.101167	2	2	2	136.4	9.55		700000		5.493347
P10493	Nidogen-1 OS=Mus musculus GN=Nid1 PE=1 SV=2		6.746988	7	20	6	136.45	5.44	4800000	5500000	4400000	58.09206
Q9R0G7	Zinc finger E-box-binding homeobox 2 OS=Mus musculus GN=Zeb2 PE=1 SV=2		3.374486	3	6	3	136.529	6.34	1900000	580000	480000	14.27188
Q99PU8	Putative ATP-dependent RNA helicase DHX30 OS=Mus musculus GN=Dhx30 PE=1 SV=1		0.657354	1	1	1	136.583	8.75			220000	1.958907
Q64455	Receptor-type tyrosine-protein phosphatase eta OS=Mus musculus GN=Ptprj PE=1 SV=2		1.373183	1	2	1	136.683	5.57	480000	450000		6.701038
Q922B9	Sperm-specific antigen 2 homolog OS=Mus musculus GN=Ssfa2 PE=1 SV=3		1.996805	2	3	2	136.862	5.26	67000000	420000	650000	7.746915
P55066	Neurocan core protein OS=Mus musculus GN=Ncan PE=1 SV=1		5.283912	5	12	5	137.114	5.72	3400000	2300000	1000000	24.33828
Q3UMF0	Cordon-bleu protein-like 1 OS=Mus musculus GN=Cobll1 PE=1 SV=2		0.942655	1	1	1	137.296	8.16		770000		2.621943
Q9R1X4	Protein timeless homolog OS=Mus musculus GN=Timeless PE=1 SV=3		0.835422	1	1	1	137.417	5.49		180000		2.461619
Q05D44	Eukaryotic translation initiation factor 5B OS=Mus musculus GN=Eif5b PE=1 SV=2		18.17434	18	38	18	137.532	5.59	4900000	4800000	4800000	118.3473
Q810U3	Neurofascin OS=Mus musculus GN=Nfasc PE=1 SV=1		11.37097	10	23	10	137.889	6.19	10000000	5500000	5100000	61.51613
P11087	Collagen alpha-1(I) chain OS=Mus musculus GN=Col1a1 PE=1 SV=4		9.015829	9	50	9	137.948	5.85	1.8E+08	2.4E+08	2.1E+08	177.5384
Q9Z1B3	1-phosphatidylinositol 4,5-bisphosphate phosphodiesterase beta-1 OS=Mus musculus GN=Plcb1 PE=1 SV=2		12.58224	12	25	12	138.309	6.13	4000000	2500000	2500000	74.72405
Q8CIE6	Coatomer subunit alpha OS=Mus musculus GN=Copa PE=1 SV=2		16.66667	17	44	17	138.344	7.65	19000000	17000000	16000000	127.1603
P04258	SWISS-PROT:P04258 (Bos taurus) Similar to Collagen alpha 1(III) chain	x	3.61528	4	33	3	138.354	6.43	1.9E+08	2.2E+08	1.8E+08	123.1763
Q810U4	Neuronal cell adhesion molecule OS=Mus musculus GN=Nrcam PE=1 SV=2		5.015924	5	8	5	138.435	5.91	2300000	1400000	1200000	21.64701
Q0GNC1	Inverted formin-2 OS=Mus musculus GN=Inf2 PE=1 SV=1		4.79183	4	8	4	138.474	5.21	1700000	1300000	1500000	26.51213
Q7TT18	Activating transcription factor 7-interacting protein 1 OS=Mus musculus GN=Atf7ip PE=1 SV=1		4.211332	4	7	4	138.508	4.77	1200000	1600000	910000	17.9568
P08121	Collagen alpha-1(III) chain OS=Mus musculus GN=Col3a1 PE=1 SV=4		6.898907	7	17	6	138.858	6.52	18000000	24000000	15000000	57.72959
Q69ZW3	EH domain-binding protein 1 OS=Mus musculus GN=Ehbp1 PE=1 SV=3		1.299756	1	1	1	139.019	5.38		440000		3.125922
P06909	Complement factor H OS=Mus musculus GN=Cfh PE=1 SV=2		1.539708	1	2	1	139.047	6.99		550000		4.53601
O08808	Protein diaphanous homolog 1 OS=Mus musculus GN=Diaph1 PE=1 SV=1		1.752988	2	5	2	139.255	5.57	1200000	1200000	900000	13.8248
P51432	1-phosphatidylinositol 4,5-bisphosphate phosphodiesterase beta-3 OS=Mus musculus GN=Plcb3 PE=1 SV=2		1.458671	1	2	1	139.4	5.94	180000	410000		6.268155
Q5SV85	Synergin gamma OS=Mus musculus GN=Synrg PE=1 SV=1		3.522205	3	8	3	139.528	5.03	760000	680000	730000	23.31451
Q64514	Tripeptidyl-peptidase 2 OS=Mus musculus GN=Tpp2 PE=1 SV=3		12.67829	12	29	12	139.791	6.58	3600000	3100000	2300000	87.70089
Q9Z1Q9	Valine--tRNA ligase OS=Mus musculus GN=Vars PE=1 SV=1		20.34838	23	63	23	140.127	7.77	22000000	22000000	21000000	188.3117
O35206	Collagen alpha-1(XV) chain OS=Mus musculus GN=Col15a1 PE=1 SV=2		1.02414	1	1	1	140.385	4.89	160000			2.755358
P97820	Mitogen-activated protein kinase kinase kinase kinase 4 OS=Mus musculus GN=Map4k4 PE=1 SV=1		3.163017	3	10	1	140.515	7.47	660000	230000		30.14993
Q8BRT1	CLIP-associating protein 2 OS=Mus musculus GN=Clasp2 PE=1 SV=1		4.587869	4	5	4	140.652	8.63	2500000	1600000	4100000	17.40478
Q6ZPZ3	Zinc finger CCCH domain-containing protein 4 OS=Mus musculus GN=Zc3h4 PE=1 SV=2		5.521472	4	15	4	140.88	6.27	2000000	2100000	1900000	51.59539
P11627	Neural cell adhesion molecule L1 OS=Mus musculus GN=L1cam PE=1 SV=1		0.952381	1	1	1	140.881	5.97				2.61327
O88879	Apoptotic protease-activating factor 1 OS=Mus musculus GN=Apaf1 PE=1 SV=3		1.441153	2	2	2	140.913	6.43		890000		4.405502
Q925H1	Zinc finger transcription factor Trps1 OS=Mus musculus GN=Trps1 PE=1 SV=1		1.483216	1	3	1	140.946	7.59	290000	270000	280000	9.40628
O35638	Cohesin subunit SA-2 OS=Mus musculus GN=Stag2 PE=1 SV=3		2.680747	3	3	3	141.19	5.43	1300000			3.925761
O54824	Pro-interleukin-16 OS=Mus musculus GN=Il16 PE=1 SV=3		1.134644	1	1	1	141.347	7.78		440000		3.046257
O54988	STE20-like serine/threonine-protein kinase OS=Mus musculus GN=Slk PE=1 SV=2		6.163828	5	11	4	141.37	5.14	1900000	1800000	1400000	34.39672
Q9CW03	Structural maintenance of chromosomes protein 3 OS=Mus musculus GN=Smc3 PE=1 SV=2		20.46015	21	50	21	141.468	7.18	4400000	4700000	4800000	156.8755
O08788	Dynactin subunit 1 OS=Mus musculus GN=Dctn1 PE=1 SV=3		16.15925	17	42	17	141.588	5.9	6900000	5100000	5100000	118.9258
Q8VDJ3	Vigilin OS=Mus musculus GN=Hdlbp PE=1 SV=1		15.9306	16	32	16	141.655	6.87	9300000	8300000	8800000	98.32987
Q5SVR0	TBC1 domain family member 9B OS=Mus musculus GN=Tbc1d9b PE=1 SV=1		2.29612	2	3	2	141.69	5.29		750000		8.746991
P28481	Collagen alpha-1(II) chain OS=Mus musculus GN=Col2a1 PE=1 SV=2		5.245461	7	35	6	141.886	6.92	95000000	81000000	50000000	132.526
Q80X82	Symplekin OS=Mus musculus GN=Sympk PE=1 SV=1		2.725857	3	4	3	142.194	6.05		1300000	1100000	10.2934
O35604	Niemann-Pick C1 protein OS=Mus musculus GN=Npc1 PE=1 SV=2		0.783085	1	2	1	142.791	5.71	930000		1000000	4.080373
Q3UYK3	TBC1 domain family member 9 OS=Mus musculus GN=Tbc1d9 PE=2 SV=2		0.870253	1	1	1	142.933	5.34				0
Q62417	Sorbin and SH3 domain-containing protein 1 OS=Mus musculus GN=Sorbs1 PE=1 SV=2		3.643411	4	10	4	142.982	8.25	1900000	820000	1100000	27.52734
Q6P5B0	RRP12-like protein OS=Mus musculus GN=Rrp12 PE=1 SV=1		5.791506	5	9	5	143.041	8.91	2300000	1000000	2600000	25.97486
Q9CU62	Structural maintenance of chromosomes protein 1A OS=Mus musculus GN=Smc1a PE=1 SV=4		16.3017	17	34	17	143.146	7.64	4100000	5400000	4300000	94.85619
Q8BSS9	Liprin-alpha-2 OS=Mus musculus GN=Ppfia2 PE=1 SV=2		1.670644	1	1	1	143.146	6.11				2.6743
Q99KY4	Cyclin-G-associated kinase OS=Mus musculus GN=Gak PE=1 SV=2		0.91954	1	1	1	143.55	5.73			1500000	1.848079
Q8BU30	Isoleucine--tRNA ligase, cytoplasmic OS=Mus musculus GN=Iars PE=1 SV=2		5.071315	5	10	5	144.179	6.55	2800000	2300000	3000000	30.37848
P06800	Receptor-type tyrosine-protein phosphatase C OS=Mus musculus GN=Ptprc PE=1 SV=3		16.96359	19	55	19	144.514	5.94	10000000	14000000	12000000	181.1228
Q9JJ28	Protein flightless-1 homolog OS=Mus musculus GN=Flii PE=1 SV=1		4.720692	5	9	5	144.712	6.06	2500000	2700000	1600000	25.79505
Q61137	Astrotactin-1 OS=Mus musculus GN=Astn1 PE=1 SV=4		1.382488	1	1	1	144.791	5.21	520000			1.994277

Q3U962	Collagen alpha-2(V) chain OS=Mus musculus GN=Col5a2 PE=1 SV=1		3.072812	2	6	1	144.929	6.7	2800000	3400000	1600000	20.51311
Q60520	Paired amphipathic helix protein Sin3a OS=Mus musculus GN=Sin3a PE=1 SV=3		4.945055	5	6	5	144.998	7.25	1800000	1000000	1100000	16.92209
Q7TMB8	Cytoplasmic FMR1-interacting protein 1 OS=Mus musculus GN=Cyfip1 PE=1 SV=1		11.49242	13	33	7	145.148	6.9	5900000	6400000	5700000	82.27118
Q8K4P0	pre-mRNA 3' end processing protein WDR33 OS=Mus musculus GN=Wdr33 PE=1 SV=1		2.556391	3	5	3	145.176	9.13	780000	1100000	1600000	12.34229
Q6PGL7	WASH complex subunit FAM21 OS=Mus musculus GN=Fam21 PE=1 SV=1		9.595202	8	15	8	145.224	4.77	2000000	1900000	1300000	38.89133
Q5SQX6	Cytoplasmic FMR1-interacting protein 2 OS=Mus musculus GN=Cyfip2 PE=1 SV=2		10.45491	12	22	6	145.565	7.05	3000000	2300000	1400000	58.94764
Q99NB9	Splicing factor 3B subunit 1 OS=Mus musculus GN=Sf3b1 PE=1 SV=1		21.39571	20	48	20	145.724	7.09	9100000	9200000	9300000	152.6658
Q58A65	C-Jun-amino-terminal kinase-interacting protein 4 OS=Mus musculus GN=Spag9 PE=1 SV=2		4.163512	4	9	4	146.129	5.15	1800000	1800000	1200000	24.19418
Q64331	Unconventional myosin-VI OS=Mus musculus GN=Myo6 PE=1 SV=1		6.166008	5	6	5	146.317	8.85	1000000	1700000	710000	19.31795
Q00519	Xanthine dehydrogenase/oxidase OS=Mus musculus GN=Xdh PE=1 SV=5		2.022472	2	4	2	146.468	7.56	1700000	2300000		13.75304
Q9QY30	Bile salt export pump OS=Mus musculus GN=Abcb11 PE=1 SV=2		1.286904	1	1	1	146.655	7.66		480000		2.938556
Q8CG47	Structural maintenance of chromosomes protein 4 OS=Mus musculus GN=Smc4 PE=1 SV=1		5.909798	6	11	6	146.803	7.3	1300000	2800000	1200000	31.07413
Q9JM52	Misshapen-like kinase 1 OS=Mus musculus GN=Mink1 PE=1 SV=3		2.752294	3	7	1	147.203	7.44			2100000	18.29843
Q8CIH5	1-phosphatidylinositol 4,5-bisphosphate phosphodiesterase gamma-2 OS=Mus musculus GN=Plcg2 PE=1 SV=1		2.687747	2	3	2	147.498	6.77	1500000		1100000	7.85052
Q61526	Receptor tyrosine-protein kinase erbB-3 OS=Mus musculus GN=Erbb3 PE=1 SV=2		0.746826	1	3	1	147.517	6.55	1300000	1300000	1100000	7.470978
Q6PCM1	Lysine-specific demethylase 3A OS=Mus musculus GN=Kdm3a PE=1 SV=1		1.284958	1	1	1	147.754	7.74				2.777489
Q5SW19	Clustered mitochondria protein homolog OS=Mus musculus GN=Cluh PE=1 SV=2		2.965779	2	3	2	147.975	6.02	940000	600000		10.46708
F6SEU4	Ras/Rap GTPase-activating protein SynGAP OS=Mus musculus GN=Syngap1 PE=1 SV=2		2.089552	2	3	2	148.145	8.98	1700000		700000	7.912853
Q6Y7W8	PERQ amino acid-rich with GYF domain-containing protein 2 OS=Mus musculus GN=Gigyf2 PE=1 SV=2		3.408211	3	3	3	149.102	5.57	980000	420000		7.451162
Q8K4Q0	Regulatory-associated protein of mTOR OS=Mus musculus GN=Rptor PE=1 SV=1		1.198502	1	1	1	149.375	6.87	870000			2.912625
O70133	ATP-dependent RNA helicase A OS=Mus musculus GN=Dhx9 PE=1 SV=2		17.68116	19	60	19	149.381	6.83	21000000	23000000	23000000	201.6523
Q7TT37	Elongator complex protein 1 OS=Mus musculus GN=Ikbkap PE=1 SV=2		5.326332	6	11	6	149.489	6	2200000	2200000	2200000	32.07987
Q8BYI9	Tenascin-R OS=Mus musculus GN=Tnr PE=1 SV=2		7.142857	8	12	8	149.495	4.94	4800000	3300000	2800000	34.4969
Q6PDH0	Pleckstrin homology-like domain family B member 1 OS=Mus musculus GN=Phldb1 PE=1 SV=1		2.552881	3	6	3	149.978	8.87	2800000	3500000	3000000	18.3882
Q6A026	Sister chromatid cohesion protein PDS5 homolog A OS=Mus musculus GN=Pds5a PE=1 SV=3		8.333333	10	20	9	150.232	7.85	3200000	3300000	3000000	66.14987
P83510	Traf2 and NCK-interacting protein kinase OS=Mus musculus GN=Tnik PE=1 SV=2		2.87226	3	7	1	150.274	7.27		210000		18.08763
Q6P9K8	Caskin-1 OS=Mus musculus GN=Caskin1 PE=1 SV=2		1.18798	1	1	1	150.403	9.17	480000			3.474536
Q3UJD6	Ubiquitin carboxyl-terminal hydrolase 19 OS=Mus musculus GN=Usp19 PE=1 SV=1		3.970588	4	6	4	150.454	6.38	780000	640000	400000	16.63781
Q9JIX8	Apoptotic chromatin condensation inducer in the nucleus OS=Mus musculus GN=Acin1 PE=1 SV=3		16.44245	15	44	15	150.629	5.91	9600000	9500000	9000000	144.677
P54276	DNA mismatch repair protein Msh6 OS=Mus musculus GN=Msh6 PE=1 SV=3		5.228277	5	10	5	150.989	6.73	1500000	2500000	1500000	33.62239
Q6EDY6	F-actin-uncapping protein LRRC16A OS=Mus musculus GN=Lrrc16a PE=1 SV=2		0.800582	1	1	1	151.765	7.8	1000000			2.288437
Q7TPV4	Myb-binding protein 1A OS=Mus musculus GN=Mybbp1a PE=1 SV=2		19.41964	20	69	20	151.942	8.95	14000000	21000000	16000000	231.2749
Q3UJB9	Enhancer of mRNA-decapping protein 4 OS=Mus musculus GN=Edc4 PE=1 SV=2		2.418208	3	3	3	152.389	5.78			1400000	7.244508
Q8BMG7	Rab3 GTPase-activating protein non-catalytic subunit OS=Mus musculus GN=Rab3gap2 PE=1 SV=2		2.781845	4	6	4	152.438	6.23	2000000	990000	1800000	10.89688
Q61595	Kinectin OS=Mus musculus GN=Ktn1 PE=1 SV=1		14.92087	18	36	17	152.498	5.86	4500000	4700000	5000000	105.8367
Q8VD65	Phosphoinositide 3-kinase regulatory subunit 4 OS=Mus musculus GN=Pik3r4 PE=1 SV=3		2.061856	2	4	2	152.502	7.12	370000	1100000	750000	11.7176
Q9JLM4	Zinc finger MYM-type protein 3 OS=Mus musculus GN=Zmym3 PE=1 SV=1		1.094891	1	1	1	152.78	6.39			1000000	3.193702
Q80TJ1	Calcium-dependent secretion activator 1 OS=Mus musculus GN=Cadps PE=1 SV=3		4.280443	5	8	5	153.016	5.74	3500000	1700000	1300000	22.95076
P70388	DNA repair protein RAD50 OS=Mus musculus GN=Rad50 PE=1 SV=1		3.810976	5	11	5	153.392	6.95	1600000	2200000	1900000	26.47304
Q689Z5	Protein strawberry notch homolog 1 OS=Mus musculus GN=Sbno1 PE=1 SV=2		0.935252	1	3	1	153.642	8.07		870000		7.495418
Q5FWI3	Transmembrane protein 2 OS=Mus musculus GN=Tmem2 PE=1 SV=1		1.156905	1	1	1	153.704	7.78			320000	2.680209
O88322	Nidogen-2 OS=Mus musculus GN=Nid2 PE=1 SV=2		5.274412	5	11	4	153.816	5.38	2300000	2100000	2800000	31.92629
Q6PGC1	ATP-dependent RNA helicase Dhx29 OS=Mus musculus GN=Dhx29 PE=1 SV=1		4.175824	4	5	4	153.879	7.94	910000	1100000	920000	13.98927
O35099	Mitogen-activated protein kinase kinase kinase 5 OS=Mus musculus GN=Map3k5 PE=1 SV=3		0.507246	1	4	1	154.414	5.78	18000000	17000000	2400000	8.44165
Q9CU65	Zinc finger MYM-type protein 2 OS=Mus musculus GN=Zmym2 PE=1 SV=3		2.107558	2	6	2	154.541	6.37	760000	740000	510000	18.08458
Q8BZH4	Pogo transposable element with ZNF domain OS=Mus musculus GN=Pogz PE=1 SV=2		5.110007	5	11	5	154.812	7.52	790000	1100000	1000000	36.14917
Q99P88	Nuclear pore complex protein Nup155 OS=Mus musculus GN=Nup155 PE=1 SV=1		5.176132	6	15	6	155.019	6.15	4000000	4000000	4000000	39.54627
Q9R0I7	YLP motif-containing protein 1 OS=Mus musculus GN=Ylpm1 PE=2 SV=2		1.731602	2	3	2	155.032	6.65	2500000	1500000		6.734203
Q5SSZ5	Tensin-3 OS=Mus musculus GN=Tns3 PE=1 SV=1		2.222222	2	4	2	155.491	6.65	1900000	1300000	1500000	13.30232
P15208	Insulin receptor OS=Mus musculus GN=Insr PE=1 SV=2		2.113703	2	3	2	155.51	5.95	690000	650000	320000	7.947726
Q8K2Z4	Condensin complex subunit 1 OS=Mus musculus GN=Ncapd2 PE=1 SV=2		5.316092	4	8	4	155.567	6.38	1800000	3000000	2000000	25.19335
Q8BLX7	Collagen alpha-1(XVI) chain OS=Mus musculus GN=Col16a1 PE=1 SV=2		2.911392	3	8	3	155.707	7.91	2200000	1500000	610000	19.51003
Q922J3	CAP-Gly domain-containing linker protein 1 OS=Mus musculus GN=Clip1 PE=1 SV=1		7.620417	8	15	6	155.718	5.24	1700000	1600000	1300000	44.47577

Q9ESU6	Bromodomain-containing protein 4 OS=Mus musculus GN=Brd4 PE=1 SV=2		2.571429	2	4	2	155.798	9.19	2400000	1700000	2400000	10.38856
Q91ZV0	Melanoma inhibitory activity protein 2 OS=Mus musculus GN=Mia2 PE=1 SV=3		5.659026	6	10	6	156.365	4.55	1100000	2000000	820000	29.48897
Q6PB66	Leucine-rich PPR motif-containing protein, mitochondrial OS=Mus musculus GN=Lrpprc PE=1 SV=2		7.255747	9	17	9	156.516	6.83	1400000	2200000	1100000	47.70379
Q8BY87	Ubiquitin carboxyl-terminal hydrolase 47 OS=Mus musculus GN=Usp47 PE=1 SV=2		5.813953	5	6	5	157.356	5.11	510000	490000	370000	17.93519
P70335	Rho-associated protein kinase 1 OS=Mus musculus GN=Rock1 PE=1 SV=1		5.982275	7	12	5	158.072	5.86	1600000	1800000	1500000	30.06621
Q9Z0W3	Nuclear pore complex protein Nup160 OS=Mus musculus GN=Nup160 PE=1 SV=2		2.282454	3	6	3	158.13	5.52	1100000	1600000	2000000	13.61713
Q6P9L6	Kinesin-like protein KIF15 OS=Mus musculus GN=Kif15 PE=1 SV=1		2.451334	2	3	2	160.021	6.05	280000	310000		8.429106
P61406	Telomerase-binding protein EST1A OS=Mus musculus GN=Smg6 PE=1 SV=1		0.634697	1	1	1	160.396	6.77				2.324064
P70336	Rho-associated protein kinase 2 OS=Mus musculus GN=Rock2 PE=1 SV=1		12.89625	16	36	14	160.485	5.99	7400000	5700000	5000000	93.1948
P02463	Collagen alpha-1(IV) chain OS=Mus musculus GN=Col4a1 PE=1 SV=4		2.995806	4	18	4	160.579	8.24	5500000	5600000	4900000	54.05297
Q8CGB3	Uveal autoantigen with coiled-coil domains and ankyrin repeats OS=Mus musculus GN=Uaca PE=1 SV=2		4.394047	5	8	5	160.714	7.2	1400000	590000	1000000	24.07602
Q9EPU4	Cleavage and polyadenylation specificity factor subunit 1 OS=Mus musculus GN=Cpsf1 PE=1 SV=1		2.012491	2	7	2	160.716	6.39	1500000	1700000	1600000	21.6396
Q7TPM1	Protein PRRC2B OS=Mus musculus GN=Prrc2b PE=1 SV=1		2.557201	2	5	2	160.814	8.16	480000	450000	390000	17.15943
Q8BL66	Early endosome antigen 1 OS=Mus musculus GN=Eea1 PE=1 SV=2		8.433735	10	19	10	160.817	5.77	2200000	2100000	1500000	49.40381
Q8R422	CD109 antigen OS=Mus musculus GN=Cd109 PE=1 SV=1		4.230236	5	10	5	161.557	5.57	3300000	1900000	2800000	27.27477
Q5DU25	IQ motif and SEC7 domain-containing protein 2 OS=Mus musculus GN=Iqsec2 PE=1 SV=3		2.232747	3	3	1	161.687	8.56	440000			6.455737
P23116	Eukaryotic translation initiation factor 3 subunit A OS=Mus musculus GN=Eif3a PE=1 SV=5		17.63393	26	71	26	161.838	6.77	13000000	12000000	13000000	192.9029
Q6RHR9	Membrane-associated guanylate kinase, WW and PDZ domain-containing protein 1 OS=Mus musculus GN=Magi1 PE=1 SV=1		0.951734	1	2	1	161.876	7.36		550000	550000	4.793185
Q6PAR5	GTPase-activating protein and VPS9 domain-containing protein 1 OS=Mus musculus GN=Gapvd1 PE=1 SV=2		0.754458	1	1	1	162.3	5.19		920000		2.423391
Q1W617	Protein Shroom4 OS=Mus musculus GN=Shroom4 PE=1 SV=1		1.288136	1	1	1	163.137	6.73		150000		2.575413
Q8C1B1	Calmodulin-regulated spectrin-associated protein 2 OS=Mus musculus GN=Camsap2 PE=1 SV=3		1.779603	2	2	2	164.23	6.86		900000		4.651211
Q4VA53	Sister chromatid cohesion protein PDS5 homolog B OS=Mus musculus GN=Pds5b PE=1 SV=1		12.30982	14	27	13	164.315	8.5	4000000	3200000	4300000	71.83356
Q69ZA1	Cyclin-dependent kinase 13 OS=Mus musculus GN=Cdk13 PE=1 SV=3		2.845797	4	8	3	164.452	9.69	630000	510000		20.0365
A2A6A1	G patch domain-containing protein 8 OS=Mus musculus GN=Gpatch8 PE=1 SV=1		1.993355	2	2	2	164.888	7.64		1100000	810000	5.951295
Q3UQ28	Peroxidasin homolog OS=Mus musculus GN=Pxdn PE=1 SV=2		0.881356	1	1	1	164.998	7.14	270000			2.257228
P28665	Murinoglobulin-1 OS=Mus musculus GN=Mug1 PE=1 SV=3		4.810298	6	14	6	165.193	6.42	2000000	2000000	1500000	35.33922
Q61838	Pregnancy zone protein OS=Mus musculus GN=Pzp PE=1 SV=3		5.618729	8	17	8	165.748	6.68	5600000	7000000	5400000	43.24908
Q01097	Glutamate receptor ionotropic, NMDA 2B OS=Mus musculus GN=Grin2b PE=1 SV=3		0.674764	1	1	1	165.853	6.87	410000			1.957385
Q8BX17	Gem-associated protein 5 OS=Mus musculus GN=Gemin5 PE=1 SV=2		5.05992	5	12	5	166.487	6.71	1800000	1800000	1800000	37.42174
Q64449	C-type mannose receptor 2 OS=Mus musculus GN=Mrc2 PE=1 SV=3		0.878972	1	2	1	166.968	5.99				5.544687
Q925J9	Mediator of RNA polymerase II transcription subunit 1 OS=Mus musculus GN=Med1 PE=1 SV=2		1.84127	2	3	2	167.036	8.73		740000	960000	7.552844
P55937	Golgin subfamily A member 3 OS=Mus musculus GN=Golga3 PE=1 SV=3		8.944183	10	24	10	167.118	5.4	2300000	2200000	2100000	75.42687
P08122	Collagen alpha-2(IV) chain OS=Mus musculus GN=Col4a2 PE=1 SV=4		3.04628	4	10	4	167.22	8.48	4800000	12000000	5600000	25.19925
P33609	DNA polymerase alpha catalytic subunit OS=Mus musculus GN=Pola1 PE=1 SV=2		3.208191	3	4	3	167.233	5.74		830000	710000	12.16018
Q5BLK4	Terminal uridylyltransferase 7 OS=Mus musculus GN=Zcchc6 PE=1 SV=3		4.091214	3	5	3	168.996	6.65	350000	1000000	680000	13.65187
Q80TV8	CLIP-associating protein 1 OS=Mus musculus GN=Clasp1 PE=1 SV=2		3.843648	4	7	4	169.123	9.03	860000	1100000	2100000	21.14189
Q8CAF4	NHS-like protein 1 OS=Mus musculus GN=Nhsl1 PE=1 SV=3		2.457467	2	2	2	169.315	7.49			230000	6.786838
Q8CGC7	Bifunctional glutamate/proline--tRNA ligase OS=Mus musculus GN=Eprs PE=1 SV=4		23.01587	27	65	27	169.972	7.66	13000000	13000000	11000000	192.3106
Q8CFQ3	Intron-binding protein aquarius OS=Mus musculus GN=Aqr PE=1 SV=2		2.228224	3	4	3	170.185	6.27		830000	1100000	9.491365
Q91YM2	Rho GTPase-activating protein 35 OS=Mus musculus GN=Arhgap35 PE=1 SV=3		1.334223	2	4	2	170.285	6.61	1100000	780000	1300000	10.43315
Q9Z277	Tyrosine-protein kinase BAZ1B OS=Mus musculus GN=Baz1b PE=1 SV=2		9.398242	11	28	11	170.544	8.6	5100000	3500000	3900000	81.09051
Q3UH60	Disco-interacting protein 2 homolog B OS=Mus musculus GN=Dip2b PE=1 SV=1		2.92249	3	9	3	171.017	8.09	2100000	1600000	1400000	24.56342
O35379	Multidrug resistance-associated protein 1 OS=Mus musculus GN=Abcc1 PE=1 SV=1		1.374346	1	2	1	171.075	7.36			310000	6.547547
Q64739	Collagen alpha-2(XI) chain OS=Mus musculus GN=Col11a2 PE=2 SV=3		1.382488	2	6	2	171.431	6.48	2600000	2600000	2100000	16.54259
Q8R4H2	Rho guanine nucleotide exchange factor 12 OS=Mus musculus GN=Arhgef12 PE=1 SV=2		0.842515	1	3	1	172.242	5.74		1000000	120000	6.526386
Q8CHC4	Synaptojanin-1 OS=Mus musculus GN=Synj1 PE=1 SV=3		4.510801	5	10	5	172.509	6.89	3900000	2700000	1500000	33.39039
Q01320	DNA topoisomerase 2-alpha OS=Mus musculus GN=Top2a PE=1 SV=2		23.29843	31	76	21	172.682	8.6	11000000	15000000	15000000	222.5312
Q99PL5	Ribosome-binding protein 1 OS=Mus musculus GN=Rrbp1 PE=1 SV=2		25.42056	35	115	35	172.776	9.33	27000000	32000000	31000000	374.5463
Q80U72	Protein scribble homolog OS=Mus musculus GN=Scrib PE=1 SV=2		1.861042	2	3	2	173.952	5.12	720000		750000	7.605809
Q80XI3	Eukaryotic translation initiation factor 4 gamma 3 OS=Mus musculus GN=Eif4g3 PE=1 SV=2		9.183027	11	25	10	174.781	5.53	1400000	1700000	1400000	68.28318
Q80TM9	Nischarin OS=Mus musculus GN=Nisch PE=1 SV=2		1.506591	1	1	1	174.903	5.16			300000	2.367632
Q6A065	Centrosomal protein of 170 kDa OS=Mus musculus GN=Cep170 PE=1 SV=2		3.085642	4	9	4	174.943	7.17	1600000	1400000	600000	24.62544
Q3UHC0	Trinucleotide repeat-containing gene 6C protein OS=Mus musculus GN=Tnrc6c PE=1 SV=2		1.538462	1	1	1	175.667	6.86		50000		3.489697

Q6NZJ6	Eukaryotic translation initiation factor 4 gamma 1 OS=Mus musculus GN=Eif4g1 PE=1 SV=1		15.9375	20	50	19	175.967	5.4	9600000	10000000	9800000	146.5586
Q6P5E4	UDP-glucose:glycoprotein glucosyltransferase 1 OS=Mus musculus GN=Uggt1 PE=1 SV=4		17.60155	21	49	21	176.323	5.62	13000000	11000000	9700000	140.8715
P02468	Laminin subunit gamma-1 OS=Mus musculus GN=Lamc1 PE=1 SV=2		19.47729	25	63	25	177.185	5.21	7500000	9000000	8700000	190.0672
O88379	Bromodomain adjacent to zinc finger domain protein 1A OS=Mus musculus GN=Baz1a PE=1 SV=3		1.093248	2	3	2	178.349	6.54	880000	360000	930000	6.142228
Q8K4G1	Latent-transforming growth factor beta-binding protein 4 OS=Mus musculus GN=Ltbp4 PE=1 SV=2		3.961585	4	5	4	178.523	5.47	1100000	720000	460000	15.20464
Q3URU2	Paternally-expressed gene 3 protein OS=Mus musculus GN=Peg3 PE=1 SV=1		0.954806	1	2	1	178.822	5.45	460000	700000		5.134691
Q69Z37	Sterile alpha motif domain-containing protein 9-like OS=Mus musculus GN=Samd9l PE=1 SV=2		2.626521	3	7	3	180.089	6.99	1500000	1200000	1300000	21.62676
Q3UQ44	Ras GTPase-activating-like protein IQGAP2 OS=Mus musculus GN=Iqgap2 PE=1 SV=2		6.222222	8	16	6	180.416	5.64	870000	820000	1100000	40.63136
Q9WTQ5	A-kinase anchor protein 12 OS=Mus musculus GN=Akap12 PE=1 SV=1		18.17102	19	45	19	180.586	4.44	4100000	3000000	2700000	143.5999
Q61245	Collagen alpha-1(XI) chain OS=Mus musculus GN=Col11a1 PE=1 SV=2		4.711752	6	13	6	180.921	5.21	6900000	11000000	8600000	30.95573
Q3TKT4	Transcription activator BRG1 OS=Mus musculus GN=Smarca4 PE=1 SV=1		8.431494	11	28	11	181.313	8	4200000	4300000	4800000	74.26286
P58871	182 kDa tankyrase-1-binding protein OS=Mus musculus GN=Tnks1bp1 PE=1 SV=2		5.465116	6	12	6	181.714	4.88	1300000	590000	980000	32.57068
Q64511	DNA topoisomerase 2-beta OS=Mus musculus GN=Top2b PE=1 SV=2		17.12159	24	69	14	181.795	8.29	4200000	4600000	4400000	196.7001
P39061	Collagen alpha-1(XVIII) chain OS=Mus musculus GN=Col18a1 PE=1 SV=4		8.455468	11	26	11	182.059	5.62	14000000	10000000	14000000	77.9938
B1AZI6	THO complex subunit 2 OS=Mus musculus GN=Thoc2 PE=1 SV=1		3.199498	5	8	5	182.658	8.44	1000000	470000	1400000	14.23803
Q62230	Sialoadhesin OS=Mus musculus GN=Siglec1 PE=1 SV=2		0.766962	1	1	1	182.865	6.67				1.94105
P13864	DNA (cytosine-5)-methyltransferase 1 OS=Mus musculus GN=Dnmt1 PE=1 SV=5		16.23457	19	43	19	183.074	7.74	4600000	5200000	5200000	134.4936
O88207	Collagen alpha-1(V) chain OS=Mus musculus GN=Col5a1 PE=1 SV=2		0.707291	1	3	1	183.564	4.98	7300000	7700000	7100000	11.57436
A6H619	PHD and RING finger domain-containing protein 1 OS=Mus musculus GN=Phrf1 PE=1 SV=2		1.36742	1	1	1	183.968	8.59				2.323309
B2RX14	Terminal uridylyltransferase 4 OS=Mus musculus GN=Zcchc11 PE=1 SV=2		0.547445	1	1	1	184.532	8.19			550000	2.168504
Q5PSV9	Mediator of DNA damage checkpoint protein 1 OS=Mus musculus GN=Mdc1 PE=1 SV=1		10.77914	11	19	11	184.558	5.19	2400000	2100000	1900000	58.25554
Q69ZK0	Phosphatidylinositol 3,4,5-trisphosphate-dependent Rac exchanger 1 protein OS=Mus musculus GN=Prex1 PE=1 SV=2		2.909091	2	4	2	184.818	6.29	710000	1000000		10.80074
Q6PB44	Tyrosine-protein phosphatase non-receptor type 23 OS=Mus musculus GN=Ptpn23 PE=1 SV=2		1.06383	1	2	1	185.1	6.8	690000	480000		5.678161
Q99MV7	RING finger protein 17 OS=Mus musculus GN=Rnf17 PE=1 SV=2		0.487805	1	1	1	185.48	5.48	980000			2.609912
Q8C0T9	Adenylate cyclase type 10 OS=Mus musculus GN=Adcy10 PE=2 SV=2		1.115242	1	1	1	186.29	6.9	410000			2.268139
P01027	Complement C3 OS=Mus musculus GN=C3 PE=1 SV=3		9.260373	11	28	11	186.366	6.73	3500000	4200000	3000000	83.06149
Q9QXL2	Kinesin-like protein KIF21A OS=Mus musculus GN=Kif21a PE=1 SV=2		4.784689	5	11	5	186.42	6.27	1800000	1300000	750000	33.91546
Q8BSQ9	Protein polybromo-1 OS=Mus musculus GN=Pbrm1 PE=1 SV=4		1.529988	2	3	2	187.069	6.9	1500000	1800000	2000000	7.442332
Q9JKF1	Ras GTPase-activating-like protein IQGAP1 OS=Mus musculus GN=Iqgap1 PE=1 SV=2		31.80447	43	126	41	188.624	6.48	21000000	19000000	16000000	399.4422
P06684	Complement C5 OS=Mus musculus GN=C5 PE=1 SV=2		2.440476	3	4	3	188.759	6.81	460000		800000	10.0602
Q9Z0R6	Intersectin-2 OS=Mus musculus GN=Itsn2 PE=1 SV=2		1.3261	2	4	2	188.789	8.13	1200000	790000	770000	10.3986
Q61194	Phosphatidylinositol 4-phosphate 3-kinase C2 domain-containing subunit alpha OS=Mus musculus GN=Pik3c2a PE=1 SV=2		1.542112	2	2	2	190.637	8.02		840000		4.708306
Q68FD5	Clathrin heavy chain 1 OS=Mus musculus GN=Cltc PE=1 SV=3		32.29851	44	140	44	191.435	5.69	75000000	64000000	59000000	433.9667
Q8BKI2	Trinucleotide repeat-containing gene 6B protein OS=Mus musculus GN=Tnrc6b PE=1 SV=2		0.939227	1	1	1	191.845	6.3				2.303348
Q8BJ34	Meiosis arrest female protein 1 OS=Mus musculus GN=Marf1 PE=1 SV=3		0.404624	1	3	1	191.942	8.22	1100000		930000	6.133669
Q3UXZ9	Lysine-specific demethylase 5A OS=Mus musculus GN=Kdm5a PE=1 SV=2		0.946746	1	1	1	192.093	6.39			250000	2.293927
P01029	Complement C4-B OS=Mus musculus GN=C4b PE=1 SV=3		9.95397	14	35	14	192.794	7.53	6300000	6800000	6600000	97.00583
O35134	DNA-directed RNA polymerase I subunit RPA1 OS=Mus musculus GN=Polr1a PE=1 SV=2		3.610949	3	5	3	193.987	6.93	2700000	1100000		16.5927
Q9Z0R4	Intersectin-1 OS=Mus musculus GN=Itsn1 PE=1 SV=2		1.575263	2	5	2	194.176	7.91	1600000	990000	760000	14.94564
P97789	5'-3' exoribonuclease 1 OS=Mus musculus GN=Xrn1 PE=1 SV=1		2.094241	2	2	2	194.185	7.5	240000		1100000	5.5123
Q8CHG3	GRIP and coiled-coil domain-containing protein 2 OS=Mus musculus GN=Gcc2 PE=1 SV=2		2.561048	3	6	3	194.325	5.12	610000	860000	350000	16.40942
P39447	Tight junction protein ZO-1 OS=Mus musculus GN=Tjp1 PE=1 SV=2		13.46705	18	39	18	194.622	6.64	8400000	6700000	7600000	121.2865
Q7TT50	Serine/threonine-protein kinase MRCK beta OS=Mus musculus GN=Cdc42bpb PE=1 SV=2		3.03561	4	8	4	194.63	6.46	1500000	1700000	1400000	25.65304
P40201	Chromodomain-helicase-DNA-binding protein 1 OS=Mus musculus GN=Chd1 PE=1 SV=3		5.084746	5	9	5	196.264	7.37	970000	600000	1100000	25.52011
Q61292	Laminin subunit beta-2 OS=Mus musculus GN=Lamb2 PE=1 SV=2		10.28349	14	27	13	196.451	6.67	2600000	3600000	3700000	68.68743
Q8C0T5	Signal-induced proliferation-associated 1-like protein 1 OS=Mus musculus GN=Sipa1l1 PE=1 SV=2		1.851852	2	3	2	196.909	8.13	500000	280000		9.399401
P02469	Laminin subunit beta-1 OS=Mus musculus GN=Lamb1 PE=1 SV=3		11.42217	14	31	13	196.961	4.94	5300000	7000000	6300000	90.95953
Q6PFD9	Nuclear pore complex protein Nup98-Nup96 OS=Mus musculus GN=Nup98 PE=1 SV=2		3.634361	5	9	5	197.118	6.18	1600000	2600000	1800000	25.93634
Q60767	Lymphocyte antigen 75 OS=Mus musculus GN=Ly75 PE=1 SV=2		0.870575	1	1	1	197.225	6.39	710000			2.618113
Q6A009	E3 ubiquitin-protein ligase listerin OS=Mus musculus GN=Ltn1 PE=1 SV=3		1.6412	2	4	2	198.793	6.55	1800000	1400000	510000	14.06261
Q62383	Transcription elongation factor SPT6 OS=Mus musculus GN=Supt6h PE=1 SV=2		5.735805	6	13	6	198.962	4.93	830000	830000	1100000	36.09366
P20357	Microtubule-associated protein 2 OS=Mus musculus GN=Map2 PE=1 SV=2		13.45733	18	34	18	199.01	4.91	7900000	4500000	2700000	99.24174
P97868	E3 ubiquitin-protein ligase RBBP6 OS=Mus musculus GN=Rbbp6 PE=1 SV=5		3.072626	4	9	4	199.466	9.63	1000000	1100000	1100000	25.40926

Q0VGY8	Protein TANC1 OS=Mus musculus GN=Tanc1 PE=1 SV=2		0.700431	1	1	1	200.679	8.35		590000		2.629297
A2AIV2	Protein virilizer homolog OS=Mus musculus GN=Kiaa1429 PE=1 SV=1		0.717835	1	2	1	201.313	4.96		880000	800000	5.867517
P97927	Laminin subunit alpha-4 OS=Mus musculus GN=Lama4 PE=1 SV=2		9.251101	11	19	11	201.692	6.21	2300000	3700000	3300000	50.65098
A2A5R2	Brefeldin A-inhibited guanine nucleotide-exchange protein 2 OS=Mus musculus GN=Arfgef2 PE=1 SV=1		2.287946	3	3	3	202.11	6.55	560000	550000	720000	8.597274
Q6PDI5	Proteasome-associated protein ECM29 homolog OS=Mus musculus GN=Ecm29 PE=1 SV=3		3.913043	5	8	5	203.573	7.06	950000	1000000	750000	23.97787
E9Q784	Zinc finger CCCH domain-containing protein 13 OS=Mus musculus GN=Zc3h13 PE=1 SV=1		0.75188	1	2	1	203.635	9.38		1200000	1000000	4.850768
Q2EMV9	Poly [ADP-ribose] polymerase 14 OS=Mus musculus GN=Parp14 PE=1 SV=3		5.72372	7	14	7	203.674	6.79	1500000	1700000	1800000	36.27764
Q9QY81	Nuclear pore membrane glycoprotein 210 OS=Mus musculus GN=Nup210 PE=1 SV=2		2.704136	3	6	3	203.973	6.65	2400000	1400000	1300000	16.50189
B2RXS4	Plexin-B2 OS=Mus musculus GN=Plxnb2 PE=1 SV=1		3.474484	5	7	5	206.099	5.87	710000	670000	1200000	18.83012
Q9QZQ1	Afadin OS=Mus musculus GN=Mllt4 PE=1 SV=3		3.791209	5	11	5	206.371	6.32	1700000	1200000	750000	32.47821
Q8VDD9	PH-interacting protein OS=Mus musculus GN=Phip PE=1 SV=2		1.812191	2	6	2	206.596	8.82	1600000	1400000	1900000	21.22524
Q6P5H2	Nestin OS=Mus musculus GN=Nes PE=1 SV=1		35.78326	43	121	43	207	4.34	15000000	15000000	18000000	392.583
A2ASQ1	Agrin OS=Mus musculus GN=Agrn PE=1 SV=1		4.153846	6	13	6	207.403	6.32	1300000	1700000	1500000	40.48429
Q6NS46	Protein RRP5 homolog OS=Mus musculus GN=Pdcd11 PE=1 SV=2		4.296455	5	8	5	207.649	8.75	2300000	820000	2500000	23.97645
G3X9K3	Brefeldin A-inhibited guanine nucleotide-exchange protein 1 OS=Mus musculus GN=Arfgef1 PE=1 SV=1		3.141928	4	5	4	208.368	5.86	290000	460000	520000	13.21744
Q6ZPE2	Myotubularin-related protein 5 OS=Mus musculus GN=Sbf1 PE=1 SV=2		2.731655	4	5	4	208.561	7.12	730000	510000	390000	10.49334
Q91YE5	Bromodomain adjacent to zinc finger domain protein 2A OS=Mus musculus GN=Baz2a PE=1 SV=2		0.847009	1	1	1	209.487	6.51				2.984473
Q61191	Host cell factor 1 OS=Mus musculus GN=Hcfc1 PE=1 SV=2		2.738386	5	9	5	210.306	7.18	3100000	2700000	2200000	22.48282
P21271	Unconventional myosin-Vb OS=Mus musculus GN=Myo5b PE=1 SV=2		19.14191	28	59	23	210.438	7.11	4700000	5300000	4900000	172.0973
P70399	Tumor suppressor p53-binding protein 1 OS=Mus musculus GN=Tp53bp1 PE=1 SV=2		7.307103	9	19	9	211.209	4.63	1800000	2300000	2000000	61.87191
Q8C3J5	Dedicator of cytokinesis protein 2 OS=Mus musculus GN=Dock2 PE=1 SV=3		4.266958	7	15	7	211.568	6.99	2900000	3300000	2800000	41.07513
Q6PDN3	Myosin light chain kinase, smooth muscle OS=Mus musculus GN=Mylk PE=1 SV=3		0.515198	1	1	1	212.792	6.25		840000		2.710953
Q80U93	Nuclear pore complex protein Nup214 OS=Mus musculus GN=Nup214 PE=1 SV=2		0.719424	1	3	1	212.847	7.08	1400000	1700000	1600000	8.506969
Q8CC88	von Willebrand factor A domain-containing protein 8 OS=Mus musculus GN=Vwa8 PE=1 SV=2		0.682415	1	1	1	213.287	6.6	920000			2.691186
Q8BI84	Melanoma inhibitory activity protein 3 OS=Mus musculus GN=Mia3 PE=1 SV=2		11.86528	15	31	15	213.544	4.75	3100000	2800000	2900000	90.09088
Q64487	Receptor-type tyrosine-protein phosphatase delta OS=Mus musculus GN=Ptprd PE=1 SV=3		2.144351	3	3	3	214.274	6.55	530000	420000		6.44431
Q8CFT2	Histone-lysine N-methyltransferase SETD1B OS=Mus musculus GN=Setd1b PE=1 SV=2		0.403023	1	2	1	215.217	4.96	3000000	4200000		3.97045
Q99104	Unconventional myosin-Va OS=Mus musculus GN=Myo5a PE=1 SV=2		10.41554	18	36	13	215.402	8.63	5500000	4400000	3000000	96.57145
Q5SNZ0	Girdin OS=Mus musculus GN=Ccdc88a PE=1 SV=2		2.829685	4	8	4	215.786	6.24	1300000	730000	450000	21.62734
P53995	Anaphase-promoting complex subunit 1 OS=Mus musculus GN=Anapc1 PE=1 SV=2		1.028807	1	1	1	215.855	6.35	590000			2.491033
P08775	DNA-directed RNA polymerase II subunit RPB1 OS=Mus musculus GN=Polr2a PE=1 SV=3		7.15736	10	19	10	217.039	7.37	2500000	1900000	1800000	51.50875
A8C756	Thyroid adenoma-associated protein homolog OS=Mus musculus GN=Thada PE=1 SV=1		0.567595	1	1	1	217.15	6.25		210000		1.850821
Q6PDQ2	Chromodomain-helicase-DNA-binding protein 4 OS=Mus musculus GN=Chd4 PE=1 SV=1		19.06005	27	79	27	217.614	5.81	12000000	13000000	13000000	230.3806
Q61464	Zinc finger protein 638 OS=Mus musculus GN=Znf638 PE=1 SV=2		6.020408	8	18	8	217.999	6.89	2500000	2700000	2400000	57.288
Q8R151	NFX1-type zinc finger-containing protein 1 OS=Mus musculus GN=Znfx1 PE=1 SV=3		2.985856	4	7	4	218.687	7.47	1200000	1400000	990000	21.46255
P70670	Nascent polypeptide-associated complex subunit alpha, muscle-specific form OS=Mus musculus GN=Naca PE=1 SV=2		1.920439	3	16	3	220.364	9.35	19000000	17000000	17000000	58.02378
Q61282	Aggrecan core protein OS=Mus musculus GN=Acan PE=1 SV=2		2.06379	4	9	4	221.805	4.3	2600000	1300000	2100000	23.52817
Q99KW3	TRIO and F-actin-binding protein OS=Mus musculus GN=Triobp PE=1 SV=3		1.390268	2	2	2	223.232	8.06	500000		750000	6.198935
Q8CJ19	Protein-methionine sulfoxide oxidase MICAL3 OS=Mus musculus GN=Mical3 PE=1 SV=2		0.652283	1	1	1	223.583	5.47				2.690964
G5E870	E3 ubiquitin-protein ligase TRIP12 OS=Mus musculus GN=Trip12 PE=1 SV=1		8.740741	13	23	13	223.988	8.35	4300000	3500000	3800000	73.96777
A2AGT5	Cytoskeleton-associated protein 5 OS=Mus musculus GN=Ckap5 PE=1 SV=1		7.431102	12	30	12	225.492	7.96	4200000	3000000	3100000	87.12973
Q6P5D8	Structural maintenance of chromosomes flexible hinge domain-containing protein 1 OS=Mus musculus GN=Smchd1 PE=1 SV=2		9.018435	15	30	15	225.506	7.24	4300000	3900000	3500000	78.60147
Q8VDD5	Myosin-9 OS=Mus musculus GN=Myh9 PE=1 SV=4		49.84694	102	455	81	226.232	5.66	96000000	1E+08	91000000	1613.106
O08638	Myosin-11 OS=Mus musculus GN=Myh11 PE=1 SV=1		9.584178	19	53	4	226.888	5.45	800000	34000000	1200000	175.6679
Q6URW6	Myosin-14 OS=Mus musculus GN=Myh14 PE=1 SV=1		10.7	16	37	10	228.446	5.55	13000000	14000000	20000000	129.5095
A2APX8	Sodium channel protein type 1 subunit alpha OS=Mus musculus GN=Scn1a PE=1 SV=1		0.647088	1	2	1	228.651	5.87	2400000		540000	5.993083
Q9R0L6	Pericentriolar material 1 protein OS=Mus musculus GN=Pcm1 PE=1 SV=2		3.703704	5	8	5	228.706	5.01	680000	620000	870000	27.10129
Q61879	Myosin-10 OS=Mus musculus GN=Myh10 PE=1 SV=2		19.23077	32	75	17	228.855	5.54	7200000	3400000	4000000	241.8192
Q7TSC1	Protein PRRC2A OS=Mus musculus GN=Prrc2a PE=1 SV=1		6.904541	9	13	9	229.063	9.39	1000000	1100000	1600000	37.64143
Q80YX1	Tenascin OS=Mus musculus GN=Tnc PE=1 SV=1		33.83886	46	161	46	231.659	4.89	27000000	23000000	20000000	529.0777
Q9JMH9	Unconventional myosin-XVIIIa OS=Mus musculus GN=Myo18a PE=1 SV=2		13.21951	21	39	21	232.611	6.28	5600000	4300000	4400000	114.8166
Q69ZN7	Myoferlin OS=Mus musculus GN=Myof PE=1 SV=2		8.59375	13	22	13	233.177	6.16	2900000	3100000	2200000	63.88479
Q8BIK4	Dedicator of cytokinesis protein 9 OS=Mus musculus GN=Dock9 PE=1 SV=2		3.552311	5	7	5	235.162	7.25	980000	760000	710000	18.66825

P49025	Citron Rho-interacting kinase OS=Mus musculus GN=Cit PE=1 SV=3		0.778589	1	1	1	235.242	6.54				3.20767
Q9Z1T6	1-phosphatidylinositol 3-phosphate 5-kinase OS=Mus musculus GN=Pikfyve PE=1 SV=3		0.858369	1	1	1	236.727	6.68		820000		3.092295
E9Q3L2	Phosphatidylinositol 4-kinase alpha OS=Mus musculus GN=Pi4ka PE=1 SV=2		2.327791	4	6	4	236.889	7.06	1500000	350000	800000	15.0947
Q8K284	General transcription factor 3C polypeptide 1 OS=Mus musculus GN=Gtf3c1 PE=1 SV=2		0.951928	1	3	1	237.326	7.25	520000	880000	450000	11.09026
Q9QY06	Unconventional myosin-IXb OS=Mus musculus GN=Myo9b PE=1 SV=2		3.784295	5	12	5	238.685	8.63	1900000	1900000	1500000	43.41526
Q8C147	Dedicator of cytokinesis protein 8 OS=Mus musculus GN=Dock8 PE=1 SV=4		2	4	8	3	238.826	6.96	1500000	890000	750000	25.85213
Q8R1A4	Dedicator of cytokinesis protein 7 OS=Mus musculus GN=Dock7 PE=1 SV=3		7.464789	13	25	12	241.286	6.71	1700000	1900000	2300000	69.51891
A2BH40	AT-rich interactive domain-containing protein 1A OS=Mus musculus GN=Arid1a PE=1 SV=1		2.890933	4	8	4	241.939	6.68	360000	1100000	1100000	27.45467
Q8K4L3	Supervillin OS=Mus musculus GN=Svil PE=1 SV=1		2.02765	3	5	3	243.011	6.87	680000	580000	440000	12.81672
B2RQC6	CAD protein OS=Mus musculus GN=Cad PE=1 SV=1		5.52809	8	16	8	243.084	6.43	3200000	3100000	2000000	52.45703
Q6P4T2	U5 small nuclear ribonucleoprotein 200 kDa helicase OS=Mus musculus GN=Snrnp200 PE=1 SV=1		16.94757	31	78	31	244.392	6.06	9300000	9600000	9400000	237.5527
A2AGH6	Mediator of RNA polymerase II transcription subunit 12 OS=Mus musculus GN=Med12 PE=1 SV=1		0.547945	1	1	1	244.407	7.17				2.691059
Q8BZN6	Dedicator of cytokinesis protein 10 OS=Mus musculus GN=Dock10 PE=1 SV=3		1.953488	3	5	3	245.602	7.05		1100000	1100000	11.41499
Q8C9B9	Death-inducer obliterator 1 OS=Mus musculus GN=Dido1 PE=1 SV=4		5.141844	6	9	6	247.024	7.91	740000	690000	900000	27.97818
Q8CJF7	Protein ELYS OS=Mus musculus GN=Ahctf1 PE=1 SV=1		5.260811	7	12	7	247.493	6.55	1000000	1400000	1500000	45.76684
Q5D862	SWISS-PROT:Q5D862 Tax_Id=9606 Gene_Symbol=FLG2 Filaggrin-2	x	8.657465	10	20	10	247.928	8.31	2800000	210000	15000000	69.15583
E9PZJ8	Activating signal cointegrator 1 complex subunit 3 OS=Mus musculus GN=Ascc3 PE=1 SV=1		2.320291	4	9	4	250.398	7.02	2500000	1900000	1800000	27.06479
P83741	Serine/threonine-protein kinase WNK1 OS=Mus musculus GN=Wnk1 PE=1 SV=2		0.546908	1	3	1	250.779	6.43	2800000	1800000	440000	11.0951
Q8VHY0	Chondroitin sulfate proteoglycan 4 OS=Mus musculus GN=Cspg4 PE=1 SV=3		13.70864	24	63	24	252.153	5.44	9500000	8300000	8500000	199.358
Q71LX4	Talin-2 OS=Mus musculus GN=Tln2 PE=1 SV=3		2.021053	4	8	1	253.462	5.8	260000			26.49091
B9EKR1	Receptor-type tyrosine-protein phosphatase zeta OS=Mus musculus GN=Ptprz1 PE=1 SV=1		4.108997	7	16	7	254.247	4.88	8700000	4400000	6500000	51.17191
Q91VW5	Golgin subfamily A member 4 OS=Mus musculus GN=Golga4 PE=1 SV=2		0.670241	1	1	1	257.406	5.36	410000			2.509235
Q924A2	Protein capicua homolog OS=Mus musculus GN=Cic PE=1 SV=2		0.63745	1	3	1	257.972	8.02	810000	860000	850000	7.934527
Q69ZK6	Probable JmjC domain-containing histone demethylation protein 2C OS=Mus musculus GN=Jmjd1c PE=1 SV=3		1.446809	3	4	3	260.479	8.07	750000	710000	1200000	9.02519
B2RWS6	Histone acetyltransferase p300 OS=Mus musculus GN=Ep300 PE=1 SV=2		1.741294	2	4	2	263.136	8.54	580000	430000	740000	13.62265
Q5SWU9	Acetyl-CoA carboxylase 1 OS=Mus musculus GN=Acaca PE=1 SV=1		1.705757	4	6	4	265.088	6.39	1100000	1300000	860000	15.79
Q9QX47	Protein SON OS=Mus musculus GN=Son PE=1 SV=2		8.592471	14	32	14	265.483	5.6	4700000	4700000	4700000	97.40676
Q6PR54	Telomere-associated protein RIF1 OS=Mus musculus GN=Rif1 PE=1 SV=2		4.754031	8	13	8	266.063	5.57	1100000	1400000	1200000	35.68764
Q6ZQ08	CCR4-NOT transcription complex subunit 1 OS=Mus musculus GN=Cnot1 PE=1 SV=2		4	8	15	8	266.637	7.11	2500000	2800000	2400000	39.30966
Q9WU42	Nuclear receptor corepressor 2 OS=Mus musculus GN=Ncor2 PE=1 SV=3		0.687702	1	3	1	269.642	7.44	2000000	2900000	1700000	7.439073
P26039	Talin-1 OS=Mus musculus GN=Tln1 PE=1 SV=2		20.58245	40	110	37	269.653	6.18	11000000	12000000	9900000	352.9644
P14873	Microtubule-associated protein 1B OS=Mus musculus GN=Map1b PE=1 SV=2		21.30682	34	84	33	270.089	4.83	9400000	17000000	5900000	266.6753
Q64512	Tyrosine-protein phosphatase non-receptor type 13 OS=Mus musculus GN=Ptpn13 PE=1 SV=2		1.222992	2	3	2	270.167	6.37	650000		590000	6.475545
Q60974	Nuclear receptor corepressor 1 OS=Mus musculus GN=Ncor1 PE=1 SV=1		2.03832	3	3	3	270.477	6.93		630000	220000	7.596198
P19096	Fatty acid synthase OS=Mus musculus GN=Fasn PE=1 SV=2		12.85942	26	50	26	272.257	6.58	6900000	6700000	6200000	145.2928
P11276	Fibronectin OS=Mus musculus GN=Fn1 PE=1 SV=4		27.93702	51	190	51	272.368	5.59	70000000	74000000	64000000	616.8636
Q99PV0	Pre-mRNA-processing-splicing factor 8 OS=Mus musculus GN=Prpf8 PE=1 SV=2		13.40471	27	59	27	273.443	8.84	8100000	9500000	9900000	164.7492
F6ZDS4	Nucleoprotein TPR OS=Mus musculus GN=Tpr PE=1 SV=1		15.05553	30	68	30	273.824	5.03	6700000	5600000	6300000	215.6776
Q99NH0	Ankyrin repeat domain-containing protein 17 OS=Mus musculus GN=Ankrd17 PE=1 SV=2		3.419132	7	14	7	274.043	6.52	4000000	3300000	3500000	35.69546
Q62261	Spectrin beta chain, non-erythrocytic 1 OS=Mus musculus GN=Sptbn1 PE=1 SV=2		39.6953	77	229	77	274.052	5.58	25000000	21000000	21000000	696.7681
Q80X90	Filamin-B OS=Mus musculus GN=Flnb PE=1 SV=3		29.01614	57	166	50	277.651	5.71	16000000	16000000	14000000	515.4468
Q61687	Transcriptional regulator ATRX OS=Mus musculus GN=Atrx PE=1 SV=3		11.14701	22	48	22	278.414	6.68	6300000	6700000	6500000	144.2938
Q80SU7	Interferon-induced very large GTPase 1 OS=Mus musculus GN=Gvin1 PE=1 SV=1		10.87763	24	53	24	280.637	6.57	6200000	8600000	7900000	139.2073
Q8BTM8	Filamin-A OS=Mus musculus GN=Flna PE=1 SV=5		40.4609	75	235	69	281.046	6.04	41000000	42000000	39000000	761.6833
Q86YZ3	SWISS-PROT:Q86YZ3 Tax_Id=9606 Gene_Symbol=HRNR Hornerin	x	16.17544	13	34	13	282.228	10.04	2900000	360000	13000000	105.0293
P16546	Spectrin alpha chain, non-erythrocytic 1 OS=Mus musculus GN=Sptan1 PE=1 SV=4		42.27346	92	257	92	284.422	5.33	32000000	24000000	23000000	844.848
E9Q5F9	Histone-lysine N-methyltransferase SETD2 OS=Mus musculus GN=Setd2 PE=1 SV=1		1.340166	2	2	2	285.486	6.3		370000		6.324831
Q9JLN9	Serine/threonine-protein kinase mTOR OS=Mus musculus GN=Mtor PE=1 SV=2		0.706159	1	3	1	288.605	7.17	1200000	1000000	720000	9.624419
Q69ZR2	E3 ubiquitin-protein ligase HECTD1 OS=Mus musculus GN=Hectd1 PE=1 SV=2		1.680672	3	5	3	289.905	5.41	960000	460000	280000	12.88612
P70398	Probable ubiquitin carboxyl-terminal hydrolase FAF-X OS=Mus musculus GN=Usp9x PE=1 SV=2		7.033998	13	27	13	290.526	5.87	2800000	2400000	2500000	80.49914
Q8VHX6	Filamin-C OS=Mus musculus GN=Flnc PE=1 SV=3		12.76596	27	66	19	290.937	5.95	15000000	13000000	8900000	196.5513
Q80YV3	Transformation/transcription domain-associated protein OS=Mus musculus GN=Trrap PE=1 SV=2		0.545809	1	2	1	291.37	8.48		660000	840000	4.885284
E9PVA8	eIF-2-alpha kinase activator GCN1 OS=Mus musculus GN=Gcn1 PE=1 SV=1		4.941969	11	24	11	292.834	7.36	2300000	2600000	2700000	65.53488

Q8BTI8	Serine/arginine repetitive matrix protein 2 OS=Mus musculus GN=Srrm2 PE=1 SV=3		9.100999	16	47	16	294.666	12.03	11000000	10000000	7800000	129.8407
Q9QYR6	Microtubule-associated protein 1A OS=Mus musculus GN=Map1a PE=1 SV=2		10.1585	20	42	19	299.957	5	7200000	4000000	3900000	119.431
E9Q394	A-kinase anchor protein 13 OS=Mus musculus GN=Akap13 PE=1 SV=1		0.396254	1	3	1	303.786	5.38	570000	670000	640000	7.837524
Q9Z329	Inositol 1,4,5-trisphosphate receptor type 2 OS=Mus musculus GN=Itpr2 PE=1 SV=4		4.035542	6	12	6	307.278	6.44	1900000	1300000	1600000	40.92409
Q3TLH4	Protein PRRC2C OS=Mus musculus GN=Prrc2c PE=1 SV=3		6.711174	11	26	11	310.703	9.1	3200000	3500000	3200000	79.02741
Q61554	Fibrillin-1 OS=Mus musculus GN=Fbn1 PE=1 SV=2		30.38636	60	220	59	312.083	4.92	25000000	26000000	26000000	627.581
P11881	Inositol 1,4,5-trisphosphate receptor type 1 OS=Mus musculus GN=Itpr1 PE=1 SV=2		3.965078	7	13	7	312.968	6.04	2000000	1800000	2000000	43.51667
Q61555	Fibrillin-2 OS=Mus musculus GN=Fbn2 PE=1 SV=2		4.712762	10	21	9	313.601	4.84	3800000	2000000	1800000	57.40481
Q6KCD5	Nipped-B-like protein OS=Mus musculus GN=Nipbl PE=1 SV=1		2.037169	4	10	4	315.253	7.91	940000	760000	910000	31.27625
Q9ESE1	Lipopolysaccharide-responsive and beige-like anchor protein OS=Mus musculus GN=Lrba PE=1 SV=1		1.995798	3	3	2	316.863	5.69			490000	7.055546
Q5XG71	Small subunit processome component 20 homolog OS=Mus musculus GN=Utp20 PE=1 SV=2		2.51076	6	9	6	317.542	8.09	1500000	850000	780000	25.02249
Q04690	Neurofibromin OS=Mus musculus GN=Nf1 PE=1 SV=1		0.457585	1	1	1	319.391	7.39	340000			2.547549
Q9EPN1	Neurobeachin OS=Mus musculus GN=Nbea PE=1 SV=1		0.919619	2	2	1	326.536	6.2	480000			4.799632
E9Q6J5	Biorientation of chromosomes in cell division protein 1-like 1 OS=Mus musculus GN=Bod1l PE=1 SV=1		7.124011	13	24	13	327.254	5.33	1300000	1200000	1300000	64.83739
Q5SSH7	Zinc finger ZZ-type and EF-hand domain-containing protein 1 OS=Mus musculus GN=Zzef1 PE=1 SV=2		0.512996	1	1	1	328.102	6.11	400000			2.41324
E9QAM5	Helicase with zinc finger domain 2 OS=Mus musculus GN=Helz2 PE=1 SV=1		0.305395	1	1	1	331.352	8.07		19000000		2.312547
E9Q557	Desmoplakin OS=Mus musculus GN=Dsp PE=1 SV=1		5.861949	16	21	16	332.706	6.8	3500000		13000000	56.0923
A2AJK6	Chromodomain-helicase-DNA-binding protein 7 OS=Mus musculus GN=Chd7 PE=1 SV=1		0.334896	1	1	1	333.851	6.47			230000	2.259637
Q6PNC0	DmX-like protein 1 OS=Mus musculus GN=Dmxl1 PE=1 SV=1		0.564222	1	1	1	335.796	6.42	560000			2.838331
Q8CHI8	E1A-binding protein p400 OS=Mus musculus GN=Ep400 PE=1 SV=3		0.520833	1	3	1	336.97	9.14	340000	370000	76000	8.366226
P19137	Laminin subunit alpha-1 OS=Mus musculus GN=Lama1 PE=1 SV=1		1.297017	2	3	2	337.954	6.7		350000	150000	8.674555
Q8BPN8	DmX-like protein 2 OS=Mus musculus GN=Dmxl2 PE=1 SV=3		0.824538	2	2	2	337.995	6.42	2000000			5.608686
E9Q8I9	Protein furry homolog OS=Mus musculus GN=Fry PE=1 SV=1		0.529801	1	2	1	338.877	6.01		690000	760000	5.354158
Q60847	Collagen alpha-1(XII) chain OS=Mus musculus GN=Col12a1 PE=2 SV=3		20.89744	52	144	52	340.004	5.64	18000000	21000000	17000000	414.4459
Q9ERU9	E3 SUMO-protein ligase RanBP2 OS=Mus musculus GN=Ranbp2 PE=1 SV=2		15.03439	31	67	31	340.907	6.18	4400000	5600000	5000000	215.2698
Q60675	Laminin subunit alpha-2 OS=Mus musculus GN=Lama2 PE=1 SV=2		4.00898	10	20	10	343.593	6.09	1300000	1800000	1900000	55.15971
P42859	Huntingtin OS=Mus musculus GN=Htt PE=1 SV=2		1.923693	4	7	4	344.471	6.29	380000	930000	710000	18.64428
Q0KL02	Triple functional domain protein OS=Mus musculus GN=Trio PE=1 SV=3		4.190845	7	13	7	347.643	6.35	1900000	1500000	1500000	36.37343
Q62388	Serine-protein kinase ATM OS=Mus musculus GN=Atm PE=1 SV=2		0.782779	2	2	2	349.195	6.86		270000		4.934385
E9PVX6	Proliferation marker protein Ki-67 OS=Mus musculus GN=Mki67 PE=1 SV=1		16.36764	33	72	33	350.65	9.72	3500000	4400000	4300000	218.2965
Q91ZI0	Cadherin EGF LAG seven-pass G-type receptor 3 OS=Mus musculus GN=Celsr3 PE=2 SV=2		0.242351	1	1	1	358.253	6.6		190000		1.848692
Q5H8C4	Vacuolar protein sorting-associated protein 13A OS=Mus musculus GN=Vps13a PE=1 SV=1		0.442198	1	1	1	359.173	6.19			550000	1.949951
Q62059	Versican core protein OS=Mus musculus GN=Vcan PE=1 SV=2		6.374739	16	54	16	366.56	4.64	19000000	19000000	19000000	175.3965
Q05793	Basement membrane-specific heparan sulfate proteoglycan core protein OS=Mus musculus GN=Hspg2 PE=1 SV=1		20.23199	54	142	54	398.039	6.32	20000000	21000000	17000000	418.1166
Q61001	Laminin subunit alpha-5 OS=Mus musculus GN=Lama5 PE=1 SV=4		1.667563	4	9	4	403.792	6.73	550000	760000	620000	24.21896
Q8BKX6	Serine/threonine-protein kinase SMG1 OS=Mus musculus GN=Smg1 PE=1 SV=3		0.82012	2	4	2	409.511	6.4	1100000	760000	640000	10.42797
O88737	Protein bassoon OS=Mus musculus GN=Bsn PE=1 SV=4		2.714358	8	10	8	418.587	7.71	1300000	410000		28.03769
Q8BX70	Vacuolar protein sorting-associated protein 13C OS=Mus musculus GN=Vps13c PE=1 SV=2		0.800427	2	3	2	419.824	6.81	1200000	790000		8.15837
P11531	Dystrophin OS=Mus musculus GN=Dmd PE=1 SV=3		0.788472	2	3	2	425.566	5.94	650000	490000		9.037947
Q8C8R3	Ankyrin-2 OS=Mus musculus GN=Ank2 PE=1 SV=2		8.337609	22	48	22	425.999	5.17	6700000	3800000	2400000	147.4763
P20930	SWISS-PROT:P20930 Tax_Id=9606 Gene_Symbol=FLG Filaggrin	x	3.398178	7	8	7	434.922	9.25			4200000	26.14606
Q70FJ1	A-kinase anchor protein 9 OS=Mus musculus GN=Akap9 PE=1 SV=2		1.053463	3	4	3	435.943	5.03	680000	920000	370000	8.707236
Q7TMY8	E3 ubiquitin-protein ligase HUWE1 OS=Mus musculus GN=Huwe1 PE=1 SV=5		0.731094	2	2	2	482.332	5.22		800000	45000	6.08107
Q91ZX7	Prolow-density lipoprotein receptor-related protein 1 OS=Mus musculus GN=Lrp1 PE=1 SV=1		8.250825	24	48	24	504.411	5.36	2700000	2600000	2200000	147.6453
Q7TPH6	E3 ubiquitin-protein ligase MYCBP2 OS=Mus musculus GN=Mycbp2 PE=1 SV=2		1.231161	4	7	4	517.408	7.12	650000	960000	1100000	17.177
Q9JHU4	Cytoplasmic dynein 1 heavy chain 1 OS=Mus musculus GN=Dync1h1 PE=1 SV=2		18.64772	77	206	77	531.71	6.42	19000000	16000000	15000000	585.4603
O88738	Baculoviral IAP repeat-containing protein 6 OS=Mus musculus GN=Birc6 PE=1 SV=2		0.327735	1	3	1	531.833	6.07	790000	1000000	520000	10.78244
Q9QXS1	Plectin OS=Mus musculus GN=Plec PE=1 SV=3		33.06331	136	361	135	533.861	5.96	23000000	24000000	22000000	1083.489
Q9QYX7	Protein piccolo OS=Mus musculus GN=Pclo PE=1 SV=4		0.611681	2	2	2	550.496	6.51	660000			4.512348
A2AN08	E3 ubiquitin-protein ligase UBR4 OS=Mus musculus GN=Ubr4 PE=1 SV=1		2.799228	11	15	11	571.927	6.06	1900000	1600000	1400000	42.88091
Q80W93	Hydrocephalus-inducing protein OS=Mus musculus GN=Hydin PE=1 SV=2		0.213426	1	1	1	581.154	6.38	4200000			1.877604
E9Q555	E3 ubiquitin-protein ligase RNF213 OS=Mus musculus GN=Rnf213 PE=1 SV=2		13.02407	55	124	55	584.411	6.8	9500000	9700000	8900000	364.8502
Q6ZWQ0	Nesprin-2 OS=Mus musculus GN=Syne2 PE=1 SV=2		1.338376	6	8	6	782.238	5.33	580000	440000	690000	25.02634

Q9QXZ0	Microtubule-actin cross-linking factor 1 OS=Mus musculus GN=Macf1 PE=1 SV=2		4.623334	26	56	25	831.362	5.43	2700000	9600000	1900000	160.3922
Q91ZU6	Dystonin OS=Mus musculus GN=Dst PE=1 SV=2		4.382524	24	48	24	833.701	5.31	2200000	1900000	2100000	143.9185
